# Hypoxia-Induced LIN28A mRNA Promotes the Metastasis of Colon Cancer in a Protein-Coding-Independent Manner

**DOI:** 10.3389/fcell.2021.642930

**Published:** 2021-02-16

**Authors:** Mingjiao Weng, Yukuan Feng, Yan He, Weiwei Yang, Jing Li, Yuanyuan Zhu, Tianzhen Wang, Chuhan Wang, Xiao Zhang, Yu Qiao, Qi Li, Lingyu Zhao, Shuangshu Gao, Lei Zhang, Yiqi Wu, Ran Zhao, Guangyu Wang, Zhiwei Li, Xiaoming Jin, Tongsen Zheng, Xiaobo Li

**Affiliations:** ^1^Department of Pathology, Harbin Medical University, Harbin, China; ^2^Key Laboratory of Heilongjiang Province for Cancer Prevention and Control, School of Basic Medicine, Mudanjiang Medical University, Mudanjiang, China; ^3^Department of Histology and Embryology, Harbin Medical University, Harbin, China; ^4^Department of Gastrointestinal Medical Oncology, Harbin Medical University Cancer Hospital, Harbin, China

**Keywords:** colon cancer, LIN28A, hypoxia, P-bodies, METAP2, metastasis

## Abstract

The hypoxic microenvironment is beneficial to the metastasis but not to the proliferation of cancer cells. However, the mechanisms regarding to hypoxia differentially regulating cancer metastasis and proliferation are largely unknown. In this study, we revealed that hypoxia induced the expression of LIN28A at mRNA level but segregated LIN28A mRNAs in the P-bodies and thus inhibits the production of LIN28A protein. This unexpected finding suggests that there may be non-coding role for LIN28A mRNA in the progression of colon cancer. We further showed that the non-coding LIN28A mRNA promotes the metastasis but not proliferation of colon cancer cells *in vitro* and *in vivo*. Mechanistically, we revealed that methionyl aminopeptidase 2 (METAP2) is one of the up-regulated metastasis regulators upon over-expression of non-coding LIN28A identified by mass spectrum, and confirmed that it is non-coding LIN28A mRNA instead of LIN28A protein promotes the expression of METAP2. Moreover, we demonstrated that knockdown of DICER abolished the promotional effects of non-coding LIN28A on the metastasis and METAP2 expression. Conclusively, we showed that hypoxia induces the production of LIN28A mRNAs but segregated them into the P-bodies together with miRNAs targeting both LIN28A and METAP2, and then promotes the metastasis by positively regulating the expression of METAP2. This study uncovered a distinctive role of hypoxia in manipulating the metastasis and proliferation by differently regulating the expression of LIN28A at mRNA and protein level.

## Introduction

Recent studies demonstrated that hypoxia is a common characteristic of solid malignant tumors and the hypoxic microenvironment is beneficial to cancer metastasis (Dhani et al., 2015). The RNA-binding protein LIN28A has been reported to be associated with poor prognosis and often up-regulated in a variety of malignant tumors (Viswanathan et al., 2009; Salmena et al., 2011; Li et al., 2012b; Wang et al., 2016a) and facilitate cancer metastasis (Wang et al., 2016b). However, it is undetermined if hypoxia promotes cancer metastasis by means of regulating the expression of LIN28A.

It has been demonstrated that gene expression at the mRNA level and the protein level is not parallel in cells (Gedeon and Bokes, 2012; Mertins et al., 2016) and that the post-transcriptional and translational regulations contribute to the different expressional patterns of genes at the mRNA and protein levels (Liu et al., 2016). Hypoxia and other cellular stresses induce the accumulation of the mRNA-processing bodies (P-bodies) in the cytoplasm, the cytoplasmic granules containing non-translating mRNAs toward degradation or translation repression (Andrei et al., 2005; Ferraiuolo et al., 2005; Liu et al., 2005; Teixeira et al., 2005). It is also undetected if the expression of LIN28A gene at the mRNA level and the protein level is parallel in cancer cells under hypoxia.

Additionally, it has been widely acknowledged that LIN28A promotes tumor progression by regulating the translation of its target mRNA and inhibiting the production of let-7 at the post-transcriptional level, a microRNA suppressing tumor progression potently, both mechanisms relying upon the RNA binding motif of LIN28A protein (Wang et al., 2015). The human LIN28A mRNA contains around 4000 nt, and the coding region only takes up about 15% of the full length of the mRNA, it is therefore possible if the human LIN28A mRNA served its functions in a protein-coding-independent manner. However, there have been no such reports documenting the non-coding function of LIN28A mRNA at the moment.

In this study, we examined the roles and molecular mechanisms of hypoxia in regulating the expression of LIN28A in colon cancer and explored the non-coding function and potential mechanisms of LIN28A mRNA in the progression of colon cancer.

## Materials and Methods

### Cell Lines and Colon Cancer Tissues

The human colon cancer cell lines HTC116, SW1116, and HCT15 were purchased from the Cell Lines Service (Cellcook Biotech Co., Ltd., Guangzhou, China) and authenticated by STR. The cells were cultured in 1640 and DMEM (Gibico, NY, United States) containing 10% fetal bovine serum (Gibico) with 100 IU/ml penicillin and 100 μ g/ml streptomycin at 37°C in a 5% CO_2_ humidified atmosphere. Cells were cultured under 1% O_2_ or treated with DFO at a final concentration of 130 μ mol/L to mimic the hypoxic environment (Wang and Semenza, 1993).

A total of 46 fresh colon cancer tissue samples were obtained from the Affiliated Tumor Hospital of Harbin Medical University between May 2015 and June 2016. The informed consent was signed by all patients enrolled in this study. This study was approved by the Harbin Medical University Institutional Ethnic Committee.

### Lentiviral Vector Construction

The pseudo lentiviruses were prepared and packaged as previously described (Li et al., 2012b). Briefly, the shRNA sequences targeting Dicer were synthesized and inserted into pLKO plasmid (Okada et al., 2019). The ORF of LIN28A was amplified by PCR and inserted into pLVX plasmid (Li et al., 2019). To over-express non-coding LIN28A mRNA, the full length of LIN28A cDNA was synthesis with translation initiation codon replacement (from ATG to TGA) and inserted into the 3′UTR of GFP in the pLVX-GFP plasmid. Then the constructed plasmid was co-transfected into the 293TN cells with packaging plasmids pMD2.G and pSPAX2. The supernatants were collected and used to infect colon cancer cells as previously described (Li et al., 2012b). The PCR primers and shRNA sequences were included in Supplementary Table 1.

### Transfection of siRNAs and miRNAs

The siRNAs targeting METAP2 and LIN28A, miR181a mimic, let-7 mimic, and negative control were synthesized by GenePharma (Suzhou, Zhejiang, China). The sequences of these miRNAs and siRNAs were included in Supplementary Table 1. The transfection was mediated by Lipofectamine 2000 (Invitrogen, United States) according to the manufacturer’s protocol.

### Mass Spectrum Assay

The proteins were digested with trypsin after quantified and mixed with the same amount of standard proteins (GST and MBP). The resulting peptides were dissolved in 0.1% formic acid and loaded into EASY-nLC 1000 system (Thermo Scientific, Waltham, MA, United States) and chromatographed by elution with a linear gradient of 6% to 80% of acetonitrile in 0.1% formic acid for 40 min. The separated peptides were analyzed in a Q Exactive^TM^ Plus mass spectrometer (Thermo Scientific, MA, United States) and searched in the Swiss-Prot database using the MASCOT 2.3 search engines.

### Total RNA Extraction and Real-Time qPCR Assays

Total RNA was extracted from tissues or cells with Trizol reagent (Invitrogen, United States). RNA was reverse-transcribed into cDNA using reverse Transcriptase M-MLV (Takara, Dalian, China). The relative expression of genes was detected with SYBR Green PCR Mix (Bioresearcher, Beijing, China). Primer sequences were summarized in Supplementary Table 1.

### Western Blot Analysis

Total protein was extracted from tissues or cells using RIPA buffer. 40 μg of total protein were separated by SDS-PAGE and then transferred onto PVDF membranes (Bio-Rad, Hercules, CA, United States). Membranes were blocked and then incubated with rabbit anti-HIF1 alpha (GeneTex Inc., CA, United States); rabbit anti-LIN28A (Abcam, Cambridge, United Kingdom); rabbit anti-METAP2 (Abcam); mouse anti-β-actin (ZSGB-BIO, Beijing, China) or mouse anti-GAPDH (Proteintech, IL, United States) antibodies at 4°C overnight. After incubated with secondary antibody, the detected proteins were visualized by ECL enhanced chemiluminescence detection system (Thermo Scientific, Rockford, IL, United States).

### Cell Proliferation Assay

Colorectal cancer cells were plated in 96-well plates at a density of 2 × 10^4^ cells/well. At 0, 24, 48, and 72 h, 150 μl CellTiter-Glo reagent (Biofroxx, Germany) was added into each well and mixed thoroughly. Following incubation for 10 min at room temperature, the luminometer was used to assess the luminescence signal according to the manufacturer’s protocol.

### Invasion and Migration Assay

Invasion and migration of colon cancer cells were detected by using *trans*-well chambers with and without matrigel (Corning Incorporated, Corning, NY, United States), respectively. Briefly, 8 × 10^4^ cells/ml of tumor cell suspension was prepared separately in serum-free medium, and 0.5 ml cell suspension was inoculated separately to a pore size of 8.0 μm chamber. Then 0.75 ml of complete culture was added into each 24-well plate containing the chamber. After 24 h, the chamber was removed, wiped off the cells on the filter, and fixed with crystal violet. The number of cells across the filter was counted. Wound healing experiment was also used for assessing the migration of cancer cells. Briefly, the HCT116 or SW1116 cells were seed in 6-well plate and cultured until confluent. A pipette tip was used to make a straight scratch on the confluent cells in each well. The cells were continued to culture after changed the medium and the scratch wound healing were recorded at the designed time points.

### Immunofluorescence Assay

HCT116 and SW1116 cells were seeded in a 24-well plate and cultured under normoxia and hypoxia for 24 h. The cells were fixed with 4% paraformaldehyde for 30 min, permeabilized with 0.1% Triton X-100 for 10 min at RT, blocked with goat serum for 30 min and incubated with mouse anti-RAP55 antibody (Santa Cruz Biotechnology, CA, United States) at 4°C overnight to detect the P-bodies. After washing with PBS, the cells were incubated with the phycoerythrin-conjugated secondary antibody (Santa Cruz Biotechnology) at 37°C for 1 h.

### Dual-Luciferase Reporter Assay

Fragment of the promoter of LIN28A and promoter of EPO were amplified by using PCR (primer sequences are provided in Supplementary Table 1) and then cloned into PGL3-control vector (Promega, Madison, WI, United States), respectively. Then the renilla luciferase plasmid (as an internal control) and PGL3-LIN28A promoter or PGL3-EPO promoter were co-transfected into HEK293T cells. 24 h after transfection, cells were lysed, and then renilla and firefly luciferase activities were measured by Dual-Luciferase Reporter Assay System (Promega).

### Chromatin Immunoprecipitation (ChIP) Assay

Chromatin immunoprecipitation analysis was performed with the ChIP kit (Millipore Corporation, Billerica, MA, United States) as previously described (Li et al., 2012a). Briefly, 1 × 10^7^ colon cancer cells cultured in normoxia or hypoxia were collected and lysed with denaturing buffer after crosslinking by using 1% formaldehyde (Sigma-Aldrich). The chromatin was subsequently sheared by sonication, and the chromatin fraction immunoprecipitated overnight at 4°C with the anti-HIF1α antibody and the homotype antibody. The DNA was extracted for PCR amplification. The PCR amplification was performed for 30 cycles under standard reaction conditions with pre-designed primers, and the sequences of primers were listed in Supplementary Table 1.

### Extraction of P-Bodies

The P-bodies extraction was performed as previously described (Hubstenberger et al., 2017). Briefly, the colon cancer cells cultured under normoxia or hypoxia were scrapped in cold PBS. After centrifuge, the pellets were suspended in cold lysis buffer containing 65 U/mL RNaseOut ribonuclease inhibitor (Promega) and EDTA-free protease inhibitor cocktail (Roche Diagnostics, Meylan, France), and then lysates were spun at 200 × *g* for 5 min to remove nuclei. Supernatants were centrifuged at 10,000 × *g* for 7 min, and pellets were resuspended into 100 ul of lysis buffer with 80 Units of RNaseOut. 20 μl primary antibody against RAP55 (Santa Cruz Biotechnology) was added to supernatants and incubated for 2 h at 4°C before 20 μl of resuspended Protein A/G PLUS-Agarose (Santa Cruz Biotechnology) was added for an additional overnight incubation at 4°C on a rotating device. Finally, immunoprecipitations were collected by centrifugation at 3,000 rpm for 5 min and resuspended in 0.5 ml Trizol reagent after washed with cold PBS.

### Animal Experiments

Four-week-old male athymic nude mice were purchased from Vital River Laboratory (Beijing, China). For tumor growth assay, 1 × 10^6^ cells were injected subcutaneously into each nude mouse, and five mice were injected for each cell line. The animals were sacrificed at the end of the experiment, the tumor weight measured. For tumor metastasis assay, 1.5 × 10^6^ cancer cells were injected into the tail vein of each nude mouse, and eight mice were injected for each cell line. After the nude mice were sacrificed at the end of the experiment, the lungs were examined and fixed. Then the micrometastasis in lungs were detected by H&E staining. All proposals were approved and supervised by the institutional animal care and use committee of Harbin Medical University.

### Immunohistochemistry

The expression of HIF1A and Ki67 at protein level in the experimental lung metastatic colon cancer tissues was detected by immunohistochemistry. Briefly, 5-μm-thick tissue sections were prepared. After deparaffinization, rehydration, antigen retrieval and blocking with H_2_O_2_ and 5% bovine serum albumin, respectively, the sections were incubated with primary antibodies against HIF1α (GeneTex Inc.) or Ki67 (Daco, Glostrup, Denmark) at 4°C overnight. After incubation with secondary antibody for 1 h at room temperature, DAB substrate (ZSGB Bio, Beijing, China) was used for staining. The staining score was given and calculated by allying intensity with extent as previously described (Wang et al., 2016b).

### Statistical Analysis

Data are expressed as the mean ± standard deviation. Data were analyzed with Student’s *t*-test or one-way analysis of variance using GraphPad Prism (GraphPad Software, Inc., CA, United States). *P* < 0.05 was considered to indicate a statistically significant difference.

## Results

### Hypoxia Induces the Expression of LIN28A at the mRNA Level Rather Than the Protein Level

**FIGURE 1:**
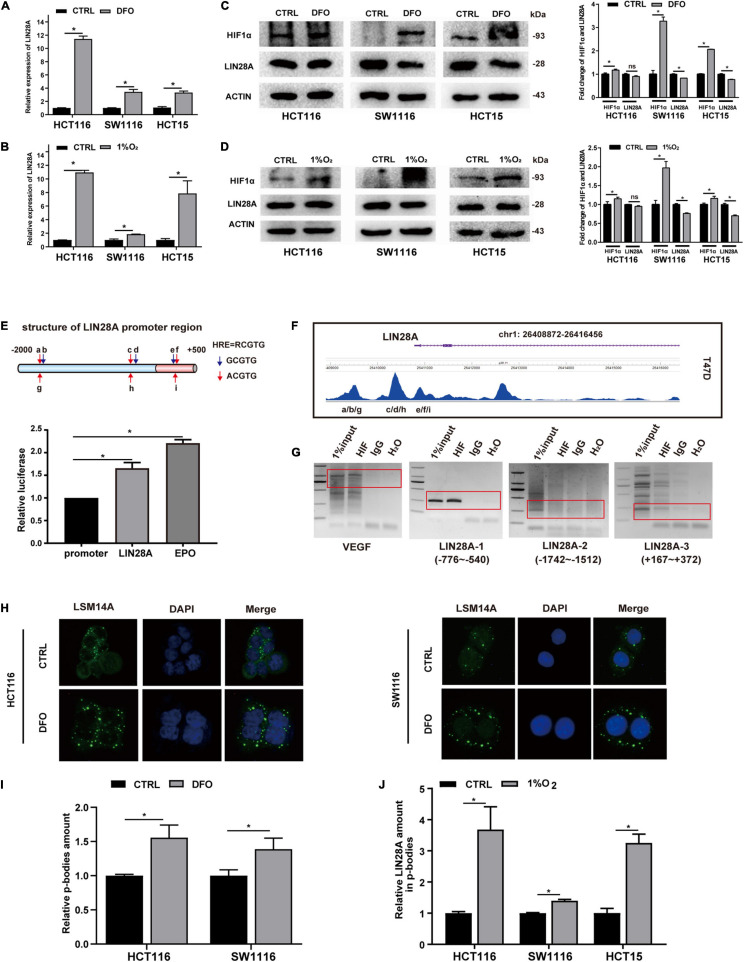
Hypoxia induces the expression of LIN28A mRNA but segregates LIN28A mRNA in the P-bodies. LIN28A mRNA level increased significantly in colon cancer cells treated with DFO **(A)** or cultured in 1% O_2_ **(B)**. LIN28A expression decreased at protein level when cells were treated with DFO **(C)** or cultured in 1% O_2_ **(D)**. **(E)** The promoter region of LIN28A gene contains functional HIF1α binding sites detected by dual-luciferase assay. **(F)** HIF1α directly binds to LIN28A promoter in T47D cell line based on the ChIP sequence data. **(G)** ChIP assay showed that HIF1α directly binds to LIN28A promoter in HCT116 cells (VEGF as positive control, LIN28A-2 as negative control). **(H)** Representative images of P-bodies in HCT116 and SW1116 cells by LSM14A staining with immunofluorescence (magnification: 400×; nucleus stained in blue with DAPI and P-bodies stained in green). **(I)** The number of P-bodies in HCT116 and SW1116 cells was counted by using ImageJ software, and the relative amount of P-bodies in colon cancer cells under hypoxia to normoxia was calculated. **(J)** The relative abundance of LIN28A mRNA in the P-bodies of colon cancer cells under hypoxia was detected by real time PCR. The data in the bar graphs in panels **(A,B,J)** were calculated as the mean ± SD from three independent experiments. Statistical significance was represented by *P < 0.05.

To determine the effect of hypoxia on LIN28A expression, we first examined the changes of LIN28A expression in colon cancer cells treated with DFO (Jung et al., 2017) or cultured in 1% O_2_. Real time PCR results showed that LIN28A mRNA was elevated in all cell lines detected upon DFO treatment or cultured in 1% O_2_ (Figures 1A,B). In contrast, detected by Western Blot, the expression of LIN28A protein was not altered (in HCT116 cells) accordingly or even decreased (in SW1116 and HCT15 cells) under both hypoxia models (Figures 1C,D). These results indicate that hypoxia induces LIN28A expression at the mRNA level rather than the protein level in colon cancer cells.

### Hypoxia Induces LIN28A Transcription but Segregates LIN28A mRNA in the P-Bodies

To explore the mechanism of hypoxia regulating LIN28A expression in colon cancer cells, we analyzed the promoter sequence of LIN28A gene and found several potential HIF1α binding sites (Figure 1E). Then we confirmed that there are functional HIF1α binding sites (5′-RCGTG-3′, R representing A or G) (Salvi and Thanabalu, 2017) within the 2500 bp region upstream of LIN28A transcriptional starting site by using luciferase reporter assay (Figure 1E). Moreover, by analyzing the published ChIP sequence data from breast cancer T47D cell line in Cistrome database^1^ (Schörg et al., 2015; Zhang et al., 2015), we found that HIF1α indeed binds to LIN28A *in vivo* (Figure 1F). Furthermore, we demonstrated that HIF1α binds to LIN28A in the colon cancer cells by ChIP assay (Figure 1G). These results indicate that the transcription factor HIF1α directly binds to the promoter of LIN28A and induces its transcription.

Having confirmed that HIF1α could promote LIN28A transcription, we turned our attention to the problem emerged next: why didn’t LIN28A protein elevate in synchronism with its mRNA under hypoxia? Upon cellular stresses such as hypoxia and infection, certain mRNAs are sequestered into P-bodies, their translations being suppressed or shut down (Teixeira et al., 2005; Tkach et al., 2012; Jain and Parker, 2013). Therefore, we detected and confirmed that hypoxia increased the number of P-bodies in colon cancer cells by immunofluorescence assay using LSM14A as the marker of P-bodies (Hubstenberger et al., 2017) (Figures 1H,I). Meanwhile, we examined the abundance of LIN28A mRNA in P-bodies and showed that hypoxia promoted the segregation of LIN28A mRNAs in P-bodies (Figure 1J). This observation may explain why LIN28A protein level was decreased in hypoxic conditions even though the transcription was elevated.

### LIN28A mRNA Promotes the Metastasis of Colon Cancer Cells in a Protein-Coding-Independent Manner

**FIGURE 2:**
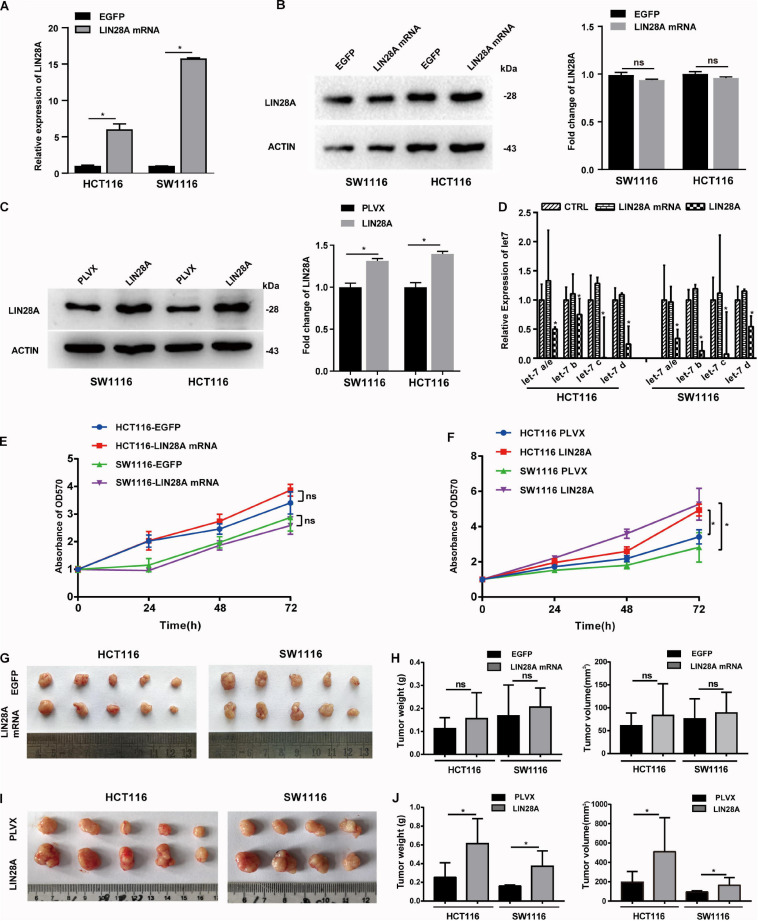
Non-coding LIN28A mRNA has no effect on the proliferation of colon cancer cells. **(A)** The over-expression of LIN28A mRNA in HCT116 cells and SW1116 cells was confirmed by qPCR. **(B)** LIN28A protein was detected by western blot in the full-length LIN28A mRNA exogenously over-expressing cells. **(C)** The over-expression of LIN28A protein was confirmed in HCT116 and SW1116 cells with LIN28A ORF over-expression by using western blot assay. **(D)** The expressional change of mature let-7 family miRNAs was detected by qPCR upon over-expression of LIN28A protein or non-coding LIN28A mRNA in HCT116 and SW1116 cells. **(E)** The effect of non-coding LIN28A mRNA on the proliferation of HCT116 and SW1116 cells *in vitro* detected by growth curve assay. **(F)** The effect of LIN28A protein on the proliferation of HCT116 and SW1116 cells *in vitro* detected by growth curve assay. **(G,H)** The effect of non-coding LIN28A mRNA on the growth of colon cancer cells in xenograft mice model by evaluating the weight and volume of tumor, respectively. **(I,J)** The effect of LIN28A protein on the growth of colon cancer cells in xenograft mice model by evaluating the weight and volume of tumor, respectively. The data in panels **(A,D)** were calculated as the mean ± SD from three independent experiments, and data in panels **(G–J)** are presented as the mean ± SD for each group. Statistical significance was represented by *P < 0.05.

In view of the fact that hypoxia only induced the mRNA expression of LIN28A, we hypothesized that the elevated LIN28A mRNA might promote the progression of colon cancer independent of its protein-coding function. To explore this hypothesis, we established stable colon cancer cell lines exogenously expressing the full length LIN28A mRNA lacking coding function by inserting it into the 3′UTR of GFP and replacing the translation initiation codon ATG with termination codon TGA. As a control, we also established stable cells exogenously expressing the ORF of LIN28A mRNA. We confirmed that the mRNA of LIN28A is over-expressed (Figure 2A), whereas LIN28A protein level is not altered (Figure 2B) in cancer cells exogenously expressing the full length of LIN28A mRNA. As expected, the over-expression of LIN28A ORF enhanced the production of LIN28A protein (Figure 2C) and thus suppressed the maturation of the let-7 family miRNAs (Figure 2D). By contrast, over-expression of the full length of LIN28A mRNA did not cause the decrease of let-7 family miRNAs (Figure 2D). These results suggest that we successfully over-expressed non-coding LIN28A mRNA in colon cancer cells.

**FIGURE 3:**
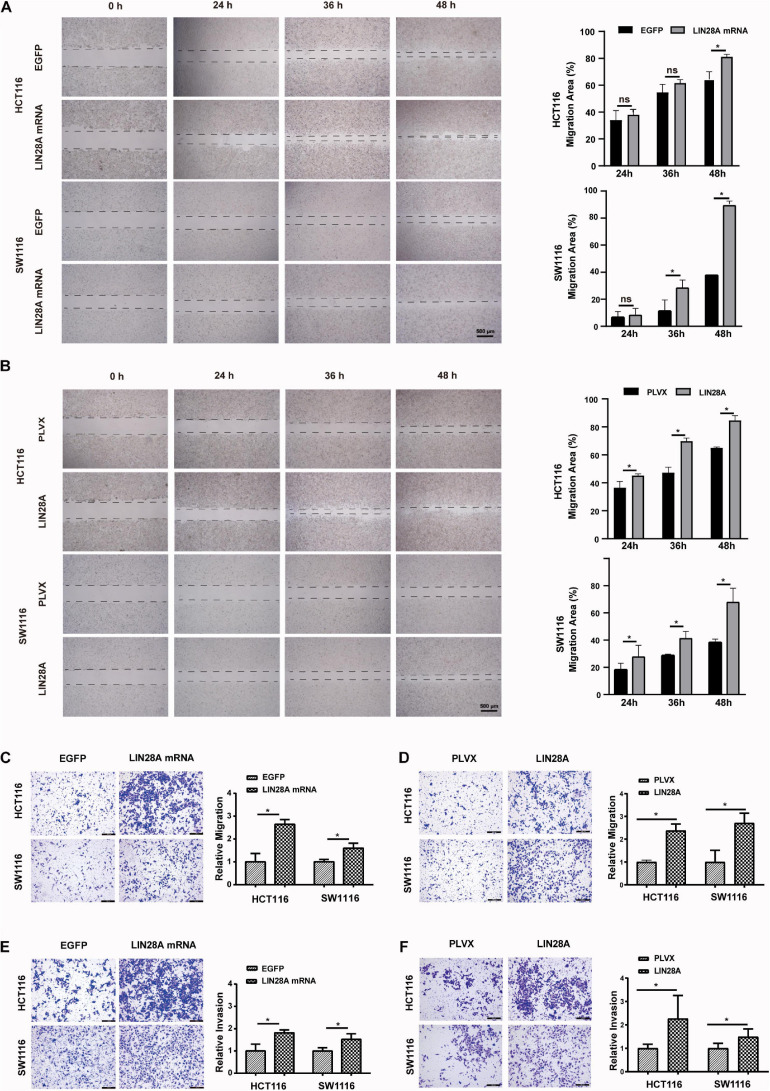
Non-coding LIN28A mRNA promotes migration and invasion of colon cancer cells. **(A)** Over-expression of non-coding LIN28A mRNA enhances the wound healing of HCT116 and SW1116 cells (Scale bars, 500 μm). **(B)** Over-expression of LIN28A protein promotes the wound healing of HCT116 and SW1116 cells (Scale bars, 500 μm). **(C)** Over-expression of non-coding LIN28A mRNA enhances the migration of HCT116 and SW1116 cells detected by transwell assay (magnification, 100×; Scale bars, 200 μm). **(D)** Over-expression of LIN28A protein promotes the migration of HCT116 and SW1116 cells detected by transwell assay (magnification, 100×; Scale bars, 200 μm). **(E)** The over-expression of non-coding LIN28A mRNA enhances the invasion of HCT116 and SW1116 cells detected by transwell assay (magnification, 100×; Scale bars, 200 μm). **(F)** The over-expression of LIN28A protein enhances the invasion of HCT116 and SW1116 cells detected by transwell assay (magnification, 100×; Scale bars, 200 μm). The data were based on the mean ± SD from three independent experiments, and the statistical significance was represented by *P < 0.05.

**FIGURE 4:**
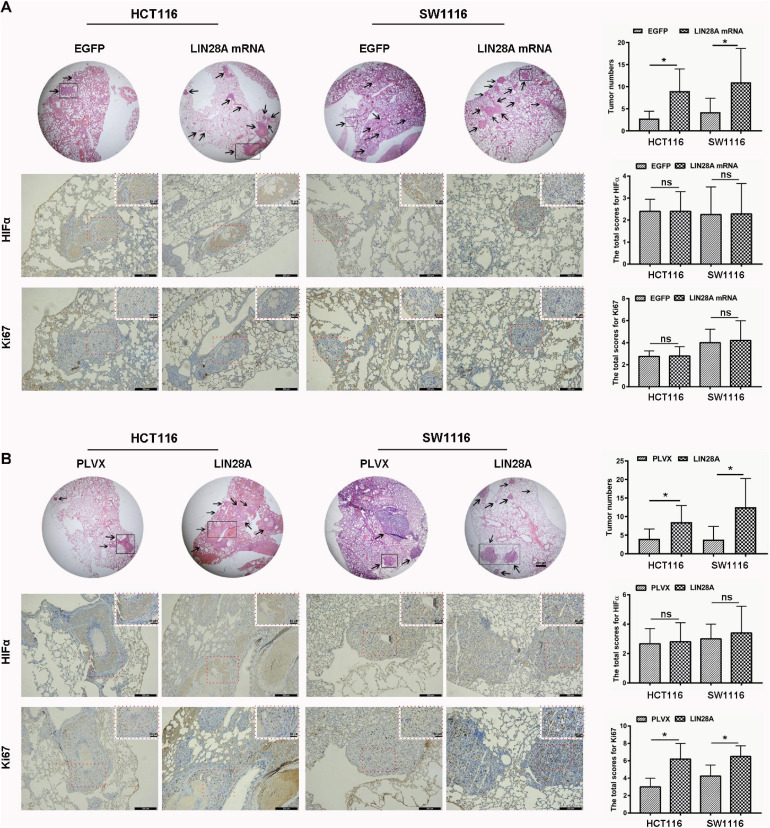
Non-coding LIN28A mRNA promotes lung metastasis of colon cancer. **(A)** Over-expression of non-coding LIN28A mRNA promotes lung metastasis of colon cancer cells *in vivo* by using tail vein injection model (magnification, 20×; Scale bars, 500 μm; the black arrow represents the location of the metastasis). The expression of HIF1α and Ki67 in metastatic cancer tissues was evaluated by using immunohistochemistry (Scale bars, 200 μm for low-power field; 50 μm for high-power field). **(B)** LIN28A protein promotes metastasis of colon cancer cells *in vivo* (magnification, 20×; Scale bars, 500 μm; the black arrow represents the location of the metastasis.). The expression of HIF1α and Ki67 in metastatic cancer tissues was evaluated by using immunohistochemistry (Scale bars, 200 μm for low-power field; 50 μm for high-power field). The statistical significance was represented by *P < 0.05.

Next, we investigated the effect of the exogenous over-expressed non-coding LIN28A mRNA on the growth of colon cancer cells. The results revealed that non-coding LIN28A mRNA had no effect on colon cancer growth *in vitro* (Figure 2E) and *in vivo* (Figures 2G,H). As a control, elevated LIN28A protein evidently promoted cancer growth both *in vitro* (Figure 2F) and *in vivo* (Figures 2I,J). Then we assessed the role of non-coding LIN28A mRNA in colon cancer metastasis. By using the wound healing assay and *trans*-well assay, we found that non-coding LIN28A mRNA promoted the migration and invasion of colon cancer cells *in vitro* significantly (Figures 3A,C,E). The non-coding LIN28A mRNA also enhanced the lung metastasis of colon cancer cells in a tail vein injection model (Figure 4A). As a control, enforced expression of LIN28A protein also accelerated tumor metastasis both *in vitro* and *in vivo* (Figures 3B,D,F, 4B). Moreover, we showed that LIN28A protein (Figure 4B) but not LIN28A mRNA (Figure 4A) increased the Ki67 level in experimental metastatic colon cancer tissues, which is consistent with that LIN28A mRNA does not affect the proliferation of colon cancer cells (Figures 2E,G,H). Collectively, we demonstrated that LIN28A mRNA facilitates colon cancer metastasis in a protein-coding-independent manner.

### Non-coding LIN28A mRNA Enhances the Expression of METAP2 in Colon Cancer Cells

**FIGURE 5:**
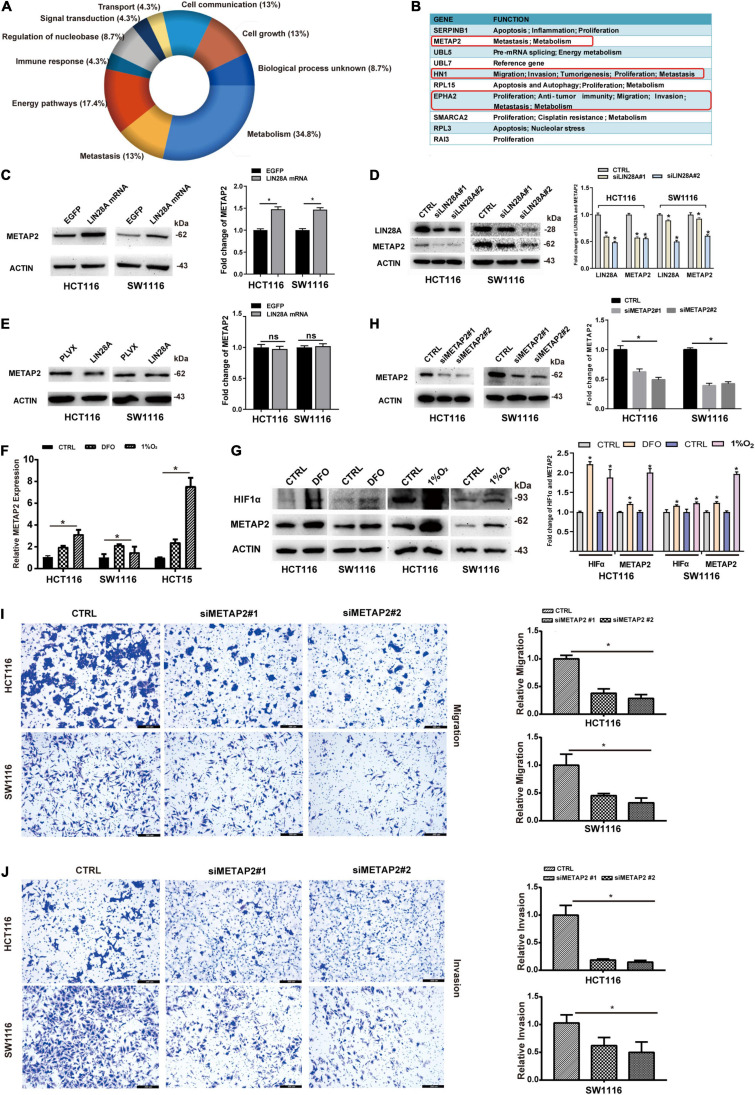
Non-coding LIN28A mRNA increases the expression of METAP2 in colon cancer cells. **(A)** Functional enrichment analysis of the proteins with altered expression corresponding to over-expression of non-coding LIN28A mRNA in SW1116 cells detected by MS. **(B)** The top 10 up-regulated proteins in LIN28A mRNA over-expressing cells are shown, and the potential metastasis regulators are highlighted. **(C)** The expression of METAP2 increased after over-expression of LIN28A mRNA, validated by Western blot assay. **(D)** The expression of METAP2 decreased after knockdown LIN28A, detected by Western Blot assay. **(E)** The over-expression of LIN28A protein does not affect the expression of METAP2 in colon cancer cells. The expression of METAP2 elevated under hypoxia at both the mRNA level **(F)** and the protein level **(G)**. **(H)** Knockdown of METAP2 was confirmed by Western blot assay. **(I)** The migration of colon cancer cells decreased upon METAP2 knockdown detected by using transwell assay (magnification, 100×; Scale bars, 200 μm). **(J)** The invasion of colon cancer cells decreased upon METAP2 knockdown detected by using transwell assay (magnification, 100×; Scale bars, 200 μm). The statistical significance was represented by *P < 0.05.

To investigate the underlying mechanism by which the non-coding LIN28A mRNA accelerates the colon cancer metastasis, we detected the expression change of protein profiles using MS after the over-expression of non-coding LIN28A mRNA in SW1116 cells. We identified 5171 proteins in total (Supplementary Table 2) and found that 30 of them significantly changed their expression levels upon the over-expression of non-coding LIN28A. Functional enrichment assay showed that these proteins with altered expressions are in the majority involved in metabolism and metastasis processes (Figure 5A). We next searched for the potential metastasis regulators among the top 10 up-regulated proteins and identified three candidates that could potentially promote cancer metastasis (Figure 5B). Seeing that METAP2 was the top candidate (Figure 5B), then we confirmed that over-expression of non-coding LIN28A mRNA significantly elevated the expression of METAP2 in both HCT116 and SW1116 cells (Figure 5C), whereas knockdown of LIN28A significantly down-regulated the expression of METAP2 (Figure 5D). However, over-expression of LIN28A protein did not change the METAP2 protein level in both cell lines (Figure 5E). These results confirmed that LIN28A promotes the expression of METAP2 in a protein-coding-independent manner. Considering that hypoxia induced the production of LIN28A mRNA, we also evaluated the impact of hypoxia on the METAP2 expression. As expected, both METAP2 mRNA and protein levels were increased upon hypoxia in either DFO treatment or 1% O_2_ culture condition (Figures 5F,G).

We further determined whether the roles of METAP2 in colon cancer are consistent with those of the non-coding LIN28A mRNA by assessing the effects of METAP2 on the migration and invasion of colon cancer cells. The results showed that METAP2 knockdown by siRNA in HCT116 and SW1116 cells (Figure 5H) significantly suppressed the invasion and migration of both cell lines (Figures 5I,J).

### Non-coding LIN28A mRNA Promotes the Expression of METAP2 Depending on miRNAs

**FIGURE 6:**
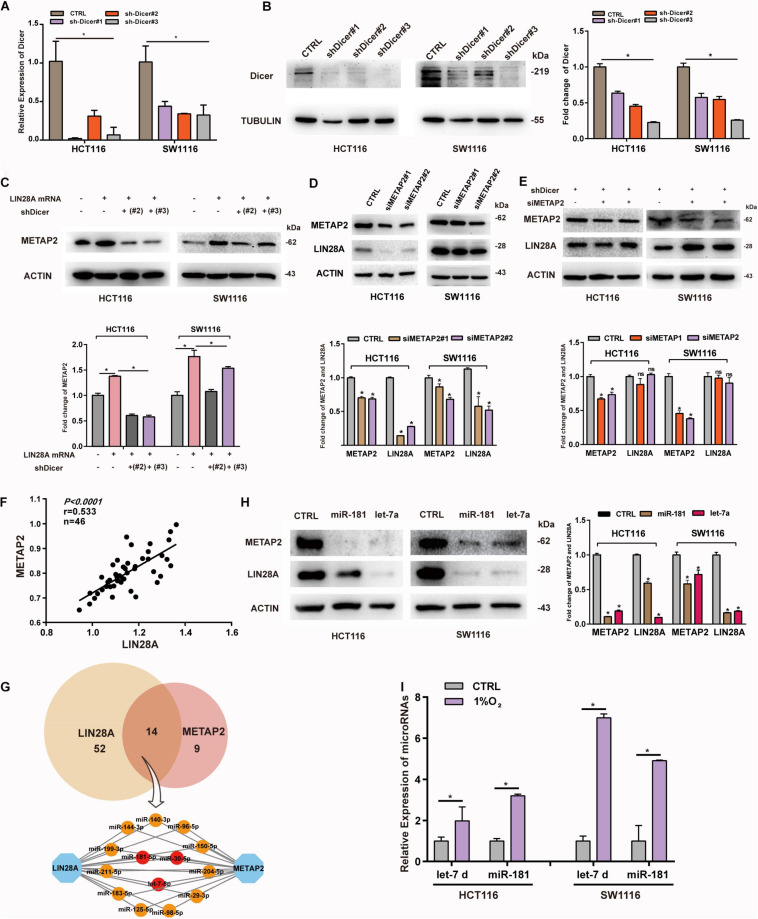
Non-coding LIN28A mRNA promotes the expression of METAP2 depending on miRNAs. **(A)** Knockdown of Dicer in HCT116 and SW1116 cells was confirmed by qPCR. **(B)** Knockdown of Dicer in HCT116 and SW1116 cells was detected by western blot. **(C)** LIN28A mRNA fails to promote the expression of METAP2 upon Dicer knockdown. **(D)** Knockdown of METAP2 decreases the expression of LIN28A. **(E)** Knockdown of METAP2 does not affect the expression of LIN28A upon Dicer knockdown. **(F)** The mRNA expression of LIN28A and METAP2 are positively correlated to each other (*r* = 0.533). **(G)** miRNAs target LIN28A mRNA and METAP2 mRNA. **(H)** miR-181 and let-7 inhibit the expression of LIN28A and METAP2 in colon cancer cell lines, respectively. **(I)** The relative abundance of let-7 and miR-181 in the p-bodies of colon cancer cells under hypoxia. The date in panels **(A,I)** were based on the mean ± SD from three independent experiments. The statistical significance was represented by *P < 0.05.

Previous studies suggested that mRNAs bind to miRNAs and are segregated into P-bodies upon stresses, and then promote the expressions of other mRNAs targeted by those miRNAs (Liu et al., 2005; Saito et al., 2011; Cui et al., 2012). These observations offer a potential explanation for the interaction between LIN28A and METAP2. To find out if non-coding LIN28A mRNA regulates the expression of METAP2 through sponging miRNAs in colon cancer, we established Dicer knockdown cell lines (Figure 6A,B) and then evaluated the impact of over-expressed non-coding LIN28A mRNA on the expression of METAP2. We showed that the non-coding LIN28A mRNA failed to enhance the expression of METAP2 upon Dicer knockdown (Figure 6C). Consistently, the expression of LIN28A decreased upon knockdown of METAP2 in colon cancer cells (Figure 6D), whereas this phenomenon was also abolished upon knockdown of Dicer (Figure 6E). Moreover, we detected the mRNA expression of LIN28A and METAP2 by using RT-PCR and revealed that the two were positively correlated with each other in 46 colon cancer tissues (*r* = 0.533, Figure 6F). These results suggest that non-coding LIN28A promotes the expression of METAP2 depending on miRNAs.

Considering the fact that both of LIN28A mRNA and METAP2 mRNA contain a long 3′UTR (2030 nt length for METAP2 and 3270 nt length for LIN28A), both mRNAs could be targeted by various miRNAs simultaneously. To identify miRNAs bridging these two mRNAs, we searched the TargetScan and identified 66 conserved miRNAs targeting LIN28A mRNA and 23 conserved miRNAs targeting METAP2 mRNA, respectively (Supplementary Table 3), among which 14 miRNAs can potentially target both LIN28A and METAP2 simultaneously. Among these miRNAs, let-7-5p, miR-181-5p and miR-30-5p are experimentally validated for their ability to target both of LIN28A and METAP2 (Yi et al., 2017; Chou et al., 2018) (Figure 6G). In this study, we chose let-7-5p and miR-181-5p for further validations in the colon cancer cells. As expected, we showed that both miRNAs significantly targeted the expression of LIN28A and METAP2 in two colon cancer cell lines (Figure 6H). Moreover, we detected the abundance of let-7 and miR-181 in the P-bodies upon hypoxia treatment and showed that hypoxia also induced the segregation of both miRNAs into P-bodies (Figure 6I). These results suggest that let-7 and miR-181 could mediate the regulation of METAP2 expression by non-coding LIN28A mRNA in colon cancer.

**FIGURE 7:**
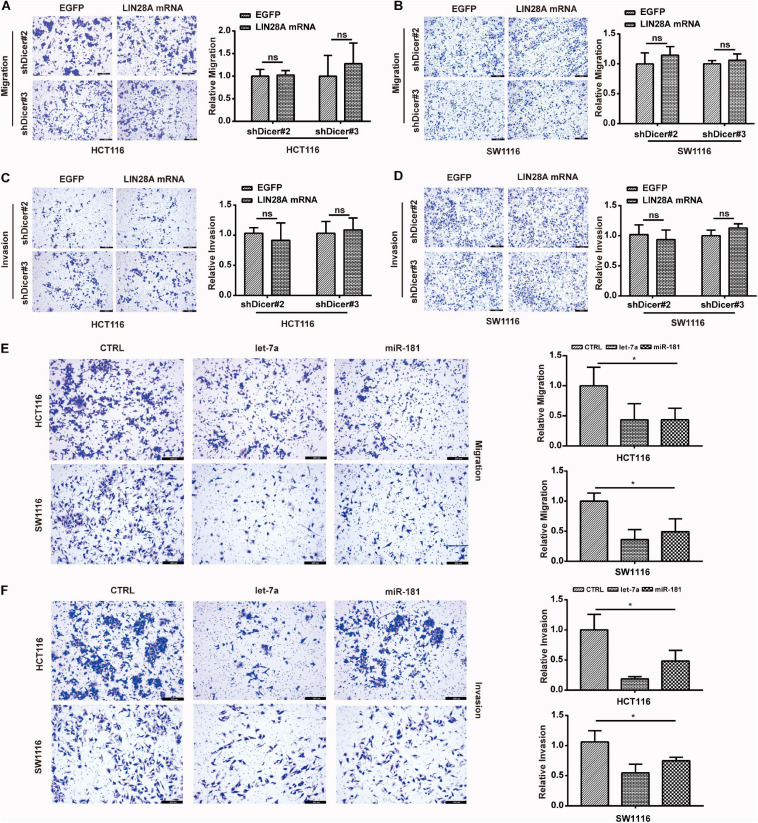
Non-coding LIN28A mRNA promotes the metastasis of colon cancer cells depending on miRNAs. **(A,B)** Knockdown of Dicer abolishes the promotion role of non-coding LIN28A mRNA in the migration of colon cancer cells detected by using transwell assay (magnification, 100×; Scale bars, 200 μm). **(C,D)** Knockdown of Dicer abolishes the promotion role of non-coding LIN28A mRNA in the invasion of colon cancer cells detected by using transwell assay (magnification, 100×; Scale bars, 200 μm). **(E)** miR-181 and let-7 inhibit the migration of colon cancer cells detected by using transwell assay (magnification, 100×; Scale bars, 200 μm). **(F)** miR-181 and let-7 suppress the invasion of colon cancer cells detected by using transwell assay (magnification, 100×; Scale bars, 200 μm).

Finally, we detected the impact of Dicer knockdown on the function of non-coding LIN28A mRNA in colon cancer cells. The *trans*-well assay results showed that knockdown of Dicer also abolished the promotional roles of non-coding LIN28A mRNA in terms of migration and invasion of colon cancer cells (Figures 7A–D). Additionally, we noticed that, contrary to non-coding LIN28A and METAP2, let-7 and miR-181 inhibited the migration and invasion of colon cancer cells (Figures 7E,F). These results suggest that the function of LIN28A facilitating colon cancer metastasis is also dependent on the presence of miRNAs, such as miR-181 and let-7.

## Discussion

RNA-binding protein LIN28A is often elevated in various cancer types. However, few studies investigated the mechanisms concerning LIN28A over-expression in cancers. In this study, we were able to demonstrate for the first time that hypoxia induced the expression of LIN28A in cancers. Unexpectedly, we also discovered that hypoxia only induced the expression of LIN28A at the transcriptional level. How hypoxia differentially regulates the expression of LIN28A at different levels is a fascinating question, and the significance of this regulation in cancer progression is an attractive topic. In this study, we confirmed that hypoxia increased the number and size of P-bodies in the colon cancer cells and that the relevant abundance of LIN28A mRNA in the P-bodies was also increased. These observations suggest that, though not exclusively, the segregation of LIN28A mRNA in the P-bodies was responsible for hypoxia differentially regulating the expression of LIN28A at the mRNA and the protein levels.

A previous study reported that epithelial morphology maintaining factor E-cadherin did not express in HCCs at the protein level, yet it abundantly expressed at the mRNA level (Ghafoory et al., 2015). By analyzing the expression of E-cadherin mRNA with *in situ* hybridization (ISH), Ghafoory et al. (2015) found that E-cadherin mRNA was located in the nuclei of HCCs, yet it was present in the cytoplasm of adjacent normal cells. By using immunohistochemistry staining, they revealed that E-cadherin protein is aberrant in HCCs but detected in the cell membrane of normal cells, suggesting that segregation of E-cadherin in the nuclei contributes to the loss of E-cadherin in HCCs (Ghafoory et al., 2015). A rich body of studies suggests that hypoxia induces the loss of E-cadherin in the cell membrane of some cancer cells (Howard et al., 2005; Sun et al., 2009; Hongo et al., 2013). Interestingly, they revealed that a higher level of HIF1α mRNA in the cancerous tissues is associated with stronger E-cadherin mRNA in the nuclei detected by ISH co-staining (Ghafoory et al., 2015), suggesting that hypoxia may suppress the expression of E-cadherin protein in HCCs by inducing the segregation of E-cadherin mRNA in the nuclei. However, the detailed molecular mechanisms underlying this phenomenon are not investigated. In this study, we showed that hypoxia induces the segregation of LIN28A mRNA in the P-bodies instead of in the nuclei, but the consequent effects of hypoxia on the protein expression of E-cadherin and LIN28A are similar. We proposed that translocation of RNA binding proteins under hypoxia may contribute to hypoxia inducing different mRNAs to be sequestered into different organelle of the cells.

Hypoxia suppresses the expression of E-cadherin protein in cancer cells and enhances the survival of cancer cells (Chu et al., 2013). On the other hand, hypoxia also promotes the epithelial-mesenchymal transition (EMT) and then facilitates cancer metastasis (Sun et al., 2016; Wigerup et al., 2016). LIN28A is a master oncogene and promotes both metastasis and proliferation of cancer cells (Wang et al., 2015; Zhang et al., 2018). In this study, we also confirmed the positive roles of LIN28A protein in the proliferation and metastasis of colon cancer cells. However, we established that non-coding LIN28A mRNA only promotes the metastasis and has no noticeable effect on the proliferation of colon cancer cells. Consequently, the fact that hypoxia only induces the production of LIN28A mRNA in colon cancer cells is consistent with the observation that the hypoxic microenvironment is beneficial to the metastasis but not to the proliferation of cancer cells (Sprague et al., 2006; Wigerup et al., 2016).

It has been well acknowledged that the miRNAs play important roles in regulating gene expression at the post-transcriptional and/or translational levels. It has been believed that the miRNAs regulate gene expression in a simple “miRNA→mRNA→protein” pattern. However, recent studies suggest that miRNA activity can be regulated by “target mimics,” and miRNA–mRNA interactions are bilateral instead of unilateral (Ebert et al., 2007; Franco-Zorrilla et al., 2007; Salmena et al., 2011; Yang et al., 2016). By knocking down the expression of the Dicer-1 gene and subsequently abolishing the miRNA maturation, we demonstrated that non-coding LIN28A mRNA functions as “miRNA sponges” to promote the metastasis of colon cancer cells. We then used MS to identify the target genes regulated by LIN28A mRNA, and confirmed that METAP2 is one of the target genes of LIN28A mRNA at both the expressional and the functional levels. In spite of the fact that only two well-validated miRNAs (let-7 and miR-181) were selected as representatives in this study, there must be other miRNAs mediating the interactions between LIN28A and METAP2, considering that hundreds of miRNAs are predicted to simultaneously target both LIN28A and METAP2.

Conclusively, we showed that hypoxia differentially regulates the expression of LIN28A at both the mRNA and the protein levels in colon cancer, and revealed that non-coding LIN28A mRNA promotes the metastasis of colon cancer cells by positively regulating the expression of METAP2 as “miRNA sponges.”

## Data Availability Statement

The original contributions presented in the study are included in the article/Supplementary Material, further inquiries can be directed to the corresponding authors.

## Ethics Statement

The studies involving human participants were reviewed and approved by Harbin Medical University Institutional Ethics Committee. The patients/participants provided their written informed consent to participate in this study. The animal study was reviewed and approved by the Institutional Animal Care and Use Committee of Harbin Medical University.

## Author Contributions

XL, TZ, and XJ designed and supervised this study. MW, YF, and TW drafted the manuscript. WY, JL, and YZ illustrated the figures for the manuscript. XZ, CW, YQ, LiZ, and SG performed the experiments. YH, LeZ, and YW helped with the *in vivo* experiments. GW collected tissue samples and the clinical data. RZ analyzed the data. All authors approved the final manuscript.

## Conflict of Interest

The authors declare that the research was conducted in the absence of any commercial or financial relationships that could be construed as a potential conflict of interest.

## Footnotes

1 http://cistrome.org/db/#/

## Supplementary Material

Supplementary Table 1 The primer sequences used in this study.

Supplementary Table 2 Protein expression profile detected by MS.

Supplementary Table 3 The miRNAs targeting both LIN28A and METAP2.

## Abbreviations

METAP2: methionyl aminopeptidase 2
DFO: deferoxamine
HCCs: hepatocellular carcinoma cells
HIF1 α: hypoxia-inducible factor 1 alpha.
