# Supplementary material for: Hypoxia-Induced LIN28A mRNA Promotes the Metastasis of Colon Cancer in a Protein-Coding-Independent Manner
Source: Front Cell Dev Biol. 2021 Feb 16;9:642930. doi: 10.3389/fcell.2021.642930 (PMC7921329; doi:10.3389/fcell.2021.642930)
Supplement: Supplementary Table 1 — The primer sequences used in this study. [file Data_Sheet_1.PDF]

Supplementary Table 1. Primer sequences used in this study

Primer name	Primer sequence (5’ to 3’)
LIN28A-F	TGCGGGCATCTGTAAGTGG
LIN28A-R	GGAACCCTTCCATGTGCAG
METAP2-F	GCTTGGGAGAAAGTAAAT
METAP2-R	GACGCAACAGGATGGTAT
DICER-F	AAAGACTCGGAATATGGTT
DICER-R	AGCCTGGAGAAGGTAAGC
RPL19-F	GAGTATGCTCAGGCTTCAGA
RPL19-R	ATTGGCGATTTCATTGGT
Tubulin-F	ACCTTAACCGCCTTATTAGCCA
Tubulin-R	ACATTCAGGGCTCCATCAAATC
U6-F	CTCGCTTCGGCAGCACATATACT
U6-R	ACGCTTCACGAATTTGCGTGTC
let-7a/e-F	TGAGGTAGTAGGTTG
let-7b-F	TGAGGTAGTAGGTTGTGT
let-7c-F	TGAGGTAGTAGGTTGTAT
let-7d-F	AGAGGTAGTAGGTTGCAT
miR-181-F	AACATTCAACGCTGTCGGTGAGT
Universal-R	GTCGTATCCAGTGCAGGGTCCGAGGT
U6-RT	AAAATATGGAACGCTTCACGAATTTG
let-7a/d/e-RT	GTCGTATCCAGTGCAGGGTCCGAGGTAT<br>TCGCACTGGATACGACAACTAT
let-7b-RT	GTCGTATCCAGTGCAGGGTCCGAGGTAT<br>TCGCACTGGATACGACAACCAC
let-7c-RT	GTCGTATCCAGTGCAGGGTCCGAGGTAT<br>TCGCACTGGATACGACAACCAT
miR-181-RT	GTCGTATCCAGTGCAGGGTCCGAGGTAT<br>TCGCACTGGATACGACACTCAC
LIN28A luciferase -F:	CCGACGCGTTTTTAGGCGGAGCAGCAA
LIN28A luciferase -R:	CCGCTCGAGGCTCCTCTTCCCACAACG
LIN28A chip-1-F	GAGCCACAGCGTGAGAAT
LIN28A chip-1-R	TACTGCCCTGGTCGGAGA
LIN28A chip-2-F	GTGTTTCTGATTGGCCAGC
LIN28A chip-2-R	ACCCCGGAATTTGAGATC
LIN28A chip-3-F	GGGACACTTTAGGATTCAGGG
LIN28A chip-3-R	AGACTCACGGCGGGACAA
negative control	UUCUCCGAACGUGUCACGUTT<br>ACGUGACACGUUCGGAGAATT
microRNA-181a mimic	AACAUUCAACGCUGUCGGUGAGU<br>UCACCGACAGCGUUGAAUGUUUU

microRNA let-7 mimic	UGAGGUAGUAGGUUGUAUAGUU<br>CUAUACAACCUACUACCUCAUU
si-METAP2-homo-1754(#1)	CCCUGUAAGUUUGGAGCAATT<br>UUGCUCCAAACUUACAGGGTT
si-METAP2-homo-4421(#2)	GCACCUAUUCAAAUACCAATT<br>UUGGUAUUUGAAUAGGUGCTT
si-LIN28A-homo-324(#1)	GGAUGUCUUUGUGCACCAGTT<br>CUGGUGCACAAAGACAUCCTT
si-LIN28A-homo-535(#2)	CUGUGGAGGUCUAGAUCAUTT<br>AUGAUCUAGACCUCCACAGTT
pLKO-Dicer #1042(#1)	ACAGACACTTTCCTAAGGAAA<br>TTTCCTTAGGAAAGTGTCTGT
pLKO-Dicer #1904(#2)	ATAGATACTGTGCTAGATTAC<br>GTAATCTAGCACAGTATCTAT
pLKO-Dicer #1996(#3)	ACTCTTTATCTGCCAATTAAC<br>GTTAATTGGCAGATAAAGAGT

Supplemental Table 3. miRNAs targeting LIN28A and METAP2

Target Gene	miRNA	Species (miRNA)
LIN28A	let-7-5p	Homo sapiens
	miR-101-3p	Homo sapiens
	miR-103-3p/107	Homo sapiens
	miR-10-5p	Homo sapiens
	miR-106-5p	Homo sapiens
	miR-122-5p	Homo sapiens
	miR-125-5p	Homo sapiens
	miR-128-3p	Homo sapiens
	miR-129-5p	Homo sapiens
	miR-130-3p	Homo sapiens
	miR-140-3p	Homo sapiens
	miR-142-3p	Homo sapiens
	miR-142-5p	Homo sapiens
	miR-144-3p	Homo sapiens
	miR-145-5p	Homo sapiens
	miR-146-5p	Homo sapiens
	miR-148-3p	Homo sapiens
	miR-150-5p	Homo sapiens
	miR-152-3p	Homo sapiens
	miR-153-3p	Homo sapiens
	miR-15-5p	Homo sapiens
	miR-16-5p	Homo sapiens
	miR-17-5p	Homo sapiens
	miR-181-5p	Homo sapiens
	miR-182-5p	Homo sapiens
	miR-183-5P.1	Homo sapiens
	miR-18-5p	Homo sapiens
	miR-195-5p	Homo sapiens
	miR-196-5p	Homo sapiens
	miR-199-3p	Homo sapiens
	miR-203-3p.1	Homo sapiens
	miR-204-5p	Homo sapiens
	miR-20-5p	Homo sapiens
	miR-211-5p	Homo sapiens
	miR-214-5p	Homo sapiens
	miR-22-3p	Homo sapiens
	miR-23-3p	Homo sapiens
	miR-24-3p	Homo sapiens
	miR-25-3p	Homo sapiens
	miR-29-3p	Homo sapiens

	miR-301-3p	Homo sapiens
	miR-302-3p	Homo sapiens
	miR-30-5p	Homo sapiens
	miR-32-5p	Homo sapiens
	miR-33-5p	Homo sapiens
	miR-338-3p	Homo sapiens
	miR-34-5p	Homo sapiens
	miR-363-3p	Homo sapiens
	miR-367-3p	Homo sapiens
	miR-372-3p	Homo sapiens
	miR-373-3p	Homo sapiens
	miR-383-5p.1	Homo sapiens
	miR-424-5p	Homo sapiens
	miR-425-5p	Homo sapiens
	miR-449-5p	Homo sapiens
	miR-454-3p	Homo sapiens
	miR-489-3p	Homo sapiens
	miR-497-5p	Homo sapiens
	miR-519-3p	Homo sapiens
	miR-520-3p	Homo sapiens
	miR-802	Homo sapiens
	miR-92-3p	Homo sapiens
	miR-93-5p	Homo sapiens
	miR-9-5p	Homo sapiens
	miR-96-5p	Homo sapiens
	miR-98-5p	Homo sapiens
METAP2	let-7-5p	Homo sapiens
	miR-124-3p	Homo sapiens
	miR-125-5p	Homo sapiens
	miR-1271-5p	Homo sapiens
	miR-140-3p	Homo sapiens
	miR-143-3p	Homo sapiens
	miR-144-3p	Homo sapiens
	miR-150-5p	Homo sapiens
	miR-155-5p	Homo sapiens
	miR-181-5p	Homo sapiens
	miR-183-5p	Homo sapiens
	miR-194-5p	Homo sapiens
	miR-199-3p	Homo sapiens
	miR-204-5p	Homo sapiens
	miR-211-5p	Homo sapiens
	miR-216-5p	Homo sapiens
	miR-219-5p	Homo sapiens

miR-26-5p	Homo sapiens
miR-29-3p	Homo sapiens
miR-30-5p	Homo sapiens
miR-506-3p	Homo sapiens
miR-96-5p	Homo sapiens
miR-98-5p	Homo sapiens
