# Supplementary material for: Hypoxia-Induced LIN28A mRNA Promotes the Metastasis of Colon Cancer in a Protein-Coding-Independent Manner
Source: Front Cell Dev Biol. 2021 Feb 16;9:642930. doi: 10.3389/fcell.2021.642930 (PMC7921329; doi:10.3389/fcell.2021.642930)
Supplement: Supplementary Table 2 — Protein expression profile detected by MS. [file Data_Sheet_2.pdf]

Supplemental Table 2. Protein expression profile detected by MS

Protein accession	Protein description	MW [kDa]	calc.pI	Score	Coverage [%]	#Peptides	#PSMs	#Unique peptides	SW1116-Ctrl intensity	SW1116-28AF intensity	SW1116-28AF/SW1116-Ctrl Ratio	SW1116-28AF/SW1116-Ctrl P value
P06732	Creatine kinase M-type	43.074	7.25	9.0695875	3.9370079	2	5	1	186.6	13.4	0.072	
P00326	Alcohol dehydrogenase 1C	39.842	8.29	10.650334	4.5333333	1	1	1	172	28	0.163	
P69905	Hemoglobin subunit alpha	15.248	8.68	8.4096101	19.014085	2	2	2	157.9	42.1	0.267	
P05062	Fructose-bisphosphate aldolase B	39.448	7.87	24.482615	10.164835	3	6	2	151.6	48.4	0.319	
Q8NHP1	Aflatoxin B1 aldehyde reductase member 4	36.941	6.76	9.7172232	5.4380665	2	2	1	137.4	62.6	0.456	
A2RTX5	Probable threonine--tRNA ligase 2, cytoplasmic	92.586	6.05	8.9702401	3.6159601	4	4	2	132.5	67.5	0.509	
Q4KMQ2	Anoctamin-6	106.096	7.77	10.479328	2.967033	3	3	3	132.5	67.5	0.509	
O75385	Serine/threonine-protein kinase ULK1	112.56	8.79	2.3196645	0.6666667	1	1	1	129	71	0.55	
O00264	Membrane-associated progesterone receptor component 1	21.658	4.7	15.519818	14.358974	3	6	3	127.3	72.7	0.571	3.35E-01
O75145	Liprin-alpha-3	133.414	5.68	3.882363	1.2562814	2	3	1	125.5	74.5	0.594	
Q5JPF3	Ankyrin repeat domain-containing protein 36C	199.626	7.83	3.0826197	0.3374578	1	2	1	121.9	78.1	0.641	
Q86W25	NACHT, LRR and PYD domains-containing protein 13	118.807	5.66	2.6088884	0.6711409	1	1	1	121.1	78.9	0.652	
Q9NSI2	Protein FAM207A	25.441	11.08	4.4170281	6.0869565	1	1	1	121.1	78.9	0.652	
P02748	Complement component C9	63.133	5.59	6.1892971	2.1466905	1	1	1	120.8	79.2	0.656	
Q8WV99	AN1-type zinc finger protein 2B	28.004	6.92	4.1466253	5.8365759	2	2	2	120.7	79.3	0.657	
P05204	Non-histone chromosomal protein HMG-17	9.387	9.99	4.8738686	15.555556	1	1	1	118.5	81.5	0.688	
O60613	15 kDa selenoprotein	17.78	5.03	28.005874	17.901235	2	3	2	117.9	82.1	0.696	3.72E-01
O94760	N(G),N(G)-dimethylarginine dimethylaminohydrolase 1	31.102	5.81	22.643175	16.842105	5	11	4	117.8	82.2	0.698	2.22E-01
Q8TE67	Epidermal growth factor receptor kinase substrate 8-like protein 3	66.819	7.99	17.891235	6.9139966	4	4	4	117	83	0.709	
P46976	Glycogenin-1	39.359	5.53	24.786762	12.285714	3	5	3	116.7	83.3	0.714	4.88E-01
O00399	Dynactin subunit 6	20.734	6.32	11.252676	15.789474	3	3	3	115.9	84.1	0.726	
Q5T440	Putative transferase CAF17, mitochondrial	38.131	9.83	8.4051939	5.8988764	2	2	2	115.8	84.2	0.727	
Q9HC07	Transmembrane protein 165	34.883	7.02	15.513872	13.271605	2	3	2	115.7	84.3	0.729	1.88E-01
P09234	U1 small nuclear ribonucleoprotein C	17.381	9.67	39.020833	13.207547	2	9	2	115.5	84.5	0.732	3.47E-01
Q7Z7K6	Centromere protein V	29.927	9.73	9.1595729	6.9090909	2	7	2	114.9	85.1	0.741	4.66E-01
Q15554	Telomeric repeat-binding factor 2	59.557	9.35	5.3381873	1.8450185	1	1	1	114.5	85.5	0.747	
Q9UII2	ATPase inhibitor, mitochondrial	12.241	9.35	37.880268	32.075472	6	21	6	114.5	85.5	0.747	4.87E-02
Q8TBP6	Solute carrier family 25 member 40	38.1	9.35	2.7587026	2.9585799	1	1	1	114	86	0.754	
P09417	Dihydropteridine reductase	25.773	7.37	28.447971	28.688525	5	5	5	113.8	86.2	0.757	
Q58FF6	Putative heat shock protein HSP 90-beta 4	58.228	4.73	57.70856	9.7029703	7	47	1	113.7	86.3	0.759	
P48735	Isocitrate dehydrogenase [NADP], mitochondrial	50.877	8.69	54.232484	18.584071	8	14	7	113.4	86.6	0.764	6.05E-02
P0DI83	Ras-related protein Rab-34, isoform NARR	21.106	12.16	9.4773229	13.636364	2	2	2	113.2	86.8	0.767	
P15104	Glutamine synthetase	42.037	6.89	46.468439	16.89008	7	17	7	113.2	86.8	0.767	7.55E-02
P38919	Eukaryotic initiation factor 4A-III	46.841	6.73	160.74292	29.927007	16	36	13	113.1	86.9	0.768	8.00E-02
P16402	Histone H1.3	22.336	11.02	114.16383	26.696833	9	36	1	112.9	87.1	0.771	
Q9GZZ9	Ubiquitin-like modifier-activating enzyme 5	44.834	4.84	18.822297	7.6732673	3	4	3	112.9	87.1	0.771	4.40E-03
O15111	Inhibitor of nuclear factor kappa-B kinase subunit alpha	84.585	6.73	3.3060973	2.147651	1	1	1	112.7	87.3	0.775	
Q9BT17	Mitochondrial ribosome-associated GTPase 1	37.213	9.47	21.499302	12.275449	4	5	4	112.3	87.7	0.781	
O43761	Synaptogyrin-3	24.539	8.18	3.2860057	6.5502183	1	1	1	112.2	87.8	0.783	
Q03405	Urokinase plasminogen activator surface receptor	36.953	6.65	7.8395315	6.8656716	1	1	1	111.9	88.1	0.787	
Q01995	Transgelin	22.596	8.84	13.768404	5.4726368	1	2	1	111.6	88.4	0.792	
Q9NXG6	Transmembrane prolyl 4-hydroxylase	56.625	6.06	2.7297871	1.3944223	1	1	1	111.5	88.5	0.794	

P35244	Replication protein A 14 kDa subunit	13.56	5.08	18.344926	21.487603	2	5	2	111	89	0.802	
Q07954	Prolow-density lipoprotein receptor-related protein 1	504.276	5.39	75.453243	3.2790493	13	14	13	110.7	89.3	0.807	6.37E-05
Q7Z2E3	Aprataxin	40.714	9.17	3.2391254	2.247191	1	1	1	110.3	89.7	0.813	
A1L390	Pleckstrin homology domain-containing family G member 3	134.329	6.55	14.6724	2.1328958	2	2	2	110.1	89.9	0.817	
P28799	Granulins	63.5	6.83	10.67369	3.5413153	2	2	2	110	90	0.818	8.36E-02
O75935	Dynactin subunit 3	21.106	5.47	9.3054652	18.27957	4	5	4	109.8	90.2	0.821	6.82E-03
Q9NQL2	Ras-related GTP-binding protein D	45.559	4.92	9.2519399	5.75	2	2	2	109.7	90.3	0.823	4.05E-01
P16403	Histone H1.2	21.352	10.93	131.48081	39.43662	12	42	4	109.6	90.4	0.825	4.51E-02
Q15696	ıll nuclear ribonucleoprotein auxiliary factor 35 kDa subunit-related pı	58.009	9.72	2.7972393	1.659751	1	1	1	109.6	90.4	0.825	
Q32P51	Heterogeneous nuclear ribonucleoprotein A1-like 2	34.204	9	260.62402	35	15	119	1	109.6	90.4	0.825	
Q75QN2	Integrator complex subunit 8	113.016	7.05	9.293709	1.4070352	1	1	1	109.6	90.4	0.825	
Q96EK6	Glucosamine 6-phosphate N-acetyltransferase	20.736	7.99	50.949553	25	3	8	3	109.6	90.4	0.825	3.79E-01
O15360	Fanconi anemia group A protein	162.671	6.6	14.413895	1.9931271	2	2	2	109.5	90.5	0.826	
P07305	Histone H1.0	20.85	10.84	27.230101	15.463918	3	5	3	109.4	90.6	0.828	
Q9BZQ8	Protein Niban	103.07	4.78	15.415958	3.6637931	3	3	3	109.4	90.6	0.828	
Q969G5	Protein kinase C delta-binding protein	27.685	6.43	14.923719	12.260536	3	5	3	109.2	90.8	0.832	
P56589	Peroxisomal biogenesis factor 3	42.113	8.15	9.5832024	6.7024129	2	2	2	109.1	90.9	0.833	
P48059	LIM and senescent cell antigen-like-containing domain protein 1	37.226	8.05	12.77585	11.384615	4	4	4	109	91	0.835	
Q587I9	Vesicle transport protein SFT2C	21.776	9.89	6.1237822	6.9767442	1	1	1	109	91	0.835	
Q9Y2Z9	Ubiquinone biosynthesis monooxygenase COQ6, mitochondrial	50.838	7.3	3.6108339	2.1367521	1	1	1	109	91	0.835	
O00443	;phatidylinositol 4-phosphate 3-kinase C2 domain-containing subunit a	190.559	8.02	24.556981	2.2538553	4	5	4	108.9	91.1	0.837	
P02768	Serum albumin	69.321	6.28	12.257288	5.090312	3	3	3	108.9	91.1	0.837	
Q9BVL2	Nucleoporin p58/p45	60.86	9.33	26.363004	6.8447412	3	4	3	108.9	91.1	0.837	1.00E-01
P09104	Gamma-enolase	47.239	5.03	115.33171	27.18894	8	45	5	108.8	91.2	0.838	3.62E-04
P34949	Mannose-6-phosphate isomerase	46.626	5.95	41.974675	13.711584	4	5	4	108.6	91.4	0.842	1.12E-01
P49770	Translation initiation factor eIF-2B subunit beta	38.965	6.16	33.070985	17.094017	5	7	5	108.4	91.6	0.845	8.50E-02
Q5VST9	Obscurin	867.94	5.99	4.2564118	0.125502	1	1	1	108.4	91.6	0.845	
Q96KV7	WD repeat-containing protein 90	187.317	6.99	4.7330631	0.5720824	1	1	1	108.4	91.6	0.845	
O75794	Cell division cycle protein 123 homolog	39.11	4.81	10.689446	10.714286	3	3	3	108.3	91.7	0.847	2.06E-03
P16401	Histone H1.5	22.566	10.92	94.968437	32.743363	10	28	8	108.3	91.7	0.847	1.95E-07
P53597	Succinyl-CoA ligase [ADP/GDP-forming] subunit alpha, mitochondria	36.227	8.79	42.648171	24.277457	8	14	8	108.3	91.7	0.847	3.50E-02
Q07002	Cyclin-dependent kinase 18	54.146	8.66	6.2938996	3.3898305	2	2	1	108.2	91.8	0.848	
Q9H081	Protein MIS12 homolog	24.124	5.69	31.517067	23.902439	4	4	4	108.2	91.8	0.848	
O15533	Tapasin	47.596	7.15	21.646017	5.3571429	2	4	2	108.1	91.9	0.85	3.14E-01
P23743	Diacylglycerol kinase alpha	82.577	6.73	45.651135	11.020408	7	9	7	108.1	91.9	0.85	8.68E-02
Q96G46	tRNA-dihydrouridine(47) synthase [NAD(P)(+)]-like	72.548	8.05	19.673775	6.3076923	4	4	4	108	92	0.852	2.18E-01
P28065	Proteasome subunit beta type-9	23.25	5.03	11.348449	18.721461	4	4	4	107.8	92.2	0.855	8.48E-03
Q08209	Serine/threonine-protein phosphatase 2B catalytic subunit alpha isoforn	58.65	5.86	30.187581	11.708253	6	9	1	107.8	92.2	0.855	
Q16527	Cysteine and glycine-rich protein 2	20.94	8.62	7.9710223	5.1813472	1	1	1	107.8	92.2	0.855	
P10412	Histone H1.4	21.852	11.03	135.81615	38.356164	12	40	4	107.7	92.3	0.857	5.96E-01
Q92520	Protein FAM3C	24.665	8.29	19.038752	9.2511013	2	5	2	107.6	92.4	0.859	
Q96ME7	Zinc finger protein 512	64.641	9.76	6.7411234	2.292769	1	1	1	107.6	92.4	0.859	
Q9BUT9	Protein FAM195A	17.817	9.41	6.3337816	6.875	1	2	1	107.6	92.4	0.859	
P30519	Heme oxygenase 2	36.01	5.41	67.223798	25.949367	6	11	6	107.5	92.5	0.86	8.44E-01
Q8NC56	LEM domain-containing protein 2	56.94	9	25.857316	8.3499006	4	5	4	107.5	92.5	0.86	5.24E-01
Q9H3G5	Probable serine carboxypeptidase CPVL	54.129	5.62	10.430624	4.2016807	2	2	2	107.5	92.5	0.86	
Q9Y3C0	WASH complex subunit CCDC53	21.16	4.46	9.2743413	10.824742	2	2	2	107.5	92.5	0.86	

Q9Y6M7	Sodium bicarbonate cotransporter 3	135.957	6.71	13.543884	2.4711697	2	2	2	107.4	92.6	0.862	6.97E-02
Q13421	Mesothelin	68.942	6.38	13.209781	2.2222222	1	2	1	107.3	92.7	0.864	
Q9GZU8	Protein FAM192A	28.895	5.45	15.912391	11.811024	3	4	3	107.3	92.7	0.864	
P24468	COUP transcription factor 2	45.542	8.28	10.795466	4.3478261	2	4	2	107.2	92.8	0.866	5.28E-02
Q6IQ23	Pleckstrin homology domain-containing family A member 7	127.056	9.35	12.982703	2.4977698	3	3	3	107.2	92.8	0.866	7.15E-02
P19971	Thymidine phosphorylase	49.924	5.53	2.5187007	2.2821577	1	1	1	107.1	92.9	0.867	
Q8NAF0	Zinc finger protein 579	60.471	8.69	2.7452103	1.2455516	1	1	1	107.1	92.9	0.867	
Q9BSF4	Uncharacterized protein C19orf52	29.215	8.09	10.133161	5.3846154	1	2	1	107.1	92.9	0.867	
Q9UKV5	E3 ubiquitin-protein ligase AMFR	72.949	6.39	4.5136347	3.2659409	2	2	2	107.1	92.9	0.867	4.59E-01
P52434	DNA-directed RNA polymerases I, II, and III subunit RPABC3	17.132	4.68	16.424838	18	2	3	2	107	93	0.869	
Q96AE7	Tetratricopeptide repeat protein 17	129.477	6.58	5.7130946	0.9640666	1	1	1	107	93	0.869	
Q9H0W9	Ester hydrolase C11orf54	35.095	6.7	10.833866	6.6666667	1	1	1	107	93	0.869	
P02511	Alpha-crystallin B chain	20.146	7.33	5.6695862	6.2857143	1	1	1	106.9	93.1	0.871	
Q9Y6M1	Insulin-like growth factor 2 mRNA-binding protein 2	66.081	8.46	26.529152	6.5108514	3	5	1	106.9	93.1	0.871	
Q9NPD3	Exosome complex component RRP41	26.366	6.52	16.069233	14.693878	4	6	4	106.8	93.2	0.873	
P05091	Aldehyde dehydrogenase, mitochondrial	56.346	7.05	97.473745	25.531915	11	19	10	106.7	93.3	0.874	1.14E-07
P42330	Aldo-keto reductase family 1 member C3	36.83	7.94	52.056719	24.767802	8	16	4	106.7	93.3	0.874	4.00E-01
P51159	Ras-related protein Rab-27A	24.852	5.22	13.799507	4.9773756	1	2	1	106.7	93.3	0.874	
Q06546	GA-binding protein alpha chain	51.263	4.97	13.178714	5.7268722	2	2	2	106.7	93.3	0.874	
Q13042	Cell division cycle protein 16 homolog	71.609	5.85	35.676056	11.451613	6	6	6	106.7	93.3	0.874	1.84E-01
Q8TCT9	Minor histocompatibility antigen H13	41.462	6.43	20.709913	8.2228117	4	10	4	106.7	93.3	0.874	2.00E-01
Q8TDX7	Serine/threonine-protein kinase Nek7	34.528	8.25	17.658625	8.2781457	3	5	3	106.7	93.3	0.874	3.41E-01
Q9BRP1	Programmed cell death protein 2-like	39.391	4.86	7.405386	6.424581	2	2	2	106.7	93.3	0.874	
Q9UEG4	Zinc finger protein 629	96.559	7.93	5.5196589	1.9562716	2	2	2	106.7	93.3	0.874	
Q9Y6D6	Brefeldin A-inhibited guanine nucleotide-exchange protein 1	208.634	5.85	29.99813	3.0286641	6	7	4	106.7	93.3	0.874	5.75E-01
Q13287	N-myc-interactor	35.035	5.34	18.978574	12.37785	4	4	4	106.6	93.4	0.876	
Q14166	Tubulin--tyrosine ligase-like protein 12	74.356	5.53	32.951829	8.3850932	4	4	4	106.6	93.4	0.876	
P06396	Gelsolin	85.644	6.28	72.558285	15.984655	12	17	12	106.5	93.5	0.878	2.86E-04
Q8WVX9	Fatty acyl-CoA reductase 1	59.319	9.17	30.853638	6.407767	3	5	3	106.5	93.5	0.878	6.22E-03
Q9UI08	Ena/VASP-like protein	44.592	8.84	2.5093393	3.125	1	1	1	106.5	93.5	0.878	
Q6ZRP7	Sulfhydryl oxidase 2	77.48	7.72	33.898996	8.0229226	5	8	5	106.4	93.6	0.88	3.57E-02
Q8WXD5	Gem-associated protein 6	18.812	5.12	10.065066	7.7844311	1	2	1	106.4	93.6	0.88	
Q96HP4	Oxidoreductase NAD-binding domain-containing protein 1	34.832	8.37	7.0170959	3.8461538	1	1	1	106.4	93.6	0.88	
Q9H0H5	Rac GTPase-activating protein 1	70.982	8.88	46.609099	17.879747	11	13	11	106.4	93.6	0.88	8.86E-05
P15289	Arylsulfatase A	53.554	6.07	8.9922522	3.3530572	1	1	1	106.3	93.7	0.881	
P29992	Guanine nucleotide-binding protein subunit alpha-11	42.097	5.69	16.651706	6.1281337	2	5	2	106.3	93.7	0.881	3.89E-01
Q03519	Antigen peptide transporter 2	75.616	8.02	12.826258	4.664723	4	4	3	106.3	93.7	0.881	4.35E-01
Q4J6C6	Prolyl endopeptidase-like	83.873	6.38	61.504178	12.517194	8	12	8	106.3	93.7	0.881	4.62E-01
Q8IV48	3'-5' exoribonuclease 1	40.038	6.7	10.310815	6.3037249	2	2	2	106.3	93.7	0.881	4.73E-01
Q8WVB6	Chromosome transmission fidelity protein 18 homolog	107.317	7.21	20.584785	5.7435897	4	4	3	106.3	93.7	0.881	
Q8WZ82	Ovarian cancer-associated gene 2 protein	24.403	6.89	5.7476058	9.69163	2	2	2	106.3	93.7	0.881	
Q99081	Transcription factor 12	72.92	7.02	2.9125735	1.3196481	1	1	1	106.3	93.7	0.881	
Q9UMR5	Lysosomal thioesterase PPT2	34.203	6.33	6.5989011	6.9536424	2	2	2	106.3	93.7	0.881	
P42696	RNA-binding protein 34	48.535	10.11	42.417515	16.744186	7	8	7	106.2	93.8	0.883	7.80E-02
Q8N9B5	Junction-mediating and -regulatory protein	111.376	6.18	3.1897675	0.8097166	1	1	1	106.2	93.8	0.883	
Q8WWI5	Choline transporter-like protein 1	73.253	8.6	2.4282912	1.826484	1	1	1	106.2	93.8	0.883	
Q9NR50	Translation initiation factor eIF-2B subunit gamma	50.208	6.47	63.69484	17.699115	6	10	6	106.2	93.8	0.883	6.44E-02

Q9NY97	N-acetyllactosaminide beta-1,3-N-acetylglucosaminyltransferase 2	45.993	8.54	3.1402015	3.7783375	1	1	1	106.2	93.8	0.883	
P08572	Collagen alpha-2(IV) chain	167.449	8.66	5.9090369	0.9345794	1	1	1	106.1	93.9	0.885	
P50135	Histamine N-methyltransferase	33.274	5.34	4.3771645	2.739726	1	1	1	106.1	93.9	0.885	
P53675	Clathrin heavy chain 2	186.91	5.85	205.34208	10.487805	17	45	2	106.1	93.9	0.885	
Q86V81	THO complex subunit 4	26.872	11.15	83.160137	40.077821	9	20	9	106.1	93.9	0.885	8.96E-01
Q9H2G2	STE20-like serine/threonine-protein kinase	142.607	5.15	88.246591	14.65587	19	27	17	106.1	93.9	0.885	1.86E-01
Q9P0J0	NADH dehydrogenase [ubiquinone] 1 alpha subcomplex subunit 13	16.688	8.43	43.39903	33.333333	5	8	5	106.1	93.9	0.885	5.90E-02
P39748	Flap endonuclease 1	42.566	8.62	135.54177	36.315789	11	27	11	106	94	0.887	4.02E-01
Q16134	lectron transfer flavoprotein-ubiquinone oxidoreductase, mitochondria	68.452	7.55	24.134912	5.3484603	2	3	2	106	94	0.887	
O43920	NADH dehydrogenase [ubiquinone] iron-sulfur protein 5	12.509	9.14	28.008152	50.943396	5	7	5	105.9	94.1	0.889	7.83E-02
Q12962	Transcription initiation factor TFIID subunit 10	21.698	6.57	21.054972	17.889908	2	2	2	105.9	94.1	0.889	
Q5JTD0	Tight junction-associated protein 1	61.783	5.97	3.5284149	1.2567325	1	1	1	105.9	94.1	0.889	
Q6NVY1	3-hydroxyisobutyryl-CoA hydrolase, mitochondrial	43.454	8.19	5.191789	3.1088083	1	1	1	105.9	94.1	0.889	
Q92597	Protein NDRG1	42.808	5.82	77.150545	14.720812	5	16	4	105.9	94.1	0.889	2.47E-01
O00767	Acyl-CoA desaturase	41.496	9	5.3164127	3.6211699	1	1	1	105.8	94.2	0.89	
P45880	Voltage-dependent anion-selective channel protein 2	31.547	7.56	148.88927	54.421769	13	34	12	105.8	94.2	0.89	4.11E-02
Q5SSJ5	Heterochromatin protein 1-binding protein 3	61.169	9.67	73.896214	18.264014	9	17	9	105.8	94.2	0.89	2.43E-01
Q86Y39	NADH dehydrogenase [ubiquinone] 1 alpha subcomplex subunit 11	14.843	8.72	13.28715	10.638298	1	2	1	105.8	94.2	0.89	
Q96HY7	able 2-oxoglutarate dehydrogenase E1 component DHKTD1, mitochor	103.011	6.93	18.95379	4.0261153	3	3	3	105.8	94.2	0.89	
Q9HCN4	GPN-loop GTPase 1	41.714	4.92	16.514641	6.4171123	2	2	2	105.8	94.2	0.89	5.86E-01
O00566	U3 small nucleolar ribonucleoprotein protein MPP10	78.816	4.86	34.603517	11.160059	6	6	6	105.7	94.3	0.892	2.85E-01
O15460	Prolyl 4-hydroxylase subunit alpha-2	60.864	5.71	24.286206	7.6635514	4	5	4	105.7	94.3	0.892	1.33E-03
P07108	Acyl-CoA-binding protein	10.038	6.57	71.351831	62.068966	5	15	5	105.7	94.3	0.892	1.10E-01
P63165	Small ubiquitin-related modifier 1	11.55	5.52	10.218354	26.732673	4	6	4	105.7	94.3	0.892	2.67E-02
Q8IVH8	Mitogen-activated protein kinase kinase kinase kinase 3	101.251	7.56	4.3186683	1.3422819	1	1	1	105.7	94.3	0.892	
Q8WVQ1	Soluble calcium-activated nucleotidase 1	44.812	6.09	3.1030882	1.7456359	1	1	1	105.7	94.3	0.892	
P07205	Phosphoglycerate kinase 2	44.767	8.54	53.75537	13.908873	7	31	2	105.6	94.4	0.894	
P63279	SUMO-conjugating enzyme UBC9	17.995	8.66	76.746475	34.177215	6	13	6	105.6	94.4	0.894	1.31E-01
P82970	High mobility group nucleosome-binding domain-containing protein 5	31.506	4.55	6.6227346	6.0283688	2	2	2	105.6	94.4	0.894	1.33E-01
P05161	Ubiquitin-like protein ISG15	17.876	7.44	5.7852965	9.6969697	2	2	2	105.5	94.5	0.896	1.41E-02
P15374	Ubiquitin carboxyl-terminal hydrolase isozyme L3	26.166	4.92	69.062115	37.391304	8	11	8	105.5	94.5	0.896	3.25E-01
P55210	Caspase-7	34.255	6.07	2.7913586	4.290429	2	2	2	105.5	94.5	0.896	
P98194	Calcium-transporting ATPase type 2C member 1	100.512	6.74	16.937469	3.5908596	3	3	3	105.5	94.5	0.896	1.20E-01
Q96JN8	Neuralized-like protein 4	166.802	5.86	7.9732583	0.5121639	1	2	1	105.5	94.5	0.896	
O15344	E3 ubiquitin-protein ligase Midline-1	75.203	6.8	24.523313	7.4962519	5	5	5	105.4	94.6	0.898	8.89E-02
P04040	Catalase	59.719	7.39	91.391824	25.237192	13	19	13	105.4	94.6	0.898	1.49E-01
P30837	Aldehyde dehydrogenase X, mitochondrial	57.17	6.8	25.793528	9.2843327	5	7	4	105.4	94.6	0.898	
P45973	Chromobox protein homolog 5	22.211	5.86	87.404946	38.743455	7	18	6	105.4	94.6	0.898	1.49E-01
P48637	Glutathione synthetase	52.352	5.92	73.390472	20.675105	9	14	9	105.4	94.6	0.898	5.01E-03
Q13642	Four and a half LIM domains protein 1	36.239	8.97	43.192663	18.885449	6	7	6	105.4	94.6	0.898	1.67E-03
Q15388	Mitochondrial import receptor subunit TOM20 homolog	16.288	8.6	8.1647582	13.793103	2	2	2	105.4	94.6	0.898	
Q92686	Neurogranin	7.614	7.87	6.3833145	19.230769	1	2	1	105.4	94.6	0.898	
Q96I25	Splicing factor 45	44.934	5.97	38.183814	14.713217	6	8	5	105.4	94.6	0.898	1.81E-02
Q9C0B0	RING finger protein unkempt homolog	88.029	6.86	14.404588	2.4691358	2	2	2	105.4	94.6	0.898	
Q9NXR1	Nuclear distribution protein nudE homolog 1	38.785	5.27	15.656335	9.2485549	2	2	2	105.4	94.6	0.898	
O75367	Core histone macro-H2A.1	39.592	9.79	67.16414	26.344086	11	16	11	105.3	94.7	0.899	4.93E-04
Q5TAQ9	DDB1- and CUL4-associated factor 8	66.811	5.39	5.8648673	1.8425461	1	1	1	105.3	94.7	0.899	

Q9UBQ6	Exostosin-like 2	37.441	8.95	5.1795501	2.1212121	1	2	1	105.3	94.7	0.899	
P07902	Galactose-1-phosphate uridylyltransferase	43.336	6.99	3.1865525	2.1108179	1	1	1	105.2	94.8	0.901	
P10176	Cytochrome c oxidase subunit 8A, mitochondrial	7.574	10.24	7.3019974	13.043478	1	3	1	105.2	94.8	0.901	
P49674	Casein kinase I isoform epsilon	47.285	9.66	5.3338566	3.125	1	1	1	105.2	94.8	0.901	
Q13107	Ubiquitin carboxyl-terminal hydrolase 4	108.496	5.71	7.8388192	1.7653167	2	2	1	105.2	94.8	0.901	
Q16540	39S ribosomal protein L23, mitochondrial	17.77	9.69	4.0491485	5.8823529	1	2	1	105.2	94.8	0.901	
Q96P48	AP with Rho-GAP domain, ANK repeat and PH domain-containing pr	162.089	6.23	39.984547	4.5517241	5	7	5	105.2	94.8	0.901	3.62E-02
Q9H270	Vacuolar protein sorting-associated protein 11 homolog	107.768	7.05	13.905357	4.0382572	4	4	4	105.2	94.8	0.901	1.32E-01
P05413	Fatty acid-binding protein, heart	14.849	6.8	17.491732	33.834586	4	4	4	105.1	94.9	0.903	2.28E-02
P16278	Beta-galactosidase	76.027	6.57	37.360251	6.7946824	4	6	4	105.1	94.9	0.903	5.10E-02
P40938	Replication factor C subunit 3	40.53	8.34	46.88169	17.977528	6	8	6	105.1	94.9	0.903	2.19E-01
P42773	Cyclin-dependent kinase 4 inhibitor C	18.116	6.52	14.387141	16.071429	3	4	3	105.1	94.9	0.903	
P46108	Adapter molecule crk	33.81	5.55	12.06584	9.2105263	3	3	3	105.1	94.9	0.903	
P84157	Matrix-remodeling-associated protein 7	21.452	4.26	3.4522253	5.3921569	1	1	1	105.1	94.9	0.903	
P98155	Very low-density lipoprotein receptor	96.035	4.79	3.3398938	1.1454754	1	1	1	105.1	94.9	0.903	
Q14764	Major vault protein	99.266	5.48	98.155516	18.589026	14	16	14	105.1	94.9	0.903	1.19E-09
Q15042	Rab3 GTPase-activating protein catalytic subunit	110.454	5.55	48.054774	8.4607543	7	8	7	105.1	94.9	0.903	8.70E-02
Q8TCD5	5'(3')-deoxyribonucleotidase, cytosolic type	23.368	6.64	29.101079	29.850746	6	7	6	105.1	94.9	0.903	9.45E-02
Q8TD22	Sideroflexin-5	37.101	9.33	3.7652297	2.6470588	1	1	1	105.1	94.9	0.903	
Q9BPW8	Protein NipSnap homolog 1	33.289	9.31	13.475217	7.3943662	2	4	1	105.1	94.9	0.903	
Q9BUQ8	Probable ATP-dependent RNA helicase DDX23	95.524	9.55	83.98864	13.658537	11	18	11	105.1	94.9	0.903	1.17E-01
Q9H9C1	Spermatogenesis-defective protein 39 homolog	56.97	7.4	8.3418199	3.8539554	2	2	2	105.1	94.9	0.903	
Q9UH65	Switch-associated protein 70	68.954	5.87	92.823461	27.863248	15	22	15	105.1	94.9	0.903	4.37E-02
Q9Y2C4	Nuclease EXOG, mitochondrial	41.059	8.27	15.852553	11.684783	4	4	4	105.1	94.9	0.903	1.90E-04
P13929	Beta-enolase	46.957	7.71	152.65878	29.032258	11	69	7	105	95	0.905	3.31E-04
P53804	E3 ubiquitin-protein ligase TTC3	229.724	7.52	3.5204247	0.5432099	1	1	1	105	95	0.905	
Q02252	Iethylmalonate-semialdehyde dehydrogenase [acylating], mitochondri	57.803	8.5	58.511218	24.485981	10	12	10	105	95	0.905	9.80E-03
Q15651	High mobility group nucleosome-binding domain-containing protein 3	10.66	9.66	3.3977229	9.0909091	1	1	1	105	95	0.905	
Q53FV1	ORM1-like protein 2	17.352	9.64	3.1346591	7.1895425	1	1	1	105	95	0.905	
Q8N335	Glycerol-3-phosphate dehydrogenase 1-like protein	38.394	7.02	9.8041523	5.4131054	2	2	2	105	95	0.905	3.57E-02
Q96L58	Beta-1,3-galactosyltransferase 6	37.114	9.66	9.5118348	6.6869301	2	2	2	105	95	0.905	
Q9BZV1	UBX domain-containing protein 6	49.723	6.89	16.318849	8.1632653	2	2	2	105	95	0.905	
Q9UHL4	Dipeptidyl peptidase 2	54.307	6.32	13.59036	5.6910569	2	3	2	105	95	0.905	6.85E-03
O60493	Sorting nexin-3	18.751	8.66	19.531002	19.135802	3	6	2	104.9	95.1	0.907	
P62424	60S ribosomal protein L7a	29.977	10.61	134.56257	42.481203	14	28	14	104.9	95.1	0.907	1.65E-03
Q15526	Surfeit locus protein 1	33.31	9.6	12.637318	8.6666667	2	2	2	104.9	95.1	0.907	
Q96HH9	GRAM domain-containing protein 3	47.839	7.9	7.7759852	5.787037	1	1	1	104.9	95.1	0.907	
Q9P253	Vacuolar protein sorting-associated protein 18 homolog	110.116	6.07	9.1687063	1.5416238	1	1	1	104.9	95.1	0.907	
Q9Y6N5	Sulfide:quinone oxidoreductase, mitochondrial	49.929	9.11	12.26442	4.6666667	3	3	3	104.9	95.1	0.907	
O00400	Acetyl-coenzyme A transporter 1	60.87	7.33	20.361582	5.8287796	3	4	3	104.8	95.2	0.908	
P17568	NADH dehydrogenase [ubiquinone] 1 beta subcomplex subunit 7	16.391	8.92	27.093933	25.547445	3	6	3	104.8	95.2	0.908	1.72E-02
P40306	Proteasome subunit beta type-10	28.918	7.81	4.8227522	3.6630037	1	1	1	104.8	95.2	0.908	
Q13148	TAR DNA-binding protein 43	44.711	6.19	46.961434	13.768116	6	11	6	104.8	95.2	0.908	7.97E-01
Q96D15	Reticulocalbin-3	37.47	4.89	59.050091	18.597561	6	11	6	104.8	95.2	0.908	9.53E-03
Q9BPX6	Calcium uptake protein 1, mitochondrial	54.316	8.41	29.180354	15.336134	7	7	7	104.8	95.2	0.908	1.77E-01
Q9H7C9	Mth938 domain-containing protein	13.324	8.46	7.473744	15.57377	2	2	2	104.8	95.2	0.908	
Q9Y3A3	MOB-like protein phocein	26.016	5.78	24.51805	20	3	3	3	104.8	95.2	0.908	

O14684	Prostaglandin E synthase	17.091	9.5	6.9872071	11.842105	2	4	2	104.7	95.3	0.91	
P10301	Ras-related protein R-Ras	23.466	6.93	35.937616	12.844037	2	4	1	104.7	95.3	0.91	
P14854	Cytochrome c oxidase subunit 6B1	10.186	7.05	29.657259	52.325581	4	6	4	104.7	95.3	0.91	7.06E-01
P37837	Transaldolase	37.516	6.81	116.19859	38.575668	14	35	14	104.7	95.3	0.91	6.62E-02
Q03154	Aminoacylase-1	45.856	6.18	33.234492	15.441176	5	6	5	104.7	95.3	0.91	5.75E-04
Q08AF3	Schlafen family member 5	100.99	8.22	6.6744843	1.3468013	1	1	1	104.7	95.3	0.91	
Q92522	Histone H1x	22.474	10.76	74.564994	35.211268	6	12	6	104.7	95.3	0.91	1.07E-02
Q99543	DnaJ homolog subfamily C member 2	71.952	8.7	68.208726	20.128824	11	11	11	104.7	95.3	0.91	3.36E-01
Q9BW91	ADP-ribose pyrophosphatase, mitochondrial	39.101	8.22	11.4968	5.7142857	2	2	2	104.7	95.3	0.91	
P08758	Annexin A5	35.914	5.05	145.5719	49.0625	15	49	14	104.6	95.4	0.912	1.92E-01
P29590	Protein PML	97.489	6.21	11.085554	3.6281179	3	3	3	104.6	95.4	0.912	4.75E-02
P48426	Phosphatidylinositol 5-phosphate 4-kinase type-2 alpha	46.195	6.99	5.2531212	4.1871921	2	2	2	104.6	95.4	0.912	
P51153	Ras-related protein Rab-13	22.76	9.19	33.276132	21.182266	4	13	2	104.6	95.4	0.912	
P78347	General transcription factor II-I	112.346	6.39	213.23119	28.356713	25	39	25	104.6	95.4	0.912	9.02E-02
Q13596	Sorting nexin-1	59.033	5.15	70.406714	18.965517	9	13	7	104.6	95.4	0.912	5.65E-01
Q7Z5K2	Wings apart-like protein homolog	132.863	5.44	15.576424	3.8655462	4	4	4	104.6	95.4	0.912	2.72E-01
Q969T9	WW domain-binding protein 2	28.068	5.91	14.298778	12.643678	4	5	4	104.6	95.4	0.912	3.01E-01
Q96BN8	Ubiquitin thioesterase otulin	40.237	5.47	40.786379	17.897727	6	8	6	104.6	95.4	0.912	3.72E-01
Q9UBM7	7-dehydrocholesterol reductase	54.454	8.7	6.6171769	4	2	2	2	104.6	95.4	0.912	1.72E-01
Q9UKB3	DnaJ homolog subfamily C member 12	23.4	5.71	16.42439	12.626263	2	3	2	104.6	95.4	0.912	1.70E-01
Q9Y450	HBS1-like protein	75.426	6.61	64.381028	19.590643	11	11	11	104.6	95.4	0.912	2.03E-01
O00487	26S proteasome non-ATPase regulatory subunit 14	34.555	6.52	57.383669	31.290323	9	11	9	104.5	95.5	0.914	1.53E-01
P37268	Squalene synthase	48.084	6.54	17.191897	10.551559	3	3	3	104.5	95.5	0.914	9.43E-02
Q03169	Tumor necrosis factor alpha-induced protein 2	72.616	6.46	12.829838	4.8929664	2	2	2	104.5	95.5	0.914	
Q16654	ıvate dehydrogenase (acetyl-transferring)] kinase isozyme 4, mitochon	46.44	6.65	3.0054189	2.676399	1	1	1	104.5	95.5	0.914	
Q6UXH1	Cysteine-rich with EGF-like domain protein 2	38.166	4.59	9.6373983	5.9490085	2	2	2	104.5	95.5	0.914	
Q8N568	Serine/threonine-protein kinase DCLK2	83.554	8.34	5.7435228	1.5665796	1	1	1	104.5	95.5	0.914	
Q8N8N7	Prostaglandin reductase 2	38.474	5.41	10.898202	5.6980057	2	2	2	104.5	95.5	0.914	2.23E-01
Q9BRJ2	39S ribosomal protein L45, mitochondrial	35.328	9.03	4.2653602	2.9411765	1	1	1	104.5	95.5	0.914	
Q9BRP4	Proteasomal ATPase-associated factor 1	42.163	6.32	11.286816	7.1428571	3	3	3	104.5	95.5	0.914	
Q9Y2Y9	Krueppel-like factor 13	31.161	9.54	5.7000571	5.9027778	1	1	1	104.5	95.5	0.914	
Q9Y6K5	2'-5'-oligoadenylate synthase 3	121.093	8.4	65.142719	13.063477	12	13	12	104.5	95.5	0.914	8.38E-02
O95749	Geranylgeranyl pyrophosphate synthase	34.849	6.14	27.913308	12.666667	4	6	4	104.4	95.6	0.916	
P15328	Folate receptor alpha	29.799	7.97	136.53419	29.571984	8	53	8	104.4	95.6	0.916	1.64E-03
P19838	Nuclear factor NF-kappa-B p105 subunit	105.29	5.4	12.407762	2.9958678	3	3	2	104.4	95.6	0.916	
P23919	Thymidylate kinase	23.804	8.27	35.96499	32.075472	8	9	8	104.4	95.6	0.916	2.07E-01
P49768	Presenilin-1	52.634	5.31	14.02362	7.7087794	3	3	3	104.4	95.6	0.916	1.16E-01
P51570	Galactokinase	42.246	6.46	40.171573	13.010204	4	8	4	104.4	95.6	0.916	1.29E-02
Q03518	Antigen peptide transporter 1	87.163	8.02	47.962964	10.024752	6	8	6	104.4	95.6	0.916	2.63E-02
Q53HL2	Borealin	31.304	9.86	3.3623102	3.5714286	1	1	1	104.4	95.6	0.916	
Q7Z460	CLIP-associating protein 1	169.346	9.03	122.24815	12.873862	18	22	17	104.4	95.6	0.916	1.80E-01
Q86VI3	Ras GTPase-activating-like protein IQGAP3	184.584	7.65	29.984026	3.4334764	5	7	3	104.4	95.6	0.916	
Q8N5G2	Macoilin	76.13	9.07	4.2227907	1.6566265	1	1	1	104.4	95.6	0.916	
Q92574	Hamartin	129.685	6.47	14.169608	2.5773196	3	3	3	104.4	95.6	0.916	
Q96AG4	Leucine-rich repeat-containing protein 59	34.909	9.57	100.57228	38.110749	12	26	12	104.4	95.6	0.916	4.24E-02
Q9BQ69	O-acetyl-ADP-ribose deacetylase MACROD1	35.483	9.51	14.838341	8.9230769	3	3	3	104.4	95.6	0.916	1.25E-01
Q9GZX9	Twisted gastrulation protein homolog 1	24.999	5.34	4.4016473	4.0358744	1	1	1	104.4	95.6	0.916	

Q9H1K0	Rabenosyn-5	88.815	5.5	2.319302	0.8928571	1	1	1	104.4	95.6	0.916	
Q9Y256	CAAX prenyl protease 2	35.809	7.97	7.3559555	4.2553191	1	1	1	104.4	95.6	0.916	
O00459	Phosphatidylinositol 3-kinase regulatory subunit beta	81.495	6.43	24.526099	7.5549451	4	4	4	104.3	95.7	0.918	
O75828	Carbonyl reductase [NADPH] 3	30.831	6.18	46.734217	23.465704	5	8	2	104.3	95.7	0.918	1.39E-02
P0CG29	Glutathione S-transferase theta-2	27.489	7.02	6.9051796	5.3278689	1	1	1	104.3	95.7	0.918	
P14384	Carboxypeptidase M	50.481	7.36	20.930881	10.383747	4	4	4	104.3	95.7	0.918	
P15954	Cytochrome c oxidase subunit 7C, mitochondrial	7.241	10.27	20.256763	38.095238	3	5	3	104.3	95.7	0.918	5.31E-03
P23443	Ribosomal protein S6 kinase beta-1	59.102	6.65	10.919838	4.952381	2	2	1	104.3	95.7	0.918	
P57678	Gem-associated protein 4	119.96	6.04	25.901642	6.0491493	6	6	6	104.3	95.7	0.918	1.33E-02
Q12893	Transmembrane protein 115	38.172	8.16	12.108897	5.1282051	1	2	1	104.3	95.7	0.918	
Q13185	Chromobox protein homolog 3	20.798	5.33	122.14817	43.169399	7	22	5	104.3	95.7	0.918	1.75E-01
Q5JUR7	Testis-expressed sequence 30 protein	25.568	8.66	16.811962	14.977974	3	3	3	104.3	95.7	0.918	2.54E-03
Q6ZRV2	Protein FAM83H	127.044	6.98	5.5322668	2.2900763	2	2	2	104.3	95.7	0.918	
Q92871	Phosphomannomutase 1	29.728	5.74	11.460536	8.3969466	2	3	1	104.3	95.7	0.918	
Q96SZ5	2-aminoethanethiol dioxygenase	29.732	6.04	10.498274	11.111111	3	3	3	104.3	95.7	0.918	1.10E-01
Q99550	M-phase phosphoprotein 9	132.942	6.24	2.6996219	0.7607777	1	1	1	104.3	95.7	0.918	
Q9H9L4	KAT8 regulatory NSL complex subunit 2	55.007	6.62	6.3165027	2.4390244	1	1	1	104.3	95.7	0.918	
Q9UFF9	CCR4-NOT transcription complex subunit 8	33.519	4.81	17.635145	7.1917808	2	3	1	104.3	95.7	0.918	
Q9UH99	SUN domain-containing protein 2	80.262	6.73	26.664094	6.2761506	3	3	3	104.3	95.7	0.918	3.95E-02
Q9Y5X2	Sorting nexin-8	52.536	7.39	39.99641	11.827957	4	5	4	104.3	95.7	0.918	1.99E-02
Q9Y6B6	GTP-binding protein SAR1b	22.396	6.11	50.440896	28.787879	6	13	3	104.3	95.7	0.918	2.58E-01
O14732	Inositol monophosphatase 2	31.301	6.61	4.427942	3.4722222	1	1	1	104.2	95.8	0.919	
O15382	Branched-chain-amino-acid aminotransferase, mitochondrial	44.259	8.65	41.188245	23.214286	8	8	8	104.2	95.8	0.919	1.82E-02
O75438	NADH dehydrogenase [ubiquinone] 1 beta subcomplex subunit 1	6.957	8.92	7.5573084	18.965517	1	2	1	104.2	95.8	0.919	
O95251	Histone acetyltransferase KAT7	70.598	8.85	7.9447704	4.2553191	2	2	2	104.2	95.8	0.919	1.29E-01
O95834	Echinoderm microtubule-associated protein-like 2	70.634	6.32	58.665134	12.48074	8	10	8	104.2	95.8	0.919	4.58E-02
P07948	Tyrosine-protein kinase Lyn	58.537	7.11	16.648441	8.3984375	5	5	3	104.2	95.8	0.919	
P10253	Lysosomal alpha-glucosidase	105.257	6	28.048498	4.6218487	4	5	4	104.2	95.8	0.919	1.02E-04
Q13123	Protein Red	65.562	6.64	44.116945	11.669659	6	11	6	104.2	95.8	0.919	2.93E-01
Q13228	Selenium-binding protein 1	52.358	6.37	67.786037	23.940678	10	13	10	104.2	95.8	0.919	2.47E-02
Q5M775	Cytospin-B	118.512	6.7	28.25872	6.3670412	5	5	5	104.2	95.8	0.919	4.46E-02
Q6RW13	Type-1 angiotensin II receptor-associated protein	17.408	6.14	8.2672043	13.836478	1	1	1	104.2	95.8	0.919	
Q6UVK1	Chondroitin sulfate proteoglycan 4	250.382	5.47	12.252588	0.9043928	1	1	1	104.2	95.8	0.919	
Q6UW63	KDEL motif-containing protein 1	58.005	7.71	13.280037	7.5697211	4	4	3	104.2	95.8	0.919	
Q8IY67	Ribonucleoprotein PTB-binding 1	63.837	8.48	30.882475	10.231023	5	7	5	104.2	95.8	0.919	2.45E-02
Q8TBA6	Golgin subfamily A member 5	82.974	5.83	13.351677	3.6935705	2	2	2	104.2	95.8	0.919	
Q96GS4	Uncharacterized protein C17orf59	37.204	5.76	14.783834	7.2829132	1	1	1	104.2	95.8	0.919	
Q9BXV9	Uncharacterized protein C14orf142	10.852	4.27	17.662002	41	2	2	2	104.2	95.8	0.919	3.90E-03
Q9Y3E2	BolA-like protein 1	14.28	7.96	11.430905	16.058394	2	2	2	104.2	95.8	0.919	
O00418	Eukaryotic elongation factor 2 kinase	82.092	5.33	10.837684	2.0689655	2	3	2	104.1	95.9	0.921	8.67E-03
O14672	Disintegrin and metalloproteinase domain-containing protein 10	84.088	7.77	9.4435999	5.2139037	3	3	3	104.1	95.9	0.921	6.08E-01
P42765	3-ketoacyl-CoA thiolase, mitochondrial	41.898	8.09	179.23845	50.377834	13	28	13	104.1	95.9	0.921	4.55E-02
Q6AI08	HEAT repeat-containing protein 6	128.699	7.03	8.8467951	1.2701101	1	1	1	104.1	95.9	0.921	
Q99933	BAG family molecular chaperone regulator 1	38.755	7.81	60.925781	22.318841	7	8	7	104.1	95.9	0.921	3.14E-01
Q9HAT2	Sialate O-acetylesterase	58.277	7.33	6.4954214	3.6328872	2	3	2	104.1	95.9	0.921	7.72E-03
Q9UGN5	Poly [ADP-ribose] polymerase 2	66.164	8.88	3.0802422	1.3722127	1	1	1	104.1	95.9	0.921	
Q9UL25	Ras-related protein Rab-21	24.332	7.94	46.029509	20.444444	5	9	5	104.1	95.9	0.921	2.10E-01

Q9UNK0	Syntaxin-8	26.89	4.98	21.406119	13.983051	2	2	2	104.1	95.9	0.921	
O43237	Cytoplasmic dynein 1 light intermediate chain 2	54.066	6.38	42.572633	12.398374	4	6	4	104	96	0.923	5.65E-02
O43924	ıl rod rhodopsin-sensitive cGMP 3',5'-cyclic phosphodiesterase subuni	17.409	5.67	3.4277094	8	1	1	1	104	96	0.923	
P11172	Uridine 5'-monophosphate synthase	52.189	7.24	66.191206	21.458333	13	17	13	104	96	0.923	2.77E-01
P49419	Alpha-aminoadipic semialdehyde dehydrogenase	58.45	7.99	235.21818	42.486085	18	37	18	104	96	0.923	1.31E-07
Q03426	Mevalonate kinase	42.424	6.46	9.2110492	4.7979798	2	2	2	104	96	0.923	
Q13315	Serine-protein kinase ATM	350.462	6.81	8.4155841	0.9489529	3	3	3	104	96	0.923	
Q8NAV1	Pre-mRNA-splicing factor 38A	37.453	9.96	15.341847	3.8461538	1	2	1	104	96	0.923	
Q969J3	Loss of heterozygosity 12 chromosomal region 1 protein	22.208	6.35	15.837553	14.795918	3	4	3	104	96	0.923	1.91E-01
Q99622	Protein C10	13.17	5.14	38.661931	47.619048	4	6	4	104	96	0.923	6.40E-02
Q9H3H3	UPF0696 protein C11orf68	27.338	5.45	32.387472	18.7251	4	5	4	104	96	0.923	5.20E-03
Q9NR19	Acetyl-coenzyme A synthetase, cytoplasmic	78.529	6.46	23.348687	6.4194009	5	5	5	104	96	0.923	1.36E-02
Q9UKM7	ndoplasmic reticulum mannosyl-oligosaccharide 1,2-alpha-mannosida	79.53	7.72	7.0693056	1.5736767	1	2	1	104	96	0.923	
O00244	Copper transport protein ATOX1	7.397	7.24	6.4343438	25	2	3	2	103.9	96.1	0.925	3.79E-02
O14681	Etoposide-induced protein 2.4 homolog	38.94	9.72	12.609846	3.2352941	1	2	1	103.9	96.1	0.925	
O75462	Cytokine receptor-like factor 1	46.273	9.11	3.2855864	2.3696682	1	1	1	103.9	96.1	0.925	
P13489	Ribonuclease inhibitor	49.941	4.82	173.02604	49.457701	19	33	19	103.9	96.1	0.925	3.81E-02
P14550	Alcohol dehydrogenase [NADP(+)]	36.55	6.79	87.91749	34.769231	9	21	8	103.9	96.1	0.925	2.44E-06
P28331	NADH-ubiquinone oxidoreductase 75 kDa subunit, mitochondrial	79.417	6.23	177.68006	31.911967	18	29	18	103.9	96.1	0.925	1.81E-03
P28676	Grancalcin	23.994	5.21	2.3607128	3.2258065	1	1	1	103.9	96.1	0.925	
P30154	/threonine-protein phosphatase 2A 65 kDa regulatory subunit A beta is	66.171	4.94	49.404156	14.808652	8	16	6	103.9	96.1	0.925	3.34E-03
P36542	ATP synthase subunit gamma, mitochondrial	32.975	9.22	72.60764	24.832215	8	23	7	103.9	96.1	0.925	6.52E-01
P46063	ATP-dependent DNA helicase Q1	73.41	7.88	78.781524	18.181818	11	17	10	103.9	96.1	0.925	1.17E-01
P49914	5-formyltetrahydrofolate cyclo-ligase	23.241	7.88	17.846086	10.344828	2	4	2	103.9	96.1	0.925	7.76E-02
P84090	Enhancer of rudimentary homolog	12.251	5.92	64.403047	33.653846	3	13	3	103.9	96.1	0.925	2.66E-01
Q12834	Cell division cycle protein 20 homolog	54.689	9.23	9.070168	5.01002	2	2	2	103.9	96.1	0.925	
Q15628	umor necrosis factor receptor type 1-associated DEATH domain prote	34.226	6.27	26.58975	11.538462	3	3	3	103.9	96.1	0.925	8.41E-02
Q8TC12	Retinol dehydrogenase 11	35.363	8.82	19.506442	7.5471698	2	3	2	103.9	96.1	0.925	2.93E-01
Q96DB5	Regulator of microtubule dynamics protein 1	35.785	8.5	30.672343	15.605096	5	7	4	103.9	96.1	0.925	5.37E-02
Q9P2R7	Succinyl-CoA ligase [ADP-forming] subunit beta, mitochondrial	50.285	7.42	46.408288	17.710583	8	13	8	103.9	96.1	0.925	3.45E-02
Q9Y315	Deoxyribose-phosphate aldolase	35.208	8.94	35.269909	15.09434	5	8	5	103.9	96.1	0.925	8.34E-02
O00178	GTP-binding protein 1	72.408	8.34	33.404999	7.7727952	4	6	4	103.8	96.2	0.927	4.00E-01
P51397	Death-associated protein 1	11.158	9.32	11.575237	15.686275	2	7	2	103.8	96.2	0.927	1.38E-03
P51991	Heterogeneous nuclear ribonucleoprotein A3	39.571	9.01	155.0991	36.243386	17	30	14	103.8	96.2	0.927	2.24E-01
P61201	COP9 signalosome complex subunit 2	51.564	5.53	55.216177	17.38149	9	15	8	103.8	96.2	0.927	3.13E-06
Q02241	Kinesin-like protein KIF23	109.99	8.51	129.53093	20.9375	16	22	15	103.8	96.2	0.927	2.68E-04
Q15274	Nicotinate-nucleotide pyrophosphorylase [carboxylating]	30.826	6.21	53.393733	31.649832	7	10	7	103.8	96.2	0.927	1.59E-03
Q15382	GTP-binding protein Rheb	20.485	5.92	32.890954	28.804348	5	6	5	103.8	96.2	0.927	5.11E-01
Q16352	Alpha-internexin	55.357	5.4	34.3516	10.621242	6	10	5	103.8	96.2	0.927	2.12E-02
Q16610	Extracellular matrix protein 1	60.635	6.71	4.6703988	2.4074074	1	1	1	103.8	96.2	0.927	
Q6P2E9	Enhancer of mRNA-decapping protein 4	151.567	5.86	102.74427	12.276945	13	16	13	103.8	96.2	0.927	5.55E-02
Q9H0R4	Haloacid dehalogenase-like hydrolase domain-containing protein 2	28.518	6.24	3.9051796	3.0888031	1	1	1	103.8	96.2	0.927	
Q9NSE4	Isoleucine--tRNA ligase, mitochondrial	113.719	7.2	82.272756	12.944664	12	20	12	103.8	96.2	0.927	1.10E-04
Q9NWZ5	Uridine-cytidine kinase-like 1	61.103	7.36	2.8884015	1.6423358	1	1	1	103.8	96.2	0.927	
O00469	Procollagen-lysine,2-oxoglutarate 5-dioxygenase 2	84.632	6.71	7.8435696	2.578019	2	2	2	103.7	96.3	0.929	1.93E-01
P04406	Glyceraldehyde-3-phosphate dehydrogenase	36.03	8.46	463.76318	64.776119	20	239	20	103.7	96.3	0.929	6.11E-02
P07237	Protein disulfide-isomerase	57.081	4.87	299.07893	50.19685	29	89	29	103.7	96.3	0.929	1.16E-02

P09601	Heme oxygenase 1	32.798	8.25	15.537955	8.3333333	2	3	2	103.7	96.3	0.929	
P30536	Translocator protein	18.816	9.36	3.0361178	4.7337278	1	1	1	103.7	96.3	0.929	
P49327	Fatty acid synthase	273.254	6.44	659.66877	29.788929	65	157	65	103.7	96.3	0.929	4.05E-09
Q5RI15	Cytochrome c oxidase protein 20 homolog	13.283	8.76	27.812226	22.881356	3	4	3	103.7	96.3	0.929	5.36E-02
Q86WA6	Valacyclovir hydrolase	32.522	9.14	6.8330302	5.4982818	2	2	2	103.7	96.3	0.929	
Q8TF40	Folliculin-interacting protein 1	130.472	5.52	5.0098832	0.8576329	1	1	1	103.7	96.3	0.929	
Q92614	Unconventional myosin-XVIIIa	232.971	6.3	51.408648	4.4790652	9	12	9	103.7	96.3	0.929	5.75E-03
Q96LJ7	Dehydrogenase/reductase SDR family member 1	33.887	7.83	11.26686	7.6677316	2	2	2	103.7	96.3	0.929	
Q9NP79	Vacuolar protein sorting-associated protein VTA1 homolog	33.858	6.29	31.275401	18.566775	5	5	5	103.7	96.3	0.929	2.75E-01
Q9UM54	Unconventional myosin-VI	149.596	8.53	22.204701	3.5548686	3	3	3	103.7	96.3	0.929	1.40E-02
O75208	Ubiquinone biosynthesis protein COQ9, mitochondrial	35.487	5.94	17.842416	9.1194969	3	4	3	103.6	96.4	0.931	
O95571	Persulfide dioxygenase ETHE1, mitochondrial	27.855	6.83	44.60889	24.409449	5	8	5	103.6	96.4	0.931	1.08E-01
P06730	Eukaryotic translation initiation factor 4E	25.082	6.15	19.226583	13.824885	3	5	3	103.6	96.4	0.931	1.40E-01
P07199	Major centromere autoantigen B	65.132	4.55	3.3484313	2.5041736	1	1	1	103.6	96.4	0.931	
P09972	Fructose-bisphosphate aldolase C	39.431	6.87	156.33569	41.483516	13	33	8	103.6	96.4	0.931	2.07E-02
P16083	Ribosyldihydronicotinamide dehydrogenase [quinone]	25.902	6.29	27.901634	16.883117	3	5	3	103.6	96.4	0.931	2.75E-01
P30038	Delta-1-pyrroline-5-carboxylate dehydrogenase, mitochondrial	61.681	8.07	11.607465	3.374778	2	2	2	103.6	96.4	0.931	
P40426	Pre-B-cell leukemia transcription factor 3	47.16	7.06	4.3634118	2.5345622	1	1	1	103.6	96.4	0.931	
P47736	Rap1 GTPase-activating protein 1	73.315	5.82	14.641592	4.0723982	2	3	2	103.6	96.4	0.931	
P63218	Guanine nucleotide-binding protein G(I)/G(S)/G(O) subunit gamma-5	7.314	9.85	3.6987529	13.235294	1	1	1	103.6	96.4	0.931	
P99999	Cytochrome c	11.741	9.57	53.842987	49.52381	5	9	5	103.6	96.4	0.931	2.81E-02
Q03164	Histone-lysine N-methyltransferase 2A	431.497	9.09	3.831208	0.2771479	1	1	1	103.6	96.4	0.931	
Q53QV2	Protein LBH	12.209	4.41	7.7886124	18.095238	1	1	1	103.6	96.4	0.931	
Q6NXR4	TELO2-interacting protein 2	56.879	7.09	22.056384	11.023622	4	4	4	103.6	96.4	0.931	9.92E-05
Q6P2P2	Putative protein arginine N-methyltransferase 9	94.441	5.25	2.3741733	0.8284024	1	1	1	103.6	96.4	0.931	
Q7L273	BTB/POZ domain-containing protein KCTD9	42.54	6.37	26.88673	13.624679	3	3	3	103.6	96.4	0.931	1.33E-02
Q9BV86	N-terminal Xaa-Pro-Lys N-methyltransferase 1	25.371	5.52	64.362838	28.251121	5	10	5	103.6	96.4	0.931	2.11E-02
Q9NNX1	Tuftelin	44.237	6	3.3082998	2.5641026	1	1	1	103.6	96.4	0.931	
Q9NZD8	Maspardin	34.938	6.28	28.488073	13.311688	3	4	3	103.6	96.4	0.931	2.39E-01
O14653	Golgi SNAP receptor complex member 2	24.76	8.06	6.4851336	8.9622642	2	2	2	103.5	96.5	0.932	
O95219	Sorting nexin-4	51.876	5.99	4.3385659	2	1	1	1	103.5	96.5	0.932	
P14406	Cytochrome c oxidase subunit 7A2, mitochondrial	9.39	9.76	15.288745	27.710843	2	5	2	103.5	96.5	0.932	1.11E-01
P30405	Peptidyl-prolyl cis-trans isomerase F, mitochondrial	22.026	9.38	27.012214	20.289855	6	12	4	103.5	96.5	0.932	1.59E-01
P38117	Electron transfer flavoprotein subunit beta	27.826	8.1	111.09089	56.862745	14	28	14	103.5	96.5	0.932	2.55E-03
P48067	Sodium- and chloride-dependent glycine transporter 1	78.209	7.93	10.948405	4.5325779	2	2	2	103.5	96.5	0.932	
Q06124	Tyrosine-protein phosphatase non-receptor type 11	68.393	7.3	56.997688	23.115578	14	15	14	103.5	96.5	0.932	2.35E-02
Q12907	Vesicular integral-membrane protein VIP36	40.203	6.95	31.534824	21.067416	7	9	7	103.5	96.5	0.932	5.60E-01
Q14432	cGMP-inhibited 3',5'-cyclic phosphodiesterase A	124.901	6	30.024342	6.1349693	7	7	7	103.5	96.5	0.932	1.17E-01
Q15637	Splicing factor 1	68.286	8.98	41.889532	15.492958	7	12	7	103.5	96.5	0.932	5.34E-01
Q16795	H dehydrogenase [ubiquinone] 1 alpha subcomplex subunit 9, mitocho	42.483	9.8	51.548806	22.811671	7	10	7	103.5	96.5	0.932	5.30E-01
Q6MZW2	Follistatin-related protein 4	93.037	6.32	14.776157	5.2256532	2	2	2	103.5	96.5	0.932	
Q86YV9	Hermansky-Pudlak syndrome 6 protein	82.923	6.28	13.138485	2.9677419	2	2	2	103.5	96.5	0.932	7.23E-02
Q92570	Nuclear receptor subfamily 4 group A member 3	68.186	7.81	7.4304108	3.3546326	2	2	2	103.5	96.5	0.932	
Q9BV20	Methylthioribose-1-phosphate isomerase	39.125	6.3	37.514615	20.325203	6	7	6	103.5	96.5	0.932	3.63E-03
Q9NV35	Probable 8-oxo-dGTP diphosphatase NUDT15	18.597	6.14	7.8054857	6.7073171	1	2	1	103.5	96.5	0.932	
Q9P032	ADH dehydrogenase [ubiquinone] 1 alpha subcomplex assembly factor	20.254	8.82	14.255619	21.142857	4	4	4	103.5	96.5	0.932	
Q9Y333	U6 snRNA-associated Sm-like protein LSm2	10.828	6.52	20.896868	38.947368	3	5	3	103.5	96.5	0.932	

O15119	T-box transcription factor TBX3	79.339	8.16	2.9519468	1.076716	1	1	1	103.4	96.6	0.934	
O43674	H dehydrogenase [ubiquinone] 1 beta subcomplex subunit 5, mitochon	21.737	9.63	7.9912528	10.05291	2	2	2	103.4	96.6	0.934	6.76E-01
O94855	Protein transport protein Sec24D	112.936	7.25	58.772527	11.143411	9	11	8	103.4	96.6	0.934	2.99E-01
P13928	Annexin A8	36.858	5.78	105.76494	32.110092	10	22	1	103.4	96.6	0.934	
P17252	Protein kinase C alpha type	76.7	7.05	18.856454	4.0178571	2	3	2	103.4	96.6	0.934	
P42858	Huntingtin	347.383	6.2	5.5121549	0.4137492	1	1	1	103.4	96.6	0.934	
P60002	Transcription elongation factor 1 homolog	9.456	8	30.881382	21.686747	1	3	1	103.4	96.6	0.934	
Q07817	Bcl-2-like protein 1	26.033	4.93	7.5257834	4.2918455	1	2	1	103.4	96.6	0.934	
Q13685	Angio-associated migratory cell protein	46.721	4.42	11.013421	5.0691244	2	3	2	103.4	96.6	0.934	
Q14257	Reticulocalbin-2	36.854	4.4	29.945566	12.933754	5	7	5	103.4	96.6	0.934	3.35E-01
Q8N4Q0	Zinc-binding alcohol dehydrogenase domain-containing protein 2	40.115	8.18	9.0941924	6.3660477	2	3	2	103.4	96.6	0.934	
Q8N8A2	rine/threonine-protein phosphatase 6 regulatory ankyrin repeat subunit	107.535	6.3	6.07053	1.3091641	1	1	1	103.4	96.6	0.934	
Q96BJ3	Axin interactor, dorsalization-associated protein	35.001	6.55	3.6495581	4.248366	1	1	1	103.4	96.6	0.934	
Q96I24	Far upstream element-binding protein 3	61.602	8.38	125.14266	38.636364	17	26	15	103.4	96.6	0.934	6.01E-01
Q96IZ7	Serine/Arginine-related protein 53	38.654	11.08	9.0760016	8.0838323	3	3	2	103.4	96.6	0.934	
Q9HCY8	Protein S100-A14	11.655	5.24	5.6972363	10.576923	1	1	1	103.4	96.6	0.934	
Q9P2X0	Dolichol-phosphate mannosyltransferase subunit 3	10.087	5.94	4.6008457	10.869565	1	1	1	103.4	96.6	0.934	
O00754	Lysosomal alpha-mannosidase	113.672	7.28	30.761405	6.5281899	6	7	6	103.3	96.7	0.936	8.30E-02
O75832	26S proteasome non-ATPase regulatory subunit 10	24.412	6.1	44.615907	23.00885	5	10	5	103.3	96.7	0.936	4.85E-01
O95479	GDH/6PGL endoplasmic bifunctional protein	88.836	7.3	4.5341712	1.2642225	1	1	1	103.3	96.7	0.936	
P04179	Superoxide dismutase [Mn], mitochondrial	24.707	8.25	29.602168	17.117117	3	5	3	103.3	96.7	0.936	5.33E-02
P04792	Heat shock protein beta-1	22.768	6.4	115.16796	40.487805	8	29	8	103.3	96.7	0.936	2.10E-02
P10620	Microsomal glutathione S-transferase 1	17.587	9.39	35.731167	9.0322581	2	6	2	103.3	96.7	0.936	1.12E-01
P16455	Methylated-DNA--protein-cysteine methyltransferase	21.632	8.1	9.2398361	10.628019	2	2	2	103.3	96.7	0.936	1.18E-04
P29373	Cellular retinoic acid-binding protein 2	15.683	5.4	35.763365	24.637681	3	9	3	103.3	96.7	0.936	1.13E-02
P45984	Mitogen-activated protein kinase 9	48.108	5.63	4.4444219	1.8867925	1	1	1	103.3	96.7	0.936	
P48506	Glutamate--cysteine ligase catalytic subunit	72.719	6.09	59.83405	16.012559	7	8	7	103.3	96.7	0.936	1.87E-02
P49441	Inositol polyphosphate 1-phosphatase	43.97	5.26	20.798226	6.5162907	2	3	2	103.3	96.7	0.936	4.15E-01
Q04446	1,4-alpha-glucan-branching enzyme	80.423	6.32	42.445532	12.108262	7	9	7	103.3	96.7	0.936	6.95E-03
Q16698	2,4-dienoyl-CoA reductase, mitochondrial	36.045	9.28	27.858768	17.313433	6	8	6	103.3	96.7	0.936	3.32E-01
Q5T280	Putative methyltransferase C9orf114	41.982	7.43	27.443999	12.5	5	8	5	103.3	96.7	0.936	1.61E-02
Q8IX01	SURP and G-patch domain-containing protein 2	120.132	7.28	18.324057	2.4953789	3	4	3	103.3	96.7	0.936	9.90E-03
Q8N128	Protein FAM177A1	23.742	4.45	14.505011	12.206573	1	1	1	103.3	96.7	0.936	
Q96BW5	Phosphotriesterase-related protein	38.993	6.52	22.154305	11.174785	4	4	4	103.3	96.7	0.936	
Q96BY6	Dedicator of cytokinesis protein 10	249.373	7.14	9.1646229	0.8234218	2	2	2	103.3	96.7	0.936	
Q96IU4	Alpha/beta hydrolase domain-containing protein 14B	22.332	6.4	36.870663	22.380952	4	8	4	103.3	96.7	0.936	1.55E-02
Q9BXS5	AP-1 complex subunit mu-1	48.556	7.3	46.626077	21.040189	9	11	9	103.3	96.7	0.936	3.53E-01
Q9H1P3	Oxysterol-binding protein-related protein 2	55.166	6.35	3.1707606	2.0833333	1	1	1	103.3	96.7	0.936	
Q9H307	Pinin	81.565	7.14	72.654533	14.923291	10	13	10	103.3	96.7	0.936	5.65E-02
Q9HD15	Steroid receptor RNA activator 1	25.657	7.03	34.057846	31.779661	6	7	6	103.3	96.7	0.936	1.16E-01
O60762	Dolichol-phosphate mannosyltransferase subunit 1	29.616	9.57	18.014573	15	4	6	4	103.2	96.8	0.938	1.07E-02
O75608	Acyl-protein thioesterase 1	24.653	6.77	26.89791	13.478261	3	5	3	103.2	96.8	0.938	8.66E-02
P05362	Intercellular adhesion molecule 1	57.789	7.99	14.178401	6.3909774	2	2	2	103.2	96.8	0.938	
P07203	Glutathione peroxidase 1	22.075	6.55	29.714452	23.152709	4	5	4	103.2	96.8	0.938	2.68E-02
P08648	Integrin alpha-5	114.465	5.77	10.252764	3.1458532	3	3	3	103.2	96.8	0.938	
P11166	Solute carrier family 2, facilitated glucose transporter member 1	54.049	8.72	29.013182	6.504065	4	6	4	103.2	96.8	0.938	2.10E-01
P35914	Hydroxymethylglutaryl-CoA lyase, mitochondrial	34.338	8.54	34.658029	13.538462	4	7	4	103.2	96.8	0.938	5.15E-03

P61421	V-type proton ATPase subunit d 1	40.303	5	45.365283	26.495726	8	11	8	103.2	96.8	0.938	1.16E-02
Q14691	DNA replication complex GINS protein PSF1	22.974	7.39	15.65404	17.857143	3	3	2	103.2	96.8	0.938	
Q16630	Cleavage and polyadenylation specificity factor subunit 6	59.173	7.15	107.73039	17.785844	10	21	10	103.2	96.8	0.938	6.39E-01
Q5ST30	Valine--tRNA ligase, mitochondrial	118.415	7.02	6.1403214	1.3170273	1	1	1	103.2	96.8	0.938	
Q8NHM5	Lysine-specific demethylase 2B	152.517	8.56	5.8934691	1.497006	1	1	1	103.2	96.8	0.938	
Q9BV73	Centrosome-associated protein CEP250	280.967	5.02	7.5589682	0.9418509	2	2	2	103.2	96.8	0.938	
Q9BXS6	Nucleolar and spindle-associated protein 1	49.422	9.91	40.868257	15.646259	7	11	7	103.2	96.8	0.938	7.16E-01
Q9HC21	Mitochondrial thiamine pyrophosphate carrier	35.488	9.55	5.7454519	3.125	1	1	1	103.2	96.8	0.938	
Q9NP97	Dynein light chain roadblock-type 1	10.915	7.25	25.127197	29.166667	2	3	2	103.2	96.8	0.938	2.39E-01
Q9NUJ3	T-complex protein 11-like protein 1	56.999	5.59	30.714348	5.8939096	3	6	3	103.2	96.8	0.938	5.13E-02
Q9UJ83	2-hydroxyacyl-CoA lyase 1	63.687	7.36	41.894572	12.629758	6	8	6	103.2	96.8	0.938	8.00E-02
Q9Y277	Voltage-dependent anion-selective channel protein 3	30.639	8.66	56.78404	23.321555	7	15	5	103.2	96.8	0.938	4.89E-01
O95139	NADH dehydrogenase [ubiquinone] 1 beta subcomplex subunit 6	15.479	9.63	33.112055	19.53125	2	6	2	103.1	96.9	0.94	1.79E-01
P07858	Cathepsin B	37.797	6.3	44.770929	19.469027	5	8	5	103.1	96.9	0.94	8.39E-03
P09382	Galectin-1	14.706	5.5	111.88297	58.518519	8	44	8	103.1	96.9	0.94	4.05E-02
P22033	Methylmalonyl-CoA mutase, mitochondrial	83.082	6.93	63.98311	10.133333	7	11	7	103.1	96.9	0.94	7.84E-02
P29597	Non-receptor tyrosine-protein kinase TYK2	133.565	7.15	7.6593576	1.26369	1	1	1	103.1	96.9	0.94	
P51553	Isocitrate dehydrogenase [NAD] subunit gamma, mitochondrial	42.767	8.5	20.943528	13.994911	5	5	5	103.1	96.9	0.94	
P52655	Transcription initiation factor IIA subunit 1	41.488	4.55	18.905698	9.0425532	3	3	3	103.1	96.9	0.94	6.17E-02
Q15011	-responsive endoplasmic reticulum-resident ubiquitin-like domain me	43.693	5.25	4.4897232	4.0920716	1	1	1	103.1	96.9	0.94	
Q8IU81	Interferon regulatory factor 2-binding protein 1	61.649	8.18	17.20094	6.5068493	3	3	3	103.1	96.9	0.94	4.64E-02
Q8IVM0	Coiled-coil domain-containing protein 50	35.8	6.65	10.541967	4.9019608	1	1	1	103.1	96.9	0.94	
Q8IWJ2	GRIP and coiled-coil domain-containing protein 2	195.789	5.14	130.79005	13.004751	20	23	18	103.1	96.9	0.94	5.19E-01
Q8NBT0	POC1 centriolar protein homolog A	44.98	7.59	2.7759852	1.965602	1	1	1	103.1	96.9	0.94	
Q9BR39	Junctophilin-2	74.176	8.72	2.5187007	1.4367816	1	1	1	103.1	96.9	0.94	
Q9BTU6	Phosphatidylinositol 4-kinase type 2-alpha	53.989	8.29	10.247578	6.263048	3	3	3	103.1	96.9	0.94	
Q9H008	Phospholysine phosphohistidine inorganic pyrophosphate phosphatase	29.147	6.15	16.967277	11.481481	2	2	2	103.1	96.9	0.94	
Q9NQS7	Inner centromere protein	105.365	9.44	16.332784	4.0305011	4	5	4	103.1	96.9	0.94	3.52E-01
Q9UER7	Death domain-associated protein 6	81.323	4.87	9.0105164	3.5135135	2	2	2	103.1	96.9	0.94	
Q9UPQ0	LIM and calponin homology domains-containing protein 1	121.792	6.47	71.721626	11.357341	11	18	11	103.1	96.9	0.94	3.84E-02
O14561	Acyl carrier protein, mitochondrial	17.406	4.93	36.66237	15.384615	3	6	3	103	97	0.942	7.00E-01
O15525	Transcription factor MafG	17.839	10.04	7.0596068	12.962963	3	3	3	103	97	0.942	6.70E-01
O60831	PRA1 family protein 2	19.246	9.19	29.744442	16.853933	3	6	3	103	97	0.942	9.38E-03
O95298	NADH dehydrogenase [ubiquinone] 1 subunit C2	14.178	8.98	13.451265	22.689076	3	6	3	103	97	0.942	1.53E-02
P09497	Clathrin light chain B	25.175	4.64	32.325291	21.39738	5	10	5	103	97	0.942	1.86E-02
P09923	Intestinal-type alkaline phosphatase	56.776	5.86	95.45142	26.136364	11	23	8	103	97	0.942	6.25E-01
P24752	Acetyl-CoA acetyltransferase, mitochondrial	45.171	8.85	139.59556	40.046838	16	36	16	103	97	0.942	6.99E-03
P31327	Carbamoyl-phosphate synthase [ammonia], mitochondrial	164.835	6.74	821.71942	47.866667	67	219	63	103	97	0.942	9.73E-13
P47712	Cytosolic phospholipase A2	85.184	5.38	68.75065	13.751669	10	15	10	103	97	0.942	1.04E-01
Q02818	Nucleobindin-1	53.846	5.25	179.53677	39.913232	16	33	16	103	97	0.942	7.34E-01
Q14914	Prostaglandin reductase 1	35.847	8.29	89.267236	24.924012	9	22	9	103	97	0.942	2.91E-04
Q3KQV9	UDP-N-acetylhexosamine pyrophosphorylase-like protein 1	56.994	6.32	47.565527	15.976331	7	8	6	103	97	0.942	4.15E-02
Q63HN8	E3 ubiquitin-protein ligase RNF213	591.03	6.48	35.431707	1.6324179	8	9	7	103	97	0.942	3.85E-03
Q71SY5	Mediator of RNA polymerase II transcription subunit 25	78.121	8.34	3.1655157	1.0709505	1	1	1	103	97	0.942	
Q8WTV0	Scavenger receptor class B member 1	60.838	8.24	18.771145	5.7971014	3	4	3	103	97	0.942	1.94E-02
Q8WUH6	Transmembrane protein 263	11.741	9.32	13.452149	31.896552	3	3	3	103	97	0.942	
Q92989	Polyribonucleotide 5'-hydroxyl-kinase Clp1	47.615	6.62	10.295629	7.0588235	2	3	2	103	97	0.942	4.22E-02

Q96SU4	Oxysterol-binding protein-related protein 9	83.132	6.18	14.887975	3.8043478	3	3	3	103	97	0.942	9.52E-02
Q9H553	Alpha-1,3/1,6-mannosyltransferase ALG2	47.061	7.05	6.9303319	2.8846154	1	1	1	103	97	0.942	
Q9NX14	H dehydrogenase [ubiquinone] 1 beta subcomplex subunit 11, mitocho	17.306	5.22	10.325067	7.1895425	1	2	1	103	97	0.942	
Q9NX62	Inositol monophosphatase 3	38.657	6.86	28.672498	8.0779944	4	8	4	103	97	0.942	2.76E-01
Q9UQ90	Paraplegin	88.179	8.69	16.072597	4.5283019	3	3	3	103	97	0.942	9.74E-02
O14974	Protein phosphatase 1 regulatory subunit 12A	115.211	5.4	136.99019	16.796117	15	24	15	102.9	97.1	0.944	5.38E-02
P00403	Cytochrome c oxidase subunit 2	25.548	4.82	15.380849	16.299559	4	7	4	102.9	97.1	0.944	1.82E-02
P06132	Uroporphyrinogen decarboxylase	40.761	6.14	24.446061	10.354223	3	4	3	102.9	97.1	0.944	4.65E-03
P09960	Leukotriene A-4 hydrolase	69.241	6.18	164.427	34.042553	18	35	18	102.9	97.1	0.944	2.17E-01
P11310	Medium-chain specific acyl-CoA dehydrogenase, mitochondrial	46.559	8.37	71.903091	22.565321	8	15	8	102.9	97.1	0.944	2.19E-01
P18031	Tyrosine-protein phosphatase non-receptor type 1	49.935	6.27	97.410896	34.712644	13	23	13	102.9	97.1	0.944	3.83E-02
P20645	Cation-dependent mannose-6-phosphate receptor	30.973	5.83	19.757641	10.830325	4	5	4	102.9	97.1	0.944	1.17E-01
P30040	Endoplasmic reticulum resident protein 29	28.975	7.31	98.093437	39.846743	10	22	10	102.9	97.1	0.944	6.05E-01
P32929	Cystathionine gamma-lyase	44.479	6.7	80.323321	25.185185	8	15	8	102.9	97.1	0.944	2.85E-02
P35222	Catenin beta-1	85.442	5.86	22.5929	4.0973111	3	5	2	102.9	97.1	0.944	2.56E-02
P36639	7,8-dihydro-8-oxoguanine triphosphatase	22.505	5.27	26.243514	17.258883	3	4	2	102.9	97.1	0.944	
P53602	Diphosphomevalonate decarboxylase	43.377	7.23	31.288269	10.5	4	6	4	102.9	97.1	0.944	7.23E-02
P56945	Breast cancer anti-estrogen resistance protein 1	93.314	5.67	8.1363537	2.0689655	2	2	2	102.9	97.1	0.944	
P58107	Epiplakin	555.279	5.6	396.81427	30.923379	50	74	46	102.9	97.1	0.944	5.66E-09
Q56VL3	OCIA domain-containing protein 2	16.943	9.03	23.505386	18.831169	3	5	3	102.9	97.1	0.944	8.24E-02
Q58WW2	DDB1- and CUL4-associated factor 6	96.232	5.27	4.3532043	1.744186	1	1	1	102.9	97.1	0.944	
Q5T6V5	UPF0553 protein C9orf64	39.004	5.88	37.426922	22.28739	7	8	7	102.9	97.1	0.944	3.45E-02
Q7Z5G4	Golgin subfamily A member 7	15.814	7.05	7.7884235	16.788321	2	2	2	102.9	97.1	0.944	
Q8IWC1	MAP7 domain-containing protein 3	98.368	9.32	15.248836	4.109589	4	4	4	102.9	97.1	0.944	
Q8N163	Cell cycle and apoptosis regulator protein 2	102.838	5.22	108.86133	26.218852	18	24	18	102.9	97.1	0.944	2.66E-01
Q8WVY7	Ubiquitin-like domain-containing CTD phosphatase 1	36.781	6.46	22.691017	16.352201	4	5	4	102.9	97.1	0.944	3.20E-02
Q96A65	Exocyst complex component 4	110.429	6.49	35.526602	7.1868583	6	6	6	102.9	97.1	0.944	1.24E-02
Q96GC5	39S ribosomal protein L48, mitochondrial	23.92	8.98	28.502585	16.981132	3	4	3	102.9	97.1	0.944	5.94E-04
Q99816	Tumor susceptibility gene 101 protein	43.916	6.46	28.907816	7.6923077	3	5	3	102.9	97.1	0.944	
Q9H0W8	Protein SMG9	57.614	7.01	23.136368	9.2307692	3	3	3	102.9	97.1	0.944	3.17E-01
Q9H5N1	Rab GTPase-binding effector protein 2	63.504	4.78	27.149876	10.193322	6	10	5	102.9	97.1	0.944	
Q9NQ55	Suppressor of SWI4 1 homolog	53.161	10.13	19.057926	8.0338266	4	4	4	102.9	97.1	0.944	3.49E-01
Q9NXC5	WD repeat-containing protein mio	98.521	6.73	20.845943	4.6857143	3	3	3	102.9	97.1	0.944	3.37E-01
Q9UDW1	Cytochrome b-c1 complex subunit 9	7.304	9.47	11.460798	26.984127	1	1	1	102.9	97.1	0.944	
Q9Y305	Acyl-coenzyme A thioesterase 9, mitochondrial	49.87	8.6	80.503786	22.551253	9	15	9	102.9	97.1	0.944	1.89E-01
Q9Y512	Sorting and assembly machinery component 50 homolog	51.943	6.9	58.554517	17.697228	8	11	8	102.9	97.1	0.944	3.63E-01
O15304	Apoptosis regulatory protein Siva	18.682	7.61	16.281569	17.714286	2	2	2	102.8	97.2	0.946	3.29E-01
O95295	SNARE-associated protein Snapin	14.865	9.31	8.0615136	7.3529412	1	2	1	102.8	97.2	0.946	
P06703	Protein S100-A6	10.173	5.48	14.713913	34.444444	4	12	4	102.8	97.2	0.946	4.41E-04
P13804	Electron transfer flavoprotein subunit alpha, mitochondrial	35.058	8.38	151.28503	40.24024	10	33	10	102.8	97.2	0.946	1.30E-02
P14324	Farnesyl pyrophosphate synthase	48.245	6.15	65.820454	16.706444	6	14	6	102.8	97.2	0.946	6.53E-02
P28340	DNA polymerase delta catalytic subunit	123.553	7.03	65.513277	11.29178	11	13	11	102.8	97.2	0.946	5.09E-05
P30533	Alpha-2-macroglobulin receptor-associated protein	41.441	8.78	54.901163	30.532213	11	14	11	102.8	97.2	0.946	5.10E-04
P31150	Rab GDP dissociation inhibitor alpha	50.55	5.14	129.44361	39.821029	13	24	9	102.8	97.2	0.946	9.17E-01
P31749	RAC-alpha serine/threonine-protein kinase	55.651	6.07	20.644957	10.416667	4	5	1	102.8	97.2	0.946	
P31751	RAC-beta serine/threonine-protein kinase	55.733	6.37	16.10368	8.9397089	4	5	1	102.8	97.2	0.946	
P56385	ATP synthase subunit e, mitochondrial	7.928	9.35	26.780381	31.884058	3	6	3	102.8	97.2	0.946	2.82E-01

P68431	Histone H3.1	15.394	11.12	34.833953	31.617647	7	18	2	102.8	97.2	0.946	6.57E-02
Q13085	Acetyl-CoA carboxylase 1	265.385	6.37	145.19318	12.745098	25	30	25	102.8	97.2	0.946	5.19E-03
Q13952	Nuclear transcription factor Y subunit gamma	50.271	6.1	10.708234	4.5851528	2	2	2	102.8	97.2	0.946	1.58E-01
Q5RKV6	Exosome complex component MTR3	28.218	6.28	38.768052	21.323529	5	9	5	102.8	97.2	0.946	5.52E-01
Q6DD87	Zinc finger protein 787	40.518	7.84	30.372698	17.754569	4	4	4	102.8	97.2	0.946	1.09E-01
Q6UWP7	Lysocardiolipin acyltransferase 1	48.888	8.62	6.3427881	4.3478261	2	2	2	102.8	97.2	0.946	
Q6UXN9	WD repeat-containing protein 82	35.056	7.69	6.9246512	6.0702875	2	2	2	102.8	97.2	0.946	
Q8N142	Adenylosuccinate synthetase isozyme 1	50.177	8.59	43.135755	10.065646	4	6	3	102.8	97.2	0.946	9.38E-02
Q9BQ61	Uncharacterized protein C19orf43	18.408	9.44	16.466783	13.068182	2	4	2	102.8	97.2	0.946	
Q9BRX8	Redox-regulatory protein FAM213A	25.747	8.84	15.0592	8.7336245	2	3	2	102.8	97.2	0.946	
Q9C0E2	Exportin-4	130.056	5.05	22.277644	4.1702867	5	8	5	102.8	97.2	0.946	1.14E-02
Q9NWU2	Glucose-induced degradation protein 8 homolog	26.732	4.97	30.067578	18.859649	3	4	3	102.8	97.2	0.946	
Q9UGM6	Tryptophan--tRNA ligase, mitochondrial	40.121	9.28	19.674077	11.666667	4	4	4	102.8	97.2	0.946	
Q9Y3Y2	Chromatin target of PRMT1 protein	26.38	12.23	38.517934	10.483871	2	5	2	102.8	97.2	0.946	8.55E-01
O43149	Zinc finger ZZ-type and EF-hand domain-containing protein 1	330.864	5.95	21.190216	1.2495778	4	4	3	102.7	97.3	0.947	
O60749	Sorting nexin-2	58.435	5.12	53.597486	16.955684	9	12	6	102.7	97.3	0.947	6.37E-02
P0C0S5	Histone H2A.Z	13.545	10.58	45.536638	31.25	4	19	2	102.7	97.3	0.947	1.06E-01
P0C0S8	Histone H2A type 1	14.083	10.9	48.78312	35.384615	5	20	2	102.7	97.3	0.947	1.73E-01
P15529	Membrane cofactor protein	43.719	6.74	17.36709	8.6734694	4	7	4	102.7	97.3	0.947	1.21E-01
P16066	Atrial natriuretic peptide receptor 1	118.844	6.64	11.169065	2.0735156	2	2	2	102.7	97.3	0.947	6.43E-02
P20339	Ras-related protein Rab-5A	23.644	8.15	39.071708	31.162791	5	8	3	102.7	97.3	0.947	4.90E-02
P46781	40S ribosomal protein S9	22.578	10.65	51.837681	43.814433	10	32	10	102.7	97.3	0.947	4.19E-02
P47914	60S ribosomal protein L29	17.741	11.66	31.166612	18.867925	3	10	3	102.7	97.3	0.947	2.83E-01
P51970	NADH dehydrogenase [ubiquinone] 1 alpha subcomplex subunit 8	20.092	7.65	9.2024934	12.209302	2	2	2	102.7	97.3	0.947	7.15E-02
P53794	Sodium/myo-inositol cotransporter	79.641	7.27	9.1346591	3.0640669	1	1	1	102.7	97.3	0.947	
P54727	UV excision repair protein RAD23 homolog B	43.145	4.84	117.08864	35.94132	13	25	10	102.7	97.3	0.947	1.07E-03
P67936	Tropomyosin alpha-4 chain	28.504	4.69	174.05243	51.209677	23	76	11	102.7	97.3	0.947	8.36E-01
Q04760	Lactoylglutathione lyase	20.764	5.31	34.68936	21.73913	5	12	5	102.7	97.3	0.947	8.14E-03
Q05655	Protein kinase C delta type	77.455	7.75	21.017002	8.8757396	5	5	5	102.7	97.3	0.947	5.08E-01
Q13884	Beta-1-syntrophin	58.025	8.63	14.153742	5.5762082	2	2	2	102.7	97.3	0.947	
Q14254	Flotillin-2	47.035	5.25	103.71026	27.803738	11	17	11	102.7	97.3	0.947	3.96E-01
Q14566	DNA replication licensing factor MCM6	92.831	5.41	188.78362	33.61754	28	50	28	102.7	97.3	0.947	5.85E-01
Q15287	RNA-binding protein with serine-rich domain 1	34.188	11.84	66.846874	22.295082	5	9	5	102.7	97.3	0.947	2.46E-01
Q16774	Guanylate kinase	21.712	6.55	2.9531148	5.0761421	1	1	1	102.7	97.3	0.947	
Q52LJ0	Protein FAM98B	37.167	6.29	41.319975	13.333333	4	6	3	102.7	97.3	0.947	3.67E-02
Q53FT3	Protein Hikeshi	21.614	5.45	4.7228494	4.5685279	1	1	1	102.7	97.3	0.947	
Q5VU43	Myomegalin	264.918	5.44	14.897332	0.8525149	2	3	1	102.7	97.3	0.947	
Q7Z2W9	39S ribosomal protein L21, mitochondrial	22.8	9.89	16.458602	19.02439	3	4	3	102.7	97.3	0.947	2.76E-01
Q8NCG7	Sn1-specific diacylglycerol lipase beta	73.684	6.55	24.073054	8.6309524	4	4	4	102.7	97.3	0.947	1.54E-03
Q96I51	Williams-Beuren syndrome chromosomal region 16 protein	49.965	8.4	10.036996	6.4655172	2	2	2	102.7	97.3	0.947	1.46E-01
Q99720	Sigma non-opioid intracellular receptor 1	25.112	5.96	14.401403	5.8295964	1	2	1	102.7	97.3	0.947	
Q9Y2T3	Guanine deaminase	50.971	5.68	94.219933	24.229075	10	23	10	102.7	97.3	0.947	1.02E-03
O14777	Kinetochore protein NDC80 homolog	73.867	5.6	11.240961	4.0498442	3	3	3	102.6	97.4	0.949	
O15066	Kinesin-like protein KIF3B	85.073	7.69	5.831208	1.4725569	1	1	1	102.6	97.4	0.949	
O60256	Phosphoribosyl pyrophosphate synthase-associated protein 2	40.899	7.44	18.171957	11.653117	3	3	3	102.6	97.4	0.949	1.85E-01
O75915	PRA1 family protein 3	21.6	9.77	55.316744	23.93617	5	11	5	102.6	97.4	0.949	4.38E-01
P06748	Nucleophosmin	32.555	4.78	165.43713	42.857143	12	71	12	102.6	97.4	0.949	1.62E-01

P08559	ate dehydrogenase E1 component subunit alpha, somatic form, mitoch	43.268	8.06	72.865139	27.179487	12	15	12	102.6	97.4	0.949	8.51E-03
P09493	Tropomyosin alpha-1 chain	32.689	4.74	156.78988	35.211268	15	42	6	102.6	97.4	0.949	2.91E-03
P27824	Calnexin	67.526	4.6	179.64225	28.547297	20	74	20	102.6	97.4	0.949	4.15E-02
P35221	Catenin alpha-1	100.009	6.29	248.88869	33.88521	27	43	27	102.6	97.4	0.949	8.45E-02
P43353	Aldehyde dehydrogenase family 3 member B1	51.807	7.62	21.98322	10.683761	4	4	4	102.6	97.4	0.949	6.62E-01
P54709	Sodium/potassium-transporting ATPase subunit beta-3	31.492	8.35	50.395527	26.88172	6	10	6	102.6	97.4	0.949	2.74E-01
P62070	Ras-related protein R-Ras2	23.385	6.01	49.578886	30.392157	5	7	4	102.6	97.4	0.949	5.01E-01
Q13126	S-methyl-5'-thioadenosine phosphorylase	31.216	7.18	41.482313	21.908127	4	5	4	102.6	97.4	0.949	1.94E-01
Q13217	DnaJ homolog subfamily C member 3	57.544	6.15	38.866385	17.65873	9	10	9	102.6	97.4	0.949	6.51E-02
Q15102	Platelet-activating factor acetylhydrolase IB subunit gamma	25.718	6.84	34.098397	23.376623	5	11	5	102.6	97.4	0.949	1.14E-03
Q5T8P6	RNA-binding protein 26	113.527	9.16	56.263669	7.0506455	8	10	6	102.6	97.4	0.949	1.11E-01
Q68EM7	Rho GTPase-activating protein 17	95.377	7.62	37.358623	6.4699205	5	7	5	102.6	97.4	0.949	2.39E-01
Q8NBJ5	Procollagen galactosyltransferase 1	71.59	7.31	54.013261	16.237942	11	16	11	102.6	97.4	0.949	9.77E-01
Q8TDQ7	Glucosamine-6-phosphate isomerase 2	31.065	6.95	25.942759	11.956522	3	6	2	102.6	97.4	0.949	5.49E-01
Q8TF64	PDZ domain-containing protein GIPC3	33.96	5.63	32.401101	18.910256	4	5	3	102.6	97.4	0.949	4.30E-01
Q8WXH0	Nesprin-2	795.944	5.36	139.96465	4.86565	30	33	28	102.6	97.4	0.949	1.46E-05
Q92544	Transmembrane 9 superfamily member 4	74.47	6.54	13.117327	4.9844237	3	4	3	102.6	97.4	0.949	1.16E-01
Q96DG6	Carboxymethylenebutenolidase homolog	28.03	7.18	32.537572	21.22449	7	14	7	102.6	97.4	0.949	2.85E-02
Q96FZ7	Charged multivesicular body protein 6	23.47	5.31	13.563403	16.41791	3	3	3	102.6	97.4	0.949	
Q96JH7	Deubiquitinating protein VCIP135	134.236	7.2	29.879763	6.3011457	5	5	5	102.6	97.4	0.949	
Q9BSC4	Nucleolar protein 10	80.251	8.46	7.5152385	3.4883721	3	3	3	102.6	97.4	0.949	6.05E-01
Q9BTE6	Alanyl-tRNA editing protein Aarsd1	45.451	6.42	5.176006	4.6116505	2	2	2	102.6	97.4	0.949	
Q9H2D6	TRIO and F-actin-binding protein	261.217	8.48	7.5852981	1.3107822	3	5	3	102.6	97.4	0.949	6.82E-02
Q9NP80	Calcium-independent phospholipase A2-gamma	88.421	9.23	24.780189	6.0102302	5	5	3	102.6	97.4	0.949	
Q9UPT8	Zinc finger CCCH domain-containing protein 4	140.169	6.27	27.18207	3.3768227	3	4	3	102.6	97.4	0.949	7.29E-02
Q9Y3I1	F-box only protein 7	58.466	6.55	11.885132	4.5977011	2	3	2	102.6	97.4	0.949	5.48E-01
O00422	Histone deacetylase complex subunit SAP18	17.55	9.35	38.547143	20.261438	4	11	4	102.5	97.5	0.951	3.76E-02
O15061	Synemin	172.663	5.16	49.144267	5.5591054	8	9	8	102.5	97.5	0.951	6.26E-02
O43181	NADH dehydrogenase [ubiquinone] iron-sulfur protein 4, mitochondria	20.095	10.3	23.946601	20	3	5	3	102.5	97.5	0.951	4.50E-02
O43852	Calumenin	37.084	4.64	155.17882	52.063492	14	31	14	102.5	97.5	0.951	1.27E-01
O95861	3'(2'),5'-bisphosphate nucleotidase 1	33.371	5.69	51.85662	23.701299	7	10	7	102.5	97.5	0.951	3.52E-02
P13984	General transcription factor IIF subunit 2	28.363	9.23	26.764429	16.86747	5	6	5	102.5	97.5	0.951	5.62E-02
P15144	Aminopeptidase N	109.471	5.48	61.985453	9.5139607	9	13	9	102.5	97.5	0.951	1.33E-01
P17813	Endoglin	70.533	6.61	21.303599	7.2948328	3	3	3	102.5	97.5	0.951	8.70E-01
P21291	Cysteine and glycine-rich protein 1	20.554	8.57	118.74733	59.067358	9	43	9	102.5	97.5	0.951	9.37E-01
P30838	Aldehyde dehydrogenase, dimeric NADP-preferring	50.363	6.54	12.423324	4.1942605	2	2	2	102.5	97.5	0.951	3.52E-02
P46100	Transcriptional regulator ATRX	282.411	6.58	40.60509	3.4510433	7	7	7	102.5	97.5	0.951	5.64E-02
P49407	Beta-arrestin-1	47.036	6.2	19.203411	4.5454545	2	3	2	102.5	97.5	0.951	1.66E-01
P51572	B-cell receptor-associated protein 31	27.974	8.44	42.913378	26.01626	6	12	6	102.5	97.5	0.951	4.93E-03
P62072	Mitochondrial import inner membrane translocase subunit Tim10	10.326	6.29	21.51422	33.333333	4	5	4	102.5	97.5	0.951	9.39E-02
Q13423	NAD(P) transhydrogenase, mitochondrial	113.823	8.09	296.54909	29.650092	32	68	32	102.5	97.5	0.951	7.56E-04
Q14697	Neutral alpha-glucosidase AB	106.807	6.14	208.12417	25.529661	23	68	23	102.5	97.5	0.951	2.25E-02
Q4G176	Acyl-CoA synthetase family member 3, mitochondrial	64.089	8.37	17.153078	7.8125	4	4	4	102.5	97.5	0.951	
Q4V328	GRIP1-associated protein 1	95.931	5.11	92.236941	19.381688	12	16	12	102.5	97.5	0.951	5.96E-03
Q5VT79	Annexin A8-like protein 1	36.856	5.78	110.54594	32.110092	10	22	1	102.5	97.5	0.951	
Q6XQN6	Nicotinate phosphoribosyltransferase	57.542	5.68	73.767138	17.657993	8	12	8	102.5	97.5	0.951	7.25E-02
Q6XZF7	Dynamin-binding protein	177.236	5.39	12.077265	1.7755231	2	2	2	102.5	97.5	0.951	

Q86W56	Poly(ADP-ribose) glycohydrolase	111.041	6.43	8.3894364	2.0491803	2	2	2	102.5	97.5	0.951	
Q8N1G4	Leucine-rich repeat-containing protein 47	63.434	8.28	89.501339	22.12693	11	21	11	102.5	97.5	0.951	6.19E-01
Q96FJ0	AMSH-like protease	49.751	7.23	13.480458	7.1100917	2	2	2	102.5	97.5	0.951	2.93E-01
Q9BRF8	Serine/threonine-protein phosphatase CPPED1	35.526	6.2	23.991685	10.828025	3	4	3	102.5	97.5	0.951	2.93E-01
Q9BUR5	MICOS complex subunit MIC26	22.271	9.13	6.0820221	7.5757576	1	1	1	102.5	97.5	0.951	
Q9BW71	HIRA-interacting protein 3	61.92	8.54	26.064339	10.251799	5	6	5	102.5	97.5	0.951	9.71E-01
Q9H425	Uncharacterized protein C1orf198	36.324	5.72	17.136352	15.59633	4	4	4	102.5	97.5	0.951	5.38E-01
Q9HBL8	NmrA-like family domain-containing protein 1	33.323	7.52	19.67332	9.0301003	3	5	3	102.5	97.5	0.951	1.23E-01
Q9NXF1	Testis-expressed sequence 10 protein	105.608	9.36	24.780126	4.6286329	3	3	3	102.5	97.5	0.951	
Q9NYL9	Tropomodulin-3	39.57	5.19	124.605	46.306818	13	20	13	102.5	97.5	0.951	2.38E-01
O00764	Pyridoxal kinase	35.08	6.13	57.534335	23.397436	9	13	9	102.4	97.6	0.953	1.29E-04
O14745	Na(+)/H(+) exchange regulatory cofactor NHE-RF1	38.845	5.77	82.59754	34.636872	13	22	13	102.4	97.6	0.953	1.61E-01
O75306	NADH dehydrogenase [ubiquinone] iron-sulfur protein 2, mitochondria	52.512	7.55	63.437793	22.24622	8	15	8	102.4	97.6	0.953	8.43E-01
O75369	Filamin-B	277.99	5.73	813.9176	40.046118	89	166	82	102.4	97.6	0.953	4.09E-04
O95573	Long-chain-fatty-acid--CoA ligase 3	80.368	8.38	162.66394	30.833333	17	31	15	102.4	97.6	0.953	5.50E-02
P17050	Alpha-N-acetylgalactosaminidase	46.534	5.19	14.604658	2.919708	1	2	1	102.4	97.6	0.953	
P28288	ATP-binding cassette sub-family D member 3	75.428	9.36	52.801559	11.98786	8	11	7	102.4	97.6	0.953	3.92E-01
P50583	Bis(5'-nucleosyl)-tetraphosphatase [asymmetrical]	16.819	5.35	11.843277	20.408163	2	2	2	102.4	97.6	0.953	2.63E-02
P50914	60S ribosomal protein L14	23.417	10.93	39.157782	21.860465	5	14	5	102.4	97.6	0.953	3.12E-03
P55211	Caspase-9	46.251	6.05	15.265687	8.1730769	3	3	3	102.4	97.6	0.953	5.10E-02
P62249	40S ribosomal protein S16	16.435	10.21	45.719227	52.054795	9	14	9	102.4	97.6	0.953	5.15E-04
P63313	Thymosin beta-10	5.023	5.36	10.685062	18.181818	2	6	1	102.4	97.6	0.953	
Q07065	Cytoskeleton-associated protein 4	65.983	5.92	305.18904	54.817276	29	63	28	102.4	97.6	0.953	4.41E-07
Q5T8D3	Acyl-CoA-binding domain-containing protein 5	60.054	5.33	12.380743	5.0561798	2	2	2	102.4	97.6	0.953	
Q86W42	THO complex subunit 6 homolog	37.511	7.43	44.201561	28.152493	7	9	7	102.4	97.6	0.953	4.05E-02
Q8IXB1	DnaJ homolog subfamily C member 10	91.021	7.18	30.459199	5.8007566	5	10	5	102.4	97.6	0.953	3.07E-02
Q8N697	Solute carrier family 15 member 4	61.993	9	6.5081049	3.812825	2	2	2	102.4	97.6	0.953	
Q8NEM2	SHC SH2 domain-binding protein 1	75.642	4.75	51.722248	16.964286	10	11	10	102.4	97.6	0.953	9.81E-01
Q92887	Canalicular multispecific organic anion transporter 1	174.096	8.32	6.3685467	0.9708738	2	2	2	102.4	97.6	0.953	
Q96EY8	Cob(I)yrinic acid a,c-diamide adenosyltransferase, mitochondrial	27.371	8.6	51.434788	18.4	3	6	3	102.4	97.6	0.953	1.56E-03
Q96JC1	Vam6/Vps39-like protein	101.744	6.99	4.8931295	1.5801354	1	1	1	102.4	97.6	0.953	
Q9BTT4	Mediator of RNA polymerase II transcription subunit 10	15.678	6.19	3.4489161	9.6296296	1	1	1	102.4	97.6	0.953	
Q9BUL9	Ribonuclease P protein subunit p25	20.62	9.61	9.4955987	9.0452261	2	2	2	102.4	97.6	0.953	7.03E-02
Q9BWJ5	Splicing factor 3B subunit 5	10.129	6.35	25.006893	37.209302	3	6	3	102.4	97.6	0.953	5.56E-01
Q9BWM7	Sideroflexin-3	35.956	9.09	55.271074	18.769231	5	11	5	102.4	97.6	0.953	6.38E-01
Q9GZL7	Ribosome biogenesis protein WDR12	47.678	5.9	14.762466	9.2198582	4	4	4	102.4	97.6	0.953	1.09E-01
Q9H9Y6	DNA-directed RNA polymerase I subunit RPA2	128.146	7.83	32.692777	5.9030837	6	7	6	102.4	97.6	0.953	1.41E-02
Q9NX46	Poly(ADP-ribose) glycohydrolase ARH3	38.922	5.07	30.8353	17.07989	5	5	5	102.4	97.6	0.953	1.93E-01
Q9NXR7	BRCA1-A complex subunit BRE	43.524	5.81	9.5603081	6.2663185	2	2	2	102.4	97.6	0.953	
Q9NYY8	FAST kinase domain-containing protein 2	81.41	8.05	26.810403	4.5070423	2	3	2	102.4	97.6	0.953	1.14E-01
Q9P0W2	matrix-associated actin-dependent regulator of chromatin subfamily E	35.791	9.35	15.401366	6.6246057	2	3	2	102.4	97.6	0.953	6.91E-01
Q9P1Y6	PHD and RING finger domain-containing protein 1	178.557	8.95	7.8236688	1.7586416	3	3	3	102.4	97.6	0.953	1.66E-01
Q9Y3D0	Mitotic spindle-associated MMXD complex subunit MIP18	17.652	5.19	12.851339	17.177914	2	2	2	102.4	97.6	0.953	3.20E-02
Q9Y4U1	Methylmalonic aciduria and homocystinuria type C protein	31.708	7.81	4.2343314	3.1914894	1	1	1	102.4	97.6	0.953	
O43813	LanC-like protein 1	45.254	7.75	25.398712	15.037594	5	7	5	102.3	97.7	0.955	2.27E-01
O75976	Carboxypeptidase D	152.835	6.05	13.947527	2.5362319	4	4	4	102.3	97.7	0.955	8.29E-02
P00441	Superoxide dismutase [Cu-Zn]	15.926	6.13	67.328747	46.753247	9	24	9	102.3	97.7	0.955	2.42E-02

P13473	Lysosome-associated membrane glycoprotein 2	44.932	5.63	15.420311	7.0731707	3	3	3	102.3	97.7	0.955	3.52E-02
P20340	Ras-related protein Rab-6A	23.578	5.54	69.110946	28.846154	5	15	4	102.3	97.7	0.955	1.06E-01
P21964	Catechol O-methyltransferase	30.018	5.47	36.461206	25.461255	7	8	7	102.3	97.7	0.955	1.73E-01
P22413	Ectonucleotide pyrophosphatase/phosphodiesterase family member 1	104.857	7.14	10.482741	2.1621622	2	3	2	102.3	97.7	0.955	
P27695	DNA-(apurinic or apyrimidinic site) lyase	35.532	8.12	140.36248	40.880503	13	22	13	102.3	97.7	0.955	3.87E-01
P27797	Calreticulin	48.112	4.44	159.71118	30.455635	14	49	14	102.3	97.7	0.955	8.66E-04
P32519	ETS-related transcription factor Elf-1	67.456	5.21	10.472923	5.0080775	2	2	2	102.3	97.7	0.955	4.00E-02
P35232	Prohibitin	29.786	5.76	119.26139	50.367647	13	31	13	102.3	97.7	0.955	1.60E-04
P41743	Protein kinase C iota type	68.218	5.85	42.133804	11.073826	5	8	5	102.3	97.7	0.955	6.37E-01
P52209	6-phosphogluconate dehydrogenase, decarboxylating	53.106	7.23	204.5415	39.958592	18	43	18	102.3	97.7	0.955	2.92E-03
P55854	Small ubiquitin-related modifier 3	11.63	5.49	23.982423	20.38835	2	5	1	102.3	97.7	0.955	
P56545	C-terminal-binding protein 2	48.914	6.95	58.773872	21.123596	9	13	5	102.3	97.7	0.955	1.50E-03
P62158	Calmodulin	16.827	4.22	110.90143	55.033557	9	46	9	102.3	97.7	0.955	3.88E-01
Q14353	Guanidinoacetate N-methyltransferase	26.301	6.14	27.36817	17.372881	3	5	3	102.3	97.7	0.955	2.97E-01
Q15075	Early endosome antigen 1	162.367	5.68	222.00221	23.03331	28	38	28	102.3	97.7	0.955	8.44E-04
Q1KMD3	Heterogeneous nuclear ribonucleoprotein U-like protein 2	85.052	4.91	160.67307	27.443106	18	31	18	102.3	97.7	0.955	1.13E-04
Q69YQ0	Cytospin-A	124.525	5.72	24.914732	4.8343778	5	7	3	102.3	97.7	0.955	
Q6IAA8	Ragulator complex protein LAMTOR1	17.734	5.15	21.113467	15.52795	2	3	2	102.3	97.7	0.955	
Q6ZU65	Ubinuclein-2	145.998	9.19	8.4586191	1.7074981	2	2	2	102.3	97.7	0.955	
Q6ZUT6	Uncharacterized protein C15orf52	57.29	9.13	4.8404328	1.8726592	1	1	1	102.3	97.7	0.955	
Q86TB9	Protein PAT1 homolog 1	86.796	6.67	11.334932	4.2857143	3	3	3	102.3	97.7	0.955	
Q8IV08	Phospholipase D3	54.671	6.47	18.254673	8.1632653	4	5	4	102.3	97.7	0.955	
Q8NB90	Spermatogenesis-associated protein 5	97.843	5.66	37.96755	4.2553191	3	7	2	102.3	97.7	0.955	3.45E-03
Q8TE02	Elongator complex protein 5	34.819	4.97	9.1422455	6.6455696	1	1	1	102.3	97.7	0.955	
Q96CM8	Acyl-CoA synthetase family member 2, mitochondrial	68.081	7.55	12.190017	3.5772358	2	2	2	102.3	97.7	0.955	1.95E-01
Q99704	Docking protein 1	52.359	6.47	3.6115435	1.8711019	1	1	1	102.3	97.7	0.955	
Q9BVM2	Protein DPCD	23.225	9.03	14.944126	13.793103	3	3	3	102.3	97.7	0.955	5.61E-03
Q9BXB5	Oxysterol-binding protein-related protein 10	83.917	8.31	19.13516	5.3664921	3	3	2	102.3	97.7	0.955	4.99E-01
Q9GZS1	DNA-directed RNA polymerase I subunit RPA49	53.928	8.56	3.2816487	3.3264033	2	2	2	102.3	97.7	0.955	2.60E-01
Q9H061	Transmembrane protein 126A	21.513	9.26	13.758197	15.897436	2	2	2	102.3	97.7	0.955	
Q9H0U6	39S ribosomal protein L18, mitochondrial	20.564	9.54	7.6198148	10	2	2	2	102.3	97.7	0.955	
Q9H6Z4	Ran-binding protein 3	60.173	4.78	36.525071	9.1710758	4	5	4	102.3	97.7	0.955	5.07E-01
Q9UBQ0	Vacuolar protein sorting-associated protein 29	20.493	6.79	36.482364	21.978022	4	7	4	102.3	97.7	0.955	2.47E-04
O00471	Exocyst complex component 5	81.801	6.71	37.821935	6.2146893	4	6	4	102.2	97.8	0.957	5.88E-01
O43772	Mitochondrial carnitine/acylcarnitine carrier protein	32.922	9.41	17.489111	13.953488	5	5	5	102.2	97.8	0.957	4.86E-03
O60231	ıtative pre-mRNA-splicing factor ATP-dependent RNA helicase DHX	119.189	6.8	46.704154	7.4927954	7	9	7	102.2	97.8	0.957	6.31E-03
O60645	Exocyst complex component 3	86.79	6.11	52.597795	14.153439	8	9	8	102.2	97.8	0.957	3.72E-01
O60888	Protein CutA	19.104	5.5	65.556405	23.463687	4	12	4	102.2	97.8	0.957	7.97E-01
O75489	JADH dehydrogenase [ubiquinone] iron-sulfur protein 3, mitochondria	30.223	7.5	36.407109	29.924242	6	13	6	102.2	97.8	0.957	2.47E-02
O95271	Tankyrase-1	141.95	7.05	6.2439728	0.7535795	1	1	1	102.2	97.8	0.957	
O95486	Protein transport protein Sec24A	119.674	7.66	22.377974	5.3064959	5	5	5	102.2	97.8	0.957	
O95833	Chloride intracellular channel protein 3	26.632	6.43	44.349972	40.254237	9	11	9	102.2	97.8	0.957	2.62E-01
P06756	Integrin alpha-V	115.964	5.68	39.538494	7.9198473	8	10	8	102.2	97.8	0.957	4.52E-04
P11766	Alcohol dehydrogenase class-3	39.698	7.49	114.6042	36.898396	12	26	12	102.2	97.8	0.957	7.74E-02
P17987	T-complex protein 1 subunit alpha	60.306	6.11	165.5854	36.690647	17	37	17	102.2	97.8	0.957	3.64E-01
P23786	Carnitine O-palmitoyltransferase 2, mitochondrial	73.73	8.18	83.70676	20.06079	11	16	11	102.2	97.8	0.957	2.00E-03
P25815	Protein S100-P	10.393	4.88	27.261075	24.210526	2	6	2	102.2	97.8	0.957	1.02E-04

P29966	Myristoylated alanine-rich C-kinase substrate	31.536	4.45	84.884388	21.686747	5	12	5	102.2	97.8	0.957	1.04E-02
P33992	DNA replication licensing factor MCM5	82.233	8.37	208.22606	34.059946	21	43	21	102.2	97.8	0.957	7.15E-02
P46934	E3 ubiquitin-protein ligase NEDD4	149.022	6.58	29.728275	4.0181956	4	5	4	102.2	97.8	0.957	9.49E-01
P51608	Methyl-CpG-binding protein 2	52.409	9.95	12.659509	2.8806584	1	2	1	102.2	97.8	0.957	
P54105	Methylosome subunit pICln	26.199	4.11	21.904017	16.033755	3	9	3	102.2	97.8	0.957	7.37E-01
P57772	Selenocysteine-specific elongation factor	65.263	8.35	34.211912	8.8926174	5	6	5	102.2	97.8	0.957	1.33E-01
P62495	Eukaryotic peptide chain release factor subunit 1	49	5.71	112.35252	28.146453	11	24	11	102.2	97.8	0.957	1.69E-03
Q00325	Phosphate carrier protein, mitochondrial	40.069	9.38	51.676583	28.176796	11	20	11	102.2	97.8	0.957	7.54E-01
Q01581	Hydroxymethylglutaryl-CoA synthase, cytoplasmic	57.257	5.41	132.12966	24.615385	12	28	12	102.2	97.8	0.957	4.88E-01
Q01650	Large neutral amino acids transporter small subunit 1	54.974	7.72	26.00399	6.3116371	4	8	4	102.2	97.8	0.957	1.32E-01
Q15018	BRISC complex subunit Abro1	46.872	6.21	19.541865	9.6385542	4	4	4	102.2	97.8	0.957	1.75E-01
Q8IX04	Ubiquitin-conjugating enzyme E2 variant 3	52.231	7.09	6.1632586	3.6093418	2	2	1	102.2	97.8	0.957	
Q8N612	FTS and Hook-interacting protein	105.502	6.77	4.747147	0.9259259	1	1	1	102.2	97.8	0.957	
Q96Q05	Trafficking protein particle complex subunit 9	128.449	6.62	10.466924	0.9581882	1	2	1	102.2	97.8	0.957	
Q96RU3	Formin-binding protein 1	71.262	5.72	76.44201	22.528363	12	14	12	102.2	97.8	0.957	8.66E-01
Q9BXW7	Cat eye syndrome critical region protein 5	46.292	8.13	68.967035	29.787234	9	14	9	102.2	97.8	0.957	2.91E-01
Q9H4L4	Sentrin-specific protease 3	64.969	8.56	25.893917	6.6202091	3	6	3	102.2	97.8	0.957	9.85E-01
Q9H6T0	Epithelial splicing regulatory protein 2	78.351	6.71	24.455824	5.914718	4	5	4	102.2	97.8	0.957	1.57E-01
Q9UBX3	Mitochondrial dicarboxylate carrier	31.262	9.54	64.062047	27.526132	7	11	7	102.2	97.8	0.957	2.91E-05
Q9UFC0	Leucine-rich repeat and WD repeat-containing protein 1	70.816	7.21	28.656838	11.128284	5	6	5	102.2	97.8	0.957	1.15E-01
Q9UMX1	Suppressor of fused homolog	53.913	5.33	12.432406	5.5785124	2	2	2	102.2	97.8	0.957	
Q9Y237	Peptidyl-prolyl cis-trans isomerase NIMA-interacting 4	13.801	9.77	10.08546	16.793893	2	3	2	102.2	97.8	0.957	2.58E-01
A4D1P6	WD repeat-containing protein 91	83.292	6.58	7.119867	2.1419009	2	2	2	102.1	97.9	0.959	
O14976	Cyclin-G-associated kinase	143.1	5.73	9.5346861	1.6781083	3	3	3	102.1	97.9	0.959	9.13E-02
O95881	Thioredoxin domain-containing protein 12	19.194	5.4	23.139528	30.232558	4	4	4	102.1	97.9	0.959	
P01891	HLA class I histocompatibility antigen, A-68 alpha chain	40.883	6.7	81.736736	22.465753	7	20	4	102.1	97.9	0.959	3.10E-02
P07741	Adenine phosphoribosyltransferase	19.595	6.02	48.455596	42.777778	7	12	7	102.1	97.9	0.959	1.62E-02
P15924	Desmoplakin	331.569	6.81	457.42291	23.058168	63	87	63	102.1	97.9	0.959	2.16E-05
P18206	Vinculin	123.722	5.66	852.98456	61.375661	64	232	64	102.1	97.9	0.959	5.44E-03
P29401	Transketolase	67.835	7.66	274.96946	40.288925	26	79	26	102.1	97.9	0.959	1.38E-03
P30046	D-dopachrome decarboxylase	12.704	7.3	68.484203	45.762712	6	18	6	102.1	97.9	0.959	6.06E-09
P36871	Phosphoglucomutase-1	61.411	6.76	71.495032	22.953737	11	14	11	102.1	97.9	0.959	4.10E-03
P49411	Elongation factor Tu, mitochondrial	49.51	7.61	268.29551	58.185841	22	61	22	102.1	97.9	0.959	1.87E-02
P61020	Ras-related protein Rab-5B	23.692	8.13	30.206394	15.813953	3	6	1	102.1	97.9	0.959	
Q02809	Procollagen-lysine,2-oxoglutarate 5-dioxygenase 1	83.497	6.95	147.44172	23.658872	14	23	13	102.1	97.9	0.959	1.20E-01
Q12929	Epidermal growth factor receptor kinase substrate 8	91.824	7.5	11.363652	3.0413625	2	2	2	102.1	97.9	0.959	
Q13907	Isopentenyl-diphosphate Delta-isomerase 1	26.302	6.34	32.740447	22.026432	5	7	5	102.1	97.9	0.959	8.60E-01
Q15286	Ras-related protein Rab-35	23.011	8.29	61.845799	35.323383	8	20	5	102.1	97.9	0.959	2.74E-01
Q16512	Serine/threonine-protein kinase N1	103.868	6.37	18.266984	5.3078556	5	5	4	102.1	97.9	0.959	1.71E-01
Q16775	Hydroxyacylglutathione hydrolase, mitochondrial	33.784	8.12	32.480592	18.506494	5	8	5	102.1	97.9	0.959	5.68E-02
Q4KMP7	TBC1 domain family member 10B	87.145	9.19	12.655934	3.8366337	2	3	2	102.1	97.9	0.959	3.33E-01
Q86T03	Type 1 phosphatidylinositol 4,5-bisphosphate 4-phosphatase	29.45	8.91	6.8683813	3.9711191	1	1	1	102.1	97.9	0.959	
Q8WUY1	Protein THEM6	23.85	9.55	12.024585	12.019231	2	2	2	102.1	97.9	0.959	
Q92599	Septin-8	55.721	6.28	54.254908	15.52795	7	11	3	102.1	97.9	0.959	6.22E-02
Q92817	Envoplakin	231.463	6.96	67.434809	6.9355632	12	12	11	102.1	97.9	0.959	3.76E-02
Q99623	Prohibitin-2	33.276	9.83	127.62719	49.16388	14	32	14	102.1	97.9	0.959	1.96E-05
Q9BWG4	Single-stranded DNA-binding protein 4	39.362	6.8	10.090911	5.4545455	2	2	1	102.1	97.9	0.959	

Q9H0L4	Cleavage stimulation factor subunit 2 tau variant	64.396	7.25	92.874802	20.779221	9	14	4	102.1	97.9	0.959	5.50E-02
Q9H1H9	Kinesin-like protein KIF13A	202.183	5.6	43.041362	4.2105263	6	8	5	102.1	97.9	0.959	2.67E-01
Q9NV56	MRG/MORF4L-binding protein	22.403	5.83	10.099253	6.372549	1	2	1	102.1	97.9	0.959	
Q9NV92	NEDD4 family-interacting protein 2	36.367	5.08	10.977572	4.1666667	1	1	1	102.1	97.9	0.959	
Q9ULC4	Malignant T-cell-amplified sequence 1	20.542	8.82	75.594966	46.961326	7	11	7	102.1	97.9	0.959	3.60E-01
Q9Y232	Chromodomain Y-like protein	66.44	9.45	16.723202	2.6755853	2	2	2	102.1	97.9	0.959	
A6NHL2	Tubulin alpha chain-like 3	49.877	6.05	47.7488	9.4170404	4	14	1	102	98	0.961	
O00468	Agrin	217.092	6.39	44.464124	4.6444122	7	9	7	102	98	0.961	8.59E-02
O15144	Actin-related protein 2/3 complex subunit 2	34.311	7.36	56.985039	20.666667	7	14	7	102	98	0.961	7.31E-06
O43670	BUB3-interacting and GLEBS motif-containing protein ZNF207	50.717	9.1	37.251116	12.343096	5	9	5	102	98	0.961	6.39E-01
O60503	Adenylate cyclase type 9	150.604	7.34	12.887933	1.8477458	2	3	2	102	98	0.961	4.96E-03
O60832	H/ACA ribonucleoprotein complex subunit 4	57.638	9.42	73.7181	15.953307	8	14	8	102	98	0.961	8.73E-01
O75414	Nucleoside diphosphate kinase 6	21.129	8.32	12.516127	11.290323	2	3	2	102	98	0.961	5.13E-02
O75717	WD repeat and HMG-box DNA-binding protein 1	125.888	5.62	82.698708	11.603189	10	12	10	102	98	0.961	3.63E-02
P00390	Glutathione reductase, mitochondrial	56.221	8.5	67.699185	20.498084	10	16	10	102	98	0.961	9.06E-01
P00558	Phosphoglycerate kinase 1	44.586	8.1	474.01966	67.625899	29	173	24	102	98	0.961	3.55E-03
P09132	Signal recognition particle 19 kDa protein	16.145	9.85	28.297536	20.833333	3	3	3	102	98	0.961	3.05E-01
P15735	Phosphorylase b kinase gamma catalytic chain, liver/testis isoform	46.413	6.38	10.744075	5.91133	2	2	2	102	98	0.961	
P16152	Carbonyl reductase [NADPH] 1	30.356	8.32	121.09798	64.259928	13	22	10	102	98	0.961	4.45E-01
P30043	Flavin reductase (NADPH)	22.105	7.65	78.638839	41.262136	8	19	8	102	98	0.961	3.30E-01
P33993	DNA replication licensing factor MCM7	81.257	6.46	194.61203	38.108484	23	42	23	102	98	0.961	2.08E-01
P34913	Bifunctional epoxide hydrolase 2	62.575	6.28	6.2647206	1.6216216	1	1	1	102	98	0.961	
P43246	DNA mismatch repair protein Msh2	104.677	5.77	159.94388	23.447537	22	39	22	102	98	0.961	2.87E-01
P60228	Eukaryotic translation initiation factor 3 subunit E	52.187	6.04	114.59364	34.157303	14	25	14	102	98	0.961	1.08E-01
P98160	3asement membrane-specific heparan sulfate proteoglycan core protei	468.532	6.51	20.871622	0.8881804	3	3	3	102	98	0.961	5.80E-01
Q05932	Folylpolyglutamate synthase, mitochondrial	64.568	7.94	29.72007	10.391823	5	6	5	102	98	0.961	1.01E-02
Q12857	Nuclear factor 1 A-type	55.909	8.44	6.9763361	2.5540275	1	1	1	102	98	0.961	
Q15738	Sterol-4-alpha-carboxylate 3-dehydrogenase, decarboxylating	41.874	8.06	62.981682	31.099196	10	16	10	102	98	0.961	8.53E-03
Q16186	Proteasomal ubiquitin receptor ADRM1	42.127	5.07	88.912573	21.867322	8	23	8	102	98	0.961	9.00E-01
Q6FI81	Anamorsin	33.561	5.62	56.703918	30.128205	9	13	9	102	98	0.961	6.49E-01
Q8IVD9	NudC domain-containing protein 3	40.797	5.25	3.5015881	3.8781163	2	2	2	102	98	0.961	
Q8IWB1	Inositol 1,4,5-trisphosphate receptor-interacting protein	62.019	5.88	3.3454384	2.1937843	1	1	1	102	98	0.961	
Q8TDN6	Ribosome biogenesis protein BRX1 homolog	41.375	9.92	49.379664	9.0651558	6	11	6	102	98	0.961	3.20E-01
Q92696	Geranylgeranyl transferase type-2 subunit alpha	65.03	5.67	12.056745	2.1164021	1	2	1	102	98	0.961	
Q96AA3	Protein RFT1 homolog	60.296	8.85	3.620332	1.663586	1	1	1	102	98	0.961	
Q96E39	RNA binding motif protein, X-linked-like-1	42.116	9.89	66.677742	25.641026	12	29	1	102	98	0.961	
Q96G23	Ceramide synthase 2	44.847	8.98	10.992105	6.5789474	2	2	2	102	98	0.961	
Q96TC7	Regulator of microtubule dynamics protein 3	52.086	5.1	18.3433	4.4680851	2	3	2	102	98	0.961	
Q99536	Synaptic vesicle membrane protein VAT-1 homolog	41.893	6.29	112.09213	22.900763	8	17	8	102	98	0.961	3.60E-04
Q9BW27	Nuclear pore complex protein Nup85	74.971	5.55	55.180816	16.310976	8	10	8	102	98	0.961	2.14E-01
Q9H3N1	Thioredoxin-related transmembrane protein 1	31.771	4.98	18.218278	12.142857	3	4	3	102	98	0.961	4.86E-01
Q9H479	Fructosamine-3-kinase	35.149	7.55	66.097197	30.097087	7	10	7	102	98	0.961	7.68E-03
Q9NXS2	Glutaminyl-peptide cyclotransferase-like protein	42.897	9.82	11.948601	5.7591623	2	3	2	102	98	0.961	
Q9UBR2	Cathepsin Z	33.846	7.11	30.504219	15.181518	4	5	4	102	98	0.961	5.52E-01
Q9UK22	F-box only protein 2	33.307	4.37	17.133635	4.0540541	1	2	1	102	98	0.961	
Q9Y276	Mitochondrial chaperone BCS1	47.504	8.5	16.841263	4.7732697	2	2	2	102	98	0.961	
Q9Y6D5	Brefeldin A-inhibited guanine nucleotide-exchange protein 2	201.909	6.33	28.991878	3.697479	6	6	4	102	98	0.961	4.37E-02

O14562	Ubiquitin domain-containing protein UBFD1	33.361	5.77	23.584966	11.326861	3	6	3	101.9	98.1	0.963	8.09E-02
O15042	U2 snRNP-associated SURP motif-containing protein	118.219	8.47	51.797634	7.191448	7	9	7	101.9	98.1	0.963	4.19E-01
O94808	Glutamine--fructose-6-phosphate aminotransferase [isomerizing] 2	76.882	7.37	60.678806	14.956012	8	9	6	101.9	98.1	0.963	5.86E-01
P00338	L-lactate dehydrogenase A chain	36.665	8.27	161.34224	39.759036	16	83	13	101.9	98.1	0.963	8.99E-05
P01111	GTPase NRas	21.216	5.17	35.859109	30.15873	5	8	3	101.9	98.1	0.963	9.88E-01
P05186	Alkaline phosphatase, tissue-nonspecific isozyme	57.269	6.67	197.22156	29.770992	14	67	14	101.9	98.1	0.963	2.93E-01
P08133	Annexin A6	75.826	5.6	180.65735	38.187221	27	44	27	101.9	98.1	0.963	2.48E-01
P09012	U1 small nuclear ribonucleoprotein A	31.259	9.83	25.967325	17.375887	6	8	4	101.9	98.1	0.963	5.57E-01
P09661	U2 small nuclear ribonucleoprotein A'	28.398	8.62	62.121777	29.019608	8	11	8	101.9	98.1	0.963	7.78E-01
P0DMN0	Sulfotransferase 1A4	34.174	6.01	24.549745	19.661017	5	5	2	101.9	98.1	0.963	
P14314	Glucosidase 2 subunit beta	59.388	4.41	158.18841	25.757576	14	38	14	101.9	98.1	0.963	1.05E-03
P18754	Regulator of chromosome condensation	44.941	7.52	92.572518	23.04038	8	18	8	101.9	98.1	0.963	3.97E-02
P19440	Gamma-glutamyltranspeptidase 1	61.372	7.12	33.134019	9.1388401	4	5	4	101.9	98.1	0.963	
P20700	Lamin-B1	66.368	5.16	348.22377	54.095563	35	72	30	101.9	98.1	0.963	9.90E-01
P23634	Plasma membrane calcium-transporting ATPase 4	137.833	6.6	101.04719	11.925866	12	19	4	101.9	98.1	0.963	1.32E-01
P35613	Basigin	42.174	5.66	38.144017	21.558442	6	7	6	101.9	98.1	0.963	9.81E-02
P42345	Serine/threonine-protein kinase mTOR	288.707	7.17	29.240804	3.4915653	9	9	8	101.9	98.1	0.963	8.68E-02
P46379	Large proline-rich protein BAG6	119.334	5.6	76.489341	15.459364	13	15	13	101.9	98.1	0.963	1.01E-03
P56277	Cx9C motif-containing protein 4	7.742	8.18	2.9918258	10.294118	1	1	1	101.9	98.1	0.963	
P62745	Rho-related GTP-binding protein RhoB	22.109	5.24	34.755289	31.632653	5	6	4	101.9	98.1	0.963	
P62979	Ubiquitin-40S ribosomal protein S27a	17.953	9.64	119.55037	53.846154	11	52	11	101.9	98.1	0.963	8.17E-01
Q13438	Protein OS-9	75.515	4.87	6.2889524	2.0989505	1	1	1	101.9	98.1	0.963	
Q15208	Serine/threonine-protein kinase 38	54.155	7.15	43.350233	15.483871	7	9	7	101.9	98.1	0.963	1.06E-01
Q16555	Dihydropyrimidinase-related protein 2	62.255	6.38	165.63617	34.965035	16	32	13	101.9	98.1	0.963	1.37E-03
Q16762	Thiosulfate sulfurtransferase	33.408	7.25	26.265506	10.43771	3	4	3	101.9	98.1	0.963	
Q16875	6-phosphofructo-2-kinase/fructose-2,6-bisphosphatase 3	59.571	8.21	5.768021	2.1153846	1	1	1	101.9	98.1	0.963	
Q6NZ67	Mitotic-spindle organizing protein 2B	16.216	10.15	8.4748256	13.924051	1	1	1	101.9	98.1	0.963	
Q7Z333	Probable helicase senataxin	302.689	7.17	23.004938	1.9798282	6	8	5	101.9	98.1	0.963	4.00E-01
Q8TF42	Ubiquitin-associated and SH3 domain-containing protein B	72.649	6.93	57.408226	15.100154	8	11	8	101.9	98.1	0.963	8.89E-01
Q96A35	39S ribosomal protein L24, mitochondrial	24.899	9.29	8.8777841	6.0185185	1	1	1	101.9	98.1	0.963	
Q96NT1	Nucleosome assembly protein 1-like 5	19.581	4.21	6.4610494	6.5934066	1	1	1	101.9	98.1	0.963	
Q96NU1	Sterile alpha motif domain-containing protein 11	72.663	7.59	12.830197	2.4963289	1	3	1	101.9	98.1	0.963	
Q96RE7	Nucleus accumbens-associated protein 1	57.222	5.74	27.933978	11.195446	4	4	4	101.9	98.1	0.963	3.54E-02
Q9BSH4	Translational activator of cytochrome c oxidase 1	32.457	8.13	3.9578184	3.3670034	1	1	1	101.9	98.1	0.963	
Q9BVG4	Protein PBDC1	26.041	4.79	14.847968	10.729614	3	3	3	101.9	98.1	0.963	
Q9BW92	Threonine--tRNA ligase, mitochondrial	80.985	7.3	29.583191	8.2172702	6	6	6	101.9	98.1	0.963	4.98E-02
Q9C0D3	Protein zyg-11 homolog B	83.867	6.86	6.6844495	1.7473118	1	1	1	101.9	98.1	0.963	
Q9GZY8	Mitochondrial fission factor	38.441	8.95	82.318856	30.994152	6	11	6	101.9	98.1	0.963	9.70E-01
Q9H6S3	Epidermal growth factor receptor kinase substrate 8-like protein 2	80.57	6.84	43.504525	11.608392	8	11	8	101.9	98.1	0.963	3.56E-01
Q9UGV2	Protein NDRG3	41.382	5.31	41.814962	18.666667	6	8	5	101.9	98.1	0.963	3.48E-01
Q9UI09	NADH dehydrogenase [ubiquinone] 1 alpha subcomplex subunit 12	17.104	9.63	22.188785	26.206897	3	5	3	101.9	98.1	0.963	6.70E-01
Q9ULR0	Pre-mRNA-splicing factor ISY1 homolog	32.972	5.17	23.887189	14.035088	3	3	3	101.9	98.1	0.963	1.32E-01
Q9Y4Z0	U6 snRNA-associated Sm-like protein LSm4	15.34	9.99	16.696767	15.827338	2	5	2	101.9	98.1	0.963	6.02E-01
A1L0T0	Acetolactate synthase-like protein	67.825	8.15	42.656004	10.443038	5	7	5	101.8	98.2	0.965	9.92E-02
O14880	Microsomal glutathione S-transferase 3	16.506	9.38	6.7661882	5.2631579	1	3	1	101.8	98.2	0.965	
O14950	Myosin regulatory light chain 12B	19.767	4.84	92.932232	62.209302	8	20	3	101.8	98.2	0.965	3.04E-02
O15067	Phosphoribosylformylglycinamidine synthase	144.643	5.76	113.85236	15.844544	20	30	20	101.8	98.2	0.965	6.87E-01

O75781	Paralemmin-1	42.05	5	4.0132283	2.5839793	1	1	1	101.8	98.2	0.965	
O94888	UBX domain-containing protein 7	54.828	5.16	38.436751	13.496933	6	7	6	101.8	98.2	0.965	6.80E-01
O95352	Ubiquitin-like modifier-activating enzyme ATG7	77.909	6.24	35.358653	9.6728307	6	7	6	101.8	98.2	0.965	1.50E-01
P02545	Prelamin-A/C	74.095	7.02	488.21074	66.566265	46	148	44	101.8	98.2	0.965	9.33E-02
P17844	Probable ATP-dependent RNA helicase DDX5	69.105	8.92	246.61457	39.087948	28	75	19	101.8	98.2	0.965	9.80E-01
P35573	Glycogen debranching enzyme	174.652	6.76	55.196945	6.2010444	10	13	10	101.8	98.2	0.965	5.66E-02
P38935	DNA-binding protein SMUBP-2	109.082	8.97	5.5276825	1.1077543	1	1	1	101.8	98.2	0.965	
P49821	NADH dehydrogenase [ubiquinone] flavoprotein 1, mitochondrial	50.785	8.21	32.931548	16.163793	7	7	7	101.8	98.2	0.965	4.27E-01
P52630	Signal transducer and activator of transcription 2	97.855	5.49	15.615122	4.1128085	3	3	3	101.8	98.2	0.965	
P61160	Actin-related protein 2	44.732	6.74	89.328549	28.172589	11	31	10	101.8	98.2	0.965	4.29E-02
P62714	Serine/threonine-protein phosphatase 2A catalytic subunit beta isoform	35.552	5.43	96.678597	31.71521	7	14	1	101.8	98.2	0.965	
Q03013	Glutathione S-transferase Mu 4	25.545	5.9	74.626804	51.834862	11	18	2	101.8	98.2	0.965	3.52E-02
Q04323	UBX domain-containing protein 1	33.305	5.25	16.357591	12.121212	4	5	4	101.8	98.2	0.965	9.80E-01
Q14061	Cytochrome c oxidase copper chaperone	6.91	7.24	16.642151	57.142857	3	4	3	101.8	98.2	0.965	
Q5TC12	ATP synthase mitochondrial F1 complex assembly factor 1	36.414	7.96	20.597253	12.5	4	5	4	101.8	98.2	0.965	2.73E-02
Q6I9Y2	THO complex subunit 7 homolog	23.728	5.67	7.5460507	10.294118	2	2	2	101.8	98.2	0.965	2.34E-01
Q6PI48	Aspartate--tRNA ligase, mitochondrial	73.516	8.02	33.138461	12.403101	7	7	7	101.8	98.2	0.965	1.25E-01
Q6ZYL4	General transcription factor IIH subunit 5	8.048	4.65	10.941579	36.619718	2	2	2	101.8	98.2	0.965	
Q7RTP6	Protein-methionine sulfoxide oxidase MICAL3	224.156	5.55	22.806201	3.2967033	6	7	6	101.8	98.2	0.965	3.08E-02
Q8N3X1	Formin-binding protein 4	110.198	4.74	16.031312	4.2281219	3	3	3	101.8	98.2	0.965	2.10E-01
Q8N5N7	39S ribosomal protein L50, mitochondrial	18.313	7.88	25.595427	37.341772	4	4	4	101.8	98.2	0.965	7.26E-01
Q8WVM0	Dimethyladenosine transferase 1, mitochondrial	39.518	9.26	8.1019333	4.0462428	1	1	1	101.8	98.2	0.965	
Q8WXI9	Transcriptional repressor p66-beta	65.22	9.7	38.156083	14.333895	6	6	6	101.8	98.2	0.965	3.85E-01
Q96AQ6	Pre-B-cell leukemia transcription factor-interacting protein 1	80.594	5.33	37.234505	9.7127223	5	7	5	101.8	98.2	0.965	9.12E-02
Q96BH1	E3 ubiquitin-protein ligase RNF25	51.187	6.54	16.992943	4.5751634	2	3	2	101.8	98.2	0.965	5.53E-01
Q96FZ2	Embryonic stem cell-specific 5-hydroxymethylcytosine-binding protein	40.549	8.15	53.682156	20.056497	6	10	6	101.8	98.2	0.965	4.77E-01
Q96IV0	Peptide-N(4)-(N-acetyl-beta-glucosaminyl)asparagine amidase	74.343	6.89	7.5233677	3.2110092	2	2	2	101.8	98.2	0.965	
Q99829	Copine-1	59.022	5.83	72.869638	21.22905	12	20	12	101.8	98.2	0.965	5.87E-01
Q9BQB6	Vitamin K epoxide reductase complex subunit 1	18.223	9.36	9.9125735	7.9754601	1	2	1	101.8	98.2	0.965	
Q9C0E8	Protein lunapark	47.71	5.11	3.9965395	1.8691589	1	1	1	101.8	98.2	0.965	
Q9GZP4	PITH domain-containing protein 1	24.163	5.74	23.013557	21.327014	3	5	3	101.8	98.2	0.965	1.00E+00
Q9HAV0	Guanine nucleotide-binding protein subunit beta-4	37.543	6	68.209569	22.647059	8	21	5	101.8	98.2	0.965	6.95E-01
Q9HCU5	Prolactin regulatory element-binding protein	45.44	7.88	32.894693	11.7506	4	5	4	101.8	98.2	0.965	1.54E-03
Q9NP81	Serine--tRNA ligase, mitochondrial	58.246	8.13	47.061797	17.181467	6	7	6	101.8	98.2	0.965	3.34E-02
Q9NPJ3	Acyl-coenzyme A thioesterase 13	14.951	9.14	23.881694	25	3	4	3	101.8	98.2	0.965	
Q9NR45	Sialic acid synthase	40.281	6.74	113.55421	38.440111	11	24	11	101.8	98.2	0.965	1.04E-01
Q9NWB6	Arginine and glutamate-rich protein 1	33.197	10.35	15.367588	11.355311	4	4	4	101.8	98.2	0.965	
Q9UNH6	Sorting nexin-7	45.274	5.11	16.345474	6.4599483	3	4	3	101.8	98.2	0.965	4.60E-02
Q9Y6C9	Mitochondrial carrier homolog 2	33.309	7.97	16.551065	13.531353	3	3	3	101.8	98.2	0.965	5.08E-01
O15230	Laminin subunit alpha-5	399.479	7.02	34.129496	1.8673884	6	6	6	101.7	98.3	0.967	3.61E-01
O75475	PC4 and SFRS1-interacting protein	60.067	9.13	77.17589	17.735849	10	19	9	101.7	98.3	0.967	2.58E-01
O95865	N(G),N(G)-dimethylarginine dimethylaminohydrolase 2	29.625	6.01	68.516929	41.052632	9	10	8	101.7	98.3	0.967	1.49E-03
P00367	Glutamate dehydrogenase 1, mitochondrial	61.359	7.8	216.09942	33.691756	17	38	17	101.7	98.3	0.967	4.52E-02
P07384	Calpain-1 catalytic subunit	81.838	5.67	70.243001	13.72549	9	14	9	101.7	98.3	0.967	2.15E-01
P12004	Proliferating cell nuclear antigen	28.75	4.69	143.44821	44.061303	11	33	11	101.7	98.3	0.967	2.60E-03
P14625	Endoplasmin	92.411	4.84	431.77196	41.344956	37	147	35	101.7	98.3	0.967	9.72E-03
P20908	Collagen alpha-1(V) chain	183.447	5.06	25.873268	3.3188248	5	6	5	101.7	98.3	0.967	2.34E-01

P30520	Adenylosuccinate synthetase isozyme 2	50.066	6.55	106.71308	30.263158	11	23	10	101.7	98.3	0.967	5.96E-02
P32119	Peroxiredoxin-2	21.878	5.97	79.543212	36.363636	9	25	8	101.7	98.3	0.967	4.00E-01
P35579	Myosin-9	226.392	5.6	1326.6169	49.183673	102	284	94	101.7	98.3	0.967	4.67E-08
P43121	Cell surface glycoprotein MUC18	71.563	5.76	44.730494	14.705882	8	8	8	101.7	98.3	0.967	1.73E-02
P46778	60S ribosomal protein L21	18.553	10.49	39.569281	36.25	8	24	8	101.7	98.3	0.967	2.60E-02
P78417	Glutathione S-transferase omega-1	27.548	6.6	72.315037	26.556017	8	24	8	101.7	98.3	0.967	7.48E-01
P78527	DNA-dependent protein kinase catalytic subunit	468.788	7.12	888.7601	25.750969	99	177	99	101.7	98.3	0.967	6.78E-03
Q00653	Nuclear factor NF-kappa-B p100 subunit	96.689	6.25	57.670757	10.777778	8	10	8	101.7	98.3	0.967	5.30E-01
Q10567	AP-1 complex subunit beta-1	104.57	5.06	138.58159	19.072708	16	29	8	101.7	98.3	0.967	2.00E-03
Q13490	Baculoviral IAP repeat-containing protein 2	69.854	6.7	6.7401673	1.4563107	1	1	1	101.7	98.3	0.967	
Q14161	ARF GTPase-activating protein GIT2	84.49	7.23	22.723369	6.1923584	4	6	3	101.7	98.3	0.967	4.74E-01
Q14232	Translation initiation factor eIF-2B subunit alpha	33.691	7.33	49.929214	25.245902	9	12	9	101.7	98.3	0.967	1.25E-01
Q15058	Kinesin-like protein KIF14	186.375	7.91	20.774622	2.8519417	5	5	5	101.7	98.3	0.967	3.51E-01
Q15126	Phosphomevalonate kinase	21.981	5.73	26.600032	22.395833	4	5	4	101.7	98.3	0.967	5.00E-01
Q15257	Serine/threonine-protein phosphatase 2A activator	40.641	5.94	49.484842	24.860335	8	10	8	101.7	98.3	0.967	1.93E-01
Q15435	Protein phosphatase 1 regulatory subunit 7	41.539	4.91	34.533893	13.611111	5	7	5	101.7	98.3	0.967	2.93E-01
Q15477	Helicase SKI2W	137.668	6.06	96.862061	16.131621	14	18	14	101.7	98.3	0.967	1.06E-02
Q15596	Nuclear receptor coactivator 2	159.056	6.64	10.696156	1.2295082	1	1	1	101.7	98.3	0.967	
Q15648	Mediator of RNA polymerase II transcription subunit 1	168.373	8.73	5.1467278	0.56926	1	1	1	101.7	98.3	0.967	
Q32MZ4	Leucine-rich repeat flightless-interacting protein 1	89.199	4.65	123.37621	16.584158	11	22	10	101.7	98.3	0.967	6.89E-02
Q6P1N9	Putative deoxyribonuclease TATDN1	33.58	6.96	47.800924	21.212121	6	9	6	101.7	98.3	0.967	7.44E-01
Q6YN16	Hydroxysteroid dehydrogenase-like protein 2	45.366	7.99	106.98179	32.057416	11	20	11	101.7	98.3	0.967	7.76E-02
Q7L2H7	Eukaryotic translation initiation factor 3 subunit M	42.476	5.63	82.558158	25.13369	10	18	10	101.7	98.3	0.967	7.18E-01
Q8NBS9	Thioredoxin domain-containing protein 5	47.599	5.97	98.402326	27.546296	11	23	11	101.7	98.3	0.967	1.47E-01
Q8NHH9	Atlastin-2	66.187	5.48	33.525957	6.3464837	3	6	3	101.7	98.3	0.967	7.76E-01
Q96CU9	FAD-dependent oxidoreductase domain-containing protein 1	53.778	7.78	22.510023	9.8765432	5	5	5	101.7	98.3	0.967	4.93E-02
Q96DE0	U8 snoRNA-decapping enzyme	21.26	6.89	46.467925	35.384615	6	8	5	101.7	98.3	0.967	8.38E-01
Q99442	Translocation protein SEC62	45.833	7.12	18.085556	12.030075	5	5	5	101.7	98.3	0.967	2.13E-03
Q9BUE0	Mediator of RNA polymerase II transcription subunit 18	23.647	6.54	6.7066926	8.6538462	2	2	2	101.7	98.3	0.967	
Q9HAD4	WD repeat-containing protein 41	51.695	5.44	3.4506288	1.9607843	1	1	1	101.7	98.3	0.967	
Q9NRG0	Chromatin accessibility complex protein 1	14.701	5.1	18.209692	13.740458	1	3	1	101.7	98.3	0.967	
Q9NRW7	Vacuolar protein sorting-associated protein 45	65.036	8.24	16.772036	6.4912281	4	5	4	101.7	98.3	0.967	1.36E-01
Q9UKS6	Protein kinase C and casein kinase substrate in neurons protein 3	48.457	6.18	73.462126	27.122642	10	16	10	101.7	98.3	0.967	2.21E-01
Q9ULH0	Kinase D-interacting substrate of 220 kDa	196.419	6.62	21.571535	1.2987013	2	4	2	101.7	98.3	0.967	4.68E-02
Q9Y5K6	CD2-associated protein	71.407	6.4	5.325966	2.0344288	1	1	1	101.7	98.3	0.967	
O15403	Monocarboxylate transporter 7	57.356	7.81	22.461218	8.7954111	3	4	3	101.6	98.4	0.969	7.84E-02
O60502	Protein O-GlcNAcase	102.849	4.91	64.230606	10.262009	8	10	8	101.6	98.4	0.969	2.44E-02
O60563	Cyclin-T1	80.634	8.78	31.179268	5.5096419	4	6	4	101.6	98.4	0.969	9.06E-01
O76041	Nebulette	116.38	7.99	14.762028	2.3668639	2	3	2	101.6	98.4	0.969	9.52E-01
O95379	Tumor necrosis factor alpha-induced protein 8	22.989	7.93	4.6138579	4.040404	1	1	1	101.6	98.4	0.969	
O95400	CD2 antigen cytoplasmic tail-binding protein 2	37.623	4.61	37.619715	19.941349	5	6	5	101.6	98.4	0.969	7.35E-01
P08621	U1 small nuclear ribonucleoprotein 70 kDa	51.526	9.94	61.951404	20.594966	10	20	10	101.6	98.4	0.969	7.99E-01
P14927	Cytochrome b-c1 complex subunit 7	13.522	8.78	24.416001	23.423423	3	5	3	101.6	98.4	0.969	3.52E-02
P16298	Serine/threonine-protein phosphatase 2B catalytic subunit beta isoform	58.987	5.91	38.209484	14.885496	7	10	2	101.6	98.4	0.969	
P21266	Glutathione S-transferase Mu 3	26.542	5.54	211.94661	68.444444	20	61	19	101.6	98.4	0.969	8.17E-01
P25787	Proteasome subunit alpha type-2	25.882	7.43	53.566232	31.623932	4	7	4	101.6	98.4	0.969	4.28E-01
P36404	ADP-ribosylation factor-like protein 2	20.865	6.34	44.060262	23.913043	4	8	4	101.6	98.4	0.969	1.22E-01

P36543	V-type proton ATPase subunit E 1	26.129	8	71.094454	32.743363	8	12	8	101.6	98.4	0.969	2.16E-01
P36776	Lon protease homolog, mitochondrial	106.422	6.39	165.30759	22.210636	18	26	18	101.6	98.4	0.969	5.58E-02
P38606	V-type proton ATPase catalytic subunit A	68.26	5.52	130.84887	28.849271	17	31	17	101.6	98.4	0.969	2.04E-01
P40939	Trifunctional enzyme subunit alpha, mitochondrial	82.947	9.04	296.01001	38.138925	26	64	26	101.6	98.4	0.969	3.38E-01
P51157	Ras-related protein Rab-28	24.826	5.97	2.5207127	3.1674208	1	1	1	101.6	98.4	0.969	
P54619	5'-AMP-activated protein kinase subunit gamma-1	37.556	6.92	38.065904	15.10574	5	7	5	101.6	98.4	0.969	9.66E-02
P55060	Exportin-2	110.346	5.77	295.91791	25.23172	27	72	27	101.6	98.4	0.969	2.87E-01
P57737	Coronin-7	100.542	5.8	6.4032831	1.9459459	2	2	2	101.6	98.4	0.969	4.07E-01
P60953	Cell division control protein 42 homolog	21.245	6.55	75.083187	31.413613	5	31	4	101.6	98.4	0.969	5.67E-01
P61457	Pterin-4-alpha-carbinolamine dehydratase	11.992	6.8	24.085053	29.807692	4	7	4	101.6	98.4	0.969	1.40E-01
Q05209	Tyrosine-protein phosphatase non-receptor type 12	88.051	5.62	26.35866	7.5641026	5	5	5	101.6	98.4	0.969	2.17E-01
Q10471	Polypeptide N-acetylgalactosaminyltransferase 2	64.691	8.35	100.44496	23.642732	14	22	14	101.6	98.4	0.969	6.33E-02
Q12802	A-kinase anchor protein 13	307.359	5.24	52.187934	3.163882	6	8	6	101.6	98.4	0.969	2.62E-01
Q14573	Inositol 1,4,5-trisphosphate receptor type 3	303.912	6.48	126.568	8.4612505	22	25	17	101.6	98.4	0.969	1.41E-01
Q14919	Dr1-associated corepressor	22.336	5.17	33.757033	29.756098	5	7	5	101.6	98.4	0.969	3.53E-01
Q15363	Transmembrane emp24 domain-containing protein 2	22.746	5.17	16.574034	4.4776119	1	9	1	101.6	98.4	0.969	
Q15642	Cdc42-interacting protein 4	68.31	5.73	60.092697	19.633943	10	12	10	101.6	98.4	0.969	8.40E-01
Q32P41	tRNA (guanine(37)-N1)-methyltransferase	58.209	8.62	15.688894	8.8408644	5	9	4	101.6	98.4	0.969	8.16E-01
Q4G0N4	NAD kinase 2, mitochondrial	49.402	8.18	49.2578	20.81448	9	12	9	101.6	98.4	0.969	5.59E-01
Q5VT66	Mitochondrial amidoxime-reducing component 1	37.476	8.88	46.288245	20.178042	7	11	6	101.6	98.4	0.969	1.43E-01
Q6PD74	Alpha- and gamma-adaptin-binding protein p34	34.572	4.64	6.5400227	5.0793651	2	2	2	101.6	98.4	0.969	
Q8NEM7	Transcription factor SPT20 homolog	85.735	8.48	5.650334	1.9255456	1	1	1	101.6	98.4	0.969	
Q8NF37	Lysophosphatidylcholine acyltransferase 1	59.113	6.02	67.88993	12.359551	6	11	6	101.6	98.4	0.969	1.98E-01
Q8TCS8	Polyribonucleotide nucleotidyltransferase 1, mitochondrial	85.897	7.77	82.076591	13.793103	10	15	10	101.6	98.4	0.969	9.72E-01
Q8WVJ2	NudC domain-containing protein 2	17.665	5.07	15.486995	29.299363	3	3	3	101.6	98.4	0.969	8.45E-01
Q96A33	Coiled-coil domain-containing protein 47	55.838	4.87	52.309422	17.391304	8	11	8	101.6	98.4	0.969	2.34E-01
Q96I99	Succinyl-CoA ligase [GDP-forming] subunit beta, mitochondrial	46.481	6.39	141.97061	29.861111	12	25	12	101.6	98.4	0.969	1.30E-02
Q96MW5	Conserved oligomeric Golgi complex subunit 8	68.38	5.2	5.4103168	3.2679739	2	2	2	101.6	98.4	0.969	
Q96RS6	NudC domain-containing protein 1	66.713	5.11	30.57554	11.149228	6	6	6	101.6	98.4	0.969	8.05E-01
Q9BRT9	DNA replication complex GINS protein SLD5	26.03	4.98	21.642816	18.38565	3	3	3	101.6	98.4	0.969	7.62E-01
Q9BY89	Uncharacterized protein KIAA1671	196.59	8.47	39.732434	4.5404208	6	7	6	101.6	98.4	0.969	5.14E-01
Q9H4P4	E3 ubiquitin-protein ligase NRDP1	35.882	6.14	7.0732163	4.7318612	2	2	1	101.6	98.4	0.969	
Q9NRY2	SOSS complex subunit C	11.418	9.25	8.6561977	12.5	1	1	1	101.6	98.4	0.969	
Q9NW08	DNA-directed RNA polymerase III subunit RPC2	127.702	8.5	27.462013	3.8834951	4	4	4	101.6	98.4	0.969	6.93E-01
Q9UIJ7	GTP:AMP phosphotransferase AK3, mitochondrial	25.55	9.16	43.144363	24.229075	5	8	5	101.6	98.4	0.969	1.45E-02
Q9UJZ1	Stomatin-like protein 2, mitochondrial	38.51	7.39	62.67123	28.932584	7	14	7	101.6	98.4	0.969	4.62E-03
Q9UKY7	Protein CDV3 homolog	27.318	6.4	69.263059	39.922481	6	11	6	101.6	98.4	0.969	8.42E-01
Q9Y295	Developmentally-regulated GTP-binding protein 1	40.517	8.9	103.28348	33.787466	10	18	10	101.6	98.4	0.969	2.89E-01
A3KMH1	von Willebrand factor A domain-containing protein 8	214.689	7.4	33.42937	4.5144357	7	7	7	101.5	98.5	0.97	1.11E-01
O00186	Syntaxin-binding protein 3	67.721	7.8	46.649073	12.331081	7	9	7	101.5	98.5	0.97	3.45E-02
O15126	Secretory carrier-associated membrane protein 1	37.896	7.42	20.761011	5.9171598	2	3	2	101.5	98.5	0.97	8.52E-01
O75122	CLIP-associating protein 2	141.046	8.47	39.252324	5.4095827	6	7	5	101.5	98.5	0.97	6.37E-01
O94901	SUN domain-containing protein 1	90.007	7.08	113.23051	27.586207	15	18	15	101.5	98.5	0.97	2.52E-01
O96006	Zinc finger BED domain-containing protein 1	78.106	6.1	33.480598	9.5100865	5	5	5	101.5	98.5	0.97	1.63E-01
P04844	olichyl-diphosphooligosaccharide--protein glycosyltransferase subunit	69.241	5.69	100.709	21.394612	10	25	10	101.5	98.5	0.97	3.88E-01
P09525	Annexin A4	35.86	6.13	107.92218	45.454545	14	23	13	101.5	98.5	0.97	7.35E-01
P09543	2',3'-cyclic-nucleotide 3'-phosphodiesterase	47.549	9.07	61.322841	25.653207	10	12	10	101.5	98.5	0.97	1.53E-03

P13797	Plastin-3	70.766	5.6	309.31822	43.968254	28	82	28	101.5	98.5	0.97	4.00E-03
P14174	Macrophage migration inhibitory factor	12.468	7.88	28.471395	23.478261	3	12	3	101.5	98.5	0.97	1.43E-01
P14209	CD99 antigen	18.836	4.75	19.527496	15.135135	3	5	3	101.5	98.5	0.97	1.43E-01
P15531	Nucleoside diphosphate kinase A	17.138	6.19	107.06142	48.684211	10	41	4	101.5	98.5	0.97	6.88E-02
P43355	Melanoma-associated antigen 1	34.32	4.86	27.499537	16.504854	5	7	5	101.5	98.5	0.97	2.59E-01
P49848	Transcription initiation factor TFIID subunit 6	72.623	8.6	19.529369	5.760709	3	3	3	101.5	98.5	0.97	8.86E-01
P50213	Isocitrate dehydrogenase [NAD] subunit alpha, mitochondrial	39.566	6.92	60.198698	28.961749	10	13	10	101.5	98.5	0.97	4.92E-01
P52888	Thimet oligopeptidase	78.789	6.05	74.639414	19.738752	11	15	11	101.5	98.5	0.97	6.03E-02
Q00169	Phosphatidylinositol transfer protein alpha isoform	31.786	6.55	69.095974	42.592593	10	12	7	101.5	98.5	0.97	4.12E-01
Q13464	Rho-associated protein kinase 1	158.076	5.9	69.134894	8.788774	11	12	7	101.5	98.5	0.97	3.54E-01
Q15084	Protein disulfide-isomerase A6	48.091	5.08	149.98356	34.090909	13	37	13	101.5	98.5	0.97	3.18E-02
Q15165	Serum paraoxonase/arylesterase 2	39.372	5.6	20.483465	12.146893	4	7	4	101.5	98.5	0.97	4.28E-01
Q16851	UTP--glucose-1-phosphate uridylyltransferase	56.905	8.15	116.26691	33.464567	16	26	16	101.5	98.5	0.97	1.38E-01
Q32P28	Prolyl 3-hydroxylase 1	83.341	5.14	61.311625	12.36413	9	12	9	101.5	98.5	0.97	4.92E-04
Q3ZCQ8	Mitochondrial import inner membrane translocase subunit TIM50	39.622	8.37	49.136411	21.246459	6	8	6	101.5	98.5	0.97	5.64E-01
Q4KWH8	1-phosphatidylinositol 4,5-bisphosphate phosphodiesterase eta-1	189.104	7.74	6.8207529	0.472534	1	4	1	101.5	98.5	0.97	
Q567U6	Coiled-coil domain-containing protein 93	73.152	8.15	26.489156	7.7654517	5	6	5	101.5	98.5	0.97	4.10E-01
Q5T5P2	Sickle tail protein homolog	213.983	7.06	2.9465374	0.514668	1	1	1	101.5	98.5	0.97	
Q5T5U3	Rho GTPase-activating protein 21	217.198	7.8	4.851089	0.5109862	1	1	1	101.5	98.5	0.97	
Q70E73	Ras-associated and pleckstrin homology domains-containing protein 1	135.171	8.85	18.727853	2.4	3	4	3	101.5	98.5	0.97	2.63E-01
Q8N7H5	RNA polymerase II-associated factor 1 homolog	59.939	4.63	43.83478	8.2862524	3	5	3	101.5	98.5	0.97	2.11E-01
Q8NEU8	DCC-interacting protein 13-beta	74.446	4.94	17.081567	4.3674699	2	2	2	101.5	98.5	0.97	3.38E-02
Q8NHV4	Protein NEDD1	71.921	7.97	6.8643311	2.4242424	2	2	2	101.5	98.5	0.97	
Q8WUA4	General transcription factor 3C polypeptide 2	100.616	7.31	32.016171	5.8177827	5	7	4	101.5	98.5	0.97	3.45E-01
Q96EY7	Pentatricopeptide repeat domain-containing protein 3, mitochondrial	78.5	6.42	43.016374	12.191582	7	8	7	101.5	98.5	0.97	7.29E-03
Q96I15	Selenocysteine lyase	48.119	7.12	5.7607005	3.3707865	1	1	1	101.5	98.5	0.97	
Q96I59	Probable asparagine--tRNA ligase, mitochondrial	54.056	7.24	12.628331	6.0796646	2	2	2	101.5	98.5	0.97	3.64E-01
Q96IG2	F-box/LRR-repeat protein 20	48.392	7.49	8.9450041	2.5229358	1	1	1	101.5	98.5	0.97	
Q96IJ6	Mannose-1-phosphate guanyltransferase alpha	46.262	7.21	24.160518	13.571429	5	5	5	101.5	98.5	0.97	
Q96QR8	Transcriptional activator protein Pur-beta	33.22	5.43	6.3185778	4.4871795	1	1	1	101.5	98.5	0.97	
Q96S66	Chloride channel CLIC-like protein 1	61.983	5.55	7.8074189	3.8112523	2	2	2	101.5	98.5	0.97	9.80E-01
Q9C0H2	Protein tweety homolog 3	57.508	5.39	4.6673154	3.4416826	2	2	2	101.5	98.5	0.97	4.29E-01
Q9H0J9	Poly [ADP-ribose] polymerase 12	79.013	8.51	4.987774	3.2810271	2	2	2	101.5	98.5	0.97	
Q9H2H8	Peptidyl-prolyl cis-trans isomerase-like 3	18.143	6.79	20.893983	27.329193	4	5	4	101.5	98.5	0.97	2.03E-01
Q9H4A3	Serine/threonine-protein kinase WNK1	250.64	6.34	73.965685	5.7094878	11	15	11	101.5	98.5	0.97	8.63E-03
Q9H4I3	TraB domain-containing protein	42.294	8	6.2239529	3.1914894	1	1	1	101.5	98.5	0.97	
Q9HB40	Retinoid-inducible serine carboxypeptidase	50.798	5.81	25.031346	7.9646018	3	4	3	101.5	98.5	0.97	5.20E-04
Q9NVS9	Pyridoxine-5'-phosphate oxidase	29.969	7.06	11.636897	9.5785441	2	2	2	101.5	98.5	0.97	
Q9NVV4	Poly(A) RNA polymerase, mitochondrial	66.13	9.04	17.995591	6.7010309	3	4	3	101.5	98.5	0.97	6.78E-01
Q9UBI6	Guanine nucleotide-binding protein G(I)/G(S)/G(O) subunit gamma-12	8.001	8.97	45.221016	59.722222	4	10	4	101.5	98.5	0.97	8.28E-02
Q9UK41	Vacuolar protein sorting-associated protein 28 homolog	25.409	5.54	20.871816	23.529412	5	5	5	101.5	98.5	0.97	1.67E-02
Q9UK61	Protein FAM208A	188.914	5.8	5.8198627	1.257485	2	2	2	101.5	98.5	0.97	
Q9ULP9	TBC1 domain family member 24	62.879	7.36	8.3847653	3.7567084	2	2	2	101.5	98.5	0.97	6.52E-01
Q9Y2Q5	Ragulator complex protein LAMTOR2	13.499	5.4	20.873869	29.6	3	4	3	101.5	98.5	0.97	4.07E-01
Q9Y3E0	Vesicle transport protein GOT1B	15.415	10.36	5.4161582	5.7971014	1	2	1	101.5	98.5	0.97	
O15226	NF-kappa-B-repressing factor	77.624	8.79	46.246838	16.376812	11	12	11	101.4	98.6	0.972	2.05E-01
O60244	Mediator of RNA polymerase II transcription subunit 14	160.504	8.73	7.9277389	1.3755158	2	2	2	101.4	98.6	0.972	

O75695	Protein XRP2	39.615	5.12	8.8222944	4.5714286	2	2	2	101.4	98.6	0.972	
O95674	Phosphatidate cytidylyltransferase 2	51.384	7.09	7.6003552	5.6179775	2	2	1	101.4	98.6	0.972	
P00966	Argininosuccinate synthase	46.501	8.02	234.31379	52.427184	24	110	24	101.4	98.6	0.972	2.68E-01
P04075	Fructose-bisphosphate aldolase A	39.395	8.09	319.22561	68.131868	26	114	21	101.4	98.6	0.972	3.77E-03
P06744	Glucose-6-phosphate isomerase	63.107	8.32	187.48706	33.870968	20	52	20	101.4	98.6	0.972	8.42E-01
P09211	Glutathione S-transferase P	23.341	5.64	172.38142	46.666667	9	75	9	101.4	98.6	0.972	5.82E-01
P11441	Ubiquitin-like protein 4A	17.766	8.66	33.505075	31.210191	4	7	4	101.4	98.6	0.972	4.16E-01
P17612	cAMP-dependent protein kinase catalytic subunit alpha	40.564	8.79	27.069243	12.820513	5	6	2	101.4	98.6	0.972	
P28838	Cytosol aminopeptidase	56.131	7.93	167.07847	38.728324	14	24	14	101.4	98.6	0.972	1.29E-03
P37802	Transgelin-2	22.377	8.25	262.72608	74.874372	15	75	15	101.4	98.6	0.972	2.29E-01
P46459	Vesicle-fusing ATPase	82.542	6.95	165.08027	28.360215	19	35	18	101.4	98.6	0.972	6.41E-01
P53990	IST1 homolog	39.725	5.35	39.866575	21.703297	6	7	6	101.4	98.6	0.972	1.62E-02
P61086	Ubiquitin-conjugating enzyme E2 K	22.393	5.44	48.323495	32	7	9	7	101.4	98.6	0.972	1.28E-02
P61962	DDB1- and CUL4-associated factor 7	38.901	5.52	25.933855	16.081871	4	4	4	101.4	98.6	0.972	7.31E-02
P62244	40S ribosomal protein S15a	14.83	10.13	50.402653	33.076923	5	16	5	101.4	98.6	0.972	3.89E-01
P62879	Guanine nucleotide-binding protein G(I)/G(S)/G(T) subunit beta-2	37.307	6	91.904278	25.294118	7	23	3	101.4	98.6	0.972	7.56E-02
P78549	Endonuclease III-like protein 1	34.368	9.67	12.98579	8.3333333	2	2	2	101.4	98.6	0.972	1.68E-01
P80217	Interferon-induced 35 kDa protein	31.527	6.09	13.906067	6.6433566	2	3	2	101.4	98.6	0.972	2.17E-01
Q13409	Cytoplasmic dynein 1 intermediate chain 2	71.412	5.2	54.719	12.068966	7	13	7	101.4	98.6	0.972	5.04E-02
Q13418	Integrin-linked protein kinase	51.386	8.07	62.391573	23.893805	11	18	11	101.4	98.6	0.972	9.12E-01
Q13868	Exosome complex component RRP4	32.768	7.5	29.259799	19.112628	4	5	4	101.4	98.6	0.972	1.26E-01
Q14186	Transcription factor Dp-1	45.043	6.05	6.3099812	2.6829268	1	1	1	101.4	98.6	0.972	
Q14692	Ribosome biogenesis protein BMS1 homolog	145.716	6.44	17.193536	2.6521061	3	3	3	101.4	98.6	0.972	4.13E-01
Q15059	Bromodomain-containing protein 3	79.492	9.36	52.178504	14.46281	9	11	5	101.4	98.6	0.972	1.58E-01
Q15172	:/threonine-protein phosphatase 2A 56 kDa regulatory subunit alpha is	56.158	6.71	24.535851	7.2016461	3	4	3	101.4	98.6	0.972	6.26E-02
Q15459	Splicing factor 3A subunit 1	88.831	5.22	212.82519	28.87768	21	39	21	101.4	98.6	0.972	1.72E-01
Q3LXA3	tional ATP-dependent dihydroxyacetone kinase/FAD-AMP lyase (cyc	58.91	7.49	99.689581	28.173913	13	23	13	101.4	98.6	0.972	1.74E-02
Q53H12	Acylglycerol kinase, mitochondrial	47.107	8.09	42.587494	17.061611	6	7	6	101.4	98.6	0.972	4.78E-01
Q7Z589	Protein EMSY	141.381	9.33	3.982289	1.2859304	2	2	1	101.4	98.6	0.972	
Q86VS8	Protein Hook homolog 3	83.074	5.17	32.935192	4.8746518	3	5	3	101.4	98.6	0.972	1.02E-01
Q8IXQ5	Kelch-like protein 7	65.949	6.48	2.5128616	1.8771331	1	1	1	101.4	98.6	0.972	
Q8N6R0	Methyltransferase-like protein 13	78.718	6.73	29.185021	6.1516452	4	5	4	101.4	98.6	0.972	1.94E-01
Q8NI36	WD repeat-containing protein 36	105.255	7.53	31.967189	7.0452156	6	7	6	101.4	98.6	0.972	6.06E-01
Q8WUQ7	Cactin	88.648	9.14	16.835507	4.2216359	2	2	2	101.4	98.6	0.972	8.90E-01
Q92576	PHD finger protein 3	229.339	6.96	30.531168	2.8935753	5	5	5	101.4	98.6	0.972	1.56E-02
Q92747	Actin-related protein 2/3 complex subunit 1A	41.543	8.18	10.444682	5.9459459	2	2	2	101.4	98.6	0.972	8.29E-01
Q92759	General transcription factor IIH subunit 4	52.153	9.04	6.2548471	3.4632035	1	1	1	101.4	98.6	0.972	
Q969G3	ted matrix-associated actin-dependent regulator of chromatin subfamil	46.621	4.88	49.269755	11.435523	4	8	3	101.4	98.6	0.972	9.78E-01
Q96C36	Pyrroline-5-carboxylate reductase 2	33.616	7.77	59.341197	23.125	7	11	7	101.4	98.6	0.972	3.36E-01
Q96H20	Vacuolar-sorting protein SNF8	28.846	6.65	3.6256183	3.1007752	1	1	1	101.4	98.6	0.972	
Q99496	E3 ubiquitin-protein ligase RING2	37.632	6.84	18.040022	11.011905	3	4	2	101.4	98.6	0.972	7.51E-01
Q9BZE2	tRNA pseudouridine(38/39) synthase	55.612	7.49	7.6154002	4.3659044	2	2	2	101.4	98.6	0.972	
Q9C0B1	Alpha-ketoglutarate-dependent dioxygenase FTO	58.245	5.22	102.19254	20.990099	10	16	10	101.4	98.6	0.972	3.71E-02
Q9HBM1	Kinetochore protein Spc25	26.137	8	34.465862	15.625	3	5	3	101.4	98.6	0.972	
Q9NQ48	Leucine zipper transcription factor-like protein 1	34.571	5.36	101.16467	31.103679	8	14	8	101.4	98.6	0.972	7.60E-02
Q9NR28	Diablo homolog, mitochondrial	27.114	5.9	15.068987	11.715481	3	4	3	101.4	98.6	0.972	5.70E-02
Q9NRF9	DNA polymerase epsilon subunit 3	16.849	4.74	16.340857	24.489796	3	3	3	101.4	98.6	0.972	

Q9NVZ3	Adaptin ear-binding coat-associated protein 2	28.321	8.38	10.553042	6.4638783	2	3	2	101.4	98.6	0.972	2.62E-01
Q9NZN4	EH domain-containing protein 2	61.123	6.46	58.815502	16.022099	9	11	8	101.4	98.6	0.972	7.89E-01
Q9UBB4	Ataxin-10	53.455	5.25	157.87788	39.789474	18	32	17	101.4	98.6	0.972	4.38E-01
Q9UBS8	E3 ubiquitin-protein ligase RNF14	53.802	4.75	14.178257	4.8523207	2	3	2	101.4	98.6	0.972	3.38E-02
Q9ULU4	Protein kinase C-binding protein 1	131.61	7.2	27.149346	4.9747049	5	7	5	101.4	98.6	0.972	3.94E-01
Q9ULV4	Coronin-1C	53.215	7.08	102.75314	23.628692	13	24	13	101.4	98.6	0.972	3.08E-01
Q9UNE7	E3 ubiquitin-protein ligase CHIP	34.834	5.87	53.699134	33.663366	12	19	11	101.4	98.6	0.972	7.95E-02
Q9UPW5	Cytosolic carboxypeptidase 1	138.361	6.15	8.4709704	1.7128874	2	2	2	101.4	98.6	0.972	
O43678	NADH dehydrogenase [ubiquinone] 1 alpha subcomplex subunit 2	10.915	9.57	32.533562	55.555556	5	7	5	101.3	98.7	0.974	6.37E-01
O60664	Perilipin-3	47.046	5.44	178.93716	34.792627	13	32	13	101.3	98.7	0.974	9.17E-01
O75431	Metaxin-2	29.744	6.29	8.6332608	4.1825095	1	2	1	101.3	98.7	0.974	
O95292	Vesicle-associated membrane protein-associated protein B/C	27.211	7.3	48.382636	29.218107	8	12	7	101.3	98.7	0.974	4.98E-01
P04004	Vitronectin	54.271	5.8	16.125649	3.1380753	1	2	1	101.3	98.7	0.974	
P04080	Cystatin-B	11.133	7.56	44.068541	40.816327	4	14	4	101.3	98.7	0.974	1.30E-03
P06454	Prothymosin alpha	12.196	3.78	59.554583	23.423423	6	44	6	101.3	98.7	0.974	2.74E-01
P11279	Lysosome-associated membrane glycoprotein 1	44.854	8.75	53.328785	14.148681	6	12	6	101.3	98.7	0.974	5.66E-01
P13798	Acylamino-acid-releasing enzyme	81.173	5.48	107.08405	22.540984	14	22	14	101.3	98.7	0.974	5.37E-01
P14868	Aspartate--tRNA ligase, cytoplasmic	57.1	6.55	137.0552	45.708583	21	34	21	101.3	98.7	0.974	1.31E-01
P25098	Beta-adrenergic receptor kinase 1	79.522	7.28	3.4414114	1.161103	1	1	1	101.3	98.7	0.974	
P25325	3-mercaptopyruvate sulfurtransferase	33.158	6.6	21.173197	21.212121	4	4	4	101.3	98.7	0.974	5.70E-01
P27361	Mitogen-activated protein kinase 3	43.108	6.74	58.851507	26.385224	9	11	5	101.3	98.7	0.974	8.57E-01
P31040	ıccinate dehydrogenase [ubiquinone] flavoprotein subunit, mitochondr	72.645	7.39	174.94378	33.433735	16	40	16	101.3	98.7	0.974	2.04E-01
P42785	Lysosomal Pro-X carboxypeptidase	55.764	7.21	14.49972	6.8548387	3	3	3	101.3	98.7	0.974	4.39E-01
P61586	Transforming protein RhoA	21.754	6.1	86.374797	41.450777	9	20	3	101.3	98.7	0.974	1.75E-01
P80723	Brain acid soluble protein 1	22.68	4.63	229.18421	86.784141	19	81	19	101.3	98.7	0.974	1.86E-01
P82673	28S ribosomal protein S35, mitochondrial	36.821	8.24	21.396959	12.074303	3	4	3	101.3	98.7	0.974	2.25E-02
Q13232	Nucleoside diphosphate kinase 3	19.003	7.84	12.49622	10.059172	2	3	2	101.3	98.7	0.974	4.68E-01
Q13439	Golgin subfamily A member 4	260.98	5.39	110.88107	9.6412556	20	22	19	101.3	98.7	0.974	1.05E-01
Q13617	Cullin-2	86.927	6.92	51.130383	12.080537	8	11	8	101.3	98.7	0.974	2.41E-02
Q14643	Inositol 1,4,5-trisphosphate receptor type 1	313.729	6.04	151.22604	9.0282814	26	33	18	101.3	98.7	0.974	1.60E-01
Q15019	Septin-2	41.461	6.6	141.76593	53.185596	15	32	15	101.3	98.7	0.974	4.14E-01
Q5SW79	Centrosomal protein of 170 kDa	175.187	7.11	61.694135	7.3863636	10	13	10	101.3	98.7	0.974	4.22E-01
Q6R327	Rapamycin-insensitive companion of mTOR	192.097	7.47	21.597388	2.5761124	4	4	4	101.3	98.7	0.974	8.47E-02
Q86VQ1	Glucocorticoid-induced transcript 1 protein	57.988	9.44	10.412027	5.3016453	2	2	2	101.3	98.7	0.974	
Q86WB0	Nuclear-interacting partner of ALK	55.226	5.62	25.730886	14.541833	7	7	7	101.3	98.7	0.974	5.96E-02
Q8IUD2	ELKS/Rab6-interacting/CAST family member 1	128.008	5.97	112.38498	13.978495	17	23	17	101.3	98.7	0.974	1.91E-01
Q8IWE2	Protein NOXP20	60.704	4.68	47.166152	7.4600355	4	7	4	101.3	98.7	0.974	6.13E-02
Q8N766	ER membrane protein complex subunit 1	111.689	7.66	18.42318	5.4380665	6	6	6	101.3	98.7	0.974	4.27E-01
Q8TCG1	Protein CIP2A	102.121	6.23	61.374123	13.59116	11	13	11	101.3	98.7	0.974	2.45E-01
Q8TCJ2	:hyl-diphosphooligosaccharide--protein glycosyltransferase subunit ST	93.614	8.91	25.768784	5.811138	5	8	4	101.3	98.7	0.974	4.89E-02
Q96E29	Transcription termination factor 3, mitochondrial	47.941	8.53	7.6679758	5.5155875	2	2	2	101.3	98.7	0.974	8.50E-01
Q96RQ3	Methylcrotonoyl-CoA carboxylase subunit alpha, mitochondrial	80.422	7.78	21.337468	5.2413793	3	3	3	101.3	98.7	0.974	1.13E-01
Q96T60	Bifunctional polynucleotide phosphatase/kinase	57.04	8.46	12.440538	6.9097889	4	4	4	101.3	98.7	0.974	1.06E-01
Q9BTT0	Acidic leucine-rich nuclear phosphoprotein 32 family member E	30.674	3.85	61.863915	20.522388	6	8	6	101.3	98.7	0.974	1.71E-01
Q9BWH6	RNA polymerase II-associated protein 1	152.659	6.38	28.19188	4.7379756	4	4	4	101.3	98.7	0.974	2.97E-01
Q9BXR0	Queuine tRNA-ribosyltransferase	44.019	7.23	39.34551	18.114144	6	8	6	101.3	98.7	0.974	1.55E-03
Q9C0D5	Protein TANC1	202.093	8.32	6.0794071	0.5910801	1	1	1	101.3	98.7	0.974	

Q9HBL0	Tensin-1	185.586	7.75	29.597533	3.6887608	5	5	3	101.3	98.7	0.974	2.82E-01
Q9NWU5	39S ribosomal protein L22, mitochondrial	23.626	9.94	10.483397	6.3106796	1	2	1	101.3	98.7	0.974	
Q9P016	Thymocyte nuclear protein 1	25.681	9.25	38.580664	29.333333	8	12	8	101.3	98.7	0.974	1.87E-01
Q9P0K7	Ankycorbin	109.973	6.21	88.345353	13.367347	13	16	13	101.3	98.7	0.974	2.10E-02
Q9P260	LisH domain and HEAT repeat-containing protein KIAA1468	134.545	5.45	3.7033348	0.8223684	1	1	1	101.3	98.7	0.974	
Q9P2E9	Ribosome-binding protein 1	152.381	8.6	388.42473	34.751773	43	73	42	101.3	98.7	0.974	3.17E-03
Q9P2W1	Homologous-pairing protein 2 homolog	24.891	7.81	8.9288547	5.9907834	1	1	1	101.3	98.7	0.974	
Q9UBP6	tRNA (guanine-N(7)-)-methyltransferase	31.451	7.64	32.490705	14.130435	4	6	4	101.3	98.7	0.974	3.61E-01
Q9UDT6	CAP-Gly domain-containing linker protein 2	115.767	6.73	67.039798	10.133843	10	12	3	101.3	98.7	0.974	4.55E-02
Q9UGP8	Translocation protein SEC63 homolog	87.942	5.31	47.283835	10	6	7	6	101.3	98.7	0.974	2.59E-01
Q9ULV3	Cip1-interacting zinc finger protein	99.983	6.11	12.807256	3.5634744	2	2	2	101.3	98.7	0.974	
Q9Y230	RuvB-like 2	51.125	5.64	181.36375	42.332613	19	37	19	101.3	98.7	0.974	1.17E-02
Q9Y2S6	Translation machinery-associated protein 7	7.062	9.99	9.6890409	14.0625	1	2	1	101.3	98.7	0.974	
Q9Y5J5	Pleckstrin homology-like domain family A member 3	13.882	9.67	10.901791	14.96063	2	2	2	101.3	98.7	0.974	
Q9Y5P6	Mannose-1-phosphate guanyltransferase beta	39.809	6.61	35.598231	12.777778	4	6	4	101.3	98.7	0.974	2.27E-01
O00165	HCLS1-associated protein X-1	31.601	4.92	20.004945	15.412186	4	4	4	101.2	98.8	0.976	7.84E-02
O00483	Cytochrome c oxidase subunit NDUFA4	9.364	9.38	14.563913	23.45679	2	5	2	101.2	98.8	0.976	9.20E-02
O15511	Actin-related protein 2/3 complex subunit 5	16.31	5.67	23.833224	20.529801	2	3	2	101.2	98.8	0.976	3.69E-01
O43583	Density-regulated protein	22.078	5.3	7.8920424	8.0808081	2	3	2	101.2	98.8	0.976	7.12E-03
O43768	Alpha-endosulfine	13.381	7.24	8.8553533	19.834711	2	2	2	101.2	98.8	0.976	
O60333	Kinesin-like protein KIF1B	204.349	5.6	46.724709	4.2951542	7	9	4	101.2	98.8	0.976	2.37E-01
O94826	Mitochondrial import receptor subunit TOM70	67.412	7.12	83.2335	22.697368	14	18	14	101.2	98.8	0.976	5.95E-01
O95831	Apoptosis-inducing factor 1, mitochondrial	66.859	8.95	148.83571	28.874388	15	29	15	101.2	98.8	0.976	4.29E-01
P05166	Propionyl-CoA carboxylase beta chain, mitochondrial	58.179	7.64	26.583731	11.131725	5	5	5	101.2	98.8	0.976	3.44E-01
P09488	Glutathione S-transferase Mu 1	25.695	6.7	81.888802	54.587156	13	23	4	101.2	98.8	0.976	1.20E-01
P10586	Receptor-type tyrosine-protein phosphatase F	212.744	6.3	17.709672	1.5731515	2	2	2	101.2	98.8	0.976	
P10619	Lysosomal protective protein	54.431	6.61	32.43499	6.875	3	4	3	101.2	98.8	0.976	1.72E-02
P11802	Cyclin-dependent kinase 4	33.708	7.01	50.006558	27.062706	7	13	6	101.2	98.8	0.976	4.86E-01
P14923	Junction plakoglobin	81.693	6.14	126.44668	29.261745	18	29	17	101.2	98.8	0.976	2.63E-01
P16949	Stathmin	17.292	5.97	114.92325	61.073826	12	46	12	101.2	98.8	0.976	4.06E-01
P21333	Filamin-A	280.564	6.06	1211.6045	47.147714	100	279	93	101.2	98.8	0.976	2.18E-06
P26373	60S ribosomal protein L13	24.247	11.65	61.456577	32.701422	8	19	8	101.2	98.8	0.976	2.97E-01
P28066	Proteasome subunit alpha type-5	26.394	4.79	125.78873	41.078838	9	34	9	101.2	98.8	0.976	1.64E-02
P34896	Serine hydroxymethyltransferase, cytosolic	53.049	7.71	47.678683	17.805383	8	11	8	101.2	98.8	0.976	5.71E-01
P34932	Heat shock 70 kDa protein 4	94.271	5.19	456.64181	54.047619	40	101	38	101.2	98.8	0.976	2.15E-01
P39880	Homeobox protein cut-like 1	164.087	5.9	38.566299	4.9169435	7	7	7	101.2	98.8	0.976	5.97E-02
P46926	Glucosamine-6-phosphate isomerase 1	32.648	6.92	46.866511	19.723183	6	13	5	101.2	98.8	0.976	6.48E-01
P46937	Transcriptional coactivator YAP1	54.427	5.17	67.927422	26.388889	8	10	8	101.2	98.8	0.976	4.11E-01
P50991	T-complex protein 1 subunit delta	57.888	7.83	325.20578	60.296846	27	69	26	101.2	98.8	0.976	9.56E-01
P52735	Guanine nucleotide exchange factor VAV2	101.224	7.08	102.82109	16.742597	12	17	12	101.2	98.8	0.976	2.84E-01
P55039	Developmentally-regulated GTP-binding protein 2	40.72	8.88	25.049808	11.263736	3	4	3	101.2	98.8	0.976	2.21E-02
P68036	Ubiquitin-conjugating enzyme E2 L3	17.85	8.51	43.747806	25.974026	3	11	3	101.2	98.8	0.976	2.90E-02
Q04206	Transcription factor p65	60.181	5.68	54.073144	17.241379	8	11	8	101.2	98.8	0.976	9.10E-02
Q06323	Proteasome activator complex subunit 1	28.705	6.02	71.155927	31.726908	8	12	8	101.2	98.8	0.976	5.26E-01
Q07020	60S ribosomal protein L18	21.621	11.72	50.612399	26.595745	5	10	5	101.2	98.8	0.976	8.83E-01
Q0VDG4	Secernin-3	48.513	5.55	16.23329	11.084906	4	5	4	101.2	98.8	0.976	2.03E-01
Q14554	Protein disulfide-isomerase A5	59.556	7.91	26.210843	10.597303	5	6	5	101.2	98.8	0.976	1.73E-02

Q14571	Inositol 1,4,5-trisphosphate receptor type 2	307.867	6.43	32.86423	2.2954461	8	8	1	101.2	98.8	0.976	
Q15811	Intersectin-1	195.3	7.77	21.878808	3.4863451	5	5	4	101.2	98.8	0.976	
Q16891	MICOS complex subunit MIC60	83.626	6.48	222.42622	41.029024	30	50	30	101.2	98.8	0.976	9.79E-02
Q5JTZ9	Alanine--tRNA ligase, mitochondrial	107.273	6.27	13.253481	2.9441624	3	4	3	101.2	98.8	0.976	9.44E-04
Q69YN2	CWF19-like protein 1	60.581	7.24	37.984085	11.710037	6	7	6	101.2	98.8	0.976	9.14E-01
Q6P4R8	Nuclear factor related to kappa-B-binding protein	138.915	9.25	3.1721786	1.0007698	1	1	1	101.2	98.8	0.976	
Q6UXV4	MICOS complex subunit MIC27	29.14	9.52	13.002127	9.7014925	2	2	2	101.2	98.8	0.976	
Q7Z406	Myosin-14	227.732	5.6	113.06822	5.9649123	12	24	4	101.2	98.8	0.976	7.42E-01
Q8NB37	Parkinson disease 7 domain-containing protein 1	23.283	6.61	11.831347	4.5454545	1	4	1	101.2	98.8	0.976	
Q8NCC3	Group XV phospholipase A2	46.628	6.73	10.087833	7.038835	2	2	2	101.2	98.8	0.976	3.72E-01
Q8TEM1	Nuclear pore membrane glycoprotein 210	204.983	6.81	112.99356	8.9030207	17	24	17	101.2	98.8	0.976	5.95E-01
Q92665	28S ribosomal protein S31, mitochondrial	45.29	9.29	32.093747	17.721519	5	5	5	101.2	98.8	0.976	3.27E-01
Q96HW7	Integrator complex subunit 4	108.102	6.44	6.1701106	1.7653167	2	2	2	101.2	98.8	0.976	
Q9BRX5	DNA replication complex GINS protein PSF3	24.519	5.34	45.64329	29.62963	5	8	5	101.2	98.8	0.976	3.20E-02
Q9BXK5	Bcl-2-like protein 13	52.691	4.44	18.68209	2.8865979	1	3	1	101.2	98.8	0.976	
Q9H6S0	Probable ATP-dependent RNA helicase YTHDC2	160.147	8.4	20.608463	2.9370629	3	3	3	101.2	98.8	0.976	6.02E-02
Q9HD20	Manganese-transporting ATPase 13A1	132.87	8.13	32.837654	5.8139535	6	8	6	101.2	98.8	0.976	8.07E-02
Q9NZN5	Rho guanine nucleotide exchange factor 12	173.125	5.74	10.396319	2.3316062	4	4	4	101.2	98.8	0.976	4.86E-01
Q9P015	39S ribosomal protein L15, mitochondrial	33.399	10.01	25.77911	19.932432	5	5	5	101.2	98.8	0.976	1.10E-02
Q9UMX0	Ubiquilin-1	62.479	5.11	96.676531	17.996604	7	18	2	101.2	98.8	0.976	4.19E-01
Q9UPN3	Microtubule-actin cross-linking factor 1, isoforms 1/2/3/5	837.787	5.39	383.65797	8.6491608	51	62	51	101.2	98.8	0.976	1.34E-02
Q9UQR1	Zinc finger protein 148	88.921	6.48	7.7418089	2.1410579	2	2	2	101.2	98.8	0.976	
Q9Y248	DNA replication complex GINS protein PSF2	21.414	5.44	31.820873	24.864865	4	6	4	101.2	98.8	0.976	8.11E-01
Q9Y376	Calcium-binding protein 39	39.844	6.89	22.102217	12.316716	5	6	5	101.2	98.8	0.976	2.68E-01
Q9Y4X5	E3 ubiquitin-protein ligase ARIH1	64.076	5.08	13.866138	4.4883303	2	4	2	101.2	98.8	0.976	1.12E-01
O00308	NEDD4-like E3 ubiquitin-protein ligase WWP2	98.85	7.12	3.4494048	1.4942529	1	1	1	101.1	98.9	0.978	
O14656	Torsin-1A	37.784	6.99	21.353913	9.3373494	3	4	3	101.1	98.9	0.978	5.39E-01
O43707	Alpha-actinin-4	104.788	5.44	692.25482	56.750823	51	210	34	101.1	98.9	0.978	7.08E-03
O43708	Maleylacetoacetate isomerase	24.197	8.54	5.0477889	4.1666667	1	1	1	101.1	98.9	0.978	
O75352	Mannose-P-dolichol utilization defect 1 protein	26.62	8.94	9.2088787	4.048583	1	4	1	101.1	98.9	0.978	
O75530	Polycomb protein EED	50.166	7.03	34.894315	16.780045	6	8	6	101.1	98.9	0.978	4.32E-01
O75792	Ribonuclease H2 subunit A	33.374	5.25	26.520171	23.411371	6	6	6	101.1	98.9	0.978	5.86E-01
O76003	Glutaredoxin-3	37.408	5.39	100.9651	28.358209	9	21	9	101.1	98.9	0.978	8.67E-02
P01033	Metalloproteinase inhibitor 1	23.156	8.1	2.6534514	3.3816425	1	1	1	101.1	98.9	0.978	
P04843	olichyl-diphosphooligosaccharide--protein glycosyltransferase subunit	68.527	6.38	171.52635	33.113674	21	61	21	101.1	98.9	0.978	1.84E-02
P10644	cAMP-dependent protein kinase type I-alpha regulatory subunit	42.955	5.35	38.301884	11.548556	4	8	4	101.1	98.9	0.978	8.98E-02
P17858	ATP-dependent 6-phosphofructokinase, liver type	84.964	7.5	159.12502	23.974359	15	26	12	101.1	98.9	0.978	1.34E-01
P21796	Voltage-dependent anion-selective channel protein 1	30.754	8.54	192.83514	69.964664	16	45	14	101.1	98.9	0.978	3.29E-01
P21912	uccinate dehydrogenase [ubiquinone] iron-sulfur subunit, mitochondri	31.609	8.76	40.913582	21.428571	6	11	6	101.1	98.9	0.978	9.73E-01
P28074	Proteasome subunit beta type-5	28.462	6.92	65.944266	31.939163	8	16	8	101.1	98.9	0.978	8.32E-01
P30101	Protein disulfide-isomerase A3	56.747	6.35	301.45464	52.079208	29	95	29	101.1	98.9	0.978	6.07E-03
P32780	General transcription factor IIH subunit 1	61.993	8.66	20.079692	6.9343066	4	4	4	101.1	98.9	0.978	6.12E-01
P42575	Caspase-2	50.652	6.81	23.120558	10.176991	3	3	3	101.1	98.9	0.978	4.79E-01
P47755	F-actin-capping protein subunit alpha-2	32.929	5.85	74.983611	43.706294	9	13	8	101.1	98.9	0.978	2.99E-01
P49189	4-trimethylaminobutyraldehyde dehydrogenase	53.767	5.87	77.52093	21.659919	10	16	10	101.1	98.9	0.978	1.75E-02
P49721	Proteasome subunit beta type-2	22.822	7.02	45.72392	21.393035	5	10	5	101.1	98.9	0.978	3.90E-01
P50151	Guanine nucleotide-binding protein G(I)/G(S)/G(O) subunit gamma-1(	7.201	7.85	10.798876	26.470588	1	1	1	101.1	98.9	0.978	

P50225	Sulfotransferase 1A1	34.143	6.62	21.864245	17.288136	5	6	2	101.1	98.9	0.978	
P50552	Vasodilator-stimulated phosphoprotein	39.805	8.94	90.972519	30.789474	10	24	10	101.1	98.9	0.978	1.38E-02
P51148	Ras-related protein Rab-5C	23.468	8.41	77.435214	37.5	6	17	4	101.1	98.9	0.978	5.33E-01
P54252	Ataxin-3	41.754	4.91	6.0131387	2.7472527	1	1	1	101.1	98.9	0.978	
P57105	Synaptojanin-2-binding protein	15.918	6.3	24.936908	17.931034	2	5	2	101.1	98.9	0.978	8.09E-02
P61604	10 kDa heat shock protein, mitochondrial	10.925	8.92	69.251831	69.607843	8	24	8	101.1	98.9	0.978	2.36E-01
P61981	14-3-3 protein gamma	28.285	4.89	154.83964	48.987854	13	86	7	101.1	98.9	0.978	8.43E-01
Q01844	RNA-binding protein EWS	68.436	9.33	72.548869	9.6036585	5	16	5	101.1	98.9	0.978	5.41E-02
Q03252	Lamin-B2	67.647	5.35	154.86581	41.166667	27	37	22	101.1	98.9	0.978	3.39E-01
Q06830	Peroxiredoxin-1	22.096	8.13	183.25255	50.251256	13	83	10	101.1	98.9	0.978	5.32E-04
Q07812	Apoptosis regulator BAX	21.171	5.22	48.505246	35.9375	5	9	5	101.1	98.9	0.978	9.77E-01
Q08257	Quinone oxidoreductase	35.185	8.44	58.441795	28.571429	9	12	9	101.1	98.9	0.978	9.08E-01
Q08J23	tRNA (cytosine(34)-C(5))-methyltransferase	86.416	6.77	285.63829	46.936115	31	64	31	101.1	98.9	0.978	4.16E-02
Q13263	Transcription intermediary factor 1-beta	88.493	5.77	224.59189	33.892216	22	48	22	101.1	98.9	0.978	9.62E-01
Q13637	Ras-related protein Rab-32	24.982	6.54	37.613301	20.888889	4	6	4	101.1	98.9	0.978	6.07E-01
Q15907	Ras-related protein Rab-11B	24.473	5.94	72.245553	46.788991	9	15	9	101.1	98.9	0.978	4.57E-01
Q2M389	WASH complex subunit 7	136.316	7.44	13.015473	2.8985507	4	4	4	101.1	98.9	0.978	
Q2TAY7	WD40 repeat-containing protein SMU1	57.507	7.18	77.126617	27.095517	12	15	12	101.1	98.9	0.978	9.08E-02
Q53EP0	Fibronectin type III domain-containing protein 3B	132.803	5.95	22.781569	4.8172757	5	5	5	101.1	98.9	0.978	6.66E-01
Q53F19	Uncharacterized protein C17orf85	70.549	5.73	13.275692	3.7096774	2	2	2	101.1	98.9	0.978	7.20E-01
Q6NUK1	Calcium-binding mitochondrial carrier protein SCaMC-1	53.32	6.33	89.070098	25.995807	13	19	13	101.1	98.9	0.978	6.56E-02
Q7L5N1	COP9 signalosome complex subunit 6	36.14	5.73	56.077165	19.266055	5	10	5	101.1	98.9	0.978	8.47E-02
Q7Z478	ATP-dependent RNA helicase DHX29	155.139	8.09	32.939903	5.4784514	6	6	6	101.1	98.9	0.978	6.92E-01
Q86V48	Leucine zipper protein 1	120.202	8.5	58.033987	11.05948	11	13	11	101.1	98.9	0.978	1.42E-02
Q86X55	Histone-arginine methyltransferase CARM1	65.811	6.73	14.517156	6.25	5	5	5	101.1	98.9	0.978	6.42E-01
Q8IZQ5	Selenoprotein H	13.446	9.74	10.118495	17.213115	2	2	2	101.1	98.9	0.978	2.33E-01
Q8N9N8	Probable RNA-binding protein EIF1AD	19.041	5.21	23.081853	26.666667	3	4	3	101.1	98.9	0.978	4.61E-01
Q8NBN7	Retinol dehydrogenase 13	35.91	8.1	6.6503809	5.7401813	2	2	2	101.1	98.9	0.978	9.04E-01
Q8TAE8	Growth arrest and DNA damage-inducible proteins-interacting protein	25.368	10.02	54.281752	36.486486	6	9	6	101.1	98.9	0.978	3.76E-01
Q8TB61	Adenosine 3'-phospho 5'-phosphosulfate transporter 1	47.484	9.16	13.863434	9.4907407	4	5	4	101.1	98.9	0.978	
Q96B01	RAD51-associated protein 1	38.434	9.11	4.7804155	2.2727273	1	1	1	101.1	98.9	0.978	
Q96C57	Uncharacterized protein C12orf43	28.153	9.42	8.5919599	9.1603053	2	2	2	101.1	98.9	0.978	
Q96E11	Ribosome-recycling factor, mitochondrial	29.259	9.79	14.635419	6.1068702	1	2	1	101.1	98.9	0.978	
Q96EQ0	Small glutamine-rich tetratricopeptide repeat-containing protein beta	33.408	4.92	7.8127614	5.2631579	1	1	1	101.1	98.9	0.978	
Q96MW1	Coiled-coil domain-containing protein 43	25.233	4.92	19.328095	11.160714	3	3	3	101.1	98.9	0.978	5.41E-01
Q96N06	Spermatogenesis-associated protein 33	15.452	9.22	6.5310622	10.791367	1	1	1	101.1	98.9	0.978	
Q9BQS8	FYVE and coiled-coil domain-containing protein 1	166.879	4.92	13.051851	1.894452	2	2	2	101.1	98.9	0.978	6.88E-02
Q9BRR3	Transmembrane protein 246	46.558	7.59	13.193882	6.9478908	2	2	2	101.1	98.9	0.978	
Q9BU89	Deoxyhypusine hydroxylase	32.883	4.83	37.928709	23.178808	5	6	5	101.1	98.9	0.978	8.63E-02
Q9H0P0	Cytosolic 5'-nucleotidase 3A	37.924	7.12	25.24754	20.535714	6	6	6	101.1	98.9	0.978	2.14E-01
Q9H477	Ribokinase	34.121	5.05	16.326178	6.8322981	2	3	2	101.1	98.9	0.978	4.41E-01
Q9H8M7	Protein FAM188A	49.693	4.77	9.3964588	4.9438202	2	2	2	101.1	98.9	0.978	3.24E-01
Q9HBH0	Rho-related GTP-binding protein RhoF	23.61	8.65	15.161709	9.9526066	2	3	2	101.1	98.9	0.978	4.00E-01
Q9NRY4	Rho GTPase-activating protein 35	170.407	6.64	22.42286	1.8012008	3	4	3	101.1	98.9	0.978	7.02E-01
Q9P0L0	Vesicle-associated membrane protein-associated protein A	27.875	8.62	59.825002	24.899598	7	14	6	101.1	98.9	0.978	6.61E-01
Q9Y259	Choline/ethanolamine kinase	45.243	5.49	6.1394823	2.7848101	1	1	1	101.1	98.9	0.978	
O00267	Transcription elongation factor SPT5	120.925	5.06	119.76661	19.319227	17	22	17	101	99	0.98	4.82E-01

O00273	DNA fragmentation factor subunit alpha	36.5	4.79	59.679517	28.700906	8	11	8	101	99	0.98	6.52E-01
O00625	Pirin	32.093	6.92	47.823596	20.689655	5	13	5	101	99	0.98	6.68E-02
O14639	Actin-binding LIM protein 1	87.631	8.59	20.424539	6.0411311	4	4	4	101	99	0.98	8.10E-01
O43681	ATPase ASNA1	38.767	4.91	16.116065	11.494253	3	3	3	101	99	0.98	4.35E-01
O75439	Mitochondrial-processing peptidase subunit beta	54.331	6.83	71.117866	21.063395	10	17	9	101	99	0.98	1.89E-01
O96028	Histone-lysine N-methyltransferase NSD2	152.16	8.69	12.115074	2.7106227	4	4	4	101	99	0.98	6.39E-01
P00167	Cytochrome b5	15.321	4.96	34.15881	31.343284	4	7	4	101	99	0.98	1.27E-01
P04183	Thymidine kinase, cytosolic	25.452	8.51	33.677937	19.65812	5	8	5	101	99	0.98	2.93E-01
P06733	Alpha-enolase	47.139	7.39	463.55928	60.599078	26	255	22	101	99	0.98	1.44E-03
P13674	Prolyl 4-hydroxylase subunit alpha-1	61.011	6.01	62.214626	18.35206	8	11	8	101	99	0.98	2.22E-01
P22234	Multifunctional protein ADE2	47.049	7.23	197.51227	42.117647	19	45	19	101	99	0.98	5.27E-01
P22695	Cytochrome b-c1 complex subunit 2, mitochondrial	48.413	8.63	127.44282	29.580574	12	25	12	101	99	0.98	8.97E-01
P25788	Proteasome subunit alpha type-3	28.415	5.33	93.745616	30.980392	9	22	9	101	99	0.98	9.15E-01
P27816	Microtubule-associated protein 4	120.93	5.43	434.3712	43.142361	48	104	48	101	99	0.98	2.23E-04
P35080	Profilin-2	15.036	6.99	56.722238	43.571429	5	13	5	101	99	0.98	4.39E-01
P37840	Alpha-synuclein	14.451	4.7	92.553061	49.285714	6	10	6	101	99	0.98	1.57E-01
P49662	Caspase-4	43.235	6	8.3339075	6.6312997	3	3	3	101	99	0.98	8.92E-01
P51812	Ribosomal protein S6 kinase alpha-3	83.683	6.89	75.689096	18.243243	13	18	8	101	99	0.98	4.46E-01
P54136	Arginine--tRNA ligase, cytoplasmic	75.331	6.68	299.60441	41.515152	25	64	25	101	99	0.98	9.95E-01
P55809	Succinyl-CoA:3-ketoacid coenzyme A transferase 1, mitochondrial	56.122	7.46	148.65235	36.730769	16	42	16	101	99	0.98	4.57E-01
P60660	Myosin light polypeptide 6	16.919	4.65	102.06202	52.317881	9	30	9	101	99	0.98	1.29E-01
P62805	Histone H4	11.36	11.36	111.23081	59.223301	9	60	9	101	99	0.98	1.55E-01
Q12906	Interleukin enhancer-binding factor 3	95.279	8.76	190.49721	24.161074	22	53	19	101	99	0.98	4.77E-01
Q13503	Mediator of RNA polymerase II transcription subunit 21	15.555	4.45	9.8043771	13.888889	1	1	1	101	99	0.98	
Q13795	ADP-ribosylation factor-related protein 1	22.599	7.56	8.1995579	8.9552239	1	1	1	101	99	0.98	
Q14004	Cyclin-dependent kinase 13	164.823	9.69	11.258462	1.1243386	2	3	1	101	99	0.98	
Q14376	UDP-glucose 4-epimerase	38.257	6.73	62.227247	19.827586	6	11	6	101	99	0.98	4.14E-01
Q14493	Histone RNA hairpin-binding protein	31.266	7.47	8.8992849	7.037037	1	1	1	101	99	0.98	
Q147X3	N-alpha-acetyltransferase 30	39.295	5.52	14.049172	8.5635359	2	2	2	101	99	0.98	5.50E-01
Q16822	Phosphoenolpyruvate carboxykinase [GTP], mitochondrial	70.685	7.62	31.171745	9.6875	5	6	5	101	99	0.98	3.33E-01
Q6GMV2	SET and MYND domain-containing protein 5	47.31	5.05	7.2602695	2.6315789	1	1	1	101	99	0.98	
Q6IA86	Elongator complex protein 2	92.441	5.96	29.381077	8.2324455	6	6	6	101	99	0.98	5.48E-01
Q6NUQ4	Transmembrane protein 214	77.101	9.14	57.707281	13.497823	8	14	8	101	99	0.98	5.91E-01
Q6ZSZ5	Rho guanine nucleotide exchange factor 18	130.7	7.08	12.265638	2.3870418	3	4	2	101	99	0.98	2.99E-02
Q7Z6E9	E3 ubiquitin-protein ligase RBBP6	201.442	9.64	48.729551	5.1339286	8	9	8	101	99	0.98	1.91E-01
Q86YS7	C2 domain-containing protein 5	110.377	5.69	24.719348	6.4	5	5	5	101	99	0.98	5.54E-01
Q8NEZ2	Vacuolar protein sorting-associated protein 37A	44.287	5.57	10.87517	5.0377834	1	1	1	101	99	0.98	
Q8WX92	Negative elongation factor B	65.655	6.13	55.90793	16.37931	9	13	9	101	99	0.98	9.92E-01
Q92841	Probable ATP-dependent RNA helicase DDX17	80.222	8.27	268.58974	43.347051	30	76	21	101	99	0.98	4.33E-01
Q92974	Rho guanine nucleotide exchange factor 2	111.473	7.27	148.57746	22.312373	19	25	19	101	99	0.98	6.21E-01
Q96CP2	FLYWCH family member 2	14.555	8.46	51.185974	70	7	10	7	101	99	0.98	1.52E-01
Q96GX2	Putative ataxin-7-like protein 3B	10.764	4.36	3.5713793	10.309278	1	1	1	101	99	0.98	
Q96JJ3	Engulfment and cell motility protein 2	82.562	5.9	53.49165	14.444444	8	12	8	101	99	0.98	4.44E-01
Q96MG8	'rotein-L-isoaspartate O-methyltransferase domain-containing protein	40.65	5.66	4.9833845	2.5210084	1	1	1	101	99	0.98	
Q9BWT6	Meiotic nuclear division protein 1 homolog	23.738	8.19	7.3061851	6.8292683	1	1	1	101	99	0.98	
Q9GZT6	Coiled-coil domain-containing protein 90B, mitochondrial	29.487	7.55	3.3171332	3.9370079	1	1	1	101	99	0.98	
Q9H845	Acyl-CoA dehydrogenase family member 9, mitochondrial	68.717	7.96	78.082339	19.806763	13	22	13	101	99	0.98	3.52E-02

Q9NRF8	CTP synthase 2	65.636	6.9	57.96097	15.870307	10	12	8	101	99	0.98	5.38E-01
Q9NTZ6	RNA-binding protein 12	97.333	8.63	74.516099	14.377682	12	19	12	101	99	0.98	2.40E-01
Q9NYB0	Telomeric repeat-binding factor 2-interacting protein 1	44.233	4.73	4.086504	7.2681704	1	1	1	101	99	0.98	
Q9UHY1	Nuclear receptor-binding protein	59.807	5.08	65.204503	12.149533	6	9	6	101	99	0.98	5.68E-01
Q9UKJ3	G patch domain-containing protein 8	164.098	8.66	15.691354	1.930759	3	3	3	101	99	0.98	6.51E-01
Q9UL46	Proteasome activator complex subunit 2	27.384	5.73	87.570533	41.422594	9	17	9	101	99	0.98	9.50E-01
Q9UNF0	Protein kinase C and casein kinase substrate in neurons protein 2	55.704	5.2	49.344	19.547325	8	10	8	101	99	0.98	4.38E-01
Q9UQ80	Proliferation-associated protein 2G4	43.759	6.55	174.25969	49.238579	17	52	17	101	99	0.98	7.50E-01
O14773	Tripeptidyl-peptidase 1	61.21	6.48	19.600053	6.9271758	3	4	3	100.9	99.1	0.982	
O15173	Membrane-associated progesterone receptor component 2	23.804	4.88	6.5066532	7.1748879	2	2	2	100.9	99.1	0.982	
O60437	Periplakin	204.623	5.6	193.59839	18.906606	28	32	28	100.9	99.1	0.982	6.92E-02
O75884	Putative hydrolase RBBP9	20.986	6.2	8.0298392	9.1397849	1	1	1	100.9	99.1	0.982	
O95365	Zinc finger and BTB domain-containing protein 7A	61.401	5.19	18.513428	3.9383562	1	1	1	100.9	99.1	0.982	
P02786	Transferrin receptor protein 1	84.818	6.61	166.8937	30.131579	20	30	20	100.9	99.1	0.982	1.58E-01
P06576	ATP synthase subunit beta, mitochondrial	56.525	5.4	306.56972	49.52741	18	84	18	100.9	99.1	0.982	5.27E-01
P08729	Keratin, type II cytoskeletal 7	51.354	5.48	235.79923	44.989339	26	65	21	100.9	99.1	0.982	7.37E-03
P16383	GC-rich sequence DNA-binding factor 2	89.329	5.99	3.0710923	2.3047375	1	1	1	100.9	99.1	0.982	
P20073	Annexin A7	52.706	5.68	148.5238	35.245902	17	36	17	100.9	99.1	0.982	1.16E-01
P20962	Parathymosin	11.523	4.16	37.372013	22.54902	3	35	3	100.9	99.1	0.982	9.34E-02
P29218	Inositol monophosphatase 1	30.169	5.26	42.134626	23.826715	6	8	6	100.9	99.1	0.982	6.24E-01
P30049	ATP synthase subunit delta, mitochondrial	17.479	5.49	31.340839	13.690476	2	6	2	100.9	99.1	0.982	1.43E-01
P32320	Cytidine deaminase	16.174	6.92	58.898488	30.136986	3	11	3	100.9	99.1	0.982	4.45E-02
P35250	Replication factor C subunit 2	39.132	6.44	70.906159	30.79096	10	13	10	100.9	99.1	0.982	9.14E-01
P39687	Acidic leucine-rich nuclear phosphoprotein 32 family member A	28.568	4.09	74.874638	29.317269	9	21	5	100.9	99.1	0.982	1.24E-01
P42229	Signal transducer and activator of transcription 5A	90.59	6.39	115.29318	19.017632	16	24	3	100.9	99.1	0.982	3.90E-01
P49591	Serine--tRNA ligase, cytoplasmic	58.74	6.43	77.283135	27.042802	14	23	14	100.9	99.1	0.982	7.12E-01
P50570	Dynamin-2	98.003	7.44	80.289316	16.896552	14	21	14	100.9	99.1	0.982	4.60E-01
P52565	Rho GDP-dissociation inhibitor 1	23.193	5.11	63.363253	30.392157	6	15	6	100.9	99.1	0.982	5.20E-02
P52594	Arf-GAP domain and FG repeat-containing protein 1	58.224	8.63	16.777123	7.4733096	4	4	4	100.9	99.1	0.982	6.31E-01
P67812	Signal peptidase complex catalytic subunit SEC11A	20.612	9.48	20.061477	20.111732	5	9	5	100.9	99.1	0.982	9.50E-02
P86790	Vacuolar fusion protein CCZ1 homolog B	55.83	6.48	11.149793	4.9792531	2	2	2	100.9	99.1	0.982	1.22E-02
Q00610	Clathrin heavy chain 1	191.493	5.69	856.23554	42.447761	60	163	45	100.9	99.1	0.982	1.27E-01
Q01469	Fatty acid-binding protein, epidermal	15.155	7.01	26.634555	30.37037	3	4	3	100.9	99.1	0.982	7.90E-01
Q08378	Golgin subfamily A member 3	167.252	5.44	139.00908	16.154873	21	23	21	100.9	99.1	0.982	4.62E-01
Q13332	Receptor-type tyrosine-protein phosphatase S	216.905	6.46	9.4617219	1.0780287	2	2	2	100.9	99.1	0.982	
Q13724	Mannosyl-oligosaccharide glucosidase	91.861	8.9	64.140023	12.425329	8	13	8	100.9	99.1	0.982	1.46E-02
Q13813	Spectrin alpha chain, non-erythrocytic 1	284.364	5.35	995.9895	42.961165	95	184	95	100.9	99.1	0.982	2.40E-02
Q15714	TSC22 domain family protein 1	109.61	5.64	29.502572	2.6095061	3	9	3	100.9	99.1	0.982	3.67E-01
Q15813	Tubulin-specific chaperone E	59.309	6.76	53.797468	13.662239	7	12	6	100.9	99.1	0.982	4.40E-01
Q5JWF2	Guanine nucleotide-binding protein G(s) subunit alpha isoforms XLas	110.956	5.03	45.193356	5.4001929	5	12	4	100.9	99.1	0.982	7.58E-01
Q5VTL8	Pre-mRNA-splicing factor 38B	64.429	10.54	44.795859	10.07326	7	11	7	100.9	99.1	0.982	5.05E-01
Q658P3	Metalloreductase STEAP3	54.566	8.6	24.03881	10.860656	4	5	4	100.9	99.1	0.982	4.68E-01
Q6P3W7	SCY1-like protein 2	103.642	8.22	24.488797	6.1356297	4	4	4	100.9	99.1	0.982	2.93E-01
Q709C8	Vacuolar protein sorting-associated protein 13C	422.124	6.83	26.782743	1.4388489	6	7	6	100.9	99.1	0.982	2.26E-01
Q7Z7L7	Protein zer-1 homolog	88.113	5.62	3.3653212	0.9138381	1	1	1	100.9	99.1	0.982	
Q8IUH3	RNA-binding protein 45	53.469	7.17	4.4119525	1.8907563	1	1	1	100.9	99.1	0.982	
Q8IVF2	Protein AHNAK2	616.242	5.36	266.11455	20.793788	37	51	36	100.9	99.1	0.982	8.52E-01

Q92900	Regulator of nonsense transcripts 1	124.267	6.61	214.23592	27.192205	25	36	25	100.9	99.1	0.982	9.34E-01
Q96TA1	Niban-like protein 1	84.085	6.19	136.18205	25.603217	17	33	17	100.9	99.1	0.982	9.81E-01
Q9BT30	pha-ketoglutarate-dependent dioxygenase alkB homolog 7, mitochondı	24.501	7.11	7.1363203	9.9547511	1	1	1	100.9	99.1	0.982	
Q9BVG9	Phosphatidylserine synthase 2	56.216	6.25	6.3385659	3.0800821	1	1	1	100.9	99.1	0.982	
Q9BZF9	Uveal autoantigen with coiled-coil domains and ankyrin repeats	162.404	7.03	39.508371	4.8728814	6	6	6	100.9	99.1	0.982	2.94E-01
Q9C0J8	pre-mRNA 3' end processing protein WDR33	145.799	9.17	26.493322	3.5179641	4	6	4	100.9	99.1	0.982	2.22E-01
Q9H173	Nucleotide exchange factor SIL1	52.052	5.36	9.3140391	4.989154	2	2	2	100.9	99.1	0.982	
Q9H1E3	Nuclear ubiquitous casein and cyclin-dependent kinase substrate 1	27.28	5.08	32.439033	21.8107	4	9	4	100.9	99.1	0.982	5.68E-01
Q9HDC9	Adipocyte plasma membrane-associated protein	46.451	6.16	30.566476	17.067308	7	9	7	100.9	99.1	0.982	7.72E-01
Q9NPA0	ER membrane protein complex subunit 7	26.454	9.25	8.163643	9.5041322	2	2	2	100.9	99.1	0.982	
Q9P0V3	SH3 domain-binding protein 4	107.428	7.71	35.438465	5.6074766	5	7	5	100.9	99.1	0.982	1.72E-01
Q9P2N5	RNA-binding protein 27	118.645	9.19	112.37988	15.377358	14	19	12	100.9	99.1	0.982	4.27E-01
Q9Y4F1	FERM, RhoGEF and pleckstrin domain-containing protein 1	118.559	8.15	25.608749	4.4976077	4	5	4	100.9	99.1	0.982	3.92E-01
Q9Y5V0	Zinc finger protein 706	8.492	10.01	2.6983191	15.789474	1	1	1	100.9	99.1	0.982	
Q9Y5X3	Sorting nexin-5	46.787	6.76	57.990457	24.257426	9	13	7	100.9	99.1	0.982	5.78E-01
O14530	Thioredoxin domain-containing protein 9	26.517	5.88	16.077853	13.716814	3	5	3	100.8	99.2	0.984	
O43314	sitol hexakisphosphate and diphosphoinositol-pentakisphosphate kinas	140.318	8.22	2.9446217	0.5631537	1	1	1	100.8	99.2	0.984	
O60763	General vesicular transport factor p115	107.828	4.91	158.04479	20.686071	15	23	15	100.8	99.2	0.984	6.93E-01
O60936	Nucleolar protein 3	22.616	4.18	38.987505	23.076923	3	5	3	100.8	99.2	0.984	4.06E-02
O75164	Lysine-specific demethylase 4A	120.585	5.85	6.3300326	1.1278195	1	2	1	100.8	99.2	0.984	
O75380	NADH dehydrogenase [ubiquinone] iron-sulfur protein 6, mitochondria	13.703	8.28	31.502796	21.774194	2	5	2	100.8	99.2	0.984	3.67E-01
O75643	U5 small nuclear ribonucleoprotein 200 kDa helicase	244.353	6.06	434.67234	25.187266	50	85	50	100.8	99.2	0.984	1.08E-01
O95155	Ubiquitin conjugation factor E4 B	146.092	6.55	62.214537	9.984639	10	11	10	100.8	99.2	0.984	6.84E-01
O95394	Phosphoacetylglucosamine mutase	59.814	6.25	53.968288	17.712177	9	12	9	100.8	99.2	0.984	1.00E+00
O95721	Synaptosomal-associated protein 29	28.953	5.81	43.914441	26.356589	6	9	6	100.8	99.2	0.984	5.58E-01
O95785	Protein Wiz	178.563	6.86	22.598981	2.7861902	4	5	4	100.8	99.2	0.984	9.65E-01
P01023	Alpha-2-macroglobulin	163.188	6.46	18.928893	2.5101764	4	6	4	100.8	99.2	0.984	6.21E-01
P07355	Annexin A2	38.58	7.75	279.61374	62.536873	25	121	25	100.8	99.2	0.984	3.79E-01
P08195	4F2 cell-surface antigen heavy chain	67.952	5.01	229.42469	32.539683	18	49	18	100.8	99.2	0.984	9.43E-01
P10606	Cytochrome c oxidase subunit 5B, mitochondrial	13.687	8.81	27.453071	24.806202	4	6	4	100.8	99.2	0.984	5.03E-01
P10909	Clusterin	52.461	6.27	22.588025	10.022272	3	3	3	100.8	99.2	0.984	1.02E-04
P12955	Xaa-Pro dipeptidase	54.513	6	93.110806	21.298174	9	15	9	100.8	99.2	0.984	2.91E-01
P16615	Sarcoplasmic/endoplasmic reticulum calcium ATPase 2	114.683	5.34	263.72491	29.270633	30	66	30	100.8	99.2	0.984	9.15E-01
P21127	Cyclin-dependent kinase 11B	92.65	5.57	44.214223	13.207547	10	10	10	100.8	99.2	0.984	8.00E-01
P22059	Oxysterol-binding protein 1	89.365	7.3	78.789608	20.446097	14	17	14	100.8	99.2	0.984	3.00E-02
P28482	Mitogen-activated protein kinase 1	41.363	6.98	92.677785	36.666667	13	24	9	100.8	99.2	0.984	7.13E-02
P30085	UMP-CMP kinase	22.208	5.57	66.358466	21.938776	4	12	4	100.8	99.2	0.984	2.27E-01
P33991	DNA replication licensing factor MCM4	96.498	6.74	181.27566	30.822711	23	36	23	100.8	99.2	0.984	2.77E-01
P40616	ADP-ribosylation factor-like protein 1	20.404	5.72	42.671928	37.016575	4	5	4	100.8	99.2	0.984	2.49E-01
P49247	Ribose-5-phosphate isomerase	33.248	8.54	13.763387	8.3601286	3	4	3	100.8	99.2	0.984	5.53E-01
P49321	Nuclear autoantigenic sperm protein	85.186	4.3	164.89651	25.888325	19	36	18	100.8	99.2	0.984	6.61E-01
P49748	Very long-chain specific acyl-CoA dehydrogenase, mitochondrial	70.345	8.75	202.03904	36.48855	19	42	19	100.8	99.2	0.984	3.40E-02
P51948	CDK-activating kinase assembly factor MAT1	35.8	6.09	24.245014	10.032362	4	5	4	100.8	99.2	0.984	6.42E-01
P52564	Dual specificity mitogen-activated protein kinase kinase 6	37.468	7.39	33.006197	18.562874	6	7	4	100.8	99.2	0.984	7.89E-01
P52701	DNA mismatch repair protein Msh6	152.689	6.9	250.72898	25.073529	30	45	30	100.8	99.2	0.984	1.30E-01
P53007	Tricarboxylate transport protein, mitochondrial	33.991	9.89	76.414397	24.758842	8	15	8	100.8	99.2	0.984	2.64E-01
P61513	60S ribosomal protein L37a	10.268	10.43	4.6292224	15.217391	2	4	1	100.8	99.2	0.984	

P62277	40S ribosomal protein S13	17.212	10.54	37.738908	47.682119	8	16	8	100.8	99.2	0.984	3.57E-01
Q04721	Neurogenic locus notch homolog protein 2	265.226	5.14	11.910123	1.3354917	3	3	2	100.8	99.2	0.984	5.68E-01
Q12824	ted matrix-associated actin-dependent regulator of chromatin subfamil	44.113	6.23	31.293546	15.844156	4	5	4	100.8	99.2	0.984	9.40E-01
Q13243	Serine/arginine-rich splicing factor 5	31.245	11.59	46.896092	27.573529	7	10	6	100.8	99.2	0.984	8.00E-01
Q13526	Peptidyl-prolyl cis-trans isomerase NIMA-interacting 1	18.232	8.82	23.655436	13.496933	2	2	2	100.8	99.2	0.984	4.64E-01
Q14847	LIM and SH3 domain protein 1	29.698	7.05	127.28469	45.977011	13	32	13	100.8	99.2	0.984	2.21E-01
Q15334	Lethal(2) giant larvae protein homolog 1	115.346	6.29	33.146857	7.7067669	5	5	5	100.8	99.2	0.984	1.10E-01
Q6NUK4	Receptor expression-enhancing protein 3	29.245	9.57	4.6054226	7.0588235	2	2	2	100.8	99.2	0.984	
Q7L8J4	SH3 domain-binding protein 5-like	43.473	5.77	2.9132843	2.0356234	1	1	1	100.8	99.2	0.984	
Q7LBC6	Lysine-specific demethylase 3B	191.461	7.18	48.237588	5.2243044	8	10	8	100.8	99.2	0.984	6.52E-01
Q7Z7H5	Transmembrane emp24 domain-containing protein 4	25.926	8.28	35.441925	25.991189	6	7	3	100.8	99.2	0.984	3.12E-02
Q86UE4	Protein LYRIC	63.799	9.32	83.247124	22.508591	10	14	10	100.8	99.2	0.984	5.19E-01
Q86X76	Nitrilase homolog 1	35.873	7.74	9.6338312	7.6452599	2	2	2	100.8	99.2	0.984	2.26E-01
Q8N0S6	Centromere protein L	38.973	6.52	5.853252	3.4883721	1	1	1	100.8	99.2	0.984	
Q8NB16	Mixed lineage kinase domain-like protein	54.445	8.82	17.152103	9.5541401	5	6	5	100.8	99.2	0.984	9.63E-01
Q8NBK3	Sulfatase-modifying factor 1	40.531	6.65	9.9888747	7.2192513	2	2	2	100.8	99.2	0.984	1.26E-01
Q8NE86	Calcium uniporter protein, mitochondrial	39.842	8.65	46.573783	19.94302	6	7	6	100.8	99.2	0.984	9.90E-01
Q8NG31	Protein CASC5	265.224	5.47	16.551745	1.4517506	4	4	4	100.8	99.2	0.984	6.29E-01
Q8WUF5	RelA-associated inhibitor	89.036	6.81	83.626887	16.545894	10	13	10	100.8	99.2	0.984	2.27E-01
Q8WUW1	Protein BRICK1	8.739	5.45	29.653773	64	5	9	5	100.8	99.2	0.984	8.82E-01
Q92734	Protein TFG	43.421	5.1	110.41989	30	9	19	9	100.8	99.2	0.984	8.17E-01
Q93009	Ubiquitin carboxyl-terminal hydrolase 7	128.22	5.55	202.1454	29.038113	27	40	27	100.8	99.2	0.984	5.66E-01
Q969F9	Hermansky-Pudlak syndrome 3 protein	113.662	6.43	14.770635	3.3864542	3	3	3	100.8	99.2	0.984	2.93E-01
Q96AY3	Peptidyl-prolyl cis-trans isomerase FKBP10	64.204	5.62	153.78765	27.835052	15	45	15	100.8	99.2	0.984	5.29E-01
Q96C92	Serologically defined colon cancer antigen 3	47.932	5.14	7.3118472	2.5287356	1	1	1	100.8	99.2	0.984	
Q96EB6	NAD-dependent protein deacetylase sirtuin-1	81.63	4.67	22.464795	6.5595716	4	4	4	100.8	99.2	0.984	9.09E-01
Q96HE7	ERO1-like protein alpha	54.358	5.68	52.740846	18.589744	7	8	7	100.8	99.2	0.984	2.42E-02
Q96II8	,eucine-rich repeat and calponin homology domain-containing protein	86.03	6.71	9.833195	2.5740026	2	2	2	100.8	99.2	0.984	
Q96T76	MMS19 nucleotide excision repair protein homolog	113.217	6.35	37.436206	8.4466019	7	7	7	100.8	99.2	0.984	5.17E-01
Q9BQC3	Diphthamide biosynthesis protein 2	52.05	5.53	6.8972234	3.8854806	1	1	1	100.8	99.2	0.984	
Q9BT78	COP9 signalosome complex subunit 4	46.24	5.83	123.99944	44.08867	15	20	14	100.8	99.2	0.984	1.57E-01
Q9BV57	1,2-dihydroxy-3-keto-5-methylthiopentene dioxygenase	21.485	5.68	22.240926	18.994413	3	5	3	100.8	99.2	0.984	2.65E-01
Q9H7N4	Splicing factor, arginine/serine-rich 19	139.186	9.25	14.133049	3.0487805	3	3	3	100.8	99.2	0.984	5.13E-02
Q9H9Q2	COP9 signalosome complex subunit 7b	29.603	6.15	49.72633	16.287879	3	6	3	100.8	99.2	0.984	4.52E-01
Q9HC38	Glyoxalase domain-containing protein 4	34.771	5.6	51.18593	27.795527	9	16	9	100.8	99.2	0.984	2.47E-01
Q9NPJ8	NTF2-related export protein 2	16.218	5.48	4.3322669	5.6338028	1	1	1	100.8	99.2	0.984	
Q9NRD1	F-box only protein 6	33.911	6.09	8.4990942	4.0955631	1	2	1	100.8	99.2	0.984	
Q9NVM4	Protein arginine N-methyltransferase 7	78.409	5.57	18.978571	4.7687861	3	4	3	100.8	99.2	0.984	2.13E-01
Q9NX40	OCIA domain-containing protein 1	27.609	7.49	40.144251	20.816327	5	6	5	100.8	99.2	0.984	2.71E-01
Q9NYR9	NF-kappa-B inhibitor-interacting Ras-like protein 2	21.495	8.05	10.032762	11.518325	2	2	2	100.8	99.2	0.984	
Q9NZ43	Vesicle transport protein USE1	29.352	9.07	16.850491	15.830116	4	4	4	100.8	99.2	0.984	2.99E-02
Q9P0U3	Sentrin-specific protease 1	73.435	8.47	8.5136104	3.1055901	2	2	2	100.8	99.2	0.984	4.00E-01
Q9UBQ7	Glyoxylate reductase/hydroxypyruvate reductase	35.646	7.39	47.48549	16.463415	5	10	5	100.8	99.2	0.984	4.85E-01
Q9UIL1	Short coiled-coil protein	18.034	8.85	5.5250557	6.2893082	1	1	1	100.8	99.2	0.984	
Q9UIV1	CCR4-NOT transcription complex subunit 7	32.724	4.84	20.564932	10.877193	3	4	2	100.8	99.2	0.984	
Q9UJY5	ADP-ribosylation factor-binding protein GGA1	70.34	5.29	9.546722	4.3818466	2	2	1	100.8	99.2	0.984	
Q9Y613	FH1/FH2 domain-containing protein 1	126.473	6.39	54.525732	7.4742268	8	9	8	100.8	99.2	0.984	7.50E-01

Q9Y619	Mitochondrial ornithine transporter 1	32.715	9.13	6.2095037	3.3222591	1	1	1	100.8	99.2	0.984	
O00268	Transcription initiation factor TFIID subunit 4	110.047	9.94	16.212922	1.843318	2	2	2	100.7	99.3	0.986	
O14497	AT-rich interactive domain-containing protein 1A	241.892	6.7	9.7345034	0.7002188	2	4	2	100.7	99.3	0.986	9.04E-01
O15027	Protein transport protein Sec16A	233.373	5.63	100.85879	8.4442405	14	17	14	100.7	99.3	0.986	1.44E-01
O15155	BET1 homolog	13.281	9.06	10.794525	15.254237	1	1	1	100.7	99.3	0.986	
O15228	Dihydroxyacetone phosphate acyltransferase	77.138	6.57	14.018161	2.7941176	2	2	2	100.7	99.3	0.986	4.63E-01
O75175	CCR4-NOT transcription complex subunit 3	81.822	6.2	15.188924	4.7808765	3	3	3	100.7	99.3	0.986	2.56E-02
O75396	Vesicle-trafficking protein SEC22b	24.578	6.92	117.16002	34.418605	7	27	7	100.7	99.3	0.986	2.54E-01
O75822	Eukaryotic translation initiation factor 3 subunit J	29.045	4.83	66.189499	31.007752	7	15	7	100.7	99.3	0.986	2.78E-01
O94913	Pre-mRNA cleavage complex 2 protein Pcf11	172.944	8.48	9.4315698	1.1575563	2	2	2	100.7	99.3	0.986	1.56E-01
O95302	Peptidyl-prolyl cis-trans isomerase FKBP9	63.044	5.08	42.585719	17.894737	8	10	8	100.7	99.3	0.986	2.26E-01
O95613	Pericentrin	377.806	5.55	4.9800533	0.5395683	1	1	1	100.7	99.3	0.986	
P04083	Annexin A1	38.69	7.02	266.66043	63.00578	22	78	22	100.7	99.3	0.986	2.77E-01
P10398	Serine/threonine-protein kinase A-Raf	67.542	9.01	6.4947392	3.30033	2	2	1	100.7	99.3	0.986	
P12270	Nucleoprotein TPR	267.131	5.02	579.81936	29.538722	66	98	66	100.7	99.3	0.986	5.55E-01
P16144	Integrin beta-4	202.039	6.09	26.584642	2.4149286	4	5	4	100.7	99.3	0.986	4.19E-01
P16219	Short-chain specific acyl-CoA dehydrogenase, mitochondrial	44.269	7.99	4.7612014	2.9126214	1	1	1	100.7	99.3	0.986	
P22314	Ubiquitin-like modifier-activating enzyme 1	117.774	5.76	323.04701	33.459357	31	87	31	100.7	99.3	0.986	1.28E-01
P23528	Cofilin-1	18.491	8.09	181.39057	74.698795	18	67	14	100.7	99.3	0.986	4.43E-01
P24386	Rab proteins geranylgeranyltransferase component A 1	73.429	4.75	5.4990778	1.3782542	1	1	1	100.7	99.3	0.986	
P30044	Peroxiredoxin-5, mitochondrial	22.073	8.7	149.92558	49.53271	9	39	9	100.7	99.3	0.986	8.20E-01
P31483	Nucleolysin TIA-1 isoform p40	42.936	7.74	52.554647	14.766839	5	8	3	100.7	99.3	0.986	1.75E-01
P31939	Bifunctional purine biosynthesis protein PURH	64.575	6.71	263.50098	45.945946	21	49	21	100.7	99.3	0.986	3.01E-01
P35249	Replication factor C subunit 4	39.657	8.02	97.857374	32.231405	10	15	10	100.7	99.3	0.986	2.40E-01
P35270	Sepiapterin reductase	28.031	8.05	53.291048	37.164751	7	9	7	100.7	99.3	0.986	6.96E-01
P36507	Dual specificity mitogen-activated protein kinase kinase 2	44.396	6.55	65.86214	29.75	10	15	8	100.7	99.3	0.986	2.38E-01
P45974	Ubiquitin carboxyl-terminal hydrolase 5	95.725	5.03	125.11713	22.610723	17	28	17	100.7	99.3	0.986	1.54E-01
P46939	Utrophin	394.22	5.33	50.084924	2.8837751	10	12	9	100.7	99.3	0.986	5.20E-01
P46940	Ras GTPase-activating-like protein IQGAP1	189.134	6.48	863.70249	44.779722	69	186	67	100.7	99.3	0.986	6.68E-02
P48163	NADP-dependent malic enzyme	64.109	6.13	37.799974	7.6923077	5	9	5	100.7	99.3	0.986	5.26E-01
P48449	Lanosterol synthase	83.255	6.61	34.887833	9.1530055	6	8	6	100.7	99.3	0.986	8.96E-01
P54652	Heat shock-related 70 kDa protein 2	69.978	5.74	115.4643	23.630673	16	48	4	100.7	99.3	0.986	3.20E-01
P56192	Methionine--tRNA ligase, cytoplasmic	101.052	6.16	146.22345	27.333333	19	27	19	100.7	99.3	0.986	5.59E-01
P61769	Beta-2-microglobulin	13.706	6.52	7.8941648	8.4033613	1	2	1	100.7	99.3	0.986	
P63010	AP-2 complex subunit beta	104.486	5.38	200.53566	23.79936	20	42	12	100.7	99.3	0.986	2.61E-01
P68402	Platelet-activating factor acetylhydrolase IB subunit beta	25.553	5.92	4.3723983	6.9868996	2	2	2	100.7	99.3	0.986	
P82914	28S ribosomal protein S15, mitochondrial	29.823	10.48	9.7968664	11.673152	3	3	3	100.7	99.3	0.986	8.79E-01
Q02880	DNA topoisomerase 2-beta	183.152	8	135.14987	11.931119	20	36	9	100.7	99.3	0.986	3.28E-01
Q04695	Keratin, type I cytoskeletal 17	48.076	5.02	239.96751	49.537037	24	66	23	100.7	99.3	0.986	4.85E-01
Q05639	Elongation factor 1-alpha 2	50.438	9.03	193.07017	34.989201	16	73	9	100.7	99.3	0.986	1.32E-01
Q09666	Neuroblast differentiation-associated protein AHNAK	628.699	6.15	2065.3142	59.337861	196	479	195	100.7	99.3	0.986	2.51E-02
Q12797	Aspartyl/asparaginyl beta-hydroxylase	85.809	5.01	78.819757	16.226913	12	14	12	100.7	99.3	0.986	7.02E-01
Q15392	Delta(24)-sterol reductase	60.062	8.16	31.348657	12.015504	7	8	7	100.7	99.3	0.986	2.08E-01
Q16629	Serine/arginine-rich splicing factor 7	27.35	11.82	48.913114	29.411765	8	18	7	100.7	99.3	0.986	6.66E-01
Q53EZ4	Centrosomal protein of 55 kDa	54.145	7.01	37.036891	12.284483	6	7	6	100.7	99.3	0.986	6.15E-01
Q5JRX3	Presequence protease, mitochondrial	117.338	6.92	105.36526	15.33269	13	16	13	100.7	99.3	0.986	2.36E-01
Q6P1M0	Long-chain fatty acid transport protein 4	72.018	8.47	43.485953	11.664075	6	8	6	100.7	99.3	0.986	2.91E-01

Q6PD62	RNA polymerase-associated protein CTR9 homolog	133.42	6.77	47.300131	7.7578858	10	12	10	100.7	99.3	0.986	1.31E-01
Q86V21	Acetoacetyl-CoA synthetase	75.096	6.24	63.790789	12.202381	8	11	8	100.7	99.3	0.986	8.33E-01
Q8IZH2	5'-3' exoribonuclease 1	193.985	7.21	28.996951	3.4583822	4	5	4	100.7	99.3	0.986	5.53E-01
Q8NCA5	Protein FAM98A	55.366	9.03	42.780294	10.982659	5	7	4	100.7	99.3	0.986	5.03E-01
Q8NFI3	Cytosolic endo-beta-N-acetylglucosaminidase	83.933	6.79	4.3205721	1.4804845	1	1	1	100.7	99.3	0.986	
Q8TDY2	RB1-inducible coiled-coil protein 1	182.975	5.41	17.927326	2.3839398	3	3	2	100.7	99.3	0.986	8.04E-01
Q8WUD1	Ras-related protein Rab-2B	24.199	7.83	82.257296	33.796296	6	15	1	100.7	99.3	0.986	
Q8WXG6	MAP kinase-activating death domain protein	183.188	6.04	6.6597542	1.0928962	1	1	1	100.7	99.3	0.986	
Q8WY36	HMG box transcription factor BBX	105.064	8.79	5.3759978	2.4442083	2	2	2	100.7	99.3	0.986	
Q96C19	EF-hand domain-containing protein D2	26.68	5.2	100.10675	46.25	11	23	8	100.7	99.3	0.986	9.53E-01
Q96FQ6	Protein S100-A16	11.794	6.79	31.88864	37.864078	4	8	4	100.7	99.3	0.986	4.34E-01
Q96K21	Abscission/NoCut checkpoint regulator	51.514	5.73	14.097262	5.0955414	3	4	2	100.7	99.3	0.986	8.09E-02
Q96KP1	Exocyst complex component 2	104	6.9	27.52821	5.952381	6	7	5	100.7	99.3	0.986	7.71E-01
Q99618	Cell division cycle-associated protein 3	28.981	6.43	11.112383	8.5820896	1	1	1	100.7	99.3	0.986	
Q9BQA1	Methylosome protein 50	36.701	5.17	50.776086	24.561404	6	8	6	100.7	99.3	0.986	8.20E-01
Q9BRJ7	Protein syndesmos	23.323	8.91	36.493013	36.018957	7	10	6	100.7	99.3	0.986	1.34E-01
Q9BWE0	Replication initiator 1	63.534	9.98	43.844906	9.3474427	4	6	4	100.7	99.3	0.986	6.65E-01
Q9BYD6	39S ribosomal protein L1, mitochondrial	36.885	8.78	32.6511	12	4	8	4	100.7	99.3	0.986	2.23E-01
Q9GZR2	RNA exonuclease 4	46.643	9.77	33.091456	11.374408	4	6	4	100.7	99.3	0.986	6.30E-01
Q9GZV5	WW domain-containing transcription regulator protein 1	44.073	5.82	9.2195787	6.25	3	4	3	100.7	99.3	0.986	1.33E-01
Q9H0A0	N-acetyltransferase 10	115.657	8.27	199.62485	21.463415	19	34	19	100.7	99.3	0.986	6.13E-01
Q9H0S4	Probable ATP-dependent RNA helicase DDX47	50.615	9.1	65.343624	26.153846	9	13	9	100.7	99.3	0.986	3.58E-01
Q9H4A6	Golgi phosphoprotein 3	33.79	6.44	41.280687	17.785235	4	6	4	100.7	99.3	0.986	1.33E-01
Q9H6D7	HAUS augmin-like complex subunit 4	42.373	5.68	20.587412	7.7134986	2	3	2	100.7	99.3	0.986	8.92E-01
Q9H900	Protein zwilch homolog	67.172	6.27	38.571959	9.3062606	5	6	5	100.7	99.3	0.986	2.81E-01
Q9HC36	rRNA methyltransferase 3, mitochondrial	46.99	8.73	5.9430951	2.8571429	1	1	1	100.7	99.3	0.986	
Q9UJY4	ADP-ribosylation factor-binding protein GGA2	67.108	6.55	11.675359	5.5464927	4	4	3	100.7	99.3	0.986	9.90E-01
Q9UPN4	Centrosomal protein of 131 kDa	122.075	8.69	43.519439	8.033241	8	8	8	100.7	99.3	0.986	4.03E-01
Q9Y371	Endophilin-B1	40.771	6.04	35.973443	17.534247	7	8	7	100.7	99.3	0.986	2.70E-01
Q9Y520	Protein PRRC2C	316.718	9.13	126.38949	8.9779006	22	30	21	100.7	99.3	0.986	7.11E-01
A3KN83	Protein strawberry notch homolog 1	154.216	7.88	50.123477	5.0251256	6	8	6	100.6	99.4	0.988	8.11E-01
O15020	Spectrin beta chain, non-erythrocytic 2	271.157	6.11	76.422945	6.1506276	15	18	10	100.6	99.4	0.988	4.66E-01
O15439	Multidrug resistance-associated protein 4	149.432	8.19	6.6884237	1.2830189	2	2	2	100.6	99.4	0.988	
O43252	Bifunctional 3'-phosphoadenosine 5'-phosphosulfate synthase 1	70.788	6.86	77.21972	24.358974	13	16	10	100.6	99.4	0.988	3.81E-01
O43432	Eukaryotic translation initiation factor 4 gamma 3	176.542	5.38	55.483556	5.6782334	9	16	4	100.6	99.4	0.988	
O43447	Peptidyl-prolyl cis-trans isomerase H	19.196	8.07	41.751933	29.943503	6	13	5	100.6	99.4	0.988	4.73E-01
O60443	Non-syndromic hearing impairment protein 5	54.52	5.17	69.427705	16.330645	7	10	7	100.6	99.4	0.988	7.49E-01
O60825	6-phosphofructo-2-kinase/fructose-2,6-bisphosphatase 2	58.44	8.38	45.416424	14.653465	9	9	9	100.6	99.4	0.988	7.33E-01
O75521	Enoyl-CoA delta isomerase 2, mitochondrial	43.557	9	79.030787	16.497462	7	10	7	100.6	99.4	0.988	5.16E-01
O75718	Cartilage-associated protein	46.532	5.73	42.251867	21.197007	8	9	8	100.6	99.4	0.988	6.24E-01
O95429	BAG family molecular chaperone regulator 4	49.563	5.12	15.598681	9.190372	4	5	3	100.6	99.4	0.988	8.01E-01
O95714	E3 ubiquitin-protein ligase HERC2	526.895	6.28	30.5485	1.6549441	8	9	7	100.6	99.4	0.988	8.28E-01
O96013	Serine/threonine-protein kinase PAK 4	64.032	9.73	13.672672	4.7377327	3	4	3	100.6	99.4	0.988	9.00E-01
P05114	Non-histone chromosomal protein HMG-14	10.653	9.6	54.10063	37	4	12	4	100.6	99.4	0.988	1.54E-01
P06737	Glycogen phosphorylase, liver form	97.087	7.17	144.86619	30.342385	25	31	23	100.6	99.4	0.988	3.96E-01
P07954	Fumarate hydratase, mitochondrial	54.602	8.76	243.37484	42.54902	18	54	18	100.6	99.4	0.988	8.29E-01
P11021	78 kDa glucose-regulated protein	72.288	5.16	461.12657	53.058104	35	183	32	100.6	99.4	0.988	4.85E-01

P11413	Glucose-6-phosphate 1-dehydrogenase	59.219	6.84	215.17502	41.747573	19	55	19	100.6	99.4	0.988	1.87E-01
P12429	Annexin A3	36.353	5.92	133.74949	37.770898	12	29	12	100.6	99.4	0.988	2.44E-01
P13073	Cytochrome c oxidase subunit 4 isoform 1, mitochondrial	19.564	9.51	73.524731	36.094675	7	22	7	100.6	99.4	0.988	5.27E-01
P14618	Pyruvate kinase PKM	57.9	7.84	632.28731	69.491525	39	220	39	100.6	99.4	0.988	1.56E-01
P18074	TFIIH basal transcription factor complex helicase XPD subunit	86.854	7.15	21.971801	7.6315789	7	7	7	100.6	99.4	0.988	3.19E-02
P20618	Proteasome subunit beta type-1	26.472	8.13	80.58592	34.439834	7	24	7	100.6	99.4	0.988	1.72E-01
P23284	Peptidyl-prolyl cis-trans isomerase B	23.728	9.41	99.665333	43.981481	12	26	10	100.6	99.4	0.988	3.24E-01
P25705	ATP synthase subunit alpha, mitochondrial	59.714	9.13	248.40245	46.835443	24	74	23	100.6	99.4	0.988	4.92E-01
P26440	Isovaleryl-CoA dehydrogenase, mitochondrial	46.29	8.19	46.17658	21.513002	9	9	9	100.6	99.4	0.988	5.88E-01
P27105	Erythrocyte band 7 integral membrane protein	31.711	7.88	104.37955	35.416667	8	19	8	100.6	99.4	0.988	3.71E-02
P30086	Phosphatidylethanolamine-binding protein 1	21.044	7.53	110.28304	49.73262	8	25	8	100.6	99.4	0.988	9.46E-01
P35241	Radixin	68.521	6.37	260.20268	40.308748	32	83	16	100.6	99.4	0.988	6.25E-01
P40121	Macrophage-capping protein	38.474	6.19	98.48071	23.563218	7	18	7	100.6	99.4	0.988	2.49E-01
P49773	Histidine triad nucleotide-binding protein 1	13.793	6.95	18.825886	29.365079	5	8	5	100.6	99.4	0.988	1.90E-01
P53396	ATP-citrate synthase	120.762	7.33	406.99941	42.143506	42	97	42	100.6	99.4	0.988	1.80E-01
P55263	Adenosine kinase	40.52	6.7	58.125798	22.375691	8	12	8	100.6	99.4	0.988	4.40E-01
P61081	NEDD8-conjugating enzyme Ubc12	20.887	7.69	54.356831	45.901639	9	13	9	100.6	99.4	0.988	8.33E-01
P62316	Small nuclear ribonucleoprotein Sm D2	13.518	9.91	101.74311	61.864407	8	27	8	100.6	99.4	0.988	3.20E-02
P82675	28S ribosomal protein S5, mitochondrial	47.976	9.92	43.897948	20.697674	9	10	9	100.6	99.4	0.988	5.99E-01
P82909	28S ribosomal protein S36, mitochondrial	11.459	9.99	17.408421	12.621359	2	4	2	100.6	99.4	0.988	3.93E-01
Q00535	Cyclin-dependent-like kinase 5	33.283	7.66	32.299043	20.205479	6	9	5	100.6	99.4	0.988	9.60E-02
Q00536	Cyclin-dependent kinase 16	55.681	7.62	5.520922	4.0322581	3	3	1	100.6	99.4	0.988	
Q01658	Protein Dr1	19.432	4.75	23.939719	15.909091	3	6	3	100.6	99.4	0.988	9.11E-01
Q07955	Serine/arginine-rich splicing factor 1	27.728	10.36	112.66801	41.129032	12	35	12	100.6	99.4	0.988	9.89E-01
Q13242	Serine/arginine-rich splicing factor 9	25.526	8.65	47.606648	38.461538	9	12	9	100.6	99.4	0.988	8.80E-01
Q13308	Inactive tyrosine-protein kinase 7	118.317	7.09	28.951499	5.4205607	5	6	5	100.6	99.4	0.988	6.81E-01
Q15393	Splicing factor 3B subunit 3	135.492	5.26	216.39002	23.336072	23	49	22	100.6	99.4	0.988	8.60E-01
Q16181	Septin-7	50.648	8.63	96.064045	32.036613	14	24	13	100.6	99.4	0.988	7.51E-02
Q2TAL8	Glutamine-rich protein 1	86.382	5.87	19.6943	5.6701031	5	5	5	100.6	99.4	0.988	9.83E-01
Q5VTR2	E3 ubiquitin-protein ligase BRE1A	113.592	5.94	114.80735	14.25641	13	19	11	100.6	99.4	0.988	3.04E-01
Q6IAN0	Dehydrogenase/reductase SDR family member 7B	35.097	9.55	7.4004444	4.6153846	1	2	1	100.6	99.4	0.988	
Q6P1M3	Lethal(2) giant larvae protein homolog 2	113.377	7.52	23.174735	4.6078431	4	5	4	100.6	99.4	0.988	4.97E-01
Q6P2C8	Mediator of RNA polymerase II transcription subunit 27	35.409	9.31	5.4761331	5.1446945	2	2	2	100.6	99.4	0.988	
Q70J99	Protein unc-13 homolog D	123.205	6.65	86.227746	13.577982	12	18	12	100.6	99.4	0.988	2.62E-01
Q7Z2K6	Endoplasmic reticulum metallopeptidase 1	100.167	7.52	21.455028	3.7610619	3	4	3	100.6	99.4	0.988	9.99E-02
Q7Z6Z7	E3 ubiquitin-protein ligase HUWE1	481.589	5.22	289.94198	11.911294	43	57	43	100.6	99.4	0.988	1.29E-01
Q86VP6	Cullin-associated NEDD8-dissociated protein 1	136.289	5.78	229.52074	21.788618	24	54	24	100.6	99.4	0.988	1.79E-01
Q8N6M0	OTU domain-containing protein 6B	33.791	6.05	34.278032	17.064846	4	5	4	100.6	99.4	0.988	4.86E-01
Q8NFH3	Nucleoporin Nup43	42.124	5.63	45.488526	14.473684	4	7	4	100.6	99.4	0.988	7.23E-01
Q8NFQ8	Torsin-1A-interacting protein 2	51.232	4.96	15.44833	6.3829787	3	3	3	100.6	99.4	0.988	8.76E-01
Q8TAG9	Exocyst complex component 6	93.663	6.2	4.0939345	1.2437811	1	1	1	100.6	99.4	0.988	
Q96AT1	Uncharacterized protein KIAA1143	17.455	6.11	5.3515424	7.1428571	1	1	1	100.6	99.4	0.988	
Q96EK5	KIF1-binding protein	71.768	5.49	42.640436	11.433172	6	8	6	100.6	99.4	0.988	2.83E-01
Q96T23	Remodeling and spacing factor 1	163.72	5.01	30.333107	3.8167939	5	6	5	100.6	99.4	0.988	3.29E-01
Q99538	Legumain	49.379	6.55	17.59385	3.926097	1	2	1	100.6	99.4	0.988	
Q9BSL1	Ubiquitin-associated domain-containing protein 1	45.31	4.92	46.306748	15.308642	5	8	5	100.6	99.4	0.988	4.43E-01
Q9BV38	WD repeat-containing protein 18	47.375	6.7	50.407618	10.87963	4	7	4	100.6	99.4	0.988	3.49E-01

Q9BVK6	Transmembrane emp24 domain-containing protein 9	27.26	8.02	34.817228	20.425532	5	8	2	100.6	99.4	0.988	
Q9BWF3	RNA-binding protein 4	40.289	7.08	76.964249	26.648352	9	19	1	100.6	99.4	0.988	
Q9H1B7	Interferon regulatory factor 2-binding protein-like	82.607	8.24	23.109746	6.5326633	5	7	3	100.6	99.4	0.988	7.22E-01
Q9H3P7	Golgi resident protein GCP60	60.556	5.06	60.890015	22.916667	7	8	7	100.6	99.4	0.988	9.23E-02
Q9H9J2	39S ribosomal protein L44, mitochondrial	37.512	8.4	44.936136	19.277108	5	8	5	100.6	99.4	0.988	8.86E-01
Q9NUL3	Double-stranded RNA-binding protein Staufen homolog 2	62.601	9.61	24.52914	6.3157895	4	5	2	100.6	99.4	0.988	9.15E-01
Q9NZQ3	NCK-interacting protein with SH3 domain	78.91	6.38	28.41374	8.5872576	6	6	6	100.6	99.4	0.988	7.81E-01
Q9UHD8	Septin-9	65.361	8.97	122.67518	31.228669	17	29	17	100.6	99.4	0.988	3.06E-01
Q9UI10	Translation initiation factor eIF-2B subunit delta	57.521	9.38	76.138063	22.753346	9	10	9	100.6	99.4	0.988	3.26E-01
Q9Y5S2	Serine/threonine-protein kinase MRCK beta	194.193	6.37	57.135672	5.4938632	9	10	9	100.6	99.4	0.988	3.06E-01
O60907	F-box-like/WD repeat-containing protein TBL1X	62.456	6.55	38.356919	11.091854	6	7	1	100.5	99.5	0.99	
O75955	Flotillin-1	47.326	7.49	62.823919	27.63466	9	10	9	100.5	99.5	0.99	6.30E-01
O94973	AP-2 complex subunit alpha-2	103.895	6.96	56.839041	11.182109	9	11	8	100.5	99.5	0.99	2.80E-01
O95782	AP-2 complex subunit alpha-1	107.478	7.03	151.51444	17.604913	15	34	14	100.5	99.5	0.99	2.38E-01
P00374	Dihydrofolate reductase	21.439	7.42	72.464493	53.475936	10	27	10	100.5	99.5	0.99	9.14E-02
P00492	Hypoxanthine-guanine phosphoribosyltransferase	24.564	6.68	79.642157	40.366972	7	20	7	100.5	99.5	0.99	7.90E-01
P05423	DNA-directed RNA polymerase III subunit RPC4	44.368	6.98	6.2666422	3.5175879	1	1	1	100.5	99.5	0.99	
P05787	Keratin, type II cytoskeletal 8	53.671	5.59	471.81823	60.248447	37	173	32	100.5	99.5	0.99	4.02E-01
P07311	Acylphosphatase-1	11.254	9.31	7.4522253	13.131313	1	1	1	100.5	99.5	0.99	
P14859	POU domain, class 2, transcription factor 1	76.425	6.81	12.896926	5.3835801	3	3	3	100.5	99.5	0.99	7.08E-01
P22102	Trifunctional purine biosynthetic protein adenosine-3	107.699	6.7	262.84023	28.712871	25	51	25	100.5	99.5	0.99	5.41E-01
P27144	Adenylate kinase 4, mitochondrial	25.252	8.4	44.691217	34.977578	5	9	5	100.5	99.5	0.99	2.56E-01
P27708	CAD protein	242.829	6.46	318.86387	21.348315	40	68	36	100.5	99.5	0.99	9.20E-01
P36915	Guanine nucleotide-binding protein-like 1	68.619	5.8	46.344638	17.957166	9	9	9	100.5	99.5	0.99	5.34E-01
P41236	Protein phosphatase inhibitor 2	23.001	4.74	79.001206	17.560976	5	16	5	100.5	99.5	0.99	8.39E-01
P42356	Phosphatidylinositol 4-kinase alpha	231.17	6.87	18.717095	2.6418787	4	4	4	100.5	99.5	0.99	1.28E-01
P52294	Importin subunit alpha-5	60.184	5.01	82.125544	18.587361	10	14	6	100.5	99.5	0.99	7.13E-01
P60468	Protein transport protein Sec61 subunit beta	9.968	11.56	23.4895	44.791667	4	7	4	100.5	99.5	0.99	6.92E-01
P60709	Actin, cytoplasmic 1	41.71	5.48	526.04418	60	21	409	8	100.5	99.5	0.99	3.65E-01
P61224	Ras-related protein Rap-1b	20.812	5.78	80.058549	41.304348	7	18	3	100.5	99.5	0.99	1.80E-01
P61599	N-alpha-acetyltransferase 20	20.355	5.03	14.999531	11.235955	2	3	2	100.5	99.5	0.99	4.09E-01
P82664	28S ribosomal protein S10, mitochondrial	22.985	8	18.695207	18.40796	3	3	3	100.5	99.5	0.99	7.97E-01
P82979	SAP domain-containing ribonucleoprotein	23.656	6.42	46.653319	31.904762	6	9	6	100.5	99.5	0.99	9.71E-01
Q06203	Amidophosphoribosyltransferase	57.362	6.76	60.475715	22.05029	9	10	9	100.5	99.5	0.99	7.16E-01
Q13162	Peroxiredoxin-4	30.521	6.29	75.259517	39.483395	8	21	6	100.5	99.5	0.99	8.76E-01
Q13510	Acid ceramidase	44.631	7.62	29.203862	12.911392	5	6	5	100.5	99.5	0.99	2.14E-01
Q14141	Septin-6	49.685	6.67	83.243902	28.571429	11	18	3	100.5	99.5	0.99	5.75E-01
Q14789	Golgin subfamily B member 1	375.79	5	358.6844	19.208346	51	64	48	100.5	99.5	0.99	2.17E-01
Q15054	DNA polymerase delta subunit 3	51.368	9.35	54.045298	15.236052	6	8	6	100.5	99.5	0.99	8.03E-01
Q15751	Probable E3 ubiquitin-protein ligase HERC1	531.891	6.04	21.457551	0.9668792	4	5	4	100.5	99.5	0.99	7.69E-01
Q16576	Histone-binding protein RBBP7	47.79	5.05	61.661373	26.117647	10	21	4	100.5	99.5	0.99	7.49E-01
Q16836	Hydroxyacyl-coenzyme A dehydrogenase, mitochondrial	34.272	8.85	54.048487	35.031847	9	16	9	100.5	99.5	0.99	1.58E-01
Q6ZRS2	Helicase SRCAP	343.343	5.96	18.435463	0.9597523	3	3	3	100.5	99.5	0.99	4.09E-01
Q6ZVM7	TOM1-like protein 2	55.522	4.79	6.8890134	3.3530572	2	2	2	100.5	99.5	0.99	
Q86WJ1	Chromodomain-helicase-DNA-binding protein 1-like	100.921	6.9	6.5221555	1.8952062	1	1	1	100.5	99.5	0.99	
Q86WR0	Coiled-coil domain-containing protein 25	24.463	6.8	24.568716	14.903846	3	5	3	100.5	99.5	0.99	6.69E-01
Q86YQ8	Copine-8	63.068	5.96	29.725196	9.3971631	5	6	4	100.5	99.5	0.99	1.76E-01

Q8N2Z9	Centromere protein S	15.883	6.05	3.793714	5.0724638	1	1	1	100.5	99.5	0.99	
Q8NBT2	Kinetochore protein Spc24	22.464	4.7	46.965489	35.025381	5	8	4	100.5	99.5	0.99	7.30E-01
Q8NBX0	Saccharopine dehydrogenase-like oxidoreductase	47.121	9.14	42.792443	14.685315	5	9	5	100.5	99.5	0.99	8.96E-01
Q8NEJ9	Neuroguidin	35.872	9.57	11.65085	7.6190476	2	2	2	100.5	99.5	0.99	
Q8WUX9	Charged multivesicular body protein 7	50.879	5.35	23.255861	7.7262693	3	4	3	100.5	99.5	0.99	
Q8WVC0	RNA polymerase-associated protein LEO1	75.359	4.51	21.012702	3.003003	2	4	2	100.5	99.5	0.99	9.45E-01
Q8WXE0	Caskin-2	126.705	7.09	7.5356954	2.3294509	2	2	2	100.5	99.5	0.99	1.86E-01
Q92990	Glomulin	68.165	5.33	31.519379	7.0707071	4	5	4	100.5	99.5	0.99	6.06E-01
Q96FV2	Secernin-2	46.567	5.67	7.8016008	5.1764706	2	2	2	100.5	99.5	0.99	
Q96GM5	ted matrix-associated actin-dependent regulator of chromatin subfamil	58.196	9.25	38.763891	16.116505	6	7	5	100.5	99.5	0.99	9.63E-01
Q96KG9	N-terminal kinase-like protein	89.575	6.3	48.519995	8.539604	6	10	6	100.5	99.5	0.99	4.57E-01
Q96P70	Importin-9	115.889	4.81	148.97693	14.697406	13	22	13	100.5	99.5	0.99	9.20E-01
Q99714	3-hydroxyacyl-CoA dehydrogenase type-2	26.906	7.78	238.57303	61.302682	11	35	11	100.5	99.5	0.99	1.25E-01
Q9BRK5	45 kDa calcium-binding protein	41.78	4.86	43.52036	14.364641	4	6	4	100.5	99.5	0.99	9.33E-01
Q9BRP8	Partner of Y14 and mago	22.642	9.45	51.46319	44.607843	7	7	7	100.5	99.5	0.99	4.09E-01
Q9BUJ2	Heterogeneous nuclear ribonucleoprotein U-like protein 1	95.679	6.92	108.33671	22.429907	18	29	17	100.5	99.5	0.99	6.83E-01
Q9BWD1	Acetyl-CoA acetyltransferase, cytosolic	41.324	6.92	28.999954	17.632242	5	6	5	100.5	99.5	0.99	1.21E-01
Q9NY93	Probable ATP-dependent RNA helicase DDX56	61.551	9.26	40.562384	11.517367	6	9	6	100.5	99.5	0.99	1.08E-01
Q9P0V9	Septin-10	52.56	6.8	60.520675	19.823789	8	13	5	100.5	99.5	0.99	5.12E-01
Q9UIG0	Tyrosine-protein kinase BAZ1B	170.796	8.48	104.27381	10.856372	15	18	15	100.5	99.5	0.99	5.17E-01
Q9UJA5	ɌNA (adenine(58)-N(1))-methyltransferase non-catalytic subunit TRM	55.764	7.55	2.7432823	1.6096579	1	1	1	100.5	99.5	0.99	
Q9UMY1	Nucleolar protein 7	29.409	9.67	9.2859524	10.116732	3	3	3	100.5	99.5	0.99	
Q9UMY4	Sorting nexin-12	19.718	7.87	28.481767	27.906977	5	12	4	100.5	99.5	0.99	3.34E-02
Q9UP83	Conserved oligomeric Golgi complex subunit 5	92.685	6.6	31.917107	7.0321812	5	7	5	100.5	99.5	0.99	2.33E-01
Q9UQ13	Leucine-rich repeat protein SHOC-2	64.847	8.46	10.719021	3.6082474	2	2	2	100.5	99.5	0.99	6.36E-01
Q9UQE7	Structural maintenance of chromosomes protein 3	141.454	7.18	342.54139	36.236647	38	67	37	100.5	99.5	0.99	4.17E-01
Q9Y265	RuvB-like 1	50.196	6.42	188.75909	52.850877	17	29	17	100.5	99.5	0.99	3.12E-01
Q9Y2V2	Calcium-regulated heat stable protein 1	15.882	8.21	36.458805	16.326531	2	5	2	100.5	99.5	0.99	2.41E-01
Q9Y3B2	Exosome complex component CSL4	21.438	8.24	9.2873875	12.307692	2	2	2	100.5	99.5	0.99	8.93E-01
Q9Y3Z3	Deoxynucleoside triphosphate triphosphohydrolase SAMHD1	72.155	7.14	25.780996	8.3067093	4	4	4	100.5	99.5	0.99	8.99E-01
Q9Y597	BTB/POZ domain-containing protein KCTD3	88.929	7.03	4.5368973	2.4539877	2	2	2	100.5	99.5	0.99	6.53E-01
Q9Y5K5	Ubiquitin carboxyl-terminal hydrolase isozyme L5	37.583	5.33	96.047766	36.778116	9	21	9	100.5	99.5	0.99	8.58E-01
Q9Y5Y2	Cytosolic Fe-S cluster assembly factor NUBP2	28.807	5.83	21.53076	18.450185	3	3	3	100.5	99.5	0.99	2.65E-01
O00115	Deoxyribonuclease-2-alpha	39.556	8.05	6.5663902	2.7777778	1	1	1	100.4	99.6	0.992	
O00139	Kinesin-like protein KIF2A	79.905	6.68	46.511125	12.464589	9	10	8	100.4	99.6	0.992	5.07E-01
O00303	Eukaryotic translation initiation factor 3 subunit F	37.54	5.45	105.43421	28.571429	9	23	9	100.4	99.6	0.992	6.25E-01
O14980	Exportin-1	123.306	6.06	219.64108	21.195145	19	47	19	100.4	99.6	0.992	8.74E-01
O75027	ATP-binding cassette sub-family B member 7, mitochondrial	82.589	9.33	59.283309	12.5	8	13	8	100.4	99.6	0.992	9.00E-01
O75368	SH3 domain-binding glutamic acid-rich-like protein	12.766	5.25	30.437941	20.175439	3	6	3	100.4	99.6	0.992	7.02E-01
O75821	Eukaryotic translation initiation factor 3 subunit G	35.589	6.13	87.235774	30.3125	10	18	10	100.4	99.6	0.992	8.98E-01
O76021	Ribosomal L1 domain-containing protein 1	54.939	10.13	119.97821	31.020408	15	26	15	100.4	99.6	0.992	1.00E+00
O95149	Snurportin-1	41.116	6.64	3.4678829	2.7777778	1	1	1	100.4	99.6	0.992	
O95347	Structural maintenance of chromosomes protein 2	135.572	8.43	222.5517	23.391813	29	46	29	100.4	99.6	0.992	4.56E-01
O95425	Supervillin	247.593	6.98	20.803076	2.4841915	4	4	4	100.4	99.6	0.992	6.84E-01
P01130	Low-density lipoprotein receptor	95.314	5.05	6.5756084	1.9767442	1	1	1	100.4	99.6	0.992	
P16333	Cytoplasmic protein NCK1	42.837	6.47	61.597432	23.342175	9	14	9	100.4	99.6	0.992	8.28E-01
P17405	Sphingomyelin phosphodiesterase	69.707	7.28	5.8153795	2.8616852	2	2	1	100.4	99.6	0.992	

P18583	Protein SON	263.664	5.64	102.76327	8.8623248	17	19	17	100.4	99.6	0.992	7.68E-01
P23526	Adenosylhomocysteinase	47.685	6.34	193.26161	34.722222	15	44	13	100.4	99.6	0.992	6.26E-01
P26599	Polypyrimidine tract-binding protein 1	57.186	9.17	58.697071	14.500942	8	22	5	100.4	99.6	0.992	5.55E-01
P35556	Fibrillin-2	314.558	4.86	71.144946	5.2197802	13	13	12	100.4	99.6	0.992	4.57E-01
P35998	26S protease regulatory subunit 7	48.603	5.95	274.33335	62.586605	24	56	23	100.4	99.6	0.992	6.82E-01
P36873	Serine/threonine-protein phosphatase PP1-gamma catalytic subunit	36.96	6.54	122.83847	37.770898	10	32	2	100.4	99.6	0.992	8.94E-01
P39656	chyl-diphosphooligosaccharide--protein glycosyltransferase 48 kDa su	50.769	6.55	41.170854	16.885965	8	13	8	100.4	99.6	0.992	7.67E-01
P40937	Replication factor C subunit 5	38.472	7.2	68.349687	30.588235	9	14	9	100.4	99.6	0.992	4.55E-01
P49458	Signal recognition particle 9 kDa protein	10.105	7.97	46.526477	62.790698	6	22	6	100.4	99.6	0.992	5.81E-01
P50613	Cyclin-dependent kinase 7	39.014	8.47	28.07178	12.427746	4	5	4	100.4	99.6	0.992	2.45E-01
P50995	Annexin A11	54.355	7.65	116.70791	26.138614	12	24	12	100.4	99.6	0.992	4.93E-01
P51398	28S ribosomal protein S29, mitochondrial	45.538	8.88	73.998408	25.879397	8	13	8	100.4	99.6	0.992	4.88E-01
P55036	26S proteasome non-ATPase regulatory subunit 4	40.711	4.79	142.87569	44.03183	14	24	14	100.4	99.6	0.992	1.75E-01
P55786	Puromycin-sensitive aminopeptidase	103.211	5.72	142.2908	25.897715	21	28	21	100.4	99.6	0.992	1.26E-01
P59998	Actin-related protein 2/3 complex subunit 4	19.654	8.43	31.776331	27.97619	5	11	5	100.4	99.6	0.992	4.50E-01
P62140	Serine/threonine-protein phosphatase PP1-beta catalytic subunit	37.163	6.19	125.69908	34.556575	9	33	2	100.4	99.6	0.992	4.63E-01
P80303	Nucleobindin-2	50.164	5.12	77.514712	24.047619	9	15	9	100.4	99.6	0.992	1.55E-01
P82921	28S ribosomal protein S21, mitochondrial	10.734	10.21	24.824849	29.885057	2	4	2	100.4	99.6	0.992	5.20E-04
Q01968	Inositol polyphosphate 5-phosphatase OCRL-1	104.138	6.55	16.981897	3.5516093	3	3	3	100.4	99.6	0.992	6.61E-01
Q06210	Glutamine--fructose-6-phosphate aminotransferase [isomerizing] 1	78.756	7.11	194.87335	32.188841	19	37	17	100.4	99.6	0.992	2.27E-01
Q12765	Secernin-1	46.353	4.75	69.672134	22.222222	9	13	9	100.4	99.6	0.992	1.69E-01
Q12888	Tumor suppressor p53-binding protein 1	213.443	4.7	154.80924	13.083164	20	28	20	100.4	99.6	0.992	1.06E-01
Q12931	Heat shock protein 75 kDa, mitochondrial	80.06	8.21	293.64823	37.5	27	76	26	100.4	99.6	0.992	2.54E-01
Q13435	Splicing factor 3B subunit 2	100.165	5.67	170.40243	25.810056	21	36	21	100.4	99.6	0.992	6.29E-03
Q14240	Eukaryotic initiation factor 4A-II	46.373	5.48	142.45215	33.906634	15	53	5	100.4	99.6	0.992	2.50E-01
Q14676	Mediator of DNA damage checkpoint protein 1	226.529	5.47	21.314829	3.015797	6	6	6	100.4	99.6	0.992	3.67E-01
Q15020	Squamous cell carcinoma antigen recognized by T-cells 3	109.865	5.57	72.904112	14.018692	13	13	12	100.4	99.6	0.992	5.28E-01
Q15386	Ubiquitin-protein ligase E3C	123.844	6.71	17.130527	4.0627886	5	5	5	100.4	99.6	0.992	4.56E-01
Q16831	Uridine phosphorylase 1	33.912	7.88	8.4906856	6.7741935	2	2	2	100.4	99.6	0.992	
Q5JSH3	WD repeat-containing protein 44	101.304	5.45	318.6	41.073384	35	61	35	100.4	99.6	0.992	5.22E-01
Q6BDS2	UHRF1-binding protein 1	159.385	6.14	5.9048347	1.0416667	2	2	2	100.4	99.6	0.992	
Q7KZ85	Transcription elongation factor SPT6	198.949	4.91	58.70174	6.5469293	9	11	9	100.4	99.6	0.992	5.39E-01
Q7L5Y9	Macrophage erythroblast attacher	45.258	8.69	10.275203	5.3030303	2	2	2	100.4	99.6	0.992	6.55E-01
Q7Z6M1	Rab9 effector protein with kelch motifs	40.539	6.25	14.854662	9.1397849	3	3	3	100.4	99.6	0.992	4.63E-01
Q86U42	Polyadenylate-binding protein 2	32.729	5.06	30.661035	11.764706	4	11	4	100.4	99.6	0.992	6.65E-01
Q86UP2	Kinectin	156.179	5.64	466.18904	38.761975	46	77	45	100.4	99.6	0.992	5.34E-01
Q86X10	Ral GTPase-activating protein subunit beta	166.692	6.79	14.072823	2.5435074	3	3	3	100.4	99.6	0.992	
Q8N183	Mimitin, mitochondrial	19.844	8.97	52.157042	48.52071	7	9	7	100.4	99.6	0.992	7.41E-01
Q92796	Disks large homolog 3	90.258	7.03	17.7643	4.7735618	3	3	2	100.4	99.6	0.992	
Q92888	Rho guanine nucleotide exchange factor 1	102.371	5.66	128.31375	21.820175	17	23	17	100.4	99.6	0.992	1.23E-01
Q96B97	SH3 domain-containing kinase-binding protein 1	73.082	6.62	14.722799	4.6616541	2	2	2	100.4	99.6	0.992	
Q96CN4	EVI5-like protein	91.318	5.34	14.090486	3.02267	2	2	2	100.4	99.6	0.992	
Q96CX2	BTB/POZ domain-containing protein KCTD12	35.679	5.64	54.52512	25.538462	6	13	6	100.4	99.6	0.992	3.82E-02
Q96GG9	DCN1-like protein 1	30.105	5.34	48.866757	28.185328	7	10	7	100.4	99.6	0.992	8.37E-01
Q96HS1	Serine/threonine-protein phosphatase PGAM5, mitochondrial	31.985	8.68	55.099668	32.525952	8	12	8	100.4	99.6	0.992	2.45E-01
Q96JM3	Chromosome alignment-maintaining phosphoprotein 1	89.043	8.44	57.435968	14.162562	11	13	11	100.4	99.6	0.992	3.98E-01
Q96QK1	Vacuolar protein sorting-associated protein 35	91.649	5.49	164.00202	26.633166	19	40	19	100.4	99.6	0.992	5.27E-01

Q96RN5	Mediator of RNA polymerase II transcription subunit 15	86.699	9.42	24.833635	4.9492386	3	4	3	100.4	99.6	0.992	
Q96SW2	Protein cereblon	50.513	5.63	4.6272721	2.2624434	1	1	1	100.4	99.6	0.992	
Q99436	Proteasome subunit beta type-7	29.946	7.68	48.095844	22.743682	6	14	6	100.4	99.6	0.992	7.90E-01
Q99757	Thioredoxin, mitochondrial	18.372	8.29	7.2924298	9.0361446	1	1	1	100.4	99.6	0.992	
Q9BQE5	Apolipoprotein L2	37.069	6.74	15.640822	10.682493	4	5	4	100.4	99.6	0.992	5.52E-01
Q9BQI0	Allograft inflammatory factor 1-like	17.057	7.2	3.67788	8	2	2	2	100.4	99.6	0.992	
Q9BTY7	Protein HGH1 homolog	42.103	4.81	35.742231	23.076923	7	9	7	100.4	99.6	0.992	7.20E-01
Q9BUE6	Iron-sulfur cluster assembly 1 homolog, mitochondrial	14.17	9.07	9.1429692	12.403101	1	1	1	100.4	99.6	0.992	
Q9BUH6	Protein PAXX	21.626	5.48	9.6942789	15.196078	3	3	3	100.4	99.6	0.992	
Q9BXB4	Oxysterol-binding protein-related protein 11	83.591	7.06	21.721927	4.8192771	3	4	2	100.4	99.6	0.992	5.28E-01
Q9H936	Mitochondrial glutamate carrier 1	34.448	9.29	15.549444	9.5975232	3	4	3	100.4	99.6	0.992	3.41E-01
Q9NRY5	Protein FAM114A2	55.434	4.88	16.355939	7.3267327	3	3	3	100.4	99.6	0.992	6.80E-01
Q9NVP1	ATP-dependent RNA helicase DDX18	75.359	9.5	129.07465	21.044776	13	23	13	100.4	99.6	0.992	5.55E-01
Q9NX63	MICOS complex subunit MIC19	26.136	8.28	53.592336	35.682819	8	11	8	100.4	99.6	0.992	3.50E-01
Q9UJW0	Dynactin subunit 4	52.304	7.34	8.1351931	4.3478261	2	2	2	100.4	99.6	0.992	
Q9Y5Q0	Fatty acid desaturase 3	51.112	7.77	11.355183	9.6629213	3	3	3	100.4	99.6	0.992	2.65E-01
Q9Y6E0	Serine/threonine-protein kinase 24	49.277	5.69	57.673052	16.252822	6	10	3	100.4	99.6	0.992	9.60E-01
O00592	Podocalyxin	58.599	5.49	17.226513	5.1971326	3	6	3	100.3	99.7	0.994	6.62E-01
O60925	Prefoldin subunit 1	14.202	6.81	23.330444	23.770492	3	5	3	100.3	99.7	0.994	9.51E-01
O75063	Glycosaminoglycan xylosylkinase	46.403	6.87	17.076898	7.5794621	3	4	3	100.3	99.7	0.994	3.30E-01
O75083	WD repeat-containing protein 1	66.152	6.65	155.72534	33.663366	15	38	15	100.3	99.7	0.994	1.39E-01
O75410	Transforming acidic coiled-coil-containing protein 1	87.74	4.88	33.563293	9.689441	6	6	4	100.3	99.7	0.994	6.32E-01
O95619	YEATS domain-containing protein 4	26.483	8.41	11.700355	3.9647577	1	2	1	100.3	99.7	0.994	
O95639	Cleavage and polyadenylation specificity factor subunit 4	30.235	8.31	8.6738293	7.8066914	2	3	2	100.3	99.7	0.994	9.60E-01
O96005	Cleft lip and palate transmembrane protein 1	76.048	6.3	31.438628	5.6801196	3	6	3	100.3	99.7	0.994	4.41E-01
P04150	Glucocorticoid receptor	85.605	6.38	53.513514	12.741313	8	9	8	100.3	99.7	0.994	5.34E-01
P07195	L-lactate dehydrogenase B chain	36.615	6.05	207.95534	43.113772	18	92	16	100.3	99.7	0.994	8.92E-01
P08397	Porphobilinogen deaminase	39.306	7.18	41.381926	19.113573	7	10	7	100.3	99.7	0.994	5.24E-01
P0DMV9	Heat shock 70 kDa protein 1B	70.009	5.66	349.47944	48.985959	26	126	15	100.3	99.7	0.994	1.18E-01
P10768	S-formylglutathione hydrolase	31.442	7.02	67.367843	27.659574	7	12	7	100.3	99.7	0.994	4.98E-01
P11117	Lysosomal acid phosphatase	48.313	6.74	12.984429	5.2009456	2	3	2	100.3	99.7	0.994	
P11142	Heat shock cognate 71 kDa protein	70.854	5.52	483.37319	55.108359	33	164	20	100.3	99.7	0.994	8.89E-01
P13667	Protein disulfide-isomerase A4	72.887	5.07	328.21854	46.666667	31	77	31	100.3	99.7	0.994	8.40E-01
P15170	Eukaryotic peptide chain release factor GTP-binding subunit ERF3A	55.72	5.62	90.841056	26.252505	14	26	14	100.3	99.7	0.994	1.99E-01
P27348	14-3-3 protein theta	27.747	4.78	227.48129	61.632653	17	102	10	100.3	99.7	0.994	7.34E-01
P27694	Replication protein A 70 kDa DNA-binding subunit	68.095	7.21	128.87792	26.136364	14	25	14	100.3	99.7	0.994	6.98E-02
P28070	Proteasome subunit beta type-4	29.185	5.97	74.711084	28.409091	5	13	5	100.3	99.7	0.994	1.00E+00
P29083	General transcription factor IIE subunit 1	49.421	4.82	19.169133	9.3394077	4	4	4	100.3	99.7	0.994	2.84E-01
P29400	Collagen alpha-5(IV) chain	160.943	7.62	9.0796366	1.4243323	2	2	2	100.3	99.7	0.994	6.84E-01
P36957	idue succinyltransferase component of 2-oxoglutarate dehydrogenase	48.724	8.95	91.990755	27.593819	10	19	10	100.3	99.7	0.994	3.79E-02
P38432	Coilin	62.57	9.07	18.569847	3.9930556	2	3	2	100.3	99.7	0.994	
P40926	Malate dehydrogenase, mitochondrial	35.481	8.68	244.65618	65.384615	18	68	18	100.3	99.7	0.994	4.57E-01
P46060	Ran GTPase-activating protein 1	63.502	4.68	268.60056	41.567291	21	49	21	100.3	99.7	0.994	1.96E-02
P50336	Protoporphyrinogen oxidase	50.734	8.16	14.265269	5.4507338	3	3	2	100.3	99.7	0.994	9.33E-01
P51784	Ubiquitin carboxyl-terminal hydrolase 11	109.747	5.45	28.888936	4.7767394	4	6	4	100.3	99.7	0.994	9.54E-01
P52657	Transcription initiation factor IIA subunit 2	12.449	6.62	4.914364	14.678899	2	3	2	100.3	99.7	0.994	4.86E-01
P53999	Activated RNA polymerase II transcriptional coactivator p15	14.386	9.6	68.621511	44.88189	6	22	6	100.3	99.7	0.994	6.57E-01

P54578	Ubiquitin carboxyl-terminal hydrolase 14	56.033	5.3	146.02188	30.566802	12	19	12	100.3	99.7	0.994	4.30E-01
P54802	Alpha-N-acetylglucosaminidase	82.214	6.65	4.353236	2.0188425	2	2	2	100.3	99.7	0.994	
P56282	DNA polymerase epsilon subunit 2	59.499	6.35	8.1480693	2.0872865	1	1	1	100.3	99.7	0.994	
P57076	UPF0769 protein C21orf59	33.203	7.44	14.567645	9.6551724	2	2	2	100.3	99.7	0.994	7.07E-01
P60510	Serine/threonine-protein phosphatase 4 catalytic subunit	35.057	5.06	75.253955	36.482085	7	10	6	100.3	99.7	0.994	3.85E-01
P61758	Prefoldin subunit 3	22.643	7.11	90.06239	52.284264	12	23	12	100.3	99.7	0.994	6.52E-01
P61978	Heterogeneous nuclear ribonucleoprotein K	50.944	5.54	151.29226	35.421166	17	85	17	100.3	99.7	0.994	2.43E-01
P62318	Small nuclear ribonucleoprotein Sm D3	13.907	10.32	68.923301	47.619048	4	15	4	100.3	99.7	0.994	1.99E-01
P62937	Peptidyl-prolyl cis-trans isomerase A	18.001	7.81	188.60151	62.424242	14	115	13	100.3	99.7	0.994	8.89E-01
P98175	RNA-binding protein 10	103.469	5.97	44.78122	7.311828	7	13	5	100.3	99.7	0.994	4.16E-01
Q01970	1-phosphatidylinositol 4,5-bisphosphate phosphodiesterase beta-3	138.713	5.9	59.18334	7.3743922	7	8	7	100.3	99.7	0.994	6.32E-01
Q02218	2-oxoglutarate dehydrogenase, mitochondrial	115.861	6.86	197.8092	23.851417	22	39	22	100.3	99.7	0.994	3.74E-01
Q0PNE2	Elongator complex protein 6	29.774	6.84	11.930173	12.781955	3	3	3	100.3	99.7	0.994	2.24E-01
Q12905	Interleukin enhancer-binding factor 2	43.035	5.26	86.92562	34.102564	9	19	9	100.3	99.7	0.994	4.81E-01
Q14980	Nuclear mitotic apparatus protein 1	238.115	5.78	646.84545	36.217494	70	108	70	100.3	99.7	0.994	9.43E-01
Q3YEC7	Rab-like protein 6	79.5	5.22	33.178001	5.7613169	3	4	3	100.3	99.7	0.994	9.47E-01
Q5T2E6	UPF0668 protein C10orf76	78.659	6.6	4.9842212	1.3062409	1	1	1	100.3	99.7	0.994	
Q5T447	E3 ubiquitin-protein ligase HECTD3	97.051	5.64	6.8636245	2.9036005	2	2	2	100.3	99.7	0.994	
Q5VV42	Threonylcarbamoyladenosine tRNA methylthiotransferase	65.07	7.46	14.307329	3.2815199	1	1	1	100.3	99.7	0.994	
Q6UW68	Transmembrane protein 205	21.184	8.62	10.10055	15.873016	2	3	2	100.3	99.7	0.994	
Q70UQ0	Inhibitor of nuclear factor kappa-B kinase-interacting protein	39.285	9.17	65.613828	27.714286	10	15	10	100.3	99.7	0.994	8.08E-01
Q7L266	Isoaspartyl peptidase/L-asparaginase	32.034	6.24	4.0073994	4.5454545	2	2	2	100.3	99.7	0.994	
Q7Z5L9	Interferon regulatory factor 2-binding protein 2	60.987	8.69	49.781937	15.332198	7	9	5	100.3	99.7	0.994	3.18E-01
Q86U90	YrdC domain-containing protein, mitochondrial	29.309	8.57	20.414703	16.129032	4	5	4	100.3	99.7	0.994	4.86E-01
Q96B26	Exosome complex component RRP43	30.02	5.3	12.008744	7.9710145	2	4	2	100.3	99.7	0.994	2.93E-01
Q96K76	Ubiquitin carboxyl-terminal hydrolase 47	157.212	5.08	172.97152	20.436364	23	29	23	100.3	99.7	0.994	4.12E-01
Q96SB4	SRSF protein kinase 1	74.278	6.16	48.539015	12.977099	7	12	6	100.3	99.7	0.994	6.84E-01
Q96SB8	Structural maintenance of chromosomes protein 6	126.246	6.99	7.6798873	2.3831347	3	3	3	100.3	99.7	0.994	1.94E-02
Q99798	Aconitate hydratase, mitochondrial	85.372	7.61	130.16595	25.512821	16	27	16	100.3	99.7	0.994	8.07E-01
Q9BYT8	Neurolysin, mitochondrial	80.6	6.64	82.862823	14.204545	9	13	9	100.3	99.7	0.994	2.78E-01
Q9H7D0	Dedicator of cytokinesis protein 5	215.172	7.96	56.630573	4.7593583	9	11	9	100.3	99.7	0.994	8.33E-01
Q9H832	Ubiquitin-conjugating enzyme E2 Z	38.186	5.62	34.025523	11.864407	4	7	4	100.3	99.7	0.994	4.66E-02
Q9H993	Protein-glutamate O-methyltransferase	51.14	5.76	19.491217	9.9773243	4	4	4	100.3	99.7	0.994	5.82E-01
Q9NTK5	Obg-like ATPase 1	44.715	7.81	92.805673	31.060606	11	19	11	100.3	99.7	0.994	4.36E-01
Q9NVT9	Armadillo repeat-containing protein 1	31.261	5.74	8.9355609	9.2198582	3	3	3	100.3	99.7	0.994	3.06E-01
Q9UHR6	Zinc finger HIT domain-containing protein 2	42.857	5.99	25.467447	7.1960298	2	4	2	100.3	99.7	0.994	9.60E-01
Q9UNH7	Sorting nexin-6	46.62	6.16	92.538701	26.847291	12	18	11	100.3	99.7	0.994	9.25E-01
Q9Y5L0	Transportin-3	104.136	5.57	81.305825	14.626219	12	18	12	100.3	99.7	0.994	6.53E-01
Q9Y5Y0	Feline leukemia virus subgroup C receptor-related protein 1	59.824	5.97	25.760372	10.45045	3	4	3	100.3	99.7	0.994	5.89E-01
Q9Y6W3	Calpain-7	92.594	7.65	15.689024	4.5510455	4	4	4	100.3	99.7	0.994	2.71E-01
O15143	Actin-related protein 2/3 complex subunit 1B	40.923	8.35	40.707302	20.698925	7	11	7	100.2	99.8	0.996	6.92E-01
O15145	Actin-related protein 2/3 complex subunit 3	20.533	8.59	12.606386	12.921348	2	2	2	100.2	99.8	0.996	
O43765	Small glutamine-rich tetratricopeptide repeat-containing protein alpha	34.042	4.87	20.508487	14.376997	4	5	4	100.2	99.8	0.996	6.19E-01
O75964	ATP synthase subunit g, mitochondrial	11.421	9.64	31.172238	36.893204	5	10	5	100.2	99.8	0.996	5.26E-01
O95456	Proteasome assembly chaperone 1	32.833	7.17	34.547384	21.180556	5	9	5	100.2	99.8	0.996	7.57E-01
P00387	NADH-cytochrome b5 reductase 3	34.213	7.59	94.618178	35.215947	8	13	8	100.2	99.8	0.996	5.73E-01
P00568	Adenylate kinase isoenzyme 1	21.621	8.63	53.774358	38.659794	8	16	8	100.2	99.8	0.996	8.46E-01

P02647	Apolipoprotein A-I	30.759	5.76	9.3093137	9.3632959	2	2	2	100.2	99.8	0.996	6.52E-01
P05549	Transcription factor AP-2-alpha	48.032	8.02	6.9666329	4.1189931	2	2	2	100.2	99.8	0.996	
P08670	Vimentin	53.619	5.12	568.55623	67.811159	41	190	37	100.2	99.8	0.996	4.49E-01
P10599	Thioredoxin	11.73	4.92	71.243308	51.428571	6	34	6	100.2	99.8	0.996	5.13E-01
P11498	Pyruvate carboxylase, mitochondrial	129.551	6.84	189.45523	25.382003	21	35	21	100.2	99.8	0.996	4.84E-01
P12235	ADP/ATP translocase 1	33.043	9.76	81.692586	35.234899	12	24	3	100.2	99.8	0.996	4.19E-01
P21926	CD9 antigen	25.399	7.15	7.3608033	7.4561404	2	2	2	100.2	99.8	0.996	
P24539	ATP synthase F(0) complex subunit B1, mitochondrial	28.89	9.36	52.152388	22.65625	6	19	6	100.2	99.8	0.996	5.35E-01
P26038	Moesin	67.778	6.4	430.52709	57.538995	45	120	31	100.2	99.8	0.996	4.38E-01
P26374	Rab proteins geranylgeranyltransferase component A 2	74.024	4.93	30.207759	6.8597561	4	6	4	100.2	99.8	0.996	7.31E-01
P30084	Enoyl-CoA hydratase, mitochondrial	31.367	8.07	136.10071	45.172414	12	28	12	100.2	99.8	0.996	3.88E-01
P34897	Serine hydroxymethyltransferase, mitochondrial	55.958	8.53	164.48408	33.531746	15	33	15	100.2	99.8	0.996	6.07E-01
P38646	Stress-70 protein, mitochondrial	73.635	6.16	327.37335	49.926362	33	95	32	100.2	99.8	0.996	1.30E-01
P40925	Malate dehydrogenase, cytoplasmic	36.403	7.36	107.19207	46.706587	16	35	16	100.2	99.8	0.996	3.51E-01
P41240	Tyrosine-protein kinase CSK	50.672	7.06	22.187904	8	4	4	4	100.2	99.8	0.996	7.12E-01
P42126	Enoyl-CoA delta isomerase 1, mitochondrial	32.795	8.54	68.673408	17.880795	5	15	5	100.2	99.8	0.996	9.72E-02
P48739	Phosphatidylinositol transfer protein beta isoform	31.52	6.87	74.560937	41.328413	11	15	8	100.2	99.8	0.996	6.19E-01
P49750	YLP motif-containing protein 1	219.849	6.57	44.32049	5.3818555	8	8	8	100.2	99.8	0.996	4.04E-01
P52272	Heterogeneous nuclear ribonucleoprotein M	77.464	8.7	184.23033	37.945205	28	63	28	100.2	99.8	0.996	7.87E-02
P54278	Mismatch repair endonuclease PMS2	95.736	6.86	9.2235234	2.0881671	2	2	2	100.2	99.8	0.996	
P54819	Adenylate kinase 2, mitochondrial	26.461	7.81	135.85146	51.464435	11	29	11	100.2	99.8	0.996	2.44E-01
P55072	Transitional endoplasmic reticulum ATPase	89.266	5.26	428.15996	46.401985	33	113	33	100.2	99.8	0.996	5.82E-01
P61163	Alpha-centractin	42.587	6.64	47.308573	18.882979	7	27	3	100.2	99.8	0.996	3.11E-01
P62258	14-3-3 protein epsilon	29.155	4.74	257.95369	65.882353	18	82	15	100.2	99.8	0.996	4.68E-01
P62857	40S ribosomal protein S28	7.836	10.7	32.073924	46.376812	3	14	3	100.2	99.8	0.996	2.36E-01
P63096	Guanine nucleotide-binding protein G(i) subunit alpha-1	40.335	5.97	34.878102	16.101695	5	11	3	100.2	99.8	0.996	7.79E-01
Q01082	Spectrin beta chain, non-erythrocytic 1	274.439	5.57	694.23538	33.92555	69	140	64	100.2	99.8	0.996	3.67E-01
Q13214	Semaphorin-3B	83.069	8.91	3.3698531	2.5367156	2	2	2	100.2	99.8	0.996	7.41E-01
Q14653	Interferon regulatory factor 3	47.19	5.34	5.0177445	3.7470726	2	2	2	100.2	99.8	0.996	
Q5JTH9	RRP12-like protein	143.611	8.75	87.114019	12.644564	13	18	13	100.2	99.8	0.996	8.59E-01
Q5R372	Rab GTPase-activating protein 1-like	92.454	5.31	15.067238	4.4171779	3	4	3	100.2	99.8	0.996	7.17E-01
Q5VW32	BRO1 domain-containing protein BROX	46.447	7.65	24.849848	9.7323601	4	8	4	100.2	99.8	0.996	8.16E-01
Q5W111	SPRY domain-containing protein 7	21.652	6.7	9.6268772	10.204082	2	2	2	100.2	99.8	0.996	
Q8N5V2	Ephexin-1	82.445	5.57	7.2774123	2.3943662	2	2	2	100.2	99.8	0.996	
Q8TE77	Protein phosphatase Slingshot homolog 3	72.951	5.3	16.952291	1.9726859	1	2	1	100.2	99.8	0.996	
Q8WUD4	Coiled-coil domain-containing protein 12	19.169	7.34	13.309885	15.662651	2	2	2	100.2	99.8	0.996	9.70E-01
Q8WXX5	DnaJ homolog subfamily C member 9	29.891	5.73	91.384652	44.230769	10	14	10	100.2	99.8	0.996	6.38E-01
Q92499	ATP-dependent RNA helicase DDX1	82.38	7.23	189.24152	33.783784	22	37	22	100.2	99.8	0.996	2.24E-01
Q92575	UBX domain-containing protein 4	56.743	6.38	23.699073	10.03937	4	5	4	100.2	99.8	0.996	7.68E-01
Q92959	Solute carrier organic anion transporter family member 2A1	69.998	8.84	9.4511135	3.5769829	2	2	2	100.2	99.8	0.996	5.36E-01
Q96AE4	Far upstream element-binding protein 1	67.518	7.61	221.51312	50.621118	28	53	23	100.2	99.8	0.996	8.07E-04
Q96EM0	Trans-3-hydroxy-L-proline dehydratase	38.113	6.68	2.5696024	2.8248588	1	1	1	100.2	99.8	0.996	
Q96GD0	Pyridoxal phosphate phosphatase	31.678	6.55	2.4410516	2.7027027	1	1	1	100.2	99.8	0.996	
Q99584	Protein S100-A13	11.464	6.16	61.099057	48.979592	6	20	6	100.2	99.8	0.996	2.96E-01
Q99797	Mitochondrial intermediate peptidase	80.589	7.05	26.462117	7.1528752	4	5	4	100.2	99.8	0.996	5.46E-01
Q9BQ70	Transcription factor 25	76.619	6.35	13.124384	3.4023669	2	2	2	100.2	99.8	0.996	4.25E-01
Q9BTX1	Nucleoporin NDC1	76.255	9.09	5.1615917	1.3353116	1	1	1	100.2	99.8	0.996	

Q9H7X7	Intraflagellar transport protein 22 homolog	20.821	5.15	11.089927	11.351351	2	2	2	100.2	99.8	0.996	
Q9H9B1	Histone-lysine N-methyltransferase EHMT1	141.377	5.76	28.950661	7.2419106	7	7	7	100.2	99.8	0.996	4.90E-01
Q9H9B4	Sideroflexin-1	35.596	9.07	76.769762	29.503106	7	12	7	100.2	99.8	0.996	6.45E-01
Q9HD45	Transmembrane 9 superfamily member 3	67.843	7.21	35.47634	6.4516129	4	7	4	100.2	99.8	0.996	4.04E-01
Q9NYH9	U3 small nucleolar RNA-associated protein 6 homolog	70.149	7.28	40.772231	8.5427136	5	7	5	100.2	99.8	0.996	9.91E-01
Q9NZ08	Endoplasmic reticulum aminopeptidase 1	107.166	6.46	16.378375	3.8257173	3	3	3	100.2	99.8	0.996	3.79E-01
Q9P035	Very-long-chain (3R)-3-hydroxyacyl-CoA dehydratase 3	43.132	8.94	12.937845	6.9060773	2	3	2	100.2	99.8	0.996	7.72E-01
Q9P2W9	Syntaxin-18	38.65	5.49	4.6845776	4.4776119	2	2	2	100.2	99.8	0.996	1.94E-02
Q9Y3D9	28S ribosomal protein S23, mitochondrial	21.757	8.9	23.098184	25.263158	4	4	4	100.2	99.8	0.996	
Q9Y490	Talin-1	269.599	6.07	662.34646	36.324282	72	111	71	100.2	99.8	0.996	3.97E-01
Q9Y4B6	Protein VPRBP	168.9	5.06	30.988851	3.450564	5	5	5	100.2	99.8	0.996	5.36E-01
Q9Y6M0	Testisin	34.861	7.62	13.554775	10.509554	3	3	3	100.2	99.8	0.996	8.74E-01
Q9Y6N1	Cytochrome c oxidase assembly protein COX11, mitochondrial	31.41	9.06	4.0246601	3.6231884	1	1	1	100.2	99.8	0.996	
O00299	Chloride intracellular channel protein 1	26.906	5.17	196.26734	67.634855	13	46	12	100.1	99.9	0.998	2.49E-01
O00571	ATP-dependent RNA helicase DDX3X	73.198	7.18	273.92216	45.92145	29	77	28	100.1	99.9	0.998	4.21E-01
O15260	Surfeit locus protein 4	30.374	7.78	46.293931	21.561338	5	12	5	100.1	99.9	0.998	3.41E-01
O15400	Syntaxin-7	29.797	5.55	10.383504	8.4291188	2	2	2	100.1	99.9	0.998	
O60524	Nuclear export mediator factor NEMF	122.878	6.35	31.761885	5.5762082	6	6	6	100.1	99.9	0.998	8.62E-01
O75436	Vacuolar protein sorting-associated protein 26A	38.146	6.57	45.325219	19.571865	5	11	5	100.1	99.9	0.998	8.21E-01
O77932	Decapping and exoribonuclease protein	44.9	7.69	3.992679	2.5252525	1	1	1	100.1	99.9	0.998	
O94905	Erlin-2	37.815	5.62	71.817434	23.893805	8	10	5	100.1	99.9	0.998	5.03E-01
O95182	NADH dehydrogenase [ubiquinone] 1 alpha subcomplex subunit 7	12.544	10.18	26.128837	39.823009	5	6	4	100.1	99.9	0.998	5.48E-01
O95453	Poly(A)-specific ribonuclease PARN	73.405	6.2	51.677633	13.302034	7	9	7	100.1	99.9	0.998	2.22E-01
P15941	Mucin-1	122.029	7.47	20.763311	2.2310757	2	3	2	100.1	99.9	0.998	9.04E-01
P19793	Retinoic acid receptor RXR-alpha	50.778	7.8	9.0161933	3.8961039	2	2	1	100.1	99.9	0.998	
P36551	Oxygen-dependent coproporphyrinogen-III oxidase, mitochondrial	50.12	8.25	23.001664	9.9118943	3	4	3	100.1	99.9	0.998	4.76E-01
P40123	Adenylyl cyclase-associated protein 2	52.791	6.37	57.220174	14.884696	5	7	5	100.1	99.9	0.998	6.39E-01
P47756	F-actin-capping protein subunit beta	31.331	5.59	86.151359	34.296029	9	21	9	100.1	99.9	0.998	9.76E-01
P47985	Cytochrome b-c1 complex subunit Rieske, mitochondrial	29.649	8.32	36.180149	19.343066	6	7	6	100.1	99.9	0.998	8.59E-01
P49366	Deoxyhypusine synthase	40.945	5.36	54.725364	18.97019	5	7	5	100.1	99.9	0.998	7.00E-01
P51648	Fatty aldehyde dehydrogenase	54.813	7.88	121.00476	23.505155	11	19	11	100.1	99.9	0.998	6.96E-01
P55265	Double-stranded RNA-specific adenosine deaminase	135.981	8.65	148.07887	22.267537	26	36	26	100.1	99.9	0.998	7.79E-01
P56199	Integrin alpha-1	130.765	6.29	27.929244	4.0712468	4	4	4	100.1	99.9	0.998	6.79E-01
P60842	Eukaryotic initiation factor 4A-I	46.125	5.48	205.79871	36.945813	18	70	8	100.1	99.9	0.998	8.58E-01
P62269	40S ribosomal protein S18	17.708	10.99	39.113256	38.157895	7	21	7	100.1	99.9	0.998	6.26E-01
P62328	Thymosin beta-4	5.05	5.06	7.9613957	43.181818	2	3	1	100.1	99.9	0.998	
P67775	erine/threonine-protein phosphatase 2A catalytic subunit alpha isofor	35.571	5.54	122.08735	35.92233	8	22	2	100.1	99.9	0.998	3.24E-01
P84101	Small EDRK-rich factor 2	6.896	10.45	13.028202	30.508475	2	3	2	100.1	99.9	0.998	5.35E-01
Q04864	Proto-oncogene c-Rel	68.476	5.86	4.5858626	1.453958	1	1	1	100.1	99.9	0.998	
Q12959	Disks large homolog 1	100.393	5.76	58.495965	10.840708	8	8	7	100.1	99.9	0.998	8.14E-01
Q13547	Histone deacetylase 1	55.068	5.48	50.909427	14.315353	5	9	4	100.1	99.9	0.998	8.67E-01
Q13613	Myotubularin-related protein 1	74.631	7.14	14.811298	6.9172932	5	5	5	100.1	99.9	0.998	7.74E-01
Q13618	Cullin-3	88.873	8.48	20.913819	5.859375	5	6	5	100.1	99.9	0.998	7.17E-01
Q13619	Cullin-4A	87.624	8.13	100.00457	21.870883	18	24	11	100.1	99.9	0.998	7.59E-01
Q14116	Interleukin-18	22.312	4.67	78.467352	43.523316	8	19	8	100.1	99.9	0.998	4.87E-01
Q14126	Desmoglein-2	122.218	5.24	98.817781	13.32737	10	12	10	100.1	99.9	0.998	1.02E-01
Q14558	Phosphoribosyl pyrophosphate synthase-associated protein 1	39.369	7.2	54.231316	23.595506	7	8	7	100.1	99.9	0.998	8.19E-01

Q15181	Inorganic pyrophosphatase	32.639	5.86	229.95516	67.474048	17	47	15	100.1	99.9	0.998	7.73E-01
Q16643	Drebrin	71.385	4.45	127.46278	28.505393	14	23	14	100.1	99.9	0.998	3.77E-01
Q5BJF2	Transmembrane protein 97	20.834	9.38	7.1680385	5.6818182	1	2	1	100.1	99.9	0.998	
Q5MNZ6	WD repeat domain phosphoinositide-interacting protein 3	38.097	7.59	17.27152	9.3023256	2	2	2	100.1	99.9	0.998	7.65E-01
Q5XKP0	MICOS complex subunit MIC13	13.079	9.42	17.709145	28.813559	3	4	3	100.1	99.9	0.998	
Q68CZ2	Tensin-3	155.169	6.81	46.240288	5.8131488	7	8	5	100.1	99.9	0.998	6.81E-01
Q6NZY4	Zinc finger CCHC domain-containing protein 8	78.529	4.87	54.640638	11.032532	5	7	5	100.1	99.9	0.998	6.22E-01
Q6PJG2	ELM2 and SANT domain-containing protein 1	114.918	9.19	15.115759	2.6794258	2	2	2	100.1	99.9	0.998	6.49E-01
Q6VY07	Phosphofurin acidic cluster sorting protein 1	104.833	7.74	36.425287	6.4382139	5	6	5	100.1	99.9	0.998	9.56E-01
Q6WCQ1	Myosin phosphatase Rho-interacting protein	116.461	6.21	79.650083	14.04878	10	13	10	100.1	99.9	0.998	9.53E-01
Q7Z3K3	Pogo transposable element with ZNF domain	155.245	7.4	47.334954	5.5319149	7	8	7	100.1	99.9	0.998	8.41E-01
Q7Z3U7	Protein MON2 homolog	190.237	6.06	69.11847	5.7658707	10	13	10	100.1	99.9	0.998	9.42E-01
Q86TP1	Protein prune homolog	50.168	5.5	19.034968	8.388521	3	4	3	100.1	99.9	0.998	6.77E-01
Q8IWE5	Pleckstrin homology domain-containing family M member 2	112.709	4.93	9.9698052	1.0794897	1	1	1	100.1	99.9	0.998	
Q8IYB8	ATP-dependent RNA helicase SUPV3L1, mitochondrial	87.935	7.99	63.064158	13.867684	9	12	9	100.1	99.9	0.998	7.21E-02
Q8N2F6	Armadillo repeat-containing protein 10	37.517	6.61	3.6064248	3.2069971	1	1	1	100.1	99.9	0.998	
Q8ND04	Protein SMG8	109.614	7.68	14.101785	3.2290616	2	3	2	100.1	99.9	0.998	5.90E-01
Q8TD19	Serine/threonine-protein kinase Nek9	107.1	5.74	46.940191	8.886619	7	9	7	100.1	99.9	0.998	8.92E-01
Q8WUM4	Programmed cell death 6-interacting protein	95.963	6.52	262.68038	36.059908	29	62	29	100.1	99.9	0.998	7.56E-01
Q92905	COP9 signalosome complex subunit 5	37.555	6.54	57.491353	29.341317	9	12	9	100.1	99.9	0.998	2.66E-01
Q96C23	Aldose 1-epimerase	37.742	6.65	33.516609	16.666667	4	6	4	100.1	99.9	0.998	6.12E-01
Q96CV9	Optineurin	65.88	5.24	29.195713	9.1854419	5	6	5	100.1	99.9	0.998	7.53E-01
Q96D46	60S ribosomal export protein NMD3	57.566	7.14	38.848575	14.512922	7	10	7	100.1	99.9	0.998	5.78E-01
Q96S55	ATPase WRNIP1	72.088	6.1	27.229054	9.0225564	6	7	6	100.1	99.9	0.998	8.52E-01
Q96T37	Putative RNA-binding protein 15	107.124	10.08	34.149741	10.337769	7	9	7	100.1	99.9	0.998	7.26E-01
Q96T58	Msx2-interacting protein	402.004	7.64	24.528792	1.3100437	5	5	5	100.1	99.9	0.998	8.39E-01
Q9BT09	Protein canopy homolog 3	30.729	5.49	47.157004	17.625899	5	8	5	100.1	99.9	0.998	9.10E-01
Q9BU23	Lipase maturation factor 2	79.647	10.1	11.039737	4.5261669	2	2	2	100.1	99.9	0.998	4.15E-01
Q9BVV7	Mitochondrial import inner membrane translocase subunit Tim21	28.185	9.7	10.3236	7.6612903	2	2	2	100.1	99.9	0.998	
Q9BZZ5	Apoptosis inhibitor 5	58.968	7.34	88.586841	30.534351	13	17	13	100.1	99.9	0.998	6.10E-01
Q9GZN8	UPF0687 protein C20orf27	19.279	6.84	3.9825493	8.045977	1	1	1	100.1	99.9	0.998	
Q9H1A3	Methyltransferase-like protein 9	36.513	7.36	41.749149	16.037736	4	5	4	100.1	99.9	0.998	4.42E-01
Q9HAU5	Regulator of nonsense transcripts 2	147.717	5.69	18.367641	3.2232704	4	4	4	100.1	99.9	0.998	6.84E-01
Q9NRZ9	Lymphoid-specific helicase	97.012	7.93	57.206233	12.052506	10	12	10	100.1	99.9	0.998	9.31E-01
Q9NTJ3	Structural maintenance of chromosomes protein 4	147.091	6.79	244.45765	25.854037	34	52	34	100.1	99.9	0.998	8.58E-01
Q9NXG2	THUMP domain-containing protein 1	39.291	7.88	39.857548	16.997167	5	6	5	100.1	99.9	0.998	8.59E-01
Q9NYU2	UDP-glucose:glycoprotein glucosyltransferase 1	177.078	5.63	272.42986	22.700965	33	55	33	100.1	99.9	0.998	4.87E-01
Q9UBE0	SUMO-activating enzyme subunit 1	38.426	5.3	107.13804	38.439306	10	20	10	100.1	99.9	0.998	4.67E-01
Q9UK45	U6 snRNA-associated Sm-like protein LSm7	11.595	5.27	26.197582	33.009709	2	7	2	100.1	99.9	0.998	1.94E-02
Q9UM00	Transmembrane and coiled-coil domain-containing protein 1	21.161	9.74	20.356772	11.170213	2	3	2	100.1	99.9	0.998	8.04E-01
Q9UNZ2	NSFL1 cofactor p47	40.548	5.1	124.55585	45.135135	14	23	14	100.1	99.9	0.998	5.85E-01
Q9Y383	Putative RNA-binding protein Luc7-like 2	46.486	10.01	106.58418	25.765306	10	24	6	100.1	99.9	0.998	9.60E-01
Q9Y3A5	Ribosome maturation protein SBDS	28.745	8.75	100.9392	38.4	14	23	14	100.1	99.9	0.998	1.97E-01
Q9Y697	Cysteine desulfurase, mitochondrial	50.164	8.31	27.876303	10.940919	4	6	4	100.1	99.9	0.998	5.13E-02
O00330	Pyruvate dehydrogenase protein X component, mitochondrial	54.089	8.66	26.092266	10.379242	5	6	5	100	100	1	9.84E-01
O00461	Golgi integral membrane protein 4	81.831	4.77	101.01025	17.95977	11	25	11	100	100	1	9.86E-01
O14776	Transcription elongation regulator 1	123.823	8.65	103.68731	14.025501	18	25	17	100	100	1	2.66E-01

O43491	Band 4.1-like protein 2	112.519	5.44	75.581454	14.129353	11	14	11	100	100	1	5.93E-01
O60264	ted matrix-associated actin-dependent regulator of chromatin subfamil	121.828	8.09	98.170204	18.631179	20	28	20	100	100	1	1.13E-01
O60271	C-Jun-amino-terminal kinase-interacting protein 4	146.115	5.15	97.837541	11.582135	13	16	13	100	100	1	3.22E-01
O60427	Fatty acid desaturase 1	51.931	8.87	11.985843	5.6306306	2	3	2	100	100	1	
O60739	Eukaryotic translation initiation factor 1b	12.816	7.37	35.267044	27.433628	3	4	3	100	100	1	8.94E-01
O75146	Huntingtin-interacting protein 1-related protein	119.315	6.67	84.003741	13.389513	13	15	11	100	100	1	5.76E-01
O75607	Nucleoplasmin-3	19.331	4.63	18.483954	8.4269663	1	4	1	100	100	1	
P07686	Beta-hexosaminidase subunit beta	63.071	6.76	63.493034	19.244604	11	19	10	100	100	1	2.39E-01
P07711	Cathepsin L1	37.54	5.45	17.690378	7.8078078	2	3	2	100	100	1	
P09669	Cytochrome c oxidase subunit 6C	8.776	10.39	25.278327	37.333333	4	7	4	100	100	1	8.09E-01
P21399	Cytoplasmic aconitate hydratase	98.337	6.68	84.035256	17.097863	14	18	14	100	100	1	6.79E-01
P22087	rRNA 2'-O-methyltransferase fibrillarin	33.763	10.18	87.880579	33.956386	8	20	8	100	100	1	5.69E-01
P22307	Non-specific lipid-transfer protein	58.956	6.89	99.682029	20.47532	10	21	10	100	100	1	4.66E-02
P31947	14-3-3 protein sigma	27.757	4.74	146.11538	45.967742	12	59	8	100	100	1	6.53E-01
P33316	Deoxyuridine 5'-triphosphate nucleotidohydrolase, mitochondrial	26.547	9.36	91.695408	39.285714	7	23	7	100	100	1	2.89E-01
P41250	Glycine--tRNA ligase	83.113	7.03	105.79618	23.815968	18	31	17	100	100	1	4.78E-01
P41252	Isoleucine--tRNA ligase, cytoplasmic	144.406	6.15	211.49345	22.503962	24	40	24	100	100	1	5.32E-01
P46199	Translation initiation factor IF-2, mitochondrial	81.266	7.15	24.296536	3.9889959	2	3	2	100	100	1	
P51692	Signal transducer and activator of transcription 5B	89.81	6.05	131.46039	18.297332	16	25	3	100	100	1	2.20E-01
P53365	Arfaptin-2	37.832	6.04	51.89542	17.888563	5	8	5	100	100	1	5.09E-01
P58546	Myotrophin	12.887	5.52	43.487861	38.983051	4	10	4	100	100	1	9.38E-01
P61088	Ubiquitin-conjugating enzyme E2 N	17.127	6.57	64.983892	50.657895	8	24	8	100	100	1	5.36E-01
P61106	Ras-related protein Rab-14	23.882	6.21	89.318342	41.395349	8	17	7	100	100	1	2.22E-01
P61225	Ras-related protein Rap-2b	20.491	4.81	30.741963	25.136612	4	5	3	100	100	1	1.12E-01
P62304	Small nuclear ribonucleoprotein E	10.797	9.44	11.25898	25	2	4	2	100	100	1	
P83731	60S ribosomal protein L24	17.768	11.25	58.645956	30.573248	6	14	6	100	100	1	7.85E-01
Q12933	TNF receptor-associated factor 2	55.823	7.53	98.820899	23.353293	12	18	12	100	100	1	2.50E-01
Q13523	Serine/threonine-protein kinase PRP4 homolog	116.916	10.26	47.751169	8.5402185	8	11	8	100	100	1	3.46E-01
Q14203	Dynactin subunit 1	141.607	5.81	167.42772	21.126761	20	25	20	100	100	1	4.47E-01
Q14683	Structural maintenance of chromosomes protein 1A	143.144	7.64	280.29504	27.088402	33	50	33	100	100	1	3.54E-01
Q14746	Conserved oligomeric Golgi complex subunit 2	83.155	6.62	15.685585	4.200542	2	2	2	100	100	1	
Q15008	26S proteasome non-ATPase regulatory subunit 6	45.502	5.62	97.944188	37.532134	15	26	15	100	100	1	8.85E-01
Q15149	Plectin	531.466	5.96	1669.3722	43.23228	193	376	187	100	100	1	9.27E-01
Q15365	Poly(rC)-binding protein 1	37.474	7.09	196.49457	43.539326	13	44	7	100	100	1	1.42E-01
Q15555	Microtubule-associated protein RP/EB family member 2	37.008	5.57	17.052785	10.397554	3	3	2	100	100	1	7.57E-01
Q15796	Mothers against decapentaplegic homolog 2	52.273	6.58	46.966678	11.563169	5	9	5	100	100	1	5.77E-01
Q16401	26S proteasome non-ATPase regulatory subunit 5	56.16	5.48	178.94867	40.674603	16	31	16	100	100	1	6.03E-01
Q5EBL4	RILP-like protein 1	47.079	5.21	9.5246194	4.4665012	1	1	1	100	100	1	
Q5T6F2	Ubiquitin-associated protein 2	117.044	7.34	32.263037	5.9874888	6	8	6	100	100	1	9.21E-01
Q6PKG0	La-related protein 1	123.434	8.82	102.33385	14.324818	14	23	14	100	100	1	1.64E-01
Q6YP21	Kynurenine--oxoglutarate transaminase 3	51.368	8.19	28.24416	7.2687225	3	4	2	100	100	1	4.54E-01
Q7L9L4	MOB kinase activator 1B	25.075	6.73	37.292524	25.925926	5	9	5	100	100	1	6.92E-01
Q86UV5	Ubiquitin carboxyl-terminal hydrolase 48	118.956	6.05	38.661512	6.0869565	5	6	5	100	100	1	7.86E-01
Q8N1B4	Vacuolar protein sorting-associated protein 52 homolog	82.17	5.99	22.104414	6.0857538	3	3	3	100	100	1	5.53E-01
Q8N2G8	GH3 domain-containing protein	57.487	7.88	7.6661511	3.2075472	2	2	2	100	100	1	
Q8NHP8	Putative phospholipase B-like 2	65.43	6.8	27.990674	7.4702886	5	7	5	100	100	1	
Q8WW12	PEST proteolytic signal-containing nuclear protein	18.913	7.49	72.481151	42.134831	6	13	6	100	100	1	5.28E-01

Q8WW59	SPRY domain-containing protein 4	23.114	6.93	11.175776	12.560386	2	2	2	100	100	1	
Q92619	Minor histocompatibility protein HA-1	124.536	6.1	12.412032	2.3767606	2	2	2	100	100	1	9.68E-01
Q92688	Acidic leucine-rich nuclear phosphoprotein 32 family member B	28.77	4.06	91.883308	27.091633	8	32	4	100	100	1	7.26E-02
Q96BP3	Peptidylprolyl isomerase domain and WD repeat-containing protein 1	73.528	7.15	35.98543	12.229102	8	8	8	100	100	1	8.88E-01
Q96H79	Zinc finger CCCH-type antiviral protein 1-like	32.94	8.13	30.150307	18	5	7	5	100	100	1	5.11E-01
Q96JP2	Unconventional myosin-XVB	167.013	8.41	90.021132	9.1503268	12	16	12	100	100	1	3.51E-01
Q96LD4	Tripartite motif-containing protein 47	69.488	6.44	54.518223	15.047022	9	13	9	100	100	1	6.22E-01
Q99575	Ribonucleases P/MRP protein subunit POP1	114.636	9.22	45.661989	8.3984375	6	8	6	100	100	1	8.03E-01
Q9BQ24	Zinc finger FYVE domain-containing protein 21	26.488	8.41	15.434811	4.7008547	1	2	1	100	100	1	
Q9BW19	Kinesin-like protein KIFC1	73.702	8.98	73.84019	15.156018	10	11	10	100	100	1	2.76E-01
Q9BX68	Histidine triad nucleotide-binding protein 2, mitochondrial	17.151	9.16	30.009239	41.104294	5	5	5	100	100	1	7.51E-01
Q9H3R5	Centromere protein H	28.463	5.29	7.0473807	10.121457	2	2	2	100	100	1	
Q9H488	GDP-fucose protein O-fucosyltransferase 1	43.927	8.53	44.286281	14.948454	6	12	6	100	100	1	2.23E-02
Q9H6V9	Lipid droplet-associated hydrolase	37.294	6.54	4.7788467	2.7692308	1	1	1	100	100	1	
Q9HBR0	Putative sodium-coupled neutral amino acid transporter 10	119.687	5.73	10.154815	2.0554066	2	3	2	100	100	1	
Q9NPE2	Neugrin	32.388	9.1	5.1451476	4.1237113	1	2	1	100	100	1	
Q9NQC3	Reticulon-4	129.851	4.5	46.686181	8.0536913	7	9	7	100	100	1	4.37E-01
Q9NUI1	Peroxisomal 2,4-dienoyl-CoA reductase	30.758	9.22	34.069931	26.369863	7	9	7	100	100	1	5.07E-01
Q9NVD7	Alpha-parvin	42.217	5.95	29.171413	11.55914	3	4	3	100	100	1	9.02E-01
Q9NWZ3	Interleukin-1 receptor-associated kinase 4	51.497	5.41	11.553351	5.2173913	2	2	2	100	100	1	
Q9NX55	Huntingtin-interacting protein K	14.656	4.93	91.378511	51.937984	7	13	7	100	100	1	8.81E-01
Q9UHD9	Ubiquilin-2	65.655	5.22	104.7728	24.519231	9	17	5	100	100	1	1.99E-01
Q9Y4D7	Plexin-D1	211.871	7.15	28.322428	2.0779221	3	4	3	100	100	1	1.00E+00
Q9Y4W6	AFG3-like protein 2	88.528	8.66	98.800505	17.565872	15	20	15	100	100	1	8.64E-01
Q9Y5K8	V-type proton ATPase subunit D	28.245	9.36	32.572906	22.672065	4	6	4	100	100	1	8.56E-01
Q9Y679	Ancient ubiquitous protein 1	52.995	8.09	13.224423	6.9327731	3	3	3	100	100	1	5.86E-01
A6NJ78	Probable methyltransferase-like protein 15	46.092	8.62	7.8117506	3.4398034	1	2	1	99.9	100.1	1.002	
O00291	Huntingtin-interacting protein 1	116.148	5.3	50.920429	11.861138	12	13	9	99.9	100.1	1.002	4.32E-01
O00534	von Willebrand factor A domain-containing protein 5A	86.434	6.58	128.25043	18.575064	12	25	12	99.9	100.1	1.002	2.06E-01
O00743	Serine/threonine-protein phosphatase 6 catalytic subunit	35.121	5.69	46.211664	23.934426	7	9	7	99.9	100.1	1.002	8.20E-01
O15031	Plexin-B2	204.997	6.24	70.169756	6.6376496	11	16	11	99.9	100.1	1.002	9.43E-01
O15305	Phosphomannomutase 2	28.064	6.77	56.331601	44.715447	12	17	11	99.9	100.1	1.002	5.42E-01
O43172	U4/U6 small nuclear ribonucleoprotein Prp4	58.412	7.42	81.523688	21.072797	10	16	10	99.9	100.1	1.002	8.85E-01
O43719	HIV Tat-specific factor 1	85.801	4.4	39.948045	9.9337748	8	11	8	99.9	100.1	1.002	7.65E-01
O75494	Serine/arginine-rich splicing factor 10	31.282	11.27	28.546973	15.267176	4	7	4	99.9	100.1	1.002	9.28E-01
P04049	RAF proto-oncogene serine/threonine-protein kinase	73.005	9.2	9.4457313	4.4753086	3	3	2	99.9	100.1	1.002	2.32E-01
P05165	Propionyl-CoA carboxylase alpha chain, mitochondrial	80.008	7.52	18.573311	3.9835165	2	3	2	99.9	100.1	1.002	9.76E-01
P05387	60S acidic ribosomal protein P2	11.658	4.54	128.86306	75.652174	8	31	7	99.9	100.1	1.002	1.97E-01
P06858	Lipoprotein lipase	53.129	8.15	18.91107	7.3684211	3	4	3	99.9	100.1	1.002	5.77E-01
P07339	Cathepsin D	44.524	6.54	90.269823	27.912621	9	18	9	99.9	100.1	1.002	9.89E-02
P08238	Heat shock protein HSP 90-beta	83.212	5.03	558.8126	51.657459	42	272	23	99.9	100.1	1.002	3.15E-01
P10155	60 kDa SS-A/Ro ribonucleoprotein	60.631	8.03	82.280793	20.260223	11	18	11	99.9	100.1	1.002	3.65E-01
P12814	Alpha-actinin-1	102.993	5.41	591.41771	51.008969	41	159	24	99.9	100.1	1.002	6.34E-01
P19784	Casein kinase II subunit alpha'	41.187	8.56	37.30056	15.428571	4	6	3	99.9	100.1	1.002	6.35E-01
P26640	Valine--tRNA ligase	140.387	7.59	260.66672	27.294304	30	56	30	99.9	100.1	1.002	7.93E-01
P27707	Deoxycytidine kinase	30.499	5.21	12.950757	8.8461538	2	2	2	99.9	100.1	1.002	
P29353	SHC-transforming protein 1	62.782	6.44	39.415358	12.521441	5	8	5	99.9	100.1	1.002	2.72E-01

P32322	Pyrroline-5-carboxylate reductase 1, mitochondrial	33.34	7.61	93.709167	23.197492	6	15	6	99.9	100.1	1.002	9.08E-01
P41208	Centrin-2	19.726	5	38.925038	31.976744	5	7	5	99.9	100.1	1.002	7.44E-01
P47897	Glutamine--tRNA ligase	87.743	7.15	127.95004	20.258065	12	23	12	99.9	100.1	1.002	8.41E-01
P49736	DNA replication licensing factor MCM2	101.832	5.52	224.21242	32.079646	29	49	29	99.9	100.1	1.002	8.77E-01
P50402	Emerin	28.976	5.5	29.486874	26.771654	6	8	6	99.9	100.1	1.002	5.69E-01
P52298	Nuclear cap-binding protein subunit 2	17.99	8.21	22.42958	23.076923	4	6	4	99.9	100.1	1.002	
P61019	Ras-related protein Rab-2A	23.531	6.54	118.6545	49.528302	9	22	4	99.9	100.1	1.002	6.59E-01
P62241	40S ribosomal protein S8	24.19	10.32	79.310837	43.269231	8	21	8	99.9	100.1	1.002	1.33E-01
P63104	14-3-3 protein zeta/delta	27.728	4.79	245.45728	61.632653	17	120	10	99.9	100.1	1.002	1.27E-02
P85037	Forkhead box protein K1	75.411	9.32	20.889993	9.9590723	6	6	5	99.9	100.1	1.002	2.25E-01
Q00013	55 kDa erythrocyte membrane protein	52.264	7.37	22.00629	6.4377682	3	4	3	99.9	100.1	1.002	6.84E-01
Q01081	Splicing factor U2AF 35 kDa subunit	27.854	8.81	22.520258	16.666667	3	5	3	99.9	100.1	1.002	
Q05519	Serine/arginine-rich splicing factor 11	53.51	10.52	25.209796	10.53719	5	10	5	99.9	100.1	1.002	4.62E-01
Q13155	minoacyl tRNA synthase complex-interacting multifunctional protein	35.326	8.22	43.895864	22.8125	6	11	6	99.9	100.1	1.002	3.81E-02
Q13442	28 kDa heat- and acid-stable phosphoprotein	20.618	8.87	68.03461	30.38674	7	16	7	99.9	100.1	1.002	6.75E-01
Q13492	Phosphatidylinositol-binding clathrin assembly protein	70.71	7.9	56.640294	11.042945	6	11	6	99.9	100.1	1.002	7.57E-01
Q14019	Coactosin-like protein	15.935	5.67	61.141836	50.704225	8	19	8	99.9	100.1	1.002	3.21E-01
Q14318	Peptidyl-prolyl cis-trans isomerase FKBP8	44.534	4.84	63.740917	21.359223	7	14	7	99.9	100.1	1.002	4.06E-01
Q14839	Chromodomain-helicase-DNA-binding protein 4	217.867	5.86	198.58287	15.271967	23	37	23	99.9	100.1	1.002	8.34E-01
Q14CX7	N-alpha-acetyltransferase 25, NatB auxiliary subunit	112.221	6.64	33.87787	7.3045267	7	10	7	99.9	100.1	1.002	3.27E-01
Q15031	Probable leucine--tRNA ligase, mitochondrial	101.911	8.22	28.701319	6.9767442	5	5	5	99.9	100.1	1.002	9.36E-01
Q15397	Pumilio domain-containing protein KIAA0020	73.538	9.64	35.013871	10.493827	7	9	7	99.9	100.1	1.002	9.75E-01
Q53H82	Beta-lactamase-like protein 2	32.785	6.8	45.230326	23.958333	6	8	6	99.9	100.1	1.002	7.11E-01
Q5K651	Sterile alpha motif domain-containing protein 9	184.165	7.83	12.741585	1.6991819	3	3	2	99.9	100.1	1.002	7.92E-01
Q5VTB9	E3 ubiquitin-protein ligase RNF220	62.725	6.04	16.576613	2.4734982	1	2	1	99.9	100.1	1.002	
Q66K74	Microtubule-associated protein 1S	112.142	7.3	32.794169	5.5712937	6	8	6	99.9	100.1	1.002	8.36E-01
Q676U5	Autophagy-related protein 16-1	68.223	6.64	8.3271706	2.4711697	2	2	2	99.9	100.1	1.002	5.47E-01
Q6NUM9	All-trans-retinol 13,14-reductase	66.777	8.28	15.28985	3.9344262	2	3	2	99.9	100.1	1.002	9.60E-01
Q7L014	Probable ATP-dependent RNA helicase DDX46	117.29	9.29	234.73288	28.322017	28	49	28	99.9	100.1	1.002	7.67E-01
Q7L592	NADH dehydrogenase [ubiquinone] complex I, assembly factor 7	49.206	8.34	19.128715	7.7097506	3	3	3	99.9	100.1	1.002	
Q7Z3B4	Nucleoporin p54	55.401	7.02	50.141805	13.609467	6	8	6	99.9	100.1	1.002	6.39E-01
Q7Z5J4	Retinoic acid-induced protein 1	203.225	8.79	7.446117	1.0493179	1	1	1	99.9	100.1	1.002	
Q86TX2	Acyl-coenzyme A thioesterase 1	46.248	7.34	37.667968	18.527316	7	7	7	99.9	100.1	1.002	7.59E-01
Q8IY81	pre-rRNA processing protein FTSJ3	96.499	8.4	80.929678	14.167651	10	13	10	99.9	100.1	1.002	6.19E-01
Q8N5M4	Tetratricopeptide repeat protein 9C	20	8.92	22.025669	20.467836	3	4	3	99.9	100.1	1.002	4.36E-01
Q8N806	Putative E3 ubiquitin-protein ligase UBR7	47.968	4.81	53.548834	13.176471	6	7	6	99.9	100.1	1.002	7.06E-01
Q8NCN4	E3 ubiquitin-protein ligase RNF169	77.147	9.1	26.207926	7.3446328	4	6	4	99.9	100.1	1.002	4.61E-01
Q8NFW8	N-acylneuraminate cytidylyltransferase	48.349	7.93	48.319949	23.041475	9	11	9	99.9	100.1	1.002	2.75E-01
Q8TDD1	ATP-dependent RNA helicase DDX54	98.534	10.02	36.180614	9.5346198	8	8	7	99.9	100.1	1.002	2.66E-01
Q8WWM7	Ataxin-2-like protein	113.304	8.59	103.02246	19.813953	19	26	18	99.9	100.1	1.002	3.53E-01
Q8WXF1	Paraspeckle component 1	58.706	6.67	52.880295	17.782027	9	17	5	99.9	100.1	1.002	9.61E-01
Q96A49	Synapse-associated protein 1	39.909	4.53	55.128716	15.909091	6	9	6	99.9	100.1	1.002	6.41E-01
Q96IK1	Biorientation of chromosomes in cell division protein 1	19.185	6.33	9.4657423	13.513514	2	2	2	99.9	100.1	1.002	
Q96MG7	Melanoma-associated antigen G1	34.287	9.28	17.261711	15.460526	4	4	4	99.9	100.1	1.002	
Q96PE7	Methylmalonyl-CoA epimerase, mitochondrial	18.737	9.09	11.903412	13.068182	2	2	2	99.9	100.1	1.002	
Q96SB3	Neurabin-2	89.138	4.97	28.822219	6.8711656	4	4	4	99.9	100.1	1.002	3.30E-01
Q99633	Pre-mRNA-splicing factor 18	39.835	8.15	4.9746941	3.5087719	1	1	1	99.9	100.1	1.002	

Q9BRR6	ADP-dependent glucokinase	54.055	6.2	31.055491	8.8531187	3	4	3	99.9	100.1	1.002	4.98E-01
Q9BS26	Endoplasmic reticulum resident protein 44	46.941	5.26	68.329613	26.108374	9	13	9	99.9	100.1	1.002	8.74E-01
Q9BTC0	Death-inducer obliterator 1	243.723	7.88	87.92955	9.7767857	16	16	16	99.9	100.1	1.002	7.24E-01
Q9BZH6	WD repeat-containing protein 11	136.598	6.92	11.944591	2.6960784	3	4	3	99.9	100.1	1.002	
Q9H0V9	VIP36-like protein	39.685	8.38	7.2594005	4.3103448	1	1	1	99.9	100.1	1.002	
Q9H4A4	Aminopeptidase B	72.549	5.74	137.89545	27.692308	15	25	15	99.9	100.1	1.002	9.84E-01
Q9H5Q4	Dimethyladenosine transferase 2, mitochondrial	45.32	9.19	17.380429	10.353535	4	4	4	99.9	100.1	1.002	4.60E-01
Q9H6W3	ifunctional lysine-specific demethylase and histidyl-hydroxylase NO6	71.041	6.46	15.72218	6.2402496	2	2	2	99.9	100.1	1.002	6.84E-01
Q9H910	Hematological and neurological expressed 1-like protein	20.051	9.26	65.663627	55.789474	8	12	8	99.9	100.1	1.002	7.55E-01
Q9HA77	Probable cysteine--tRNA ligase, mitochondrial	62.185	8.34	49.724067	12.234043	5	7	5	99.9	100.1	1.002	6.64E-01
Q9NVH2	Integrator complex subunit 7	106.766	8.02	17.856035	3.6382536	3	3	3	99.9	100.1	1.002	9.68E-01
Q9NVJ2	ADP-ribosylation factor-like protein 8B	21.525	8.43	47.600492	28.494624	6	10	3	99.9	100.1	1.002	5.40E-01
Q9NXH9	tRNA (guanine(26)-N(2))-dimethyltransferase	72.188	7.64	57.581807	15.629742	9	14	9	99.9	100.1	1.002	2.28E-01
Q9NZ01	Very-long-chain enoyl-CoA reductase	36.011	9.45	31.864449	14.935065	5	13	5	99.9	100.1	1.002	6.06E-01
Q9P265	Disco-interacting protein 2 homolog B	171.382	8.09	46.68995	6.535533	8	10	8	99.9	100.1	1.002	6.31E-01
Q9P287	BRCA2 and CDKN1A-interacting protein	35.957	4.61	34.967105	17.834395	5	5	5	99.9	100.1	1.002	5.65E-01
Q9UBF2	Coatomer subunit gamma-2	97.56	5.81	65.358818	12.858783	9	12	6	99.9	100.1	1.002	6.60E-01
Q9UKK9	ADP-sugar pyrophosphatase	24.312	4.94	57.845035	26.484018	6	11	6	99.9	100.1	1.002	9.84E-01
Q9UNM6	26S proteasome non-ATPase regulatory subunit 13	42.918	5.81	125.605	37.5	13	26	13	99.9	100.1	1.002	8.12E-01
Q9Y2W2	WW domain-binding protein 11	69.954	8.38	75.744068	14.820593	10	13	10	99.9	100.1	1.002	7.60E-01
Q9Y606	tRNA pseudouridine synthase A, mitochondrial	47.44	8.41	42.736189	17.330211	7	8	7	99.9	100.1	1.002	5.41E-01
Q9Y6M5	Zinc transporter 1	55.264	6.48	45.931861	17.948718	7	10	7	99.9	100.1	1.002	1.47E-01
O60658	finity cAMP-specific and IBMX-insensitive 3',5'-cyclic phosphodieste	93.245	6.11	7.370301	1.9300362	2	2	1	99.8	100.2	1.004	
O60669	Monocarboxylate transporter 2	52.166	9.31	7.1373613	3.9748954	2	2	2	99.8	100.2	1.004	
O60671	Cell cycle checkpoint protein RAD1	31.807	4.83	14.794799	8.5106383	2	2	2	99.8	100.2	1.004	
O60841	Eukaryotic translation initiation factor 5B	138.742	5.49	222.01231	21.885246	26	48	25	99.8	100.2	1.004	7.82E-01
O75391	Sperm-associated antigen 7	26.018	7.91	24.47547	10.132159	2	5	2	99.8	100.2	1.004	9.47E-01
O75962	Triple functional domain protein	346.683	6.37	97.196663	6.0058121	16	18	16	99.8	100.2	1.004	6.36E-01
O76071	Probable cytosolic iron-sulfur protein assembly protein CIAO1	37.816	4.97	55.154891	32.448378	8	9	8	99.8	100.2	1.004	8.95E-01
P07951	Tropomyosin beta chain	32.831	4.7	143.01357	35.915493	15	48	4	99.8	100.2	1.004	8.04E-01
P09110	3-ketoacyl-CoA thiolase, peroxisomal	44.264	8.44	58.043829	25.471698	7	8	7	99.8	100.2	1.004	3.13E-01
P11387	DNA topoisomerase 1	90.669	9.31	141.17197	23.267974	17	32	17	99.8	100.2	1.004	6.00E-01
P15121	Aldose reductase	35.83	6.98	72.843015	28.481013	10	21	9	99.8	100.2	1.004	3.39E-01
P19388	DNA-directed RNA polymerases I, II, and III subunit RPABC1	24.536	5.95	38.463175	23.333333	4	6	4	99.8	100.2	1.004	5.71E-01
P22392	Nucleoside diphosphate kinase B	17.287	8.41	106.23692	63.157895	11	41	5	99.8	100.2	1.004	3.29E-01
P22681	E3 ubiquitin-protein ligase CBL	99.569	6.54	31.490306	6.7328918	5	7	5	99.8	100.2	1.004	9.58E-01
P23368	NAD-dependent malic enzyme, mitochondrial	65.402	7.61	35.314792	11.986301	5	5	5	99.8	100.2	1.004	1.00E+00
P24941	Cyclin-dependent kinase 2	33.908	8.68	90.577814	38.926174	11	24	8	99.8	100.2	1.004	1.24E-01
P28072	Proteasome subunit beta type-6	25.341	4.92	85.668395	32.635983	8	19	8	99.8	100.2	1.004	2.03E-01
P29144	Tripeptidyl-peptidase 2	138.263	6.32	104.84403	18.494796	23	28	23	99.8	100.2	1.004	3.80E-01
P31937	3-hydroxyisobutyrate dehydrogenase, mitochondrial	35.306	8.13	98.042928	27.678571	6	13	6	99.8	100.2	1.004	6.17E-01
P35268	60S ribosomal protein L22	14.778	9.19	23.33736	18.75	2	6	2	99.8	100.2	1.004	
P40818	Ubiquitin carboxyl-terminal hydrolase 8	127.444	8.51	32.797708	5.3667263	4	5	4	99.8	100.2	1.004	3.22E-01
P42766	60S ribosomal protein L35	14.543	11.05	16.984822	26.01626	4	4	4	99.8	100.2	1.004	5.53E-01
P48634	Protein PRRC2A	228.724	9.45	82.048559	8.8548911	11	15	10	99.8	100.2	1.004	7.03E-01
P53004	Biliverdin reductase A	33.407	6.44	94.484793	30.067568	10	22	10	99.8	100.2	1.004	4.56E-01
P55795	Heterogeneous nuclear ribonucleoprotein H2	49.232	6.3	112.10998	29.621381	11	38	5	99.8	100.2	1.004	9.29E-01

P60174	Triosephosphate isomerase	30.772	5.92	256.50843	66.083916	16	77	16	99.8	100.2	1.004	8.74E-01
P60981	Destrin	18.493	7.85	73.956365	39.393939	8	24	7	99.8	100.2	1.004	3.77E-01
P61009	Signal peptidase complex subunit 3	20.301	8.62	21.208477	11.666667	2	3	2	99.8	100.2	1.004	5.53E-01
P61916	Epididymal secretory protein E1	16.559	7.65	39.024294	27.81457	4	9	4	99.8	100.2	1.004	9.72E-01
Q01518	Adenylyl cyclase-associated protein 1	51.869	8.06	237.34312	39.789474	18	56	18	99.8	100.2	1.004	1.05E-01
Q0ZGT2	Nexilin	80.609	5.33	20.232322	4.1481481	3	4	3	99.8	100.2	1.004	
Q12768	WASH complex subunit strumpellin	134.201	6.98	31.338879	5.0905953	6	7	6	99.8	100.2	1.004	5.86E-01
Q13043	Serine/threonine-protein kinase 4	55.595	5.07	46.88814	17.043121	7	9	4	99.8	100.2	1.004	4.98E-01
Q13362	/threonine-protein phosphatase 2A 56 kDa regulatory subunit gamma i	61.022	6.87	42.964635	11.068702	5	8	3	99.8	100.2	1.004	8.71E-01
Q14008	Cytoskeleton-associated protein 5	225.352	7.8	230.56362	17.224409	34	52	33	99.8	100.2	1.004	4.33E-01
Q15629	Translocating chain-associated membrane protein 1	43.044	9.63	14.405583	2.9411765	1	2	1	99.8	100.2	1.004	
Q15643	Thyroid receptor-interacting protein 11	227.447	5.26	57.060683	5.5078322	10	10	10	99.8	100.2	1.004	4.59E-01
Q15645	Pachytene checkpoint protein 2 homolog	48.52	6.09	114.31587	26.157407	10	19	10	99.8	100.2	1.004	5.81E-01
Q16637	Survival motor neuron protein	31.828	6.55	55.14045	10.204082	4	7	4	99.8	100.2	1.004	7.57E-01
Q29RF7	Sister chromatid cohesion protein PDS5 homolog A	150.734	7.91	138.93409	18.997756	20	26	18	99.8	100.2	1.004	9.28E-01
Q4G0J3	La-related protein 7	66.857	9.55	20.688316	4.6391753	2	4	2	99.8	100.2	1.004	8.26E-01
Q5JTV8	Torsin-1A-interacting protein 1	66.208	8.18	52.027018	16.123499	8	12	8	99.8	100.2	1.004	4.97E-01
Q5VZK9	Leucine-rich repeat-containing protein 16A	151.462	7.85	36.458788	4.8140044	5	6	5	99.8	100.2	1.004	5.22E-01
Q6P996	Pyridoxal-dependent decarboxylase domain-containing protein 1	86.652	5.38	74.474755	18.274112	12	18	12	99.8	100.2	1.004	3.13E-01
Q7L3T8	Probable proline--tRNA ligase, mitochondrial	53.228	8.1	5.3479472	2.7368421	1	1	1	99.8	100.2	1.004	
Q8IYL3	UPF0688 protein C1orf174	25.961	6.9	6.7880789	5.7613169	1	1	1	99.8	100.2	1.004	
Q8NFH4	Nucleoporin Nup37	36.684	5.92	12.04809	7.0552147	2	3	2	99.8	100.2	1.004	
Q92538	olgi-specific brefeldin A-resistance guanine nucleotide exchange factor	206.315	5.73	151.44152	12.587413	19	31	18	99.8	100.2	1.004	8.89E-01
Q92598	Heat shock protein 105 kDa	96.804	5.39	327.66355	40.675991	30	68	26	99.8	100.2	1.004	4.64E-01
Q92616	Translational activator GCN1	292.572	7.47	457.52555	27.854736	66	94	66	99.8	100.2	1.004	6.93E-01
Q969U7	Proteasome assembly chaperone 2	29.377	6.98	16.885022	13.257576	4	5	4	99.8	100.2	1.004	5.53E-01
Q96BI1	Solute carrier family 22 member 18	44.816	9.57	4.7988761	2.8301887	1	1	1	99.8	100.2	1.004	
Q96C01	Protein FAM136A	15.631	7.61	40.899424	41.304348	6	8	6	99.8	100.2	1.004	7.61E-01
Q96IR7	4-hydroxyphenylpyruvate dioxygenase-like protein	39.361	7.03	22.89034	6.1994609	2	6	2	99.8	100.2	1.004	6.47E-01
Q96M27	Protein PRRC1	46.672	5.83	32.38046	7.8651685	3	5	3	99.8	100.2	1.004	3.57E-01
Q99959	Plakophilin-2	97.355	9.33	7.892807	1.816118	2	3	1	99.8	100.2	1.004	
Q9BQC6	Ribosomal protein 63, mitochondrial	12.259	11.44	11.996446	12.745098	1	2	1	99.8	100.2	1.004	
Q9NPA8	Transcription and mRNA export factor ENY2	11.521	9.33	18.018876	23.762376	2	3	2	99.8	100.2	1.004	
Q9NQE9	Histidine triad nucleotide-binding protein 3	20.348	6.6	11.214928	15.934066	2	2	2	99.8	100.2	1.004	
Q9NVQ4	Fas apoptotic inhibitory molecule 1	20.202	5.83	15.641523	15.642458	3	4	3	99.8	100.2	1.004	
Q9NW82	WD repeat-containing protein 70	73.155	6.33	60.360317	17.125382	9	11	9	99.8	100.2	1.004	7.95E-01
Q9NXN4	Ganglioside-induced differentiation-associated protein 2	56.189	5.74	8.4114445	4.4265594	2	2	2	99.8	100.2	1.004	
Q9P2T1	GMP reductase 2	37.85	7.23	20.579721	15.517241	4	4	4	99.8	100.2	1.004	6.47E-01
Q9UGP4	LIM domain-containing protein 1	72.144	6.65	13.197804	4.5857988	2	2	2	99.8	100.2	1.004	6.84E-01
Q9UI30	Multifunctional methyltransferase subunit TRM112-like protein	14.19	5.26	45.337113	48.8	5	10	5	99.8	100.2	1.004	5.88E-01
Q9UKV3	Apoptotic chromatin condensation inducer in the nucleus	151.771	6.43	124.13099	17.747949	20	24	20	99.8	100.2	1.004	6.69E-01
Q9Y2H6	Fibronectin type-III domain-containing protein 3A	131.767	6.71	20.606759	1.836394	2	3	2	99.8	100.2	1.004	7.49E-01
Q9Y2S0	DNA-directed RNA polymerases I and III subunit RPAC2	15.227	5.8	26.74893	18.796992	3	5	3	99.8	100.2	1.004	7.25E-01
Q9Y3A2	Probable U3 small nucleolar RNA-associated protein 11	30.428	10.15	13.803018	10.671937	3	3	3	99.8	100.2	1.004	7.49E-01
Q9Y3D6	Mitochondrial fission 1 protein	16.927	8.79	28.859718	15.789474	2	8	2	99.8	100.2	1.004	6.41E-01
Q9Y4A5	Transformation/transcription domain-associated protein	437.318	8.19	31.663857	2.1249028	9	9	9	99.8	100.2	1.004	3.78E-01
Q9Y4L1	Hypoxia up-regulated protein 1	111.266	5.22	306.09361	31.131131	30	70	30	99.8	100.2	1.004	3.75E-01

Q9Y4W2	Ribosomal biogenesis protein LAS1L	83.013	4.73	48.445416	10.626703	7	9	7	99.8	100.2	1.004	9.21E-01
Q9Y6U3	Adseverin	80.438	5.71	22.821119	6.7132867	3	4	3	99.8	100.2	1.004	6.90E-01
A0FGR8	Extended synaptotagmin-2	102.294	9.26	146.07227	19.869707	16	31	15	99.7	100.3	1.006	6.13E-01
A4D1E9	GTP-binding protein 10	42.906	9.03	30.520847	15.245478	4	4	4	99.7	100.3	1.006	2.93E-01
A6NKF1	SAC3 domain-containing protein 1	43.526	8.69	17.636037	9.6534653	3	3	3	99.7	100.3	1.006	5.77E-01
B5ME19	Eukaryotic translation initiation factor 3 subunit C-like protein	105.407	5.64	174.27048	24.179431	21	37	21	99.7	100.3	1.006	6.66E-01
O00231	26S proteasome non-ATPase regulatory subunit 11	47.434	6.48	114.78573	28.672986	11	23	11	99.7	100.3	1.006	8.60E-01
O15397	Importin-8	119.861	5.16	54.257971	8.8717454	7	8	7	99.7	100.3	1.006	5.96E-01
O60341	Lysine-specific histone demethylase 1A	92.845	6.52	71.694477	21.126761	12	14	12	99.7	100.3	1.006	6.59E-01
O60678	Protein arginine N-methyltransferase 3	59.838	5.35	41.461346	9.2278719	5	6	5	99.7	100.3	1.006	2.22E-01
O94874	E3 UFM1-protein ligase 1	89.54	6.79	70.369776	15.11335	13	14	13	99.7	100.3	1.006	8.22E-01
O95168	NADH dehydrogenase [ubiquinone] 1 beta subcomplex subunit 4	15.199	9.85	15.385959	21.705426	2	3	2	99.7	100.3	1.006	6.50E-01
O95487	Protein transport protein Sec24B	137.331	6.67	5.6294869	0.8675079	1	1	1	99.7	100.3	1.006	
O96007	Molybdopterin synthase catalytic subunit	20.931	5.44	6.3660268	9.5744681	1	1	1	99.7	100.3	1.006	
P00491	Purine nucleoside phosphorylase	32.097	6.95	112.15735	44.636678	11	24	11	99.7	100.3	1.006	5.33E-01
P00505	Aspartate aminotransferase, mitochondrial	47.487	9.01	153.96152	32.790698	16	39	16	99.7	100.3	1.006	7.09E-01
P00533	Epidermal growth factor receptor	134.19	6.68	193.6282	23.966942	22	37	21	99.7	100.3	1.006	6.94E-01
P04899	Guanine nucleotide-binding protein G(i) subunit alpha-2	40.425	5.54	58.721971	25.070423	7	14	5	99.7	100.3	1.006	5.66E-01
P05198	Eukaryotic translation initiation factor 2 subunit 1	36.089	5.08	145.50182	55.555556	17	36	17	99.7	100.3	1.006	6.09E-01
P05386	60S acidic ribosomal protein P1	11.507	4.32	45.755481	71.929825	4	12	3	99.7	100.3	1.006	6.06E-01
P09622	Dihydrolipoyl dehydrogenase, mitochondrial	54.143	7.85	117.57344	23.379175	10	23	10	99.7	100.3	1.006	5.59E-01
P09874	Poly [ADP-ribose] polymerase 1	113.012	8.88	354.31145	36.686391	37	75	37	99.7	100.3	1.006	2.15E-01
P14866	Heterogeneous nuclear ribonucleoprotein L	64.092	8.22	211.78612	41.765705	17	50	16	99.7	100.3	1.006	1.26E-02
P15586	N-acetylglucosamine-6-sulfatase	62.042	8.31	26.574469	11.413043	6	6	6	99.7	100.3	1.006	9.66E-01
P18615	Negative elongation factor E	43.214	9.33	44.438458	23.421053	7	8	7	99.7	100.3	1.006	8.83E-01
P25685	DnaJ homolog subfamily B member 1	38.02	8.63	90.024361	32.352941	10	18	9	99.7	100.3	1.006	9.09E-02
P31942	Heterogeneous nuclear ribonucleoprotein H3	36.903	6.87	117.68766	30.057803	8	25	7	99.7	100.3	1.006	9.65E-01
P42704	Leucine-rich PPR motif-containing protein, mitochondrial	157.805	6.13	547.34623	47.560976	62	114	61	99.7	100.3	1.006	6.37E-01
P46087	Probable 28S rRNA (cytosine(4447)-C(5))-methyltransferase	89.247	9.23	191.33596	31.896552	21	41	21	99.7	100.3	1.006	2.71E-01
P49005	DNA polymerase delta subunit 2	51.257	5.58	38.641144	13.859275	5	6	5	99.7	100.3	1.006	3.07E-01
P49792	E3 SUMO-protein ligase RanBP2	357.974	6.2	473.56905	22.859801	62	95	43	99.7	100.3	1.006	5.09E-02
P50990	T-complex protein 1 subunit theta	59.583	5.6	419.95072	56.934307	31	93	31	99.7	100.3	1.006	8.47E-02
P51571	Translocon-associated protein subunit delta	18.987	6.15	56.533941	19.653179	3	11	3	99.7	100.3	1.006	8.21E-01
P51946	Cyclin-H	37.619	7.15	17.656442	4.9535604	1	2	1	99.7	100.3	1.006	
P52758	Ribonuclease UK114	14.485	8.68	36.741175	40.875912	4	5	4	99.7	100.3	1.006	3.94E-01
P60891	Ribose-phosphate pyrophosphokinase 1	34.812	6.98	121.03189	35.534591	10	24	5	99.7	100.3	1.006	7.87E-01
P62273	40S ribosomal protein S29	6.672	10.13	8.1868608	14.285714	2	6	2	99.7	100.3	1.006	6.84E-01
P62942	Peptidyl-prolyl cis-trans isomerase FKBP1A	11.943	8.16	49.997688	25	2	9	2	99.7	100.3	1.006	6.33E-01
P63220	40S ribosomal protein S21	9.106	8.5	30.990039	38.554217	4	9	4	99.7	100.3	1.006	3.38E-01
P78318	Immunoglobulin-binding protein 1	39.198	5.38	51.502611	21.238938	6	11	6	99.7	100.3	1.006	3.06E-01
Q02127	Dihydroorotate dehydrogenase (quinone), mitochondrial	42.841	9.67	41.35537	17.721519	6	8	6	99.7	100.3	1.006	6.57E-01
Q07157	Tight junction protein ZO-1	195.34	6.7	37.875576	3.6613272	6	9	5	99.7	100.3	1.006	4.40E-01
Q13057	Bifunctional coenzyme A synthase	62.29	6.99	34.844574	13.475177	6	8	6	99.7	100.3	1.006	5.04E-01
Q13098	COP9 signalosome complex subunit 1	55.501	6.74	72.58131	20.977597	8	13	8	99.7	100.3	1.006	1.48E-01
Q13610	Periodic tryptophan protein 1 homolog	55.793	4.77	19.145499	8.3832335	3	4	3	99.7	100.3	1.006	6.69E-01
Q13625	Apoptosis-stimulating of p53 protein 2	125.538	6.07	34.784917	5.7624113	4	4	4	99.7	100.3	1.006	4.63E-01
Q14204	Cytoplasmic dynein 1 heavy chain 1	532.072	6.4	843.76173	24.537236	108	171	108	99.7	100.3	1.006	5.40E-01

Q14690	Protein RRP5 homolog	208.57	8.87	86.863249	7.696419	12	16	12	99.7	100.3	1.006	6.45E-01
Q15904	V-type proton ATPase subunit S1	51.993	6.14	34.184045	7.6595745	3	5	3	99.7	100.3	1.006	5.53E-01
Q2NKX8	DNA excision repair protein ERCC-6-like	141.015	5.31	51.768256	8.4	8	10	8	99.7	100.3	1.006	5.63E-01
Q6P587	Acylpyruvase FAHD1, mitochondrial	24.827	7.39	23.644641	10.267857	2	3	2	99.7	100.3	1.006	3.83E-01
Q6PJT7	Zinc finger CCCH domain-containing protein 14	82.823	7.31	32.415597	6.3858696	3	4	3	99.7	100.3	1.006	4.05E-01
Q6UX04	Peptidyl-prolyl cis-trans isomerase CWC27 homolog	53.814	5.8	19.798243	3.1779661	1	4	1	99.7	100.3	1.006	
Q7L0Y3	Mitochondrial ribonuclease P protein 1	47.317	9.36	82.134811	38.709677	14	18	14	99.7	100.3	1.006	5.12E-01
Q7Z2W4	Zinc finger CCCH-type antiviral protein 1	101.367	8.4	116.34747	24.833703	17	20	17	99.7	100.3	1.006	6.30E-01
Q86SX6	Glutaredoxin-related protein 5, mitochondrial	16.618	6.79	32.497749	28.025478	3	6	3	99.7	100.3	1.006	8.30E-01
Q86UU1	Pleckstrin homology-like domain family B member 1	151.068	8.63	50.436199	6.1002179	7	9	7	99.7	100.3	1.006	4.22E-01
Q8IW45	ATP-dependent (S)-NAD(P)H-hydrate dehydratase	36.553	8.06	4.5342923	4.3227666	2	2	2	99.7	100.3	1.006	
Q8IXI1	Mitochondrial Rho GTPase 2	68.075	5.86	22.251952	4.368932	2	4	2	99.7	100.3	1.006	5.66E-01
Q8N5C6	S1 RNA-binding domain-containing protein 1	111.705	8.72	6.8991384	2.0100503	2	2	2	99.7	100.3	1.006	8.36E-01
Q8NEZ5	F-box only protein 22	44.48	7.03	52.295908	11.662531	4	8	4	99.7	100.3	1.006	5.47E-01
Q8NFV4	Alpha/beta hydrolase domain-containing protein 11	34.668	9.48	21.720115	12.698413	3	4	3	99.7	100.3	1.006	7.04E-01
Q8TAF3	WD repeat-containing protein 48	76.162	7.03	13.974892	5.0221566	3	4	3	99.7	100.3	1.006	6.84E-01
Q8TAQ2	SWI/SNF complex subunit SMARCC2	132.797	5.69	124.62824	12.932455	13	21	8	99.7	100.3	1.006	6.23E-01
Q8WXA3	RUN and FYVE domain-containing protein 2	75.008	6.51	16.657051	5.3435115	3	3	3	99.7	100.3	1.006	9.33E-01
Q969Q5	Ras-related protein Rab-24	23.109	6.23	9.4409317	5.4187192	1	1	1	99.7	100.3	1.006	
Q96B54	Zinc finger protein 428	20.468	4.17	15.163674	16.489362	2	3	2	99.7	100.3	1.006	8.65E-01
Q96DI7	U5 small nuclear ribonucleoprotein 40 kDa protein	39.286	8.1	32.04589	16.246499	5	7	5	99.7	100.3	1.006	9.78E-01
Q96FW1	Ubiquitin thioesterase OTUB1	31.264	4.94	112.72709	40.221402	8	20	8	99.7	100.3	1.006	1.82E-01
Q96GK7	Fumarylacetoacetate hydrolase domain-containing protein 2A	34.574	8.24	73.713747	29.617834	7	12	7	99.7	100.3	1.006	1.76E-01
Q99471	Prefoldin subunit 5	17.317	6.33	44.968785	40.909091	6	12	6	99.7	100.3	1.006	3.35E-01
Q99549	M-phase phosphoprotein 8	97.123	6.06	22.953056	3.255814	2	3	2	99.7	100.3	1.006	
Q99614	Tetratricopeptide repeat protein 1	33.505	4.84	64.970418	35.616438	9	11	9	99.7	100.3	1.006	5.82E-01
Q9BQ67	Glutamate-rich WD repeat-containing protein 1	49.388	4.92	50.428479	12.107623	5	8	5	99.7	100.3	1.006	7.83E-01
Q9BTE3	Mini-chromosome maintenance complex-binding protein	72.934	5.87	32.154945	12.149533	6	6	6	99.7	100.3	1.006	1.62E-01
Q9BZK7	F-box-like/WD repeat-containing protein TBL1XR1	55.56	5.55	89.876677	25.291829	10	13	5	99.7	100.3	1.006	1.00E+00
Q9HBG4	V-type proton ATPase 116 kDa subunit a isoform 4	96.323	6.1	10.218605	2.6190476	2	2	2	99.7	100.3	1.006	
Q9NRV9	Heme-binding protein 1	21.084	5.8	28.919662	21.693122	3	6	3	99.7	100.3	1.006	8.10E-01
Q9NVH1	DnaJ homolog subfamily C member 11	63.239	8.4	47.915259	13.416816	7	11	7	99.7	100.3	1.006	7.88E-01
Q9NZJ0	Denticleless protein homolog	79.417	8.87	3.2819137	1.0958904	1	1	1	99.7	100.3	1.006	
Q9NZT2	Opioid growth factor receptor	73.28	4.84	14.717268	4.1358936	2	2	2	99.7	100.3	1.006	8.59E-01
Q9P2E5	Chondroitin sulfate glucuronyltransferase	85.894	7.83	6.0998165	2.3316062	2	2	2	99.7	100.3	1.006	
Q9UFG5	UPF0449 protein C19orf25	12.87	5.07	10.17733	23.728814	2	2	2	99.7	100.3	1.006	
Q9UJY1	Heat shock protein beta-8	21.591	5.12	7.8023817	10.204082	2	3	2	99.7	100.3	1.006	
Q9UNF1	Melanoma-associated antigen D2	64.914	9.32	120.8256	29.372937	15	22	15	99.7	100.3	1.006	2.10E-02
Q9UPT5	Exocyst complex component 7	83.33	6.79	36.777635	8.1632653	6	7	6	99.7	100.3	1.006	9.11E-01
Q9Y2D5	A-kinase anchor protein 2	94.603	5.11	14.177774	4.4237485	2	2	2	99.7	100.3	1.006	9.47E-01
Q9Y2H0	Disks large-associated protein 4	107.945	7.08	12.601191	1.9153226	2	2	2	99.7	100.3	1.006	
Q9Y2X3	Nucleolar protein 58	59.541	8.92	112.30993	21.172023	10	21	10	99.7	100.3	1.006	1.32E-02
Q9Y320	Thioredoxin-related transmembrane protein 2	34.016	8.69	19.276859	13.513514	4	5	4	99.7	100.3	1.006	
Q9Y3B8	Oligoribonuclease, mitochondrial	26.816	6.87	37.542863	23.628692	5	8	5	99.7	100.3	1.006	6.89E-01
Q9Y3F4	Serine-threonine kinase receptor-associated protein	38.414	5.12	162.79037	49.428571	14	38	14	99.7	100.3	1.006	7.21E-01
Q9Y666	Solute carrier family 12 member 7	119.029	6.71	27.691291	4.8014774	6	10	6	99.7	100.3	1.006	4.20E-01
Q9Y6G3	39S ribosomal protein L42, mitochondrial	16.65	8.35	11.372737	10.56338	1	1	1	99.7	100.3	1.006	

Q9Y6K9	NF-kappa-B essential modulator	48.167	5.71	66.970505	17.661098	6	9	6	99.7	100.3	1.006	5.68E-01
O00429	Dynamin-1-like protein	81.826	6.81	139.6628	24.592391	14	22	14	99.6	100.4	1.008	7.81E-01
O14929	Histone acetyltransferase type B catalytic subunit	49.481	5.69	86.66956	21.479714	8	16	8	99.6	100.4	1.008	2.19E-01
O43159	Ribosomal RNA-processing protein 8	50.683	9.42	37.716574	15.350877	6	9	6	99.6	100.4	1.008	2.78E-01
O43324	Eukaryotic translation elongation factor 1 epsilon-1	19.798	8.54	34.115188	25.287356	5	7	5	99.6	100.4	1.008	3.24E-01
O43847	Nardilysin	131.488	5	107.34414	11.826087	14	23	14	99.6	100.4	1.008	9.76E-01
O60293	Zinc finger C3H1 domain-containing protein	226.214	8.13	59.811921	6.0331825	10	11	10	99.6	100.4	1.008	3.32E-01
O60306	Intron-binding protein aquarius	171.186	6.37	33.331107	4.7811448	8	8	7	99.6	100.4	1.008	7.88E-01
O60869	Endothelial differentiation-related factor 1	16.359	9.95	33.546912	35.135135	5	13	5	99.6	100.4	1.008	4.39E-01
O75165	DnaJ homolog subfamily C member 13	254.252	6.74	116.86744	9.050379	18	22	18	99.6	100.4	1.008	7.27E-01
P08240	Signal recognition particle receptor subunit alpha	69.767	8.95	137.79902	25.07837	16	33	16	99.6	100.4	1.008	6.40E-01
P08754	Guanine nucleotide-binding protein G(k) subunit alpha	40.506	5.69	46.650777	21.186441	6	13	4	99.6	100.4	1.008	6.21E-01
P10515	:-residue acetyltransferase component of pyruvate dehydrogenase com	68.953	7.84	107.04009	22.874807	13	24	13	99.6	100.4	1.008	2.09E-01
P11177	Pyruvate dehydrogenase E1 component subunit beta, mitochondrial	39.208	6.65	127.48933	30.083565	9	25	9	99.6	100.4	1.008	2.16E-01
P13010	X-ray repair cross-complementing protein 5	82.652	5.81	191.89495	29.371585	20	44	20	99.6	100.4	1.008	2.39E-02
P17655	Calpain-2 catalytic subunit	79.945	4.98	126.18836	20.857143	13	26	13	99.6	100.4	1.008	2.82E-01
P30566	Adenylosuccinate lyase	54.854	7.11	40.379292	7.8512397	4	10	4	99.6	100.4	1.008	6.20E-01
P32004	Neural cell adhesion molecule L1	139.915	6.24	74.077546	12.171838	12	14	12	99.6	100.4	1.008	3.23E-01
P43155	Carnitine O-acetyltransferase	70.812	8.44	15.938522	5.1118211	3	3	3	99.6	100.4	1.008	
P46779	60S ribosomal protein L28	15.738	12.02	33.46341	27.737226	5	10	5	99.6	100.4	1.008	9.10E-01
P48643	T-complex protein 1 subunit epsilon	59.633	5.66	350.01285	55.452865	32	91	31	99.6	100.4	1.008	4.77E-01
P48960	CD97 antigen	91.809	6.87	54.95935	8.502994	5	7	5	99.6	100.4	1.008	6.09E-01
P50454	Serpin H1	46.411	8.69	171.79655	34.92823	13	34	13	99.6	100.4	1.008	9.49E-01
P51452	Dual specificity protein phosphatase 3	20.465	7.8	71.441797	44.324324	6	12	6	99.6	100.4	1.008	1.96E-01
P55735	Protein SEC13 homolog	35.518	5.48	69.37815	24.534161	6	17	6	99.6	100.4	1.008	8.88E-01
P56134	ATP synthase subunit f, mitochondrial	10.911	9.67	18.712871	25.531915	2	5	2	99.6	100.4	1.008	2.99E-02
P62899	60S ribosomal protein L31	14.454	10.54	24.190556	33.6	5	12	5	99.6	100.4	1.008	3.95E-01
P63167	Dynein light chain 1, cytoplasmic	10.359	7.4	69.590062	32.58427	2	13	2	99.6	100.4	1.008	3.19E-01
P63208	S-phase kinase-associated protein 1	18.646	4.54	65.366999	41.104294	7	24	7	99.6	100.4	1.008	3.45E-02
P78330	Phosphoserine phosphatase	24.992	5.69	15.72057	16.444444	4	5	4	99.6	100.4	1.008	5.86E-01
P84103	Serine/arginine-rich splicing factor 3	19.318	11.65	34.753258	39.02439	7	14	6	99.6	100.4	1.008	1.85E-01
Q06136	3-ketodihydrosphingosine reductase	36.164	7.12	4.1455725	3.313253	1	1	1	99.6	100.4	1.008	
Q12769	Nuclear pore complex protein Nup160	162.017	5.5	85.248506	7.0334262	8	14	8	99.6	100.4	1.008	3.12E-01
Q13049	E3 ubiquitin-protein ligase TRIM32	71.942	6.98	8.7373042	2.7565084	2	2	2	99.6	100.4	1.008	4.59E-01
Q13136	Liprin-alpha-1	135.695	6.29	85.84888	13.477537	13	15	12	99.6	100.4	1.008	6.31E-01
Q13310	Polyadenylate-binding protein 4	70.738	9.26	147.1327	25	15	36	9	99.6	100.4	1.008	3.57E-01
Q13546	Receptor-interacting serine/threonine-protein kinase 1	75.883	6.33	23.097077	7.6005961	4	5	4	99.6	100.4	1.008	5.49E-01
Q13895	Bystin	49.57	8.12	34.362708	16.475973	6	9	6	99.6	100.4	1.008	1.87E-01
Q14739	Lamin-B receptor	70.658	9.36	5.9480376	2.2764228	2	2	2	99.6	100.4	1.008	
Q15003	Condensin complex subunit 2	82.511	5.06	54.283421	15.249663	11	13	11	99.6	100.4	1.008	1.24E-01
Q15185	Prostaglandin E synthase 3	18.685	4.54	57.651881	41.875	6	13	6	99.6	100.4	1.008	7.96E-01
Q15369	Transcription elongation factor B polypeptide 1	12.465	4.78	55.553975	47.321429	4	13	4	99.6	100.4	1.008	7.06E-01
Q5JRA6	Melanoma inhibitory activity protein 3	213.57	4.84	79.943413	6.5547981	10	13	10	99.6	100.4	1.008	5.64E-01
Q5T4S7	E3 ubiquitin-protein ligase UBR4	573.476	6.04	215.85755	8.0262396	39	48	38	99.6	100.4	1.008	2.49E-01
Q6UB35	Monofunctional C1-tetrahydrofolate synthase, mitochondrial	105.724	8.06	76.0929	15.541922	14	19	14	99.6	100.4	1.008	2.55E-01
Q7KZF4	Staphylococcal nuclease domain-containing protein 1	101.934	7.17	267.47315	35.164835	28	61	28	99.6	100.4	1.008	6.48E-01
Q7Z4W1	L-xylulose reductase	25.897	8.1	16.55355	11.885246	3	3	3	99.6	100.4	1.008	8.41E-01

Q86UU0	B-cell CLL/lymphoma 9-like protein	157.027	8.63	7.3503485	1.7344897	2	2	2	99.6	100.4	1.008	
Q86XL3	Ankyrin repeat and LEM domain-containing protein 2	104.05	7.09	17.640092	4.6908316	3	3	3	99.6	100.4	1.008	7.00E-01
Q8IY17	Neuropathy target esterase	149.9	7.81	22.346753	3.2210835	4	6	4	99.6	100.4	1.008	9.57E-01
Q8N3U4	Cohesin subunit SA-2	141.235	5.43	108.6148	14.297319	17	24	13	99.6	100.4	1.008	4.52E-01
Q8TD16	Protein bicaudal D homolog 2	93.476	5.44	43.552725	12.014563	10	11	9	99.6	100.4	1.008	3.06E-01
Q96F24	Nuclear receptor-binding factor 2	32.358	5.87	11.818726	9.0592334	3	3	2	99.6	100.4	1.008	
Q96L92	Sorting nexin-27	61.226	6.49	23.669936	6.8391867	4	5	4	99.6	100.4	1.008	1.63E-01
Q96P16	Regulation of nuclear pre-mRNA domain-containing protein 1A	35.698	7.55	43.630301	26.282051	7	9	6	99.6	100.4	1.008	2.41E-01
Q99426	Tubulin-folding cofactor B	27.308	5.15	36.856498	29.098361	7	8	7	99.6	100.4	1.008	3.50E-01
Q99459	Cell division cycle 5-like protein	92.194	8.18	153.53837	23.19202	15	23	15	99.6	100.4	1.008	5.81E-01
Q99598	Translin-associated protein X	33.092	6.55	103.36165	44.137931	12	17	12	99.6	100.4	1.008	4.91E-01
Q9BPX3	Condensin complex subunit 3	114.262	5.59	174.31381	21.871921	19	32	18	99.6	100.4	1.008	6.58E-01
Q9BQE3	Tubulin alpha-1C chain	49.863	5.1	324.58912	52.33853	19	123	7	99.6	100.4	1.008	2.80E-01
Q9BRS2	Serine/threonine-protein kinase RIO1	65.542	6.19	19.531799	4.0492958	2	3	2	99.6	100.4	1.008	9.41E-01
Q9BRT2	Ubiquinol-cytochrome-c reductase complex assembly factor 2	14.865	7.37	20.814973	28.571429	3	4	3	99.6	100.4	1.008	2.53E-01
Q9BRX2	Protein pelota homolog	43.332	6.34	42.285543	14.285714	5	7	5	99.6	100.4	1.008	7.08E-01
Q9BUB5	MAP kinase-interacting serine/threonine-protein kinase 1	51.31	6.68	8.7682756	2.5806452	1	1	1	99.6	100.4	1.008	
Q9H2K0	Translation initiation factor IF-3, mitochondrial	31.706	9.69	5.933367	6.1151079	2	2	2	99.6	100.4	1.008	
Q9H3S7	Tyrosine-protein phosphatase non-receptor type 23	178.861	6.92	69.572296	8.801956	12	13	12	99.6	100.4	1.008	3.37E-01
Q9HAB8	Phosphopantothenate--cysteine ligase	33.984	6.71	15.768613	8.3601286	3	3	3	99.6	100.4	1.008	1.94E-01
Q9NR12	PDZ and LIM domain protein 7	49.813	8.41	45.629078	19.256018	8	11	8	99.6	100.4	1.008	6.76E-01
Q9NR30	Nucleolar RNA helicase 2	87.29	9.28	357.78966	40.740741	32	69	30	99.6	100.4	1.008	5.06E-01
Q9NR31	GTP-binding protein SAR1a	22.353	6.68	74.025439	42.424242	7	15	4	99.6	100.4	1.008	3.46E-01
Q9NUJ1	Mycophenolic acid acyl-glucuronide esterase, mitochondrial	33.911	8.57	27.93218	14.052288	4	6	4	99.6	100.4	1.008	9.04E-01
Q9NVG8	TBC1 domain family member 13	46.524	5.24	26.240924	14.25	4	4	4	99.6	100.4	1.008	5.72E-01
Q9NVR2	Integrator complex subunit 10	82.183	7.44	3.4814861	1.2676056	1	1	1	99.6	100.4	1.008	
Q9NY27	Serine/threonine-protein phosphatase 4 regulatory subunit 2	46.869	4.54	43.792788	13.189448	5	8	5	99.6	100.4	1.008	7.59E-01
Q9UBW8	COP9 signalosome complex subunit 7a	30.258	8.22	48.434187	16.727273	5	7	5	99.6	100.4	1.008	9.84E-01
Q9UDY8	Mucosa-associated lymphoid tissue lymphoma translocation protein 1	92.213	5.73	39.305721	6.5533981	5	6	5	99.6	100.4	1.008	6.15E-01
Q9UHW5	GPN-loop GTPase 3	32.74	4.5	6.3237638	4.5774648	1	1	1	99.6	100.4	1.008	
Q9UID3	Vacuolar protein sorting-associated protein 51 homolog	85.988	6.47	18.670397	6.2659847	4	4	4	99.6	100.4	1.008	6.69E-01
Q9UNL2	Translocon-associated protein subunit gamma	21.067	9.61	21.585974	7.5675676	1	3	1	99.6	100.4	1.008	
Q9UNQ2	Probable dimethyladenosine transferase	35.214	9.99	18.263582	13.738019	4	5	4	99.6	100.4	1.008	1.55E-01
Q9UQN3	Charged multivesicular body protein 2b	23.891	8.76	7.7692165	7.5117371	2	3	2	99.6	100.4	1.008	
Q9Y263	Phospholipase A-2-activating protein	87.101	6.37	146.10125	29.685535	19	25	19	99.6	100.4	1.008	8.48E-01
Q9Y2K6	Ubiquitin carboxyl-terminal hydrolase 20	101.938	6.11	2.3161428	0.9846827	1	1	1	99.6	100.4	1.008	
Q9Y5Q8	General transcription factor 3C polypeptide 5	59.533	6.9	12.160301	6.1657033	3	3	3	99.6	100.4	1.008	
O00217	ADH dehydrogenase [ubiquinone] iron-sulfur protein 8, mitochondria	23.69	6.34	34.657908	27.619048	5	8	5	99.5	100.5	1.01	8.33E-01
O14737	Programmed cell death protein 5	14.276	6.04	56.489434	52.8	6	19	6	99.5	100.5	1.01	7.25E-01
O14908	PDZ domain-containing protein GIPC1	36.027	6.28	45.575468	18.918919	5	6	4	99.5	100.5	1.01	5.01E-01
O43663	Protein regulator of cytokinesis 1	71.562	6.68	62.408268	17.903226	10	12	9	99.5	100.5	1.01	1.56E-01
O75127	Pentatricopeptide repeat-containing protein 1, mitochondrial	78.806	8.59	23.010624	8.5714286	5	6	5	99.5	100.5	1.01	8.93E-01
O75477	Erlin-1	38.901	7.87	59.826244	22.254335	8	11	5	99.5	100.5	1.01	3.33E-01
O95232	Luc7-like protein 3	51.435	9.79	64.826149	22.916667	11	16	11	99.5	100.5	1.01	9.64E-01
P06280	Alpha-galactosidase A	48.735	5.6	29.469424	14.918415	5	6	5	99.5	100.5	1.01	2.09E-01
P0CG08	Golgi pH regulator B	52.882	9.28	4.7126462	2.6373626	1	1	1	99.5	100.5	1.01	
P10809	60 kDa heat shock protein, mitochondrial	61.016	5.87	596.17236	72.251309	32	141	32	99.5	100.5	1.01	3.32E-01

P11233	Ras-related protein Ral-A	23.552	7.11	46.925629	23.300971	4	9	3	99.5	100.5	1.01	1.00E+00
P12956	X-ray repair cross-complementing protein 6	69.799	6.64	250.22653	36.945813	22	58	22	99.5	100.5	1.01	9.51E-01
P14678	Small nuclear ribonucleoprotein-associated proteins B and B'	24.594	11.19	125.48118	23.75	7	25	7	99.5	100.5	1.01	9.48E-02
P17174	Aspartate aminotransferase, cytoplasmic	46.219	7.01	126.51741	34.624697	12	27	12	99.5	100.5	1.01	3.55E-01
P19367	Hexokinase-1	102.42	6.8	171.44575	27.699019	24	34	23	99.5	100.5	1.01	3.78E-01
P25208	Nuclear transcription factor Y subunit beta	22.817	4.59	8.2496391	10.144928	2	2	2	99.5	100.5	1.01	8.04E-01
P25398	40S ribosomal protein S12	14.505	7.21	91.591952	53.787879	6	21	6	99.5	100.5	1.01	2.58E-01
P26196	Probable ATP-dependent RNA helicase DDX6	54.382	8.66	117.94016	33.747412	14	25	13	99.5	100.5	1.01	7.09E-01
P26447	Protein S100-A4	11.721	6.11	23.166224	25.742574	4	12	4	99.5	100.5	1.01	8.50E-01
P26641	Elongation factor 1-gamma	50.087	6.67	197.11563	37.299771	19	51	19	99.5	100.5	1.01	2.04E-01
P33527	Multidrug resistance-associated protein 1	171.481	7.11	80.507568	6.5969954	8	11	8	99.5	100.5	1.01	6.01E-01
P35251	Replication factor C subunit 1	128.175	9.36	42.952953	7.5783972	8	10	8	99.5	100.5	1.01	4.65E-01
P41091	Eukaryotic translation initiation factor 2 subunit 3	51.077	8.4	117.27613	31.355932	14	27	14	99.5	100.5	1.01	1.27E-01
P41214	Eukaryotic translation initiation factor 2D	64.666	7.65	18.309451	6.6780822	3	3	3	99.5	100.5	1.01	9.23E-01
P43897	Elongation factor Ts, mitochondrial	35.368	8.38	82.325509	35.384615	10	13	10	99.5	100.5	1.01	1.50E-01
P49815	Tuberin	200.481	7.31	8.3622962	1.2174875	2	2	2	99.5	100.5	1.01	
P51580	Thiopurine S-methyltransferase	28.162	6.23	47.50985	26.122449	7	12	7	99.5	100.5	1.01	7.32E-01
P54289	Voltage-dependent calcium channel subunit alpha-2/delta-1	124.49	5.27	7.569182	2.0852221	2	2	2	99.5	100.5	1.01	
P61077	Ubiquitin-conjugating enzyme E2 D3	16.676	7.8	8.587166	7.4829932	1	2	1	99.5	100.5	1.01	
P62136	Serine/threonine-protein phosphatase PP1-alpha catalytic subunit	37.488	6.33	127.57253	40.30303	11	33	3	99.5	100.5	1.01	7.35E-03
Q04637	Eukaryotic translation initiation factor 4 gamma 1	175.382	5.33	361.03699	27.39212	43	82	38	99.5	100.5	1.01	5.42E-04
Q08379	Golgin subfamily A member 2	113.017	5.02	136.38363	18.363273	15	23	15	99.5	100.5	1.01	3.58E-01
Q08722	Leukocyte surface antigen CD47	35.191	7.21	7.141963	5.8823529	2	3	2	99.5	100.5	1.01	
Q13045	Protein flightless-1 homolog	144.659	6.05	117.30172	12.84476	14	21	14	99.5	100.5	1.01	8.57E-02
Q13620	Cullin-4B	103.916	7.37	99.699476	19.715225	19	25	12	99.5	100.5	1.01	3.82E-03
Q13630	GDP-L-fucose synthase	35.87	6.6	19.168776	8.0996885	2	3	2	99.5	100.5	1.01	
Q15029	116 kDa U5 small nuclear ribonucleoprotein component	109.366	5	230.24484	30.658436	25	46	25	99.5	100.5	1.01	5.82E-01
Q15417	Calponin-3	36.391	6.05	61.262477	21.276596	6	15	4	99.5	100.5	1.01	2.80E-01
Q15437	Protein transport protein Sec23B	86.424	6.89	77.31791	16.036506	10	19	8	99.5	100.5	1.01	2.42E-02
Q15836	Vesicle-associated membrane protein 3	11.302	8.79	32.64166	40	3	4	3	99.5	100.5	1.01	8.65E-01
Q16718	NADH dehydrogenase [ubiquinone] 1 alpha subcomplex subunit 5	13.45	5.99	41.666105	43.103448	4	8	4	99.5	100.5	1.01	7.97E-01
Q5T0N5	Formin-binding protein 1-like	70.021	6.64	45.980938	17.190083	10	10	10	99.5	100.5	1.01	2.00E-01
Q5W0B1	RING finger protein 219	81.066	5.72	9.6631402	2.3415978	1	1	1	99.5	100.5	1.01	
Q6DD88	Atlastin-3	60.503	5.66	112.61744	20.51756	10	29	10	99.5	100.5	1.01	7.24E-02
Q6P4E1	Protein CASC4	48.835	5.82	13.334482	5.5427252	2	2	2	99.5	100.5	1.01	
Q8N3C0	Activating signal cointegrator 1 complex subunit 3	251.301	7.09	37.339278	3.9055404	8	9	8	99.5	100.5	1.01	4.97E-01
Q8NC51	Plasminogen activator inhibitor 1 RNA-binding protein	44.938	8.65	177.78632	39.705882	17	43	17	99.5	100.5	1.01	5.53E-01
Q92615	La-related protein 4B	80.503	6.92	15.310657	3.7940379	3	4	3	99.5	100.5	1.01	5.14E-01
Q92890	Ubiquitin fusion degradation protein 1 homolog	34.478	6.7	39.055317	18.892508	5	11	5	99.5	100.5	1.01	4.04E-01
Q96ER3	Protein SAAL1	53.524	4.5	23.86331	10.548523	5	5	5	99.5	100.5	1.01	4.90E-01
Q96P11	Probable 28S rRNA (cytosine-C(5))-methyltransferase	46.662	8.62	38.162023	13.752914	4	5	4	99.5	100.5	1.01	6.28E-01
Q96QC0	Serine/threonine-protein phosphatase 1 regulatory subunit 10	98.996	9.17	21.58043	5.9574468	6	7	6	99.5	100.5	1.01	7.85E-01
Q99733	Nucleosome assembly protein 1-like 4	42.797	4.69	65.906857	26.133333	9	16	8	99.5	100.5	1.01	1.32E-01
Q99996	A-kinase anchor protein 9	453.387	4.98	56.116058	2.4546152	8	10	8	99.5	100.5	1.01	3.36E-01
Q9BSD7	Cancer-related nucleoside-triphosphatase	20.7	9.54	38.526294	34.210526	5	8	5	99.5	100.5	1.01	1.39E-01
Q9BT22	Chitobiosyldiphosphodolichol beta-mannosyltransferase	52.484	7.23	7.7910161	5.1724138	3	3	3	99.5	100.5	1.01	5.41E-01
Q9BTE1	Dynactin subunit 5	20.113	8.02	19.035866	15.384615	3	5	3	99.5	100.5	1.01	7.77E-01

Q9BTE7	DCN1-like protein 5	27.491	5.58	14.603645	8.4388186	2	3	2	99.5	100.5	1.01	
Q9H0E9	Bromodomain-containing protein 8	135.251	4.6	19.277014	2.6720648	3	5	3	99.5	100.5	1.01	9.64E-01
Q9H9A6	Leucine-rich repeat-containing protein 40	68.207	6.43	28.712299	11.79402	7	8	7	99.5	100.5	1.01	3.42E-01
Q9H9L3	Interferon-stimulated 20 kDa exonuclease-like 2	39.13	9.94	15.781949	3.6827195	1	2	1	99.5	100.5	1.01	
Q9HB71	Calcyclin-binding protein	26.194	8.25	97.070507	44.298246	9	21	9	99.5	100.5	1.01	5.28E-01
Q9HD67	Unconventional myosin-X	237.198	6.21	22.445724	2.5753158	4	4	4	99.5	100.5	1.01	1.88E-01
Q9NNW7	Thioredoxin reductase 2, mitochondrial	56.472	7.5	37.004785	13.358779	6	7	6	99.5	100.5	1.01	7.01E-01
Q9NUQ6	SPATS2-like protein	61.691	9.64	15.695012	9.1397849	4	4	4	99.5	100.5	1.01	2.58E-01
Q9NYK5	39S ribosomal protein L39, mitochondrial	38.687	7.65	44.761634	16.863905	5	7	5	99.5	100.5	1.01	7.05E-02
Q9NYV4	Cyclin-dependent kinase 12	164.054	9.44	33.398613	5.1677852	5	5	4	99.5	100.5	1.01	4.67E-01
Q9P209	Centrosomal protein of 72 kDa	71.673	6.52	8.0820051	1.5455951	1	2	1	99.5	100.5	1.01	
Q9P2J5	Leucine--tRNA ligase, cytoplasmic	134.379	7.3	209.33711	24.489796	25	35	25	99.5	100.5	1.01	5.89E-01
Q9UBI1	COMM domain-containing protein 3	22.137	5.99	49.412799	35.384615	5	6	5	99.5	100.5	1.01	3.90E-01
Q9UHG3	Prenylcysteine oxidase 1	56.604	6.18	29.29932	7.3267327	3	4	3	99.5	100.5	1.01	7.08E-01
Q9UI12	V-type proton ATPase subunit H	55.847	6.48	57.212989	14.492754	4	7	4	99.5	100.5	1.01	3.45E-01
Q9UKM9	RNA-binding protein Raly	32.444	9.17	83.420699	32.352941	11	15	11	99.5	100.5	1.01	1.92E-02
Q9Y223	ctional UDP-N-acetylglucosamine 2-epimerase/N-acetylmannosamine	79.224	6.8	43.253045	9.4182825	6	9	6	99.5	100.5	1.01	7.09E-01
Q9Y2D4	Exocyst complex component 6B	94.141	6.46	36.852099	7.2749692	5	8	5	99.5	100.5	1.01	2.34E-01
Q9Y3C8	Ubiquitin-fold modifier-conjugating enzyme 1	19.446	7.4	27.505107	23.353293	5	11	5	99.5	100.5	1.01	7.76E-01
Q9Y4C8	Probable RNA-binding protein 19	107.265	6.54	36.663663	7.0833333	6	7	6	99.5	100.5	1.01	7.63E-02
Q9Y6W5	Wiskott-Aldrich syndrome protein family member 2	54.25	5.53	26.519947	10.843373	5	6	5	99.5	100.5	1.01	2.58E-01
A2RRP1	Neuroblastoma-amplified sequence	268.401	5.96	43.154867	3.6693378	7	8	7	99.4	100.6	1.012	1.00E+00
A5YKK6	CCR4-NOT transcription complex subunit 1	266.768	7.11	154.48918	11.826599	25	29	25	99.4	100.6	1.012	3.78E-01
A6NHR9	ıral maintenance of chromosomes flexible hinge domain-containing pr	226.231	7.3	14.579094	1.7955112	4	4	4	99.4	100.6	1.012	6.29E-01
O00567	Nucleolar protein 56	66.009	9.19	133.70806	35.353535	17	27	17	99.4	100.6	1.012	4.88E-01
O14548	Cytochrome c oxidase subunit 7A-related protein, mitochondrial	12.607	9.42	21.182069	26.315789	3	4	3	99.4	100.6	1.012	8.15E-01
O14618	Copper chaperone for superoxide dismutase	29.022	5.58	15.303568	11.313869	3	3	3	99.4	100.6	1.012	3.49E-01
O14686	Histone-lysine N-methyltransferase 2D	593.017	5.58	2.4205594	0.1444826	1	1	1	99.4	100.6	1.012	
O14818	Proteasome subunit alpha type-7	27.87	8.46	172.53745	50.403226	14	38	14	99.4	100.6	1.012	5.50E-01
O15381	Nuclear valosin-containing protein-like	94.991	6.48	67.587644	13.317757	9	14	9	99.4	100.6	1.012	5.98E-02
O60313	Dynamin-like 120 kDa protein, mitochondrial	111.561	7.87	148.32339	21.354167	19	24	19	99.4	100.6	1.012	1.69E-01
O75340	Programmed cell death protein 6	21.855	5.4	39.344833	32.984293	6	9	6	99.4	100.6	1.012	2.88E-01
O95140	Mitofusin-2	86.347	6.98	69.03774	10.700132	7	10	7	99.4	100.6	1.012	9.27E-01
O95757	Heat shock 70 kDa protein 4L	94.453	5.88	170.64362	28.963051	22	38	18	99.4	100.6	1.012	4.55E-01
O95870	Abhydrolase domain-containing protein 16A	63.203	8.13	17.320092	3.046595	2	2	2	99.4	100.6	1.012	
P07099	Epoxide hydrolase 1	52.915	7.25	55.749935	15.824176	8	14	8	99.4	100.6	1.012	4.02E-02
P16070	CD44 antigen	81.487	5.33	52.602971	7.9514825	7	15	7	99.4	100.6	1.012	5.47E-01
P16220	Cyclic AMP-responsive element-binding protein 1	36.666	5.57	15.749453	8.797654	3	5	3	99.4	100.6	1.012	9.40E-01
P19338	Nucleolin	76.568	4.7	282.56133	38.169014	32	84	32	99.4	100.6	1.012	2.01E-01
P19525	Interferon-induced, double-stranded RNA-activated protein kinase	62.056	8.4	59.929188	19.963702	11	14	11	99.4	100.6	1.012	1.37E-01
P20020	Plasma membrane calcium-transporting ATPase 1	138.668	6.04	104.64949	15.500795	16	21	8	99.4	100.6	1.012	1.11E-01
P20810	Calpastatin	76.526	5.07	151.89081	32.485876	15	23	15	99.4	100.6	1.012	2.27E-01
P30622	CAP-Gly domain-containing linker protein 1	162.147	5.36	365.95968	33.588317	48	68	41	99.4	100.6	1.012	4.33E-02
P31689	DnaJ homolog subfamily A member 1	44.839	7.08	72.711529	26.700252	10	18	9	99.4	100.6	1.012	2.64E-01
P36405	ADP-ribosylation factor-like protein 3	20.443	7.24	55.253241	43.956044	7	10	7	99.4	100.6	1.012	9.87E-02
P43307	Translocon-associated protein subunit alpha	32.215	4.49	39.400168	17.132867	3	8	3	99.4	100.6	1.012	7.66E-01
P46782	40S ribosomal protein S5	22.862	9.72	58.986557	27.941176	7	18	7	99.4	100.6	1.012	4.16E-01

P48556	26S proteasome non-ATPase regulatory subunit 8	39.587	9.7	38.318555	16	6	10	6	99.4	100.6	1.012	6.05E-01
P48681	Nestin	177.332	4.36	25.241275	3.0845157	5	5	5	99.4	100.6	1.012	2.85E-01
P50502	Hsc70-interacting protein	41.305	5.27	142.89599	31.165312	12	36	12	99.4	100.6	1.012	9.64E-01
P51149	Ras-related protein Rab-7a	23.475	6.7	153.04279	70.048309	13	31	13	99.4	100.6	1.012	5.43E-01
P53992	Protein transport protein Sec24C	118.249	7.06	115.64996	14.351005	15	25	13	99.4	100.6	1.012	6.99E-01
P54920	Alpha-soluble NSF attachment protein	33.211	5.36	75.785787	31.186441	7	12	7	99.4	100.6	1.012	2.19E-01
P55199	RNA polymerase II elongation factor ELL	68.223	9.33	4.6289321	2.0933977	1	1	1	99.4	100.6	1.012	
P57740	Nuclear pore complex protein Nup107	106.307	5.43	57.493888	10.918919	9	10	9	99.4	100.6	1.012	3.32E-01
P61158	Actin-related protein 3	47.341	5.88	140.23326	34.210526	13	27	13	99.4	100.6	1.012	6.65E-01
P61247	40S ribosomal protein S3a	29.926	9.73	125.08762	49.621212	15	37	15	99.4	100.6	1.012	7.36E-01
P61970	Nuclear transport factor 2	14.469	5.38	70.066509	34.645669	4	17	3	99.4	100.6	1.012	1.83E-01
P62487	DNA-directed RNA polymerase II subunit RPB7	19.282	5.54	11.347578	18.604651	3	3	3	99.4	100.6	1.012	9.32E-01
P78316	Nucleolar protein 14	97.607	7.58	48.527915	7.8179697	7	11	7	99.4	100.6	1.012	1.94E-01
P78563	Double-stranded RNA-specific editase 1	80.713	9.01	20.740466	7.0175439	5	5	5	99.4	100.6	1.012	5.66E-01
P82650	28S ribosomal protein S22, mitochondrial	41.254	7.9	65.467041	23.333333	8	11	8	99.4	100.6	1.012	2.16E-01
P83436	Conserved oligomeric Golgi complex subunit 7	86.289	5.47	32.400368	8.0519481	6	7	6	99.4	100.6	1.012	4.51E-01
Q01780	Exosome component 10	100.768	8.46	120.72699	22.146893	15	20	15	99.4	100.6	1.012	7.23E-01
Q02388	Collagen alpha-1(VII) chain	295.041	6.27	4.7336296	0.78125	2	2	2	99.4	100.6	1.012	
Q08211	ATP-dependent RNA helicase A	140.869	6.84	392.12654	29.685039	36	105	36	99.4	100.6	1.012	4.06E-02
Q0JRZ9	F-BAR domain only protein 2	88.869	6.86	27.763446	6.9135802	5	5	4	99.4	100.6	1.012	5.57E-01
Q12904	.minoacyl tRNA synthase complex-interacting multifunctional protein	34.331	8.43	136.83974	47.115385	14	29	13	99.4	100.6	1.012	2.47E-01
Q12972	Nuclear inhibitor of protein phosphatase 1	38.455	7.37	53.882862	18.518519	4	7	4	99.4	100.6	1.012	5.68E-02
Q13451	Peptidyl-prolyl cis-trans isomerase FKBP5	51.18	5.9	36.784798	12.035011	5	7	4	99.4	100.6	1.012	4.07E-01
Q14011	Cold-inducible RNA-binding protein	18.637	9.51	16.06286	14.534884	2	2	2	99.4	100.6	1.012	
Q14160	Protein scribble homolog	174.778	5.07	42.909187	5.5214724	8	13	8	99.4	100.6	1.012	2.59E-01
Q15631	Translin	26.167	6.44	50.426945	24.122807	6	10	6	99.4	100.6	1.012	1.25E-02
Q15691	Microtubule-associated protein RP/EB family member 1	29.98	5.14	90.95322	37.686567	11	21	10	99.4	100.6	1.012	5.03E-02
Q2M2I8	AP2-associated protein kinase 1	103.821	6.6	40.944039	8.6368366	7	7	7	99.4	100.6	1.012	8.01E-01
Q4LE39	AT-rich interactive domain-containing protein 4B	147.719	5.12	26.552024	2.8963415	3	4	3	99.4	100.6	1.012	
Q53T59	HCLS1-binding protein 3	42.754	5.01	27.129197	7.1428571	2	3	2	99.4	100.6	1.012	7.83E-01
Q6NXT1	Ankyrin repeat domain-containing protein 54	32.485	6.28	15.488746	9.6666667	3	4	3	99.4	100.6	1.012	3.49E-02
Q6P1Q9	Methyltransferase-like protein 2B	43.398	5.86	28.740264	12.169312	6	7	6	99.4	100.6	1.012	6.48E-01
Q6ZSJ8	Uncharacterized protein C1orf122	11.464	6.73	5.1803903	10	1	1	1	99.4	100.6	1.012	
Q7Z3C6	Autophagy-related protein 9A	94.387	6.67	17.720996	3.2181168	3	3	3	99.4	100.6	1.012	
Q8IZL8	Proline-, glutamic acid- and leucine-rich protein 1	119.624	4.34	76.375102	12.123894	10	13	10	99.4	100.6	1.012	1.50E-01
Q8N201	Integrator complex subunit 1	244.143	6.13	25.267144	3.0593607	7	7	7	99.4	100.6	1.012	3.89E-01
Q8N8A6	ATP-dependent RNA helicase DDX51	72.413	8.16	21.482187	6.7567568	3	3	3	99.4	100.6	1.012	2.93E-01
Q8NF91	Nesprin-1	1010.456	5.53	31.95721	0.6820507	6	7	4	99.4	100.6	1.012	8.88E-02
Q8WU90	Zinc finger CCCH domain-containing protein 15	48.573	5.31	55.152316	21.126761	9	10	9	99.4	100.6	1.012	3.87E-01
Q92572	AP-3 complex subunit sigma-1	21.718	5.39	6.7796304	5.6994819	1	1	1	99.4	100.6	1.012	
Q92882	Osteoclast-stimulating factor 1	23.772	5.68	20.311879	8.8785047	2	3	2	99.4	100.6	1.012	
Q92917	G patch domain and KOW motifs-containing protein	52.197	6.15	68.681933	24.789916	8	11	8	99.4	100.6	1.012	3.75E-01
Q96EI5	Transcription elongation factor A protein-like 4	24.632	5.2	22.585254	18.604651	3	4	3	99.4	100.6	1.012	7.64E-01
Q96KP4	Cytosolic non-specific dipeptidase	52.845	5.97	90.450719	25.263158	8	13	8	99.4	100.6	1.012	5.10E-01
Q96KQ7	Histone-lysine N-methyltransferase EHMT2	132.287	5.45	21.626177	4.4628099	5	5	5	99.4	100.6	1.012	
Q9BXP5	Serrate RNA effector molecule homolog	100.604	5.96	129.63994	21.461187	17	27	17	99.4	100.6	1.012	8.15E-01
Q9BYJ9	YTH domain-containing family protein 1	60.836	8.79	19.563606	4.8300537	3	4	1	99.4	100.6	1.012	

Q9H0D6	5'-3' exoribonuclease 2	108.513	7.47	79.468587	13.263158	9	12	9	99.4	100.6	1.012	1.41E-01
Q9H2M9	Rab3 GTPase-activating protein non-catalytic subunit	155.886	5.62	58.576766	9.4759512	13	18	11	99.4	100.6	1.012	1.01E-01
Q9H3U5	Major facilitator superfamily domain-containing protein 1	51.174	6.74	6.0032257	2.5806452	1	1	1	99.4	100.6	1.012	
Q9NQR4	Omega-amidase NIT2	30.589	7.21	124.8915	47.101449	12	20	12	99.4	100.6	1.012	3.24E-01
Q9NS87	Kinesin-like protein KIF15	160.061	6	65.34662	6.4841499	9	12	9	99.4	100.6	1.012	6.17E-01
Q9NVA2	Septin-11	49.367	6.81	142.29396	29.83683	12	23	4	99.4	100.6	1.012	8.36E-01
Q9NVH0	Exonuclease 3'-5' domain-containing protein 2	70.308	8.32	17.913136	5.6360709	3	3	3	99.4	100.6	1.012	3.85E-02
Q9UBU8	Mortality factor 4-like protein 1	41.448	9.28	14.354991	9.6685083	3	3	3	99.4	100.6	1.012	3.41E-01
Q9UHY7	Enolase-phosphatase E1	28.914	4.78	32.600681	14.942529	4	6	4	99.4	100.6	1.012	7.73E-01
Q9UKV8	Protein argonaute-2	97.146	9.19	20.027997	5.4714785	4	4	4	99.4	100.6	1.012	8.10E-01
Q9UKZ1	CCR4-NOT transcription complex subunit 11	55.18	6.4	17.786839	3.9215686	2	3	2	99.4	100.6	1.012	7.83E-01
Q9UMR2	ATP-dependent RNA helicase DDX19B	53.893	6.3	91.325134	26.722338	9	16	9	99.4	100.6	1.012	4.91E-02
Q9Y3L5	Ras-related protein Rap-2c	20.731	4.94	40.992948	26.229508	4	7	3	99.4	100.6	1.012	5.32E-01
Q9Y6R0	Numb-like protein	64.851	8.85	34.335613	14.285714	7	8	6	99.4	100.6	1.012	4.16E-02
O00161	Synaptosomal-associated protein 23	23.34	5.01	67.445573	34.123223	5	8	5	99.3	100.7	1.014	5.63E-01
O14578	Citron Rho-interacting kinase	231.286	6.57	8.9431036	0.8386778	2	2	2	99.3	100.7	1.014	
O14772	Fucose-1-phosphate guanylyltransferase	66.556	6.48	8.3447655	2.6936027	1	1	1	99.3	100.7	1.014	
O43264	Centromere/kinetochore protein zw10 homolog	88.773	6.27	61.729451	13.478819	10	13	10	99.3	100.7	1.014	1.96E-02
O43488	Aflatoxin B1 aldehyde reductase member 2	39.564	7.17	34.200023	14.206128	6	13	5	99.3	100.7	1.014	4.57E-01
O43633	Charged multivesicular body protein 2a	25.088	5.97	12.250086	7.6576577	2	3	2	99.3	100.7	1.014	3.64E-01
O43715	TP53-regulated inhibitor of apoptosis 1	8.78	5.48	15.178294	44.736842	3	4	3	99.3	100.7	1.014	
O43865	Adenosylhomocysteinase 2	58.913	6.89	78.768748	22.075472	11	17	4	99.3	100.7	1.014	9.67E-01
O60884	DnaJ homolog subfamily A member 2	45.717	6.48	84.890364	26.456311	9	18	9	99.3	100.7	1.014	2.36E-01
O75351	Vacuolar protein sorting-associated protein 4B	49.271	7.23	46.161221	13.063063	5	9	2	99.3	100.7	1.014	
O75400	Pre-mRNA-processing factor 40 homolog A	108.737	7.56	44.890592	7.4190178	7	10	7	99.3	100.7	1.014	6.89E-01
O95336	6-phosphogluconolactonase	27.53	6.05	68.965811	29.457364	6	16	6	99.3	100.7	1.014	5.74E-01
P02794	Ferritin heavy chain	21.212	5.55	35.40994	31.147541	4	6	4	99.3	100.7	1.014	8.13E-01
P04424	Argininosuccinate lyase	51.625	6.48	3.7164292	3.6637931	2	2	2	99.3	100.7	1.014	
P05388	60S acidic ribosomal protein P0	34.252	5.97	164.17051	48.264984	12	40	12	99.3	100.7	1.014	6.31E-02
P07602	Prosaposin	58.074	5.17	80.490733	20.992366	11	20	11	99.3	100.7	1.014	2.12E-01
P12277	Creatine kinase B-type	42.617	5.59	242.51978	55.905512	15	62	14	99.3	100.7	1.014	6.42E-01
P18669	Phosphoglycerate mutase 1	28.786	7.18	210.21984	56.299213	13	54	13	99.3	100.7	1.014	2.76E-03
P23921	Ribonucleoside-diphosphate reductase large subunit	90.013	7.15	238.65187	32.70202	25	51	25	99.3	100.7	1.014	4.91E-01
P28290	Sperm-specific antigen 2	138.3	5.19	26.316382	3.1771247	4	5	4	99.3	100.7	1.014	7.41E-01
P30050	60S ribosomal protein L12	17.808	9.42	105.74772	62.424242	8	27	8	99.3	100.7	1.014	5.58E-01
P31946	14-3-3 protein beta/alpha	28.065	4.83	190.71884	58.943089	15	106	6	99.3	100.7	1.014	4.39E-02
P48047	ATP synthase subunit O, mitochondrial	23.263	9.96	67.606126	43.661972	9	15	9	99.3	100.7	1.014	7.53E-01
P49368	T-complex protein 1 subunit gamma	60.495	6.49	300.01165	51.743119	29	81	29	99.3	100.7	1.014	1.39E-01
P53367	Arfaptin-1	41.713	6.7	21.153367	8.847185	4	5	4	99.3	100.7	1.014	4.57E-01
P54886	Delta-1-pyrroline-5-carboxylate synthase	87.248	7.12	156.38109	33.081761	22	36	22	99.3	100.7	1.014	4.00E-02
P55769	NHP2-like protein 1	14.165	8.46	33.558098	31.25	3	7	3	99.3	100.7	1.014	3.96E-01
P61026	Ras-related protein Rab-10	22.527	8.38	51.975406	36.5	8	30	5	99.3	100.7	1.014	8.60E-01
P67870	Casein kinase II subunit beta	24.926	5.55	60.386272	37.209302	6	12	6	99.3	100.7	1.014	9.07E-01
Q06265	Exosome complex component RRP45	48.918	5.29	31.142951	9.3394077	4	5	4	99.3	100.7	1.014	2.64E-01
Q12789	General transcription factor 3C polypeptide 1	238.725	7.3	44.704567	4.7889995	8	10	8	99.3	100.7	1.014	2.55E-01
Q12874	Splicing factor 3A subunit 3	58.812	5.38	47.142026	12.774451	7	12	7	99.3	100.7	1.014	8.27E-01
Q13404	Ubiquitin-conjugating enzyme E2 variant 1	16.484	7.93	42.375618	43.537415	6	12	2	99.3	100.7	1.014	

Q13405	39S ribosomal protein L49, mitochondrial	19.186	9.45	52.905272	38.554217	6	14	6	99.3	100.7	1.014	3.61E-01
Q13564	NEDD8-activating enzyme E1 regulatory subunit	60.209	5.4	77.447904	19.101124	7	13	7	99.3	100.7	1.014	7.44E-01
Q15048	Leucine-rich repeat-containing protein 14	54.478	6.9	3.429107	1.6227181	1	1	1	99.3	100.7	1.014	
Q15418	Ribosomal protein S6 kinase alpha-1	82.671	7.83	64.082868	15.646259	11	13	6	99.3	100.7	1.014	5.04E-01
Q15814	Tubulin-specific chaperone C	39.224	5.71	7.0252091	8.3815029	2	2	2	99.3	100.7	1.014	5.53E-01
Q16531	DNA damage-binding protein 1	126.887	5.26	209.40946	24.035088	25	55	25	99.3	100.7	1.014	4.11E-01
Q2TAA2	Isoamyl acetate-hydrolyzing esterase 1 homolog	27.581	5.3	15.510177	12.903226	2	3	2	99.3	100.7	1.014	
Q7KZN9	Cytochrome c oxidase assembly protein COX15 homolog	46	9.82	19.797933	9.5121951	3	3	3	99.3	100.7	1.014	9.15E-01
Q7Z434	Mitochondrial antiviral-signaling protein	56.493	5.52	8.9654499	4.0740741	2	3	2	99.3	100.7	1.014	5.14E-01
Q7Z4G1	COMM domain-containing protein 6	9.632	6	10.56921	34.117647	3	4	3	99.3	100.7	1.014	8.11E-01
Q86TM6	E3 ubiquitin-protein ligase synoviolin	67.64	6.95	11.646945	4.2139384	3	3	3	99.3	100.7	1.014	4.74E-01
Q86VM9	Zinc finger CCCH domain-containing protein 18	106.315	8.32	18.921836	4.09234	4	4	4	99.3	100.7	1.014	5.88E-01
Q8IX12	Cell division cycle and apoptosis regulator protein 1	132.739	5.76	96.417951	12.956522	13	22	13	99.3	100.7	1.014	2.70E-01
Q8N5M9	Protein jagunal homolog 1	21.111	9.73	13.597617	13.114754	2	4	2	99.3	100.7	1.014	8.09E-02
Q8NBP0	Tetratricopeptide repeat protein 13	96.751	7.01	7.2469534	1.627907	1	1	1	99.3	100.7	1.014	
Q8NHZ8	Anaphase-promoting complex subunit CDC26	9.771	6.81	11.252023	12.941176	1	2	1	99.3	100.7	1.014	
Q8TAT6	Nuclear protein localization protein 4 homolog	68.077	6.38	96.767579	18.914474	11	16	10	99.3	100.7	1.014	5.47E-01
Q8WUA2	Peptidyl-prolyl cis-trans isomerase-like 4	57.189	5.92	10.685475	5.0813008	2	2	2	99.3	100.7	1.014	1.06E-01
Q92922	SWI/SNF complex subunit SMARCC1	122.79	5.76	95.876118	13.936652	14	22	9	99.3	100.7	1.014	2.64E-02
Q969V3	Nicalin	62.935	6.89	71.221006	16.51865	7	12	7	99.3	100.7	1.014	8.29E-01
Q969Z0	Protein TBRG4	70.693	7.42	20.15642	4.7543582	4	6	4	99.3	100.7	1.014	1.06E-01
Q96HA1	Nuclear envelope pore membrane protein POM 121	127.642	10.56	14.298604	2.9623699	2	2	2	99.3	100.7	1.014	
Q96S52	GPI transamidase component PIG-S	61.617	6.49	38.0333	13.693694	7	7	7	99.3	100.7	1.014	3.36E-01
Q99873	Protein arginine N-methyltransferase 1	41.489	5.43	116.71614	33.240997	11	23	11	99.3	100.7	1.014	8.16E-01
Q9BQ52	Zinc phosphodiesterase ELAC protein 2	92.16	7.9	81.706514	14.40678	12	16	12	99.3	100.7	1.014	3.45E-01
Q9BRJ6	Uncharacterized protein C7orf50	22.07	9.64	52.068759	27.835052	5	8	5	99.3	100.7	1.014	6.59E-02
Q9BW83	Intraflagellar transport protein 27 homolog	20.467	5.41	19.53443	19.354839	3	3	3	99.3	100.7	1.014	
Q9C0I1	Myotubularin-related protein 12	86.093	6.62	34.934136	7.3627845	4	4	4	99.3	100.7	1.014	4.89E-01
Q9H0C8	Integrin-linked kinase-associated serine/threonine phosphatase 2C	42.88	7.09	21.67318	9.9489796	4	5	4	99.3	100.7	1.014	2.93E-01
Q9H773	dCTP pyrophosphatase 1	18.669	5.03	43.716505	28.235294	6	11	6	99.3	100.7	1.014	6.99E-01
Q9HD33	39S ribosomal protein L47, mitochondrial	29.432	10.37	24.363575	12.4	3	4	3	99.3	100.7	1.014	2.32E-01
Q9NUL7	Probable ATP-dependent RNA helicase DDX28	59.545	10.42	13.113628	7.2222222	3	3	3	99.3	100.7	1.014	
Q9NY12	H/ACA ribonucleoprotein complex subunit 1	22.334	10.92	27.152121	15.668203	3	8	3	99.3	100.7	1.014	4.56E-02
Q9NY33	Dipeptidyl peptidase 3	82.538	5.1	125.02258	22.38806	13	21	13	99.3	100.7	1.014	1.33E-01
Q9P289	Serine/threonine-protein kinase 26	46.5	5.29	107.30916	25.240385	9	18	6	99.3	100.7	1.014	1.43E-02
Q9P2R3	Rabankyrin-5	128.318	6.1	80.987461	11.976048	12	16	12	99.3	100.7	1.014	1.52E-02
Q9UJ70	N-acetyl-D-glucosamine kinase	37.352	6.24	54.660113	25	8	9	8	99.3	100.7	1.014	4.62E-01
Q9UJS0	Calcium-binding mitochondrial carrier protein Aralar2	74.129	8.62	184.86359	30.962963	18	32	12	99.3	100.7	1.014	5.56E-01
Q9UL15	BAG family molecular chaperone regulator 5	51.168	6.05	44.492169	17.225951	6	9	6	99.3	100.7	1.014	2.88E-01
Q9Y3B3	Transmembrane emp24 domain-containing protein 7	25.156	6.89	5.040577	4.9107143	1	1	1	99.3	100.7	1.014	
O00232	26S proteasome non-ATPase regulatory subunit 12	52.871	7.65	96.81152	33.552632	14	25	13	99.2	100.8	1.016	9.26E-01
O15269	Serine palmitoyltransferase 1	52.71	6.01	48.50646	15.010571	6	9	6	99.2	100.8	1.016	9.62E-02
O43175	D-3-phosphoglycerate dehydrogenase	56.614	6.71	190.65924	36.022514	17	36	17	99.2	100.8	1.016	4.55E-01
O43818	U3 small nucleolar RNA-interacting protein 2	51.809	7.85	34.2441	16.842105	7	9	7	99.2	100.8	1.016	5.78E-01
O60234	Glia maturation factor gamma	16.79	5.26	14.343509	13.380282	2	3	1	99.2	100.8	1.016	
O60353	Frizzled-6	79.24	7.96	10.966548	2.6912181	2	3	2	99.2	100.8	1.016	
O60701	UDP-glucose 6-dehydrogenase	54.989	7.12	175.12004	37.246964	16	32	16	99.2	100.8	1.016	7.00E-02

O75312	Zinc finger protein ZPR1	50.893	4.73	62.541163	19.172113	8	12	8	99.2	100.8	1.016	7.18E-02
O95900	Probable tRNA pseudouridine synthase 2	36.671	8.98	6.5115903	2.7190332	1	1	1	99.2	100.8	1.016	
O96000	NADH dehydrogenase [ubiquinone] 1 beta subcomplex subunit 10	20.763	8.48	39.971893	35.465116	5	6	5	99.2	100.8	1.016	1.89E-01
P04181	Ornithine aminotransferase, mitochondrial	48.504	7.03	57.083547	19.817768	7	11	7	99.2	100.8	1.016	6.05E-01
P08579	U2 small nuclear ribonucleoprotein B"	25.47	9.72	60.789865	34.666667	8	13	6	99.2	100.8	1.016	7.31E-02
P16989	Y-box-binding protein 3	40.066	9.77	58.774422	22.580645	5	13	4	99.2	100.8	1.016	7.72E-01
P17812	CTP synthase 1	66.648	6.46	161.62257	36.209814	19	33	17	99.2	100.8	1.016	2.46E-02
P18085	ADP-ribosylation factor 4	20.498	7.14	87.856819	40.555556	7	23	5	99.2	100.8	1.016	2.18E-01
P20674	Cytochrome c oxidase subunit 5A, mitochondrial	16.752	6.79	46.604291	26.666667	6	16	5	99.2	100.8	1.016	3.56E-01
P23396	40S ribosomal protein S3	26.671	9.66	160.19773	76.954733	18	56	18	99.2	100.8	1.016	2.71E-02
P25205	DNA replication licensing factor MCM3	90.924	5.77	265.74452	33.168317	24	54	24	99.2	100.8	1.016	1.34E-01
P36578	60S ribosomal protein L4	47.667	11.06	120.88781	33.489461	14	33	14	99.2	100.8	1.016	1.19E-02
P42224	Signal transducer and activator of transcription 1-alpha/beta	87.28	6.05	112.98608	19.2	12	20	12	99.2	100.8	1.016	1.93E-01
P50443	Sulfate transporter	81.609	8.38	4.8343569	1.082544	1	2	1	99.2	100.8	1.016	
P53680	AP-2 complex subunit sigma	17.007	6.18	10.705287	11.971831	2	4	2	99.2	100.8	1.016	8.11E-01
P54725	UV excision repair protein RAD23 homolog A	39.585	4.58	28.95884	13.498623	5	7	2	99.2	100.8	1.016	5.07E-01
P62993	Growth factor receptor-bound protein 2	25.19	6.32	74.078793	45.62212	9	14	9	99.2	100.8	1.016	4.73E-01
Q00341	Vigilin	141.368	6.87	376.35893	31.388013	37	70	37	99.2	100.8	1.016	1.70E-02
Q00403	Transcription initiation factor IIB	34.811	8.35	54.488925	28.481013	6	8	6	99.2	100.8	1.016	9.73E-01
Q08170	Serine/arginine-rich splicing factor 4	56.645	11.52	52.179322	18.218623	9	15	4	99.2	100.8	1.016	2.77E-01
Q08945	FACT complex subunit SSRP1	81.024	6.87	107.86639	21.297602	16	29	16	99.2	100.8	1.016	6.61E-02
Q13011	Delta(3,5)-Delta(2,4)-dienoyl-CoA isomerase, mitochondrial	35.793	8	71.348317	33.536585	10	19	10	99.2	100.8	1.016	7.86E-02
Q13616	Cullin-1	89.622	8	32.834817	7.0876289	5	6	5	99.2	100.8	1.016	1.54E-01
Q15007	Pre-mRNA-splicing regulator WTAP	44.217	5.19	25.017241	14.89899	5	5	5	99.2	100.8	1.016	5.63E-01
Q15293	Reticulocalbin-1	38.866	5	102.66351	26.283988	8	23	8	99.2	100.8	1.016	1.82E-01
Q15746	Myosin light chain kinase, smooth muscle	210.583	6.15	12.904137	2.0898642	4	4	4	99.2	100.8	1.016	
Q15785	Mitochondrial import receptor subunit TOM34	34.538	8.98	80.254813	43.68932	11	14	11	99.2	100.8	1.016	2.80E-02
Q16513	Serine/threonine-protein kinase N2	111.964	6.3	115.12398	20.121951	16	21	15	99.2	100.8	1.016	4.02E-02
Q16740	ATP-dependent Clp protease proteolytic subunit, mitochondrial	30.161	8.09	64.958269	24.187726	5	11	5	99.2	100.8	1.016	2.40E-01
Q29963	HLA class I histocompatibility antigen, Cw-6 alpha chain	40.943	6	88.216006	26.229508	7	13	2	99.2	100.8	1.016	2.26E-01
Q5JPH6	Probable glutamate--tRNA ligase, mitochondrial	58.652	8.76	24.48517	8.0305927	5	6	5	99.2	100.8	1.016	4.91E-01
Q5MIZ7	Serine/threonine-protein phosphatase 4 regulatory subunit 3B	97.397	4.96	32.919377	8.9517079	7	7	3	99.2	100.8	1.016	7.18E-02
Q6PIJ6	F-box only protein 38	133.858	6.33	3.5352125	0.8417508	1	1	1	99.2	100.8	1.016	
Q6UWE0	E3 ubiquitin-protein ligase LRSAM1	83.541	5.94	32.70798	10.511757	7	7	7	99.2	100.8	1.016	1.25E-01
Q86XI2	Condensin-2 complex subunit G2	130.876	6.87	37.075215	6.7366579	8	10	8	99.2	100.8	1.016	9.79E-01
Q8IW35	Centrosomal protein of 97 kDa	96.921	5.02	8.3301255	1.1560694	1	1	1	99.2	100.8	1.016	
Q8N1F7	Nuclear pore complex protein Nup93	93.43	5.72	148.17455	25.763126	19	28	19	99.2	100.8	1.016	6.09E-01
Q8NE71	ATP-binding cassette sub-family F member 1	95.866	6.8	170.72917	23.076923	17	27	17	99.2	100.8	1.016	5.60E-02
Q969N2	GPI transamidase component PIG-T	65.658	8.38	18.466153	8.4775087	4	4	4	99.2	100.8	1.016	4.49E-01
Q969Y2	tRNA modification GTPase GTPBP3, mitochondrial	52.026	6.48	5.7302536	2.8455285	1	1	1	99.2	100.8	1.016	
Q96B36	Proline-rich AKT1 substrate 1	27.366	4.75	6.2675223	7.03125	2	2	2	99.2	100.8	1.016	
Q96CS2	HAUS augmin-like complex subunit 1	31.844	5.53	22.820409	16.906475	4	4	4	99.2	100.8	1.016	3.31E-01
Q96K17	Transcription factor BTF3 homolog 4	17.26	6.35	22.458984	41.772152	5	5	5	99.2	100.8	1.016	3.18E-02
Q96Q11	CCA tRNA nucleotidyltransferase 1, mitochondrial	50.096	8.1	36.669821	11.059908	5	8	5	99.2	100.8	1.016	3.04E-01
Q99447	Ethanolamine-phosphate cytidylyltransferase	43.808	6.92	41.254698	18.508997	7	10	7	99.2	100.8	1.016	7.62E-01
Q99497	Protein deglycase DJ-1	19.878	6.79	145.30398	58.730159	12	56	12	99.2	100.8	1.016	2.52E-01
Q9BRG1	Vacuolar protein-sorting-associated protein 25	20.735	6.34	28.484742	27.840909	4	5	4	99.2	100.8	1.016	5.07E-01

Q9BSJ8	Extended synaptotagmin-1	122.78	5.83	196.00922	22.554348	19	37	18	99.2	100.8	1.016	8.13E-04
Q9BZE4	Nucleolar GTP-binding protein 1	73.918	9.5	50.576527	13.249211	8	11	8	99.2	100.8	1.016	1.90E-01
Q9BZG1	Ras-related protein Rab-34	29.026	7.88	41.674694	22.393822	5	7	5	99.2	100.8	1.016	3.13E-01
Q9C0C9	E2/E3 hybrid ubiquitin-protein ligase UBE2O	141.205	5.12	37.802526	5.1083591	5	5	5	99.2	100.8	1.016	8.64E-02
Q9H2U1	ATP-dependent RNA helicase DHX36	114.688	7.68	77.25313	11.210317	10	16	9	99.2	100.8	1.016	1.78E-02
Q9H3U1	Protein unc-45 homolog A	103.011	6.07	194.2008	34.427966	28	38	28	99.2	100.8	1.016	7.43E-02
Q9HD42	Charged multivesicular body protein 1a	21.689	8.06	9.3991221	15.306122	4	5	4	99.2	100.8	1.016	
Q9NPF5	DNA methyltransferase 1-associated protein 1	52.96	9.5	25.941348	13.276231	5	5	5	99.2	100.8	1.016	2.22E-01
Q9NSD9	Phenylalanine--tRNA ligase beta subunit	66.074	6.84	115.57563	28.52292	18	30	17	99.2	100.8	1.016	2.01E-03
Q9NUQ8	ATP-binding cassette sub-family F member 3	79.695	6.34	77.014005	15.091678	8	12	8	99.2	100.8	1.016	8.22E-01
Q9NVU7	Protein SDA1 homolog	79.82	9.25	10.074753	2.9112082	2	3	2	99.2	100.8	1.016	2.93E-01
Q9NWY4	UPF0609 protein C4orf27	39.411	6.8	9.2358239	4.0462428	1	1	1	99.2	100.8	1.016	
Q9NX58	Cell growth-regulating nucleolar protein	43.588	9.54	38.170259	10.290237	3	5	3	99.2	100.8	1.016	2.08E-01
Q9NZZ3	Charged multivesicular body protein 5	24.555	4.83	45.614569	31.506849	6	8	6	99.2	100.8	1.016	5.57E-02
Q9UHD2	Serine/threonine-protein kinase TBK1	83.589	6.79	23.032538	6.4471879	4	4	4	99.2	100.8	1.016	
Q9UIF9	Bromodomain adjacent to zinc finger domain protein 2A	211.065	6.64	16.065153	1.9947507	4	4	4	99.2	100.8	1.016	5.82E-01
Q9UMX5	Neudesin	18.845	5.69	34.24207	26.162791	4	5	4	99.2	100.8	1.016	6.88E-01
Q9UNP9	Peptidyl-prolyl cis-trans isomerase E	33.41	5.6	49.762528	25.581395	7	10	6	99.2	100.8	1.016	6.27E-01
Q9Y3A6	Transmembrane emp24 domain-containing protein 5	25.988	4.84	14.43126	5.2401747	1	4	1	99.2	100.8	1.016	
Q9Y587	AP-4 complex subunit sigma-1	16.994	5.19	6.7338625	6.9444444	1	2	1	99.2	100.8	1.016	
Q9Y5B6	PAX3- and PAX7-binding protein 1	104.739	5.68	11.481683	3.3805889	3	3	3	99.2	100.8	1.016	
A8MWD9	Putative small nuclear ribonucleoprotein G-like protein 15	8.538	8.84	28.162113	34.210526	3	12	3	99.1	100.9	1.018	3.51E-01
O14657	Torsin-1B	37.955	8.54	12.56721	7.4404762	2	2	2	99.1	100.9	1.018	
O15164	Transcription intermediary factor 1-alpha	116.757	7.11	32.116874	5.5238095	4	4	4	99.1	100.9	1.018	9.30E-01
O15357	Phosphatidylinositol 3,4,5-trisphosphate 5-phosphatase 2	138.513	6.54	119.53789	17.329094	18	22	18	99.1	100.9	1.018	1.10E-01
O60547	GDP-mannose 4,6 dehydratase	41.923	7.31	22.58292	16.397849	6	6	6	99.1	100.9	1.018	9.56E-01
O75152	Zinc finger CCCH domain-containing protein 11A	89.076	8.37	54.137921	14.567901	10	12	10	99.1	100.9	1.018	1.97E-02
O75616	GTPase Era, mitochondrial	48.319	8.84	27.335	9.610984	3	4	3	99.1	100.9	1.018	
O75818	Ribonuclease P protein subunit p40	41.807	6.67	36.045152	14.049587	4	6	4	99.1	100.9	1.018	1.86E-01
O95235	Kinesin-like protein KIF20A	100.215	6.92	40.882685	8.5393258	6	6	6	99.1	100.9	1.018	3.46E-01
O95249	Golgi SNAP receptor complex member 1	28.595	9.42	9.0338991	8.4	2	2	2	99.1	100.9	1.018	6.84E-01
O95625	Zinc finger and BTB domain-containing protein 11	119.308	8.66	10.894871	2.2792023	3	3	3	99.1	100.9	1.018	
O95747	Serine/threonine-protein kinase OSR1	57.986	6.43	97.12684	24.288425	10	14	9	99.1	100.9	1.018	5.14E-01
P05783	Keratin, type I cytoskeletal 18	48.029	5.45	384.53311	56.744186	28	117	28	99.1	100.9	1.018	2.19E-03
P07814	Bifunctional glutamate/proline--tRNA ligase	170.483	7.33	574.07803	36.640212	49	112	49	99.1	100.9	1.018	8.53E-02
P07992	DNA excision repair protein ERCC-1	32.542	6.25	5.5668705	3.030303	1	1	1	99.1	100.9	1.018	
P23381	Tryptophan--tRNA ligase, cytoplasmic	53.132	6.23	168.97108	33.757962	14	29	14	99.1	100.9	1.018	7.64E-02
P24928	DNA-directed RNA polymerase II subunit RPB1	217.039	7.37	104.08707	11.42132	19	21	19	99.1	100.9	1.018	6.08E-01
P31948	Stress-induced-phosphoprotein 1	62.599	6.8	275.41411	52.117864	32	78	32	99.1	100.9	1.018	1.01E-01
P35610	Sterol O-acyltransferase 1	64.692	8.94	47.007789	12.909091	5	10	5	99.1	100.9	1.018	1.63E-01
P51610	Host cell factor 1	208.602	7.46	136.56231	11.351351	20	25	20	99.1	100.9	1.018	7.88E-02
P52597	Heterogeneous nuclear ribonucleoprotein F	45.643	5.58	163.7409	35.180723	11	47	9	99.1	100.9	1.018	9.40E-01
P52948	Nuclear pore complex protein Nup98-Nup96	197.457	6.4	165.27287	15.520088	25	34	25	99.1	100.9	1.018	1.77E-01
P55145	Mesencephalic astrocyte-derived neurotrophic factor	20.687	8.69	67.365754	26.373626	8	12	8	99.1	100.9	1.018	6.48E-02
P56182	Ribosomal RNA processing protein 1 homolog A	52.807	9.33	32.982016	17.136659	7	7	7	99.1	100.9	1.018	4.13E-01
P61764	Syntaxin-binding protein 1	67.526	6.96	46.734543	16.161616	9	11	9	99.1	100.9	1.018	5.66E-01
P62333	26S protease regulatory subunit 10B	44.145	7.49	214.43981	48.071979	16	47	16	99.1	100.9	1.018	3.61E-01

Q04726	Transducin-like enhancer protein 3	83.364	7.2	22.417867	7.9015544	6	6	3	99.1	100.9	1.018	
Q07960	Rho GTPase-activating protein 1	50.404	6.29	93.899181	31.43508	13	21	13	99.1	100.9	1.018	9.01E-02
Q14155	Rho guanine nucleotide exchange factor 7	89.955	7.09	46.082515	8.9663761	6	6	6	99.1	100.9	1.018	1.13E-01
Q14192	Four and a half LIM domains protein 2	32.171	7.55	152.61557	59.139785	13	30	13	99.1	100.9	1.018	2.14E-02
Q14320	Protein FAM50A	40.216	6.83	81.045866	25.073746	9	16	9	99.1	100.9	1.018	7.25E-02
Q15366	Poly(rC)-binding protein 2	38.556	6.79	129.41593	34.520548	11	37	4	99.1	100.9	1.018	3.55E-02
Q15654	Thyroid receptor-interacting protein 6	50.255	7.37	95.548401	26.680672	8	19	8	99.1	100.9	1.018	1.26E-01
Q53EL6	Programmed cell death protein 4	51.703	5.21	96.232585	26.865672	11	20	11	99.1	100.9	1.018	2.24E-01
Q5BKY9	Protein FAM133B	28.368	10.02	3.1590788	4.048583	1	1	1	99.1	100.9	1.018	
Q6IN85	Serine/threonine-protein phosphatase 4 regulatory subunit 3A	95.308	4.94	60.252647	13.205282	10	12	6	99.1	100.9	1.018	1.44E-01
Q6P1J9	Parafibromin	60.539	9.61	80.308787	23.352166	13	20	13	99.1	100.9	1.018	3.16E-01
Q6PK04	Coiled-coil domain-containing protein 137	33.211	10.93	9.8278396	9.6885813	3	3	3	99.1	100.9	1.018	1.60E-01
Q6Y7W6	PERQ amino acid-rich with GYF domain-containing protein 2	149.978	5.54	81.686144	11.624326	14	26	12	99.1	100.9	1.018	5.35E-02
Q7Z2T5	TRMT1-like protein	81.695	7.88	16.98659	4.3656207	3	3	3	99.1	100.9	1.018	
Q7Z4S6	Kinesin-like protein KIF21A	187.063	6.42	52.527466	5.734767	9	11	9	99.1	100.9	1.018	3.45E-01
Q86WA8	Lon protease homolog 2, peroxisomal	94.557	7.3	9.5084316	3.9906103	3	3	3	99.1	100.9	1.018	
Q8NBF2	NHL repeat-containing protein 2	79.393	5.55	33.942306	10.46832	5	5	4	99.1	100.9	1.018	4.30E-01
Q8WVM8	Sec1 family domain-containing protein 1	72.334	6.27	101.71395	21.65109	11	16	11	99.1	100.9	1.018	5.14E-01
Q8WYA6	Beta-catenin-like protein 1	65.132	5.05	32.492793	10.834813	7	11	7	99.1	100.9	1.018	4.09E-01
Q92541	RNA polymerase-associated protein RTF1 homolog	80.265	8.15	57.306522	12.676056	9	12	9	99.1	100.9	1.018	4.06E-01
Q96A72	Protein mago nashi homolog 2	17.265	6.39	54.414876	47.972973	9	14	9	99.1	100.9	1.018	9.29E-02
Q96GX9	Methylthioribulose-1-phosphate dehydratase	27.107	7.12	24.004766	25.619835	4	4	4	99.1	100.9	1.018	2.97E-01
Q96PE2	Rho guanine nucleotide exchange factor 17	221.535	6.29	19.742265	1.6965584	3	3	3	99.1	100.9	1.018	2.30E-01
Q9BQG0	Myb-binding protein 1A	148.762	9.28	179.7896	18.524096	22	39	22	99.1	100.9	1.018	7.42E-01
Q9BZI7	Regulator of nonsense transcripts 3B	57.727	9.48	58.73984	16.977226	7	11	7	99.1	100.9	1.018	8.78E-02
Q9H6R4	Nucleolar protein 6	127.513	7.64	64.974661	10.209424	10	12	10	99.1	100.9	1.018	8.29E-02
Q9HAS0	Protein Njmu-R1	44.593	5.03	14.026868	4.7979798	2	3	2	99.1	100.9	1.018	
Q9HB07	UPF0160 protein MYG1, mitochondrial	42.422	6.67	44.610893	15.425532	6	11	6	99.1	100.9	1.018	3.43E-01
Q9HCK8	Chromodomain-helicase-DNA-binding protein 8	290.338	6.47	31.909335	2.363425	6	8	5	99.1	100.9	1.018	
Q9NQG5	Regulation of nuclear pre-mRNA domain-containing protein 1B	36.877	5.97	48.979692	26.07362	6	9	5	99.1	100.9	1.018	1.33E-01
Q9NV70	Exocyst complex component 1	101.917	6.61	5.0780012	2.2371365	2	2	2	99.1	100.9	1.018	
Q9NW64	Pre-mRNA-splicing factor RBM22	46.865	8.54	36.092745	15.238095	8	9	8	99.1	100.9	1.018	5.69E-01
Q9NZM1	Myoferlin	234.561	6.18	563.10444	35.75934	64	108	64	99.1	100.9	1.018	1.16E-02
Q9UBQ5	Eukaryotic translation initiation factor 3 subunit K	25.043	4.93	53.529651	26.605505	4	9	4	99.1	100.9	1.018	2.49E-01
Q9Y224	UPF0568 protein C14orf166	28.051	6.65	62.951872	32.786885	7	17	7	99.1	100.9	1.018	9.35E-02
Q9Y281	Cofilin-2	18.725	7.88	64.268464	48.192771	8	23	4	99.1	100.9	1.018	3.63E-01
Q9Y2H5	Pleckstrin homology domain-containing family A member 6	117.056	9.1	8.4517886	1.9083969	2	2	2	99.1	100.9	1.018	2.93E-01
A6NDG6	Phosphoglycolate phosphatase	33.985	6.14	56.152772	26.791277	7	14	7	99	101	1.02	2.23E-01
O00116	Alkyldihydroxyacetonephosphate synthase, peroxisomal	72.866	7.34	101.20612	23.860182	12	16	12	99	101	1.02	2.98E-01
O00233	26S proteasome non-ATPase regulatory subunit 9	24.667	6.95	79.973131	43.946188	8	15	8	99	101	1.02	8.40E-03
O00410	Importin-5	123.55	4.94	355.55856	32.5433	33	63	31	99	101	1.02	1.50E-02
O15156	Zinc finger and BTB domain-containing protein 7B	57.99	5.86	5.1096349	2.2263451	1	1	1	99	101	1.02	
O43390	Heterogeneous nuclear ribonucleoprotein R	70.899	8.13	238.7871	35.545024	22	66	14	99	101	1.02	6.36E-02
O43617	Trafficking protein particle complex subunit 3	20.261	4.96	30.350278	20	4	6	4	99	101	1.02	5.88E-02
O75151	Lysine-specific demethylase PHF2	120.7	9.17	5.5922693	1.2773723	1	1	1	99	101	1.02	
O75691	Small subunit processome component 20 homolog	318.182	7.39	55.237347	4.7396768	11	11	11	99	101	1.02	7.13E-01
O94763	Unconventional prefoldin RPB5 interactor 1	59.796	5.05	14.556281	5.6074766	3	3	3	99	101	1.02	6.67E-02

O94842	TOX high mobility group box family member 4	66.153	5.06	17.163683	5.4750403	3	5	3	99	101	1.02	8.54E-01
O95239	Chromosome-associated kinesin KIF4A	139.794	6.27	114.42598	16.720779	17	20	16	99	101	1.02	2.01E-01
O95373	Importin-7	119.44	4.82	209.51235	23.795761	22	35	22	99	101	1.02	7.65E-01
O95793	Double-stranded RNA-binding protein Staufen homolog 1	63.143	9.44	13.684906	5.1993068	3	3	1	99	101	1.02	
O95817	BAG family molecular chaperone regulator 3	61.557	6.95	83.962451	26.782609	12	18	12	99	101	1.02	3.10E-01
P07910	Heterogeneous nuclear ribonucleoproteins C1/C2	33.65	5.08	184.03363	43.464052	17	64	17	99	101	1.02	2.10E-01
P09884	DNA polymerase alpha catalytic subunit	165.807	5.85	44.832507	6.1559508	10	11	9	99	101	1.02	3.38E-01
P12236	ADP/ATP translocase 3	32.845	9.74	108.25819	40.604027	14	32	2	99	101	1.02	5.13E-02
P15311	Ezrin	69.37	6.27	264.28102	42.491468	32	93	19	99	101	1.02	8.87E-02
P17931	Galectin-3	26.136	8.56	40.509331	20	5	16	5	99	101	1.02	2.31E-01
P17980	26S protease regulatory subunit 6A	49.172	5.24	298.22493	62.642369	25	71	25	99	101	1.02	2.58E-01
P19404	NADH dehydrogenase [ubiquinone] flavoprotein 2, mitochondrial	27.374	8.06	44.230403	20.481928	5	8	5	99	101	1.02	9.54E-03
P19623	Spermidine synthase	33.803	5.49	72.742522	31.788079	8	17	8	99	101	1.02	6.63E-01
P22626	Heterogeneous nuclear ribonucleoproteins A2/B1	37.407	8.95	303.38156	63.172805	24	136	19	99	101	1.02	6.27E-03
P24666	Low molecular weight phosphotyrosine protein phosphatase	18.031	6.74	60.68383	32.278481	5	14	5	99	101	1.02	5.56E-02
P26368	Splicing factor U2AF 65 kDa subunit	53.467	9.09	71.070182	20.210526	8	19	8	99	101	1.02	9.63E-01
P27338	Amine oxidase [flavin-containing] B	58.725	7.5	86.610405	29.230769	12	16	12	99	101	1.02	1.38E-02
P30041	Peroxiredoxin-6	25.019	6.38	169.95939	57.589286	16	46	16	99	101	1.02	8.40E-01
P30042	ES1 protein homolog, mitochondrial	28.153	8.27	35.024955	24.626866	5	6	5	99	101	1.02	
P35606	Coatomer subunit beta'	102.422	5.27	179.3575	26.600442	22	38	22	99	101	1.02	4.08E-02
P35637	RNA-binding protein FUS	53.394	9.36	129.53134	16.730038	10	36	8	99	101	1.02	5.53E-01
P43686	26S protease regulatory subunit 6B	47.337	5.21	108.94294	34.449761	15	24	14	99	101	1.02	4.16E-01
P49757	Protein numb homolog	70.759	8.51	21.827713	6.7588326	4	5	3	99	101	1.02	
P50897	Palmitoyl-protein thioesterase 1	34.171	6.52	21.283052	7.8431373	2	3	2	99	101	1.02	7.69E-02
P53618	Coatomer subunit beta	107.074	6.05	262.51332	26.652676	24	52	24	99	101	1.02	7.18E-01
P54577	Tyrosine--tRNA ligase, cytoplasmic	59.106	7.05	193.38093	44.69697	25	49	25	99	101	1.02	3.21E-03
P61006	Ras-related protein Rab-8A	23.653	9.07	57.428831	38.164251	9	21	5	99	101	1.02	9.54E-02
P62166	Neuronal calcium sensor 1	21.865	4.83	14.793905	12.105263	2	2	2	99	101	1.02	
P68104	Elongation factor 1-alpha 1	50.109	9.01	265.78073	42.857143	21	113	14	99	101	1.02	7.15E-03
Q04917	14-3-3 protein eta	28.201	4.84	88.297958	40.243902	12	42	7	99	101	1.02	7.80E-01
Q13428	Treacle protein	152.015	9.04	197.60414	23.723118	33	42	33	99	101	1.02	3.85E-04
Q13601	KRR1 small subunit processome component homolog	43.638	9.77	37.083466	10.498688	4	7	4	99	101	1.02	4.39E-01
Q14258	E3 ubiquitin/ISG15 ligase TRIM25	70.928	8.09	117.47049	29.84127	15	20	15	99	101	1.02	2.06E-01
Q14498	RNA-binding protein 39	59.343	10.1	109.32867	26.603774	13	23	13	99	101	1.02	4.04E-01
Q14684	Ribosomal RNA processing protein 1 homolog B	84.375	9.76	82.791791	19.656992	12	16	12	99	101	1.02	4.38E-01
Q15041	ADP-ribosylation factor-like protein 6-interacting protein 1	23.347	9.32	11.680073	4.9261084	1	3	1	99	101	1.02	
Q15843	NEDD8	9.066	8.43	28.349595	35.802469	4	10	4	99	101	1.02	1.85E-01
Q16706	Alpha-mannosidase 2	131.057	7.58	31.878554	6.2062937	7	9	7	99	101	1.02	8.88E-02
Q16773	Kynurenine--oxoglutarate transaminase 1	47.844	6.47	13.257415	7.3459716	3	3	2	99	101	1.02	
Q6KB66	Keratin, type II cytoskeletal 80	50.494	5.67	91.096302	26.769912	12	18	11	99	101	1.02	4.45E-01
Q7Z2Z2	Elongation factor Tu GTP-binding domain-containing protein 1	125.35	5.91	47.484447	6.9642857	6	7	6	99	101	1.02	7.11E-01
Q7Z7H8	39S ribosomal protein L10, mitochondrial	29.264	9.58	5.2753013	5.3639847	2	2	2	99	101	1.02	9.80E-01
Q8N5K1	CDGSH iron-sulfur domain-containing protein 2	15.268	9.61	45.104889	34.814815	4	8	4	99	101	1.02	4.12E-01
Q8N8S7	Protein enabled homolog	66.47	6.93	19.730609	7.106599	4	4	4	99	101	1.02	3.56E-01
Q8N9N7	Leucine-rich repeat-containing protein 57	26.737	8.43	28.885818	19.665272	4	6	4	99	101	1.02	1.07E-01
Q8TCF1	AN1-type zinc finger protein 1	30.767	7.39	25.801503	11.567164	2	3	2	99	101	1.02	2.44E-01
Q92626	Peroxidasin homolog	165.17	7.17	20.223785	2.8397566	4	4	4	99	101	1.02	9.27E-01

Q96B49	Mitochondrial import receptor subunit TOM6 homolog	7.997	4.89	20.604532	18.918919	1	3	1	99	101	1.02	
Q96C86	m7GpppX diphosphatase	38.585	6.38	48.626557	26.409496	7	14	7	99	101	1.02	2.86E-02
Q99417	C-Myc-binding protein	11.959	5.91	41.185561	43.68932	4	10	4	99	101	1.02	5.63E-01
Q99590	Protein SCAF11	164.551	8.41	59.678453	6.7669173	8	10	8	99	101	1.02	3.26E-01
Q9BYW2	Histone-lysine N-methyltransferase SETD2	287.418	6.14	13.936668	1.5600624	3	3	3	99	101	1.02	5.13E-02
Q9GZR7	ATP-dependent RNA helicase DDX24	96.271	9.06	64.514784	9.6623981	9	16	9	99	101	1.02	1.65E-01
Q9GZT3	SRA stem-loop-interacting RNA-binding protein, mitochondrial	12.341	10.24	48.360186	64.220183	7	12	7	99	101	1.02	1.01E-01
Q9H2H9	Sodium-coupled neutral amino acid transporter 1	54.012	7.02	25.337622	8.8295688	5	5	5	99	101	1.02	8.22E-03
Q9H2J4	Phosducin-like protein 3	27.597	4.84	19.570109	12.552301	3	6	3	99	101	1.02	2.73E-01
Q9H2P9	Diphthine methyl ester synthase	31.631	5.31	40.7661	21.754386	5	7	5	99	101	1.02	5.75E-01
Q9H9E3	Conserved oligomeric Golgi complex subunit 4	89.026	5.19	29.581417	8.5350318	6	6	6	99	101	1.02	6.08E-01
Q9NQX3	Gephyrin	79.698	5.43	38.579503	7.6086957	4	6	4	99	101	1.02	4.68E-01
Q9NVM6	DnaJ homolog subfamily C member 17	34.666	8.53	21.836561	16.776316	4	4	4	99	101	1.02	
Q9NWB1	RNA binding protein fox-1 homolog 1	42.758	6.86	22.081494	6.2972292	2	4	2	99	101	1.02	2.93E-01
Q9NWX5	Ankyrin repeat and SOCS box protein 6	47.106	6.01	3.8807441	2.3752969	1	1	1	99	101	1.02	
Q9NWX6	Probable tRNA(His) guanylyltransferase	34.808	8	18.064778	12.080537	3	3	3	99	101	1.02	1.43E-01
Q9NZL9	Methionine adenosyltransferase 2 subunit beta	37.528	7.36	59.332817	35.329341	11	12	11	99	101	1.02	1.34E-03
Q9UBC2	Epidermal growth factor receptor substrate 15-like 1	94.197	5.11	87.655731	14.930556	11	15	11	99	101	1.02	1.86E-02
Q9UBF8	Phosphatidylinositol 4-kinase beta	91.321	6.25	5.8830604	1.4705882	1	1	1	99	101	1.02	
Q9UKN8	General transcription factor 3C polypeptide 4	91.923	6.65	54.09948	13.017032	10	11	10	99	101	1.02	1.06E-01
Q9UPU5	Ubiquitin carboxyl-terminal hydrolase 24	294.178	6.14	81.232861	7.3664122	19	20	18	99	101	1.02	1.33E-02
Q9Y2R4	Probable ATP-dependent RNA helicase DDX52	67.456	9.67	17.346736	8.0133556	5	5	4	99	101	1.02	
Q9Y399	28S ribosomal protein S2, mitochondrial	33.228	9.26	21.622806	19.594595	7	8	7	99	101	1.02	1.34E-01
Q9Y5B9	FACT complex subunit SPT16	119.838	5.66	152.93859	17.765043	20	35	19	99	101	1.02	2.19E-02
O00425	Insulin-like growth factor 2 mRNA-binding protein 3	63.666	8.87	148.75955	32.98791	15	27	12	98.9	101.1	1.022	6.21E-01
O14617	AP-3 complex subunit delta-1	130.076	8.48	69.948278	11.361665	10	12	10	98.9	101.1	1.022	5.39E-01
O14641	Segment polarity protein dishevelled homolog DVL-2	78.899	6.02	13.283554	3.8043478	3	3	2	98.9	101.1	1.022	9.38E-02
O14964	Hepatocyte growth factor-regulated tyrosine kinase substrate	86.138	6.16	39.492525	10.810811	8	9	8	98.9	101.1	1.022	9.05E-01
O15355	Protein phosphatase 1G	59.235	4.36	133.18865	27.655678	13	30	13	98.9	101.1	1.022	3.54E-02
O43242	26S proteasome non-ATPase regulatory subunit 3	60.939	8.44	129.06366	27.715356	14	28	14	98.9	101.1	1.022	1.00E-02
O43396	Thioredoxin-like protein 1	32.231	4.96	87.711017	41.522491	7	16	7	98.9	101.1	1.022	1.02E-02
O60826	Coiled-coil domain-containing protein 22	70.712	6.74	39.5526	11.004785	5	5	5	98.9	101.1	1.022	6.17E-01
O75179	Ankyrin repeat domain-containing protein 17	274.088	6.52	117.04211	6.8766808	18	22	9	98.9	101.1	1.022	8.02E-01
O75390	Citrate synthase, mitochondrial	51.68	8.32	87.571646	22.746781	11	31	11	98.9	101.1	1.022	4.35E-01
O94992	Protein HEXIM1	40.598	4.89	41.837471	20.612813	4	6	4	98.9	101.1	1.022	1.79E-01
O95372	Acyl-protein thioesterase 2	24.721	7.23	37.235072	18.181818	4	9	4	98.9	101.1	1.022	6.56E-02
P11182	erase component of branched-chain alpha-keto acid dehydrogenase co	53.453	8.51	17.234365	7.0539419	3	4	3	98.9	101.1	1.022	
P11216	Glycogen phosphorylase, brain form	96.635	6.86	136.26317	23.250297	19	29	17	98.9	101.1	1.022	2.82E-01
P11586	C-1-tetrahydrofolate synthase, cytoplasmic	101.495	7.3	441.24203	48.877005	41	86	41	98.9	101.1	1.022	3.25E-04
P16435	NADPH--cytochrome P450 reductase	76.641	5.58	46.408366	12.407681	8	10	8	98.9	101.1	1.022	2.20E-01
P18859	ATP synthase-coupling factor 6, mitochondrial	12.58	9.52	55.842877	47.222222	5	9	5	98.9	101.1	1.022	9.44E-01
P18887	DNA repair protein XRCC1	69.434	6.39	11.431754	5.5292259	4	4	4	98.9	101.1	1.022	4.31E-01
P20042	Eukaryotic translation initiation factor 2 subunit 2	38.364	5.8	145.14041	50.15015	17	27	17	98.9	101.1	1.022	2.14E-01
P20290	Transcription factor BTF3	22.154	9.38	29.265487	15.048544	3	10	3	98.9	101.1	1.022	1.48E-01
P22570	NADPH:adrenodoxin oxidoreductase, mitochondrial	53.803	8.44	15.0737	9.7759674	4	4	4	98.9	101.1	1.022	9.33E-01
P50749	Ras association domain-containing protein 2	37.767	8.84	2.5738142	2.4539877	1	1	1	98.9	101.1	1.022	
P50851	Lipopolysaccharide-responsive and beige-like anchor protein	318.906	5.6	31.60923	2.375131	6	6	6	98.9	101.1	1.022	

P57088	Transmembrane protein 33	27.96	9.7	11.797798	14.17004	4	4	4	98.9	101.1	1.022	5.44E-01
P78406	mRNA export factor	40.942	7.83	108.72512	33.695652	10	20	10	98.9	101.1	1.022	8.64E-01
Q00765	Receptor expression-enhancing protein 5	21.479	8.1	27.445253	11.111111	3	5	3	98.9	101.1	1.022	9.63E-02
Q08752	Peptidyl-prolyl cis-trans isomerase D	40.738	7.21	89.924291	35.675676	14	22	13	98.9	101.1	1.022	6.41E-02
Q13425	Beta-2-syntrophin	57.913	8.82	30.055111	10.555556	5	6	5	98.9	101.1	1.022	
Q14103	Heterogeneous nuclear ribonucleoprotein D0	38.41	7.81	153.02094	32.394366	12	42	10	98.9	101.1	1.022	3.96E-02
Q14139	Ubiquitin conjugation factor E4 A	122.482	5.24	51.571794	8.9118199	9	11	9	98.9	101.1	1.022	2.58E-02
Q14444	Caprin-1	78.318	5.25	116.56727	16.220028	12	27	12	98.9	101.1	1.022	2.03E-03
Q14974	Importin subunit beta-1	97.108	4.78	284.27161	35.958904	25	62	25	98.9	101.1	1.022	1.80E-03
Q15370	Transcription elongation factor B polypeptide 2	13.125	4.88	48.603333	79.661017	7	16	7	98.9	101.1	1.022	1.00E+00
Q15436	Protein transport protein Sec23A	86.105	7.08	165.11067	30.065359	20	38	18	98.9	101.1	1.022	7.47E-01
Q16850	Lanosterol 14-alpha demethylase	56.769	8.53	28.510514	11.332008	5	5	5	98.9	101.1	1.022	1.49E-02
Q5H943	Kita-kyushu lung cancer antigen 1	12.776	10.2	5.4213608	9.7345133	1	1	1	98.9	101.1	1.022	
Q5THJ4	Vacuolar protein sorting-associated protein 13D	491.606	6.58	10.173449	0.6608933	3	3	3	98.9	101.1	1.022	9.67E-01
Q5VWQ0	Round spermatid basic protein 1	90.015	8.6	10.993861	2.9925187	2	2	2	98.9	101.1	1.022	
Q5W0V3	Protein FAM160B1	86.503	5.29	18.793456	5.0980392	4	4	4	98.9	101.1	1.022	
Q66PJ3	ADP-ribosylation factor-like protein 6-interacting protein 4	44.888	10.93	29.249007	5.7007126	3	5	3	98.9	101.1	1.022	
Q6P1A2	Lysophospholipid acyltransferase 5	55.998	8.69	4.1658337	2.0533881	1	1	1	98.9	101.1	1.022	
Q7L2E3	Putative ATP-dependent RNA helicase DHX30	133.855	8.78	84.40416	11.055276	12	17	12	98.9	101.1	1.022	3.04E-01
Q7Z739	YTH domain-containing family protein 3	63.822	9.04	59.590542	15.897436	8	10	6	98.9	101.1	1.022	1.54E-03
Q86UK7	Zinc finger protein 598	98.575	8.4	73.378825	16.150442	11	15	11	98.9	101.1	1.022	9.06E-02
Q86YT6	E3 ubiquitin-protein ligase MIB1	110.066	6.92	28.754625	4.8707753	4	5	4	98.9	101.1	1.022	5.19E-01
Q8IU85	Calcium/calmodulin-dependent protein kinase type 1D	42.887	7.21	17.797679	7.5324675	4	4	4	98.9	101.1	1.022	
Q8IUR7	Armadillo repeat-containing protein 8	75.46	6.73	16.234388	6.9836553	5	5	5	98.9	101.1	1.022	2.93E-01
Q8IVT2	Mitotic interactor and substrate of PLK1	75.311	6.83	73.263828	21.060383	10	14	10	98.9	101.1	1.022	2.10E-02
Q8NC60	Nitric oxide-associated protein 1	78.409	8.66	11.438647	3.4383954	2	2	2	98.9	101.1	1.022	
Q8NHG7	Small VCP/p97-interacting protein	8.437	8.91	7.8626459	14.285714	1	1	1	98.9	101.1	1.022	
Q8WY22	BRI3-binding protein	27.818	9.44	12.961552	11.952191	3	3	3	98.9	101.1	1.022	3.41E-01
Q93052	Lipoma-preferred partner	65.704	7.37	48.791559	15.686275	7	9	7	98.9	101.1	1.022	1.64E-01
Q96HC4	PDZ and LIM domain protein 5	63.904	8.21	114.23588	27.181208	14	19	14	98.9	101.1	1.022	5.29E-01
Q99439	Calponin-2	33.675	7.33	82.227673	28.15534	8	17	6	98.9	101.1	1.022	1.21E-01
Q99569	Plakophilin-4	131.787	8.94	6.1710011	1.4261745	2	2	2	98.9	101.1	1.022	
Q99805	Transmembrane 9 superfamily member 2	75.725	7.44	50.562078	8.1447964	5	8	5	98.9	101.1	1.022	9.08E-01
Q9BSJ2	Gamma-tubulin complex component 2	102.469	6.84	32.53295	8.6474501	8	10	8	98.9	101.1	1.022	1.09E-01
Q9BX95	Sphingosine-1-phosphate phosphatase 1	49.076	8.82	21.49882	6.122449	3	5	3	98.9	101.1	1.022	3.02E-01
Q9BYX2	TBC1 domain family member 2A	105.348	6.58	39.581738	7.4353448	6	8	6	98.9	101.1	1.022	1.05E-01
Q9H1K1	Iron-sulfur cluster assembly enzyme ISCU, mitochondrial	17.988	9.48	16.581701	20.359281	3	3	3	98.9	101.1	1.022	8.23E-01
Q9NR46	Endophilin-B2	43.947	5.99	36.476737	17.21519	8	11	8	98.9	101.1	1.022	2.13E-01
Q9NVI7	ATPase family AAA domain-containing protein 3A	71.325	8.98	124.84641	26.971609	14	24	5	98.9	101.1	1.022	9.44E-02
Q9NVX2	Notchless protein homolog 1	53.287	7.34	24.479521	8.6597938	3	4	3	98.9	101.1	1.022	
Q9UIA9	Exportin-7	123.828	6.32	72.282258	12.143514	12	14	12	98.9	101.1	1.022	1.81E-01
Q9Y2Q3	Glutathione S-transferase kappa 1	25.48	8.41	77.797953	31.858407	6	11	6	98.9	101.1	1.022	6.92E-01
Q9Y580	RNA-binding protein 7	30.485	9.57	11.695442	3.7593985	1	2	1	98.9	101.1	1.022	
Q9Y678	Coatomer subunit gamma-1	97.655	5.47	240.60066	33.066362	25	56	22	98.9	101.1	1.022	1.31E-02
O15160	DNA-directed RNA polymerases I and III subunit RPAC1	39.225	5.5	62.447607	24.855491	7	12	7	98.8	101.2	1.024	8.77E-02
O43143	Pre-mRNA-splicing factor ATP-dependent RNA helicase DHX15	90.875	7.46	145.88538	28.930818	21	34	21	98.8	101.2	1.024	8.52E-03
O43399	Tumor protein D54	22.224	5.36	109.41162	48.058252	10	22	10	98.8	101.2	1.024	1.43E-03

O60287	Nucleolar pre-ribosomal-associated protein 1	254.227	6.47	54.634055	3.9189784	7	8	7	98.8	101.2	1.024	1.68E-01
O60610	Protein diaphanous homolog 1	141.258	5.41	178.71084	19.575472	23	38	23	98.8	101.2	1.024	6.80E-01
O75030	Microphthalmia-associated transcription factor	58.758	6.33	28.870914	9.8859316	6	8	6	98.8	101.2	1.024	5.41E-01
O75533	Splicing factor 3B subunit 1	145.738	7.09	218.11681	24.156442	27	43	27	98.8	101.2	1.024	9.77E-01
O75934	Pre-mRNA-splicing factor SPF27	26.115	5.66	95.879976	45.777778	8	14	8	98.8	101.2	1.024	5.66E-01
O94776	Metastasis-associated protein MTA2	74.976	9.66	55.609298	14.371257	9	14	7	98.8	101.2	1.024	1.32E-01
O95081	Arf-GAP domain and FG repeat-containing protein 2	48.932	9.11	20.540926	8.5239085	3	5	3	98.8	101.2	1.024	1.65E-01
O95218	Zinc finger Ran-binding domain-containing protein 2	37.382	10.01	37.433582	17.575758	7	9	7	98.8	101.2	1.024	9.52E-01
O95376	E3 ubiquitin-protein ligase ARIH2	57.781	5.63	11.742792	3.8539554	2	2	2	98.8	101.2	1.024	
P05187	Alkaline phosphatase, placental type	57.917	6.29	195.57521	40.747664	19	47	16	98.8	101.2	1.024	1.78E-02
P07900	Heat shock protein HSP 90-alpha	84.607	5.02	496.53424	45.491803	38	235	20	98.8	101.2	1.024	3.16E-02
P07947	Tyrosine-protein kinase Yes	60.763	6.74	29.422392	9.9447514	6	6	2	98.8	101.2	1.024	
P08651	Nuclear factor 1 C-type	55.64	8.38	19.437083	5.1181102	2	4	2	98.8	101.2	1.024	5.13E-02
P11171	Protein 4.1	96.957	5.58	30.917368	7.7546296	5	5	5	98.8	101.2	1.024	
P15923	Transcription factor E2-alpha	67.559	6.47	14.203959	3.6697248	2	2	2	98.8	101.2	1.024	6.84E-01
P23258	Tubulin gamma-1 chain	51.138	6.14	40.26913	17.2949	6	7	6	98.8	101.2	1.024	9.52E-02
P25440	Bromodomain-containing protein 2	88.006	9.09	14.738387	4.619226	3	3	2	98.8	101.2	1.024	
P30153	threonine-protein phosphatase 2A 65 kDa regulatory subunit A alpha i	65.267	5.11	155.74715	30.220713	16	37	14	98.8	101.2	1.024	1.51E-01
P31943	Heterogeneous nuclear ribonucleoprotein H	49.198	6.3	158.4724	37.416481	13	53	6	98.8	101.2	1.024	2.95E-02
P40855	Peroxisomal biogenesis factor 19	32.786	4.34	77.269226	32.77592	7	11	7	98.8	101.2	1.024	1.04E-01
P42226	Signal transducer and activator of transcription 6	94.075	6.23	20.903599	5.3128689	4	5	4	98.8	101.2	1.024	3.13E-01
P43487	Ran-specific GTPase-activating protein	23.296	5.29	59.294413	32.338308	6	20	6	98.8	101.2	1.024	1.55E-02
P48444	Coatomer subunit delta	57.174	6.21	143.28439	33.268102	18	30	18	98.8	101.2	1.024	1.19E-01
P49585	Choline-phosphate cytidylyltransferase A	41.705	7.25	10.132926	9.5367847	3	3	3	98.8	101.2	1.024	1.54E-01
P51659	Peroxisomal multifunctional enzyme type 2	79.636	8.84	145.63339	28.532609	17	30	17	98.8	101.2	1.024	4.68E-03
P53609	Geranylgeranyl transferase type-1 subunit beta	42.341	6.83	9.721163	4.2440318	2	3	2	98.8	101.2	1.024	1.82E-01
P53985	Monocarboxylate transporter 1	53.909	8.66	17.972032	2.6	2	4	2	98.8	101.2	1.024	
P61923	Coatomer subunit zeta-1	20.185	4.81	46.885481	27.118644	5	9	5	98.8	101.2	1.024	1.54E-01
P62750	60S ribosomal protein L23a	17.684	10.45	89.672533	51.923077	11	31	11	98.8	101.2	1.024	1.49E-02
P68371	Tubulin beta-4B chain	49.799	4.89	313.84805	55.280899	17	194	3	98.8	101.2	1.024	2.76E-01
P78371	T-complex protein 1 subunit beta	57.452	6.46	345.19408	51.028037	29	81	29	98.8	101.2	1.024	2.01E-02
P84098	60S ribosomal protein L19	23.451	11.47	30.648642	20.408163	5	6	5	98.8	101.2	1.024	1.38E-01
Q05682	Caldesmon	93.175	5.66	137.06331	19.167718	15	42	15	98.8	101.2	1.024	4.49E-03
Q12830	Nucleosome-remodeling factor subunit BPTF	338.054	6.54	22.264649	1.2147078	3	3	3	98.8	101.2	1.024	
Q12894	Interferon-related developmental regulator 2	54.779	7.94	12.001322	5.1383399	2	2	2	98.8	101.2	1.024	
Q13309	S-phase kinase-associated protein 2	47.731	7.11	9.9204356	7.3113208	2	2	2	98.8	101.2	1.024	1.36E-01
Q13643	Four and a half LIM domains protein 3	31.171	6.2	29.868803	19.285714	4	5	4	98.8	101.2	1.024	6.55E-01
Q14527	Helicase-like transcription factor	113.857	8.6	112.05674	14.667988	13	19	13	98.8	101.2	1.024	4.02E-01
Q14997	Proteasome activator complex subunit 4	211.199	6.9	41.131627	4.7748237	9	11	9	98.8	101.2	1.024	4.23E-04
Q15067	Peroxisomal acyl-coenzyme A oxidase 1	74.376	8.16	17.348113	4.5454545	3	4	3	98.8	101.2	1.024	2.93E-01
Q15404	Ras suppressor protein 1	31.521	8.65	47.575825	22.743682	6	12	6	98.8	101.2	1.024	3.59E-01
Q15717	ELAV-like protein 1	36.069	9.17	49.612836	24.846626	7	20	7	98.8	101.2	1.024	5.79E-01
Q16222	UDP-N-acetylhexosamine pyrophosphorylase	58.732	6.33	83.472929	20.881226	10	17	9	98.8	101.2	1.024	1.10E-01
Q16543	Hsp90 co-chaperone Cdc37	44.44	5.25	157.7071	32.275132	11	35	11	98.8	101.2	1.024	1.21E-02
Q16658	Fascin	54.496	7.24	236.76666	49.69574	23	62	23	98.8	101.2	1.024	4.47E-02
Q49A26	Putative oxidoreductase GLYR1	60.518	9.17	44.763593	14.647378	6	7	6	98.8	101.2	1.024	9.95E-02
Q5GLZ8	Probable E3 ubiquitin-protein ligase HERC4	118.487	6.19	63.148405	9.6499527	8	12	8	98.8	101.2	1.024	1.14E-01

Q5R3I4	Tetratricopeptide repeat protein 38	52.753	5.99	43.359566	16.84435	6	9	6	98.8	101.2	1.024	1.17E-01
Q68E01	Integrator complex subunit 3	117.994	5.8	34.753716	5.9443912	5	6	5	98.8	101.2	1.024	2.27E-01
Q6GMV3	Putative peptidyl-tRNA hydrolase PTRHD1	15.795	9.1	32.470058	21.428571	4	6	4	98.8	101.2	1.024	2.05E-01
Q6PGP7	Tetratricopeptide repeat protein 37	175.375	7.53	84.102304	8.3759591	13	16	13	98.8	101.2	1.024	4.83E-02
Q6PJG6	BRCA1-associated ATM activator 1	88.063	5.27	21.088049	6.3337393	5	6	5	98.8	101.2	1.024	8.15E-01
Q6PL24	Protein TMED8	35.718	4.74	14.849205	11.076923	3	3	3	98.8	101.2	1.024	
Q6SPF0	Atherin	56.018	7.58	2.8706324	1.3011152	1	1	1	98.8	101.2	1.024	
Q6ZW76	Ankyrin repeat and SAM domain-containing protein 3	71.993	5.45	6.7126462	1.3719512	1	1	1	98.8	101.2	1.024	
Q7Z422	SUZ domain-containing protein 1	16.987	8.95	11.129075	21.710526	2	2	2	98.8	101.2	1.024	8.40E-03
Q86UY6	N-alpha-acetyltransferase 40	27.176	7.39	12.476821	5.0632911	1	2	1	98.8	101.2	1.024	
Q8N0U8	Vitamin K epoxide reductase complex subunit 1-like protein 1	19.823	9.13	8.4447933	5.6818182	1	2	1	98.8	101.2	1.024	
Q8N3D4	EH domain-binding protein 1-like protein 1	161.756	4.83	120.82831	15.692712	17	23	17	98.8	101.2	1.024	1.45E-02
Q8WUH1	Protein Churchill	16.101	5.54	21.820845	32.374101	4	4	4	98.8	101.2	1.024	1.89E-01
Q8WWQ0	PH-interacting protein	206.56	8.85	25.401784	3.4596376	5	5	5	98.8	101.2	1.024	7.02E-01
Q92879	CUGBP Elav-like family member 1	52.03	8.46	38.309227	12.345679	5	7	5	98.8	101.2	1.024	5.09E-01
Q92930	Ras-related protein Rab-8B	23.569	9.07	40.915767	24.154589	5	17	1	98.8	101.2	1.024	
Q93008	Probable ubiquitin carboxyl-terminal hydrolase FAF-X	292.094	5.8	180.57168	12.801556	31	38	31	98.8	101.2	1.024	2.24E-01
Q969V5	Mitochondrial ubiquitin ligase activator of NFKB 1	39.774	8.13	5.4490177	4.8295455	2	2	2	98.8	101.2	1.024	
Q96N66	Lysophospholipid acyltransferase 7	52.73	8.97	50.707234	14.618644	5	8	5	98.8	101.2	1.024	5.26E-01
Q96RT1	Protein LAP2	158.2	5.5	48.0651	7.7195467	8	8	8	98.8	101.2	1.024	1.94E-01
Q99460	26S proteasome non-ATPase regulatory subunit 1	105.769	5.39	147.93297	23.294858	20	31	20	98.8	101.2	1.024	2.09E-02
Q9BPX5	Actin-related protein 2/3 complex subunit 5-like protein	16.931	6.6	19.895869	28.75817	3	3	3	98.8	101.2	1.024	
Q9BV79	Trans-2-enoyl-CoA reductase, mitochondrial	40.436	8.76	5.2849163	3.2171582	1	1	1	98.8	101.2	1.024	
Q9BZQ6	ER degradation-enhancing alpha-mannosidase-like protein 3	104.598	4.93	10.722343	2.6824034	2	2	2	98.8	101.2	1.024	7.80E-01
Q9C035	Tripartite motif-containing protein 5	56.302	6.05	4.934815	3.4482759	2	2	2	98.8	101.2	1.024	
Q9H089	Large subunit GTPase 1 homolog	75.178	6.38	13.120806	4.4072948	3	3	3	98.8	101.2	1.024	2.24E-01
Q9H583	HEAT repeat-containing protein 1	242.215	6.54	103.69756	8.3022388	17	20	17	98.8	101.2	1.024	4.34E-02
Q9NV31	U3 small nucleolar ribonucleoprotein protein IMP3	21.837	9.5	9.1951093	11.413043	2	2	2	98.8	101.2	1.024	
Q9NWS0	PIH1 domain-containing protein 1	32.342	5.14	28.24299	22.068966	4	6	4	98.8	101.2	1.024	1.08E-02
Q9NYL2	Mitogen-activated protein kinase kinase kinase MLT	91.098	7.87	12.91094	2.75	2	2	2	98.8	101.2	1.024	3.44E-01
Q9NYM9	BET1-like protein	12.38	8.16	5.0128255	11.711712	1	1	1	98.8	101.2	1.024	
Q9UBS4	DnaJ homolog subfamily B member 11	40.489	6.18	101.52724	31.843575	9	17	9	98.8	101.2	1.024	6.70E-01
Q9UHI6	Probable ATP-dependent RNA helicase DDX20	92.183	6.95	78.15309	13.592233	9	13	9	98.8	101.2	1.024	1.84E-01
Q9UHX1	Poly(U)-binding-splicing factor PUF60	59.838	5.29	169.7513	29.516995	15	36	15	98.8	101.2	1.024	9.56E-03
Q9UJU6	Drebrin-like protein	48.178	5.05	128.25123	32.55814	11	20	11	98.8	101.2	1.024	4.66E-01
Q9UKX7	Nuclear pore complex protein Nup50	50.113	7.06	16.666168	7.0512821	3	3	3	98.8	101.2	1.024	9.51E-01
Q9UQ35	Serine/arginine repetitive matrix protein 2	299.438	12.06	198.69009	13.299419	27	38	27	98.8	101.2	1.024	7.54E-02
Q9Y2A7	Nck-associated protein 1	128.707	6.62	49.919421	8.1560284	9	10	9	98.8	101.2	1.024	6.32E-02
Q9Y5L4	Mitochondrial import inner membrane translocase subunit Tim13	10.493	8.18	29.603224	36.842105	3	9	3	98.8	101.2	1.024	2.47E-01
Q9Y5X1	Sorting nexin-9	66.55	5.58	10.753791	4.0336134	2	2	2	98.8	101.2	1.024	
E9PRG8	Uncharacterized protein C11orf98	13.79	11.69	11.9324	10.655738	1	2	1	98.7	101.3	1.026	
O14647	Chromodomain-helicase-DNA-binding protein 2	211.214	8.1	8.5808061	1.3129103	3	3	1	98.7	101.3	1.026	
O43414	ERI1 exoribonuclease 3	37.214	8.07	12.854883	3.5608309	1	3	1	98.7	101.3	1.026	
O43427	Acidic fibroblast growth factor intracellular-binding protein	41.851	6.48	7.9858997	2.7472527	1	1	1	98.7	101.3	1.026	
O43615	Mitochondrial import inner membrane translocase subunit TIM44	51.323	8.32	114.52279	27.654867	11	17	11	98.7	101.3	1.026	3.02E-01
O43913	Origin recognition complex subunit 5	50.251	7.74	21.539433	10.574713	5	7	5	98.7	101.3	1.026	5.77E-01
O60506	Heterogeneous nuclear ribonucleoprotein Q	69.56	8.59	341.09802	47.993579	26	91	18	98.7	101.3	1.026	1.04E-01

O75110	Probable phospholipid-transporting ATPase IIA	118.506	7.77	8.6839441	1.9102197	2	2	2	98.7	101.3	1.026	
O75182	Paired amphipathic helix protein Sin3b	132.983	6.93	5.1470323	0.8605852	1	1	1	98.7	101.3	1.026	
O75746	Calcium-binding mitochondrial carrier protein Aralar1	74.715	8.38	75.879519	15.781711	10	14	4	98.7	101.3	1.026	5.13E-02
O95340	Bifunctional 3'-phosphoadenosine 5'-phosphosulfate synthase 2	69.457	8.03	145.12482	33.713355	19	34	16	98.7	101.3	1.026	1.47E-01
O95391	Pre-mRNA-splicing factor SLU7	68.344	7.14	21.812711	3.5836177	2	3	2	98.7	101.3	1.026	
P04920	Anion exchange protein 2	136.923	6.29	24.746052	4.8348106	5	5	5	98.7	101.3	1.026	
P05141	ADP/ATP translocase 2	32.831	9.69	146.35207	38.926174	14	46	5	98.7	101.3	1.026	8.20E-02
P08865	40S ribosomal protein SA	32.833	4.87	114.62208	34.576271	8	24	8	98.7	101.3	1.026	3.46E-02
P12081	Histidine--tRNA ligase, cytoplasmic	57.374	5.88	116.08175	31.630648	18	23	13	98.7	101.3	1.026	5.26E-01
P17096	High mobility group protein HMG-I/HMG-Y	11.669	10.32	29.887409	48.598131	4	8	4	98.7	101.3	1.026	2.44E-02
P20839	Inosine-5'-monophosphate dehydrogenase 1	55.37	6.9	16.079647	10.505837	5	6	4	98.7	101.3	1.026	2.03E-01
P30260	Cell division cycle protein 27 homolog	91.809	7.02	58.13416	10.679612	7	9	7	98.7	101.3	1.026	4.55E-02
P30464	HLA class I histocompatibility antigen, B-15 alpha chain	40.363	6.3	55.345917	25.690608	7	15	1	98.7	101.3	1.026	
P31930	Cytochrome b-c1 complex subunit 1, mitochondrial	52.612	6.37	124.60306	30.833333	11	21	10	98.7	101.3	1.026	2.30E-01
P38159	RNA-binding motif protein, X chromosome	42.306	10.05	105.03386	37.851662	18	47	7	98.7	101.3	1.026	2.66E-02
P40692	DNA mismatch repair protein Mlh1	84.548	5.72	30.573922	11.243386	8	9	8	98.7	101.3	1.026	7.11E-02
P49590	Probable histidine--tRNA ligase, mitochondrial	56.852	8.24	64.115297	18.577075	9	14	4	98.7	101.3	1.026	2.13E-02
P49915	GMP synthase [glutamine-hydrolyzing]	76.667	6.87	215.82368	33.910534	24	50	24	98.7	101.3	1.026	2.18E-02
P50395	Rab GDP dissociation inhibitor beta	50.631	6.47	236.78196	51.011236	19	47	15	98.7	101.3	1.026	9.61E-01
P53041	Serine/threonine-protein phosphatase 5	56.842	6.28	86.739414	23.647295	12	14	12	98.7	101.3	1.026	6.67E-01
P62191	26S protease regulatory subunit 4	49.154	6.21	201.41132	44.772727	18	50	16	98.7	101.3	1.026	3.06E-01
P62826	GTP-binding nuclear protein Ran	24.408	7.49	95.248357	36.111111	9	34	9	98.7	101.3	1.026	1.70E-01
P78536	Disintegrin and metalloproteinase domain-containing protein 17	92.961	5.76	5.6513058	1.2135922	1	1	1	98.7	101.3	1.026	
Q08AD1	Calmodulin-regulated spectrin-associated protein 2	167.984	6.8	19.850211	3.3579584	4	4	4	98.7	101.3	1.026	3.22E-01
Q0VDF9	Heat shock 70 kDa protein 14	54.76	5.59	34.051441	9.6267191	6	8	5	98.7	101.3	1.026	2.98E-02
Q10713	Mitochondrial-processing peptidase subunit alpha	58.216	6.92	98.047545	24	11	17	11	98.7	101.3	1.026	8.32E-01
Q12849	G-rich sequence factor 1	53.093	6.19	52.913932	13.75	6	8	6	98.7	101.3	1.026	1.29E-01
Q13444	Disintegrin and metalloproteinase domain-containing protein 15	92.899	6.73	34.838457	7.8794902	5	6	5	98.7	101.3	1.026	7.78E-01
Q13867	Bleomycin hydrolase	52.528	6.27	37.506796	9.010989	4	9	4	98.7	101.3	1.026	7.04E-02
Q16864	V-type proton ATPase subunit F	13.362	5.52	20.161454	24.369748	3	4	3	98.7	101.3	1.026	
Q5H9R7	Serine/threonine-protein phosphatase 6 regulatory subunit 3	97.608	4.6	34.714347	8.2474227	7	7	7	98.7	101.3	1.026	
Q5VYK3	Proteasome-associated protein ECM29 homolog	204.16	7.12	225.33601	20.379404	30	41	30	98.7	101.3	1.026	4.89E-04
Q5VZM2	Ras-related GTP-binding protein B	43.223	6.21	12.405104	6.9518717	3	3	3	98.7	101.3	1.026	1.76E-01
Q6WKZ4	Rab11 family-interacting protein 1	137.083	5.43	6.9561502	2.182385	3	3	3	98.7	101.3	1.026	2.57E-01
Q70CQ2	Ubiquitin carboxyl-terminal hydrolase 34	403.973	5.82	9.8836633	0.7332205	3	3	3	98.7	101.3	1.026	2.68E-01
Q7RTV0	PHD finger-like domain-containing protein 5A	12.397	8.41	42.749782	61.818182	7	13	7	98.7	101.3	1.026	3.23E-01
Q7Z4H7	HAUS augmin-like complex subunit 6	108.553	6.47	16.499567	3.1413613	2	2	2	98.7	101.3	1.026	7.39E-02
Q86U86	Protein polybromo-1	192.825	6.89	37.918121	4.2628774	7	8	7	98.7	101.3	1.026	3.08E-01
Q8NFC6	Biorientation of chromosomes in cell division protein 1-like 1	330.266	5.08	51.241849	3.5070469	9	11	8	98.7	101.3	1.026	6.66E-02
Q8NFD5	AT-rich interactive domain-containing protein 1B	235.974	6.73	3.7139929	0.3577818	1	1	1	98.7	101.3	1.026	
Q8NI27	THO complex subunit 2	182.659	8.44	61.431942	7.0935342	11	15	11	98.7	101.3	1.026	1.32E-01
Q92692	Nectin-2	57.706	4.82	7.6731396	3.5315985	2	2	2	98.7	101.3	1.026	1.16E-02
Q96AT9	Ribulose-phosphate 3-epimerase	24.911	5.58	5.0097056	3.9473684	1	1	1	98.7	101.3	1.026	
Q96CS3	FAS-associated factor 2	52.591	5.62	77.287546	19.325843	6	11	6	98.7	101.3	1.026	7.00E-02
Q96SY0	von Willebrand factor A domain-containing protein 9	57.434	5.12	8.993962	4.8262548	1	1	1	98.7	101.3	1.026	
Q99832	T-complex protein 1 subunit eta	59.329	7.65	253.11282	44.198895	18	53	18	98.7	101.3	1.026	3.72E-02
Q99848	Probable rRNA-processing protein EBP2	34.83	10.1	85.197689	40.196078	13	20	13	98.7	101.3	1.026	3.07E-01

Q9BUL8	Programmed cell death protein 10	24.686	8.19	31.940627	10.377358	3	5	3	98.7	101.3	1.026	7.99E-04
Q9GZP8	Immortalization up-regulated protein	10.891	9.73	11.717671	9.4339623	1	6	1	98.7	101.3	1.026	
Q9H078	Caseinolytic peptidase B protein homolog	78.68	9.01	65.164454	15.417256	12	17	12	98.7	101.3	1.026	1.55E-01
Q9H2U2	Inorganic pyrophosphatase 2, mitochondrial	37.896	7.39	91.65967	35.928144	10	16	8	98.7	101.3	1.026	7.39E-02
Q9H6R0	Putative ATP-dependent RNA helicase DHX33	78.824	8.91	9.4105553	3.5360679	2	2	2	98.7	101.3	1.026	4.43E-01
Q9H875	PRKR-interacting protein 1	20.984	9.79	13.839446	10.326087	2	3	2	98.7	101.3	1.026	
Q9H9A5	CCR4-NOT transcription complex subunit 10	82.257	7.78	26.547863	6.1827957	4	5	4	98.7	101.3	1.026	6.97E-01
Q9HCN8	Stromal cell-derived factor 2-like protein 1	23.584	7.03	16.669398	12.217195	3	7	3	98.7	101.3	1.026	1.45E-02
Q9NP72	Ras-related protein Rab-18	22.963	5.24	94.050932	40.776699	7	15	7	98.7	101.3	1.026	3.47E-03
Q9NQW7	Xaa-Pro aminopeptidase 1	69.873	5.67	106.23957	22.150883	14	24	14	98.7	101.3	1.026	4.75E-03
Q9NU22	Midasin	632.42	5.68	64.469444	2.0729092	11	13	11	98.7	101.3	1.026	1.16E-01
Q9NUW8	Tyrosyl-DNA phosphodiesterase 1	68.377	7.65	6.2799245	2.4671053	2	3	2	98.7	101.3	1.026	4.37E-01
Q9NWT8	Aurora kinase A-interacting protein	22.34	10.76	10.196059	5.0251256	1	2	1	98.7	101.3	1.026	
Q9UBK8	Methionine synthase reductase	80.359	6.49	87.668836	17.517241	10	17	9	98.7	101.3	1.026	1.96E-03
Q9UDY2	Tight junction protein ZO-2	133.876	7.4	113.76458	17.647059	19	28	18	98.7	101.3	1.026	2.37E-01
Q9UN86	Ras GTPase-activating protein-binding protein 2	54.088	5.55	61.247934	22.614108	10	19	9	98.7	101.3	1.026	5.54E-01
Q9Y2B0	Protein canopy homolog 2	20.639	4.92	46.93669	31.318681	5	10	5	98.7	101.3	1.026	7.70E-02
Q9Y5J6	Mitochondrial import inner membrane translocase subunit Tim10 B	11.579	7.43	15.194532	24.271845	2	2	2	98.7	101.3	1.026	
Q9Y6G9	Cytoplasmic dynein 1 light intermediate chain 1	56.544	6.42	57.628544	16.443595	7	11	7	98.7	101.3	1.026	8.80E-02
Q9Y6H1	Coiled-coil-helix-coiled-coil-helix domain-containing protein 2	15.503	9.22	68.459088	33.774834	4	9	4	98.7	101.3	1.026	1.05E-01
A7E2V4	Zinc finger SWIM domain-containing protein 8	197.173	6.8	5.0597824	0.7621121	1	1	1	98.6	101.4	1.028	
O14744	Protein arginine N-methyltransferase 5	72.638	6.29	60.519098	14.756672	8	13	8	98.6	101.4	1.028	1.04E-01
O15371	Eukaryotic translation initiation factor 3 subunit D	63.932	6.05	113.67767	32.846715	13	23	13	98.6	101.4	1.028	7.59E-02
O43747	AP-1 complex subunit gamma-1	91.293	6.8	75.214342	9.4890511	8	16	8	98.6	101.4	1.028	8.74E-01
O60684	Importin subunit alpha-7	59.991	4.98	82.464459	20.522388	10	14	6	98.6	101.4	1.028	5.09E-01
O75223	Gamma-glutamylcyclotransferase	20.994	5.14	23.700826	12.234043	2	3	2	98.6	101.4	1.028	
O75448	Mediator of RNA polymerase II transcription subunit 24	110.234	6.95	9.5582934	2.1233569	2	2	2	98.6	101.4	1.028	6.43E-01
O75600	2-amino-3-ketobutyrate coenzyme A ligase, mitochondrial	45.256	8.05	6.5000381	2.6252983	1	1	1	98.6	101.4	1.028	
O75676	Ribosomal protein S6 kinase alpha-4	85.552	8.28	22.843908	7.3834197	6	6	4	98.6	101.4	1.028	3.79E-01
O95163	Elongator complex protein 1	150.159	5.94	114.47347	12.312312	12	17	12	98.6	101.4	1.028	4.67E-02
O95299	I dehydrogenase [ubiquinone] 1 alpha subcomplex subunit 10, mitocho	40.725	8.48	6.7729916	5.915493	2	2	2	98.6	101.4	1.028	1.94E-02
O95363	Phenylalanine--tRNA ligase, mitochondrial	52.324	7.46	9.393772	3.9911308	2	2	2	98.6	101.4	1.028	
P13807	Glycogen [starch] synthase, muscle	83.732	6.18	38.004944	7.1913161	5	7	5	98.6	101.4	1.028	3.12E-02
P15927	Replication protein A 32 kDa subunit	29.228	6.15	15.853239	9.6296296	2	5	2	98.6	101.4	1.028	2.99E-02
P18124	60S ribosomal protein L7	29.207	10.65	70.061356	29.032258	9	18	9	98.6	101.4	1.028	1.05E-02
P19174	1-phosphatidylinositol 4,5-bisphosphate phosphodiesterase gamma-1	148.438	6.05	49.318922	8.9147287	9	10	9	98.6	101.4	1.028	8.05E-03
P26639	Threonine--tRNA ligase, cytoplasmic	83.382	6.67	290.45441	39.834025	27	74	25	98.6	101.4	1.028	9.25E-01
P31153	S-adenosylmethionine synthase isoform type-2	43.633	6.48	85.270151	32.911392	10	19	10	98.6	101.4	1.028	1.46E-01
P32969	60S ribosomal protein L9	21.85	9.95	90.764418	39.0625	6	18	6	98.6	101.4	1.028	4.52E-02
P33176	Kinesin-1 heavy chain	109.617	6.51	353.45725	41.32918	35	60	35	98.6	101.4	1.028	1.57E-04
P43490	Nicotinamide phosphoribosyltransferase	55.487	7.15	76.279647	18.940937	8	12	8	98.6	101.4	1.028	1.19E-01
P46777	60S ribosomal protein L5	34.341	9.72	142.91451	49.494949	15	45	15	98.6	101.4	1.028	7.70E-03
P49588	Alanine--tRNA ligase, cytoplasmic	106.743	5.53	451.85669	42.355372	33	75	33	98.6	101.4	1.028	1.24E-01
P49756	RNA-binding protein 25	100.124	6.32	67.937331	14.709371	11	14	11	98.6	101.4	1.028	7.16E-03
P49903	Selenide, water dikinase 1	42.883	5.97	29.737345	12.5	4	6	4	98.6	101.4	1.028	1.89E-01
P51151	Ras-related protein Rab-9A	22.823	5.47	51.45056	29.353234	5	9	5	98.6	101.4	1.028	3.89E-01
P51665	26S proteasome non-ATPase regulatory subunit 7	37.002	6.77	50.632048	19.135802	7	10	7	98.6	101.4	1.028	1.15E-02

P51858	Hepatoma-derived growth factor	26.772	4.73	137.33858	49.166667	11	46	10	98.6	101.4	1.028	1.66E-01
P63244	Guanine nucleotide-binding protein subunit beta-2-like 1	35.055	7.69	187.96738	54.574132	16	59	16	98.6	101.4	1.028	1.33E-02
P82663	28S ribosomal protein S25, mitochondrial	20.103	8.82	20.54607	20.231214	3	6	3	98.6	101.4	1.028	1.01E-02
Q01804	OTU domain-containing protein 4	123.968	6.71	2.3432312	0.8976661	1	1	1	98.6	101.4	1.028	
Q12996	Cleavage stimulation factor subunit 3	82.869	8.12	42.004767	10.739191	7	10	7	98.6	101.4	1.028	2.25E-04
Q13200	26S proteasome non-ATPase regulatory subunit 2	100.136	5.2	177.45992	27.202643	24	46	24	98.6	101.4	1.028	4.44E-02
Q13356	Peptidyl-prolyl cis-trans isomerase-like 2	58.787	8.78	27.893409	14.038462	6	6	6	98.6	101.4	1.028	1.84E-01
Q13561	Dynactin subunit 2	44.204	5.21	133.01955	35.910224	11	21	11	98.6	101.4	1.028	5.95E-03
Q13823	Nucleolar GTP-binding protein 2	83.603	9.25	42.758946	9.0287278	5	8	5	98.6	101.4	1.028	9.64E-01
Q14151	Scaffold attachment factor B2	107.408	6.16	145.78651	22.665268	25	56	13	98.6	101.4	1.028	2.17E-03
Q14152	Eukaryotic translation initiation factor 3 subunit A	166.468	6.79	250.9897	29.450072	37	61	37	98.6	101.4	1.028	3.34E-03
Q14344	Guanine nucleotide-binding protein subunit alpha-13	44.022	8	26.856629	10.344828	4	10	3	98.6	101.4	1.028	3.85E-02
Q15024	Exosome complex component RRP42	31.801	5.19	19.955857	14.776632	3	3	3	98.6	101.4	1.028	
Q15398	Disks large-associated protein 5	95.056	9	18.140892	6.1465721	4	4	4	98.6	101.4	1.028	1.27E-01
Q69YH5	Cell division cycle-associated protein 2	112.606	8.4	15.562192	3.714565	3	3	2	98.6	101.4	1.028	
Q6P1N0	Coiled-coil and C2 domain-containing protein 1A	103.998	8.09	47.997552	12.407992	12	13	12	98.6	101.4	1.028	7.21E-02
Q86XP3	ATP-dependent RNA helicase DDX42	102.912	7.02	101.38301	18.017058	14	24	14	98.6	101.4	1.028	4.49E-01
Q8IWS0	PHD finger protein 6	41.264	8.68	35.482747	16.438356	6	9	6	98.6	101.4	1.028	2.45E-03
Q8IWT6	Volume-regulated anion channel subunit LRRC8A	94.139	7.94	17.830776	5.0617284	3	3	3	98.6	101.4	1.028	1.54E-01
Q8TCC3	39S ribosomal protein L30, mitochondrial	18.534	9.99	5.6471544	11.180124	2	2	2	98.6	101.4	1.028	
Q8TED1	Probable glutathione peroxidase 8	23.866	9.35	16.920955	16.746411	4	5	4	98.6	101.4	1.028	1.86E-01
Q8WUF8	Protein FAM172A	47.942	6.09	11.311771	5.2884615	2	2	2	98.6	101.4	1.028	
Q92552	28S ribosomal protein S27, mitochondrial	47.581	6.18	33.95625	14.492754	6	7	6	98.6	101.4	1.028	2.36E-01
Q93100	Phosphorylase b kinase regulatory subunit beta	124.805	6.95	5.9773626	1.4638609	2	2	2	98.6	101.4	1.028	
Q96CN7	Isochorismatase domain-containing protein 1	32.216	7.39	49.872875	21.47651	5	9	5	98.6	101.4	1.028	7.31E-03
Q96JG6	Syndetin	111.104	6.2	19.673708	4.6680498	4	4	4	98.6	101.4	1.028	2.24E-01
Q9BRQ8	Apoptosis-inducing factor 2	40.501	9.11	40.198965	14.745308	4	6	4	98.6	101.4	1.028	3.03E-01
Q9BYN0	Sulfiredoxin-1	14.25	8.19	15.60077	25.547445	3	3	3	98.6	101.4	1.028	5.64E-01
Q9H1Y0	Autophagy protein 5	32.426	5.77	3.9226321	3.2727273	1	1	1	98.6	101.4	1.028	
Q9H3K2	Growth hormone-inducible transmembrane protein	37.181	9.94	12.940223	2.8985507	1	4	1	98.6	101.4	1.028	
Q9H444	Charged multivesicular body protein 4b	24.935	4.82	69.001819	29.910714	7	13	7	98.6	101.4	1.028	3.86E-01
Q9H8Y5	Ankyrin repeat and zinc finger domain-containing protein 1	80.877	8.41	34.61959	10.743802	5	5	5	98.6	101.4	1.028	1.10E-01
Q9NP64	Nucleolar protein of 40 kDa	27.552	9.7	10.954051	9.5435685	2	2	2	98.6	101.4	1.028	2.65E-01
Q9NR33	DNA polymerase epsilon subunit 4	12.201	4.92	25.745975	38.461538	3	4	3	98.6	101.4	1.028	7.57E-01
Q9NUQ9	Protein FAM49B	36.725	6.06	107.35748	40.432099	11	17	11	98.6	101.4	1.028	1.04E-02
Q9NX47	E3 ubiquitin-protein ligase MARCH5	31.211	8.7	21.294622	10.431655	2	3	2	98.6	101.4	1.028	1.97E-01
Q9NZ45	CDGSH iron-sulfur domain-containing protein 1	12.191	9.09	65.29248	36.111111	4	13	4	98.6	101.4	1.028	9.56E-02
Q9NZL4	Hsp70-binding protein 1	39.449	5.21	41.008363	16.298343	4	6	4	98.6	101.4	1.028	3.54E-01
Q9UGR2	Zinc finger CCCH domain-containing protein 7B	111.506	7.17	4.3267026	0.9063444	1	1	1	98.6	101.4	1.028	
Q9UHQ9	NADH-cytochrome b5 reductase 1	34.073	9.38	21.595479	16.721311	4	4	4	98.6	101.4	1.028	6.70E-01
Q9UNW1	Multiple inositol polyphosphate phosphatase 1	55.016	7.81	24.475137	7.5975359	3	4	3	98.6	101.4	1.028	
Q9UPN9	E3 ubiquitin-protein ligase TRIM33	122.456	6.67	48.238844	5.501331	6	10	6	98.6	101.4	1.028	3.25E-01
Q9Y3P9	Rab GTPase-activating protein 1	121.66	5.25	17.902663	4.0224509	5	6	5	98.6	101.4	1.028	2.05E-01
Q9Y5B0	RNA polymerase II subunit A C-terminal domain phosphatase	104.335	5.27	8.6635403	1.6649324	1	1	1	98.6	101.4	1.028	
O00401	Neural Wiskott-Aldrich syndrome protein	54.793	7.93	8.347863	5.1485149	3	3	3	98.5	101.5	1.03	7.68E-01
O14979	Heterogeneous nuclear ribonucleoprotein D-like	46.409	9.57	122.95886	22.619048	10	28	8	98.5	101.5	1.03	8.06E-02
O43426	Synaptojanin-1	172.995	7.42	18.313367	2.0979021	3	3	3	98.5	101.5	1.03	1.43E-01

O43592	Exportin-T	109.893	5.39	123.54501	14.864865	12	20	12	98.5	101.5	1.03	1.43E-03
O75586	Mediator of RNA polymerase II transcription subunit 6	28.407	8.62	4.8377344	3.6585366	1	1	1	98.5	101.5	1.03	
O76031	`P-dependent Clp protease ATP-binding subunit clpX-like, mitochondı	69.181	7.58	113.18494	21.169036	11	19	11	98.5	101.5	1.03	4.40E-03
O94906	Pre-mRNA-processing factor 6	106.858	8.25	104.61258	18.809777	18	25	18	98.5	101.5	1.03	6.32E-01
O94979	Protein transport protein Sec31A	132.931	6.89	120.25956	14.180328	16	27	16	98.5	101.5	1.03	2.19E-01
O95071	E3 ubiquitin-protein ligase UBR5	309.158	5.85	70.808878	5.2518757	12	12	12	98.5	101.5	1.03	7.45E-02
O95229	ZW10 interactor	31.274	5.15	23.399235	7.5812274	2	3	2	98.5	101.5	1.03	2.99E-02
O96033	Molybdopterin synthase sulfur carrier subunit	9.749	4.72	8.9027452	28.409091	3	4	3	98.5	101.5	1.03	4.86E-01
P04818	Thymidylate synthase	35.693	7.01	35.580443	22.364217	6	9	6	98.5	101.5	1.03	5.97E-01
P08237	ATP-dependent 6-phosphofructokinase, muscle type	85.128	7.99	198.12143	26.923077	17	39	14	98.5	101.5	1.03	9.09E-06
P11717	Cation-independent mannose-6-phosphate receptor	274.199	5.94	132.96093	10.91931	23	27	23	98.5	101.5	1.03	4.96E-02
P11908	Ribose-phosphate pyrophosphokinase 2	34.747	6.61	119.55534	35.534591	10	22	5	98.5	101.5	1.03	3.23E-01
P12268	Inosine-5'-monophosphate dehydrogenase 2	55.77	6.9	202.32357	36.964981	18	36	17	98.5	101.5	1.03	1.32E-03
P17676	CCAAT/enhancer-binding protein beta	36.083	8.31	15.630278	8.115942	2	3	2	98.5	101.5	1.03	
P25786	Proteasome subunit alpha type-1	29.537	6.61	123.99521	42.585551	11	34	11	98.5	101.5	1.03	1.96E-01
P36954	DNA-directed RNA polymerase II subunit RPB9	14.514	5.14	20.964096	32	3	4	3	98.5	101.5	1.03	4.23E-01
P49454	Centromere protein F	367.537	5.07	119.26967	7.5389408	23	28	22	98.5	101.5	1.03	6.04E-04
P52815	39S ribosomal protein L12, mitochondrial	21.335	8.87	57.285049	39.89899	6	11	6	98.5	101.5	1.03	1.15E-01
P53384	Cytosolic Fe-S cluster assembly factor NUBP1	34.512	5.33	34.475978	26.5625	5	7	5	98.5	101.5	1.03	4.02E-01
P61289	Proteasome activator complex subunit 3	29.488	5.95	107.64157	38.976378	11	20	11	98.5	101.5	1.03	4.49E-01
P68366	Tubulin alpha-4A chain	49.892	5.06	358.44487	52.455357	19	107	7	98.5	101.5	1.03	5.36E-01
P83916	Chromobox protein homolog 1	21.405	4.93	78.82434	17.837838	3	13	2	98.5	101.5	1.03	9.90E-02
P84085	ADP-ribosylation factor 5	20.517	6.79	80.808895	45	8	22	5	98.5	101.5	1.03	2.70E-01
Q00688	Peptidyl-prolyl cis-trans isomerase FKBP3	25.161	9.28	24.416782	16.964286	4	6	4	98.5	101.5	1.03	1.38E-01
Q01085	Nucleolysin TIAR	41.564	7.74	60.280731	16.266667	5	10	3	98.5	101.5	1.03	1.97E-01
Q03001	Dystonin	860.127	5.25	47.792371	1.2417437	10	11	10	98.5	101.5	1.03	1.26E-01
Q05086	Ubiquitin-protein ligase E3A	100.623	5.22	36.947906	7.0857143	6	8	6	98.5	101.5	1.03	2.80E-01
Q09028	Histone-binding protein RBBP4	47.626	4.89	70.859056	29.411765	11	23	4	98.5	101.5	1.03	3.94E-01
Q09161	Nuclear cap-binding protein subunit 1	91.781	6.43	153.91407	27.468354	17	28	17	98.5	101.5	1.03	1.55E-02
Q13111	Chromatin assembly factor 1 subunit A	106.86	5.94	41.041198	8.2635983	5	6	5	98.5	101.5	1.03	8.80E-01
Q13371	Phosducin-like protein	34.26	4.73	7.4541518	7.3089701	2	2	2	98.5	101.5	1.03	
Q14108	Lysosome membrane protein 2	54.255	5.14	29.925534	11.087866	5	8	5	98.5	101.5	1.03	5.12E-03
Q14562	ATP-dependent RNA helicase DHX8	139.227	8.32	11.449151	2.1311475	2	2	2	98.5	101.5	1.03	5.13E-02
Q15652	Probable JmjC domain-containing histone demethylation protein 2C	284.349	7.87	3.0061231	0.3149606	1	1	1	98.5	101.5	1.03	
Q5JPE7	Nodal modulator 2	139.351	5.76	105.38398	13.812155	14	20	2	98.5	101.5	1.03	
Q5T1M5	FK506-binding protein 15	133.547	5.2	65.467653	6.9729286	8	11	8	98.5	101.5	1.03	9.26E-02
Q5VZL5	Zinc finger MYM-type protein 4	172.677	6.84	16.628105	3.1007752	5	5	5	98.5	101.5	1.03	2.72E-02
Q6VEQ5	WAS protein family homolog 2	50.281	5.71	14.084583	6.6666667	3	3	3	98.5	101.5	1.03	2.22E-02
Q8IYI6	Exocyst complex component 8	81.747	5.49	19.734099	6.6206897	4	4	4	98.5	101.5	1.03	8.01E-01
Q8NDX5	Polyhomeotic-like protein 3	106.096	6.62	9.696016	2.1363174	2	2	1	98.5	101.5	1.03	
Q8WUM0	Nuclear pore complex protein Nup133	128.898	5.1	113.94429	14.705882	14	19	14	98.5	101.5	1.03	1.97E-02
Q8WX93	Palladin	150.47	7.09	147.11148	15.907448	18	26	18	98.5	101.5	1.03	1.96E-03
Q92621	Nuclear pore complex protein Nup205	227.776	6.19	120.71094	10.039761	17	22	17	98.5	101.5	1.03	3.05E-02
Q92805	Golgin subfamily A member 1	88.13	5.27	40.749187	10.691004	7	7	7	98.5	101.5	1.03	3.10E-02
Q96HP0	Dedicator of cytokinesis protein 6	229.414	6.74	2.4308603	0.4396678	1	1	1	98.5	101.5	1.03	
Q99570	Phosphoinositide 3-kinase regulatory subunit 4	153.007	7.17	32.469272	4.1237113	6	7	6	98.5	101.5	1.03	5.31E-02
Q99961	Endophilin-A2	41.464	5.43	102.68647	31.521739	10	16	7	98.5	101.5	1.03	2.34E-03

Q9BQ95	utionarily conserved signaling intermediate in Toll pathway, mitochon	49.117	6.29	15.769403	5.5684455	2	3	2	98.5	101.5	1.03	
Q9BRZ2	E3 ubiquitin-protein ligase TRIM56	81.437	7.74	22.188578	5.8278146	3	3	3	98.5	101.5	1.03	9.71E-01
Q9BSV6	tRNA-splicing endonuclease subunit Sen34	33.631	8.43	21.172439	8.7096774	2	5	2	98.5	101.5	1.03	5.94E-04
Q9BUP0	EF-hand domain-containing protein D1	26.911	5.39	71.761652	30.125523	6	15	4	98.5	101.5	1.03	2.78E-01
Q9BZF1	Oxysterol-binding protein-related protein 8	101.132	6.96	14.499536	4.2744657	3	3	3	98.5	101.5	1.03	7.82E-01
Q9C0C2	182 kDa tankyrase-1-binding protein	181.685	4.86	220.58604	21.110468	27	36	27	98.5	101.5	1.03	3.91E-01
Q9H0E3	Histone deacetylase complex subunit SAP130	110.255	9.83	5.8315025	0.9541985	1	1	1	98.5	101.5	1.03	
Q9H1A4	Anaphase-promoting complex subunit 1	216.361	6.3	31.524524	4.4238683	7	7	7	98.5	101.5	1.03	4.61E-01
Q9H7D7	WD repeat-containing protein 26	72.079	6.16	22.670678	8.9258699	5	5	5	98.5	101.5	1.03	8.38E-02
Q9H8H0	Nucleolar protein 11	81.072	6.07	74.458669	15.577191	11	14	11	98.5	101.5	1.03	4.65E-03
Q9H8Y8	Golgi reassembly-stacking protein 2	47.116	4.82	33.258275	15.486726	5	6	5	98.5	101.5	1.03	3.00E-02
Q9H944	Mediator of RNA polymerase II transcription subunit 20	23.206	6.87	25.418683	25	5	5	5	98.5	101.5	1.03	5.98E-02
Q9NVI1	Fanconi anemia group I protein	149.229	6.74	73.803473	9.6385542	12	14	12	98.5	101.5	1.03	3.18E-03
Q9UHB9	Signal recognition particle subunit SRP68	70.686	8.56	184.18516	34.290271	19	29	19	98.5	101.5	1.03	8.38E-02
Q9UIQ6	Leucyl-cystinyl aminopeptidase	117.274	5.73	9.9142184	2.4390244	2	3	2	98.5	101.5	1.03	
Q9UNX4	WD repeat-containing protein 3	106.032	6.64	68.607815	11.983033	11	13	11	98.5	101.5	1.03	2.04E-01
Q9Y266	Nuclear migration protein nudC	38.219	5.38	185.72494	44.712991	17	52	17	98.5	101.5	1.03	2.77E-01
Q9Y2U8	Inner nuclear membrane protein Man1	99.935	7.55	22.201359	4.5005488	4	5	4	98.5	101.5	1.03	7.66E-02
Q9Y547	Intraflagellar transport protein 25 homolog	16.287	5.03	28.699246	10.416667	2	6	2	98.5	101.5	1.03	2.21E-03
O00159	Unconventional myosin-Ic	121.606	9.41	145.44664	22.107244	20	37	20	98.4	101.6	1.033	4.01E-02
O00442	RNA 3'-terminal phosphate cyclase	39.311	7.85	11.386992	7.3770492	3	3	3	98.4	101.6	1.033	
O15287	Fanconi anemia group G protein	68.511	5.47	5.5749551	2.2508039	1	1	1	98.4	101.6	1.033	
O43776	Asparagine--tRNA ligase, cytoplasmic	62.903	6.25	116.65209	27.554745	12	21	12	98.4	101.6	1.033	1.85E-02
O60488	Long-chain-fatty-acid--CoA ligase 4	79.137	8.38	81.301095	18.002813	11	15	9	98.4	101.6	1.033	9.70E-03
O75663	TIP41-like protein	31.424	5.91	30.904177	17.279412	5	9	5	98.4	101.6	1.033	2.15E-01
O75844	CAAX prenyl protease 1 homolog	54.778	7.49	49.423498	17.052632	7	12	7	98.4	101.6	1.033	5.89E-02
P04632	Calpain small subunit 1	28.298	5.2	60.329549	18.283582	4	13	4	98.4	101.6	1.033	3.79E-01
P08243	Asparagine synthetase [glutamine-hydrolyzing]	64.329	6.86	47.612365	13.547237	8	12	8	98.4	101.6	1.033	1.53E-02
P13639	Elongation factor 2	95.277	6.83	505.56049	46.620047	41	178	40	98.4	101.6	1.033	9.57E-03
P24534	Elongation factor 1-beta	24.748	4.67	97.92847	39.111111	9	25	6	98.4	101.6	1.033	4.05E-01
P26885	Peptidyl-prolyl cis-trans isomerase FKBP2	15.639	9.13	40.262933	23.943662	3	9	3	98.4	101.6	1.033	1.57E-01
P43007	Neutral amino acid transporter A	55.688	6.25	13.742281	5.2631579	3	3	3	98.4	101.6	1.033	1.71E-01
P48147	Prolyl endopeptidase	80.648	5.86	28.355602	10.84507	7	8	7	98.4	101.6	1.033	
P49257	Protein ERGIC-53	57.513	6.77	75.427503	22.352941	12	19	11	98.4	101.6	1.033	2.21E-01
P49902	Cytosolic purine 5'-nucleotidase	64.928	6.14	36.246531	10.338681	6	8	6	98.4	101.6	1.033	5.89E-01
P52306	Rap1 GTPase-GDP dissociation stimulator 1	66.275	5.31	89.738578	21.581549	11	16	11	98.4	101.6	1.033	5.27E-02
P57721	Poly(rC)-binding protein 3	39.44	8.07	93.8732	21.293801	7	25	1	98.4	101.6	1.033	
P62753	40S ribosomal protein S6	28.663	10.84	84.155724	30.923695	9	23	9	98.4	101.6	1.033	4.88E-03
P62841	40S ribosomal protein S15	17.029	10.39	14.54965	12.413793	2	4	2	98.4	101.6	1.033	
P62873	Guanine nucleotide-binding protein G(I)/G(S)/G(T) subunit beta-1	37.353	6	78.508881	27.352941	8	21	4	98.4	101.6	1.033	5.68E-01
P78344	Eukaryotic translation initiation factor 4 gamma 2	102.297	7.14	164.67196	22.932745	19	38	19	98.4	101.6	1.033	1.97E-01
P82932	28S ribosomal protein S6, mitochondrial	14.218	9.26	11.926726	16.8	2	3	2	98.4	101.6	1.033	1.98E-01
Q01105	Protein SET	33.469	4.32	109.58742	39.655172	10	24	10	98.4	101.6	1.033	8.12E-02
Q02790	Peptidyl-prolyl cis-trans isomerase FKBP4	51.772	5.43	207.53919	44.008715	18	42	17	98.4	101.6	1.033	2.69E-05
Q07617	Sperm-associated antigen 1	103.574	6.86	7.0562378	1.9438445	2	2	1	98.4	101.6	1.033	
Q10570	Cleavage and polyadenylation specificity factor subunit 1	160.782	6.4	27.304633	3.049203	4	6	4	98.4	101.6	1.033	5.65E-01
Q12986	Transcriptional repressor NF-X1	124.312	8.24	30.013193	4.2857143	4	5	4	98.4	101.6	1.033	2.00E-01

Q13112	Chromatin assembly factor 1 subunit B	61.454	7.5	18.173171	8.2289803	4	5	4	98.4	101.6	1.033	7.34E-02
Q13671	Ras and Rab interactor 1	84.047	8.02	12.991196	5.4916986	4	4	4	98.4	101.6	1.033	
Q14738	e/threonine-protein phosphatase 2A 56 kDa regulatory subunit delta iso	69.947	8.13	78.464766	18.770764	9	14	7	98.4	101.6	1.033	3.78E-04
Q14C86	GTPase-activating protein and VPS9 domain-containing protein 1	164.876	5.22	126.95764	14.411367	17	24	17	98.4	101.6	1.033	2.12E-05
Q15006	ER membrane protein complex subunit 2	34.811	6.57	23.037441	12.457912	3	4	3	98.4	101.6	1.033	5.53E-01
Q15154	Pericentriolar material 1 protein	228.392	5.02	54.055285	5.2371542	11	11	11	98.4	101.6	1.033	9.33E-03
Q5TFE4	5'-nucleotidase domain-containing protein 1	51.812	6.35	34.13635	12.747253	4	5	4	98.4	101.6	1.033	1.06E-01
Q6NXE6	Armadillo repeat-containing protein 6	54.107	6.24	15.467465	5.5888224	2	3	2	98.4	101.6	1.033	3.04E-01
Q6P3X3	Tetratricopeptide repeat protein 27	96.571	5.59	41.299342	5.9311981	5	6	5	98.4	101.6	1.033	2.58E-01
Q7Z6K5	Arpin	24.927	5.83	5.5203189	8.8495575	2	2	2	98.4	101.6	1.033	8.21E-01
Q86XZ4	Spermatogenesis-associated serine-rich protein 2	59.508	8.9	59.702846	15.59633	7	9	7	98.4	101.6	1.033	1.06E-01
Q86Y56	Dynein assembly factor 5, axonemal	93.462	6.42	89.386525	18.011696	13	17	13	98.4	101.6	1.033	3.23E-03
Q8IV38	Ankyrin repeat and MYND domain-containing protein 2	49.267	6.25	42.860226	14.965986	6	8	6	98.4	101.6	1.033	9.18E-01
Q8IX18	Probable ATP-dependent RNA helicase DHX40	88.504	8.65	6.4337983	1.6688062	1	1	1	98.4	101.6	1.033	
Q8IYS1	Peptidase M20 domain-containing protein 2	47.746	5.85	7.6572655	4.3577982	2	2	2	98.4	101.6	1.033	
Q8TAA5	GrpE protein homolog 2, mitochondrial	25.415	7.72	8.3978427	8.4444444	2	2	2	98.4	101.6	1.033	
Q92878	DNA repair protein RAD50	153.797	6.89	110.38651	13.262195	18	23	18	98.4	101.6	1.033	1.92E-01
Q96AC1	Fermitin family homolog 2	77.811	6.7	138.92942	25.588235	16	26	15	98.4	101.6	1.033	6.65E-01
Q96FV9	THO complex subunit 1	75.619	4.98	45.816008	11.719939	6	8	6	98.4	101.6	1.033	6.39E-01
Q96J01	THO complex subunit 3	38.747	6.09	21.263714	10.25641	4	6	4	98.4	101.6	1.033	1.29E-01
Q99717	Mothers against decapentaplegic homolog 5	52.225	7.71	16.679635	6.2365591	3	4	3	98.4	101.6	1.033	1.89E-01
Q9BYD1	39S ribosomal protein L13, mitochondrial	20.679	9.16	11.856922	17.977528	3	3	3	98.4	101.6	1.033	
Q9H6X2	Anthrax toxin receptor 1	62.749	7.61	4.0797201	2.3049645	1	1	1	98.4	101.6	1.033	
Q9NRP2	COX assembly mitochondrial protein 2 homolog	9.454	7.87	8.5438682	32.911392	3	3	3	98.4	101.6	1.033	
Q9NZU5	LIM and cysteine-rich domains protein 1	40.806	7.93	12.798486	8.7671233	4	4	4	98.4	101.6	1.033	5.30E-01
Q9P1Z2	Calcium-binding and coiled-coil domain-containing protein 1	77.289	4.82	2.8477117	1.5918958	1	1	1	98.4	101.6	1.033	
Q9P2N7	Kelch-like protein 13	73.82	6.62	33.166128	6.7175573	4	6	4	98.4	101.6	1.033	1.06E-01
Q9UMZ2	Synergin gamma	140.566	5.03	4.9096164	1.369863	2	2	1	98.4	101.6	1.033	
Q9Y3E5	Peptidyl-tRNA hydrolase 2, mitochondrial	19.181	8.73	32.918437	21.22905	2	5	2	98.4	101.6	1.033	8.73E-01
Q9Y696	Chloride intracellular channel protein 4	28.754	5.59	96.198001	43.083004	9	24	8	98.4	101.6	1.033	7.32E-01
Q9Y6E2	Basic leucine zipper and W2 domain-containing protein 2	48.132	6.68	117.81981	36.038186	17	26	15	98.4	101.6	1.033	4.15E-04
A0MZ66	Shootin-1	71.596	5.33	119.56555	26.941363	14	22	13	98.3	101.7	1.035	2.42E-02
O14920	Inhibitor of nuclear factor kappa-B kinase subunit beta	86.509	5.78	23.161287	4.4973545	3	3	3	98.3	101.7	1.035	3.97E-02
O43395	U4/U6 small nuclear ribonucleoprotein Prp3	77.481	9.5	115.28886	29.136164	17	22	17	98.3	101.7	1.035	7.10E-02
O43684	Mitotic checkpoint protein BUB3	37.131	6.84	92.501888	31.097561	9	22	9	98.3	101.7	1.035	6.60E-01
O60568	Procollagen-lysine,2-oxoglutarate 5-dioxygenase 3	84.731	6.05	87.781389	16.937669	10	16	10	98.3	101.7	1.035	9.39E-02
O75323	Protein NipSnap homolog 2	33.721	9.36	20.284133	11.888112	4	6	3	98.3	101.7	1.035	
O75940	Survival of motor neuron-related-splicing factor 30	26.694	7.24	18.681653	9.2436975	2	3	2	98.3	101.7	1.035	
O75947	ATP synthase subunit d, mitochondrial	18.479	5.3	99.738023	75.15528	11	25	11	98.3	101.7	1.035	4.88E-03
O95361	Tripartite motif-containing protein 16	63.915	5.49	23.305148	8.5106383	3	3	3	98.3	101.7	1.035	2.93E-01
P09001	39S ribosomal protein L3, mitochondrial	38.608	9.48	3.846756	4.5977011	2	2	2	98.3	101.7	1.035	
P13693	Translationally-controlled tumor protein	19.583	4.93	109.84252	38.372093	6	27	6	98.3	101.7	1.035	1.08E-01
P18621	60S ribosomal protein L17	21.383	10.17	38.491154	32.608696	7	12	7	98.3	101.7	1.035	2.14E-01
P28062	Proteasome subunit beta type-8	30.335	7.43	13.73813	7.2463768	2	4	2	98.3	101.7	1.035	
P29692	Elongation factor 1-delta	31.103	5.01	112.90619	38.078292	12	22	9	98.3	101.7	1.035	1.98E-01
P42695	Condensin-2 complex subunit D3	168.783	7.5	22.410518	2.4032043	3	4	3	98.3	101.7	1.035	9.50E-01
P49840	Glycogen synthase kinase-3 alpha	50.949	8.75	60.349285	22.360248	7	9	4	98.3	101.7	1.035	1.03E-03

P50750	Cyclin-dependent kinase 9	42.75	8.79	61.111942	33.870968	11	12	9	98.3	101.7	1.035	8.14E-02
P51668	Ubiquitin-conjugating enzyme E2 D1	16.591	7.42	11.926155	7.4829932	1	2	1	98.3	101.7	1.035	
P56377	AP-1 complex subunit sigma-2	18.603	5.47	5.6635403	5.7324841	1	1	1	98.3	101.7	1.035	
P56556	NADH dehydrogenase [ubiquinone] 1 alpha subcomplex subunit 6	17.859	10.14	10.5752	10.38961	2	3	2	98.3	101.7	1.035	5.13E-02
P60520	Gamma-aminobutyric acid receptor-associated protein-like 2	13.658	8.1	4.2939653	9.4017094	1	1	1	98.3	101.7	1.035	
P62829	60S ribosomal protein L23	14.856	10.51	116.08942	57.857143	8	33	8	98.3	101.7	1.035	9.08E-01
P62995	Transformer-2 protein homolog beta	33.646	11.25	61.297623	23.263889	7	14	5	98.3	101.7	1.035	4.69E-03
Q00796	Sorbitol dehydrogenase	38.3	7.97	59.48279	20.448179	6	10	6	98.3	101.7	1.035	7.06E-03
Q12979	Active breakpoint cluster region-related protein	97.536	6.55	27.231937	3.6088475	3	4	3	98.3	101.7	1.035	
Q13325	Interferon-induced protein with tetratricopeptide repeats 5	55.812	7.4	44.201978	15.145228	5	6	5	98.3	101.7	1.035	6.30E-02
Q13459	Unconventional myosin-IXb	243.249	8.75	21.199155	2.364395	5	5	5	98.3	101.7	1.035	1.81E-01
Q13555	Calcium/calmodulin-dependent protein kinase type II subunit gamma	62.57	7.83	49.26063	12.007168	6	10	3	98.3	101.7	1.035	6.67E-02
Q14137	Ribosome biogenesis protein BOP1	83.577	6.19	50.056416	10.857909	6	9	6	98.3	101.7	1.035	2.16E-02
Q14202	Zinc finger MYM-type protein 3	152.28	6.35	18.74446	3.1386861	3	4	3	98.3	101.7	1.035	1.12E-01
Q14696	LDLR chaperone MESD	26.06	7.78	13.245334	14.529915	3	3	3	98.3	101.7	1.035	1.52E-02
Q16204	Coiled-coil domain-containing protein 6	53.258	7.34	124.72274	32.700422	15	21	15	98.3	101.7	1.035	5.34E-02
Q2TB90	Putative hexokinase HKDC1	102.478	7.12	106.99308	21.374046	18	22	18	98.3	101.7	1.035	1.13E-02
Q5T5Y3	Calmodulin-regulated spectrin-associated protein 1	177.861	6.73	15.277612	1.9975031	3	3	3	98.3	101.7	1.035	4.94E-01
Q6KC79	Nipped-B-like protein	315.854	7.91	64.395966	3.9942939	11	13	11	98.3	101.7	1.035	1.12E-01
Q6P2H3	Centrosomal protein of 85 kDa	85.586	6	22.390195	5.1181102	3	4	2	98.3	101.7	1.035	1.02E-04
Q6UUV7	CREB-regulated transcription coactivator 3	66.918	6.84	25.550038	5.0080775	3	4	3	98.3	101.7	1.035	9.56E-02
Q86TI2	Dipeptidyl peptidase 9	98.201	6.46	20.510104	3.9397451	3	3	3	98.3	101.7	1.035	3.31E-01
Q86TU7	Histone-lysine N-methyltransferase setd3	67.215	5.96	69.869795	21.885522	11	15	11	98.3	101.7	1.035	8.11E-01
Q86W92	Liprin-beta-1	113.952	5.55	52.408526	9.0009891	7	10	7	98.3	101.7	1.035	2.65E-01
Q8IY37	Probable ATP-dependent RNA helicase DHX37	129.464	8.1	24.772787	4.9265341	5	5	5	98.3	101.7	1.035	2.93E-01
Q92530	Proteasome inhibitor PI31 subunit	29.798	5.74	4.6959492	6.6420664	2	2	2	98.3	101.7	1.035	2.99E-02
Q92542	Nicastrin	78.362	5.99	19.52534	2.6798307	2	3	2	98.3	101.7	1.035	
Q92785	Zinc finger protein ubi-d4	44.127	6.33	52.319004	16.368286	4	8	4	98.3	101.7	1.035	6.34E-03
Q92820	Gamma-glutamyl hydrolase	35.941	7.11	7.0284922	4.0880503	1	1	1	98.3	101.7	1.035	
Q96DA6	Mitochondrial import inner membrane translocase subunit TIM14	12.491	10.1	20.120944	25.862069	3	4	3	98.3	101.7	1.035	1.12E-01
Q99615	DnaJ homolog subfamily C member 7	56.405	6.96	117.51132	34.008097	18	29	18	98.3	101.7	1.035	1.86E-01
Q9BR76	Coronin-1B	54.2	5.88	62.786148	16.359918	9	15	9	98.3	101.7	1.035	2.18E-03
Q9BT25	HAUS augmin-like complex subunit 8	44.83	7.06	7.4805658	3.4146341	1	1	1	98.3	101.7	1.035	
Q9BUR4	Telomerase Cajal body protein 1	59.272	4.58	13.495175	5.1094891	2	2	2	98.3	101.7	1.035	
Q9BZD4	Kinetochore protein Nuf2	54.269	8.27	24.015867	6.4655172	3	4	3	98.3	101.7	1.035	1.62E-01
Q9C005	Protein dpy-30 homolog	11.243	4.88	11.692619	25.252525	2	2	2	98.3	101.7	1.035	2.83E-02
Q9H2W6	39S ribosomal protein L46, mitochondrial	31.685	7.05	33.937019	17.921147	4	5	4	98.3	101.7	1.035	5.85E-01
Q9NVP2	Histone chaperone ASF1B	22.419	4.56	7.9318307	13.861386	2	2	2	98.3	101.7	1.035	
Q9NXV6	CDKN2A-interacting protein	61.088	9.01	66.266049	16.034483	8	10	8	98.3	101.7	1.035	1.70E-02
Q9P0I2	ER membrane protein complex subunit 3	29.932	6.81	7.1879909	6.1302682	2	2	2	98.3	101.7	1.035	
Q9Y2Q9	28S ribosomal protein S28, mitochondrial	20.83	9.1	17.082638	23.529412	5	5	5	98.3	101.7	1.035	
Q9Y6G5	COMM domain-containing protein 10	22.952	6.54	3.1865525	5.9405941	1	1	1	98.3	101.7	1.035	
A0AVT1	Ubiquitin-like modifier-activating enzyme 6	117.895	6.14	125.56399	21.577947	20	24	20	98.2	101.8	1.037	4.78E-03
O00148	ATP-dependent RNA helicase DDX39A	49.098	5.68	93.852752	21.077283	10	22	5	98.2	101.8	1.037	4.29E-01
O14802	DNA-directed RNA polymerase III subunit RPC1	155.542	8.48	36.393832	6.3309353	7	8	6	98.2	101.8	1.037	2.84E-01
O15427	Monocarboxylate transporter 4	49.437	7.96	22.775312	8.8172043	4	7	4	98.2	101.8	1.037	2.56E-01
O15541	RING finger protein 113A	38.763	5.69	11.486554	8.7463557	2	2	1	98.2	101.8	1.037	

O43156	TELO2-interacting protein 1 homolog	121.992	5.97	42.840909	7.6216713	7	9	7	98.2	101.8	1.037	2.87E-02
O43815	Striatin	86.079	5.27	43.358621	12.820513	7	7	7	98.2	101.8	1.037	7.59E-01
O75347	Tubulin-specific chaperone A	12.847	5.29	49.816739	49.074074	7	13	7	98.2	101.8	1.037	1.50E-01
O75874	Isocitrate dehydrogenase [NADP] cytoplasmic	46.63	7.01	153.48429	35.507246	17	39	16	98.2	101.8	1.037	1.36E-04
O95197	Reticulon-3	112.541	4.96	16.476731	2.8100775	3	5	3	98.2	101.8	1.037	
P06493	Cyclin-dependent kinase 1	34.074	8.4	142.28781	59.259259	15	33	12	98.2	101.8	1.037	1.34E-03
P07737	Profilin-1	15.045	8.27	128.1739	62.142857	10	49	10	98.2	101.8	1.037	1.49E-06
P13995	ional methylenetetrahydrofolate dehydrogenase/cyclohydrolase, mitocl	37.871	8.73	46.064482	28.285714	9	9	9	98.2	101.8	1.037	9.25E-03
P25789	Proteasome subunit alpha type-4	29.465	7.72	113.31056	43.295019	10	27	10	98.2	101.8	1.037	7.46E-01
P30626	Sorcin	21.662	5.59	53.499371	31.818182	6	15	6	98.2	101.8	1.037	2.03E-02
P30876	DNA-directed RNA polymerase II subunit RPB2	133.811	6.87	55.67253	11.754685	11	12	11	98.2	101.8	1.037	9.42E-01
P31949	Protein S100-A11	11.733	7.12	49.181623	49.52381	5	19	5	98.2	101.8	1.037	1.23E-02
P38435	Vitamin K-dependent gamma-carboxylase	87.505	8.02	11.980519	3.6939314	2	3	2	98.2	101.8	1.037	
P42771	Cyclin-dependent kinase inhibitor 2A	16.522	5.81	23.18312	26.282051	3	4	3	98.2	101.8	1.037	5.01E-01
P49459	Ubiquitin-conjugating enzyme E2 A	17.305	5.15	13.423211	17.763158	2	3	2	98.2	101.8	1.037	8.93E-01
P49755	Transmembrane emp24 domain-containing protein 10	24.96	7.44	16.309041	13.69863	3	7	3	98.2	101.8	1.037	1.53E-01
P51532	Transcription activator BRG1	184.53	7.88	42.799704	6.4966606	10	11	10	98.2	101.8	1.037	1.70E-02
P51649	Succinate-semialdehyde dehydrogenase, mitochondrial	57.178	8.28	70.31322	16.82243	8	13	8	98.2	101.8	1.037	5.16E-02
P51808	Dynein light chain Tctex-type 3	13.053	5.66	19.181828	14.655172	1	2	1	98.2	101.8	1.037	
P60602	Reactive oxygen species modulator 1	8.177	9.33	9.4136016	21.518987	1	2	1	98.2	101.8	1.037	
P60900	Proteasome subunit alpha type-6	27.382	6.76	128.82226	44.715447	11	31	11	98.2	101.8	1.037	4.35E-01
P61803	chyl-diphosphooligosaccharide--protein glycosyltransferase subunit D.	12.489	7.08	4.3424661	10.619469	1	1	1	98.2	101.8	1.037	
P98170	E3 ubiquitin-protein ligase XIAP	56.648	6.65	7.8877302	2.8169014	1	1	1	98.2	101.8	1.037	
Q01415	N-acetylgalactosamine kinase	50.346	6.61	10.232303	6.768559	3	3	3	98.2	101.8	1.037	
Q07866	Kinesin light chain 1	65.269	6.2	129.78379	29.842932	17	28	13	98.2	101.8	1.037	9.97E-02
Q10472	Polypeptide N-acetylgalactosaminyltransferase 1	64.177	7.72	33.457072	9.8389982	5	7	4	98.2	101.8	1.037	3.56E-01
Q12899	Tripartite motif-containing protein 26	62.127	5.03	42.473719	10.760668	5	7	5	98.2	101.8	1.037	1.51E-02
Q14534	Squalene monooxygenase	63.882	8.63	4.1825669	2.6132404	1	1	1	98.2	101.8	1.037	
Q15046	Lysine--tRNA ligase	68.005	6.35	146.88468	28.81072	17	40	17	98.2	101.8	1.037	5.96E-02
Q15070	Mitochondrial inner membrane protein OXA1L	48.516	9.45	24.106249	8.2758621	4	5	4	98.2	101.8	1.037	5.34E-01
Q4VC31	Coiled-coil domain-containing protein 58	16.609	7.81	38.007959	31.25	4	6	4	98.2	101.8	1.037	2.30E-01
Q5QJE6	Deoxynucleotidyltransferase terminal-interacting protein 2	84.418	6.16	21.740246	6.6137566	6	6	6	98.2	101.8	1.037	1.38E-01
Q5SRE5	Nucleoporin NUP188 homolog	195.917	6.73	92.812448	10.520297	16	18	15	98.2	101.8	1.037	8.43E-03
Q5VV41	Rho guanine nucleotide exchange factor 16	80.055	7.36	74.314107	15.373766	9	11	9	98.2	101.8	1.037	2.67E-02
Q5VZE5	N-alpha-acetyltransferase 35, NatC auxiliary subunit	83.584	7.05	12.003882	4.6896552	3	3	3	98.2	101.8	1.037	
Q6ICB0	Desumoylating isopeptidase 1	18.251	4.94	7.3050369	4.7619048	1	2	1	98.2	101.8	1.037	
Q6P2Q9	Pre-mRNA-processing-splicing factor 8	273.427	8.84	301.58365	19.100642	40	64	40	98.2	101.8	1.037	1.21E-05
Q6RFH5	WD repeat-containing protein 74	42.415	8.32	33.551614	12.987013	4	7	4	98.2	101.8	1.037	4.58E-02
Q6ZNB6	NF-X1-type zinc finger protein NFXL1	101.27	8.41	10.723419	3.402854	2	2	2	98.2	101.8	1.037	2.74E-01
Q8IWZ8	SURP and G-patch domain-containing protein 1	72.425	7.61	31.644052	11.007752	5	5	5	98.2	101.8	1.037	3.65E-04
Q8IXT5	RNA-binding protein 12B	118.03	6.81	43.966085	10.589411	6	9	6	98.2	101.8	1.037	7.32E-01
Q8NBU5	ATPase family AAA domain-containing protein 1	40.718	6.9	64.496487	24.930748	9	15	9	98.2	101.8	1.037	1.00E+00
Q8WTW3	Conserved oligomeric Golgi complex subunit 1	108.909	7.31	7.9208596	2.1428571	2	2	2	98.2	101.8	1.037	
Q8WWK9	Cytoskeleton-associated protein 2	76.939	9.41	13.921755	1.9033675	1	2	1	98.2	101.8	1.037	
Q8WWY3	U4/U6 small nuclear ribonucleoprotein Prp31	55.421	5.78	17.438966	8.2164329	5	5	5	98.2	101.8	1.037	5.83E-01
Q96DH6	RNA-binding protein Musashi homolog 2	35.174	8.48	40.994762	11.585366	3	8	3	98.2	101.8	1.037	1.14E-01
Q96RP9	Elongation factor G, mitochondrial	83.418	7.01	153.756	32.756325	21	28	21	98.2	101.8	1.037	3.03E-02

Q96T51	RUN and FYVE domain-containing protein 1	79.767	5.74	51.810861	12.288136	8	11	8	98.2	101.8	1.037	1.98E-02
Q9BX10	GTP-binding protein 2	65.727	8.05	5.5902359	2.3255814	1	1	1	98.2	101.8	1.037	
Q9GZT8	NIF3-like protein 1	41.942	6.65	12.705038	6.8965517	3	3	3	98.2	101.8	1.037	7.17E-02
Q9H330	Transmembrane protein 245	100.881	8.87	6.7231463	2.3051592	2	2	2	98.2	101.8	1.037	
Q9H3P2	Negative elongation factor A	57.241	9.03	26.693078	4.7348485	2	4	2	98.2	101.8	1.037	1.86E-01
Q9H4L5	Oxysterol-binding protein-related protein 3	101.16	6.87	123.47982	19.616685	15	22	15	98.2	101.8	1.037	1.86E-02
Q9NQ50	39S ribosomal protein L40, mitochondrial	24.475	9.63	21.935157	15.048544	3	5	3	98.2	101.8	1.037	2.72E-01
Q9NQP4	Prefoldin subunit 4	15.305	4.53	67.620882	43.283582	7	11	7	98.2	101.8	1.037	3.88E-01
Q9NQT5	Exosome complex component RRP40	29.553	8.1	33.693863	17.090909	4	5	4	98.2	101.8	1.037	1.44E-02
Q9NRR5	Ubiquilin-4	63.812	5.22	74.326742	22.129784	10	14	7	98.2	101.8	1.037	2.07E-01
Q9NZB2	Constitutive coactivator of PPAR-gamma-like protein 1	121.811	8.88	107.02002	16.27907	15	18	15	98.2	101.8	1.037	2.20E-01
Q9P0J7	E3 ubiquitin-protein ligase KCMF1	41.919	5.66	18.082872	9.1863517	3	4	3	98.2	101.8	1.037	2.48E-01
Q9UBU9	Nuclear RNA export factor 1	70.139	8.51	55.141771	10.177706	5	8	5	98.2	101.8	1.037	2.74E-02
Q9UKG9	Peroxisomal carnitine O-octanoyltransferase	70.133	7.08	8.5062632	2.7777778	1	1	1	98.2	101.8	1.037	
Q9UMS4	Pre-mRNA-processing factor 19	55.146	6.61	91.007219	30.15873	12	23	12	98.2	101.8	1.037	3.75E-05
Q9Y2K7	Lysine-specific demethylase 2A	132.708	7.58	3.7363639	0.7745267	1	1	1	98.2	101.8	1.037	
Q9Y3B4	Splicing factor 3B subunit 6	14.576	9.38	52.397901	27.2	3	10	3	98.2	101.8	1.037	3.73E-02
Q9Y3C6	Peptidyl-prolyl cis-trans isomerase-like 1	18.225	7.99	32.452047	33.13253	5	7	5	98.2	101.8	1.037	6.11E-02
Q9Y3C7	Mediator of RNA polymerase II transcription subunit 31	15.795	8.54	4.3624102	6.870229	1	1	1	98.2	101.8	1.037	
O00154	Cytosolic acyl coenzyme A thioester hydrolase	41.769	8.54	87.977086	34.736842	10	19	10	98.1	101.9	1.039	3.52E-01
O15213	WD repeat-containing protein 46	68.029	9.67	36.257092	5.7377049	3	5	3	98.1	101.9	1.039	9.70E-01
O60518	Ran-binding protein 6	124.633	5.01	66.948981	9.7737557	9	13	7	98.1	101.9	1.039	1.13E-02
O95466	Formin-like protein 1	121.777	5.72	30.63627	6.2727273	6	6	5	98.1	101.9	1.039	3.45E-02
O95630	STAM-binding protein	48.047	6.29	25.747301	12.028302	4	4	4	98.1	101.9	1.039	
P04062	Glucosylceramidase	59.678	7.61	32.504262	11.567164	6	8	6	98.1	101.9	1.039	2.80E-02
P05556	Integrin beta-1	88.357	5.39	188.33546	26.190476	18	40	18	98.1	101.9	1.039	2.39E-04
P07437	Tubulin beta chain	49.639	4.89	292.6265	55.405405	17	190	3	98.1	101.9	1.039	4.96E-03
P11047	Laminin subunit gamma-1	177.489	5.12	89.027391	8.6389062	11	14	11	98.1	101.9	1.039	1.79E-01
P23229	Integrin alpha-6	126.526	6.61	26.279622	5.6637168	6	8	6	98.1	101.9	1.039	8.07E-01
P26583	High mobility group protein B2	24.019	7.81	17.184118	5.7416268	2	2	2	98.1	101.9	1.039	1.88E-01
P28702	Retinoic acid receptor RXR-beta	56.886	8.18	19.944654	5.6285178	3	4	2	98.1	101.9	1.039	
P29372	DNA-3-methyladenine glycosylase	32.848	9.57	39.284518	25.167785	6	9	6	98.1	101.9	1.039	2.21E-02
P32189	Glycerol kinase	61.205	6.54	12.773812	6.7978533	4	4	4	98.1	101.9	1.039	3.09E-01
P33240	Cleavage stimulation factor subunit 2	60.92	6.83	100.08844	25.823224	11	19	6	98.1	101.9	1.039	3.71E-01
P41223	Protein BUD31 homolog	16.989	8.82	40.077941	54.166667	7	16	7	98.1	101.9	1.039	2.94E-03
P49720	Proteasome subunit beta type-3	22.933	6.55	78.589937	28.780488	7	15	7	98.1	101.9	1.039	1.08E-01
P49790	Nuclear pore complex protein Nup153	153.843	8.73	82.469089	10.915254	12	15	12	98.1	101.9	1.039	7.81E-03
P55084	Trifunctional enzyme subunit beta, mitochondrial	51.262	9.41	88.961275	28.270042	13	24	13	98.1	101.9	1.039	6.99E-02
P61966	AP-1 complex subunit sigma-1A	18.721	5.73	4.7825161	5.6962025	1	2	1	98.1	101.9	1.039	
P62256	Ubiquitin-conjugating enzyme E2 H	20.642	4.67	29.220424	25.68306	4	6	4	98.1	101.9	1.039	1.81E-01
P62888	60S ribosomal protein L30	12.776	9.63	90.39067	54.782609	7	24	6	98.1	101.9	1.039	4.00E-01
P63272	Transcription elongation factor SPT4	13.185	8.06	11.446891	7.6923077	1	2	1	98.1	101.9	1.039	
Q01664	Transcription factor AP-4	38.702	5.87	21.720174	10.35503	3	4	3	98.1	101.9	1.039	
Q05D32	CTD small phosphatase-like protein 2	52.966	6.4	28.661498	10.085837	6	7	6	98.1	101.9	1.039	2.58E-01
Q07021	ɔmplement component 1 Q subcomponent-binding protein, mitochondr	31.343	4.84	100.8915	31.560284	6	22	6	98.1	101.9	1.039	1.65E-01
Q07666	domain-containing, RNA-binding, signal transduction-associated prote	48.197	8.66	55.255316	23.702032	8	11	7	98.1	101.9	1.039	9.11E-02
Q12774	Rho guanine nucleotide exchange factor 5	176.69	5.53	27.216408	4.6963056	5	6	5	98.1	101.9	1.039	2.35E-01

Q12965	Unconventional myosin-Ie	126.982	8.92	166.79951	20.66787	19	32	19	98.1	101.9	1.039	2.14E-04
Q13303	Voltage-gated potassium channel subunit beta-2	40.974	9	27.064591	14.713896	5	6	5	98.1	101.9	1.039	8.84E-01
Q13347	Eukaryotic translation initiation factor 3 subunit I	36.479	5.64	94.476311	28.615385	8	22	8	98.1	101.9	1.039	1.16E-02
Q13557	Calcium/calmodulin-dependent protein kinase type II subunit delta	56.334	7.25	69.839928	20.440882	8	12	5	98.1	101.9	1.039	2.39E-01
Q13614	Myotubularin-related protein 2	73.335	7.4	15.653246	6.9984448	5	5	5	98.1	101.9	1.039	2.55E-01
Q13838	Spliceosome RNA helicase DDX39B	48.96	5.67	111.80986	24.065421	10	25	5	98.1	101.9	1.039	7.06E-03
Q14677	Clathrin interactor 1	68.216	6.42	58.321779	15.36	9	11	9	98.1	101.9	1.039	1.33E-01
Q14694	Ubiquitin carboxyl-terminal hydrolase 10	87.08	5.31	74.374883	14.035088	9	15	9	98.1	101.9	1.039	4.08E-01
Q15056	Eukaryotic translation initiation factor 4H	27.368	7.23	43.208835	22.177419	4	10	4	98.1	101.9	1.039	1.13E-01
Q15773	Myeloid leukemia factor 2	28.129	6.9	22.336214	19.758065	4	5	4	98.1	101.9	1.039	1.11E-02
Q5VSL9	Striatin-interacting protein 1	95.515	6.29	19.317362	5.4958184	4	4	4	98.1	101.9	1.039	5.06E-01
Q6PCB5	Round spermatid basic protein 1-like protein	94.81	8.78	12.150645	3.7825059	4	4	4	98.1	101.9	1.039	
Q7Z6B7	SLIT-ROBO Rho GTPase-activating protein 1	124.186	6.83	31.788839	6.7281106	6	6	6	98.1	101.9	1.039	3.04E-02
Q86SQ0	Pleckstrin homology-like domain family B member 2	142.07	7.43	11.843198	2.2346369	3	3	2	98.1	101.9	1.039	4.69E-01
Q86U44	N6-adenosine-methyltransferase 70 kDa subunit	64.433	6.42	28.139663	7.0689655	3	4	3	98.1	101.9	1.039	1.02E-04
Q8IUI8	Cytokine receptor-like factor 3	49.735	5.14	19.951126	7.6923077	3	4	3	98.1	101.9	1.039	2.58E-01
Q8N9N2	Activating signal cointegrator 1 complex subunit 1	45.481	5.54	6.9538597	4	2	2	2	98.1	101.9	1.039	
Q8NBF6	Late secretory pathway protein AVL9 homolog	71.902	6.21	2.4713404	1.0802469	1	1	1	98.1	101.9	1.039	
Q8WYP5	Protein ELYS	252.342	6.6	130.21809	9.8411297	18	22	18	98.1	101.9	1.039	1.07E-01
Q92934	Bcl2-associated agonist of cell death	18.381	7.15	10.470944	14.880952	2	2	2	98.1	101.9	1.039	6.06E-01
Q96EA4	Protein Spindly	70.128	5.47	12.529407	4.7933884	3	4	3	98.1	101.9	1.039	1.17E-01
Q96EV2	RNA-binding protein 33	129.906	6.93	7.4843911	1.6239316	1	1	1	98.1	101.9	1.039	
Q96F86	Enhancer of mRNA-decapping protein 3	56.042	7.11	34.806369	12.401575	6	8	6	98.1	101.9	1.039	2.06E-02
Q99567	Nuclear pore complex protein Nup88	83.489	5.69	76.892612	17.408907	12	15	12	98.1	101.9	1.039	1.11E-02
Q99707	Methionine synthase	140.437	5.58	94.418583	12.01581	14	17	14	98.1	101.9	1.039	3.25E-01
Q9H147	Deoxynucleotidyltransferase terminal-interacting protein 1	36.99	8.97	13.056604	5.4711246	1	1	1	98.1	101.9	1.039	
Q9H4M9	EH domain-containing protein 1	60.589	6.83	178.80732	41.947566	21	34	19	98.1	101.9	1.039	1.26E-02
Q9H5V9	UPF0428 protein CXorf56	25.608	8.73	31.28174	17.567568	4	6	4	98.1	101.9	1.039	
Q9NNW5	WD repeat-containing protein 6	121.647	6.87	25.87002	3.1222123	3	4	3	98.1	101.9	1.039	7.15E-02
Q9NP58	ATP-binding cassette sub-family B member 6, mitochondrial	93.826	8.48	22.342111	4.631829	3	3	3	98.1	101.9	1.039	7.84E-01
Q9NS86	LanC-like protein 2	50.821	7.43	51.02374	14.222222	5	8	5	98.1	101.9	1.039	1.47E-01
Q9NTJ5	Phosphatidylinositide phosphatase SAC1	66.924	7.12	54.551711	10.732538	6	10	6	98.1	101.9	1.039	2.35E-02
Q9UBD5	Origin recognition complex subunit 3	82.201	7.61	14.785217	3.6568214	3	4	3	98.1	101.9	1.039	6.24E-01
Q9UEW8	STE20/SPS1-related proline-alanine-rich protein kinase	59.436	6.29	36.870606	15.229358	7	7	6	98.1	101.9	1.039	5.40E-05
Q9Y530	O-acetyl-ADP-ribose deacetylase 1	17.014	8.31	3.1560202	5.2631579	1	1	1	98.1	101.9	1.039	
Q9Y5M8	Signal recognition particle receptor subunit beta	29.684	9.04	90.628943	46.863469	12	18	12	98.1	101.9	1.039	7.91E-03
Q9Y6I3	Epsin-1	60.256	4.83	25.532623	7.8125	3	4	3	98.1	101.9	1.039	
O00203	AP-3 complex subunit beta-1	121.244	6.04	49.850248	8.4095064	10	11	10	98	102	1.041	5.26E-03
O00541	Pescadillo homolog	67.96	7.33	59.520001	17.346939	10	14	10	98	102	1.041	2.78E-02
O43402	ER membrane protein complex subunit 8	23.758	6.4	34.938772	25.714286	6	6	6	98	102	1.041	3.98E-01
O43837	Isocitrate dehydrogenase [NAD] subunit beta, mitochondrial	42.157	8.46	42.865856	17.142857	5	7	5	98	102	1.041	6.56E-01
O60513	Beta-1,4-galactosyltransferase 4	40.016	9.07	14.915614	8.1395349	2	2	2	98	102	1.041	
O75116	Rho-associated protein kinase 2	160.799	6.02	214.08664	24.135447	32	45	28	98	102	1.041	5.19E-02
O75528	Transcriptional adapter 3	48.872	6.27	7.5408295	5.0925926	2	2	2	98	102	1.041	
O94903	Proline synthase co-transcribed bacterial homolog protein	30.325	7.5	43.380962	26.181818	6	7	6	98	102	1.041	3.29E-04
P13284	Gamma-interferon-inducible lysosomal thiol reductase	27.945	4.88	26.499561	8	2	8	2	98	102	1.041	
P21980	Protein-glutamine gamma-glutamyltransferase 2	77.28	5.22	12.329834	4.657933	3	3	3	98	102	1.041	5.26E-01

P23588	Eukaryotic translation initiation factor 4B	69.11	5.73	75.08314	24.05892	15	21	15	98	102	1.041	2.16E-02
P35659	Protein DEK	42.648	8.56	93.913559	19.733333	8	20	8	98	102	1.041	8.75E-02
P40227	T-complex protein 1 subunit zeta	57.988	6.68	188.88741	43.691149	22	58	22	98	102	1.041	7.52E-03
P42285	Superkiller viralicidic activity 2-like 2	117.729	6.52	141.60591	19.193858	16	26	16	98	102	1.041	3.29E-03
P46977	:hyl-diphosphooligosaccharide--protein glycosyltransferase subunit ST	80.477	8.07	57.595569	12.340426	9	14	8	98	102	1.041	6.36E-03
P49916	DNA ligase 3	112.835	9.01	52.509998	10.307235	9	11	9	98	102	1.041	1.22E-01
P50416	Carnitine O-palmitoyltransferase 1, liver isoform	88.311	8.65	110.2261	26.002587	18	26	18	98	102	1.041	1.37E-03
P52907	F-actin-capping protein subunit alpha-1	32.902	5.69	131.14609	46.503497	8	25	7	98	102	1.041	1.09E-05
P53582	Methionine aminopeptidase 1	43.187	7.17	32.712912	9.3264249	3	8	3	98	102	1.041	1.63E-02
P53621	Coatomer subunit alpha	138.258	7.66	284.75349	34.477124	39	60	39	98	102	1.041	3.20E-05
P56211	cAMP-regulated phosphoprotein 19	12.315	9.09	8.3934037	23.214286	1	1	1	98	102	1.041	
P62266	40S ribosomal protein S23	15.798	10.49	55.134896	38.461538	8	22	8	98	102	1.041	1.14E-01
P62820	Ras-related protein Rab-1A	22.663	6.21	147.68239	67.804878	14	46	5	98	102	1.041	5.90E-03
P63000	Ras-related C3 botulinum toxin substrate 1	21.436	8.5	61.659779	27.604167	6	16	5	98	102	1.041	4.56E-02
P63173	60S ribosomal protein L38	8.213	10.1	9.9784398	24.285714	2	6	2	98	102	1.041	2.49E-01
Q00059	Transcription factor A, mitochondrial	29.078	9.72	32.281167	29.268293	8	9	8	98	102	1.041	2.47E-01
Q12800	Alpha-globin transcription factor CP2	57.22	5.8	22.526002	8.5657371	3	4	3	98	102	1.041	3.09E-01
Q15233	Non-POU domain-containing octamer-binding protein	54.197	8.95	322.28542	54.564756	26	123	21	98	102	1.041	1.28E-03
Q15438	Cytohesin-1	46.383	5.54	7.1028217	5.2763819	2	2	2	98	102	1.041	
Q3KQU3	MAP7 domain-containing protein 1	92.764	10.11	13.86121	2.9726516	2	2	2	98	102	1.041	2.37E-01
Q5SY16	Polynucleotide 5'-hydroxyl-kinase NOL9	79.272	9.13	16.051167	3.8461538	2	2	2	98	102	1.041	4.49E-01
Q5VVQ6	Ubiquitin thioesterase OTU1	38.298	6.11	4.9629721	2.5862069	1	1	1	98	102	1.041	
Q68CQ4	Digestive organ expansion factor homolog	87.001	5.88	23.088125	5.6878307	4	4	4	98	102	1.041	8.24E-02
Q7Z2Z1	Treslin	210.725	8.78	7.801893	0.6282723	1	1	1	98	102	1.041	
Q8IWA4	Mitofusin-1	84.047	6.25	30.597459	9.3117409	6	6	6	98	102	1.041	5.73E-02
Q8N4H5	Mitochondrial import receptor subunit TOM5 homolog	6.031	9.7	4.8283485	27.45098	2	3	2	98	102	1.041	4.50E-03
Q8WTS6	Histone-lysine N-methyltransferase SETD7	40.695	4.63	40.761469	16.666667	5	9	5	98	102	1.041	3.00E-01
Q8WVV9	Heterogeneous nuclear ribonucleoprotein L-like	60.045	7.72	78.322387	17.712177	8	13	7	98	102	1.041	5.62E-03
Q92896	Golgi apparatus protein 1	134.464	6.9	135.71332	16.793893	17	22	17	98	102	1.041	8.06E-03
Q92973	Transportin-1	102.289	4.98	145.163	19.153675	13	28	13	98	102	1.041	3.17E-04
Q92979	Ribosomal RNA small subunit methyltransferase NEP1	26.703	9.17	38.930866	25.819672	5	7	5	98	102	1.041	7.03E-01
Q96CT7	Coiled-coil domain-containing protein 124	25.82	9.54	33.763365	28.699552	6	8	6	98	102	1.041	1.54E-02
Q96EP5	DAZ-associated protein 1	43.356	8.56	26.604792	10.07371	3	7	3	98	102	1.041	7.46E-01
Q96IZ0	PRKC apoptosis WT1 regulator protein	36.545	5.41	24.125327	11.764706	3	4	3	98	102	1.041	6.99E-01
Q96KA5	Cleft lip and palate transmembrane protein 1-like protein	62.189	8.56	7.433315	3.3457249	2	2	2	98	102	1.041	
Q9BUP3	Oxidoreductase HTATIP2	27.032	8.38	70.437508	46.280992	11	18	11	98	102	1.041	2.05E-02
Q9BY42	Protein RTF2 homolog	33.865	8.59	46.124617	21.895425	6	9	6	98	102	1.041	1.72E-01
Q9GZS3	WD repeat-containing protein 61	33.56	5.47	27.678368	14.098361	3	4	3	98	102	1.041	3.02E-02
Q9GZZ1	N-alpha-acetyltransferase 50	19.386	8.81	62.47139	41.420118	7	15	7	98	102	1.041	1.49E-01
Q9H4A5	Golgi phosphoprotein 3-like	32.747	5.83	11.795217	5.2631579	1	2	1	98	102	1.041	
Q9H7Z7	Prostaglandin E synthase 2	41.917	9.16	17.11236	10.344828	4	7	4	98	102	1.041	1.74E-02
Q9HAV4	Exportin-5	136.222	5.8	92.334859	14.368771	14	22	14	98	102	1.041	3.26E-02
Q9NS69	Mitochondrial import receptor subunit TOM22 homolog	15.512	4.34	31.691925	16.197183	2	8	2	98	102	1.041	2.24E-01
Q9P270	SLAIN motif-containing protein 2	62.505	9.45	9.8456628	3.4423408	2	2	2	98	102	1.041	2.41E-01
Q9UBP0	Spastin	67.155	9.64	20.649322	6.1688312	3	4	3	98	102	1.041	2.33E-02
Q9UBV8	Peflin	30.361	6.54	13.923556	11.971831	4	5	4	98	102	1.041	8.09E-01
Q9UEE9	Craniofacial development protein 1	33.573	4.81	12.936838	8.361204	2	2	2	98	102	1.041	1.77E-02

Q9UHR5	SAP30-binding protein	33.85	4.84	17.262534	8.7662338	2	2	2	98	102	1.041	
Q9UK59	Lariat debranching enzyme	61.516	5.47	12.798462	5.5147059	3	3	3	98	102	1.041	2.43E-01
Q9UKK3	Poly [ADP-ribose] polymerase 4	192.472	5.66	13.832635	1.6241299	2	2	2	98	102	1.041	6.29E-02
Q9UKL0	REST corepressor 1	52.996	7.03	29.170163	10.580913	4	5	2	98	102	1.041	
Q9Y4E5	Zinc finger protein 451	121.406	6.77	7.1930742	1.5080113	1	1	1	98	102	1.041	
Q9Y5B8	Nucleoside diphosphate kinase 7	42.464	6.47	28.089043	18.617021	6	6	6	98	102	1.041	2.93E-01
Q9Y6A4	Cilia- and flagella-associated protein 20	22.76	9.76	19.295441	10.880829	2	3	2	98	102	1.041	1.27E-01
O00170	AH receptor-interacting protein	37.612	6.29	56.486043	24.848485	7	14	7	97.9	102.1	1.043	2.28E-02
O15118	Niemann-Pick C1 protein	142.074	5.36	16.468627	3.286385	3	3	3	97.9	102.1	1.043	3.42E-01
O15231	Zinc finger protein 185	73.48	7.01	40.55439	7.1117562	5	6	5	97.9	102.1	1.043	1.84E-02
O15347	High mobility group protein B3	22.965	8.37	64.238992	20.5	5	10	5	97.9	102.1	1.043	1.54E-03
O43169	Cytochrome b5 type B	16.322	4.97	11.242459	8.2191781	1	2	1	97.9	102.1	1.043	
O43709	Probable 18S rRNA (guanine-N(7))-methyltransferase	31.86	8.73	15.788095	7.8291815	1	2	1	97.9	102.1	1.043	
O43929	Origin recognition complex subunit 4	50.345	8	23.195573	7.3394495	3	4	3	97.9	102.1	1.043	4.50E-03
O75817	Ribonuclease P protein subunit p20	15.641	8.94	6.8005191	10.714286	1	1	1	97.9	102.1	1.043	
P37235	Hippocalcin-like protein 1	22.299	5.35	45.800841	29.015544	5	10	5	97.9	102.1	1.043	7.97E-01
P49643	DNA primase large subunit	58.769	7.91	39.164863	12.770138	6	9	6	97.9	102.1	1.043	1.68E-01
P52732	Kinesin-like protein KIF11	119.085	5.64	140.2359	16.950758	16	22	16	97.9	102.1	1.043	1.51E-04
P55209	Nucleosome assembly protein 1-like 1	45.346	4.46	91.252484	18.925831	6	23	5	97.9	102.1	1.043	2.82E-02
P61960	Ubiquitin-fold modifier 1	9.112	9.31	11.919983	17.647059	1	4	1	97.9	102.1	1.043	
P68400	Casein kinase II subunit alpha	45.115	7.74	44.768908	21.227621	7	9	6	97.9	102.1	1.043	9.03E-02
P78332	RNA-binding protein 6	128.565	6.32	20.47654	5.1647373	6	6	5	97.9	102.1	1.043	1.42E-01
Q14134	Tripartite motif-containing protein 29	65.793	7.15	67.31993	22.44898	13	17	13	97.9	102.1	1.043	1.30E-02
Q15057	f-GAP with coiled-coil, ANK repeat and PH domain-containing protei	87.973	6.8	14.909976	2.6992288	2	2	2	97.9	102.1	1.043	
Q15124	Phosphoglucomutase-like protein 5	62.186	7.21	14.629806	5.8201058	3	3	3	97.9	102.1	1.043	1.94E-02
Q15750	TGF-beta-activated kinase 1 and MAP3K7-binding protein 1	54.61	5.52	34.818999	6.7460317	3	5	3	97.9	102.1	1.043	3.97E-02
Q2TBE0	CWF19-like protein 2	103.722	8.65	6.3357604	1.7897092	2	2	2	97.9	102.1	1.043	
Q6IBW4	Condensin-2 complex subunit H2	68.184	4.74	18.827357	8.7603306	5	5	5	97.9	102.1	1.043	1.32E-02
Q6NZI2	Polymerase I and transcript release factor	43.45	5.6	104.1145	19.487179	7	20	7	97.9	102.1	1.043	4.49E-02
Q6ZXV5	Transmembrane and TPR repeat-containing protein 3	103.942	8.87	18.938719	4.5901639	5	7	4	97.9	102.1	1.043	
Q71RC2	La-related protein 4	80.546	6.61	23.851138	5.9392265	5	6	5	97.9	102.1	1.043	1.05E-01
Q92783	Signal transducing adapter molecule 1	59.142	4.82	24.04635	6.2962963	3	4	3	97.9	102.1	1.043	6.06E-01
Q92793	CREB-binding protein	265.182	8.53	13.415558	1.4742015	3	3	3	97.9	102.1	1.043	
Q92797	Symplekin	141.059	6.13	82.227995	13.265306	11	14	11	97.9	102.1	1.043	1.13E-02
Q96EL3	39S ribosomal protein L53, mitochondrial	12.099	8.76	36.665468	41.071429	4	6	4	97.9	102.1	1.043	9.86E-03
Q96EY1	DnaJ homolog subfamily A member 3, mitochondrial	52.456	9.26	45.050749	13.125	5	8	5	97.9	102.1	1.043	2.91E-01
Q96ST3	Paired amphipathic helix protein Sin3a	145.085	7.25	10.41903	2.8279654	3	3	3	97.9	102.1	1.043	1.91E-02
Q9BTW9	Tubulin-specific chaperone D	132.515	6.19	69.854157	9.8154362	11	12	11	97.9	102.1	1.043	1.72E-03
Q9BXJ9	N-alpha-acetyltransferase 15, NatA auxiliary subunit	101.208	7.42	129.98666	21.47806	19	33	19	97.9	102.1	1.043	2.56E-02
Q9H0N0	Ras-related protein Rab-6C	28.337	7.71	10.224244	8.6614173	1	1	1	97.9	102.1	1.043	
Q9H814	Phosphorylated adapter RNA export protein	44.375	5.4	7.3208098	2.7918782	1	2	1	97.9	102.1	1.043	
Q9NPJ6	Mediator of RNA polymerase II transcription subunit 4	29.727	5.1	15.244709	8.8888889	2	2	2	97.9	102.1	1.043	5.85E-01
Q9NX20	39S ribosomal protein L16, mitochondrial	28.431	10.13	12.26488	6.374502	1	1	1	97.9	102.1	1.043	
Q9NY61	Protein AATF	63.094	4.94	14.076719	6.25	3	3	3	97.9	102.1	1.043	
Q9NZW5	MAGUK p55 subfamily member 6	61.079	6.18	41.700912	13.148148	7	8	6	97.9	102.1	1.043	6.70E-02
Q9P2B4	CTTNBP2 N-terminal-like protein	70.115	8.06	62.506861	17.99687	10	11	10	97.9	102.1	1.043	3.69E-02
Q9UBB9	Tuftelin-interacting protein 11	96.758	5.67	44.602896	11.708483	9	9	9	97.9	102.1	1.043	2.62E-01

Q9Y282	Endoplasmic reticulum-Golgi intermediate compartment protein 3	43.194	6.06	24.548154	7.5718016	3	5	3	97.9	102.1	1.043	7.34E-02
Q9Y2L1	Exosome complex exonuclease RRP44	108.934	7.14	97.051734	13.569937	10	15	10	97.9	102.1	1.043	9.52E-03
Q9Y2Z2	Protein MTO1 homolog, mitochondrial	79.914	8.31	5.2804201	1.2552301	1	1	1	97.9	102.1	1.043	
Q9Y4E8	Ubiquitin carboxyl-terminal hydrolase 15	112.348	5.22	79.859157	14.067278	12	15	11	97.9	102.1	1.043	1.56E-02
Q9Y618	Nuclear receptor corepressor 2	274.635	7.59	45.888717	4.1980198	9	9	7	97.9	102.1	1.043	1.31E-01
Q9Y6D9	Mitotic spindle assembly checkpoint protein MAD1	83.016	5.92	64.938417	14.345404	9	11	9	97.9	102.1	1.043	8.31E-03
Q9Y6M9	NADH dehydrogenase [ubiquinone] 1 beta subcomplex subunit 9	21.817	8.38	17.990742	24.581006	4	4	4	97.9	102.1	1.043	4.81E-01
O14579	Coatomer subunit epsilon	34.46	5.12	46.872706	28.246753	8	11	8	97.8	102.2	1.045	2.47E-01
O14715	RANBP2-like and GRIP domain-containing protein 8	198.869	6.49	141.22814	12.23796	22	32	3	97.8	102.2	1.045	1.08E-04
O75694	Nuclear pore complex protein Nup155	155.1	6.16	223.90523	21.495327	24	39	24	97.8	102.2	1.045	6.69E-03
P10109	Adrenodoxin, mitochondrial	19.381	5.83	6.2661204	8.6956522	2	2	2	97.8	102.2	1.045	
P13987	CD59 glycoprotein	14.168	6.48	32.101321	25	4	8	4	97.8	102.2	1.045	4.13E-01
P35658	Nuclear pore complex protein Nup214	213.488	7.47	189.94713	13.588517	23	35	22	97.8	102.2	1.045	2.13E-05
P49589	Cysteine--tRNA ligase, cytoplasmic	85.419	6.76	97.058258	22.326203	15	21	15	97.8	102.2	1.045	1.24E-01
P54132	Bloom syndrome protein	158.901	7.49	5.0535782	1.1291461	2	2	1	97.8	102.2	1.045	
Q00839	Heterogeneous nuclear ribonucleoprotein U	90.528	6	278.01667	29.212121	25	77	24	97.8	102.2	1.045	9.51E-03
Q01433	AMP deaminase 2	100.624	6.93	37.249631	9.3287827	7	9	7	97.8	102.2	1.045	1.11E-02
Q15061	WD repeat-containing protein 43	74.843	5.57	33.086448	9.7488922	7	8	7	97.8	102.2	1.045	3.77E-02
Q5T2T1	MAGUK p55 subfamily member 7	65.483	7.14	8.0748619	3.4722222	2	2	2	97.8	102.2	1.045	3.22E-01
Q7Z7F0	UPF0469 protein KIAA0907	64.805	8.73	13.044205	2.1172638	2	2	2	97.8	102.2	1.045	8.56E-02
Q86SE9	Polycomb group RING finger protein 5	29.694	6.52	3.5234582	4.296875	1	1	1	97.8	102.2	1.045	
Q8IWB9	Testis-expressed sequence 2 protein	125.224	6.01	4.5795491	0.7985803	1	1	1	97.8	102.2	1.045	
Q8N543	Prolyl 3-hydroxylase OGFOD1	63.206	5.11	31.458579	9.7785978	6	6	6	97.8	102.2	1.045	2.22E-01
Q8NCF5	NFATC2-interacting protein	45.789	6.6	7.7408842	4.0572792	1	1	1	97.8	102.2	1.045	
Q8TC07	TBC1 domain family member 15	79.44	5.67	37.526042	10.419682	7	8	7	97.8	102.2	1.045	3.41E-01
Q8WUK0	Phosphatidylglycerophosphatase and protein-tyrosine phosphatase 1	22.829	9.77	17.832394	20.895522	4	5	4	97.8	102.2	1.045	9.63E-02
Q92769	Histone deacetylase 2	55.329	5.91	37.566791	14.139344	6	8	5	97.8	102.2	1.045	2.19E-01
Q96GM8	Target of EGR1 protein 1	56.512	7.18	14.729971	7.4509804	3	3	3	97.8	102.2	1.045	2.42E-02
Q9BQ39	ATP-dependent RNA helicase DDX50	82.514	9.17	87.214231	17.367707	10	14	8	97.8	102.2	1.045	1.21E-01
Q9BSE5	Agmatinase, mitochondrial	37.636	7.59	11.768217	11.363636	2	2	2	97.8	102.2	1.045	8.96E-02
Q9H8V3	Protein ECT2	103.439	7.71	12.216605	2.8446389	2	2	2	97.8	102.2	1.045	
Q9H8W4	Pleckstrin homology domain-containing family F member 2	27.78	8.21	17.543695	7.6305221	2	3	2	97.8	102.2	1.045	4.28E-02
Q9HCC0	Methylcrotonoyl-CoA carboxylase beta chain, mitochondrial	61.294	7.68	73.419068	17.40675	8	12	8	97.8	102.2	1.045	8.80E-01
Q9NPD8	Ubiquitin-conjugating enzyme E2 T	22.507	7.99	21.927433	31.979695	5	5	5	97.8	102.2	1.045	3.77E-01
Q9NTI5	Sister chromatid cohesion protein PDS5 homolog B	164.563	8.47	74.311501	10.573601	16	18	14	97.8	102.2	1.045	1.48E-01
Q9NZN8	CCR4-NOT transcription complex subunit 2	59.7	7.66	9.2764622	2.962963	1	1	1	97.8	102.2	1.045	
Q9UBT2	SUMO-activating enzyme subunit 2	71.179	5.29	84.042349	23.125	10	14	10	97.8	102.2	1.045	1.58E-02
Q9ULR3	Protein phosphatase 1H	56.413	6.6	9.175885	4.8638132	2	2	2	97.8	102.2	1.045	8.62E-02
Q9Y4H2	Insulin receptor substrate 2	137.249	8.65	48.842806	6.2780269	5	6	5	97.8	102.2	1.045	7.69E-02
Q9Y508	E3 ubiquitin-protein ligase RNF114	25.677	7.25	65.889776	39.473684	7	10	7	97.8	102.2	1.045	1.72E-01
Q9Y5S9	RNA-binding protein 8A	19.877	5.72	9.260699	20.114943	3	4	3	97.8	102.2	1.045	4.45E-01
Q9Y6I9	Testis-expressed sequence 264 protein	34.167	4.86	12.133094	4.4728435	1	2	1	97.8	102.2	1.045	
O95758	Polypyrimidine tract-binding protein 3	59.652	9.04	46.75885	10.326087	6	14	3	97.7	102.3	1.047	1.48E-01
P06865	Beta-hexosaminidase subunit alpha	60.664	5.16	29.008894	11.342155	5	6	5	97.7	102.3	1.047	4.38E-02
P09496	Clathrin light chain A	27.06	4.51	17.643046	15.322581	5	8	5	97.7	102.3	1.047	4.68E-01
P13861	cAMP-dependent protein kinase type II-alpha regulatory subunit	45.49	5.07	108.88834	30.693069	11	24	11	97.7	102.3	1.047	4.79E-02
P15880	40S ribosomal protein S2	31.305	10.24	132.77489	45.733788	14	42	14	97.7	102.3	1.047	5.95E-04

P16104	Histone H2AX	15.135	10.74	37.548648	32.867133	7	22	4	97.7	102.3	1.047	7.45E-01
P20585	DNA mismatch repair protein Msh3	127.332	8.02	31.864908	6.9481091	7	7	7	97.7	102.3	1.047	7.19E-01
P30419	Glycylpeptide N-tetradecanoyltransferase 1	56.77	7.8	87.350693	20.362903	9	20	9	97.7	102.3	1.047	1.93E-02
P35754	Glutaredoxin-1	11.768	8.09	38.077221	30.188679	2	4	2	97.7	102.3	1.047	3.97E-01
P46109	Crk-like protein	33.756	6.74	57.303293	31.683168	6	9	6	97.7	102.3	1.047	7.53E-02
P49406	39S ribosomal protein L19, mitochondrial	33.514	9.5	31.786998	13.356164	4	6	4	97.7	102.3	1.047	1.46E-01
P56537	Eukaryotic translation initiation factor 6	26.582	4.68	120.61266	35.918367	6	24	6	97.7	102.3	1.047	1.76E-04
P62633	Cellular nucleic acid-binding protein	19.45	7.71	74.638659	38.418079	7	16	7	97.7	102.3	1.047	7.05E-03
P63151	threonine-protein phosphatase 2A 55 kDa regulatory subunit B alpha i	51.659	6.2	49.004696	15.659955	7	11	7	97.7	102.3	1.047	1.45E-01
P82933	28S ribosomal protein S9, mitochondrial	45.806	9.51	76.898046	27.020202	9	10	9	97.7	102.3	1.047	2.85E-02
Q03135	Caveolin-1	20.458	6.02	26.726472	19.662921	5	11	5	97.7	102.3	1.047	3.35E-02
Q03701	CCAAT/enhancer-binding protein zeta	120.898	5.94	76.867005	11.195446	11	14	11	97.7	102.3	1.047	4.40E-05
Q05048	Cleavage stimulation factor subunit 1	48.327	6.58	69.225096	26.914153	9	10	9	97.7	102.3	1.047	3.72E-02
Q12788	Transducin beta-like protein 3	88.978	6.9	89.901427	17.69802	10	13	10	97.7	102.3	1.047	1.79E-01
Q15427	Splicing factor 3B subunit 4	44.357	8.56	12.218924	4.7169811	2	2	2	97.7	102.3	1.047	2.09E-01
Q15434	RNA-binding motif, single-stranded-interacting protein 2	43.931	9.07	12.299348	9.3366093	3	4	2	97.7	102.3	1.047	
Q16537	'threonine-protein phosphatase 2A 56 kDa regulatory subunit epsilon i:	54.664	6.95	38.44296	10.06424	4	6	4	97.7	102.3	1.047	1.19E-01
Q5JVF3	PCI domain-containing protein 2	46	8.53	64.644564	25.062657	9	12	9	97.7	102.3	1.047	1.00E-02
Q5VW36	Focadhesin	199.944	6.62	5.7952513	1.4436424	3	3	3	97.7	102.3	1.047	3.55E-01
Q6P1Q0	LETM1 domain-containing protein 1	41.763	10.32	9.0396244	3.3333333	1	1	1	97.7	102.3	1.047	
Q7Z417	Nuclear fragile X mental retardation-interacting protein 2	76.075	8.7	70.084926	12.94964	8	15	8	97.7	102.3	1.047	1.31E-03
Q7Z4V5	Hepatoma-derived growth factor-related protein 2	74.272	7.49	35.5624	9.6870343	6	11	5	97.7	102.3	1.047	8.76E-02
Q8IWA5	Choline transporter-like protein 2	80.071	8.57	9.3588264	1.6997167	1	2	1	97.7	102.3	1.047	
Q8IZ69	tRNA (uracil-5-)-methyltransferase homolog A	68.682	7.94	17.539695	8	5	5	5	97.7	102.3	1.047	4.34E-02
Q8TED0	U3 small nucleolar RNA-associated protein 15 homolog	58.379	9.11	22.253351	5.7915058	4	7	4	97.7	102.3	1.047	1.73E-01
Q8TEX9	Importin-4	118.64	4.96	141.70442	19.426457	16	25	16	97.7	102.3	1.047	3.08E-01
Q8WVM7	Cohesin subunit SA-1	144.336	5.59	27.680888	5.3259141	7	8	3	97.7	102.3	1.047	9.44E-04
Q969E8	Pre-rRNA-processing protein TSR2 homolog	20.881	4.39	3.5994905	8.3769634	2	2	2	97.7	102.3	1.047	
Q969H8	Myeloid-derived growth factor	18.783	6.68	39.308691	20.809249	4	7	4	97.7	102.3	1.047	4.40E-02
Q96R06	Sperm-associated antigen 5	134.338	5	13.80535	1.7602682	2	3	2	97.7	102.3	1.047	3.97E-01
Q96SQ9	Cytochrome P450 2S1	55.781	8.62	26.857562	10.119048	4	4	4	97.7	102.3	1.047	4.85E-02
Q9BSR8	Protein YIPF4	27.065	4.65	3.7380238	3.6885246	1	1	1	97.7	102.3	1.047	
Q9BSY4	Coiled-coil-helix-coiled-coil-helix domain-containing protein 5	12.387	6.73	58.960545	65.454545	5	8	5	97.7	102.3	1.047	5.91E-01
Q9BVS4	Serine/threonine-protein kinase RIO2	63.243	5.94	18.57587	6.3405797	3	3	3	97.7	102.3	1.047	3.76E-03
Q9BXF6	Rab11 family-interacting protein 5	70.372	9.23	16.760323	7.5038285	3	3	3	97.7	102.3	1.047	5.50E-02
Q9BY44	Eukaryotic translation initiation factor 2A	64.949	8.87	159.52935	32.649573	16	32	16	97.7	102.3	1.047	6.99E-01
Q9H223	EH domain-containing protein 4	61.137	6.76	41.272364	14.048059	7	8	6	97.7	102.3	1.047	1.16E-02
Q9H5K3	Protein O-mannose kinase	40.024	6.1	6.0164319	4.2857143	2	2	2	97.7	102.3	1.047	
Q9H977	WD repeat-containing protein 54	35.868	6.2	7.0655015	3.8922156	1	1	1	97.7	102.3	1.047	
Q9HCE1	Putative helicase MOV-10	113.599	8.82	54.592825	10.667996	9	10	9	97.7	102.3	1.047	3.35E-01
Q9NR56	Muscleblind-like protein 1	41.79	8.9	9.949597	2.3195876	1	2	1	97.7	102.3	1.047	
Q9NW13	RNA-binding protein 28	85.685	9.22	101.3388	15.41502	10	16	10	97.7	102.3	1.047	1.32E-02
Q9UH62	Armadillo repeat-containing X-linked protein 3	42.474	8.37	34.021872	9.762533	3	6	3	97.7	102.3	1.047	7.78E-02
Q9UHD1	Cysteine and histidine-rich domain-containing protein 1	37.466	7.87	143.13226	40.361446	13	29	13	97.7	102.3	1.047	7.31E-02
Q9UNS2	COP9 signalosome complex subunit 3	47.842	6.65	36.271315	9.2198582	4	10	4	97.7	102.3	1.047	5.95E-02
Q9Y2S7	Polymerase delta-interacting protein 2	42.007	8.63	43.388528	13.043478	4	7	4	97.7	102.3	1.047	1.82E-01
Q9Y3C1	Nucleolar protein 16	21.175	9.94	20.461938	22.47191	4	5	4	97.7	102.3	1.047	

Q9Y5J7	Mitochondrial import inner membrane translocase subunit Tim9	10.371	7.21	23.137287	47.191011	4	4	4	97.7	102.3	1.047	6.67E-02
O00488	Zinc finger protein 593	15.19	9.82	11.945843	26.119403	3	3	3	97.6	102.4	1.049	
O15320	cTAGE family member 5	90.94	5.22	36.733672	7.0895522	5	6	5	97.6	102.4	1.049	1.22E-02
O75131	Copine-3	60.092	5.85	65.451888	20.856611	11	13	10	97.6	102.4	1.049	3.30E-01
O75569	feron-inducible double-stranded RNA-dependent protein kinase activa	34.383	8.41	17.675529	4.7923323	1	2	1	97.6	102.4	1.049	
P08581	Hepatocyte growth factor receptor	155.441	7.33	32.928604	4.4604317	5	6	4	97.6	102.4	1.049	3.50E-02
P11532	Dystrophin	426.484	5.88	34.2977	1.4111262	5	7	4	97.6	102.4	1.049	1.72E-01
P15559	NAD(P)H dehydrogenase [quinone] 1	30.848	8.88	46.862473	22.627737	7	14	7	97.6	102.4	1.049	5.99E-02
P23193	Transcription elongation factor A protein 1	33.948	8.38	85.060991	24.916944	8	14	8	97.6	102.4	1.049	1.14E-01
P23497	Nuclear autoantigen Sp-100	100.354	8.22	16.239492	5.1194539	5	7	4	97.6	102.4	1.049	8.88E-02
P43034	Platelet-activating factor acetylhydrolase IB subunit alpha	46.608	7.37	86.526372	28.780488	11	17	11	97.6	102.4	1.049	2.45E-03
P51965	Ubiquitin-conjugating enzyme E2 E1	21.391	8.53	28.079183	22.797927	4	5	4	97.6	102.4	1.049	1.37E-02
P60903	Protein S100-A10	11.196	7.37	41.102436	27.835052	3	10	3	97.6	102.4	1.049	4.78E-02
P62263	40S ribosomal protein S14	16.263	10.05	136.8552	39.735099	8	31	8	97.6	102.4	1.049	8.04E-02
Q04837	Single-stranded DNA-binding protein, mitochondrial	17.249	9.6	96.949961	53.378378	7	21	7	97.6	102.4	1.049	3.06E-02
Q14181	DNA polymerase alpha subunit B	65.906	5.24	29.505507	11.036789	6	6	6	97.6	102.4	1.049	5.19E-02
Q14669	E3 ubiquitin-protein ligase TRIP12	220.296	8.48	92.663393	7.1285141	12	17	12	97.6	102.4	1.049	2.74E-05
Q5T1C6	Acyl-coenzyme A thioesterase THEM4	27.112	8.28	8.0663103	6.25	1	1	1	97.6	102.4	1.049	
Q66K14	TBC1 domain family member 9B	140.436	5.25	17.923294	3.04	3	3	3	97.6	102.4	1.049	
Q6NYC8	Phostensin	67.902	5.4	19.935064	9.1353997	4	4	4	97.6	102.4	1.049	4.12E-02
Q7Z569	BRCA1-associated protein	67.261	5.92	12.532351	3.8851351	2	2	2	97.6	102.4	1.049	
Q8IZP0	Abl interactor 1	55.047	7.06	15.797238	7.0866142	3	3	3	97.6	102.4	1.049	5.14E-01
Q8N511	Transmembrane protein 199	23.116	9.01	4.1260984	3.8461538	1	1	1	97.6	102.4	1.049	
Q8N5I9	Uncharacterized protein C12orf45	20.111	5.22	7.937418	6.4864865	1	1	1	97.6	102.4	1.049	
Q8TBC4	NEDD8-activating enzyme E1 catalytic subunit	51.819	5.45	44.40085	26.349892	8	9	8	97.6	102.4	1.049	1.04E-02
Q8WWH5	Probable tRNA pseudouridine synthase 1	37.229	8.25	22.228376	13.180516	4	4	4	97.6	102.4	1.049	1.01E-02
Q8WXA9	Splicing regulatory glutamine/lysine-rich protein 1	59.345	10.39	23.519836	10.629921	4	4	4	97.6	102.4	1.049	6.12E-03
Q969X5	Endoplasmic reticulum-Golgi intermediate compartment protein 1	32.571	7.06	39.006421	16.551724	5	8	5	97.6	102.4	1.049	9.48E-01
Q96B45	UPF0693 protein C10orf32	11.557	6.79	4.7527635	12.380952	1	1	1	97.6	102.4	1.049	
Q96GX5	Serine/threonine-protein kinase greatwall	97.257	5.99	28.535823	4.6643914	4	7	3	97.6	102.4	1.049	9.01E-01
Q96KB5	Lymphokine-activated killer T-cell-originated protein kinase	36.062	5.12	41.505211	18.63354	7	11	7	97.6	102.4	1.049	5.13E-05
Q9H267	Vacuolar protein sorting-associated protein 33B	70.54	6.71	40.686757	12.80389	8	9	8	97.6	102.4	1.049	7.95E-03
Q9H6T3	RNA polymerase II-associated protein 3	75.672	6.84	122.21882	25.413534	15	21	15	97.6	102.4	1.049	8.60E-04
Q9H788	SH2 domain-containing protein 4A	52.694	8.06	16.036445	7.7092511	3	4	3	97.6	102.4	1.049	4.13E-02
Q9NWH9	SAFB-like transcription modulator	117.079	7.87	31.168295	6.1895551	6	7	6	97.6	102.4	1.049	1.33E-01
Q9P258	Protein RCC2	56.049	8.78	152.57994	34.099617	17	28	17	97.6	102.4	1.049	2.76E-01
Q9P2K3	REST corepressor 3	55.547	8.27	32.565834	8.4848485	4	5	2	97.6	102.4	1.049	
Q9UJX2	Cell division cycle protein 23 homolog	68.79	7.02	4.4353339	1.3400335	1	1	1	97.6	102.4	1.049	
Q9UNN5	FAS-associated factor 1	73.908	4.88	88.253785	14	10	15	10	97.6	102.4	1.049	9.58E-02
Q9Y262	Eukaryotic translation initiation factor 3 subunit L	66.684	6.34	125.86117	32.092199	16	24	16	97.6	102.4	1.049	3.70E-02
Q9Y2U5	Mitogen-activated protein kinase kinase kinase 2	69.697	8	9.8942651	3.3925687	2	2	2	97.6	102.4	1.049	
Q9Y2Z0	Protein SGT1 homolog	40.998	5.16	89.956028	41.917808	12	20	12	97.6	102.4	1.049	2.58E-04
Q9Y3I0	tRNA-splicing ligase RtcB homolog	55.175	7.23	137.39013	32.277228	14	29	14	97.6	102.4	1.049	1.87E-01
Q9Y639	Neuroplastin	44.36	7.99	7.652599	5.2763819	2	2	2	97.6	102.4	1.049	
O00499	Myc box-dependent-interacting protein 1	64.659	5.06	66.594066	22.765599	11	16	11	97.5	102.5	1.051	1.41E-02
O75531	Barrier-to-autointegration factor	10.052	6.09	42.525607	42.696629	3	12	3	97.5	102.5	1.051	1.54E-01
O75787	Renin receptor	38.983	6.1	10.803426	7.7142857	3	4	3	97.5	102.5	1.051	1.09E-02

O95602	DNA-directed RNA polymerase I subunit RPA1	194.687	7.03	37.379145	5.5813953	9	11	9	97.5	102.5	1.051	3.10E-04
P17535	Transcription factor jun-D	35.152	7.37	32.249466	14.985591	3	4	3	97.5	102.5	1.051	2.12E-01
P35269	General transcription factor IIF subunit 1	58.205	7.49	38.518126	13.346228	6	9	6	97.5	102.5	1.051	7.64E-03
P49023	Paxillin	64.464	6.19	69.374239	15.228426	7	14	7	97.5	102.5	1.051	3.61E-01
P56270	Myc-associated zinc finger protein	48.576	8.95	29.30429	7.3375262	3	6	3	97.5	102.5	1.051	8.27E-01
P98082	Disabled homolog 2	82.397	5.53	174.12156	30.38961	18	36	18	97.5	102.5	1.051	2.95E-02
Q08499	cAMP-specific 3',5'-cyclic phosphodiesterase 4D	91.058	5.54	26.368409	6.1804697	5	5	5	97.5	102.5	1.051	2.51E-01
Q12846	Syntaxin-4	34.159	6.28	21.280661	9.7643098	3	5	3	97.5	102.5	1.051	3.58E-02
Q13188	Serine/threonine-protein kinase 3	56.265	5.24	71.893224	18.533605	8	12	5	97.5	102.5	1.051	1.33E-04
Q13395	Probable methyltransferase TARBP1	181.559	7.05	56.2699	5.4287477	7	9	7	97.5	102.5	1.051	1.35E-04
Q14247	Src substrate cortactin	61.549	5.4	186.73254	36.545455	19	38	19	97.5	102.5	1.051	1.21E-02
Q14966	Zinc finger protein 638	220.488	6.38	50.122578	6.471183	12	12	12	97.5	102.5	1.051	2.25E-02
Q15361	Transcription termination factor 1	102.987	9.38	16.439869	2.5414365	2	3	2	97.5	102.5	1.051	1.52E-02
Q15633	RISC-loading complex subunit TARBP2	39.015	6.54	7.2167928	7.1038251	2	2	2	97.5	102.5	1.051	
Q16270	Insulin-like growth factor-binding protein 7	29.111	7.9	33.219485	18.439716	4	5	4	97.5	102.5	1.051	2.91E-02
Q7Z7K0	COX assembly mitochondrial protein homolog	12.481	8.63	12.774956	25.471698	2	2	2	97.5	102.5	1.051	
Q86VN1	Vacuolar protein-sorting-associated protein 36	43.789	7.2	6.7428196	4.4041451	2	2	1	97.5	102.5	1.051	
Q8IVL5	Prolyl 3-hydroxylase 2	80.933	5.71	52.412343	9.6045198	5	7	5	97.5	102.5	1.051	1.50E-01
Q8NB46	rine/threonine-protein phosphatase 6 regulatory ankyrin repeat subunit	115.004	6.48	14.614036	2.1375465	1	1	1	97.5	102.5	1.051	
Q8TB36	Ganglioside-induced differentiation-associated protein 1	41.32	8.34	6.6145725	4.1899441	1	1	1	97.5	102.5	1.051	
Q8WU76	Sec1 family domain-containing protein 2	75.079	6.68	12.060371	3.6549708	2	2	2	97.5	102.5	1.051	2.68E-01
Q92540	Protein SMG7	127.202	8.72	7.9641702	1.2313105	1	1	1	97.5	102.5	1.051	
Q96DF8	Protein DGCR14	52.536	7.56	8.8511631	5.8823529	2	3	2	97.5	102.5	1.051	
Q96KR1	Zinc finger RNA-binding protein	116.939	9.04	113.35706	19.273743	16	22	16	97.5	102.5	1.051	7.60E-02
Q96PU8	Protein quaking	37.647	8.56	17.560448	7.9178886	2	3	2	97.5	102.5	1.051	
Q96Q15	Serine/threonine-protein kinase SMG1	410.241	6.46	23.807379	0.928708	3	4	3	97.5	102.5	1.051	
Q96S59	Ran-binding protein 9	77.798	6.79	14.955587	3.0178326	2	3	2	97.5	102.5	1.051	2.16E-01
Q9BU76	Multiple myeloma tumor-associated protein 2	29.394	10.02	21.149213	14.828897	3	3	3	97.5	102.5	1.051	1.40E-01
Q9H0B6	Kinesin light chain 2	68.892	7.15	86.36485	23.633441	13	18	9	97.5	102.5	1.051	3.21E-02
Q9NT62	Ubiquitin-like-conjugating enzyme ATG3	35.841	4.74	44.411366	16.878981	5	10	5	97.5	102.5	1.051	5.77E-02
Q9NVE7	Pantothenate kinase 4	85.937	6.28	4.7371338	2.0698577	2	2	2	97.5	102.5	1.051	
Q9NW68	BSD domain-containing protein 1	47.134	4.49	12.744996	7.4418605	3	3	3	97.5	102.5	1.051	4.89E-02
Q9NZI8	Insulin-like growth factor 2 mRNA-binding protein 1	63.441	9.2	98.252884	21.837088	12	21	10	97.5	102.5	1.051	4.06E-01
Q9UBB5	Methyl-CpG-binding domain protein 2	43.228	10.04	21.871037	9.4890511	3	3	3	97.5	102.5	1.051	6.41E-01
Q9Y312	Protein AAR2 homolog	43.444	5.96	31.784857	11.458333	3	6	3	97.5	102.5	1.051	3.90E-03
Q9Y3D8	Adenylate kinase isoenzyme 6	20.049	4.58	8.1736949	14.534884	2	2	2	97.5	102.5	1.051	
Q9Y446	Plakophilin-3	87.029	9.32	85.966396	16.436637	12	16	12	97.5	102.5	1.051	3.54E-04
Q9Y4R8	Telomere length regulation protein TEL2 homolog	91.689	5.76	37.258004	10.991637	9	9	9	97.5	102.5	1.051	1.77E-01
Q9Y5J1	U3 small nucleolar RNA-associated protein 18 homolog	61.964	8.76	43.425129	10.971223	4	7	4	97.5	102.5	1.051	1.67E-01
Q9Y657	Spindlin-1	29.582	6.96	10.713802	8.778626	2	2	2	97.5	102.5	1.051	2.23E-01
Q9Y6Y0	Influenza virus NS1A-binding protein	71.683	5.53	11.389888	4.6728972	3	3	3	97.5	102.5	1.051	4.28E-01
O14936	Peripheral plasma membrane protein CASK	105.056	6.43	22.424727	5.8315335	6	6	6	97.4	102.6	1.053	1.14E-01
O15234	Protein CASC3	76.232	6.48	18.706896	4.6941679	4	5	4	97.4	102.6	1.053	2.45E-02
O15372	Eukaryotic translation initiation factor 3 subunit H	39.905	6.54	72.825777	20.170455	7	19	7	97.4	102.6	1.053	1.06E-05
O43379	WD repeat-containing protein 62	165.849	5.91	10.724846	1.3833992	2	2	2	97.4	102.6	1.053	4.52E-02
O43795	Unconventional myosin-Ib	131.902	9.38	127.95805	13.996479	14	23	14	97.4	102.6	1.053	4.28E-05
O75150	E3 ubiquitin-protein ligase BRE1B	113.581	6.23	133.27971	20.779221	16	22	14	97.4	102.6	1.053	2.66E-03

O75592	E3 ubiquitin-protein ligase MYCBP2	509.759	7.03	18.818636	1.012931	4	4	4	97.4	102.6	1.053	3.07E-01
O75880	Protein SCO1 homolog, mitochondrial	33.793	8.88	12.07971	7.9734219	2	2	2	97.4	102.6	1.053	
O76094	Signal recognition particle subunit SRP72	74.56	9.26	140.55262	25.484352	16	26	16	97.4	102.6	1.053	8.55E-03
O95202	LETM1 and EF-hand domain-containing protein 1, mitochondrial	83.302	6.7	86.421159	12.85521	9	16	9	97.4	102.6	1.053	1.87E-02
P21283	V-type proton ATPase subunit C 1	43.914	7.46	42.720359	19.109948	9	12	9	97.4	102.6	1.053	1.93E-04
P22061	Protein-L-isoaspartate(D-aspartate) O-methyltransferase	24.621	7.21	28.011133	22.907489	4	8	4	97.4	102.6	1.053	6.75E-01
P37108	Signal recognition particle 14 kDa protein	14.561	10.04	85.051108	55.147059	7	21	7	97.4	102.6	1.053	2.43E-01
P67809	Nuclease-sensitive element-binding protein 1	35.903	9.88	127.1108	37.962963	7	30	6	97.4	102.6	1.053	1.57E-01
P78381	UDP-galactose translocator	41.282	9.96	8.5875395	2.7777778	1	1	1	97.4	102.6	1.053	
Q13158	FAS-associated death domain protein	23.265	5.69	48.475294	18.269231	3	6	3	97.4	102.6	1.053	1.59E-02
Q13206	Probable ATP-dependent RNA helicase DDX10	100.825	8.63	29.643689	5.6	4	4	4	97.4	102.6	1.053	3.62E-02
Q13283	Ras GTPase-activating protein-binding protein 1	52.132	5.52	104.81133	23.175966	8	30	7	97.4	102.6	1.053	8.89E-05
Q13443	Disintegrin and metalloproteinase domain-containing protein 9	90.497	7.52	42.779981	9.7680098	5	8	5	97.4	102.6	1.053	6.56E-03
Q13885	Tubulin beta-2A chain	49.875	4.89	259.98468	45.393258	15	167	2	97.4	102.6	1.053	8.09E-02
Q15942	Zyxin	61.238	6.67	84.470418	33.216783	13	25	13	97.4	102.6	1.053	3.00E-02
Q5VW38	Protein GPR107	66.948	7.24	10.319364	3.6666667	2	2	2	97.4	102.6	1.053	
Q6SJ93	Protein FAM111B	84.62	8.59	23.459381	5.4495913	4	5	4	97.4	102.6	1.053	1.92E-02
Q7L1Q6	Basic leucine zipper and W2 domain-containing protein 1	48.013	5.92	99.842114	25.775656	14	27	12	97.4	102.6	1.053	5.29E-01
Q8IWX8	Calcium homeostasis endoplasmic reticulum protein	103.637	9.04	69.389291	11.135371	9	14	9	97.4	102.6	1.053	6.53E-05
Q8TBB5	Kelch domain-containing protein 4	57.855	5.72	17.647363	6.3461538	3	3	3	97.4	102.6	1.053	
Q96AQ8	Mitochondrial calcium uniporter regulator 1	39.669	9.63	8.9353356	5.2924791	2	2	2	97.4	102.6	1.053	8.76E-01
Q9BXY0	Protein MAK16 homolog	35.347	5.38	36.49725	12.666667	3	5	3	97.4	102.6	1.053	9.57E-02
Q9BYC8	39S ribosomal protein L32, mitochondrial	21.391	9.73	21.162985	19.148936	2	3	2	97.4	102.6	1.053	
Q9GZT9	Egl nine homolog 1	45.992	8.53	65.864897	24.882629	10	14	10	97.4	102.6	1.053	3.63E-01
Q9NTX5	Ethylmalonyl-CoA decarboxylase	33.677	8.21	75.008671	27.035831	6	9	6	97.4	102.6	1.053	1.27E-01
Q9NYJ1	Cytochrome c oxidase assembly factor 4 homolog, mitochondrial	10.128	6.04	20.272606	36.781609	3	6	3	97.4	102.6	1.053	4.91E-02
Q9UJX4	Anaphase-promoting complex subunit 5	85.023	6.87	20.198169	5.6953642	3	3	3	97.4	102.6	1.053	
O00212	Rho-related GTP-binding protein RhoD	23.398	7.96	6.3747905	5.2380952	1	1	1	97.3	102.7	1.055	
O43290	U4/U6.U5 tri-snRNP-associated protein 1	90.2	6.13	145.02733	25.625	14	19	14	97.3	102.7	1.055	5.22E-02
O60220	Mitochondrial import inner membrane translocase subunit Tim8 A	10.991	5.16	16.858636	34.020619	3	4	3	97.3	102.7	1.055	
O75153	Clustered mitochondria protein homolog	146.577	6.13	116.07871	17.952636	22	31	22	97.3	102.7	1.055	7.62E-03
O75534	Cold shock domain-containing protein E1	88.829	6.25	199.94239	32.83208	26	43	26	97.3	102.7	1.055	9.65E-05
P08174	Complement decay-accelerating factor	41.374	7.59	106.02015	32.545932	12	32	12	97.3	102.7	1.055	5.59E-03
P14735	Insulin-degrading enzyme	117.893	6.61	137.0948	17.1737	16	31	16	97.3	102.7	1.055	1.69E-07
P29084	Transcription initiation factor IIE subunit beta	33.023	9.66	18.178552	13.058419	4	5	4	97.3	102.7	1.055	
P35527	Keratin, type I cytoskeletal 9	62.027	5.24	30.142713	8.8282504	5	6	4	97.3	102.7	1.055	
P40763	Signal transducer and activator of transcription 3	88.011	6.3	118.53522	20.12987	14	20	14	97.3	102.7	1.055	3.12E-02
P41227	N-alpha-acetyltransferase 10	26.442	5.64	38.522466	26.382979	6	8	6	97.3	102.7	1.055	1.39E-01
P42892	Endothelin-converting enzyme 1	87.108	5.88	31.904948	7.012987	5	5	5	97.3	102.7	1.055	6.52E-02
P43243	Matrin-3	94.565	6.25	174.05162	20.543093	15	33	15	97.3	102.7	1.055	3.35E-02
P53701	Cytochrome c-type heme lyase	30.582	6.68	20.635303	19.402985	5	6	5	97.3	102.7	1.055	2.36E-02
P61221	ATP-binding cassette sub-family E member 1	67.271	8.34	148.66704	33.722871	18	30	18	97.3	102.7	1.055	7.35E-03
P61619	Protein transport protein Sec61 subunit alpha isoform 1	52.231	8.06	32.790256	14.705882	6	10	6	97.3	102.7	1.055	5.55E-03
P62195	26S protease regulatory subunit 8	45.597	7.55	224.50653	52.216749	20	39	18	97.3	102.7	1.055	1.16E-04
P62306	Small nuclear ribonucleoprotein F	9.719	4.67	6.8407337	15.116279	1	4	1	97.3	102.7	1.055	
P78346	Ribonuclease P protein subunit p30	29.303	8.91	9.5109748	10.074627	3	4	3	97.3	102.7	1.055	1.72E-02
Q15021	Condensin complex subunit 1	157.082	6.61	177.53877	15.845824	21	39	21	97.3	102.7	1.055	1.35E-01

Q15291	Retinoblastoma-binding protein 5	59.116	5.1	26.502497	6.133829	2	4	2	97.3	102.7	1.055	1.60E-03
Q15345	Leucine-rich repeat-containing protein 41	88.594	8.38	23.876833	6.6502463	4	4	4	97.3	102.7	1.055	4.55E-02
Q15428	Splicing factor 3A subunit 2	49.224	9.64	73.914141	14.439655	5	13	5	97.3	102.7	1.055	4.81E-02
Q16881	Thioredoxin reductase 1, cytoplasmic	70.862	7.39	219.05363	27.734977	18	49	18	97.3	102.7	1.055	1.61E-04
Q53GS9	U4/U6.U5 tri-snRNP-associated protein 2	65.34	8.91	52.148606	16.99115	9	12	8	97.3	102.7	1.055	1.76E-02
Q5XPI4	E3 ubiquitin-protein ligase RNF123	148.42	6.74	10.277235	0.8371385	1	2	1	97.3	102.7	1.055	
Q7Z4Q2	HEAT repeat-containing protein 3	74.535	5.11	22.143719	8.6764706	6	6	6	97.3	102.7	1.055	3.42E-01
Q8IUF8	ifunctional lysine-specific demethylase and histidyl-hydroxylase MIN	52.767	6.7	18.245821	8.8172043	4	4	4	97.3	102.7	1.055	5.46E-02
Q8N684	Cleavage and polyadenylation specificity factor subunit 7	52.018	8	50.800957	15.07431	6	13	6	97.3	102.7	1.055	4.15E-02
Q8NBP7	Proprotein convertase subtilisin/kexin type 9	74.239	6.61	22.186677	6.7919075	4	4	4	97.3	102.7	1.055	
Q8ND56	Protein LSM14 homolog A	50.499	9.52	33.601184	10.367171	6	7	6	97.3	102.7	1.055	2.36E-02
Q92947	Glutaryl-CoA dehydrogenase, mitochondrial	48.096	8.06	22.940138	7.5342466	3	5	3	97.3	102.7	1.055	2.93E-01
Q99729	Heterogeneous nuclear ribonucleoprotein A/B	36.202	8.21	54.328497	15.060241	4	16	3	97.3	102.7	1.055	1.42E-02
Q99747	Gamma-soluble NSF attachment protein	34.724	5.41	16.5673	11.858974	4	4	4	97.3	102.7	1.055	8.62E-02
Q9H074	Polyadenylate-binding protein-interacting protein 1	53.491	4.81	30.151173	7.9331942	3	7	3	97.3	102.7	1.055	5.83E-01
Q9NPQ8	Synembryn-A	59.671	5.33	13.867433	7.3446328	5	8	4	97.3	102.7	1.055	2.83E-01
Q9NSI6	Bromodomain and WD repeat-containing protein 1	262.772	8.46	3.9913998	0.387931	1	1	1	97.3	102.7	1.055	
Q9NYZ3	G2 and S phase-expressed protein 1	76.598	9.39	5.9538952	1.1111111	1	1	1	97.3	102.7	1.055	
Q9NZ63	Uncharacterized protein C9orf78	33.667	6.74	46.64459	16.608997	6	8	6	97.3	102.7	1.055	4.03E-03
Q9P299	Coatomer subunit zeta-2	23.533	5.17	4.1827989	9.047619	2	2	2	97.3	102.7	1.055	
Q9P2I0	Cleavage and polyadenylation specificity factor subunit 2	88.431	5.11	44.245269	13.554987	9	9	9	97.3	102.7	1.055	1.63E-02
Q9UG63	ATP-binding cassette sub-family F member 2	71.245	7.37	105.72008	22.632424	15	23	15	97.3	102.7	1.055	2.81E-08
Q9Y4P3	Transducin beta-like protein 2	49.766	9.44	47.868961	13.199105	6	9	6	97.3	102.7	1.055	8.63E-01
Q9Y5U9	Immediate early response 3-interacting protein 1	8.963	8.22	19.035113	24.390244	1	2	1	97.3	102.7	1.055	
O00193	Small acidic protein	20.32	4.72	54.258656	17.486339	4	11	4	97.2	102.8	1.058	6.34E-03
O00762	Ubiquitin-conjugating enzyme E2 C	19.64	7.37	40.807588	25.698324	4	8	4	97.2	102.8	1.058	3.15E-01
O14545	TRAF-type zinc finger domain-containing protein 1	64.8	5.29	39.546324	12.371134	7	8	7	97.2	102.8	1.058	3.52E-02
O14646	Chromodomain-helicase-DNA-binding protein 1	196.567	7.14	15.701228	2.3976608	5	5	3	97.2	102.8	1.058	6.31E-02
O14975	Very long-chain acyl-CoA synthetase	70.267	8.51	24.379876	5.9677419	4	5	4	97.2	102.8	1.058	1.12E-01
O43933	Peroxisome biogenesis factor 1	142.778	6.33	5.542524	1.2470772	2	2	2	97.2	102.8	1.058	
O60216	Double-strand-break repair protein rad21 homolog	71.645	4.65	60.97611	17.274168	8	9	8	97.2	102.8	1.058	4.26E-03
O95816	BAG family molecular chaperone regulator 2	23.757	6.7	90.075226	47.867299	11	21	11	97.2	102.8	1.058	1.69E-05
P0CG39	POTE ankyrin domain family member J	117.315	5.97	31.679944	5.5876686	5	42	1	97.2	102.8	1.058	
P19387	DNA-directed RNA polymerase II subunit RPB3	31.422	4.92	44.661821	17.090909	4	10	4	97.2	102.8	1.058	6.88E-03
P21281	V-type proton ATPase subunit B, brain isoform	56.465	5.81	48.215097	17.02544	7	11	7	97.2	102.8	1.058	6.27E-02
P43304	Glycerol-3-phosphate dehydrogenase, mitochondrial	80.802	7.69	70.934354	19.11967	15	17	15	97.2	102.8	1.058	1.21E-02
P46776	60S ribosomal protein L27a	16.551	11	47.020859	29.72973	5	14	5	97.2	102.8	1.058	1.57E-04
P48507	Glutamate--cysteine ligase regulatory subunit	30.708	6.02	56.396861	25.182482	5	8	5	97.2	102.8	1.058	8.35E-02
P49642	DNA primase small subunit	49.87	8.21	21.758789	9.047619	4	6	3	97.2	102.8	1.058	6.37E-04
P55884	Eukaryotic translation initiation factor 3 subunit B	92.424	5	214.50925	28.624079	20	43	20	97.2	102.8	1.058	1.74E-04
P62917	60S ribosomal protein L8	28.007	11.03	37.532357	36.575875	8	11	8	97.2	102.8	1.058	1.18E-01
Q13485	Mothers against decapentaplegic homolog 4	60.401	6.99	9.6954075	3.9855072	3	3	3	97.2	102.8	1.058	
Q14807	Kinesin-like protein KIF22	73.217	9.45	13.491567	4.0601504	2	2	2	97.2	102.8	1.058	6.92E-02
Q5UIP0	Telomere-associated protein RIF1	274.294	5.52	80.084455	6.8770227	14	15	14	97.2	102.8	1.058	4.12E-02
Q6ZS86	Putative glycerol kinase 5	59.118	6.9	8.8027194	3.9697543	1	1	1	97.2	102.8	1.058	
Q86Y82	Syntaxin-12	31.622	5.59	22.063237	14.130435	3	3	3	97.2	102.8	1.058	
Q8IXH7	Negative elongation factor C/D	66.204	5.1	36.678909	8.1355932	4	7	4	97.2	102.8	1.058	2.43E-01

Q8IXM3	39S ribosomal protein L41, mitochondrial	15.373	9.57	12.418115	10.948905	1	3	1	97.2	102.8	1.058	
Q8N983	39S ribosomal protein L43, mitochondrial	23.416	8.65	13.093615	8.372093	2	4	2	97.2	102.8	1.058	4.35E-01
Q8NET6	Carbohydrate sulfotransferase 13	38.896	10.55	14.408808	6.744868	2	2	2	97.2	102.8	1.058	
Q8NFF5	FAD synthase	65.224	6.93	30.05774	10.221465	5	6	5	97.2	102.8	1.058	9.58E-01
Q8TDP1	Ribonuclease H2 subunit C	17.829	5.03	26.023937	9.1463415	1	3	1	97.2	102.8	1.058	
Q96CM3	RNA pseudouridylate synthase domain-containing protein 4	42.178	9.88	5.2198988	2.9177719	1	2	1	97.2	102.8	1.058	
Q96GQ7	Probable ATP-dependent RNA helicase DDX27	89.779	9.28	91.889385	17.462312	14	20	14	97.2	102.8	1.058	4.57E-02
Q96IX5	Up-regulated during skeletal muscle growth protein 5	6.453	9.76	8.2862575	25.862069	1	1	1	97.2	102.8	1.058	
Q96T88	E3 ubiquitin-protein ligase UHRF1	89.757	7.56	112.78362	20.807062	14	21	13	97.2	102.8	1.058	8.74E-04
Q9BTD8	RNA-binding protein 42	50.382	9.63	31.193768	7.9166667	2	5	2	97.2	102.8	1.058	1.68E-01
Q9BWW4	Single-stranded DNA-binding protein 3	40.395	6.9	12.83612	5.9278351	3	3	2	97.2	102.8	1.058	
Q9BY77	Polymerase delta-interacting protein 3	46.06	9.99	17.320143	8.7885986	3	3	3	97.2	102.8	1.058	
Q9H4G0	Band 4.1-like protein 1	98.442	5.62	143.62926	24.744608	18	33	18	97.2	102.8	1.058	9.72E-06
Q9H6H4	Receptor expression-enhancing protein 4	29.376	9.73	6.6869786	7.0038911	2	2	2	97.2	102.8	1.058	
Q9H7F0	Probable cation-transporting ATPase 13A3	137.953	6.64	4.0118425	0.8156607	1	1	1	97.2	102.8	1.058	
Q9Y2W1	Thyroid hormone receptor-associated protein 3	108.601	10.15	112.33124	18.534031	14	24	14	97.2	102.8	1.058	8.32E-02
Q9Y3B7	39S ribosomal protein L11, mitochondrial	20.67	9.91	44.613241	33.854167	6	9	6	97.2	102.8	1.058	5.48E-02
Q9Y448	Small kinetochore-associated protein	35.416	6.3	32.15681	21.835443	7	7	6	97.2	102.8	1.058	5.48E-02
Q9Y617	Phosphoserine aminotransferase	40.397	7.66	137.7659	41.081081	14	31	14	97.2	102.8	1.058	4.09E-04
O15270	Serine palmitoyltransferase 2	62.884	7.78	26.314565	10.676157	6	6	6	97.1	102.9	1.06	7.40E-04
O15498	Synaptobrevin homolog YKT6	22.403	6.92	10.202407	9.0909091	2	2	2	97.1	102.9	1.06	3.48E-01
O15530	3-phosphoinositide-dependent protein kinase 1	63.112	7.36	18.609406	8.9928058	5	5	5	97.1	102.9	1.06	
O60508	Pre-mRNA-processing factor 17	65.479	7.06	19.288195	3.7996546	2	3	2	97.1	102.9	1.06	
O60716	Catenin delta-1	108.103	6.23	76.586161	14.876033	11	13	11	97.1	102.9	1.06	4.35E-03
O96019	Actin-like protein 6A	47.43	5.6	69.871128	20.745921	8	15	8	97.1	102.9	1.06	5.31E-05
P08134	Rho-related GTP-binding protein RhoC	21.992	6.58	71.951843	33.160622	7	15	1	97.1	102.9	1.06	
P23246	Splicing factor, proline- and glutamine-rich	76.102	9.44	258.29807	32.390382	25	77	22	97.1	102.9	1.06	6.61E-02
P33897	ATP-binding cassette sub-family D member 1	82.885	8.95	41.03179	10.201342	6	7	6	97.1	102.9	1.06	2.89E-01
P53350	Serine/threonine-protein kinase PLK1	68.212	8.91	22.158981	9.9502488	5	5	5	97.1	102.9	1.06	1.81E-01
P55010	Eukaryotic translation initiation factor 5	49.192	5.58	101.89451	26.450116	10	24	10	97.1	102.9	1.06	2.09E-04
P84095	Rho-related GTP-binding protein RhoG	21.295	8.12	81.716898	45.549738	7	12	6	97.1	102.9	1.06	5.78E-03
Q04828	Aldo-keto reductase family 1 member C1	36.765	7.88	35.176492	21.671827	6	10	2	97.1	102.9	1.06	
Q15043	Zinc transporter ZIP14	54.177	5.33	33.542493	9.1463415	3	5	3	97.1	102.9	1.06	1.16E-01
Q15599	Na(+)/H(+) exchange regulatory cofactor NHE-RF2	37.391	7.93	27.769882	19.287834	6	8	6	97.1	102.9	1.06	9.54E-03
Q5T1V6	Probable ATP-dependent RNA helicase DDX59	68.766	7.44	25.83152	9.3699515	5	8	4	97.1	102.9	1.06	1.48E-01
Q5T3J3	Ligand-dependent nuclear receptor-interacting factor 1	84.516	9.72	6.4136378	1.4304291	1	1	1	97.1	102.9	1.06	
Q86U38	Nucleolar protein 9	69.394	7.28	59.00548	13.207547	8	10	7	97.1	102.9	1.06	3.93E-03
Q86X52	Chondroitin sulfate synthase 1	91.726	9.23	8.190171	1.6209476	1	1	1	97.1	102.9	1.06	
Q8NCN5	Pyruvate dehydrogenase phosphatase regulatory subunit, mitochondria	99.301	6.35	8.9801841	3.185438	3	3	3	97.1	102.9	1.06	3.16E-01
Q8TBX8	Phosphatidylinositol 5-phosphate 4-kinase type-2 gamma	47.27	6.84	22.373395	9.976247	5	6	5	97.1	102.9	1.06	7.15E-02
Q8TEQ6	Gem-associated protein 5	168.483	6.62	135.87665	14.190981	18	28	18	97.1	102.9	1.06	1.13E-04
Q8WW01	tRNA-splicing endonuclease subunit Sen15	18.629	4.58	13.093143	23.976608	2	2	2	97.1	102.9	1.06	7.15E-02
Q96EE3	Nucleoporin SEH1	39.623	8.09	72.869944	24.444444	7	14	7	97.1	102.9	1.06	4.17E-01
Q96PU5	E3 ubiquitin-protein ligase NEDD4-like	111.862	5.82	25.686589	3.4871795	3	5	3	97.1	102.9	1.06	5.60E-01
Q96PZ0	Pseudouridylate synthase 7 homolog	74.988	6.37	92.566409	22.239032	13	17	13	97.1	102.9	1.06	2.12E-03
Q96SZ6	CDK5 regulatory subunit-associated protein 1	67.646	8.21	8.854893	3.8269551	2	2	2	97.1	102.9	1.06	
Q96TA2	ATP-dependent zinc metalloprotease YME1L1	86.401	8.76	46.525972	12.160414	7	7	7	97.1	102.9	1.06	1.93E-02

Q9BY43	Charged multivesicular body protein 4a	25.083	4.7	61.818443	31.081081	7	11	7	97.1	102.9	1.06	1.32E-02
Q9H9F9	Actin-related protein 5	68.254	5.27	5.0489054	2.9654036	1	1	1	97.1	102.9	1.06	
Q9HC35	Echinoderm microtubule-associated protein-like 4	108.848	6.4	51.928657	9.8878695	9	10	9	97.1	102.9	1.06	2.02E-02
Q9NQ88	Fructose-2,6-bisphosphatase TIGAR	30.043	7.69	20.921435	6.2962963	1	2	1	97.1	102.9	1.06	
Q9UEU0	Vesicle transport through interaction with t-SNAREs homolog 1B	26.672	9.04	9.2735646	10.344828	2	2	2	97.1	102.9	1.06	4.30E-02
Q9Y314	Nitric oxide synthase-interacting protein	33.151	8.82	27.356371	18.936877	4	5	4	97.1	102.9	1.06	2.47E-01
Q9Y3C4	EKC/KEOPS complex subunit TPRKB	19.648	6.79	24.539714	25.142857	3	4	3	97.1	102.9	1.06	5.94E-04
Q9Y3T9	Nucleolar complex protein 2 homolog	84.866	5.62	67.24545	12.416555	9	14	9	97.1	102.9	1.06	1.99E-04
O15212	Prefoldin subunit 6	14.574	8.88	53.331066	50.387597	9	16	9	97	103	1.062	6.82E-03
O94804	Serine/threonine-protein kinase 10	112.065	6.95	37.177722	7.7479339	7	9	6	97	103	1.062	1.18E-01
P20248	Cyclin-A2	48.52	6.54	18.305291	9.9537037	3	4	3	97	103	1.062	2.14E-01
P29558	RNA-binding motif, single-stranded-interacting protein 1	44.477	8.79	28.539215	9.8522167	3	6	2	97	103	1.062	
P30048	Thioredoxin-dependent peroxide reductase, mitochondrial	27.675	7.78	43.385207	17.1875	4	13	4	97	103	1.062	6.89E-02
P37198	Nuclear pore glycoprotein p62	53.222	5.31	40.548406	11.302682	5	6	5	97	103	1.062	6.46E-02
P49354	otein farnesyltransferase/geranylgeranyltransferase type-1 subunit alpl	44.381	5.08	13.19315	6.5963061	3	3	3	97	103	1.062	5.94E-04
P49841	Glycogen synthase kinase-3 beta	46.715	8.78	61.967846	29.52381	8	9	5	97	103	1.062	2.73E-01
P61956	Small ubiquitin-related modifier 2	10.864	5.5	30.794532	27.368421	3	7	2	97	103	1.062	1.59E-04
P62913	60S ribosomal protein L11	20.24	9.6	41.652667	25.842697	6	14	6	97	103	1.062	3.59E-01
P83881	60S ribosomal protein L36a	12.433	10.58	9.483549	23.584906	3	3	3	97	103	1.062	4.65E-03
P98179	RNA-binding protein 3	17.16	8.91	6.0504114	13.375796	2	3	2	97	103	1.062	2.46E-01
Q01130	Serine/arginine-rich splicing factor 2	25.461	11.85	13.453161	14.932127	3	6	3	97	103	1.062	1.36E-02
Q14146	Unhealthy ribosome biogenesis protein 2 homolog	170.435	7.31	13.00115	2.0341207	3	3	3	97	103	1.062	1.44E-04
Q14157	Ubiquitin-associated protein 2-like	114.465	7.11	144.37441	24.195032	14	25	14	97	103	1.062	3.54E-05
Q5C9Z4	Nucleolar MIF4G domain-containing protein 1	96.198	8.1	15.645045	2.7906977	2	2	2	97	103	1.062	2.00E-01
Q5VT52	Regulation of nuclear pre-mRNA domain-containing protein 2	155.924	7.42	68.253559	10.26694	11	13	11	97	103	1.062	6.61E-06
Q68D10	Protein SPT2 homolog	75.553	9.79	8.9178323	4.2335766	3	3	3	97	103	1.062	2.26E-01
Q6MZP7	Protein lin-54 homolog	79.444	9.01	3.3414163	1.4686248	1	1	1	97	103	1.062	
Q6NTF9	Rhomboid domain-containing protein 2	39.177	9.32	4.8840568	2.4725275	1	1	1	97	103	1.062	
Q6Y1H2	Very-long-chain (3R)-3-hydroxyacyl-CoA dehydratase 2	28.35	9.55	19.686183	7.480315	2	5	2	97	103	1.062	1.28E-01
Q7L8L6	FAST kinase domain-containing protein 5	86.519	8.13	24.073864	5.4973822	4	5	4	97	103	1.062	2.47E-03
Q8N1G2	Cap-specific mRNA (nucleoside-2'-O-)-methyltransferase 1	95.26	7.05	22.690949	5.3892216	4	4	4	97	103	1.062	1.68E-03
Q8NBJ7	Sulfatase-modifying factor 2	33.822	8	47.744381	28.239203	8	10	8	97	103	1.062	3.60E-02
Q8NDH3	Probable aminopeptidase NPEPL1	55.825	6.87	27.612446	11.472275	4	4	4	97	103	1.062	9.96E-03
Q8NFA0	Ubiquitin carboxyl-terminal hydrolase 32	181.54	6.44	5.5700863	0.6857855	1	1	1	97	103	1.062	
Q8WV44	E3 ubiquitin-protein ligase TRIM41	71.625	5.06	7.4568901	4.6031746	2	2	2	97	103	1.062	1.39E-01
Q92945	Far upstream element-binding protein 2	73.07	7.3	211.77243	37.130802	23	48	19	97	103	1.062	5.63E-01
Q96H55	Unconventional myosin-XIX	109.066	7.71	5.6538427	0.9278351	1	1	1	97	103	1.062	
Q9BWH2	FUN14 domain-containing protein 2	20.663	9.73	10.675618	10.582011	3	4	3	97	103	1.062	1.29E-03
Q9UHV9	Prefoldin subunit 2	16.638	6.58	45.711405	37.662338	6	10	6	97	103	1.062	4.63E-01
Q9UNN8	Endothelial protein C receptor	26.655	7.18	13.324174	5.4621849	1	2	1	97	103	1.062	
Q9UPZ3	Hermansky-Pudlak syndrome 5 protein	127.368	5.54	28.23324	3.7201063	4	5	4	97	103	1.062	1.40E-01
O14907	Tax1-binding protein 3	13.726	8.48	30.032432	25.806452	3	4	3	96.9	103.1	1.064	
O95169	H dehydrogenase [ubiquinone] 1 beta subcomplex subunit 8, mitochor	21.751	6.8	3.0044964	5.9139785	1	1	1	96.9	103.1	1.064	
O95685	Protein phosphatase 1 regulatory subunit 3D	32.538	8.07	14.63893	9.6989967	2	3	2	96.9	103.1	1.064	
O95801	Tetratricopeptide repeat protein 4	44.65	5.6	28.514562	7.4935401	2	3	2	96.9	103.1	1.064	1.26E-04
P11388	DNA topoisomerase 2-alpha	174.276	8.72	258.36875	24.689745	35	52	25	96.9	103.1	1.064	1.35E-04
P50748	Kinetochore-associated protein 1	250.588	5.97	70.933273	7.6052512	18	18	18	96.9	103.1	1.064	1.45E-03

P51114	Fragile X mental retardation syndrome-related protein 1	69.678	6.15	58.274747	14.170692	9	13	7	96.9	103.1	1.064	6.07E-02
P84243	Histone H3.3	15.319	11.27	24.984189	31.617647	7	16	2	96.9	103.1	1.064	6.96E-03
Q03393	6-pyruvoyl tetrahydrobiopterin synthase	16.375	6.68	13.35678	21.37931	3	3	3	96.9	103.1	1.064	3.17E-01
Q04727	Transducin-like enhancer protein 4	83.703	7.5	16.330501	6.2095731	5	5	2	96.9	103.1	1.064	
Q13033	Striatin-3	87.155	5.36	8.0327684	2.8858218	2	2	1	96.9	103.1	1.064	
Q14165	Malectin	32.214	5.41	22.040152	11.643836	3	4	3	96.9	103.1	1.064	2.03E-01
Q15910	Histone-lysine N-methyltransferase EZH2	85.309	7.02	7.8285661	1.4745308	1	1	1	96.9	103.1	1.064	
Q68CP9	AT-rich interactive domain-containing protein 2	197.268	7.42	23.774159	2.7792916	4	4	4	96.9	103.1	1.064	
Q6ICG6	Uncharacterized protein KIAA0930	45.765	7.94	5.67305	2.2277228	1	1	1	96.9	103.1	1.064	
Q7L2J0	7SK snRNA methylphosphate capping enzyme	74.31	9.57	44.413568	14.223512	7	9	7	96.9	103.1	1.064	1.76E-02
Q8IYB3	Serine/arginine repetitive matrix protein 1	102.274	11.84	54.788819	8.7389381	5	11	5	96.9	103.1	1.064	1.64E-01
Q8N0X7	Spartin	72.788	5.91	64.971885	12.162162	9	12	8	96.9	103.1	1.064	2.12E-01
Q8NFH5	Nucleoporin NUP53	34.751	9.09	27.26981	14.417178	4	5	4	96.9	103.1	1.064	2.93E-01
Q92547	DNA topoisomerase 2-binding protein 1	170.571	6.96	17.565179	2.7595269	4	4	4	96.9	103.1	1.064	4.83E-02
Q96BR5	Cytochrome c oxidase assembly factor 7	25.692	6.02	11.718062	13.419913	4	4	4	96.9	103.1	1.064	3.20E-01
Q96N67	Dedicator of cytokinesis protein 7	242.407	6.8	16.74882	1.682243	4	4	4	96.9	103.1	1.064	
Q99627	COP9 signalosome complex subunit 8	23.211	5.38	39.513655	28.708134	4	5	4	96.9	103.1	1.064	1.85E-02
Q9BRA2	Thioredoxin domain-containing protein 17	13.932	5.52	24.555538	38.211382	4	5	4	96.9	103.1	1.064	4.96E-03
Q9H2P0	Activity-dependent neuroprotector homeobox protein	123.485	7.34	38.141567	8.6206897	8	9	8	96.9	103.1	1.064	1.21E-02
A6ZKI3	Protein FAM127A	13.163	5.07	8.5001755	8.8495575	1	1	1	96.8	103.2	1.066	
O00194	Ras-related protein Rab-27B	24.592	5.52	3.2266525	5.0458716	1	1	1	96.8	103.2	1.066	
O75937	DnaJ homolog subfamily C member 8	29.823	9.06	46.022155	24.901186	7	13	7	96.8	103.2	1.066	8.85E-01
O94766	Galactosylgalactosylxylosylprotein 3-beta-glucuronosyltransferase 3	37.099	8.27	4.0547284	2.6865672	1	1	1	96.8	103.2	1.066	
O95104	Splicing factor, arginine/serine-rich 15	125.79	9.55	16.169274	4.097646	4	4	4	96.8	103.2	1.066	1.40E-01
P11940	Polyadenylate-binding protein 1	70.626	9.5	220.18623	32.232704	22	62	16	96.8	103.2	1.066	1.13E-01
P18084	Integrin beta-5	87.996	6.06	23.49373	6.1326658	4	4	4	96.8	103.2	1.066	3.01E-02
P46783	40S ribosomal protein S10	18.886	10.15	89.655158	53.333333	12	39	11	96.8	103.2	1.066	2.84E-04
P62280	40S ribosomal protein S11	18.419	10.3	60.537973	50	10	17	10	96.8	103.2	1.066	5.00E-04
P62834	Ras-related protein Rap-1A	20.974	6.67	86.518681	51.630435	7	15	3	96.8	103.2	1.066	1.26E-01
P62851	40S ribosomal protein S25	13.734	10.11	50.744319	38.4	6	22	6	96.8	103.2	1.066	1.76E-03
Q06787	Fragile X mental retardation protein 1	71.131	7.42	84.562296	15.981013	8	14	6	96.8	103.2	1.066	6.44E-02
Q13247	Serine/arginine-rich splicing factor 6	39.563	11.43	40.629169	17.151163	7	13	2	96.8	103.2	1.066	
Q13427	Peptidyl-prolyl cis-trans isomerase G	88.564	10.29	12.421788	4.7745358	3	3	3	96.8	103.2	1.066	1.96E-01
Q13951	Core-binding factor subunit beta	21.495	6.6	5.4370052	9.3406593	2	2	2	96.8	103.2	1.066	
Q15121	Astrocytic phosphoprotein PEA-15	15.031	5.02	17.96313	22.307692	2	2	2	96.8	103.2	1.066	1.82E-02
Q15155	Nodal modulator 1	134.239	5.81	109.54091	13.91162	14	21	2	96.8	103.2	1.066	1.94E-02
Q15238	Pregnancy-specific beta-1-glycoprotein 5	37.689	8.87	2.8271053	2.0895522	1	1	1	96.8	103.2	1.066	
Q27J81	Inverted formin-2	135.54	5.38	213.94851	25.460368	19	36	19	96.8	103.2	1.066	3.15E-06
Q49B96	Cytochrome c oxidase assembly protein COX19	10.387	8.72	3.6520848	11.111111	1	1	1	96.8	103.2	1.066	
Q5T0F9	Coiled-coil and C2 domain-containing protein 1B	94.166	5.26	16.455899	4.6620047	4	4	4	96.8	103.2	1.066	
Q7L5D6	Golgi to ER traffic protein 4 homolog	36.481	5.41	36.464376	11.620795	3	7	3	96.8	103.2	1.066	2.93E-02
Q86WQ0	Nuclear receptor 2C2-associated protein	15.866	6.16	20.830047	35.971223	3	3	3	96.8	103.2	1.066	6.06E-01
Q8N392	Rho GTPase-activating protein 18	74.93	6.44	66.816846	10.859729	6	9	6	96.8	103.2	1.066	7.32E-05
Q8WWI1	LIM domain only protein 7	192.576	8.09	311.53291	26.024955	33	53	33	96.8	103.2	1.066	5.63E-06
Q8WYH8	Inhibitor of growth protein 5	27.733	7.61	2.3136319	3.3333333	1	1	1	96.8	103.2	1.066	
Q92828	Coronin-2A	59.725	8.05	8.5870357	2.0952381	1	1	1	96.8	103.2	1.066	
Q969E2	Secretory carrier-associated membrane protein 4	25.711	8.82	11.400617	4.8034934	1	2	1	96.8	103.2	1.066	

Q969S3	Zinc finger protein 622	54.237	6.15	57.103315	21.802935	9	12	9	96.8	103.2	1.066	4.03E-04
Q96NC0	Zinc finger matrin-type protein 2	23.597	9.01	4.664876	8.040201	2	2	2	96.8	103.2	1.066	
Q9BQ48	39S ribosomal protein L34, mitochondrial	10.159	12.25	4.8471004	10.869565	1	1	1	96.8	103.2	1.066	
Q9BUN8	Derlin-1	28.782	9.51	2.9905491	4.3824701	1	1	1	96.8	103.2	1.066	
Q9H4B0	obable tRNA N6-adenosine threonylcarbamoyltransferase, mitochondr	45.094	8.56	6.7164726	3.1400966	1	1	1	96.8	103.2	1.066	
Q9UKA9	Polypyrimidine tract-binding protein 2	57.455	8.66	33.974353	10.169492	4	8	3	96.8	103.2	1.066	1.06E-03
O00505	Importin subunit alpha-4	57.775	4.94	27.761561	5.950096	3	7	2	96.7	103.3	1.068	
O00629	Importin subunit alpha-3	57.851	4.96	34.129702	16.314779	5	8	4	96.7	103.3	1.068	8.48E-01
O14949	Cytochrome b-c1 complex subunit 8	9.9	10.08	11.174073	23.170732	3	3	3	96.7	103.3	1.068	3.20E-01
O95433	Activator of 90 kDa heat shock protein ATPase homolog 1	38.25	5.53	69.716361	24.556213	6	12	6	96.7	103.3	1.068	4.21E-01
P31350	Ribonucleoside-diphosphate reductase subunit M2	44.849	5.38	85.472833	35.218509	10	15	9	96.7	103.3	1.068	8.56E-03
P34931	Heat shock 70 kDa protein 1-like	70.331	6.02	187.82921	24.024961	12	68	1	96.7	103.3	1.068	
P56378	6.8 kDa mitochondrial proteolipid	6.658	10.08	4.7954317	15.517241	1	2	1	96.7	103.3	1.068	
P63146	Ubiquitin-conjugating enzyme E2 B	17.302	5.01	7.3432312	11.184211	1	1	1	96.7	103.3	1.068	
P63241	Eukaryotic translation initiation factor 5A-1	16.821	5.24	57.178539	43.506494	7	21	7	96.7	103.3	1.068	3.49E-01
P82912	28S ribosomal protein S11, mitochondrial	20.603	10.81	19.73654	20.103093	3	3	3	96.7	103.3	1.068	
Q12792	Twinfilin-1	40.258	6.96	53.180307	18.857143	5	11	5	96.7	103.3	1.068	4.89E-02
Q14978	Nucleolar and coiled-body phosphoprotein 1	73.56	9.47	87.593128	23.319027	18	30	18	96.7	103.3	1.068	2.95E-07
Q3MHD2	Protein LSM12 homolog	21.687	7.74	26.456304	18.461538	3	5	3	96.7	103.3	1.068	1.29E-01
Q53FA7	Quinone oxidoreductase PIG3	35.514	7.17	32.198282	21.987952	7	7	7	96.7	103.3	1.068	5.04E-04
Q6P087	RNA pseudouridylate synthase domain-containing protein 3	38.437	10.32	13.372968	10.826211	3	3	3	96.7	103.3	1.068	
Q6P1L8	39S ribosomal protein L14, mitochondrial	15.937	10.24	33.489095	26.206897	3	5	3	96.7	103.3	1.068	3.45E-02
Q6PJI9	WD repeat-containing protein 59	109.724	7.91	10.108084	2.2587269	2	2	2	96.7	103.3	1.068	1.02E-04
Q6ULP2	Aftiphilin	102.137	4.54	2.8535619	1.0672359	1	1	1	96.7	103.3	1.068	
Q8N5P1	Zinc finger CCCH domain-containing protein 8	33.554	8.28	6.4664821	5.4982818	1	1	1	96.7	103.3	1.068	
Q8N9T8	Protein KRI1 homolog	82.548	5.14	36.966206	4.6941679	3	5	3	96.7	103.3	1.068	4.59E-04
Q8NFX7	Syntaxin-binding protein 6	23.539	9.04	13.74926	14.285714	3	3	3	96.7	103.3	1.068	8.09E-02
Q8TDM6	Disks large homolog 5	213.735	7.42	15.522907	1.6675352	3	3	3	96.7	103.3	1.068	1.78E-01
Q96DZ1	Endoplasmic reticulum lectin 1	54.823	6.28	21.368366	9.1097308	4	4	4	96.7	103.3	1.068	1.34E-01
Q96SI9	Spermatid perinuclear RNA-binding protein	73.606	8.72	43.186489	5.952381	4	13	1	96.7	103.3	1.068	
Q9H0K6	Pseudouridylate synthase 7 homolog-like protein	80.649	7.56	21.187265	4.5649073	3	3	3	96.7	103.3	1.068	2.69E-02
Q9H2K8	Serine/threonine-protein kinase TAO3	105.34	7.3	26.974665	6.2360802	6	6	6	96.7	103.3	1.068	1.21E-01
Q9H3Z4	DnaJ homolog subfamily C member 5	22.134	5.07	16.150228	7.0707071	1	2	1	96.7	103.3	1.068	
Q9H410	Kinetochore-associated protein DSN1 homolog	40.042	7.05	7.027496	5.6179775	2	2	2	96.7	103.3	1.068	
Q9H8M5	Metal transporter CNNM2	96.562	6.38	3.5885487	1.0285714	1	1	1	96.7	103.3	1.068	
Q9HAV7	GrpE protein homolog 1, mitochondrial	24.264	8.12	42.931601	30.875576	7	10	7	96.7	103.3	1.068	1.81E-03
Q9NVC6	Mediator of RNA polymerase II transcription subunit 17	72.845	7.44	29.868987	10.291859	5	5	5	96.7	103.3	1.068	1.40E-02
Q9NVM9	Protein asunder homolog	80.174	6.7	31.711813	8.7818697	6	8	6	96.7	103.3	1.068	8.50E-01
Q9UHB7	AF4/FMR2 family member 4	127.382	9.31	4.1819061	1.4617369	1	1	1	96.7	103.3	1.068	
Q9UIS9	Methyl-CpG-binding domain protein 1	66.564	9.04	4.4253901	1.9834711	1	1	1	96.7	103.3	1.068	
Q9UN37	Vacuolar protein sorting-associated protein 4A	48.867	7.8	37.544985	14.874142	7	10	4	96.7	103.3	1.068	7.39E-02
Q9Y3U8	60S ribosomal protein L36	12.246	11.59	38.411765	35.238095	5	11	4	96.7	103.3	1.068	2.47E-01
Q9Y5Q9	General transcription factor 3C polypeptide 3	101.208	5.07	25.765314	5.0790068	4	6	4	96.7	103.3	1.068	
Q9Y6Y8	SEC23-interacting protein	111.007	5.54	39.844976	8.8	7	11	6	96.7	103.3	1.068	7.24E-02
O14662	Syntaxin-16	37.008	6.11	4.0181814	2.7692308	1	1	1	96.6	103.4	1.07	
O14981	TATA-binding protein-associated factor 172	206.756	6.52	27.505195	3.7317469	7	7	7	96.6	103.4	1.07	1.39E-01
O43598	2'-deoxynucleoside 5'-phosphate N-hydrolase 1	19.097	5.05	15.892811	20.114943	2	2	2	96.6	103.4	1.07	

O43665	Regulator of G-protein signaling 10	20.223	5.49	14.100587	16.184971	3	4	3	96.6	103.4	1.07	1.27E-01
O43676	NADH dehydrogenase [ubiquinone] 1 beta subcomplex subunit 3	11.395	9.2	9.2071489	21.428571	2	3	2	96.6	103.4	1.07	3.07E-02
O94925	Glutaminase kidney isoform, mitochondrial	73.414	7.77	38.473065	9.2675635	5	9	5	96.6	103.4	1.07	1.12E-01
O95983	Methyl-CpG-binding domain protein 3	32.823	5.34	20.615027	15.120275	4	4	4	96.6	103.4	1.07	4.71E-01
P05023	Sodium/potassium-transporting ATPase subunit alpha-1	112.824	5.49	291.94086	32.746823	30	59	30	96.6	103.4	1.07	1.15E-03
P07942	Laminin subunit beta-1	197.909	4.94	115.6991	9.9104143	14	16	14	96.6	103.4	1.07	2.49E-01
P20936	Ras GTPase-activating protein 1	116.329	6.54	5.4861168	1.0506208	1	1	1	96.6	103.4	1.07	
P22694	cAMP-dependent protein kinase catalytic subunit beta	40.597	8.78	30.155992	14.529915	5	6	2	96.6	103.4	1.07	
P38571	Lysosomal acid lipase/cholesteryl ester hydrolase	45.39	6.92	13.06893	2.7568922	1	2	1	96.6	103.4	1.07	
P40222	Alpha-taxilin	61.853	6.52	145.02686	29.120879	17	29	16	96.6	103.4	1.07	5.44E-04
P42566	Epidermal growth factor receptor substrate 15	98.595	4.64	59.221407	12.946429	9	11	9	96.6	103.4	1.07	6.69E-04
P52789	Hexokinase-2	102.313	6.05	26.079406	3.1624864	3	6	2	96.6	103.4	1.07	
P61204	ADP-ribosylation factor 3	20.588	7.43	111.38412	35.359116	7	32	4	96.6	103.4	1.07	2.24E-01
P63098	Calcineurin subunit B type 1	19.288	4.81	10.039076	12.941176	2	2	2	96.6	103.4	1.07	
P78362	SRSF protein kinase 2	77.478	4.97	24.988339	6.3953488	4	5	3	96.6	103.4	1.07	
Q02750	Dual specificity mitogen-activated protein kinase kinase 1	43.411	6.62	40.105104	15.012723	5	9	3	96.6	103.4	1.07	1.86E-02
Q06481	Amyloid-like protein 2	86.9	4.79	22.735399	5.3735256	4	4	4	96.6	103.4	1.07	2.36E-02
Q08AE8	Protein spire homolog 1	85.489	8.62	8.9536684	3.8359788	2	5	1	96.6	103.4	1.07	
Q13129	Zinc finger protein Rlf	217.813	6.77	4.9965395	0.6792059	1	1	1	96.6	103.4	1.07	
Q13769	THO complex subunit 5 homolog	78.458	6.87	28.283994	6.4421669	4	5	4	96.6	103.4	1.07	2.28E-02
Q14699	Raftlin	63.106	5.67	23.76953	11.245675	5	6	5	96.6	103.4	1.07	3.93E-03
Q15050	Ribosome biogenesis regulatory protein homolog	41.168	10.7	54.62896	21.09589	7	10	7	96.6	103.4	1.07	1.82E-04
Q641Q2	WASH complex subunit FAM21A	147.095	4.81	70.410284	10.738255	10	11	10	96.6	103.4	1.07	8.29E-05
Q6PIU2	Neutral cholesterol ester hydrolase 1	45.779	7.23	43.984844	20.098039	7	8	7	96.6	103.4	1.07	4.53E-02
Q6ZS17	Protein FAM65A	132.225	6.28	54.876452	7.4407195	8	11	8	96.6	103.4	1.07	8.50E-02
Q8IUX1	Complex I assembly factor TMEM126B, mitochondrial	25.926	8.81	9.4914763	6.0869565	1	2	1	96.6	103.4	1.07	
Q8N4C8	Misshapen-like kinase 1	149.729	7.85	4.477295	0.7507508	1	1	1	96.6	103.4	1.07	
Q8NEB9	Phosphatidylinositol 3-kinase catalytic subunit type 3	101.485	6.81	50.825476	11.950395	9	11	9	96.6	103.4	1.07	8.11E-02
Q93034	Cullin-5	90.897	7.94	38.477617	8.974359	6	8	6	96.6	103.4	1.07	4.85E-03
Q969X6	Cirhin	76.841	8.85	44.108078	11.370262	7	9	7	96.6	103.4	1.07	2.62E-02
Q96RK0	Protein capicua homolog	163.719	8.56	4.3231236	0.6840796	1	1	1	96.6	103.4	1.07	
Q9BVJ6	U3 small nucleolar RNA-associated protein 14 homolog A	87.924	7.87	67.900493	14.007782	9	12	9	96.6	103.4	1.07	3.21E-03
Q9BYN8	28S ribosomal protein S26, mitochondrial	24.197	10.39	42.84905	25.853659	5	8	5	96.6	103.4	1.07	4.07E-01
Q9H299	SH3 domain-binding glutamic acid-rich-like protein 3	10.431	4.93	53.566131	38.709677	4	12	4	96.6	103.4	1.07	9.84E-03
Q9H6F5	Coiled-coil domain-containing protein 86	40.211	10.33	38.365835	22.5	6	7	6	96.6	103.4	1.07	1.94E-01
Q9NPF4	Probable tRNA N6-adenosine threonylcarbamoyltransferase	36.403	6.35	21.692381	14.029851	4	5	4	96.6	103.4	1.07	3.29E-02
Q9UBW7	Zinc finger MYM-type protein 2	154.81	6.34	8.9266836	2.033406	2	2	2	96.6	103.4	1.07	
Q9UGI8	Testin	47.964	7.68	159.49859	36.104513	13	32	13	96.6	103.4	1.07	6.80E-01
Q9UNY4	Transcription termination factor 2	129.508	8.37	3.8696662	0.8605852	1	1	1	96.6	103.4	1.07	
Q9Y5A9	YTH domain-containing family protein 2	62.296	8.79	47.218699	12.435233	7	8	4	96.6	103.4	1.07	1.03E-02
P02795	Metallothionein-2	6.037	7.83	75.076508	67.213115	4	17	2	96.5	103.5	1.073	3.12E-01
P05026	Sodium/potassium-transporting ATPase subunit beta-1	35.039	8.53	39.919163	20.792079	6	8	6	96.5	103.5	1.073	3.25E-02
P07919	Cytochrome b-c1 complex subunit 6, mitochondrial	10.732	4.44	30.019211	35.164835	3	10	3	96.5	103.5	1.073	
P17480	Nucleolar transcription factor 1	89.35	5.81	80.426501	15.706806	10	16	10	96.5	103.5	1.073	1.06E-03
P25445	Tumor necrosis factor receptor superfamily member 6	37.708	7.94	9.4796774	7.1641791	2	2	2	96.5	103.5	1.073	
P36969	Phospholipid hydroperoxide glutathione peroxidase, mitochondrial	22.161	8.37	12.464784	20.304569	3	3	3	96.5	103.5	1.073	
P52292	Importin subunit alpha-1	57.826	5.4	197.52965	35.916824	13	28	13	96.5	103.5	1.073	2.20E-02

Q10469	lpha-1,6-mannosyl-glycoprotein 2-beta-N-acetylglucosaminyltransfera	51.517	8.76	8.9069287	3.5794183	1	1	1	96.5	103.5	1.073	
Q12872	Splicing factor, suppressor of white-apricot homolog	104.758	8.05	8.2170917	2.7339642	3	3	3	96.5	103.5	1.073	2.48E-01
Q13151	Heterogeneous nuclear ribonucleoprotein A0	30.822	9.29	92.139621	38.032787	9	20	7	96.5	103.5	1.073	2.31E-01
Q8NCW5	NAD(P)H-hydrate epimerase	31.654	7.66	44.498401	15.625	3	6	3	96.5	103.5	1.073	1.03E-03
Q92685	Dol-P-Man:Man(5)GlcNAc(2)-PP-Dol alpha-1,3-mannosyltransferase	50.094	9.44	9.0594835	2.739726	1	1	1	96.5	103.5	1.073	
Q96EH3	Mitochondrial assembly of ribosomal large subunit protein 1	26.153	5.49	3.5023794	2.991453	1	1	1	96.5	103.5	1.073	
Q9H8H2	Probable ATP-dependent RNA helicase DDX31	94.029	9.99	24.381916	4.7003525	4	7	4	96.5	103.5	1.073	7.39E-02
Q9HC52	Chromobox protein homolog 8	43.369	9.91	24.506914	10.539846	4	5	4	96.5	103.5	1.073	1.09E-01
Q9HCS7	Pre-mRNA-splicing factor SYF1	99.946	6.23	73.230095	11.578947	8	13	8	96.5	103.5	1.073	1.81E-02
Q9NZD2	Glycolipid transfer protein	23.834	7.39	4.0910863	4.3062201	1	1	1	96.5	103.5	1.073	
Q9UKF6	Cleavage and polyadenylation specificity factor subunit 3	77.436	5.6	15.979602	5.2631579	3	3	3	96.5	103.5	1.073	
A9UHW6	MIF4G domain-containing protein	25.407	5.33	25.285903	17.117117	3	4	3	96.4	103.6	1.075	3.06E-02
O15014	Zinc finger protein 609	151.098	8.03	3.5644741	0.5669738	1	1	1	96.4	103.6	1.075	
O43805	Sjoegren syndrome nuclear autoantigen 1	13.588	5.38	3.4802749	10.92437	2	3	2	96.4	103.6	1.075	8.04E-01
P0CW22	40S ribosomal protein S17-like	15.54	9.85	78.66804	61.481481	7	16	7	96.4	103.6	1.075	5.20E-02
P23219	Prostaglandin G/H synthase 1	68.642	7.23	20.479832	7.1786311	6	9	6	96.4	103.6	1.075	8.34E-01
P36956	Sterol regulatory element-binding protein 1	121.599	8.13	3.650334	0.9590235	1	1	1	96.4	103.6	1.075	
P39019	40S ribosomal protein S19	16.051	10.32	104.56838	50.344828	11	36	11	96.4	103.6	1.075	1.51E-02
P60983	Glia maturation factor beta	16.702	5.29	39.153356	33.098592	4	5	3	96.4	103.6	1.075	7.84E-02
Q01813	ATP-dependent 6-phosphofructokinase, platelet type	85.542	7.55	281.64502	35.076531	23	57	20	96.4	103.6	1.075	2.29E-01
Q02447	Transcription factor Sp3	81.876	5.26	2.9136402	1.1523688	1	1	1	96.4	103.6	1.075	
Q13131	5'-AMP-activated protein kinase catalytic subunit alpha-1	63.969	8.12	34.05529	9.3023256	4	5	4	96.4	103.6	1.075	1.56E-02
Q14118	Dystroglycan	97.381	8.56	20.59242	4.5810056	3	4	3	96.4	103.6	1.075	
Q5F1R6	DnaJ homolog subfamily C member 21	61.989	5.47	84.560264	18.079096	9	13	9	96.4	103.6	1.075	5.48E-04
Q5JTJ3	Cytochrome c oxidase assembly factor 6 homolog	14.107	8.25	33.298166	21.6	2	5	2	96.4	103.6	1.075	3.20E-02
Q6DKJ4	Nucleoredoxin	48.362	4.97	45.727361	12.413793	3	6	3	96.4	103.6	1.075	3.22E-02
Q6PIW4	Fidgetin-like protein 1	74.03	7.85	13.140683	3.115727	2	3	1	96.4	103.6	1.075	
Q6UN15	Pre-mRNA 3'-end-processing factor FIP1	66.487	5.59	14.380146	5.5555556	3	4	3	96.4	103.6	1.075	3.10E-01
Q86YP4	Transcriptional repressor p66-alpha	68.021	9.94	23.523348	9.9526066	5	5	5	96.4	103.6	1.075	1.15E-03
Q8IVS2	Malonyl-CoA-acyl carrier protein transacylase, mitochondrial	42.934	8.72	9.5136917	3.5897436	1	2	1	96.4	103.6	1.075	
Q8IWA0	WD repeat-containing protein 75	94.438	5.96	28.427376	6.746988	5	5	5	96.4	103.6	1.075	1.11E-03
Q8IZ21	Phosphatase and actin regulator 4	78.163	6.62	2.6786087	1.4245014	1	1	1	96.4	103.6	1.075	
Q8N0Z6	Tetratricopeptide repeat protein 5	48.897	6.48	14.42785	6.5909091	3	3	3	96.4	103.6	1.075	5.78E-01
Q8N567	Zinc finger CCHC domain-containing protein 9	30.457	9.17	3.2753146	3.6900369	1	1	1	96.4	103.6	1.075	
Q8N668	COMM domain-containing protein 1	21.165	6.2	14.861817	16.842105	2	2	2	96.4	103.6	1.075	
Q8TF74	WAS/WASL-interacting protein family member 2	46.26	10.93	13.58047	6.3636364	2	3	2	96.4	103.6	1.075	
Q96G03	Phosphoglucomutase-2	68.24	6.73	42.021583	16.993464	8	10	8	96.4	103.6	1.075	1.40E-01
Q96H35	Probable RNA-binding protein 18	21.635	9.44	3.792635	4.7368421	1	1	1	96.4	103.6	1.075	
Q9BW85	Coiled-coil domain-containing protein 94	37.063	5.92	11.76533	4.0247678	1	2	1	96.4	103.6	1.075	
Q9BZJ0	Crooked neck-like protein 1	100.389	8	21.279546	5.3066038	4	5	4	96.4	103.6	1.075	3.16E-03
Q9C0B5	Palmitoyltransferase ZDHHC5	77.496	9.01	9.2873503	2.2377622	1	1	1	96.4	103.6	1.075	
Q9H3H1	tRNA dimethylallyltransferase, mitochondrial	52.692	8.21	8.9080052	3.640257	2	2	2	96.4	103.6	1.075	
Q9NR09	Baculoviral IAP repeat-containing protein 6	529.919	6.05	29.063175	1.7088738	6	6	6	96.4	103.6	1.075	2.59E-02
Q9NSV4	Protein diaphanous homolog 3	136.839	7.03	25.454216	3.9396479	5	6	5	96.4	103.6	1.075	1.01E-04
Q9UHB6	LIM domain and actin-binding protein 1	85.173	6.84	90.071595	16.073781	12	20	12	96.4	103.6	1.075	3.09E-03
Q9UJV9	Probable ATP-dependent RNA helicase DDX41	69.793	6.84	14.158602	6.7524116	4	4	4	96.4	103.6	1.075	
Q9UJX3	Anaphase-promoting complex subunit 7	66.813	5.64	29.790697	8.1803005	4	5	4	96.4	103.6	1.075	3.00E-04

Q9Y5A7	NEDD8 ultimate buster 1	70.494	5.96	38.118024	5.3658537	3	7	3	96.4	103.6	1.075	5.72E-03
Q9Y6V7	Probable ATP-dependent RNA helicase DDX49	54.192	9.06	9.944226	4.5548654	2	2	2	96.4	103.6	1.075	
O15321	Transmembrane 9 superfamily member 1	68.816	7.17	14.506051	3.7953795	2	2	2	96.3	103.7	1.077	
P17706	Tyrosine-protein phosphatase non-receptor type 2	48.442	8.29	13.605321	6.746988	2	2	2	96.3	103.7	1.077	
P42677	40S ribosomal protein S27	9.455	9.45	28.244369	38.095238	3	7	3	96.3	103.7	1.077	
P52788	Spermine synthase	41.242	5.02	42.512366	18.306011	6	11	6	96.3	103.7	1.077	5.61E-01
Q13177	Serine/threonine-protein kinase PAK 2	58.006	5.96	112.0681	32.824427	12	22	12	96.3	103.7	1.077	1.07E-01
Q15545	Transcription initiation factor TFIID subunit 7	40.234	5.2	7.1253876	6.017192	2	2	2	96.3	103.7	1.077	2.79E-01
Q6PGN9	Proline/serine-rich coiled-coil protein 1	38.773	11.21	4.8884015	3.8567493	1	1	1	96.3	103.7	1.077	
Q8WXW3	Progesterone-induced-blocking factor 1	89.75	6.02	14.284653	4.0951123	3	3	3	96.3	103.7	1.077	
Q92600	Cell differentiation protein RCD1 homolog	33.61	8.03	46.010247	19.732441	5	6	5	96.3	103.7	1.077	3.29E-02
Q96EN8	Molybdenum cofactor sulfurase	98.058	6.7	13.141404	4.5045045	4	4	4	96.3	103.7	1.077	
Q96HR8	H/ACA ribonucleoprotein complex non-core subunit NAF1	53.683	4.87	7.0959881	2.0242915	1	1	1	96.3	103.7	1.077	
Q9BYG3	MKI67 FHA domain-interacting nucleolar phosphoprotein	34.201	9.88	52.446716	28.327645	6	9	6	96.3	103.7	1.077	1.43E-01
Q9P0L2	Serine/threonine-protein kinase MARK1	88.947	9.36	6.8335699	1.1320755	1	1	1	96.3	103.7	1.077	
Q9UHR4	rain-specific angiogenesis inhibitor 1-associated protein 2-like protein	56.847	8.68	45.185059	15.068493	8	11	8	96.3	103.7	1.077	1.22E-03
Q9Y4B5	Microtubule cross-linking factor 1	209.397	6.43	15.27503	2.519685	4	4	4	96.3	103.7	1.077	2.34E-03
Q9Y608	Leucine-rich repeat flightless-interacting protein 2	82.121	6.95	41.345376	6.3800277	3	10	2	96.3	103.7	1.077	
O00214	Galectin-8	35.786	8.25	10.890084	4.1009464	1	1	1	96.2	103.8	1.079	
O14757	Serine/threonine-protein kinase Chk1	54.399	8.25	47.051535	14.495798	6	7	6	96.2	103.8	1.079	3.90E-03
O14965	Aurora kinase A	45.781	9.39	40.542177	17.866005	6	10	6	96.2	103.8	1.079	1.25E-03
P08183	Multidrug resistance protein 1	141.389	9	6.060321	1.40625	2	2	2	96.2	103.8	1.079	
P22670	MHC class II regulatory factor RFX1	104.693	6.29	20.506362	3.3707865	3	4	3	96.2	103.8	1.079	6.42E-02
P26358	DNA (cytosine-5)-methyltransferase 1	183.05	7.75	123.1124	14.60396	22	27	22	96.2	103.8	1.079	2.56E-06
P42166	Lamina-associated polypeptide 2, isoform alpha	75.446	7.66	244.15251	35.73487	21	46	13	96.2	103.8	1.079	6.47E-04
P47813	Eukaryotic translation initiation factor 1A, X-chromosomal	16.45	5.24	63.649922	29.166667	5	17	5	96.2	103.8	1.079	1.40E-01
Q15269	Periodic tryptophan protein 2 homolog	102.387	6.15	54.157635	8.8139282	7	13	7	96.2	103.8	1.079	3.33E-03
Q5BKZ1	DBIRD complex subunit ZNF326	65.613	5.15	58.234395	15.463918	8	10	8	96.2	103.8	1.079	1.16E-05
Q8IX90	Spindle and kinetochore-associated protein 3	46.33	5.11	7.9222688	2.6699029	1	1	1	96.2	103.8	1.079	
Q8IY22	C-Maf-inducing protein	86.275	6.7	14.514227	3.4928849	3	3	3	96.2	103.8	1.079	
Q8N6T3	ADP-ribosylation factor GTPase-activating protein 1	44.64	5.66	61.108282	23.152709	7	10	7	96.2	103.8	1.079	4.99E-02
Q92804	TATA-binding protein-associated factor 2N	61.793	8.02	101.38697	36.317568	12	23	10	96.2	103.8	1.079	5.49E-04
Q92995	Ubiquitin carboxyl-terminal hydrolase 13	97.265	5.53	25.403868	3.4762457	3	5	3	96.2	103.8	1.079	
Q96FX7	℞NA (adenine(58)-N(1))-methyltransferase catalytic subunit TRMT61.	31.362	7.36	9.3046879	7.266436	2	2	2	96.2	103.8	1.079	
Q9BUF5	Tubulin beta-6 chain	49.825	4.88	157.51496	31.61435	11	54	7	96.2	103.8	1.079	1.24E-01
Q9BW61	DET1- and DDB1-associated protein 1	11.828	8.68	4.9538952	11.764706	1	1	1	96.2	103.8	1.079	
Q9H0U3	Magnesium transporter protein 1	38.011	9.63	27.891613	10.149254	3	4	3	96.2	103.8	1.079	1.23E-01
Q9HA64	Ketosamine-3-kinase	34.39	7.33	63.549991	26.537217	8	13	8	96.2	103.8	1.079	3.11E-01
Q9UDY4	DnaJ homolog subfamily B member 4	37.783	8.5	87.733969	27.893175	7	11	6	96.2	103.8	1.079	5.06E-03
Q9ULK4	Mediator of RNA polymerase II transcription subunit 23	156.372	7.4	4.1547779	0.6578947	1	1	1	96.2	103.8	1.079	
Q9Y244	Proteasome maturation protein	15.779	5.11	5.187857	12.056738	2	2	2	96.2	103.8	1.079	
O94768	Serine/threonine-protein kinase 17B	42.317	5.25	2.6647427	2.4193548	1	1	1	96.1	103.9	1.081	
O95470	Sphingosine-1-phosphate lyase 1	63.483	9.16	25.808781	9.5070423	5	6	5	96.1	103.9	1.081	7.54E-03
O96008	Mitochondrial import receptor subunit TOM40 homolog	37.869	7.25	88.921929	24.376731	7	21	7	96.1	103.9	1.081	4.13E-02
P05455	Lupus La protein	46.808	7.12	139.18568	40.196078	18	31	18	96.1	103.9	1.081	7.24E-03
P09429	High mobility group protein B1	24.878	5.74	53.652314	30.232558	7	11	6	96.1	103.9	1.081	1.37E-02
P13716	Delta-aminolevulinic acid dehydratase	36.271	6.79	6.191249	4.5454545	1	1	1	96.1	103.9	1.081	

Q14674	Separin	233.028	7.55	13.7891	1.1320755	2	2	2	96.1	103.9	1.081	
Q2NL82	Pre-rRNA-processing protein TSR1 homolog	91.752	7.42	59.50023	11.691542	8	13	8	96.1	103.9	1.081	3.66E-01
Q6NYC1	Bifunctional arginine demethylase and lysyl-hydroxylase JMJD6	46.433	8.69	11.775679	4.9627792	2	2	2	96.1	103.9	1.081	
Q6P158	Putative ATP-dependent RNA helicase DHX57	155.507	7.71	18.128187	3.3189033	4	4	3	96.1	103.9	1.081	
Q8IY18	Structural maintenance of chromosomes protein 5	128.726	8.38	2.7933722	1.0899183	2	2	2	96.1	103.9	1.081	
Q93050	V-type proton ATPase 116 kDa subunit a isoform 1	96.35	6.43	46.657443	11.35006	8	10	8	96.1	103.9	1.081	4.55E-03
Q96EB1	Elongator complex protein 4	46.558	8.51	16.190746	6.6037736	2	2	2	96.1	103.9	1.081	4.90E-02
Q9BTV4	Transmembrane protein 43	44.847	8.13	27.435123	11.75	5	7	5	96.1	103.9	1.081	4.17E-01
Q9BVC6	Transmembrane protein 109	26.194	10.48	37.575808	12.757202	4	9	4	96.1	103.9	1.081	4.09E-02
Q9H6E5	Speckle targeted PIP5K1A-regulated poly(A) polymerase	93.788	6.16	14.432328	3.7757437	3	3	3	96.1	103.9	1.081	
Q9HCD5	Nuclear receptor coactivator 5	65.496	9.6	10.02698	5.3540587	3	3	3	96.1	103.9	1.081	
Q9NRN7	ninoadipate-semialdehyde dehydrogenase-phosphopantetheinyl transfe	35.753	6.8	16.695928	11.650485	3	3	3	96.1	103.9	1.081	1.85E-02
Q9UL63	Muskelin	84.713	6.34	37.943833	8.4353741	6	8	6	96.1	103.9	1.081	1.86E-03
Q9ULX3	RNA-binding protein NOB1	46.646	7.18	35.83543	23.543689	8	8	8	96.1	103.9	1.081	7.44E-03
Q9UPR0	Inactive phospholipase C-like protein 2	125.785	6.9	15.534541	3.8154392	3	3	3	96.1	103.9	1.081	
O43896	Kinesin-like protein KIF1C	122.87	6.9	44.525874	6.2556664	6	8	3	96	104	1.083	1.80E-01
O95628	CCR4-NOT transcription complex subunit 4	63.47	7.03	6.4131022	3.6521739	2	2	2	96	104	1.083	
P53611	Geranylgeranyl transferase type-2 subunit beta	36.9	5.03	21.499109	16.012085	6	6	6	96	104	1.083	9.87E-02
P60866	40S ribosomal protein S20	13.364	9.94	42.310499	30.252101	4	16	4	96	104	1.083	8.44E-02
P61254	60S ribosomal protein L26	17.248	10.55	10.858612	17.931034	3	4	3	96	104	1.083	2.90E-01
Q08AM6	Protein VAC14 homolog	87.917	6.13	22.490384	6.5217391	4	5	4	96	104	1.083	4.64E-02
Q13144	Translation initiation factor eIF-2B subunit epsilon	80.329	5.08	38.514035	9.1539528	5	7	5	96	104	1.083	7.78E-02
Q14197	Peptidyl-tRNA hydrolase ICT1, mitochondrial	23.615	10.07	11.222932	9.223301	2	3	2	96	104	1.083	
Q16342	Programmed cell death protein 2	38.567	5.38	5.8455657	6.1046512	2	2	2	96	104	1.083	
Q86WR7	Proline and serine-rich protein 2	45.774	7.2	16.038625	8.9655172	3	3	3	96	104	1.083	
Q8NDT2	Putative RNA-binding protein 15B	97.147	9.85	11.372085	3.1460674	2	2	2	96	104	1.083	
Q92610	Zinc finger protein 592	137.441	7.84	6.3082998	1.1049724	1	1	1	96	104	1.083	
Q96D71	RalBP1-associated Eps domain-containing protein 1	86.609	5.69	15.833136	3.6432161	3	3	3	96	104	1.083	
Q9BQP7	Mitochondrial genome maintenance exonuclease 1	39.396	7.68	6.6891177	5.2325581	2	2	2	96	104	1.083	
Q9HCE5	N6-adenosine-methyltransferase subunit METTL14	52.118	6.21	12.198132	4.3859649	2	2	2	96	104	1.083	
Q9NQT4	Exosome complex component RRP46	25.233	7.59	25.089049	20.851064	3	3	3	96	104	1.083	1.80E-01
Q9ULW0	Targeting protein for Xklp2	85.6	9.23	83.540176	18.875502	12	15	12	96	104	1.083	2.09E-02
E9PAV3	scent polypeptide-associated complex subunit alpha, muscle-specific fo	205.295	9.58	89.030159	3.705486	6	27	6	95.9	104.1	1.086	4.06E-05
O75251	NADH dehydrogenase [ubiquinone] iron-sulfur protein 7, mitochondria	23.548	9.99	10.13514	10.798122	2	2	2	95.9	104.1	1.086	
P08236	Beta-glucuronidase	74.685	7.02	5.1599567	1.3824885	1	1	1	95.9	104.1	1.086	
P09651	Heterogeneous nuclear ribonucleoprotein A1	38.723	9.13	372.20631	48.11828	20	143	6	95.9	104.1	1.086	3.67E-03
P49006	MARCKS-related protein	19.517	4.67	30.949395	14.358974	2	6	2	95.9	104.1	1.086	1.23E-01
Q00534	Cyclin-dependent kinase 6	36.915	6.46	82.335848	30.368098	9	20	8	95.9	104.1	1.086	6.93E-04
Q6P1K2	Polyamine-modulated factor 1	23.325	5.49	20.53427	7.804878	1	2	1	95.9	104.1	1.086	
Q8WTT2	Nucleolar complex protein 3 homolog	92.49	9.17	54.968569	8.25	5	8	5	95.9	104.1	1.086	3.30E-03
Q96IW7	Vesicle-trafficking protein SEC22a	34.925	8.24	4.4837291	3.5830619	1	1	1	95.9	104.1	1.086	
Q96PK6	RNA-binding protein 14	69.449	9.67	86.714478	19.431988	13	24	13	95.9	104.1	1.086	7.37E-02
Q9BZX2	Uridine-cytidine kinase 2	29.281	6.7	39.043928	28.735632	6	8	6	95.9	104.1	1.086	9.01E-06
Q9H9J4	Ubiquitin carboxyl-terminal hydrolase 42	145.302	8.63	6.2322506	1.1329305	1	1	1	95.9	104.1	1.086	
Q9NP77	RNA polymerase II subunit A C-terminal domain phosphatase SSU72	22.56	5.33	25.729592	18.041237	3	5	3	95.9	104.1	1.086	9.02E-03
Q9NW81	ATP synthase subunit s-like protein	29.249	6.43	5.9969568	7.7821012	2	2	2	95.9	104.1	1.086	
Q9NZ52	ADP-ribosylation factor-binding protein GGA3	78.267	5.58	14.356692	4.2876902	3	3	2	95.9	104.1	1.086	

Q9NZJ9	Diphosphoinositol polyphosphate phosphohydrolase 2	20.293	6.35	8.0660603	10.555556	2	2	2	95.9	104.1	1.086	
Q9ULT8	E3 ubiquitin-protein ligase HECTD1	289.203	5.35	74.175244	6.091954	13	13	13	95.9	104.1	1.086	1.04E-03
O00151	PDZ and LIM domain protein 1	36.049	7.02	111.97284	55.319149	15	25	15	95.8	104.2	1.088	2.34E-04
O60784	Target of Myb protein 1	53.785	4.7	30.106924	9.3495935	4	5	4	95.8	104.2	1.088	4.94E-03
O60885	Bromodomain-containing protein 4	152.124	9.19	43.726596	6.0939794	8	10	4	95.8	104.2	1.088	6.78E-02
O75330	Hyaluronan mediated motility receptor	84.049	5.83	28.347382	5.801105	4	5	4	95.8	104.2	1.088	1.06E-02
O76024	Wolframin	100.227	8.05	12.476506	3.7078652	3	3	3	95.8	104.2	1.088	8.76E-01
P49959	Double-strand break repair protein MRE11A	80.543	5.9	39.82252	13.276836	9	9	9	95.8	104.2	1.088	6.78E-04
Q03468	DNA excision repair protein ERCC-6	168.311	8.09	33.808772	5.2243804	7	7	7	95.8	104.2	1.088	5.21E-02
Q12913	Receptor-type tyrosine-protein phosphatase eta	145.851	5.58	3.7064165	0.5983545	1	1	1	95.8	104.2	1.088	
Q8WWC4	Uncharacterized protein C2orf47, mitochondrial	32.524	9.17	13.809177	5.8419244	2	4	2	95.8	104.2	1.088	7.40E-01
Q96QD8	Sodium-coupled neutral amino acid transporter 2	55.99	8	13.512155	5.5335968	2	2	2	95.8	104.2	1.088	
Q9H501	ESF1 homolog	98.735	5.11	4.0769633	1.1750881	1	1	1	95.8	104.2	1.088	
Q9Y2R5	28S ribosomal protein S17, mitochondrial	14.493	9.85	31.051148	44.615385	4	5	4	95.8	104.2	1.088	3.04E-02
Q9Y4C2	TRPM8 channel-associated factor 1	102.061	6.54	47.17128	9.0119435	8	11	8	95.8	104.2	1.088	1.13E-03
O14828	Secretory carrier-associated membrane protein 3	38.262	7.64	48.314936	26.512968	6	8	6	95.7	104.3	1.09	1.42E-01
O75506	Heat shock factor-binding protein 1	8.538	4.36	35.84359	56.578947	3	6	3	95.7	104.3	1.09	2.21E-01
O95999	B-cell lymphoma/leukemia 10	26.235	5.74	6.26946	4.2918455	1	1	1	95.7	104.3	1.09	
P52756	RNA-binding protein 5	92.097	6.28	32.980961	5.7668712	4	5	3	95.7	104.3	1.09	7.72E-03
P55327	Tumor protein D52	24.312	4.83	77.786558	41.964286	8	17	8	95.7	104.3	1.09	6.79E-04
P59780	AP-3 complex subunit sigma-2	22.003	5.22	18.883036	10.880829	2	3	2	95.7	104.3	1.09	1.85E-01
P61011	Signal recognition particle 54 kDa protein	55.668	8.75	125.99768	26.388889	12	24	12	95.7	104.3	1.09	2.22E-02
P62861	40S ribosomal protein S30	6.644	12.15	7.0750546	16.949153	1	2	1	95.7	104.3	1.09	
Q13501	Sequestosome-1	47.657	5.22	129.85473	40.909091	12	23	12	95.7	104.3	1.09	1.60E-01
Q14728	Major facilitator superfamily domain-containing protein 10	48.308	9.6	11.044102	2.8571429	1	2	1	95.7	104.3	1.09	
Q76FK4	Nucleolar protein 8	131.535	7.09	9.2869435	2.3993145	3	3	3	95.7	104.3	1.09	4.17E-02
Q8NEF9	Serum response factor-binding protein 1	48.604	9.58	6.9589127	3.962704	2	2	2	95.7	104.3	1.09	
Q96ER9	Coiled-coil domain-containing protein 51	45.783	8.19	20.814117	12.16545	5	6	5	95.7	104.3	1.09	2.80E-03
Q96ME1	F-box/LRR-repeat protein 18	88.284	8.32	25.510743	3.8509317	2	3	2	95.7	104.3	1.09	
Q99661	Kinesin-like protein KIF2C	81.261	7.83	75.984112	18.344828	12	13	11	95.7	104.3	1.09	7.22E-07
Q99685	Monoglyceride lipase	33.24	6.99	11.437812	4.620462	1	2	1	95.7	104.3	1.09	
Q99986	Serine/threonine-protein kinase VRK1	45.447	8.91	14.290467	8.0808081	3	3	3	95.7	104.3	1.09	
Q9BZE1	39S ribosomal protein L37, mitochondrial	48.087	8.59	33.173227	14.893617	5	7	5	95.7	104.3	1.09	2.78E-03
Q9H9T3	Elongator complex protein 3	62.219	8.88	33.78154	11.882998	6	7	6	95.7	104.3	1.09	6.14E-04
O15294	acetylglucosamine--peptide N-acetylglucosaminyltransferase 110 kDa	116.85	6.7	50.871429	8.3173996	8	10	8	95.6	104.4	1.092	6.60E-05
P33764	Protein S100-A3	11.705	4.78	4.3504704	10.891089	1	1	1	95.6	104.4	1.092	
P51116	Fragile X mental retardation syndrome-related protein 2	74.178	6.23	98.511359	16.939079	8	14	5	95.6	104.4	1.092	3.73E-02
Q07864	DNA polymerase epsilon catalytic subunit A	261.351	6.39	4.204678	0.6124234	2	2	2	95.6	104.4	1.092	3.05E-02
Q13505	Metaxin-1	51.445	9.79	17.552625	6.6523605	2	2	2	95.6	104.4	1.092	6.92E-03
Q14244	Ensconsin	84.002	9.61	30.110239	8.9452603	7	9	7	95.6	104.4	1.092	5.75E-03
Q69YN4	Protein virilizer homolog	201.898	5.01	16.781769	2.4282561	4	4	4	95.6	104.4	1.092	1.02E-04
Q6EEV4	DNA-directed RNA polymerase II subunit GRINL1A, isoforms 4/5	15.122	8.37	15.941574	16.216216	1	1	1	95.6	104.4	1.092	
Q92966	snRNA-activating protein complex subunit 3	46.723	5.27	3.3791355	2.676399	1	1	1	95.6	104.4	1.092	
Q9H3R2	Mucin-13	54.569	5.07	110.98037	23.828125	8	15	8	95.6	104.4	1.092	1.61E-03
Q9NQW6	Actin-binding protein anillin	124.122	8.07	80.111056	12.366548	13	17	13	95.6	104.4	1.092	4.60E-03
Q9NWT1	p21-activated protein kinase-interacting protein 1	43.936	8.91	14.501591	10.459184	4	4	4	95.6	104.4	1.092	8.77E-01
Q9NXW2	DnaJ homolog subfamily B member 12	41.793	8.53	17.14953	8.2666667	3	4	3	95.6	104.4	1.092	6.83E-02

Q9NZJ7	Mitochondrial carrier homolog 1	41.517	9.32	10.592851	6.940874	2	3	2	95.6	104.4	1.092	
Q9UBF6	RING-box protein 2	12.675	5.44	5.2306227	10.619469	1	1	1	95.6	104.4	1.092	
Q9UKD2	mRNA turnover protein 4 homolog	27.543	8.29	50.122612	26.359833	6	8	6	95.6	104.4	1.092	3.74E-01
O15446	DNA-directed RNA polymerase I subunit RPA34	54.951	8.51	11.232206	4.3137255	2	2	2	95.5	104.5	1.094	2.14E-01
O43148	mRNA cap guanine-N7 methyltransferase	54.809	6.61	29.685287	13.865546	6	7	5	95.5	104.5	1.094	1.18E-02
O75909	Cyclin-K	64.199	8.41	30.595173	10	5	7	5	95.5	104.5	1.094	1.51E-03
O95478	Ribosome biogenesis protein NSA2 homolog	30.047	10.27	18.581284	11.153846	3	4	3	95.5	104.5	1.094	1.31E-01
P45985	Dual specificity mitogen-activated protein kinase kinase 4	44.259	8.07	5.3449577	2.5062657	1	1	1	95.5	104.5	1.094	
P48509	CD151 antigen	28.276	7.47	7.9081044	10.671937	3	5	3	95.5	104.5	1.094	9.79E-02
P48729	Casein kinase I isoform alpha	38.89	9.57	50.220861	20.178042	6	9	6	95.5	104.5	1.094	2.74E-02
P62081	40S ribosomal protein S7	22.113	10.1	53.74619	31.443299	8	14	8	95.5	104.5	1.094	3.24E-03
Q13530	Serine incorporator 3	52.546	7.46	5.5184116	1.4799154	1	2	1	95.5	104.5	1.094	
Q14671	Pumilio homolog 1	126.395	6.84	45.299013	6.4080944	6	9	6	95.5	104.5	1.094	4.45E-03
Q15742	NGFI-A-binding protein 2	56.559	6.96	8.9212015	3.2380952	2	2	2	95.5	104.5	1.094	2.49E-02
Q96GA3	Protein LTV1 homolog	54.821	4.91	9.4860455	5.0526316	2	2	2	95.5	104.5	1.094	
Q99755	Phosphatidylinositol 4-phosphate 5-kinase type-1 alpha	62.594	8.21	8.4112528	3.5587189	2	2	2	95.5	104.5	1.094	4.71E-02
Q99836	Myeloid differentiation primary response protein MyD88	33.212	6.15	23.696087	16.216216	3	3	3	95.5	104.5	1.094	9.86E-04
Q9H0U4	Ras-related protein Rab-1B	22.157	5.73	130.94302	67.164179	13	42	4	95.5	104.5	1.094	1.31E-01
Q9H0U9	Testis-specific Y-encoded-like protein 1	49.162	5.45	8.265861	3.6613272	2	2	2	95.5	104.5	1.094	
Q9H9P8	L-2-hydroxyglutarate dehydrogenase, mitochondrial	50.284	8.15	17.591354	8.6393089	3	3	3	95.5	104.5	1.094	1.66E-01
Q9NUK0	Muscleblind-like protein 3	38.506	8.81	5.8674681	2.5423729	1	2	1	95.5	104.5	1.094	
Q9Y570	Protein phosphatase methylesterase 1	42.288	5.97	94.947592	27.720207	10	21	10	95.5	104.5	1.094	2.14E-04
P17544	Cyclic AMP-dependent transcription factor ATF-7	52.934	8.65	14.531733	8.2995951	3	4	3	95.4	104.6	1.096	5.68E-02
P18077	60S ribosomal protein L35a	12.53	11.06	25.434633	40	5	11	5	95.4	104.6	1.096	3.21E-02
P50238	Cysteine-rich protein 1	8.527	8.75	14.306218	31.168831	3	7	3	95.4	104.6	1.096	1.73E-01
P55081	Microfibrillar-associated protein 1	51.927	4.98	8.3143447	6.3781321	3	4	3	95.4	104.6	1.096	2.14E-01
P62701	40S ribosomal protein S4, X isoform	29.579	10.15	140.00779	46.768061	13	36	13	95.4	104.6	1.096	6.61E-05
P83111	Serine beta-lactamase-like protein LACTB, mitochondrial	60.655	8.53	9.2837022	1.6453382	1	2	1	95.4	104.6	1.096	
Q15562	Transcriptional enhancer factor TEF-4	49.212	6.47	3.8804142	1.7897092	1	1	1	95.4	104.6	1.096	
Q5EBL8	PDZ domain-containing protein 11	16.121	7.21	18.27786	17.142857	1	1	1	95.4	104.6	1.096	
Q86UD0	Suppressor APC domain-containing protein 2	42.61	8.82	10.115361	6.0913706	2	2	2	95.4	104.6	1.096	2.36E-04
Q96GD4	Aurora kinase B	39.286	9.29	10.174349	5.8139535	2	3	2	95.4	104.6	1.096	4.50E-01
Q9NVS2	28S ribosomal protein S18a, mitochondrial	22.17	10.33	19.365842	21.428571	4	5	4	95.4	104.6	1.096	1.41E-01
Q9NX70	Mediator of RNA polymerase II transcription subunit 29	21.06	6.29	8.3901922	7	1	1	1	95.4	104.6	1.096	
Q9NXE4	Sphingomyelin phosphodiesterase 4	93.292	7.97	26.297903	6.8923821	4	5	4	95.4	104.6	1.096	2.12E-03
Q9UBV2	Protein sel-1 homolog 1	88.698	5.39	5.8164455	1.2594458	1	1	1	95.4	104.6	1.096	
Q9Y291	28S ribosomal protein S33, mitochondrial	12.621	10.11	5.4649592	8.490566	1	1	1	95.4	104.6	1.096	
Q9Y2Y0	ADP-ribosylation factor-like protein 2-binding protein	18.81	4.35	6.3136319	7.3619632	1	1	1	95.4	104.6	1.096	
O14925	Mitochondrial import inner membrane translocase subunit Tim23	21.929	8.6	28.893678	16.746411	3	4	3	95.3	104.7	1.099	
O15514	DNA-directed RNA polymerase II subunit RPB4	16.301	4.79	10.393496	16.197183	2	2	2	95.3	104.7	1.099	1.94E-02
P06753	Tropomyosin alpha-3 chain	32.93	4.72	142.86768	35.438596	17	67	7	95.3	104.7	1.099	1.20E-01
P18858	DNA ligase 1	101.673	5.62	22.50524	5.4406964	4	4	4	95.3	104.7	1.099	2.80E-03
P30825	High affinity cationic amino acid transporter 1	67.594	5.43	9.3173235	4.9284579	3	3	3	95.3	104.7	1.099	2.93E-02
P46013	Antigen KI-67	358.474	9.45	407.54369	22.297297	54	71	54	95.3	104.7	1.099	4.12E-11
P51003	Poly(A) polymerase alpha	82.791	7.37	30.455609	11.409396	7	7	7	95.3	104.7	1.099	2.23E-01
Q13190	Syntaxin-5	39.648	9.16	35.947077	12.676056	4	5	4	95.3	104.7	1.099	9.66E-05
Q5TBB1	Ribonuclease H2 subunit B	35.116	9.13	17.068958	11.858974	4	4	4	95.3	104.7	1.099	1.07E-01

Q6PL18	ATPase family AAA domain-containing protein 2	158.456	6.32	56.13656	7.4100719	9	11	9	95.3	104.7	1.099	6.81E-03
Q7L576	Cytoplasmic FMR1-interacting protein 1	145.089	6.9	76.709081	11.731844	14	19	14	95.3	104.7	1.099	3.58E-02
Q8IYU8	Calcium uptake protein 2, mitochondrial	49.634	9.09	11.074585	2.9953917	1	1	1	95.3	104.7	1.099	
Q96JJ7	Protein disulfide-isomerase TMX3	51.839	4.91	14.590124	9.030837	3	3	3	95.3	104.7	1.099	8.06E-02
Q9H490	Phosphatidylinositol glycan anchor biosynthesis class U protein	50.019	7.72	14.073242	4.8275862	2	3	2	95.3	104.7	1.099	1.22E-01
Q9H974	Queuine tRNA-ribosyltransferase subunit QTRTD1	46.683	6.81	41.197716	16.385542	5	6	5	95.3	104.7	1.099	1.52E-02
Q9NPI6	mRNA-decapping enzyme 1A	63.27	6.25	17.749112	4.467354	2	2	2	95.3	104.7	1.099	
Q9UNS1	Protein timeless homolog	138.572	5.4	15.829719	3.2284768	4	4	4	95.3	104.7	1.099	2.99E-02
O15504	Nucleoporin-like protein 2	44.844	9.19	7.8867253	3.3096927	1	1	1	95.2	104.8	1.101	
O43809	Cleavage and polyadenylation specificity factor subunit 5	26.211	8.82	68.143786	39.647577	10	15	10	95.2	104.8	1.101	2.80E-04
O60828	Polyglutamine-binding protein 1	30.454	6.33	23.219382	16.981132	4	6	4	95.2	104.8	1.101	4.11E-02
O95159	Zinc finger protein-like 1	34.092	8.07	14.978689	10.645161	3	3	3	95.2	104.8	1.101	9.25E-02
O95359	Transforming acidic coiled-coil-containing protein 2	309.237	4.79	15.699499	1.2550882	4	4	2	95.2	104.8	1.101	
P01116	GTPase KRas	21.642	6.77	31.742009	28.042328	4	5	2	95.2	104.8	1.101	1.66E-02
P61964	WD repeat-containing protein 5	36.565	8.27	31.734366	11.077844	3	5	3	95.2	104.8	1.101	2.77E-03
Q05397	Focal adhesion kinase 1	119.157	6.62	22.694167	4.9429658	5	5	5	95.2	104.8	1.101	
Q17RN3	Protein FAM98C	37.305	7.18	10.033717	4.2979943	1	1	1	95.2	104.8	1.101	
Q8N5F7	NF-kappa-B-activating protein	47.11	10.11	5.3373366	2.4096386	1	1	1	95.2	104.8	1.101	
Q96JB5	CDK5 regulatory subunit-associated protein 3	56.885	4.75	18.04346	8.6956522	5	8	4	95.2	104.8	1.101	4.10E-02
Q96KN3	Homeobox protein PKNOX2	51.995	4.88	4.3766473	1.6949153	1	2	1	95.2	104.8	1.101	
Q96Q45	Transmembrane protein 237	45.498	6.47	6.3792099	6.372549	2	2	2	95.2	104.8	1.101	
Q9H098	Protein FAM107B	15.548	8.29	20.124911	21.374046	3	5	3	95.2	104.8	1.101	5.39E-01
Q9H0A8	COMM domain-containing protein 4	21.75	7.31	20.490485	19.59799	3	3	3	95.2	104.8	1.101	5.35E-02
Q9H3L0	ethylmalonic aciduria and homocystinuria type D protein, mitochondri	32.919	5.29	20.434614	9.1216216	2	3	2	95.2	104.8	1.101	
Q9Y5T5	Ubiquitin carboxyl-terminal hydrolase 16	93.511	6.93	3.2251522	2.0656136	2	2	2	95.2	104.8	1.101	
O60814	Histone H2B type 1-K	13.882	10.32	98.736114	57.936508	9	99	4	95.1	104.9	1.103	2.02E-02
P49207	60S ribosomal protein L34	13.284	11.47	16.483534	29.059829	4	6	4	95.1	104.9	1.103	8.33E-03
P61024	Cyclin-dependent kinases regulatory subunit 1	9.654	8.94	12.649544	27.848101	2	3	2	95.1	104.9	1.103	
P62736	Actin, aortic smooth muscle	41.982	5.39	190.73163	28.381963	13	265	1	95.1	104.9	1.103	
Q15424	Scaffold attachment factor B1	102.58	5.47	108.71699	21.311475	22	52	10	95.1	104.9	1.103	2.18E-01
Q8N3V7	Synaptopodin	99.402	8.72	51.945791	7.3196986	4	7	4	95.1	104.9	1.103	3.48E-03
Q9BVP2	Guanine nucleotide-binding protein-like 3	61.954	9.16	68.713485	16.575592	7	11	7	95.1	104.9	1.103	1.73E-02
Q9BZC7	ATP-binding cassette sub-family A member 2	269.701	6.81	12.892667	1.2731006	2	2	2	95.1	104.9	1.103	
Q9NRG4	N-lysine methyltransferase SMYD2	49.656	6.71	9.5057062	4.6189376	1	1	1	95.1	104.9	1.103	
P62253	Ubiquitin-conjugating enzyme E2 G1	19.497	5.3	12.585524	7.0588235	1	2	1	95	105	1.105	
Q15906	Vacuolar protein sorting-associated protein 72 homolog	40.57	6.48	2.8010681	4.1208791	1	1	1	95	105	1.105	
Q86VU5	Catechol O-methyltransferase domain-containing protein 1	28.79	8.38	15.745097	19.847328	3	3	3	95	105	1.105	3.22E-04
Q9NX18	Succinate dehydrogenase assembly factor 2, mitochondrial	19.587	6.8	12.362826	19.277108	3	3	3	95	105	1.105	7.99E-04
Q9NZM5	Glioma tumor suppressor candidate region gene 2 protein	54.356	10.32	32.439939	14.435146	7	7	7	95	105	1.105	7.38E-01
Q9UJ41	Rab5 GDP/GTP exchange factor	79.321	6.81	27.826435	7.2033898	4	4	4	95	105	1.105	8.58E-02
Q9Y6A5	Transforming acidic coiled-coil-containing protein 3	90.304	5.05	19.40136	8.1145585	5	5	5	95	105	1.105	2.84E-02
O75376	Nuclear receptor corepressor 1	270.044	7.11	20.158691	1.2704918	3	3	1	94.9	105.1	1.107	
P17275	Transcription factor jun-B	35.857	9.22	8.5954098	6.6282421	2	2	2	94.9	105.1	1.107	
Q13595	Transformer-2 protein homolog alpha	32.669	11.27	28.606062	18.439716	5	8	3	94.9	105.1	1.107	1.72E-01
Q15833	Syntaxin-binding protein 2	66.41	6.55	67.695904	18.212479	9	11	9	94.9	105.1	1.107	1.85E-01
Q86YR5	G-protein-signaling modulator 1	74.464	6.54	12.899811	5.6296296	4	4	4	94.9	105.1	1.107	2.29E-01
Q96EY4	Translation machinery-associated protein 16	23.849	9.26	10.518557	9.8522167	1	1	1	94.9	105.1	1.107	

Q9H000	Probable E3 ubiquitin-protein ligase makorin-2	46.909	7.61	19.049831	12.019231	5	6	5	94.9	105.1	1.107	1.76E-03
A6NDU8	UPF0600 protein C5orf51	33.599	5.26	29.594251	10.884354	2	4	2	94.8	105.2	1.11	
P08047	Transcription factor Sp1	80.644	7.34	21.76178	6.1146497	3	4	3	94.8	105.2	1.11	4.75E-02
Q12841	Follistatin-related protein 1	34.963	5.52	17.302366	12.987013	4	5	4	94.8	105.2	1.11	2.61E-04
Q49AR2	UPF0489 protein C5orf22	49.935	4.78	6.7119745	2.0361991	1	1	1	94.8	105.2	1.11	
Q5J8M3	ER membrane protein complex subunit 4	20.073	8.62	2.485054	5.4644809	1	1	1	94.8	105.2	1.11	
Q8NBI6	Xyloside xylosyltransferase 1	43.778	8.13	9.4845646	5.5979644	2	2	2	94.8	105.2	1.11	
Q96CW1	AP-2 complex subunit mu	49.623	9.54	48.946806	16.551724	7	11	7	94.8	105.2	1.11	3.29E-04
Q96HY6	DDRGK domain-containing protein 1	35.589	5.12	25.046417	14.012739	3	4	3	94.8	105.2	1.11	
Q99700	Ataxin-2	140.196	9.57	57.140218	7.0068545	8	10	7	94.8	105.2	1.11	3.68E-05
Q9NX08	COMM domain-containing protein 8	21.077	5.43	13.82285	18.032787	3	3	2	94.8	105.2	1.11	
Q8N556	Actin filament-associated protein 1	80.675	8.68	12.090166	3.4246575	2	2	2	94.7	105.3	1.112	
Q96DV4	39S ribosomal protein L38, mitochondrial	44.568	7.53	55.655172	26.052632	7	10	7	94.7	105.3	1.112	5.62E-02
Q9UG56	Phosphatidylserine decarboxylase proenzyme	46.642	9.42	11.243822	6.3569682	3	3	3	94.7	105.3	1.112	1.73E-02
Q9Y2R9	28S ribosomal protein S7, mitochondrial	28.116	9.99	45.737126	24.793388	5	8	5	94.7	105.3	1.112	1.61E-02
O60934	Nibrin	84.906	6.9	27.049922	7.1618037	5	5	5	94.6	105.4	1.114	6.09E-02
P53816	HRAS-like suppressor 3	17.925	7.99	16.517343	16.049383	2	2	2	94.6	105.4	1.114	
Q02543	60S ribosomal protein L18a	20.749	10.71	46.503171	30.113636	5	9	5	94.6	105.4	1.114	1.02E-01
Q14814	Myocyte-specific enhancer factor 2D	55.903	7.88	8.1722049	6.5259117	2	2	2	94.6	105.4	1.114	
Q4KMQ1	Taperin	75.51	7.25	7.3267121	3.6568214	2	2	2	94.6	105.4	1.114	1.36E-02
Q6PCE3	Glucose 1,6-bisphosphate synthase	70.396	7.15	6.9484409	3.5369775	2	2	2	94.6	105.4	1.114	
Q96JY6	PDZ and LIM domain protein 2	37.436	8.72	17.400728	6.8181818	3	4	3	94.6	105.4	1.114	
Q9NQ29	Putative RNA-binding protein Luc7-like 1	43.701	9.92	56.271117	19.407008	7	15	3	94.6	105.4	1.114	8.83E-02
Q9UBL3	Set1/Ash2 histone methyltransferase complex subunit ASH2	68.679	5.69	23.070791	4.2993631	2	3	2	94.6	105.4	1.114	
Q9UQB8	Brain-specific angiogenesis inhibitor 1-associated protein 2	60.83	8.9	72.412079	18.84058	10	16	10	94.6	105.4	1.114	4.81E-01
O00622	Protein CYR61	41.998	8.21	3.6272721	2.3622047	1	1	1	94.5	105.5	1.116	
O43660	Pleiotropic regulator 1	57.158	9.17	49.765645	18.677043	8	11	8	94.5	105.5	1.116	5.47E-04
P49356	Protein farnesyltransferase subunit beta	48.742	5.82	15.938793	3.6613272	1	2	1	94.5	105.5	1.116	
Q5TDH0	Protein DDI1 homolog 2	44.495	5.05	52.614271	23.558897	7	8	7	94.5	105.5	1.116	2.70E-02
Q86XA9	HEAT repeat-containing protein 5A	221.863	6.58	22.96145	2.3529412	4	4	4	94.5	105.5	1.116	1.59E-04
Q96A73	Putative monooxygenase p33MONOX	33.226	9.39	4.0809742	3.2786885	1	1	1	94.5	105.5	1.116	
Q96G21	U3 small nucleolar ribonucleoprotein protein IMP4	33.736	9.47	6.6307841	4.8109966	1	1	1	94.5	105.5	1.116	
Q96ST2	Protein IWS1 homolog	91.899	4.69	41.232365	9.035409	6	9	6	94.5	105.5	1.116	8.34E-03
Q99962	Endophilin-A1	39.937	5.45	26.413994	14.772727	5	6	2	94.5	105.5	1.116	
Q9H0E2	Toll-interacting protein	30.262	5.97	6.8804142	5.1094891	1	1	1	94.5	105.5	1.116	
Q9NRG9	Aladin	59.536	7.5	20.697082	6.959707	3	3	3	94.5	105.5	1.116	2.88E-02
P55957	BH3-interacting domain death agonist	21.981	5.44	10.609552	20	2	2	2	94.4	105.6	1.119	
P78356	Phosphatidylinositol 5-phosphate 4-kinase type-2 beta	47.348	7.33	3.4509967	2.6442308	1	1	1	94.4	105.6	1.119	
Q14678	KN motif and ankyrin repeat domain-containing protein 1	147.197	5.3	24.658476	4.6597633	5	5	5	94.4	105.6	1.119	3.81E-02
Q86VZ5	Phosphatidylcholine:ceramide cholinephosphotransferase 1	49.175	8.4	9.6400727	2.1479714	1	2	1	94.4	105.6	1.119	
Q96QD5	DEP domain-containing protein 7	58.273	7.77	40.736503	17.02544	9	10	9	94.4	105.6	1.119	1.63E-04
Q9BYD2	39S ribosomal protein L9, mitochondrial	30.224	10.08	8.3667336	6.3670412	1	1	1	94.4	105.6	1.119	
Q9UNI6	Dual specificity protein phosphatase 12	37.663	6.84	25.093283	15.294118	4	5	4	94.4	105.6	1.119	3.46E-01
O00748	Cocaine esterase	61.767	6.1	5.6987529	2.1466905	1	1	1	94.3	105.7	1.121	
P82094	TATA element modulatory factor	122.767	4.92	6.735075	1.7383349	2	2	2	94.3	105.7	1.121	
Q13888	General transcription factor IIH subunit 2	44.39	6.76	13.190006	4.8101266	2	2	2	94.3	105.7	1.121	3.11E-01
Q30201	Hereditary hemochromatosis protein	40.082	6.6	6.9473061	4.0229885	1	1	1	94.3	105.7	1.121	

Q96FK6	WD repeat-containing protein 89	43.187	6.13	7.9523358	3.1007752	1	1	1	94.3	105.7	1.121	
Q99653	Calcineurin B homologous protein 1	22.442	5.1	3.4929541	6.1538462	1	1	1	94.3	105.7	1.121	
Q9BV44	THUMP domain-containing protein 3	56.967	6.37	16.761008	8.2840237	4	5	4	94.3	105.7	1.121	1.37E-01
Q9NRK6	ATP-binding cassette sub-family B member 10, mitochondrial	79.098	9.85	12.293403	3.9295393	3	3	2	94.3	105.7	1.121	
Q9NUM4	Transmembrane protein 106B	31.108	6.99	6.0802422	4.7445255	1	1	1	94.3	105.7	1.121	
Q9ULX6	A-kinase anchor protein 8-like	71.604	5.07	10.505188	3.250774	2	2	2	94.3	105.7	1.121	2.38E-01
P04626	Receptor tyrosine-protein kinase erbB-2	137.821	5.91	14.247506	1.6733068	2	2	1	94.2	105.8	1.123	
P30047	GTP cyclohydrolase 1 feedback regulatory protein	9.692	6.54	15.007943	48.809524	2	3	2	94.2	105.8	1.123	1.32E-01
Q53LP3	Ankyrin repeat domain-containing protein SOWAHC	55.638	7.03	13.358418	7.047619	3	3	3	94.2	105.8	1.123	
Q9NUT2	ATP-binding cassette sub-family B member 8, mitochondrial	79.938	8.98	7.378378	3.8095238	2	2	1	94.2	105.8	1.123	
Q9NY35	Claudin domain-containing protein 1	28.584	5.82	6.3766473	5.5335968	1	1	1	94.2	105.8	1.123	
O60566	Mitotic checkpoint serine/threonine-protein kinase BUB1 beta	119.47	5.27	15.585955	2.952381	3	3	3	94.1	105.9	1.125	2.21E-02
P02792	Ferritin light chain	20.007	5.78	14.8306	8.5714286	1	2	1	94.1	105.9	1.125	
Q15276	Rab GTPase-binding effector protein 1	99.229	5.01	25.016194	5.6844548	4	4	4	94.1	105.9	1.125	
Q6IBS0	Twinfilin-2	39.523	6.84	43.598879	16.045845	4	8	4	94.1	105.9	1.125	8.38E-02
Q6P0N0	Mis18-binding protein 1	129.005	9.25	2.8613816	0.6183746	1	1	1	94.1	105.9	1.125	
Q8N884	Cyclic GMP-AMP synthase	58.777	9.48	10.992705	3.6398467	2	2	2	94.1	105.9	1.125	
Q93096	Protein tyrosine phosphatase type IVA 1	19.802	8.97	43.246619	29.479769	4	7	4	94.1	105.9	1.125	2.67E-03
P56381	ATP synthase subunit epsilon, mitochondrial	5.776	9.92	2.6258017	13.72549	1	1	1	94	106	1.128	
Q14147	Probable ATP-dependent RNA helicase DHX34	128.04	7.56	18.515347	3.3245844	2	2	2	94	106	1.128	4.97E-02
Q9BWU0	Kanadaptin	88.759	5.19	32.15665	9.5477387	6	6	6	94	106	1.128	1.48E-02
Q9Y2Z4	Tyrosine--tRNA ligase, mitochondrial	53.166	8.98	28.52928	11.949686	4	6	4	94	106	1.128	1.01E-02
Q13416	Origin recognition complex subunit 2	65.931	6.51	8.7718597	3.812825	2	2	2	93.9	106.1	1.13	
Q13573	SNW domain-containing protein 1	61.456	9.52	24.930005	8.5820896	4	5	4	93.9	106.1	1.13	8.44E-04
Q6VN20	Ran-binding protein 10	67.214	6.77	21.610054	5.1612903	3	3	3	93.9	106.1	1.13	
Q8IWZ3	Ankyrin repeat and KH domain-containing protein 1	269.291	5.73	85.58335	4.8780488	13	17	5	93.9	106.1	1.13	4.25E-01
Q9BUK0	Coiled-coil-helix-coiled-coil-helix domain-containing protein 7	10.089	8.82	8.6645421	21.176471	1	1	1	93.9	106.1	1.13	
Q9BV40	Vesicle-associated membrane protein 8	11.431	7.34	9.4631891	10	1	1	1	93.9	106.1	1.13	
O75683	Surfeit locus protein 6	41.426	10.64	28.741212	8.5872576	4	5	4	93.8	106.2	1.132	2.82E-02
P0DMU6	Cancer/testis antigen family 45 member A4	21.231	9.76	34.295229	26.984127	5	7	5	93.8	106.2	1.132	8.70E-03
P42025	Beta-centractin	42.267	6.4	41.630274	17.553191	5	11	2	93.8	106.2	1.132	5.04E-04
P61927	60S ribosomal protein L37	11.071	11.74	9.9123564	29.896907	4	7	4	93.8	106.2	1.132	
P62906	60S ribosomal protein L10a	24.816	9.94	103.86877	37.327189	8	21	8	93.8	106.2	1.132	2.43E-05
Q5T7V8	RAB6-interacting golgin	44.965	7.08	9.477295	5.0761421	1	1	1	93.8	106.2	1.132	
Q8WZA9	Immunity-related GTPase family Q protein	62.678	4.88	42.03649	8.6677368	4	5	4	93.8	106.2	1.132	6.18E-02
Q9NV06	DDB1- and CUL4-associated factor 13	51.369	9.19	30.69395	12.58427	4	5	4	93.8	106.2	1.132	3.74E-03
P30281	G1/S-specific cyclin-D3	32.499	7.06	8.6625407	3.7671233	1	1	1	93.7	106.3	1.134	
Q03111	Protein ENL	62.018	8.59	12.580138	6.9767442	4	5	4	93.7	106.3	1.134	2.36E-02
Q13488	V-type proton ATPase 116 kDa subunit a isoform 3	92.908	7.12	11.391671	3.1325301	2	2	2	93.7	106.3	1.134	
Q8N6H7	ADP-ribosylation factor GTPase-activating protein 2	56.685	7.99	17.070138	7.6775432	3	3	3	93.7	106.3	1.134	1.21E-01
Q96MU7	YTH domain-containing protein 1	84.649	6.23	4.5761659	2.0632737	2	3	2	93.7	106.3	1.134	
Q96RQ1	Endoplasmic reticulum-Golgi intermediate compartment protein 2	42.521	6.77	15.273507	5.3050398	2	3	2	93.7	106.3	1.134	2.94E-02
P49593	Protein phosphatase 1F	49.8	5.1	23.61162	10.132159	3	3	3	93.6	106.4	1.137	
Q5T9A4	ATPase family AAA domain-containing protein 3B	72.527	9.2	41.906056	16.049383	10	14	1	93.6	106.4	1.137	
Q9Y285	Phenylalanine--tRNA ligase alpha subunit	57.528	7.8	89.08527	26.574803	10	15	10	93.6	106.4	1.137	5.57E-03
Q9Y2T2	AP-3 complex subunit mu-1	46.909	6.93	21.292236	9.3301435	3	3	3	93.6	106.4	1.137	1.06E-01
Q9Y4P1	Cysteine protease ATG4B	44.266	5.07	19.678757	11.959288	3	4	3	93.6	106.4	1.137	7.74E-04

O15085	Rho guanine nucleotide exchange factor 11	167.601	5.5	4.7609509	0.5913272	1	1	1	93.5	106.5	1.139	
O43293	Death-associated protein kinase 3	52.503	6.89	18.854885	7.0484581	3	3	2	93.5	106.5	1.139	
P13645	Keratin, type I cytoskeletal 10	58.792	5.21	62.388102	15.410959	9	11	8	93.5	106.5	1.139	1.21E-01
P62910	60S ribosomal protein L32	15.85	11.33	22.441327	23.703704	4	8	4	93.5	106.5	1.139	5.36E-02
Q96C90	Protein phosphatase 1 regulatory subunit 14B	15.901	4.86	6.2150976	17.006803	1	2	1	93.5	106.5	1.139	
Q9NWU1	3-oxoacyl-[acyl-carrier-protein] synthase, mitochondrial	48.812	7.66	10.604933	5.664488	2	2	2	93.5	106.5	1.139	
O60232	Sjoegren syndrome/scleroderma autoantigen 1	21.461	5.24	29.412385	33.668342	4	6	4	93.4	106.6	1.141	1.41E-03
P51809	Vesicle-associated membrane protein 7	24.919	8.6	13.04171	11.363636	3	4	3	93.4	106.6	1.141	3.00E-01
O43301	Heat shock 70 kDa protein 12A	74.931	6.77	14.764966	1.9259259	1	2	1	93.3	106.7	1.144	
O60783	28S ribosomal protein S14, mitochondrial	15.129	11.41	13.000782	11.71875	1	1	1	93.3	106.7	1.144	
P04732	Metallothionein-1E	6.009	7.96	61.480311	67.213115	4	16	2	93.3	106.7	1.144	5.95E-01
Q16763	Ubiquitin-conjugating enzyme E2 S	23.831	8.38	31.011231	24.324324	3	5	3	93.3	106.7	1.144	7.80E-05
Q9BTA9	WW domain-containing adapter protein with coiled-coil	70.681	9.45	14.402753	4.1731066	2	2	2	93.3	106.7	1.144	
Q9NUQ2	1-acyl-sn-glycerol-3-phosphate acyltransferase epsilon	42.045	9.1	7.8431418	6.5934066	3	3	3	93.3	106.7	1.144	
Q15758	Neutral amino acid transporter B(0)	56.562	5.48	57.885767	17.560074	7	14	7	93.2	106.8	1.146	5.11E-04
Q8NBM4	Ubiquitin-associated domain-containing protein 2	38.938	9.01	9.119118	7.8488372	2	2	2	93.2	106.8	1.146	1.59E-01
Q8NBZ7	UDP-glucuronic acid decarboxylase 1	47.547	8.94	10.450384	4.047619	1	1	1	93.2	106.8	1.146	
Q9H4H8	Protein FAM83D	64.384	6.54	11.688148	4.7863248	3	4	3	93.2	106.8	1.146	6.69E-03
Q9NRX4	14 kDa phosphohistidine phosphatase	13.824	6.07	21.46082	28	4	5	4	93.2	106.8	1.146	2.28E-01
Q9NSK0	Kinesin light chain 4	68.598	6.18	40.36069	14.216478	8	8	6	93.2	106.8	1.146	
Q9BVI4	Nucleolar complex protein 4 homolog	58.431	7.49	24.873715	4.2635659	2	3	2	93.1	106.9	1.148	
Q9P000	COMM domain-containing protein 9	21.805	5.88	13.622127	12.121212	2	2	2	93.1	106.9	1.148	
P27540	Aryl hydrocarbon receptor nuclear translocator	86.582	6.54	11.106226	3.5487959	2	2	2	93	107	1.151	
Q6PEV8	Protein FAM199X	42.775	5.08	3.7086311	3.8659794	1	1	1	93	107	1.151	
Q86V85	Integral membrane protein GPR180	49.363	7.39	14.278684	3.4090909	1	1	1	93	107	1.151	
Q9BQE4	Selenoprotein S	21.151	9.7	16.375709	19.57672	3	3	3	93	107	1.151	1.04E-01
Q9HCH0	Nck-associated protein 5-like	138.927	8.13	5.0445528	0.9774436	1	1	1	93	107	1.151	
P82930	28S ribosomal protein S34, mitochondrial	25.634	9.98	14.97157	18.348624	4	4	4	92.9	107.1	1.153	8.83E-02
Q12999	Tetraspanin-31	23.038	7.97	9.3205614	9.047619	2	4	2	92.9	107.1	1.153	4.64E-02
Q13330	Metastasis-associated protein MTA1	80.735	9.26	9.9519391	3.9160839	3	3	1	92.9	107.1	1.153	
Q8IWD4	Coiled-coil domain-containing protein 117	30.522	5.07	5.5641556	3.2258065	1	1	1	92.9	107.1	1.153	
Q8IXK0	Polyhomeotic-like protein 2	90.657	8.69	16.437779	4.1958042	3	3	2	92.9	107.1	1.153	
Q9UL40	Zinc finger protein 346	32.912	9.09	6.8175853	3.7414966	1	1	1	92.9	107.1	1.153	
P23434	Glycine cleavage system H protein, mitochondrial	18.873	4.88	36.126985	25.433526	4	6	4	92.8	107.2	1.155	3.35E-01
Q15005	Signal peptidase complex subunit 2	24.987	8.47	18.82406	17.699115	3	3	3	92.8	107.2	1.155	2.14E-01
Q8WUX2	Putative glutathione-specific gamma-glutamylcyclotransferase 2	20.862	5.43	6.5797141	8.6956522	1	1	1	92.8	107.2	1.155	
Q86VP1	Tax1-binding protein 1	90.82	5.43	34.518132	6.4638783	6	8	6	92.7	107.3	1.157	
Q8IXW5	Putative RNA polymerase II subunit B1 CTD phosphatase RPAP2	69.466	7.78	4.723572	3.4313725	2	2	2	92.7	107.3	1.157	
O43683	Mitotic checkpoint serine/threonine-protein kinase BUB1	122.297	6.47	4.8548036	0.8294931	1	1	1	92.6	107.4	1.16	
P51795	H(+)/Cl(-) exchange transporter 5	83.093	6.81	6.0790944	2.1447721	1	1	1	92.6	107.4	1.16	
P52943	Cysteine-rich protein 2	22.478	8.72	27.932088	35.576923	4	4	4	92.6	107.4	1.16	1.95E-02
Q8N9R8	Protein SCAI	70.355	8.6	4.9465374	1.650165	1	1	1	92.6	107.4	1.16	
Q9BXW9	Fanconi anemia group D2 protein	164.025	5.88	24.928084	3.6526533	4	4	4	92.6	107.4	1.16	
Q9Y3E1	Hepatoma-derived growth factor-related protein 3	22.606	7.99	9.5979655	7.8817734	2	5	1	92.6	107.4	1.16	
P00973	2'-5'-oligoadenylate synthase 1	45.999	8.22	6.891619	4.25	2	2	2	92.5	107.5	1.162	1.19E-02
O95297	Myelin protein zero-like protein 1	29.064	8.72	9.5894926	7.063197	2	2	2	92.4	107.6	1.165	
Q8WU10	yridine nucleotide-disulfide oxidoreductase domain-containing protein	55.757	5.85	10.988853	3.6	1	1	1	92.4	107.6	1.165	

Q9BUK6	Protein misato homolog 1	61.796	6.11	13.694501	4.7368421	3	4	3	92.4	107.6	1.165	
Q9H3Y8	Pancreatic progenitor cell differentiation and proliferation factor	11.77	7.42	4.3517376	14.035088	1	1	1	92.4	107.6	1.165	
O43823	A-kinase anchor protein 8	76.061	5.15	17.143258	4.6242775	3	3	3	92.3	107.7	1.167	1.21E-01
Q8IZ73	RNA pseudouridylate synthase domain-containing protein 2	61.273	7.17	9.587592	5.1376147	2	2	2	92.3	107.7	1.167	
Q92733	Proline-rich protein PRCC	52.386	5.1	4.4641997	2.8513238	1	1	1	92.3	107.7	1.167	
O15084	rine/threonine-protein phosphatase 6 regulatory ankyrin repeat subunit	112.894	6.25	6.8948931	2.2792023	2	2	2	92.2	107.8	1.169	2.27E-01
Q96IF1	LIM domain-containing protein ajuba	56.897	7.14	3.4408118	2.0446097	1	1	1	92.2	107.8	1.169	
Q9BXK1	Krueppel-like factor 16	25.415	9.88	3.2107719	4.3650794	1	1	1	92.2	107.8	1.169	
Q9P2X3	Protein IMPACT	36.453	4.97	4.6722325	3.4375	1	1	1	92.2	107.8	1.169	
O75381	Peroxisomal membrane protein PEX14	41.212	4.94	16.550351	9.0185676	3	3	3	92.1	107.9	1.172	1.47E-01
Q9H3F6	)Z domain-containing adapter for CUL3-mediated RhoA degradation p	35.41	6.34	5.1414628	3.514377	1	1	1	92.1	107.9	1.172	
P25490	Transcriptional repressor protein YY1	44.685	6.25	12.866876	7.4879227	4	4	4	92	108	1.174	
P62854	40S ribosomal protein S26	13.007	11	39.95209	26.956522	3	8	3	92	108	1.174	3.10E-01
Q02978	Mitochondrial 2-oxoglutarate/malate carrier protein	34.04	9.91	29.359073	13.694268	4	7	4	92	108	1.174	1.89E-01
Q13426	DNA repair protein XRCC4	38.263	4.98	9.9613389	9.2261905	3	3	3	92	108	1.174	3.52E-01
Q17RY6	Lymphocyte antigen 6K	18.66	7.43	13.16326	12.727273	2	2	2	92	108	1.174	
Q5T3I0	G patch domain-containing protein 4	50.351	9.63	46.95996	16.591928	7	9	7	92	108	1.174	1.29E-03
Q8TF68	Zinc finger protein 384	63.179	8.95	10.798043	3.1195841	2	2	2	92	108	1.174	3.33E-03
Q9NRL2	Bromodomain adjacent to zinc finger domain protein 1A	178.592	6.6	9.5808707	0.8997429	1	1	1	92	108	1.174	
Q14241	Transcription elongation factor B polypeptide 3	89.853	9.57	10.849154	4.0100251	4	4	4	91.9	108.1	1.176	1.89E-03
Q3V6T2	Girdin	215.909	6.21	18.048523	1.6034206	3	3	3	91.9	108.1	1.176	
Q9NYF8	Bcl-2-associated transcription factor 1	106.059	9.98	60.018478	11.847826	11	18	11	91.9	108.1	1.176	1.20E-01
O14640	Segment polarity protein dishevelled homolog DVL-1	75.14	7.81	10.3593	3.0215827	2	2	1	91.8	108.2	1.179	
P14635	G2/mitotic-specific cyclin-B1	48.306	7.47	37.205178	15.473441	6	8	6	91.8	108.2	1.179	
P27635	60S ribosomal protein L10	24.588	10.08	58.44598	19.158879	6	17	6	91.8	108.2	1.179	9.55E-03
Q02878	60S ribosomal protein L6	32.708	10.58	32.941198	20.138889	6	10	6	91.8	108.2	1.179	2.97E-03
Q9Y4K3	TNF receptor-associated factor 6	59.534	6.44	7.4170281	2.2988506	1	1	1	91.7	108.3	1.181	
P62847	40S ribosomal protein S24	15.413	10.78	63.829298	25.56391	4	13	4	91.4	108.6	1.188	1.33E-02
Q658Y4	Protein FAM91A1	93.85	6.39	12.982423	3.5799523	3	3	3	91.3	108.7	1.191	
Q8IYB7	DIS3-like exonuclease 2	99.216	6.1	9.2343111	2.8248588	3	3	3	91.3	108.7	1.191	
Q9NZ32	Actin-related protein 10	46.277	7.37	11.632188	5.9952038	2	2	2	91.2	108.8	1.193	
P61353	60S ribosomal protein L27	15.788	10.56	10.253435	12.5	2	3	2	91.1	108.9	1.195	7.47E-04
Q10589	Bone marrow stromal antigen 2	19.756	5.6	11.43905	13.333333	2	2	2	90.9	109.1	1.2	1.18E-03
Q8NFJ5	Retinoic acid-induced protein 3	40.225	8.15	22.227789	13.165266	4	7	4	90.9	109.1	1.2	6.81E-02
Q96JP5	E3 ubiquitin-protein ligase ZFP91	63.406	7.36	3.1741197	1.4035088	1	1	1	90.8	109.2	1.203	
Q9H4L7	ix-associated actin-dependent regulator of chromatin subfamily A cont	117.328	5.55	33.83835	4.8732943	4	6	4	90.8	109.2	1.203	
P39023	60S ribosomal protein L3	46.08	10.18	151.02734	32.009926	13	44	13	90.7	109.3	1.205	2.33E-07
Q9BYD3	39S ribosomal protein L4, mitochondrial	34.897	9.72	15.792617	14.14791	4	5	4	90.7	109.3	1.205	
P29317	Ephrin type-A receptor 2	108.197	6.23	24.093991	4.0983607	4	6	4	90.6	109.4	1.208	3.18E-02
Q96T21	Selenocysteine insertion sequence-binding protein 2	95.402	8.12	8.6511113	2.3419204	1	1	1	90.6	109.4	1.208	
O75764	Transcription elongation factor A protein 3	38.947	9.19	5.3398938	2.8735632	1	1	1	90.5	109.5	1.21	
Q92925	ted matrix-associated actin-dependent regulator of chromatin subfamil	58.884	9.64	15.346338	8.6629002	4	4	3	90.5	109.5	1.21	2.03E-01
Q9UJF2	Ras GTPase-activating protein nGAP	128.477	8.24	13.767866	1.7559263	2	3	2	90.5	109.5	1.21	
P61313	60S ribosomal protein L15	24.131	11.62	27.285621	18.137255	4	6	4	90.4	109.6	1.212	1.14E-01
Q9Y2P8	RNA 3'-terminal phosphate cyclase-like protein	40.817	9.26	10.430646	8.310992	3	3	3	90.4	109.6	1.212	
Q9Y2W6	Tudor and KH domain-containing protein	62.007	5.02	9.5045203	3.9215686	2	2	2	90.4	109.6	1.212	
O14841	5-oxoprolinase	137.371	6.58	3.5477534	1.863354	1	1	1	90.3	109.7	1.215	

P12931	Proto-oncogene tyrosine-protein kinase Src	59.797	7.42	29.146796	11.380597	6	6	2	90.1	109.9	1.22	
O76095	Protein JTB	16.347	8.28	4.9237237	8.9041096	1	1	1	89.9	110.1	1.225	
Q9NRX1	RNA-binding protein PNO1	27.907	9.73	17.310559	8.7301587	2	3	2	89.9	110.1	1.225	
Q9UK76	Hematological and neurological expressed 1 protein	16.005	5.6	49.971816	41.558442	4	9	4	89.9	110.1	1.225	3.02E-02
O75348	V-type proton ATPase subunit G 1	13.749	8.79	7.7517814	11.864407	1	1	1	89.8	110.2	1.227	
P40429	60S ribosomal protein L13a	23.562	10.93	41.053684	32.512315	9	18	9	89.8	110.2	1.227	2.59E-05
Q7Z4H8	KDEL motif-containing protein 2	58.535	8.24	17.15337	9.270217	4	4	4	89.7	110.3	1.23	6.86E-01
Q5VIR6	Vacuolar protein sorting-associated protein 53 homolog	79.602	6.02	6.4485817	2.4320458	2	2	2	89.6	110.4	1.232	
O75815	Breast cancer anti-estrogen resistance protein 3	92.507	7.96	6.0459988	1.0909091	1	1	1	89.4	110.6	1.237	
Q9UQR0	Sex comb on midleg-like protein 2	77.208	8.54	5.0195875	2.1428571	1	1	1	89.4	110.6	1.237	
P04264	Keratin, type II cytoskeletal 1	65.999	8.12	92.252084	16.459627	10	13	10	89	111	1.247	1.23E-03
Q96S82	Ubiquitin-like protein 7	40.485	5.07	14.907741	10.263158	3	3	3	89	111	1.247	2.93E-01
Q9H7B2	Ribosome production factor 2 homolog	35.56	9.99	3.1776357	2.6143791	1	1	1	89	111	1.247	
Q9UFN0	Protein NipSnap homolog 3A	28.449	9.16	7.2819335	8.097166	2	2	2	89	111	1.247	1.67E-01
Q53GA4	Pleckstrin homology-like domain family A member 2	17.082	9.17	7.4959371	5.9210526	1	1	1	88.8	111.2	1.252	
Q8TD55	Pleckstrin homology domain-containing family O member 2	53.317	5.43	4.8642315	4.6938776	1	1	1	88.7	111.3	1.255	
Q12805	EGF-containing fibulin-like extracellular matrix protein 1	54.604	5.07	18.382929	5.4766734	2	3	2	88.4	111.6	1.262	
Q9UPQ9	Trinucleotide repeat-containing gene 6B protein	193.883	6.76	6.2183882	0.4909984	1	1	1	88.2	111.8	1.268	
Q8N257	Histone H2B type 3-B	13.9	10.32	91.528237	57.142857	8	94	3	86.9	113.1	1.301	2.69E-01
Q9NPE3	H/ACA ribonucleoprotein complex subunit 3	7.701	9.99	9.7490459	32.8125	2	4	2	86.9	113.1	1.301	6.42E-02
Q9BX40	Protein LSM14 homolog B	42.045	9.69	14.407652	2.8571429	1	2	1	86.4	113.6	1.315	
P42167	Lamina-associated polypeptide 2, isoforms beta/gamma	50.639	9.38	131.54972	39.867841	13	31	5	86.1	113.9	1.323	2.15E-01
Q8N0T1	Uncharacterized protein C8orf59	11.449	10.46	3.6530605	9	1	1	1	86.1	113.9	1.323	
Q9BYG5	Partitioning defective 6 homolog beta	41.157	5.58	4.4306261	2.9569892	1	1	1	85.6	114.4	1.336	
Q9HAW4	Claspin	151.002	4.82	5.3974502	1.1949216	2	2	2	85.1	114.9	1.35	
Q9BZL1	Ubiquitin-like protein 5	8.541	8.44	4.0514785	24.657534	2	2	2	85	115	1.353	3.11E-01
P50579	Methionine aminopeptidase 2	52.858	5.82	61.911225	13.807531	5	9	5	84.8	115.2	1.358	3.36E-02
Q9NZM3	Intersectin-2	193.34	8.12	5.5089129	1.1785504	2	2	1	83.9	116.1	1.384	
Q16719	Kynureninase	52.318	7.03	7.2303273	2.5806452	1	1	1	81.8	118.2	1.445	
P62875	DNA-directed RNA polymerases I, II, and III subunit RPABC5	7.64	7.77	3.9775716	16.41791	1	1	1	81.5	118.5	1.454	
P30740	Leukocyte elastase inhibitor	42.715	6.28	46.605192	19.525066	8	9	8	80.7	119.3	1.478	8.92E-04
A1A4S6	Rho GTPase-activating protein 10	89.318	7.18	2.3168629	0.8905852	1	1	1				
A5PLL7	Transmembrane protein 189	31.115	6.83	3.5989443	4.0740741	1	1	1				
A6NCE7	Microtubule-associated proteins 1A/1B light chain 3 beta 2	14.619	8.68	4.3960982	11.2	1	1	1				
A6NFQ2	TRPM8 channel-associated factor 2	100.841	7.17	4.5034855	1.3057671	1	1	1				
O00221	NF-kappa-B inhibitor epsilon	52.831	6.68	4.7042131	2.4	1	1	1				
O00255	Menin	67.981	6.55	5.2632052	2.2764228	1	1	1				
O00411	DNA-directed RNA polymerase, mitochondrial	138.532	8.98	4.9336741	0.9756098	1	1	1				
O00423	Echinoderm microtubule-associated protein-like 1	89.805	7.06	4.5826944	1.2269939	1	1	1				
O00458	Interferon-related developmental regulator 1	50.236	7.18	10.076704	3.7694013	1	1	1				
O00479	High mobility group nucleosome-binding domain-containing protein 4	9.533	10.48	3.7210179	10	1	1	1				
O14521	nate dehydrogenase [ubiquinone] cytochrome b small subunit, mitocho	17.032	8.63	2.4996263	4.4025157	1	1	1				
O14730	Serine/threonine-protein kinase RIO3	59.055	5.76	4.0534004	1.5414258	1	1	1				
O14735	CDP-diacylglycerol--inositol 3-phosphatidyltransferase	23.523	8.03	3.2926002	5.1643192	1	1	1				
O14770	Homeobox protein Meis2	51.757	6.4	4.4613006	1.6771488	1	1	1				
O14972	Down syndrome critical region protein 3	32.989	7.68	5.76675	4.040404	1	1	1				
O15013	Rho guanine nucleotide exchange factor 10	151.516	5.68	4.2491061	0.6574142	1	1	1				

O15127	Secretory carrier-associated membrane protein 2	36.625	6.1	19.241748	9.7264438	2	2	2
O15235	28S ribosomal protein S12, mitochondrial	15.163	10.29	12.354283	18.115942	1	1	1
O15243	Leptin receptor gene-related protein	14.244	6.51	7.8392314	10.687023	1	1	1
O15258	Protein RER1	22.943	9.54	3.3660268	4.0816327	1	1	1
O15440	Multidrug resistance-associated protein 5	160.558	8.66	3.3898724	0.6263048	1	1	1
O43164	E3 ubiquitin-protein ligase Praja-2	78.166	4.39	2.3909395	1.1299435	1	1	1
O43257	Zinc finger HIT domain-containing protein 1	17.525	9.41	3.9681877	5.8441558	1	1	1
O43292	Glycosylphosphatidylinositol anchor attachment 1 protein	67.58	8.06	6.9922493	3.5426731	2	2	2
O43493	Trans-Golgi network integral membrane protein 2	51.082	5.73	8.8755272	5.8333333	3	4	3
O43653	Prostate stem cell antigen	12.903	5.29	10.834585	8.1300813	1	2	1
O43716	Glutamyl-tRNA(Gln) amidotransferase subunit C, mitochondrial	15.077	5.05	12.018989	20.588235	3	4	3
O43819	Protein SCO2 homolog, mitochondrial	29.791	8.85	18.489159	14.285714	3	3	3
O60294	tRNA wybutosine-synthesizing protein 4	75.554	6.73	7.2081691	1.8950437	1	1	1
O60318	Germinal-center associated nuclear protein	218.267	6.39	3.8195874	0.4545455	1	1	1
O60337	E3 ubiquitin-protein ligase MARCH6	102.478	6.28	3.4287571	0.989011	1	1	1
O60476	Mannosyl-oligosaccharide 1,2-alpha-mannosidase IB	72.957	7.61	2.58603	1.7160686	1	1	1
O60573	Eukaryotic translation initiation factor 4E type 2	28.344	8.88	3.9262816	5.3061224	1	1	1
O60870	DNA/RNA-binding protein KIN17	45.345	8.95	4.2997291	2.5445293	1	1	1
O60942	mRNA-capping enzyme	68.513	8.13	3.1898347	1.6750419	1	1	1
O75052	Carboxyl-terminal PDZ ligand of neuronal nitric oxide synthase protei	56.115	6.3	6.5958508	2.173913	1	1	1
O75147	Obscurin-like protein 1	206.817	5.63	3.1754186	0.5274262	1	1	1
O75386	Tubby-related protein 3	49.611	7.74	5.2954806	3.6199095	2	2	2
O75394	39S ribosomal protein L33, mitochondrial	7.614	10.8	6.0827571	16.923077	1	1	1
O75496	Geminin	23.551	4.96	12.784641	9.0909091	2	2	2
O75554	WW domain-binding protein 4	42.481	5.57	5.3255982	2.9255319	1	1	1
O75582	Ribosomal protein S6 kinase alpha-5	89.809	7.11	15.032745	4.8628429	4	4	2
O75674	TOM1-like protein 1	52.956	5.24	8.7936572	4.4117647	2	2	2
O75688	Protein phosphatase 1B	52.609	5.05	7.1091879	2.9227557	1	1	1
O75911	Short-chain dehydrogenase/reductase 3	33.527	8.84	6.3763373	4.3046358	1	1	1
O76064	E3 ubiquitin-protein ligase RNF8	55.482	7.33	4.7582046	1.8556701	1	1	1
O94818	Nucleolar protein 4	71.312	5.62	7.3838397	2.0376176	1	1	1
O94829	Importin-13	108.126	5.3	10.506047	3.9460021	2	2	2
O94915	Protein furry homolog-like	339.383	5.58	6.5968795	0.3650846	1	1	1
O95208	Epsin-2	68.439	7.52	9.2116195	2.6521061	1	1	1
O95263	finity cAMP-specific and IBMX-insensitive 3',5'-cyclic phosphodieste	98.916	6.83	9.361379	2.0338983	2	2	1
O95396	Adenylyltransferase and sulfurtransferase MOCS3	49.638	6.21	3.9952488	1.5217391	1	1	1
O95427	GPI ethanolamine phosphate transferase 1	105.741	8.65	4.7157949	1.2889366	1	1	1
O95551	Tyrosyl-DNA phosphodiesterase 2	40.903	5.1	19.516346	8.5635359	3	4	3
O95684	FGFR1 oncogene partner	43.039	4.81	8.8294449	4.0100251	1	1	1
O96011	Peroxisomal membrane protein 11B	28.413	9.85	5.5689581	4.2471042	1	1	1
O96017	Serine/threonine-protein kinase Chk2	60.877	5.91	5.2449641	1.8416206	1	1	1
O96018	Amyloid beta A4 precursor protein-binding family A member 3	61.416	4.94	7.8345165	2.9565217	2	3	1
P00813	Adenosine deaminase	40.739	5.95	6.2727919	4.1322314	2	2	2
P00846	ATP synthase subunit a	24.801	10.1	8.9708426	4.4247788	1	2	1
P04066	Tissue alpha-L-fucosidase	53.655	6.84	6.126912	2.360515	1	1	1
P05067	Amyloid beta A4 protein	86.888	4.82	3.9948195	1.1688312	1	1	1
P06213	Insulin receptor	156.232	6.2	3.4667365	0.6512301	1	1	1

P07738	Bisphosphoglycerate mutase	29.987	6.54	13.970211	7.3359073	1	1	1
P08069	Insulin-like growth factor 1 receptor	154.693	5.8	2.9991323	0.658376	1	1	1
P08574	Cytochrome c1, heme protein, mitochondrial	35.399	9	2.526513	2.1538462	1	1	1
P09958	Furin	86.624	6.47	3.5250557	0.8816121	1	1	1
P0DI82	Trafficking protein particle complex subunit 2B	16.434	6.52	4.6974526	5.7142857	1	1	1
P10244	Myb-related protein B	78.715	6.87	6.1339491	1.4285714	1	1	1
P11234	Ras-related protein Ral-B	23.394	6.62	15.104742	9.7087379	2	3	1
P12109	Collagen alpha-1(VI) chain	108.462	5.43	4.3950481	1.3618677	2	2	2
P12694	2-oxoisovalerate dehydrogenase subunit alpha, mitochondrial	50.439	8.27	14.224568	4.7191011	2	2	2
P13051	Uracil-DNA glycosylase	34.624	9.32	4.930702	3.514377	1	1	1
P13612	Integrin alpha-4	114.827	6.48	2.9698052	0.6782946	1	1	1
P15056	Serine/threonine-protein kinase B-raf	84.384	7.53	3.8552483	1.8276762	2	2	1
P15848	Arylsulfatase B	59.649	8.21	3.6509168	1.8761726	1	1	1
P16930	Fumarylacetoacetase	46.344	6.95	5.8987913	5.0119332	2	2	2
P17028	Zinc finger protein 24	42.129	6.21	5.190171	2.4456522	1	1	1
P17301	Integrin alpha-2	129.214	5.31	7.5639965	1.1007621	1	1	1
P19021	Peptidyl-glycine alpha-amidating monooxygenase	108.263	6.42	3.4446637	0.9249743	1	1	1
P19447	TFIIH basal transcription factor complex helicase XPB subunit	89.221	7.23	4.1013297	1.1508951	1	1	1
P19474	E3 ubiquitin-protein ligase TRIM21	54.135	6.38	5.0434954	1.8947368	1	1	1
P20338	Ras-related protein Rab-4A	24.374	6.07	15.768126	11.46789	2	5	1
P21580	Tumor necrosis factor alpha-induced protein 3	89.556	8.22	6.3030167	1.0126582	1	2	1
P21589	5'-nucleotidase	63.327	7.03	4.3732491	2.0905923	1	1	1
P22415	Upstream stimulatory factor 1	33.518	5.54	2.6039751	2.9032258	1	1	1
P22830	Ferrochelatase, mitochondrial	47.832	8.73	8.5691191	5.9101655	2	2	2
P23458	Tyrosine-protein kinase JAK1	133.191	7.55	9.2722443	1.9930676	2	3	2
P23511	Nuclear transcription factor Y subunit alpha	36.854	8.94	10.698102	5.4755043	1	1	1
P24844	Myosin regulatory light polypeptide 9	19.814	4.92	62.700019	44.186047	6	12	1
P30414	NK-tumor recognition protein	165.577	9.99	3.0379149	0.4787962	1	1	1
P30479	HLA class I histocompatibility antigen, B-41 alpha chain	40.514	6.54	71.985408	23.20442	6	15	0
P30492	HLA class I histocompatibility antigen, B-54 alpha chain	40.355	6.3	53.954097	22.651934	6	11	0
P31271	Homeobox protein Hox-A13	39.702	9.13	9.2497231	4.3814433	1	1	1
P31321	cAMP-dependent protein kinase type I-beta regulatory subunit	43.046	5.71	4.0173218	3.1496063	1	1	1
P32121	Beta-arrestin-2	46.077	7.69	4.5397037	2.9339853	1	1	1
P33981	Dual specificity protein kinase TTK	97.011	8.16	6.7882043	2.4504084	2	2	2
P34059	N-acetylgalactosamine-6-sulfatase	57.989	6.74	4.3018995	1.532567	1	1	1
P34741	Syndecan-2	22.146	4.86	4.8501653	4.4776119	1	1	1
P35226	Polycomb complex protein BMI-1	36.925	8.63	6.7488487	3.3742331	1	1	1
P35240	Merlin	69.646	6.47	2.8144578	1.8487395	1	1	1
P35555	Fibrillin-1	312.022	4.93	4.6251767	0.5921282	2	2	1
P35813	Protein phosphatase 1A	42.421	5.36	13.086111	6.0209424	2	2	2
P40261	Nicotinamide N-methyltransferase	29.555	5.74	4.8016179	3.030303	1	1	1
P42684	Abelson tyrosine-protein kinase 2	128.263	8.07	5.0722704	0.9306261	1	1	1
P42694	Probable helicase with zinc finger domain	218.833	7.42	12.010727	1.0813594	2	2	2
P42702	Leukemia inhibitory factor receptor	123.665	5.72	16.892081	3.5551504	3	3	3
P46020	hosphorylase b kinase regulatory subunit alpha, skeletal muscle isofor	137.225	6.19	4.092965	0.572363	1	1	1
P46531	Neurogenic locus notch homolog protein 1	272.323	5.12	5.4521949	0.665362	2	2	1
P46734	Dual specificity mitogen-activated protein kinase kinase 3	39.293	7.43	8.6610647	6.9164265	3	3	1

P46821	Microtubule-associated protein 1B	270.468	4.81	10.371524	0.6482982	2	2	1
P48382	DNA-binding protein RFX5	65.283	9.29	13.973455	4.0584416	2	2	2
P48723	Heat shock 70 kDa protein 13	51.895	5.76	6.0204516	2.9723992	1	1	1
P49427	Ubiquitin-conjugating enzyme E2 R1	26.72	4.54	7.7490923	4.6610169	1	1	1
P49746	Thrombospondin-3	104.135	4.65	8.4509967	1.5690377	1	1	1
P49761	Dual specificity protein kinase CLK3	73.469	9.92	8.759456	3.1347962	2	2	2
P50542	Peroxisomal targeting signal 1 receptor	70.821	4.54	6.9833845	2.3474178	1	1	1
P51798	H(+)/Cl(-) exchange transporter 7	88.622	8.43	2.8696662	0.8695652	1	1	1
P52435	DNA-directed RNA polymerase II subunit RPB11-a	13.285	5.86	3.8147412	6.8376068	1	1	1
P53355	Death-associated protein kinase 1	159.945	6.83	5.4854921	1.1888112	2	2	1
P53779	Mitogen-activated protein kinase 10	52.551	6.79	6.851089	3.0172414	1	1	1
P54098	DNA polymerase subunit gamma-1	139.473	6.9	9.0187252	1.614205	1	1	1
P55011	Solute carrier family 12 member 2	131.364	6.4	4.0993598	0.7425743	1	1	1
P56962	Syntaxin-17	33.383	6.57	4.7950665	3.3112583	1	1	1
P57081	tRNA (guanine-N(7)-)-methyltransferase non-catalytic subunit WDR4	45.461	7.11	14.143817	2.6699029	1	2	1
P60604	Ubiquitin-conjugating enzyme E2 G2	18.554	4.7	6.229148	9.0909091	1	1	1
P62310	U6 snRNA-associated Sm-like protein LSm3	11.838	4.7	5.2616704	11.764706	1	2	1
P62314	Small nuclear ribonucleoprotein Sm D1	13.273	11.56	8.071297	10.92437	1	1	1
P62330	ADP-ribosylation factor 6	20.069	8.95	7.545441	9.7142857	2	3	1
P63316	Troponin C, slow skeletal and cardiac muscles	18.391	4.18	3.7272304	7.4534161	1	1	1
P78345	Ribonuclease P protein subunit p38	31.815	9.92	3.3321736	3.5335689	1	1	1
P78537	Biogenesis of lysosome-related organelles complex 1 subunit 1	17.252	9.33	9.5788931	12.418301	2	3	2
Q00587	Cdc42 effector protein 1	40.27	7.15	4.1098026	2.3017903	1	1	1
Q00613	Heat shock factor protein 1	57.225	5.19	9.0305983	5.2930057	2	2	2
Q01167	Forkhead box protein K2	69.02	9.54	4.052168	2.1212121	2	2	1
Q01974	Tyrosine-protein kinase transmembrane receptor ROR2	104.69	6.55	8.6804776	1.9088017	1	1	1
Q02539	Histone H1.1	21.829	10.99	51.281078	17.209302	5	18	1
Q04656	Copper-transporting ATPase 1	163.27	6.24	11.420878	1.3333333	2	2	2
Q04912	Macrophage-stimulating protein receptor	152.174	6.55	6.4756974	1.2142857	2	2	1
Q04941	Proteolipid protein 2	16.68	7.24	6.3877461	8.5526316	1	1	1
Q06587	E3 ubiquitin-protein ligase RING1	42.403	5.62	14.602054	7.635468	2	3	1
Q12981	Vesicle transport protein SEC20	26.116	8.95	3.3438059	4.8245614	1	1	1
Q13084	39S ribosomal protein L28, mitochondrial	30.138	8.29	3.7011469	2.734375	1	1	1
Q13137	Calcium-binding and coiled-coil domain-containing protein 2	52.221	5.02	7.5637683	4.4843049	2	2	2
Q13227	G protein pathway suppressor 2	36.666	9.52	6.5783531	4.587156	2	2	2
Q13277	Syntaxin-3	33.134	5.44	3.3046059	3.1141869	1	1	1
Q13363	C-terminal-binding protein 1	47.505	6.77	28.776667	10.227273	5	8	1
Q13445	Transmembrane emp24 domain-containing protein 1	25.19	4.48	7.9284862	7.4889868	1	1	1
Q13535	Serine/threonine-protein kinase ATR	301.172	7.43	3.1909119	0.3782148	1	1	1
Q13586	Stromal interaction molecule 1	77.375	6.67	6.2015227	2.919708	2	2	2
Q13686	Alkylated DNA repair protein alkB homolog 1	43.804	7.08	3.2315839	2.0565553	1	1	1
Q13740	CD166 antigen	65.061	6.25	2.4152166	1.8867925	1	1	1
Q13751	Laminin subunit beta-3	129.489	7.21	8.1922621	1.3651877	1	1	1
Q14194	Dihydropyrimidinase-related protein 1	62.145	7.03	44.620305	9.6153846	5	8	2
Q14451	Growth factor receptor-bound protein 7	59.643	8.5	4.0286314	1.6917293	1	1	1
Q14BN4	Sarcolemmal membrane-associated protein	95.14	5.47	9.4235093	2.294686	2	2	2
Q15022	Polycomb protein SUZ12	83.003	8.81	8.5634969	1.35318	1	2	1

Q15032	R3H domain-containing protein 1	120.621	8.75	8.0268097	1.8198362	2	2	2
Q15036	Sorting nexin-17	52.868	7.46	7.7775437	2.7659574	1	1	1
Q15283	Ras GTPase-activating protein 2	96.553	7.2	5.5017225	1.5294118	2	2	2
Q15527	Surfeit locus protein 2	29.629	9.22	6.8661419	4.296875	1	1	1
Q15532	Protein SSXT	45.899	6.46	6.4609239	2.8708134	1	1	1
Q15650	Activating signal cointegrator 1	66.105	7.85	7.2679281	1.7211704	1	1	1
Q15819	Ubiquitin-conjugating enzyme E2 variant 2	16.352	8.09	40.272612	46.206897	6	12	2
Q15853	Upstream stimulatory factor 2	36.932	5.1	7.8332739	4.6242775	1	1	1
Q16254	Transcription factor E2F4	43.933	4.75	4.2208366	3.1476998	1	1	1
Q16539	Mitogen-activated protein kinase 14	41.267	5.78	9.1701473	7.5	3	3	3
Q16594	Transcription initiation factor TFIID subunit 9	28.956	8.66	5.4392338	6.8181818	2	2	2
Q16626	Male-enhanced antigen 1	19.892	4.22	7.6771607	7.027027	1	1	1
Q16644	MAP kinase-activated protein kinase 3	42.96	7.28	2.4221637	1.8324607	1	1	1
Q1ED39	Lysine-rich nucleolar protein 1	51.557	9.86	19.577412	8.0786026	3	3	3
Q2KHR3	Glutamine and serine-rich protein 1	189.856	7.08	7.9853348	0.9798271	2	2	2
Q2PZI1	Probable C-mannosyltransferase DPY19L1	77.267	8.95	6.1582652	3.1111111	1	1	1
Q2T9J0	Peroxisomal leader peptide-processing protease	59.271	6.2	10.415891	4.5936396	2	2	2
Q2TB10	Zinc finger protein 800	75.189	9.47	5.0018279	1.3554217	1	1	1
Q32P44	Echinoderm microtubule-associated protein-like 3	95.138	7.12	7.0625709	2.34375	2	2	2
Q3B726	DNA-directed RNA polymerase I subunit RPA43	37.409	6.98	9.7454229	6.8047337	2	2	2
Q3KR37	GRAM domain-containing protein 1B	85.346	6.21	7.5101821	2.303523	1	1	1
Q3SXM5	Inactive hydroxysteroid dehydrogenase-like protein 1	36.978	8.72	3.2722441	4.2424242	2	2	2
Q3ZCW2	Galectin-related protein	18.974	5.35	3.8667805	4.6511628	1	1	1
Q49AG3	Zinc finger BED domain-containing protein 5	78.861	8.27	3.596022	1.010101	1	1	1
Q4G0F5	Vacuolar protein sorting-associated protein 26B	39.13	7.36	10.139191	11.011905	3	3	3
Q52LW3	Rho GTPase-activating protein 29	141.974	6.74	3.1930065	0.7137193	1	1	1
Q53H96	Pyrroline-5-carboxylate reductase 3	28.645	7.72	13.6023	9.4890511	2	3	2
Q53HC5	Kelch-like protein 26	68.096	6.48	3.4841262	1.300813	1	1	1
Q53HC9	Protein TSSC1	43.576	5.06	2.9329291	2.3255814	1	1	1
Q53RE8	Ankyrin repeat domain-containing protein 39	19.639	6.89	4.6103025	7.6502732	1	1	1
Q53SF7	Cordon-bleu protein-like 1	131.705	6.67	7.7510464	1.3289037	1	1	1
Q5BJF6	Outer dense fiber protein 2	95.342	7.62	6.8007935	1.6887817	1	1	1
Q5H8A4	GPI ethanolamine phosphate transferase 2	108.103	7.15	4.4388986	1.4242116	1	1	1
Q5HYI7	Metaxin-3	35.071	7.8	3.5683147	2.5641026	1	1	1
Q5HYI8	Rab-like protein 3	26.407	7.11	21.529606	13.559322	2	5	2
Q5JS54	Proteasome assembly chaperone 4	13.766	6.52	2.8404328	7.3170732	1	1	1
Q5SNT2	Transmembrane protein 201	72.19	9.22	4.4038429	2.5525526	1	1	1
Q5SW96	Low density lipoprotein receptor adapter protein 1	33.864	6.7	7.2702684	5.1948052	1	1	1
Q5T0D9	Tumor protein p63-regulated gene 1-like protein	30.193	7.34	4.098106	4.0441176	1	1	1
Q5T653	39S ribosomal protein L2, mitochondrial	33.28	11.3	6.0268721	3.9344262	1	1	1
Q5T9L3	Protein wntless homolog	62.212	7.36	9.9705262	3.5120148	3	3	3
Q5T9S5	Coiled-coil domain-containing protein 18	168.857	5.66	3.5466817	0.4814305	1	1	1
Q5TAX3	Terminal uridylyltransferase 4	185.048	7.97	3.6800901	0.973236	2	2	2
Q5TKA1	Protein lin-9 homolog	61.907	9.04	7.3005959	2.7675277	1	1	1
Q5U5X0	Complex III assembly factor LYRM7	11.947	9.66	4.6746896	9.6153846	1	1	1
Q5UCC4	ER membrane protein complex subunit 10	27.33	6.13	6.8812191	3.0534351	1	2	1
Q5VT06	Centrosome-associated protein 350	350.716	6.33	6.446724	0.3208213	1	1	1

Q5VUA4	Zinc finger protein 318	250.958	7.2	3.8572978	0.6581834	1	1	1
Q5VWZ2	Lysophospholipase-like protein 1	26.299	7.84	5.7619774	5.907173	2	2	2
Q5VZ89	DENN domain-containing protein 4C	186.739	6.86	3.5238933	0.6575015	1	1	1
Q643R3	Lysophospholipid acyltransferase LPCAT4	57.183	8.97	13.548218	4.7709924	2	2	2
Q66K89	Transcription factor E4F1	83.444	6.34	5.6983191	1.9132653	1	1	1
Q68CQ7	Glycosyltransferase 8 domain-containing protein 1	41.909	9.35	4.5556429	2.6954178	1	1	1
Q68D91	Metallo-beta-lactamase domain-containing protein 2	31.352	6.92	8.0214251	4.6594982	1	2	1
Q68DH5	LMBR1 domain-containing protein 2	81.12	7.5	2.7859513	1.0071942	1	1	1
Q69YU5	Uncharacterized protein C12orf73	8.017	9.16	2.823041	9.8591549	1	1	1
Q6AZY7	Scavenger receptor class A member 3	65.096	6.54	3.7918275	1.8151815	1	1	1
Q6DKI1	60S ribosomal protein L7-like 1	28.643	10.51	8.2652454	7.7235772	2	2	2
Q6GYQ0	Ral GTPase-activating protein subunit alpha-1	229.686	6.19	11.024807	1.5225933	3	3	3
Q6IN84	rRNA methyltransferase 1, mitochondrial	38.614	7.94	4.919013	3.1161473	1	1	1
Q6IQ22	Ras-related protein Rab-12	27.231	8.41	15.155489	9.0163934	2	5	1
Q6L8Q7	2',5'-phosphodiesterase 12	67.309	6.57	11.649527	3.6124795	2	2	2
Q6NW29	RWD domain-containing protein 4	21.238	5.31	6.7401673	4.787234	1	1	1
Q6P1L5	Protein FAM117B	61.93	9.82	3.5961934	1.8675722	1	1	1
Q6P1X5	Transcription initiation factor TFIID subunit 2	136.883	8.19	4.2645608	0.6672227	1	1	1
Q6P4A7	Sideroflexin-4	37.974	9.19	2.4115042	2.6706231	1	1	1
Q6P4H8	Protein FAM173B	26.093	8.87	2.9473061	3.0042918	1	1	1
Q6P4I2	WD repeat-containing protein 73	41.659	5.78	2.3176744	2.6455026	1	1	1
Q6P6C2	RNA demethylase ALKBH5	44.229	9.09	9.0920611	7.8680203	3	3	3
Q6PH81	UPF0547 protein C16orf87	17.788	10.07	6.2240257	9.0909091	1	1	1
Q6PID8	Kelch domain-containing protein 10	49.067	9.38	3.2199709	2.0361991	1	1	1
Q6UVJ0	Spindle assembly abnormal protein 6 homolog	74.351	7.55	7.9296444	2.587519	2	2	2
Q6UWP2	Dehydrogenase/reductase SDR family member 11	28.29	6.64	2.9913998	2.6923077	1	1	1
Q6VMQ6	Activating transcription factor 7-interacting protein 1	136.31	4.7	8.8648673	1.2598425	1	1	1
Q6W2J9	BCL-6 corepressor	192.069	6.48	6.2998157	0.9116809	1	1	1
Q6ZMI0	Protein phosphatase 1 regulatory subunit 21	88.26	6.84	5.8745187	1.9230769	1	1	1
Q6ZMR3	L-lactate dehydrogenase A-like 6A	36.484	6.99	16.778546	7.2289157	3	9	1
Q6ZW31	Rho GTPase-activating protein SYDE1	79.743	8.43	3.0051991	1.6326531	1	1	1
Q709F0	Acyl-CoA dehydrogenase family member 11	87.228	8.12	3.227385	1.1538462	1	1	1
Q7L1V2	Vacuolar fusion protein MON1 homolog B	59.18	6.29	6.5410601	2.3765996	1	1	1
Q7L4I2	Arginine/serine-rich coiled-coil protein 2	50.529	11.33	7.3682519	2.764977	1	1	1
Q7L8W6	Diphthine--ammonia ligase	30.287	5.4	5.8196447	6.741573	2	2	2
Q7LBR1	Charged multivesicular body protein 1b	22.095	8.1	6.4821116	8.5427136	2	2	2
Q7LG56	Ribonucleoside-diphosphate reductase subunit M2 B	40.71	4.97	5.6874457	4.8433048	2	2	1
Q7Z2K8	G protein-regulated inducer of neurite outgrowth 1	102.336	8.06	3.4113922	1.1904762	1	1	1
Q7Z3E2	Coiled-coil domain-containing protein 186	103.623	6.27	4.845576	1.2249443	1	1	1
Q7Z3J2	UPF0505 protein C16orf62	109.492	7.21	15.538351	2.2845275	2	2	2
Q7Z3V4	Ubiquitin-protein ligase E3B	123.019	8.19	5.2007284	0.9363296	1	1	1
Q7Z4G4	tRNA (guanine(10)-N2)-methyltransferase homolog	53.387	7.78	9.7423541	4.1036717	2	2	2
Q7Z6K3	Protein prenyltransferase alpha subunit repeat-containing protein 1	46.376	6.99	3.6259853	2.4875622	1	1	1
Q86SF2	N-acetylgalactosaminyltransferase 7	75.341	7.11	3.0367317	1.217656	1	1	1
Q86SQ7	Serologically defined colon cancer antigen 8	82.631	5.81	4.0371573	1.2622721	1	1	1
Q86SZ2	Trafficking protein particle complex subunit 6B	17.971	8.68	3.8437538	5.0632911	1	1	1
Q86T24	Transcriptional regulator Kaiso	74.438	5.12	5.3894831	2.3809524	2	2	2

Q86TJ2	Transcriptional adapter 2-beta	48.44	7.83	4.6221476	2.6190476	1	1	1
Q86U28	Iron-sulfur cluster assembly 2 homolog, mitochondrial	16.466	5.25	4.5676722	7.7922078	1	1	1
Q86UA1	Pre-mRNA-processing factor 39	78.381	5.4	5.9621752	2.2421525	1	1	1
Q86UB9	Transmembrane protein 135	52.256	9.45	2.9531148	1.7467249	1	1	1
Q86US8	Telomerase-binding protein EST1A	160.362	7.05	2.8273971	0.4933051	1	1	1
Q86UT6	NLR family member X1	107.548	7.37	11.591192	2.6666667	2	2	2
Q86UT8	Coiled-coil domain-containing protein 84	37.95	8.22	10.072187	10.542169	3	3	3
Q86WG5	Myotubularin-related protein 13	208.333	7.06	5.6724367	0.8112493	1	1	1
Q86WX3	Active regulator of SIRT1	15.424	10.74	3.8800846	9.5588235	1	1	1
Q86XK3	Swi5-dependent recombination DNA repair protein 1 homolog	28.244	6.39	7.1195294	5.3061224	1	1	1
Q86YN1	Dolichyldiphosphatase 1	27.013	9.44	2.9884296	2.9411765	1	1	1
Q8IUC8	Polypeptide N-acetylgalactosaminyltransferase 13	64.009	6.83	12.784818	2.8776978	2	3	1
Q8IUH4	Palmitoyltransferase ZDHHC13	70.814	8.07	3.4683934	1.607717	1	1	1
Q8IVL6	Prolyl 3-hydroxylase 3	81.786	6.32	4.6544302	1.4945652	1	1	1
Q8IWF2	FAD-dependent oxidoreductase domain-containing protein 2	77.743	7.72	2.7721133	1.3157895	1	1	1
Q8IWI9	MAX gene-associated protein	331.631	6.79	3.1665979	0.2643754	1	1	1
Q8IWL3	Iron-sulfur cluster co-chaperone protein HscB, mitochondrial	27.405	7.69	6.003357	3.8297872	1	1	1
Q8IWR0	Zinc finger CCCH domain-containing protein 7A	110.466	7.3	15.905122	4.1194645	4	4	4
Q8IWU2	Serine/threonine-protein kinase LMTK2	164.799	4.48	5.4555599	0.5988024	1	1	1
Q8IWV7	E3 ubiquitin-protein ligase UBR1	200.08	6.01	13.992087	1.6580903	2	2	2
Q8IWV8	E3 ubiquitin-protein ligase UBR2	200.408	6.24	10.226067	0.7407407	1	1	1
Q8IXI2	Mitochondrial Rho GTPase 1	70.739	6.27	3.8671002	1.4563107	1	1	1
Q8IXJ6	NAD-dependent protein deacetylase sirtuin-2	43.154	5.36	4.9321855	3.0848329	1	1	1
Q8IY47	Kelch repeat and BTB domain-containing protein 2	71.284	5.62	3.5704092	1.6051364	1	1	1
Q8IYS2	Uncharacterized protein KIAA2013	69.113	8.19	5.8353498	2.681388	1	1	1
Q8IZ83	Aldehyde dehydrogenase family 16 member A1	85.074	6.79	6.0102337	2.4937656	2	2	2
Q8IZT6	Abnormal spindle-like microcephaly-associated protein	409.54	10.45	6.2110543	0.3738855	1	1	1
Q8N129	Protein canopy homolog 4	28.292	4.64	12.265211	10.080645	2	3	2
Q8N1G0	Zinc finger protein 687	129.446	8.19	3.5512937	0.6467259	1	1	1
Q8N1S5	Zinc transporter ZIP11	35.373	5.6	4.2109837	2.9239766	1	1	1
Q8N2K0	Monoacylglycerol lipase ABHD12	45.068	8.65	4.9172146	2.5125628	1	1	1
Q8N2M8	CLK4-associating serine/arginine rich protein	77.115	10.45	3.0152927	1.1869436	1	1	1
Q8N3R9	MAGUK p55 subfamily member 5	77.246	6.14	4.0370154	1.1851852	1	1	1
Q8N490	Probable hydrolase PNKD	42.849	9.09	8.5949355	4.4155844	2	2	2
Q8N584	Tetratricopeptide repeat protein 39C	65.828	6.99	2.5045944	1.5437393	1	1	1
Q8N6S5	ADP-ribosylation factor-like protein 6-interacting protein 6	24.66	6.33	6.3209357	5.3097345	1	1	1
Q8N8I0	Sterile alpha motif domain-containing protein 12	22.892	9.33	5.5825283	6.9651741	1	1	1
Q8N8R3	Mitochondrial basic amino acids transporter	32.042	8.75	4.2509597	2.970297	1	1	1
Q8N954	G patch domain-containing protein 11	30.18	5.24	21.798437	8.8803089	2	4	2
Q8N999	Uncharacterized protein C12orf29	37.466	7.06	4.0480799	3.0769231	1	1	1
Q8NB15	Zinc finger protein 511	29.409	6.79	5.7269987	4.5801527	1	1	1
Q8NBJ4	Golgi membrane protein 1	45.306	4.97	10.486632	6.4837905	3	3	3
Q8ND24	RING finger protein 214	77.619	6.95	7.3323395	2.7027027	2	3	2
Q8NDI1	EH domain-binding protein 1	139.931	5.35	13.406241	2.8432169	4	4	3
Q8NEW0	Zinc transporter 7	41.599	6.95	4.5785606	2.9255319	1	1	1
Q8NFB2	Transmembrane protein 185A	40.603	7.24	4.3448616	2.5714286	1	1	1
Q8NFZ5	TNFAIP3-interacting protein 2	48.669	6.44	6.0790423	2.3310023	1	1	1

Q8NFZ8	Cell adhesion molecule 4	42.759	6.3	12.503624	4.3814433	1	1	1
Q8NHQ9	ATP-dependent RNA helicase DDX55	68.503	9.25	6.7124159	2.5	2	2	2
Q8NI35	InaD-like protein	196.247	4.94	3.0615303	0.7218212	1	1	1
Q8NI77	Kinesin-like protein KIF18A	102.216	8.91	6.6117595	1.7817372	2	2	1
Q8TB52	F-box only protein 30	82.251	5.4	3.4377071	0.9395973	1	1	1
Q8TBC3	SH3KBP1-binding protein 1	76.296	8.28	15.795177	5.2333805	3	3	3
Q8TBE7	Solute carrier family 35 member G2	46.391	7.34	2.9842212	2.184466	1	1	1
Q8TBE9	N-acylneuraminate-9-phosphatase	27.795	6.4	7.1342448	4.8387097	1	1	1
Q8TBN0	Guanine nucleotide exchange factor for Rab-3A	42.61	6.47	13.322111	8.9005236	2	2	2
Q8TDH9	Biogenesis of lysosome-related organelles complex 1 subunit 5	21.596	7.59	5.0340176	8.0213904	2	2	1
Q8TDR0	TRAF3-interacting protein 1	78.584	7.93	8.3586251	1.5918958	1	1	1
Q8TE04	Pantothenate kinase 1	64.298	7.56	5.6028543	3.1772575	2	2	2
Q8TEA1	Putative methyltransferase NSUN6	51.737	7.96	9.1906224	4.0511727	2	2	2
Q8TEH3	DENN domain-containing protein 1A	110.508	6.96	5.1904713	2.0812686	2	2	2
Q8TEL6	Short transient receptor potential channel 4-associated protein	90.794	7.61	5.9229957	1.7565872	1	1	1
Q8TF01	Arginine/serine-rich protein PNISR	92.521	10.02	4.8874252	1.863354	2	2	2
Q8TF05	Serine/threonine-protein phosphatase 4 regulatory subunit 1	106.936	4.77	5.4655931	1.1578947	1	1	1
Q8WU79	Stromal membrane-associated protein 2	46.755	8.87	3.9792245	3.030303	1	1	1
Q8WVK2	U4/U6.U5 small nuclear ribonucleoprotein 27 kDa protein	18.849	11.62	4.7642191	9.6774194	1	1	1
Q8WWX9	Selenoprotein M	16.222	5.54	3.7830428	9.6551724	1	1	1
Q8WZA0	Protein LZIC	21.481	4.94	6.3317866	9.4736842	2	2	2
Q92466	DNA damage-binding protein 2	47.833	9.47	9.12088	5.8548009	2	2	2
Q92558	Wiskott-Aldrich syndrome protein family member 1	61.614	6.46	5.2885302	3.0411449	1	1	1
Q92604	Acyl-CoA:lysophosphatidylglycerol acyltransferase 1	43.061	8.92	3.9090369	2.1621622	1	1	1
Q92609	TBC1 domain family member 5	88.949	6.54	2.5282683	1.509434	1	1	1
Q92667	A-kinase anchor protein 1, mitochondrial	97.281	4.94	5.0629337	0.9966777	1	1	1
Q92833	Protein Jumonji	138.648	9.38	4.2827457	1.2038523	1	1	1
Q92889	DNA repair endonuclease XPF	104.42	6.93	14.983519	4.1484716	4	4	4
Q93074	Mediator of RNA polymerase II transcription subunit 12	242.928	7.05	9.5925077	0.7808911	2	2	2
Q969H4	Connector enhancer of kinase suppressor of ras 1	79.656	5.38	2.6411138	0.9722222	1	1	1
Q969M3	Protein YIPF5	27.971	4.36	3.4810914	3.5019455	1	1	1
Q969S9	Ribosome-releasing factor 2, mitochondrial	86.546	6.51	14.630991	3.465982	2	2	2
Q969T3	Sorting nexin-21	41.34	5.24	6.7110804	4.0214477	1	1	1
Q969T7	7-methylguanosine phosphate-specific 5'-nucleotidase	34.367	6.38	7.6056147	3	1	2	1
Q969Z3	Mitochondrial amidoxime reducing component 2	37.999	9.16	25.04457	6.5671642	2	5	1
Q96A26	Protein FAM162A	17.331	9.77	6.7830428	11.038961	1	1	1
Q96AJ9	Vesicle transport through interaction with t-SNAREs homolog 1A	25.202	6.4	6.6720203	4.1474654	1	2	1
Q96AX1	Vacuolar protein sorting-associated protein 33A	67.568	6.96	4.5238933	1.6778523	1	1	1
Q96B23	Uncharacterized protein C18orf25	43.298	4.88	5.3486251	2.9776675	1	1	1
Q96BD0	Solute carrier organic anion transporter family member 4A1	77.143	7.83	3.5415132	1.1080332	1	1	1
Q96BI3	Gamma-secretase subunit APH-1A	28.977	7.9	9.1522423	5.2830189	1	1	1
Q96BM9	ADP-ribosylation factor-like protein 8A	21.402	7.77	28.183366	23.655914	4	6	1
Q96BP2	Coiled-coil-helix-coiled-coil-helix domain-containing protein 1	13.466	10.21	4.6558043	9.3220339	1	1	1
Q96CB8	Integrator complex subunit 12	48.777	9.69	2.7825161	2.3809524	1	1	1
Q96CN5	Leucine-rich repeat-containing protein 45	75.905	6.23	5.2108425	1.7910448	1	1	1
Q96CN9	GRIP and coiled-coil domain-containing protein 1	87.757	5.45	3.0054189	0.9032258	1	1	1
Q96CP6	GRAM domain-containing protein 1A	80.63	6.74	2.9347939	1.3812155	1	1	1

Q96CW5	Gamma-tubulin complex component 3	103.506	8.12	12.323108	2.646086	2	2	2
Q96D53	AarF domain-containing protein kinase 4	60.031	7.23	4.4193031	1.6544118	1	1	1
Q96DC8	Enoyl-CoA hydratase domain-containing protein 3, mitochondrial	32.613	8.65	7.3576344	3.960396	1	1	1
Q96EK4	THAP domain-containing protein 11	34.433	8.98	5.9519468	2.866242	1	1	1
Q96EK9	Protein KTI12 homolog	38.592	6.95	13.732019	6.4971751	2	2	2
Q96EL2	28S ribosomal protein S24, mitochondrial	19.003	9.38	2.4917397	4.7904192	1	1	1
Q96EP0	E3 ubiquitin-protein ligase RNF31	119.575	6.57	12.089399	2.1455224	2	2	2
Q96ES7	SAGA-associated factor 29 homolog	33.217	8.1	5.7191941	4.4368601	1	1	1
Q96ET8	Golgi apparatus membrane protein TVP23 homolog C	31.085	9.29	4.222066	3.6231884	1	1	1
Q96EX3	WD repeat-containing protein 34	57.764	6.64	3.8074325	2.0522388	1	1	1
Q96F45	Zinc finger protein 503	62.516	8.7	6.7700623	1.8575851	1	1	1
Q96FS4	Signal-induced proliferation-associated protein 1	112.08	6.6	5.3589227	1.9193858	1	1	1
Q96G01	Protein bicaudal D homolog 1	110.682	5.81	10.959944	2.0512821	2	2	1
Q96G25	Mediator of RNA polymerase II transcription subunit 8	29.062	7.44	8.397401	8.2089552	2	2	2
Q96G28	Cilia- and flagella-associated protein 36	39.422	4.97	7.1592041	4.3859649	1	1	1
Q96GQ5	RUS1 family protein C16orf58	50.985	6.93	3.9593977	1.9230769	1	1	1
Q96HJ9	UPF0562 protein C7orf55	12.741	10.07	4.6062494	10.619469	1	1	1
Q96HN2	Adenosylhomocysteinase 3	66.678	7.36	41.639789	12.111293	8	10	1
Q96HV5	Transmembrane protein 41A	29.646	9.03	4.6931463	4.1666667	1	1	1
Q96IY1	Kinetochore-associated protein NSL1 homolog	32.141	6.79	5.2461872	5.3380783	1	1	1
Q96J02	E3 ubiquitin-protein ligase Itchy homolog	102.738	6.3	5.5783961	0.9966777	1	1	1
Q96JB2	Conserved oligomeric Golgi complex subunit 3	94.036	5.57	10.612286	3.0193237	2	2	2
Q96K37	Solute carrier family 35 member E1	44.744	9.79	3.823041	3.1707317	1	1	1
Q96KC8	DnaJ homolog subfamily C member 1	63.843	8.63	3.2754784	1.9855596	1	1	1
Q96KM6	Zinc finger protein 512B	97.202	9.83	3.155958	1.4573991	1	1	1
Q96MF7	E3 SUMO-protein ligase NSE2	27.915	7.74	3.8315025	4.4534413	1	1	1
Q96MH2	Protein HEXIM2	32.399	6.55	2.5568935	2.4475524	1	1	1
Q96MX6	WD repeat-containing protein 92	39.715	8.09	5.6374907	4.4817927	2	2	2
Q96PU4	E3 ubiquitin-protein ligase UHRF2	89.928	8.21	9.3181489	2.3690773	2	2	1
Q96QU8	Exportin-6	128.801	6.35	4.1799957	0.9777778	1	1	1
Q96RU2	Ubiquitin carboxyl-terminal hydrolase 28	122.414	5.2	7.8809483	1.8570102	2	2	2
Q96S19	UPF0585 protein C16orf13	22.564	7.83	13.228529	12.254902	2	3	2
Q96S44	TP53-regulating kinase	28.143	9.54	15.035701	10.27668	3	3	3
Q96SI1	BTB/POZ domain-containing protein KCTD15	31.922	7.44	2.4792545	2.8268551	1	1	1
Q96SN8	CDK5 regulatory subunit-associated protein 2	214.905	5.58	11.259486	1.5319599	4	7	2
Q96T52	Mitochondrial inner membrane protease subunit 2	19.705	9.36	3.1629801	5.7142857	1	1	1
Q99640	mbrane-associated tyrosine- and threonine-specific cdc2-inhibitory kin	54.487	6.93	7.4734639	3.6072144	2	2	2
Q99643	Succinate dehydrogenase cytochrome b560 subunit, mitochondrial	18.598	9.69	5.2370365	4.7337278	1	2	1
Q99735	Microsomal glutathione S-transferase 2	16.61	9.55	3.4292236	9.5238095	1	1	1
Q99808	Equilibrative nucleoside transporter 1	50.186	8.29	8.6442412	3.7280702	2	2	2
Q99943	1-acyl-sn-glycerol-3-phosphate acyltransferase alpha	31.696	9.38	2.5551748	3.180212	1	1	1
Q9BPY3	Protein FAM118B	39.474	5.99	9.9509994	5.982906	2	2	2
Q9BQ04	RNA-binding protein 4B	40.124	6.74	65.113378	27.019499	9	18	1
Q9BQ75	Protein CMSS1	31.864	9.19	11.176819	5.734767	2	3	1
Q9BQD7	Protein FAM173A	25.114	9.32	4.8471004	8.0851064	1	1	1
Q9BQL6	Fermitin family homolog 1	77.387	6.28	12.682705	2.9542097	2	4	1
Q9BR61	Acyl-CoA-binding domain-containing protein 6	31.131	5.11	3.6245193	3.1914894	1	1	1

Q9BRA0	N-alpha-acetyltransferase 38, NatC auxiliary subunit	13.506	5.53	3.5306199	7.2	1	1	1
Q9BRQ0	Pygopus homolog 2	41.218	7.28	8.59176	5.1724138	1	1	1
Q9BRT3	Migration and invasion enhancer 1	12.395	4.37	5.9977248	15.652174	2	2	2
Q9BRT6	Protein LLP homolog	15.215	10.37	15.904846	10.852713	1	2	1
Q9BSB4	Autophagy-related protein 101	24.987	6.15	5.9492337	5.5045872	1	1	1
Q9BSG0	Protease-associated domain-containing protein 1	21.029	5.47	3.3279021	4.787234	1	1	1
Q9BTY2	Plasma alpha-L-fucosidase	54.032	6.25	4.2669633	1.7130621	1	1	1
Q9BTZ2	Dehydrogenase/reductase SDR family member 4	29.518	8.56	12.709446	8.6330935	3	3	3
Q9BU61	ADH dehydrogenase [ubiquinone] 1 alpha subcomplex assembly factor	20.337	8.22	5.8904215	7.6086957	1	1	1
Q9BUW7	UPF0184 protein C9orf16	9.048	4.22	6.8551146	19.277108	1	1	1
Q9BUZ4	TNF receptor-associated factor 4	53.507	8.15	2.4231981	1.4893617	1	1	1
Q9BVC4	Target of rapamycin complex subunit LST8	35.853	5.86	3.638461	3.0674847	1	1	1
Q9BVG8	Kinesin-like protein KIFC3	92.718	7.69	4.7604503	1.0804322	1	1	1
Q9BVJ7	Dual specificity protein phosphatase 23	16.578	8.21	5.8607508	7.3333333	1	1	1
Q9BVQ7	Spermatogenesis-associated protein 5-like protein 1	80.66	8.09	19.847808	3.187251	2	3	2
Q9BW62	Katanin p60 ATPase-containing subunit A-like 1	55.357	6.74	7.4483556	3.6734694	2	2	2
Q9BWS9	Chitinase domain-containing protein 1	44.912	8.63	17.317679	7.8880407	2	2	2
Q9BXL6	Caspase recruitment domain-containing protein 14	113.198	5.92	16.335255	1.5936255	1	2	1
Q9BXP2	Solute carrier family 12 member 9	96.049	8.07	4.5675117	1.3129103	1	1	1
Q9BXS9	Solute carrier family 26 member 6	82.914	8.37	4.5460704	1.1857708	1	1	1
Q9C0B7	Transport and Golgi organization protein 6 homolog	120.671	6.11	3.1056285	0.7312614	1	1	1
Q9GZM5	Protein YIPF3	38.223	5.76	3.2747419	2.5714286	1	1	1
Q9H0G5	Nuclear speckle splicing regulatory protein 1	66.35	8.84	9.8156356	3.5842294	2	2	2
Q9H0X9	Oxysterol-binding protein-related protein 5	98.555	7.47	2.8362425	1.2514221	1	1	1
Q9H1I8	Activating signal cointegrator 1 complex subunit 2	86.306	5.16	8.4429047	3.4346103	2	2	2
Q9H300	Presenilins-associated rhomboid-like protein, mitochondrial	42.163	9.79	5.278107	3.1662269	1	1	1
Q9H3H5	cetylglucosamine--dolichyl-phosphate N-acetylglucosaminephosphotr	46.059	8	2.7582046	1.9607843	1	1	1
Q9H3K6	BolA-like protein 2	10.11	6.52	11.498353	30.232558	2	3	2
Q9H446	RWD domain-containing protein 1	27.922	4.2	3.5218666	3.7037037	1	1	1
Q9H4F8	SPARC-related modular calcium-binding protein 1	48.132	8.22	4.9867413	1.843318	1	1	1
Q9H582	Zinc finger protein 644	149.471	8.16	3.1572036	0.6782216	1	1	1
Q9H5U6	Zinc finger CCHC domain-containing protein 4	58.971	8.75	2.8124793	1.1695906	1	1	1
Q9H5Z1	Probable ATP-dependent RNA helicase DHX35	78.86	8.59	5.7117508	1.7069701	1	1	1
Q9H6L4	Armadillo repeat-containing protein 7	21.91	6.09	5.5673513	7.0707071	1	1	1
Q9H6R6	Palmitoyltransferase ZDHHC6	47.631	8.47	6.007049	2.905569	1	1	1
Q9H6U6	Breast carcinoma-amplified sequence 3	101.172	6.7	5.5715412	1.1853448	1	1	1
Q9H6Y7	E3 ubiquitin-protein ligase RNF167	38.274	5.63	2.3572388	2	1	1	1
Q9H7C4	Syncoilin	55.266	4.61	5.5260753	2.4896266	1	1	1
Q9H7E9	UPF0488 protein C8orf33	24.977	9.95	9.5672037	12.227074	2	2	2
Q9H7L9	Sin3 histone deacetylase corepressor complex component SDS3	38.112	5.66	6.2297373	3.0487805	1	1	1
Q9H840	Gem-associated protein 7	14.528	7.4	9.7833064	20.610687	1	1	1
Q9H857	5'-nucleotidase domain-containing protein 2	60.68	6.77	3.7356545	2.1153846	1	1	1
Q9H871	Protein RMD5 homolog A	43.964	6.06	14.694398	9.2071611	3	3	3
Q9H967	WD repeat-containing protein 76	69.725	9.25	3.8227522	1.2779553	1	1	1
Q9HA92	dical S-adenosyl methionine domain-containing protein 1, mitochondr	48.683	7.78	2.4342702	1.8099548	1	1	1
Q9HB19	Pleckstrin homology domain-containing family A member 2	47.225	8.66	29.908812	12.705882	5	5	5
Q9HBH5	Retinol dehydrogenase 14	36.841	8.79	13.532324	11.904762	4	4	4

Q9HCD6	Protein TANC2	219.512	8.07	5.7589518	0.5527638	1	1	1
Q9HCG8	Pre-mRNA-splicing factor CWC22 homolog	105.402	7.03	3.5395532	0.9911894	1	1	1
Q9HCM4	Band 4.1-like protein 5	81.805	6.58	6.2292952	1.6371078	1	1	1
Q9HD23	Magnesium transporter MRS2 homolog, mitochondrial	50.285	6.87	8.9278038	4.2889391	2	2	2
Q9HD26	Golgi-associated PDZ and coiled-coil motif-containing protein	50.489	5.92	19.12824	7.5757576	3	3	3
Q9HDC5	Junctophilin-1	71.642	9.32	3.5691191	1.5128593	1	1	1
Q9NQT8	Kinesin-like protein KIF13B	202.663	5.88	15.548072	1.5334064	2	3	1
Q9NQZ2	Something about silencing protein 10	54.525	5.62	13.289302	2.9227557	2	2	2
Q9NRG1	Phosphoribosyltransferase domain-containing protein 1	25.657	6.15	10.059184	8	2	2	2
Q9NRL3	Striatin-4	80.546	5.4	14.804196	6.1088977	4	4	3
Q9NRQ5	Single-pass membrane and coiled-coil domain-containing protein 4	6.734	10.29	3.0776379	13.559322	1	1	1
Q9NRZ7	1-acyl-sn-glycerol-3-phosphate acyltransferase gamma	43.353	8.72	5.9779843	3.1914894	1	1	1
Q9NSD4	Zinc finger protein 275	48.412	9.09	5.3482376	3.030303	1	1	1
Q9NSU2	Three-prime repair exonuclease 1	38.898	8.41	5.4627896	5.6910569	2	2	2
Q9NTG7	NAD-dependent protein deacetylase sirtuin-3, mitochondrial	43.546	8.7	5.0774302	2.7568922	1	1	1
Q9NTM9	Copper homeostasis protein cutC homolog	29.322	8.18	8.3267894	9.1575092	2	2	2
Q9NUP7	tRNA:m(4)X modification enzyme TRM13 homolog	54.213	8.09	7.0896424	2.0790021	1	1	1
Q9NUP9	Protein lin-7 homolog C	21.82	8.43	17.711214	6.5989848	1	2	1
Q9NUQ3	Gamma-taxilin	60.548	7.52	32.1305	9.8484848	4	5	3
Q9NUY8	TBC1 domain family member 23	78.272	5.41	5.452182	2.1459227	2	2	2
Q9NVA1	Ubiquinol-cytochrome-c reductase complex assembly factor 1	34.578	8.91	2.8195874	3.0100334	1	1	1
Q9NVN8	Guanine nucleotide-binding protein-like 3-like protein	65.532	8.44	3.638839	1.8900344	1	1	1
Q9NVU0	DNA-directed RNA polymerase III subunit RPC5	79.847	6.47	7.5752817	1.9774011	1	1	1
Q9NVX7	Kelch repeat and BTB domain-containing protein 4	58.107	5.67	5.8863908	2.1235521	1	1	1
Q9NWM3	CUE domain-containing protein 1	42.232	5.68	2.4988038	2.0725389	1	1	1
Q9NWQ9	Uncharacterized protein C14orf119	15.999	4.94	3.9359166	5.7142857	1	1	1
Q9NWS8	Required for meiotic nuclear division protein 1 homolog	51.571	8.69	13.927461	6.9042316	3	3	3
Q9NWT6	Hypoxia-inducible factor 1-alpha inhibitor	40.26	5.57	9.3568589	8.5959885	3	3	3
Q9NWV4	UPF0587 protein C1orf123	18.037	5.01	4.0145286	5	1	1	1
Q9NX01	Thioredoxin-like protein 4B	17.004	6	2.9767475	6.7114094	1	1	1
Q9NX24	H/ACA ribonucleoprotein complex subunit 2	17.19	8.22	6.8738686	6.5359477	1	1	1
Q9NXH8	Torsin-4A	46.885	9.94	3.4343883	1.891253	1	1	1
Q9NZ09	Ubiquitin-associated protein 1	55.048	5.11	6.3623102	3.5856574	1	1	1
Q9NZC9	d matrix-associated actin-dependent regulator of chromatin subfamily	105.872	9.06	4.1944991	1.1530398	1	1	1
Q9P003	Protein cornichon homolog 4	16.082	6.65	7.6201508	14.388489	1	1	1
Q9P0R6	GSK3-beta interaction protein	15.638	4.48	8.7382615	7.9136691	1	1	1
Q9P0S2	ytochrome c oxidase assembly protein COX16 homolog, mitochondri	12.285	9.5	2.9554602	13.207547	1	1	1
Q9P0T7	Transmembrane protein 9	20.56	6.65	4.5319482	7.1038251	1	1	1
Q9P275	Ubiquitin carboxyl-terminal hydrolase 36	122.575	9.67	24.09978	3.4790366	4	4	4
Q9P2D1	Chromodomain-helicase-DNA-binding protein 7	335.717	6.34	13.761719	0.7674341	2	3	1
Q9P2D3	HEAT repeat-containing protein 5B	224.159	7.17	12.753781	1.6900048	3	3	3
Q9P2Y5	UV radiation resistance-associated gene protein	78.102	8.29	2.6942188	1.0014306	1	1	1
Q9UBB6	Neurochondrin	78.814	5.48	14.920431	3.9780521	2	2	2
Q9UBK9	Protein UXT	18.235	7.59	4.302422	7.0063694	1	1	1
Q9UBN7	Histone deacetylase 6	131.336	5.3	3.8147412	0.7407407	1	1	1
Q9UGJ1	Gamma-tubulin complex component 4	76.041	6.65	15.629485	3.7481259	2	2	2
Q9UGQ3	Solute carrier family 2, facilitated glucose transporter member 6	54.504	8.63	3.3949105	1.9723866	1	1	1

Q9UHB4	NADPH-dependent diflavin oxidoreductase 1	66.72	6.35	2.507519	1.8425461	1	1	1
Q9UHN6	Transmembrane protein 2	154.276	8.15	2.3077702	0.5061461	1	1	1
Q9UHQ4	B-cell receptor-associated protein 29	28.302	9.54	13.455629	9.9585062	3	3	3
Q9UI26	Importin-11	112.463	5.25	4.8938332	1.9487179	2	2	2
Q9UI95	Mitotic spindle assembly checkpoint protein MAD2B	24.319	6.52	3.3612113	3.7914692	1	1	1
Q9UJ68	Mitochondrial peptide methionine sulfoxide reductase	26.116	8.09	6.1909225	3.4042553	1	2	1
Q9UJG1	Motile sperm domain-containing protein 1	24.071	7.78	5.6039751	5.1643192	1	1	1
Q9UJM8	Hydroxyacid oxidase 1	40.898	8.09	2.3928668	1.8918919	1	1	1
Q9UJX6	Anaphase-promoting complex subunit 2	93.769	5.22	9.6925519	2.676399	2	2	2
Q9UK73	Protein fem-1 homolog B	70.22	6.61	2.9515582	1.5948963	1	1	1
Q9UKA4	A-kinase anchor protein 11	210.38	5.39	4.1453904	0.5260389	1	1	1
Q9UKD1	Glucocorticoid modulatory element-binding protein 2	56.386	5.6	2.4887853	1.509434	1	1	1
Q9UKU7	Isobutyryl-CoA dehydrogenase, mitochondrial	45.04	7.85	5.6987529	3.373494	1	1	1
Q9UL03	Integrator complex subunit 6	100.326	8.62	8.0303977	2.2547914	2	2	2
Q9UL54	Serine/threonine-protein kinase TAO2	138.166	7.27	5.3489159	1.0526316	1	1	1
Q9ULC3	Ras-related protein Rab-23	26.643	6.6	4.2875187	6.7510549	1	1	1
Q9ULJ8	Neurabin-1	123.267	5.1	4.2481822	0.9107468	1	1	1
Q9ULM6	CCR4-NOT transcription complex subunit 6	63.266	7.27	5.4682657	1.7953321	1	1	1
Q9ULT0	Tetratricopeptide repeat protein 7A	96.123	6.46	4.7853305	1.6317016	2	2	2
Q9UPN6	Protein SCAF8	140.433	8.29	4.1774397	1.0228167	1	1	1
Q9UPQ3	rf-GAP with GTPase, ANK repeat and PH domain-containing protein	94.411	7.94	5.4021069	1.6336056	2	2	1
Q9UPY5	Cystine/glutamate transporter	55.387	9.19	4.1709537	1.7964072	1	1	1
Q9UQC2	GRB2-associated-binding protein 2	74.411	8.31	5.031377	1.6272189	1	1	1
Q9Y217	Myotubularin-related protein 6	71.922	7.66	4	1.7713366	1	1	1
Q9Y221	60S ribosome subunit biogenesis protein NIP7 homolog	20.449	8.51	7.0250543	10.555556	2	2	2
Q9Y251	Heparanase	61.11	9.28	3.3565473	1.6574586	1	1	1
Q9Y2G5	GDP-fucose protein O-fucosyltransferase 2	49.944	6.6	8.8979095	3.7296037	1	1	1
Q9Y2H2	Phosphatidylinositide phosphatase SAC2	128.326	7.02	3.3174939	0.8833922	1	1	1
Q9Y2I8	WD repeat-containing protein 37	54.632	7.23	3.0065197	1.8218623	1	1	1
Q9Y2R0	Cytochrome c oxidase assembly factor 3 homolog, mitochondrial	11.724	9.6	11.453279	26.415094	3	3	3
Q9Y2U9	Kelch domain-containing protein 2	46.069	6.61	4.5705707	2.7093596	1	1	1
Q9Y2X7	ARF GTPase-activating protein GIT1	84.289	6.8	5.8122371	2.2339028	2	2	1
Q9Y316	Protein MEMO1	33.711	7.14	10.473432	7.7441077	2	2	2
Q9Y324	rRNA-processing protein FCF1 homolog	23.354	9.7	6.6169031	5.5555556	1	1	1
Q9Y375	Complex I intermediate-associated protein 30, mitochondrial	37.74	7.64	7.1402015	4.8929664	1	1	1
Q9Y388	RNA-binding motif protein, X-linked 2	37.313	9.83	4.9519468	2.7950311	1	1	1
Q9Y3B9	RRP15-like protein	31.465	5.52	10.558045	9.929078	3	3	3
Q9Y3D3	28S ribosomal protein S16, mitochondrial	15.335	9.5	3.1119891	5.1094891	1	1	1
Q9Y3E7	Charged multivesicular body protein 3	25.057	5.12	2.9292235	3.1531532	1	1	1
Q9Y3P4	Rhomboid domain-containing protein 3	40.458	6.58	4.6641411	3.1088083	1	1	1
Q9Y3S2	Zinc finger protein 330	36.177	6.16	13.290074	9.0625	3	3	3
Q9Y483	Metal-response element-binding transcription factor 2	67.063	8.72	4.5639965	1.8549747	1	1	1
Q9Y496	Kinesin-like protein KIF3A	79.991	6.54	11.940818	3.8626609	3	6	1
Q9Y4D8	Probable E3 ubiquitin-protein ligase HECTD4	439.065	6.19	3.5723516	0.2002002	1	1	1
Q9Y4K0	Lysyl oxidase homolog 2	86.668	6.38	3.6245193	1.1627907	1	1	1
Q9Y5N5	HemK methyltransferase family member 2	22.886	5.21	3.1554607	4.2056075	1	1	1
Q9Y5T4	DnaJ homolog subfamily C member 15	16.373	10.08	10.861681	15.333333	2	2	2

Q9Y5U2	Protein TSSC4	34.305	5.19	4.0145286	3.9513678	1	1	1
Q9Y5Z0	Beta-secretase 2	56.145	5.15	4.6542343	2.1235521	1	1	1
Q9Y5Z9	UbiA prenyltransferase domain-containing protein 1	36.808	8.15	9.1091035	2.9585799	1	2	1
Q9Y6A1	Protein O-mannosyl-transferase 1	84.826	8.4	6.4197595	1.6064257	1	1	1
Q9Y6A9	Signal peptidase complex subunit 1	11.797	9.31	5.7654827	16.666667	1	1	1
Q9Y6J9	NA polymerase II p300/CBP-associated factor-associated factor 65 kD	67.772	8.97	4.1052963	1.7684887	1	1	1
