# Supplementary figures and images for: Hypoxia-Induced LIN28A mRNA Promotes the Metastasis of Colon Cancer in a Protein-Coding-Independent Manner (part 1 of 2)
Source: Front Cell Dev Biol. 2021 Feb 16;9:642930. doi: 10.3389/fcell.2021.642930 (PMC7921329; doi:10.3389/fcell.2021.642930)

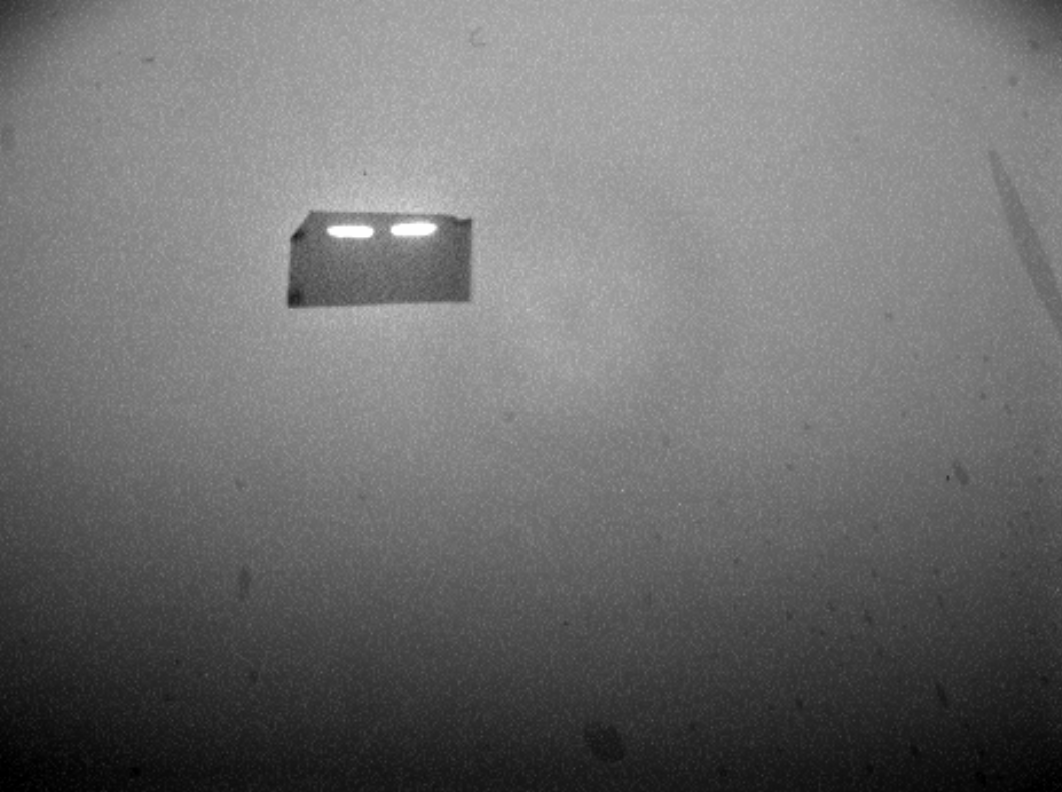

Supplement: Supplementary file 3 [file Data_Sheet_3.ZIP › FIG-1C/HCT116/membrane with marker-HCT116 -ACTIN.tif]

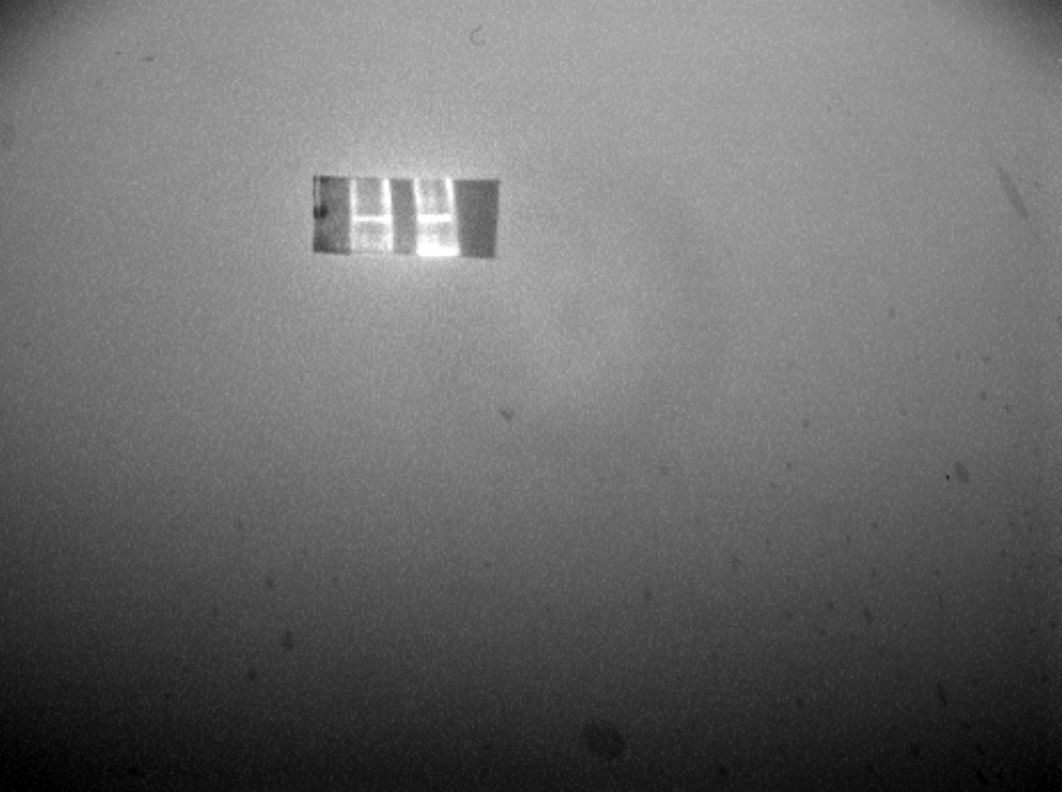

Supplement: Supplementary file 3 [file Data_Sheet_3.ZIP › FIG-1C/HCT116/membrane with marker-HCT116-HIFA.tif]

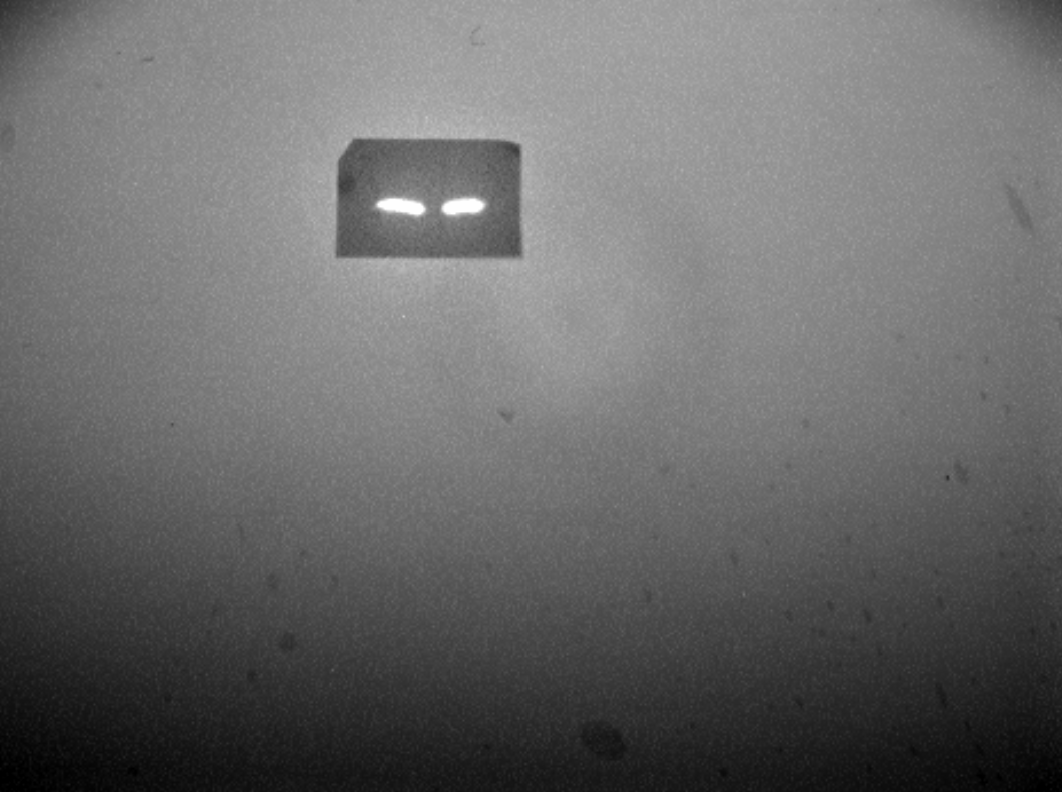

Supplement: Supplementary file 3 [file Data_Sheet_3.ZIP › FIG-1C/HCT116/membrane with marker-HCT116-LIN28A.tif]

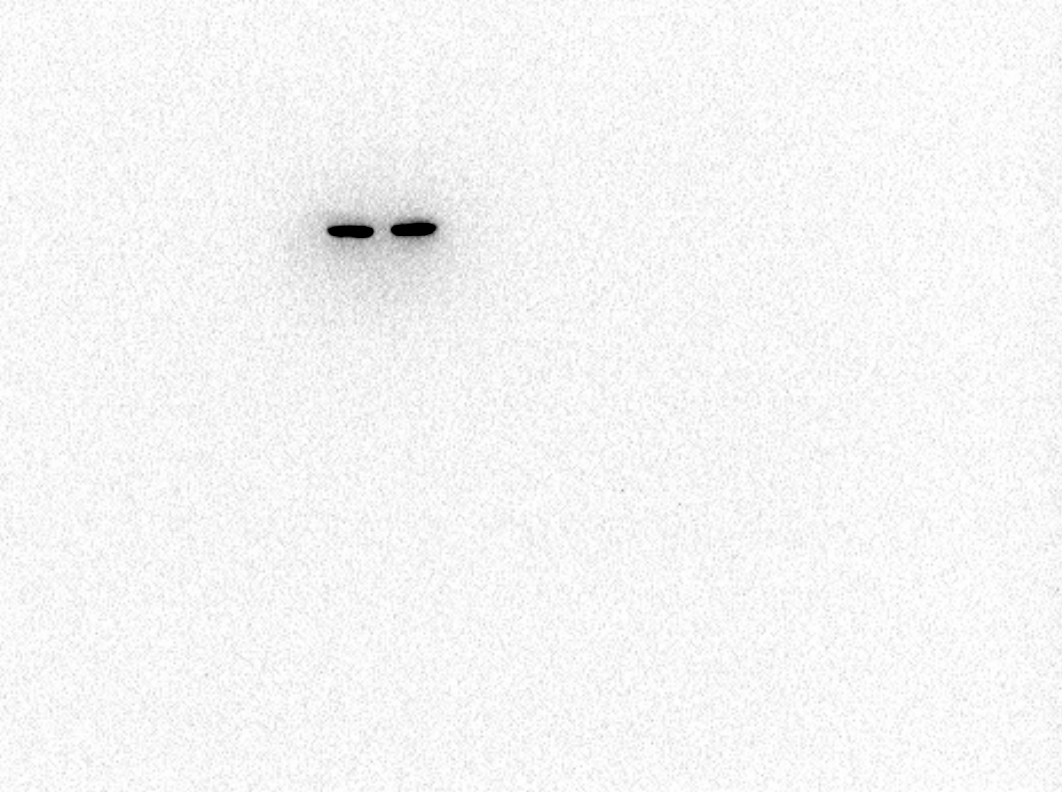

Supplement: Supplementary file 3 [file Data_Sheet_3.ZIP › FIG-1C/HCT116/membrane-HCT116-ACTIN.tif]

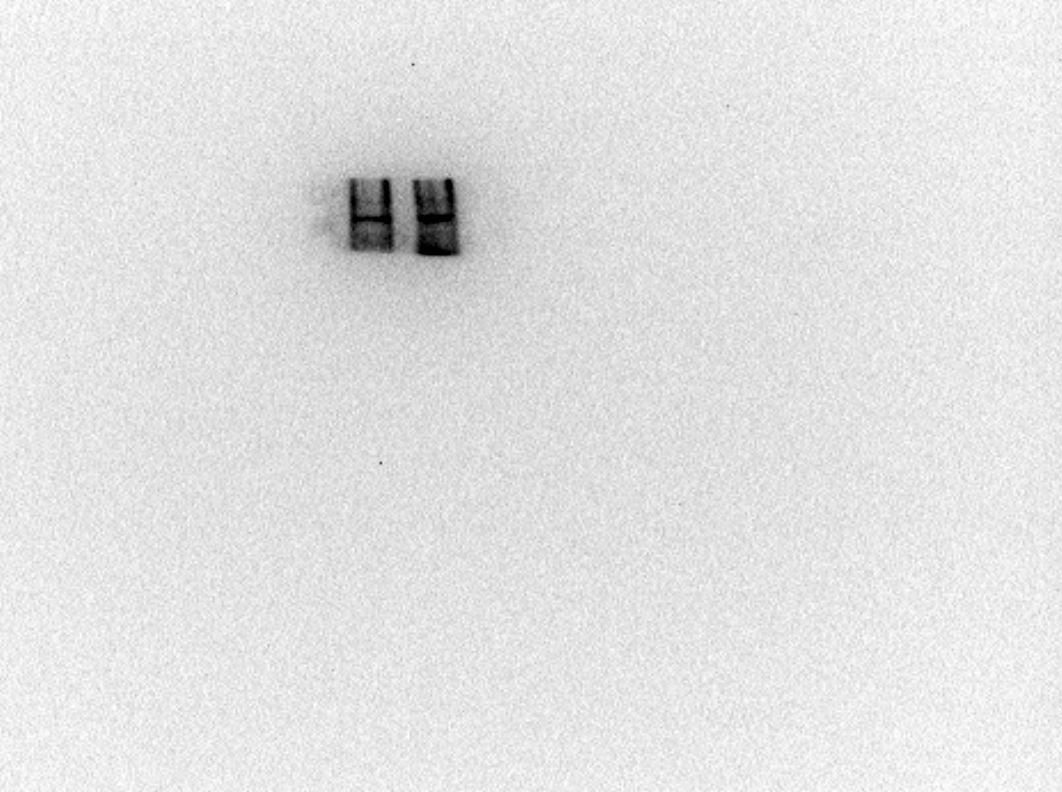

Supplement: Supplementary file 3 [file Data_Sheet_3.ZIP › FIG-1C/HCT116/membrane-HCT116-HIFA.tif]

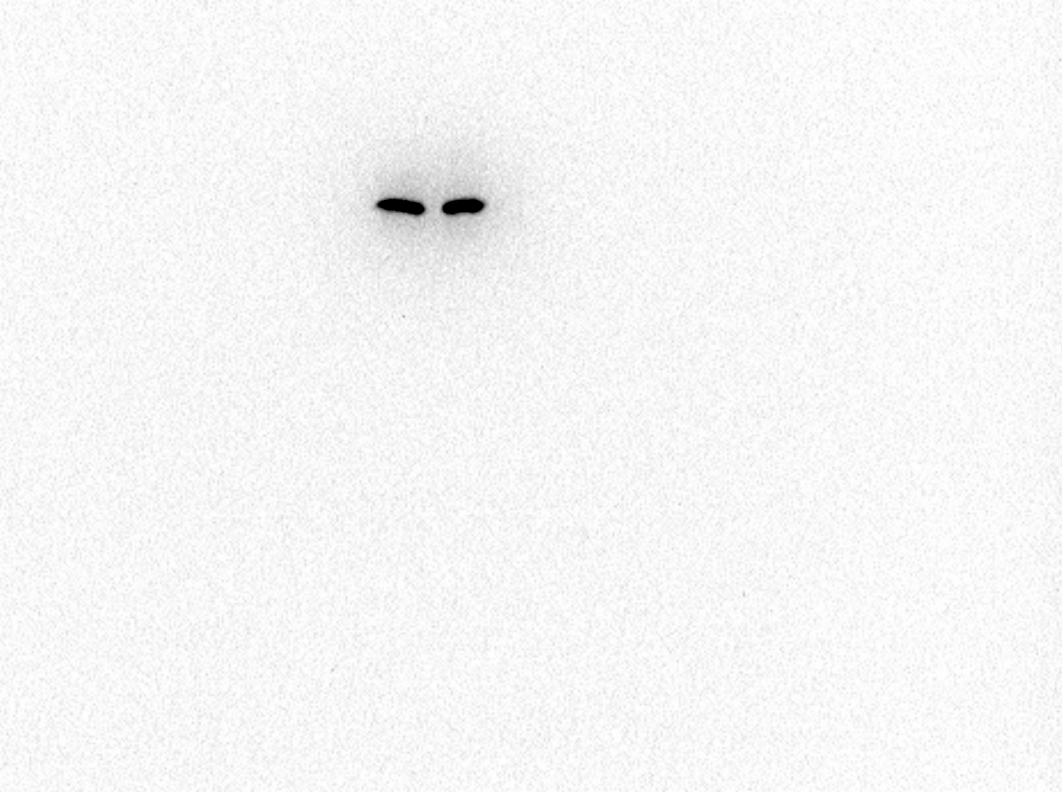

Supplement: Supplementary file 3 [file Data_Sheet_3.ZIP › FIG-1C/HCT116/membrane-HCT116-LIN28A.tif]

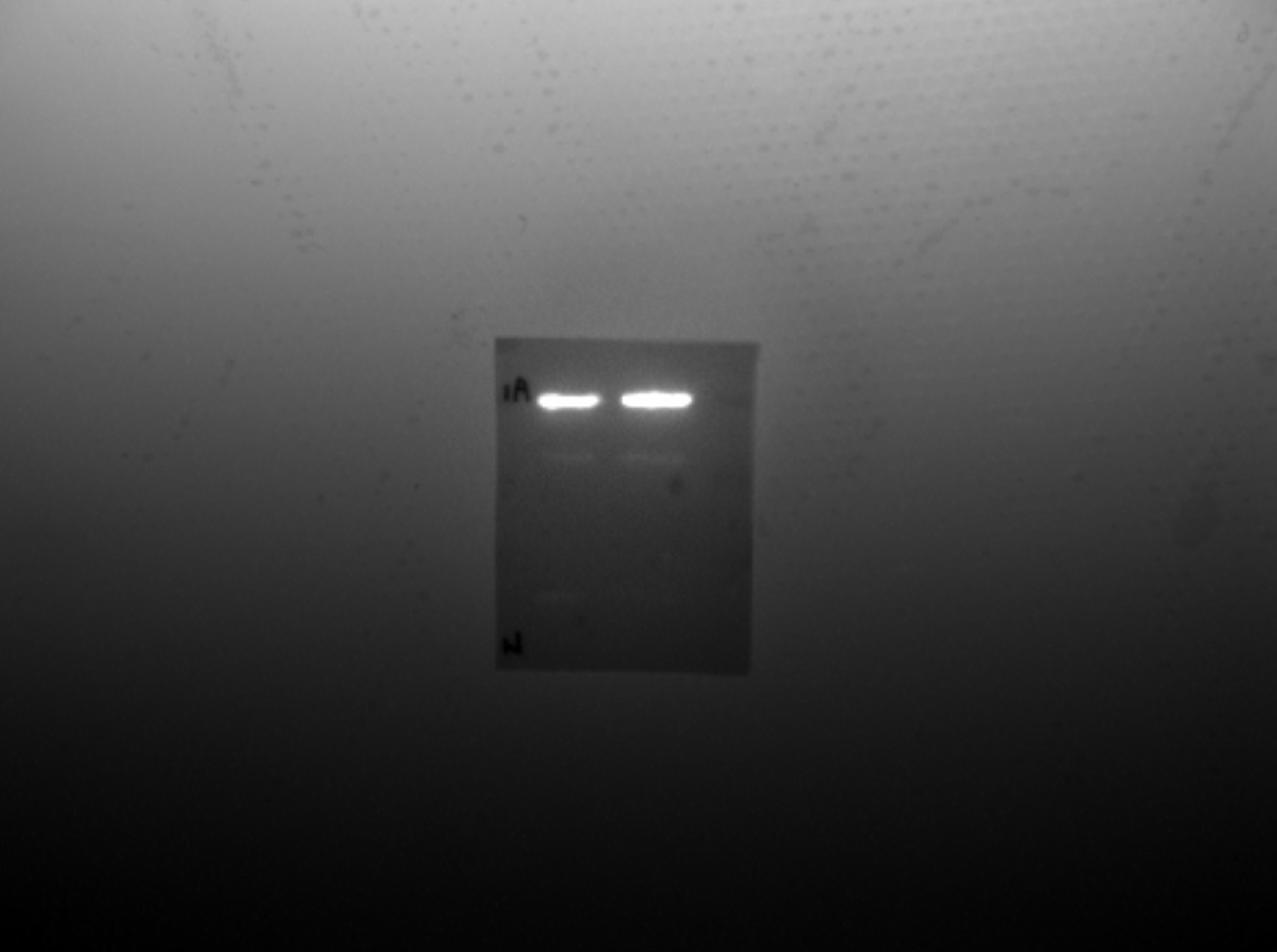

Supplement: Supplementary file 3 [file Data_Sheet_3.ZIP › FIG-1C/HCT15/membrane with marker-ACTIN.tif]

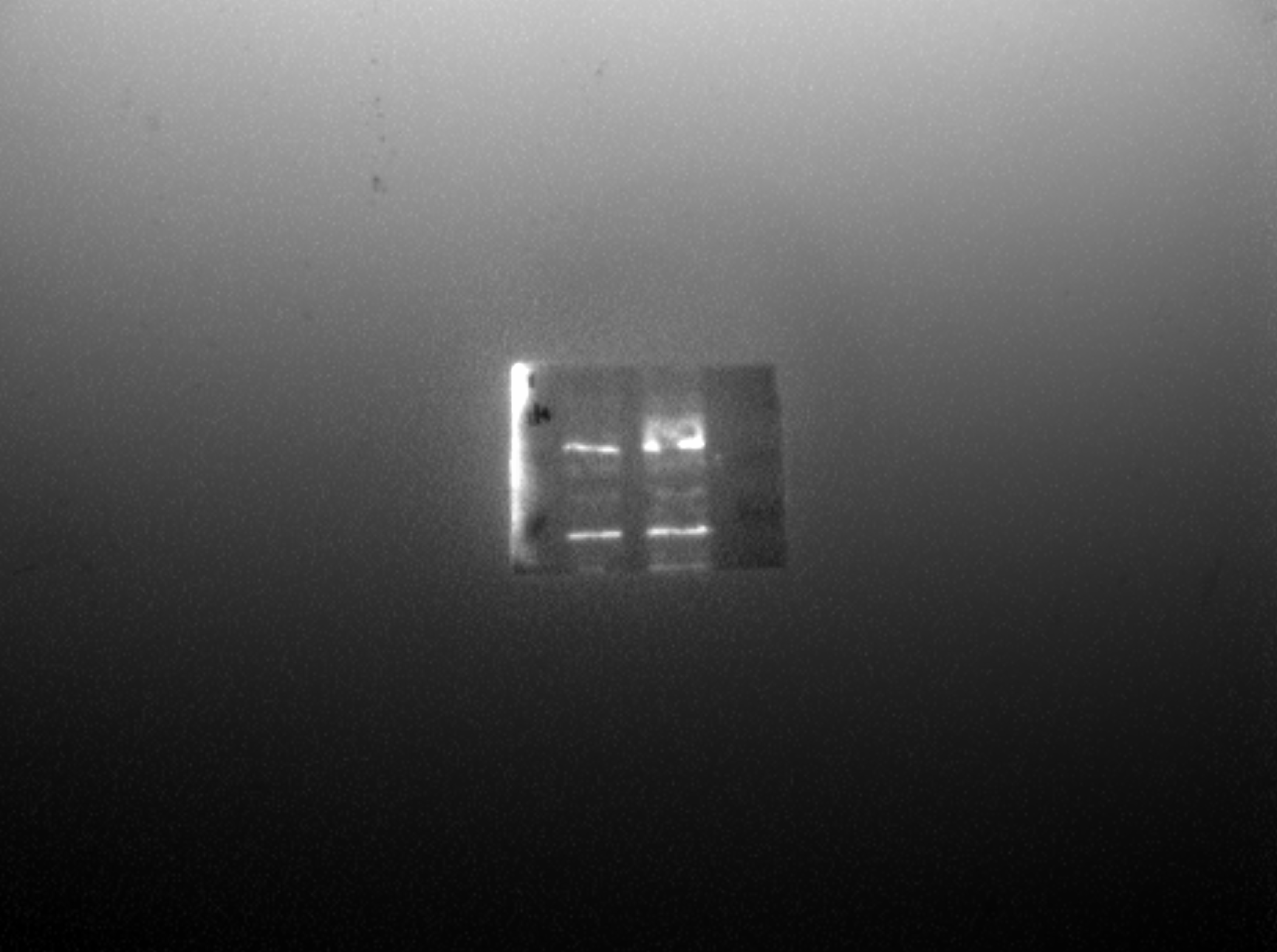

Supplement: Supplementary file 3 [file Data_Sheet_3.ZIP › FIG-1C/HCT15/membrane with marker-HIFA.tif]

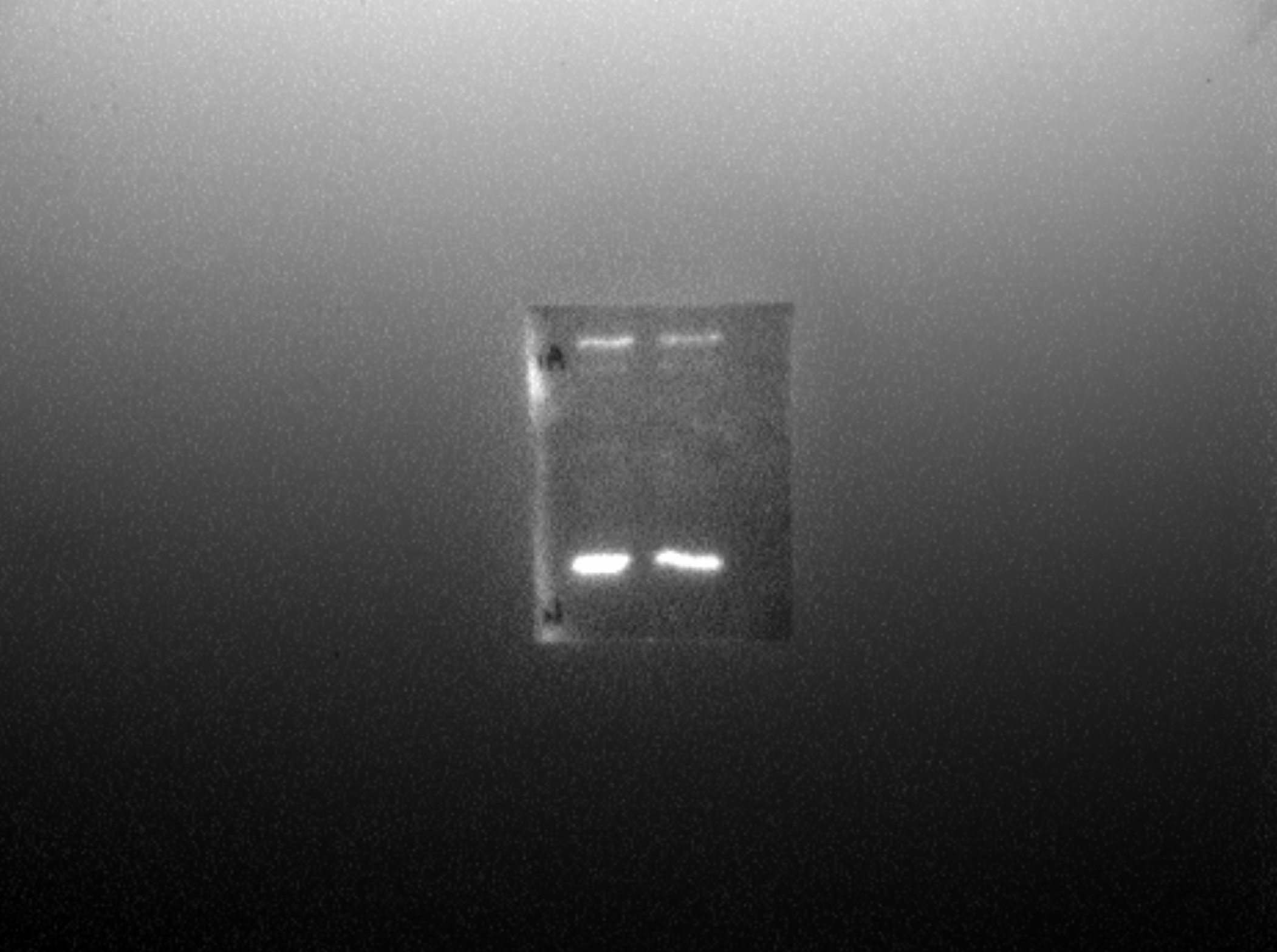

Supplement: Supplementary file 3 [file Data_Sheet_3.ZIP › FIG-1C/HCT15/membrane with marker-LIN28A.tif]

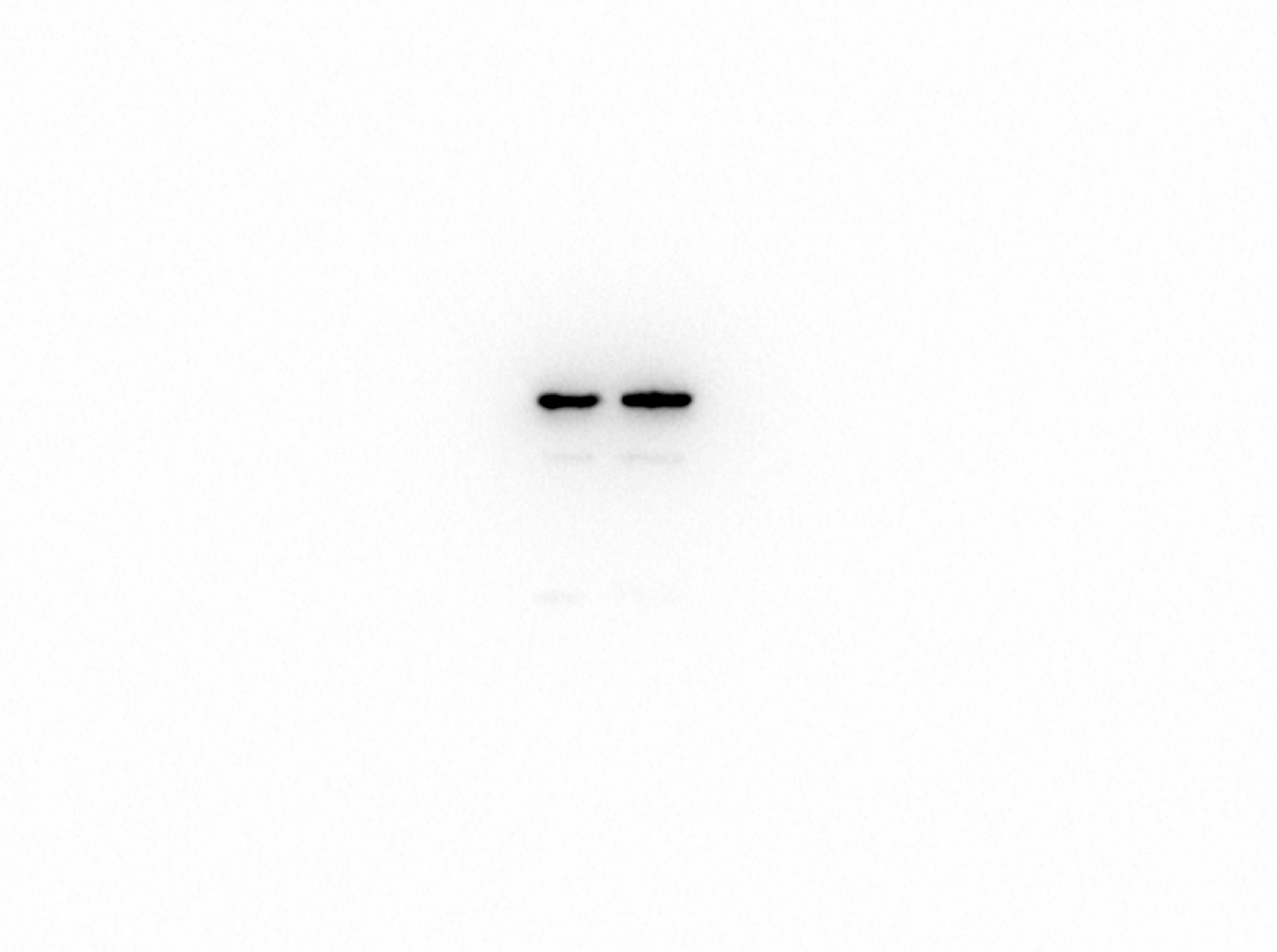

Supplement: Supplementary file 3 [file Data_Sheet_3.ZIP › FIG-1C/HCT15/membrane-ACTIN.tif]

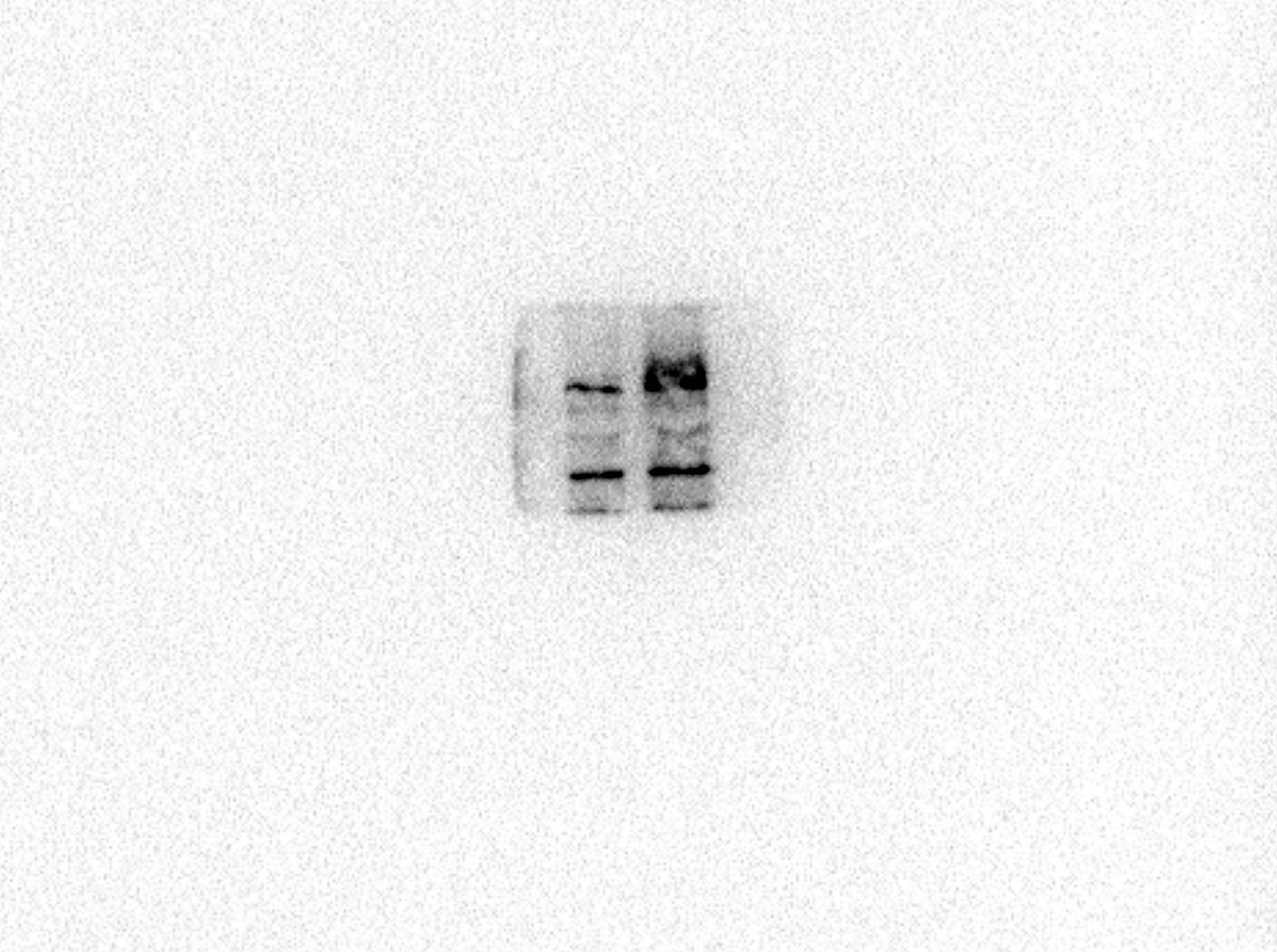

Supplement: Supplementary file 3 [file Data_Sheet_3.ZIP › FIG-1C/HCT15/membrane-HIFA.tif]

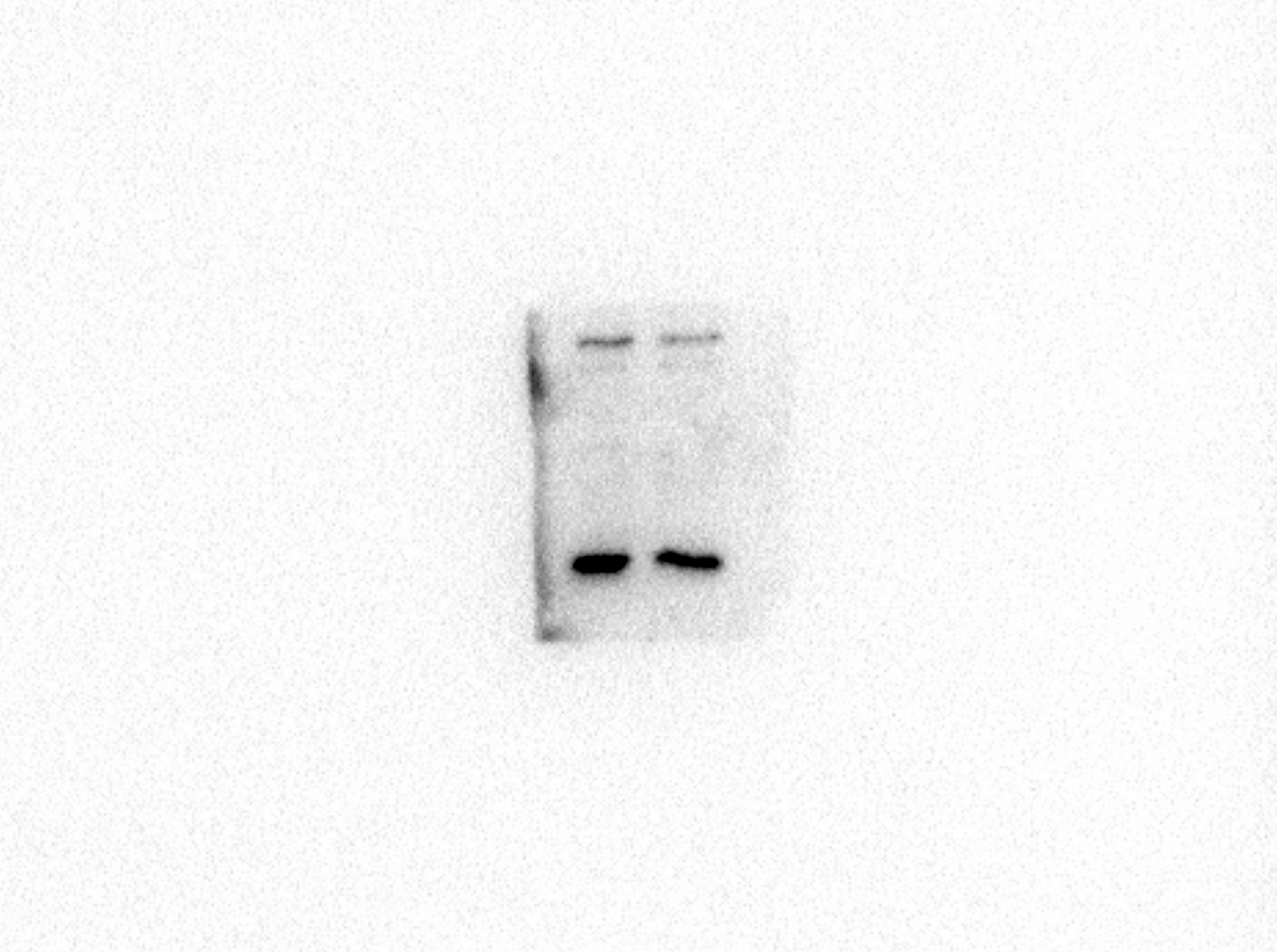

Supplement: Supplementary file 3 [file Data_Sheet_3.ZIP › FIG-1C/HCT15/membrane-LIN28A.tif]

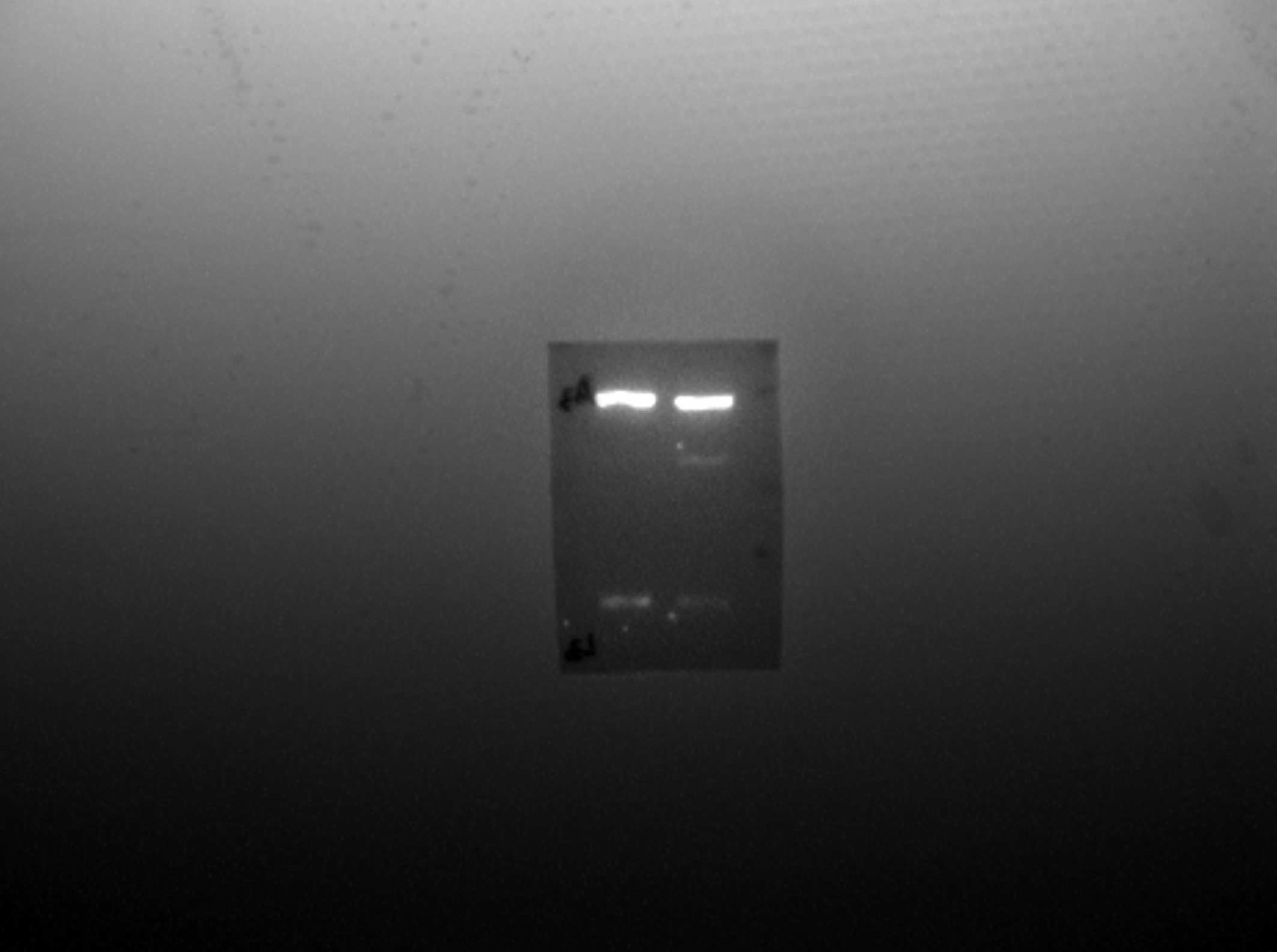

Supplement: Supplementary file 3 [file Data_Sheet_3.ZIP › FIG-1C/SW1116/membrane with marker-ACTIN.tif]

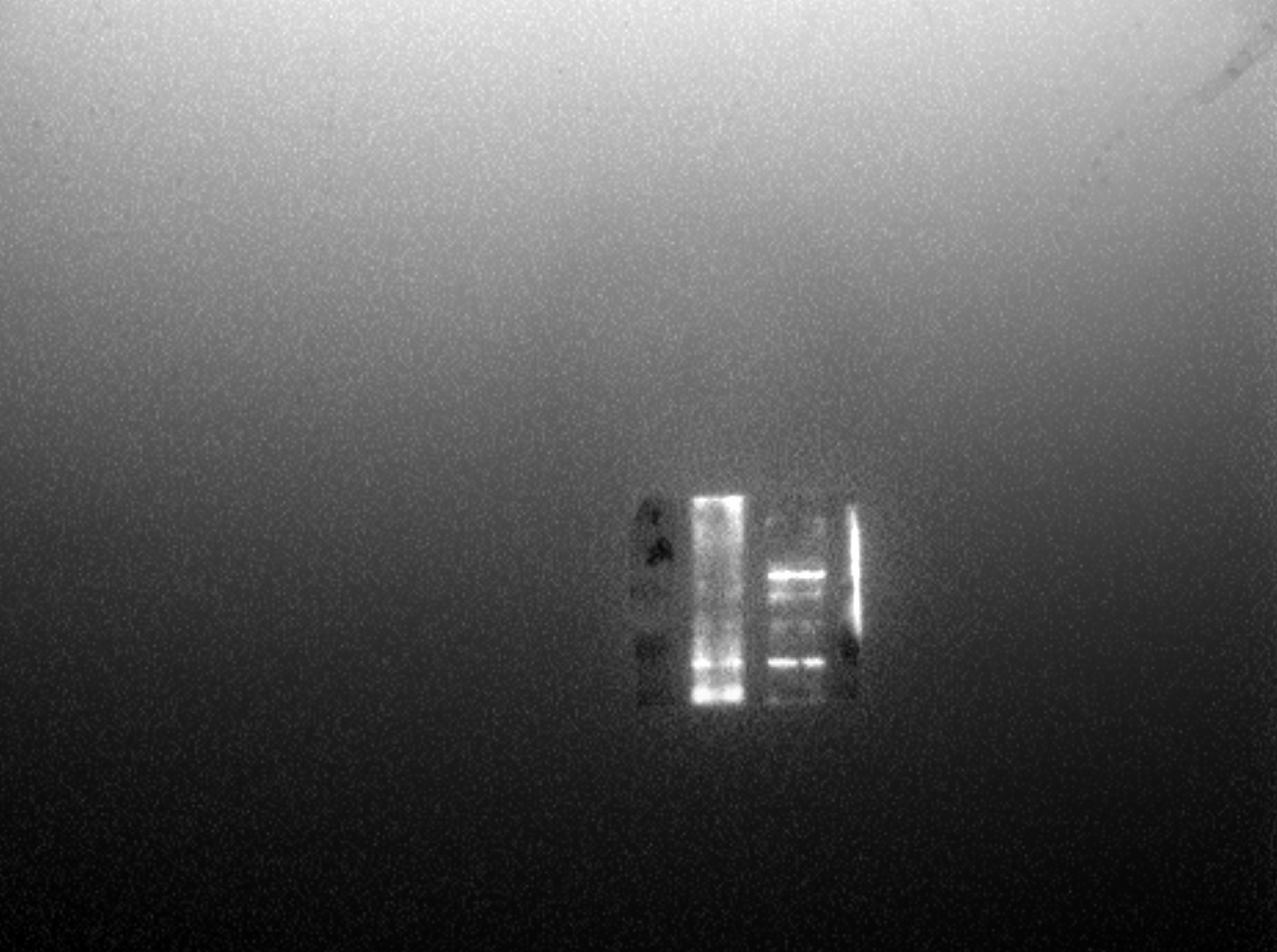

Supplement: Supplementary file 3 [file Data_Sheet_3.ZIP › FIG-1C/SW1116/membrane with marker-HIFA.tif]

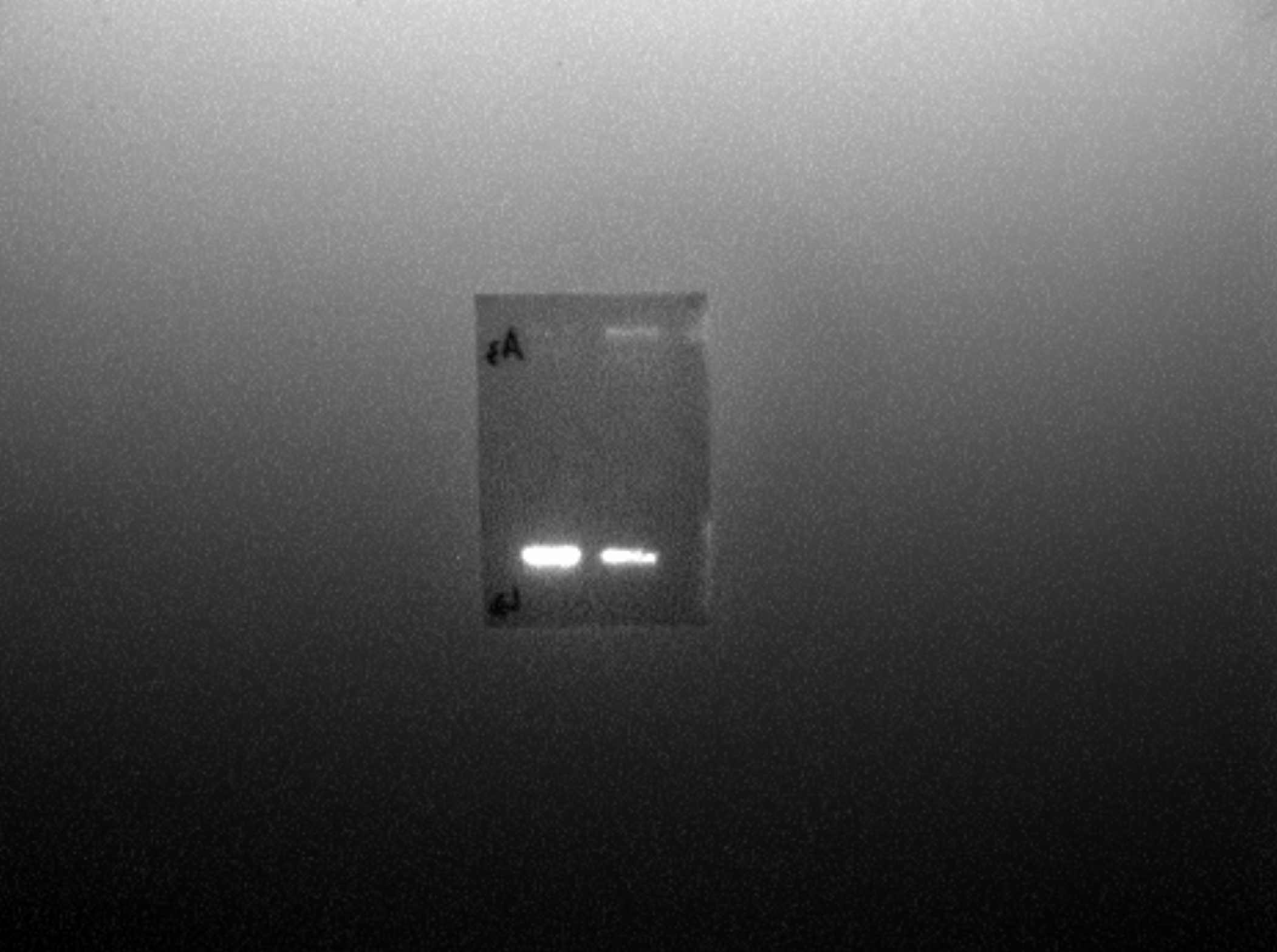

Supplement: Supplementary file 3 [file Data_Sheet_3.ZIP › FIG-1C/SW1116/membrane with marker-LIN28A.tif]

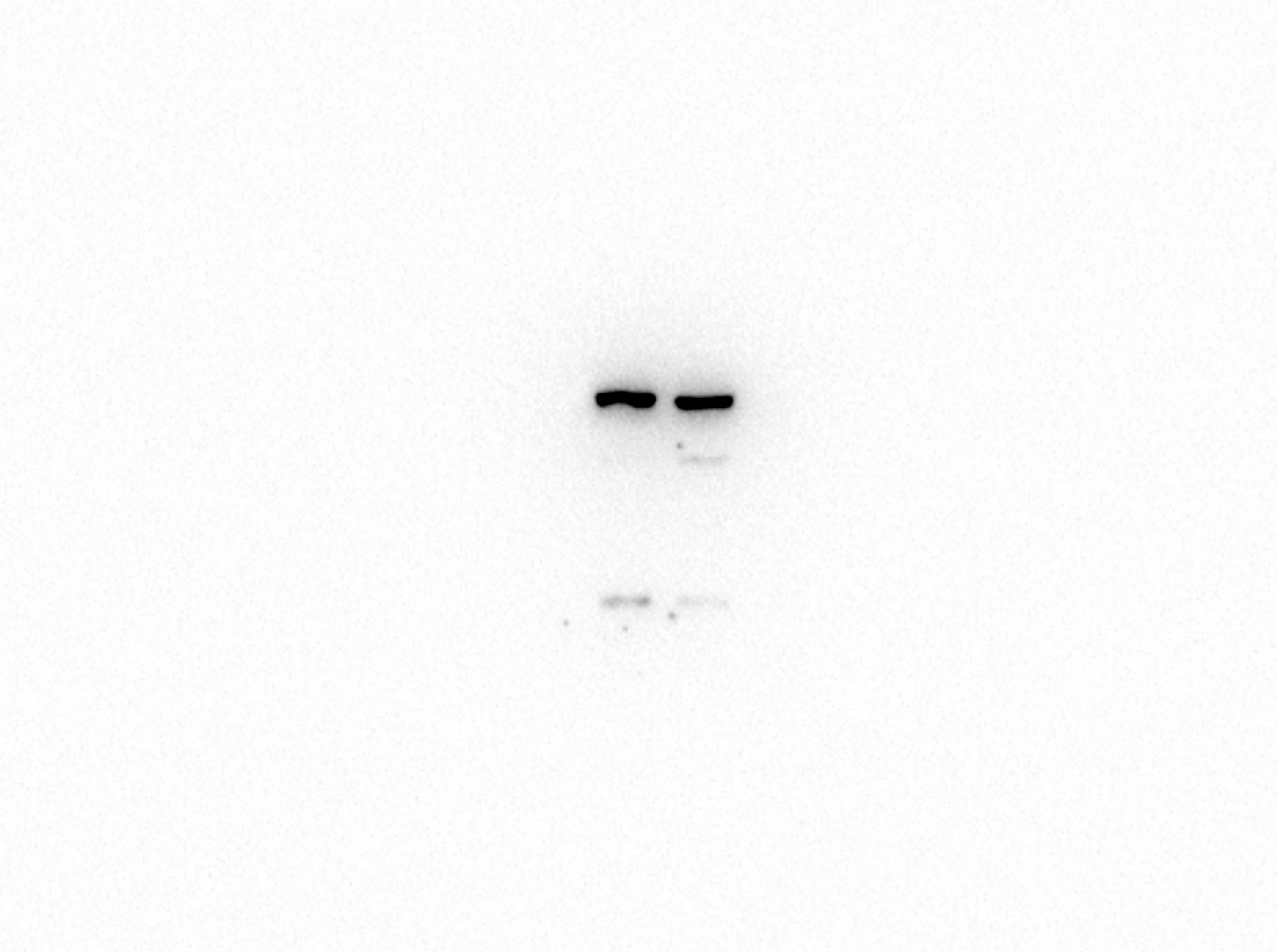

Supplement: Supplementary file 3 [file Data_Sheet_3.ZIP › FIG-1C/SW1116/membrane-ACTIN.tif]

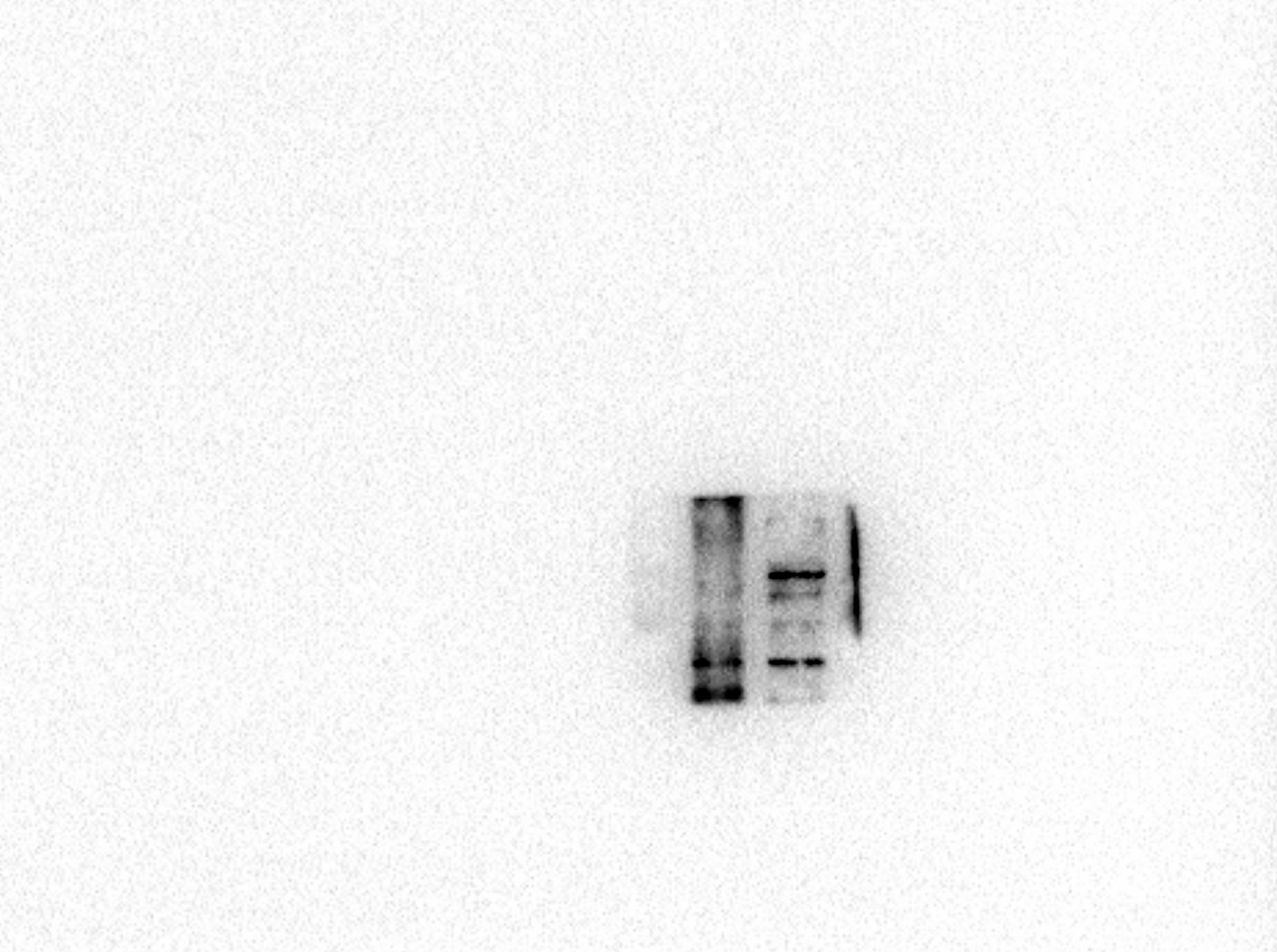

Supplement: Supplementary file 3 [file Data_Sheet_3.ZIP › FIG-1C/SW1116/membrane-HIFA.tif]

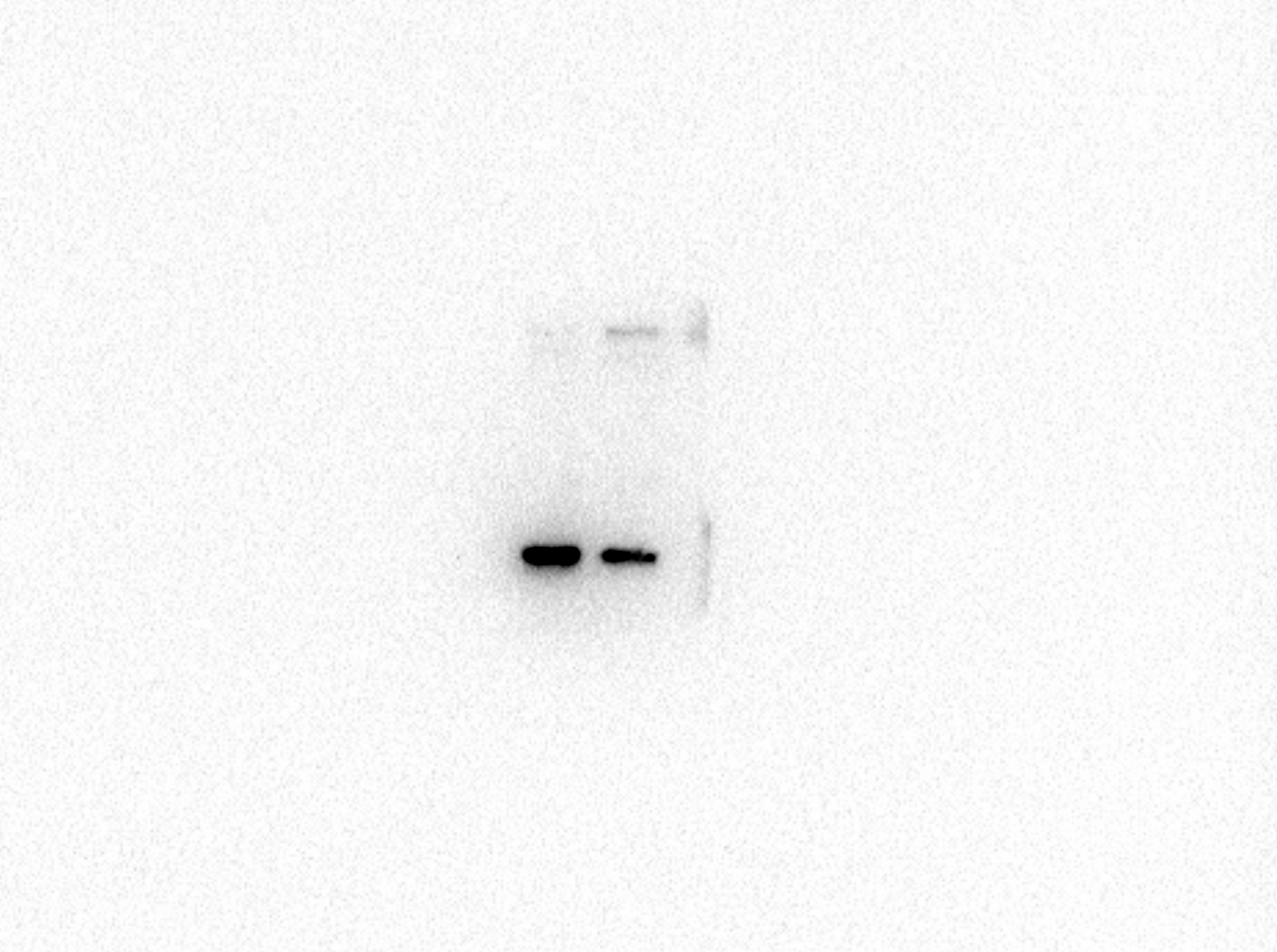

Supplement: Supplementary file 3 [file Data_Sheet_3.ZIP › FIG-1C/SW1116/membrane-LIN28A.tif]

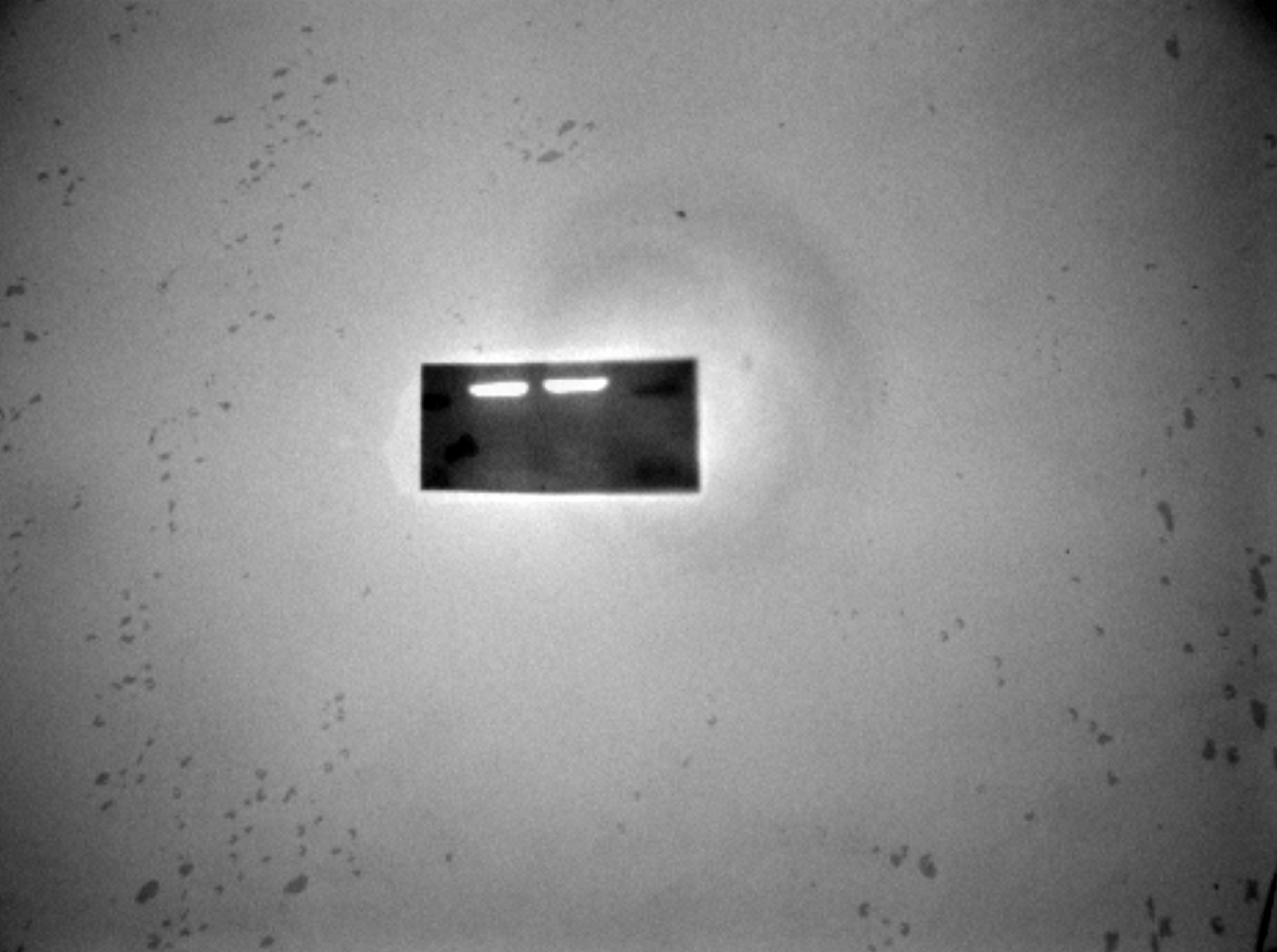

Supplement: Supplementary file 4 [file Data_Sheet_4.ZIP › FIG-1D/HCT116/membrane with marker-ACTIN.tif]

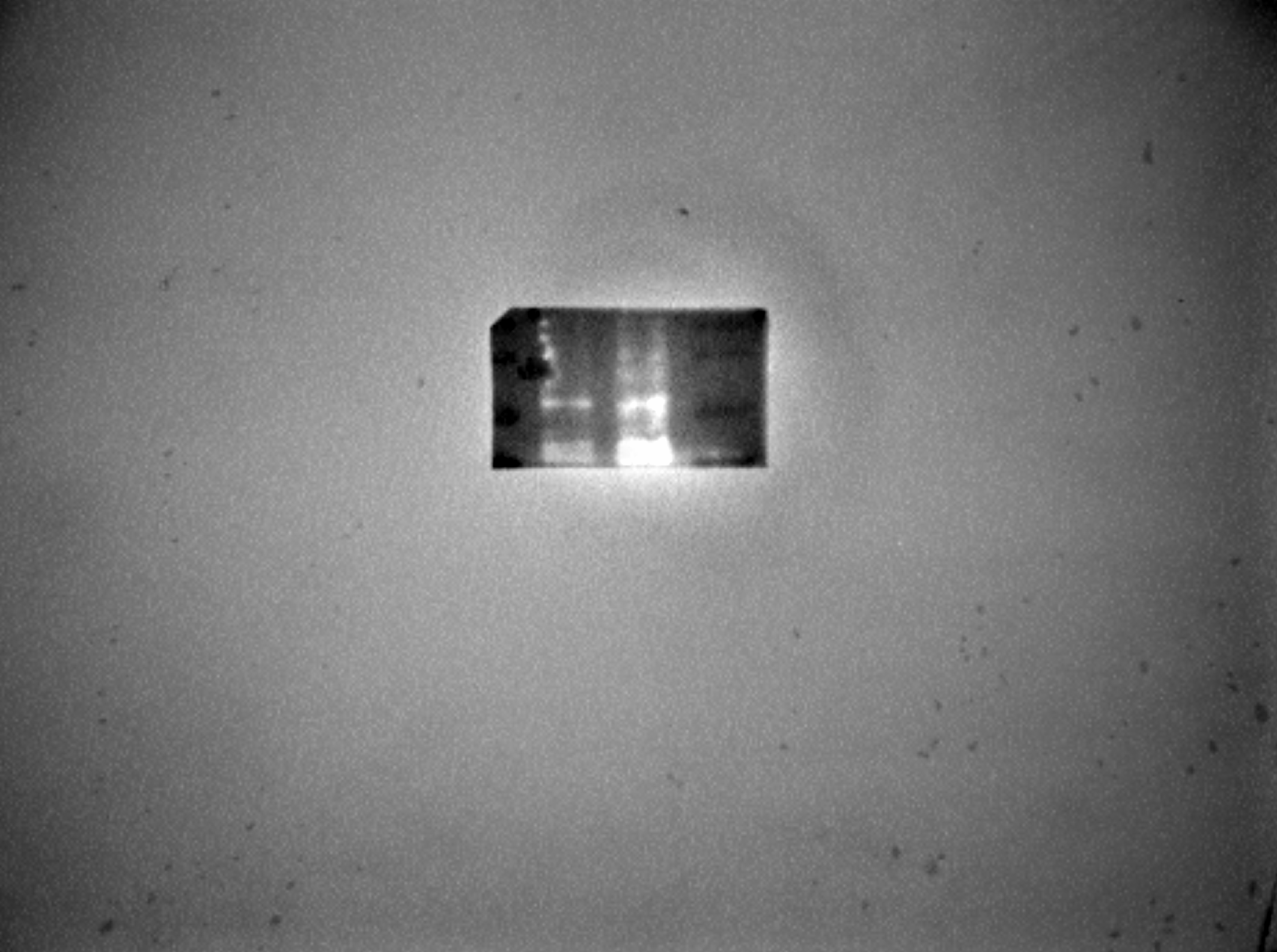

Supplement: Supplementary file 4 [file Data_Sheet_4.ZIP › FIG-1D/HCT116/membrane with marker-HIFA.tif]

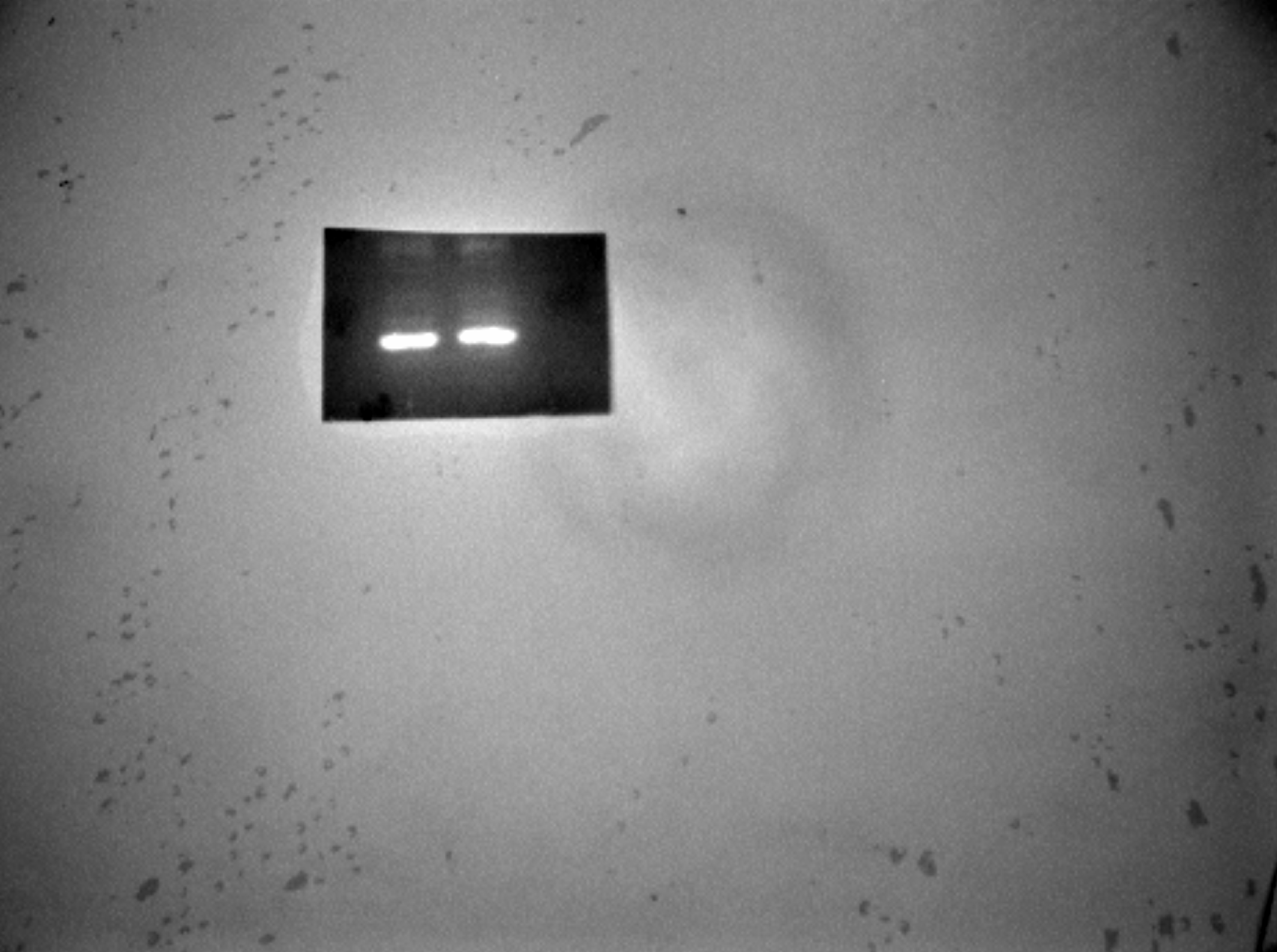

Supplement: Supplementary file 4 [file Data_Sheet_4.ZIP › FIG-1D/HCT116/membrane with marker-LIN28A.tif]

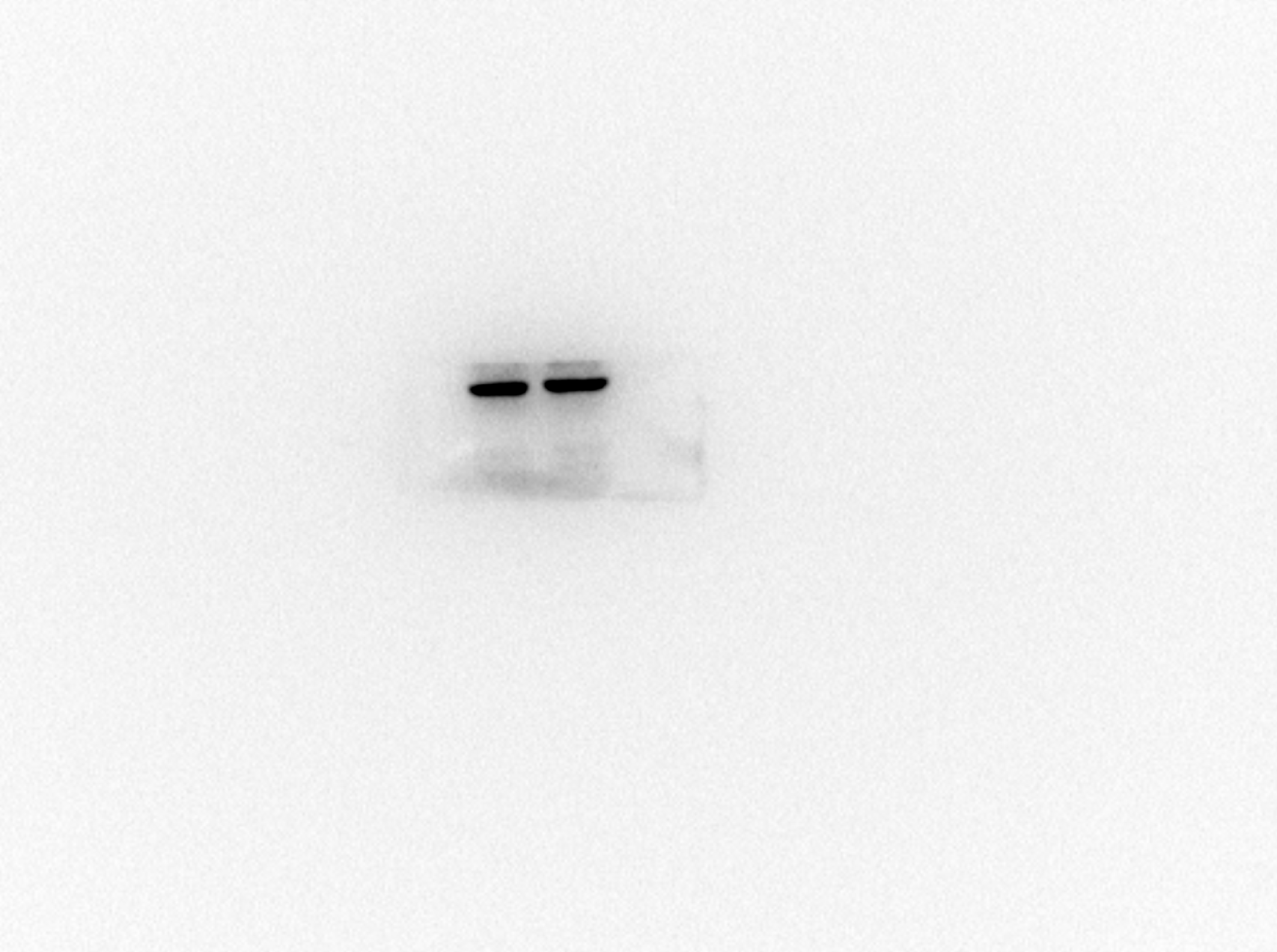

Supplement: Supplementary file 4 [file Data_Sheet_4.ZIP › FIG-1D/HCT116/membrane-ACTIN.tif]

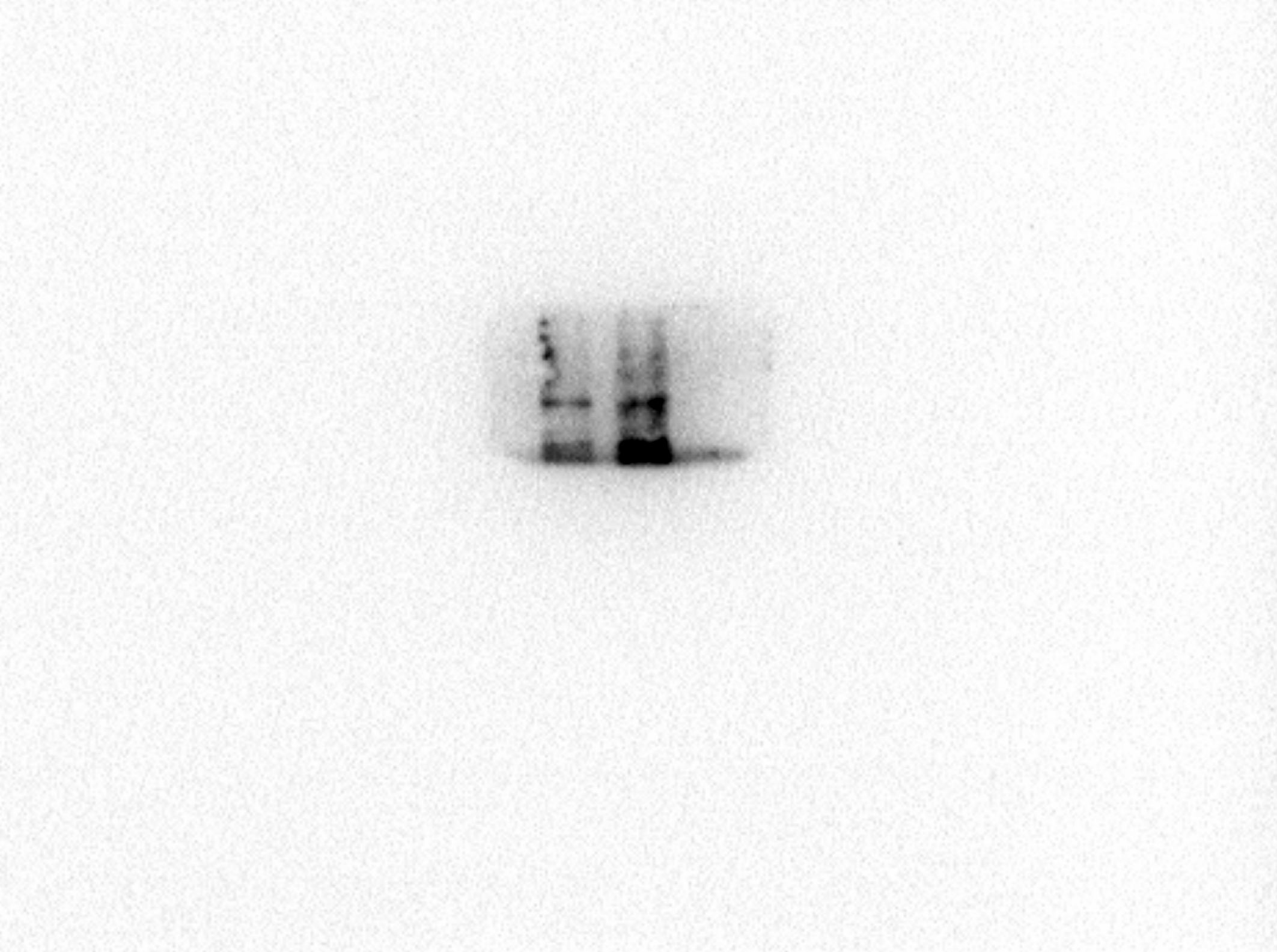

Supplement: Supplementary file 4 [file Data_Sheet_4.ZIP › FIG-1D/HCT116/membrane-HIFA.tif]

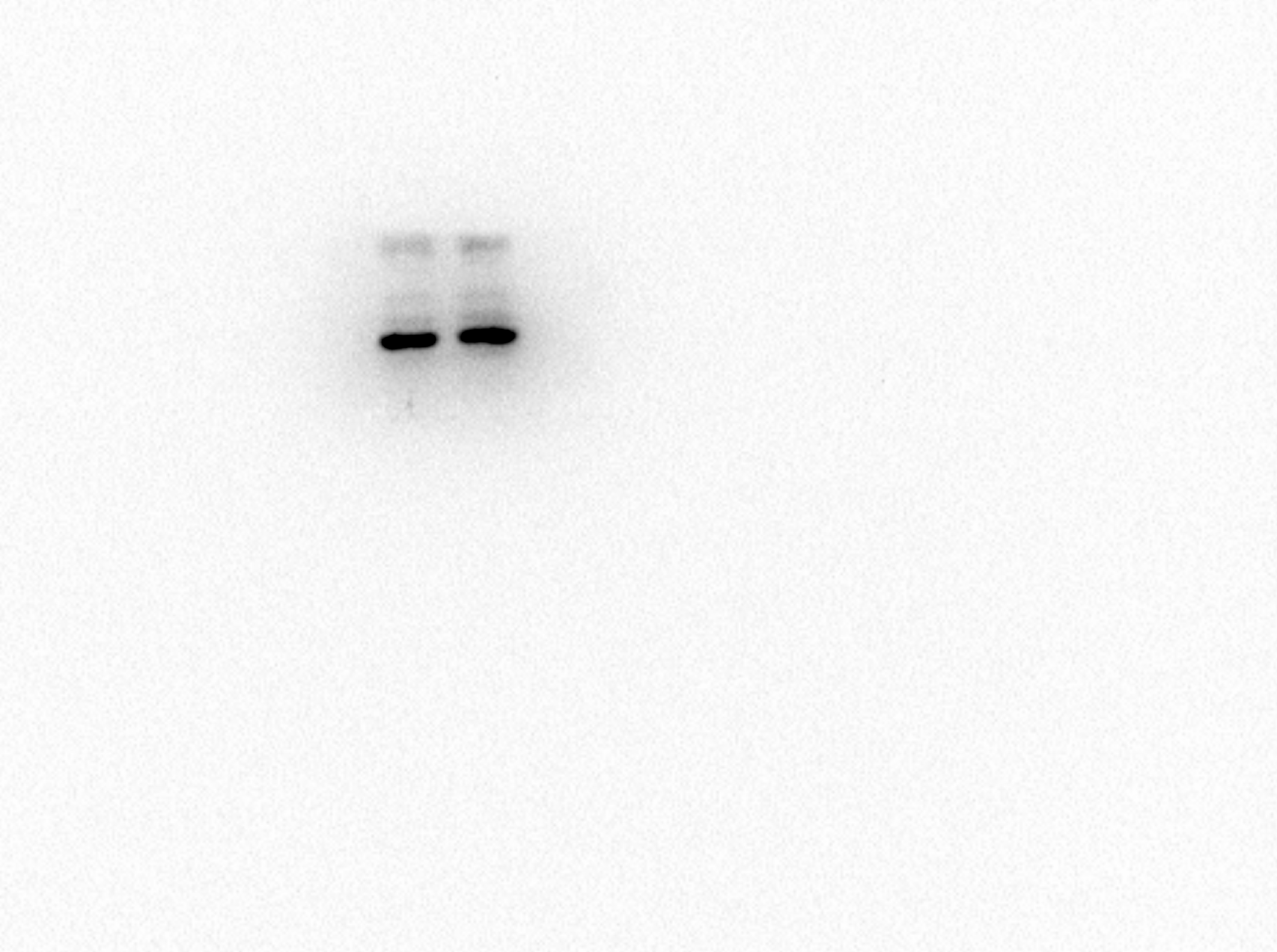

Supplement: Supplementary file 4 [file Data_Sheet_4.ZIP › FIG-1D/HCT116/membrane-LIN28A.tif]

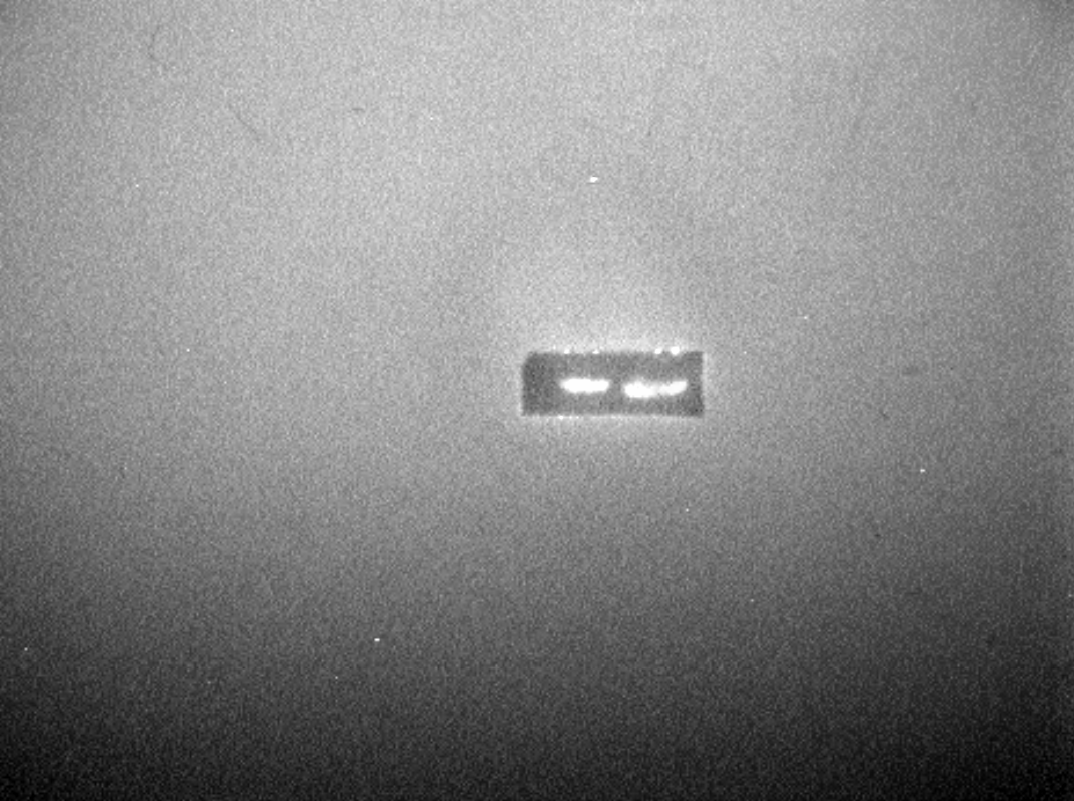

Supplement: Supplementary file 4 [file Data_Sheet_4.ZIP › FIG-1D/HCT15/membrane with marker-HCT15-actin.tif]

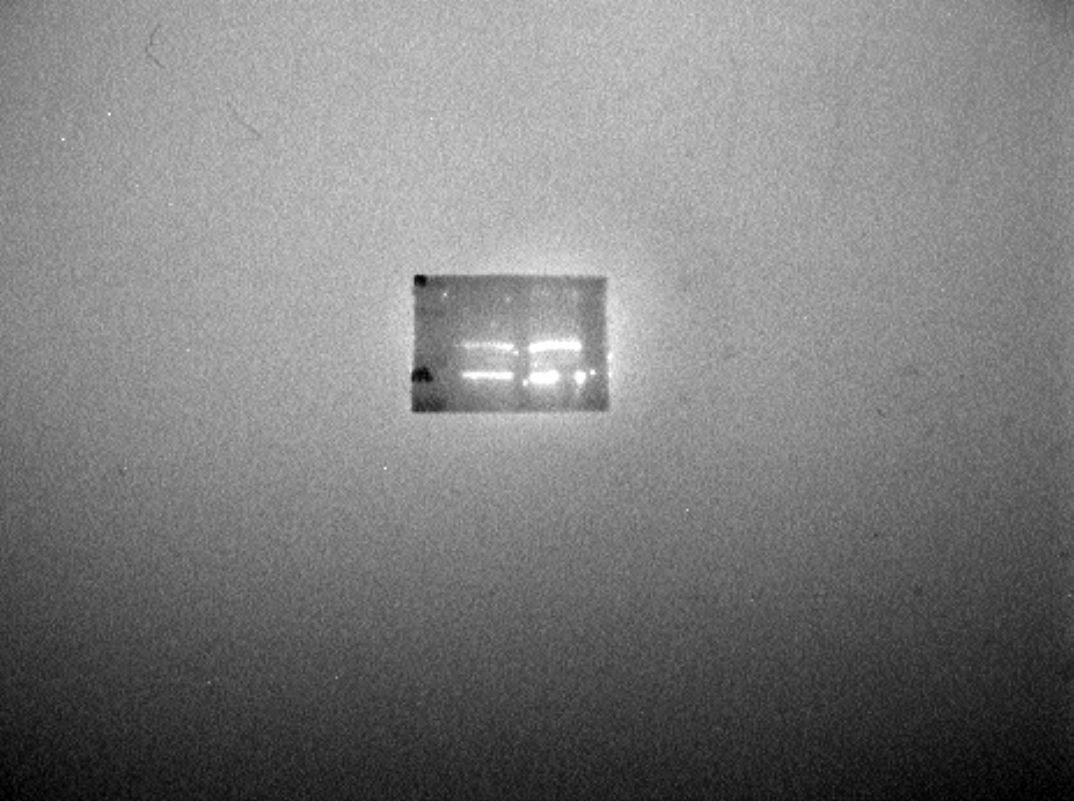

Supplement: Supplementary file 4 [file Data_Sheet_4.ZIP › FIG-1D/HCT15/membrane with marker-HCT15-hifa.tif]

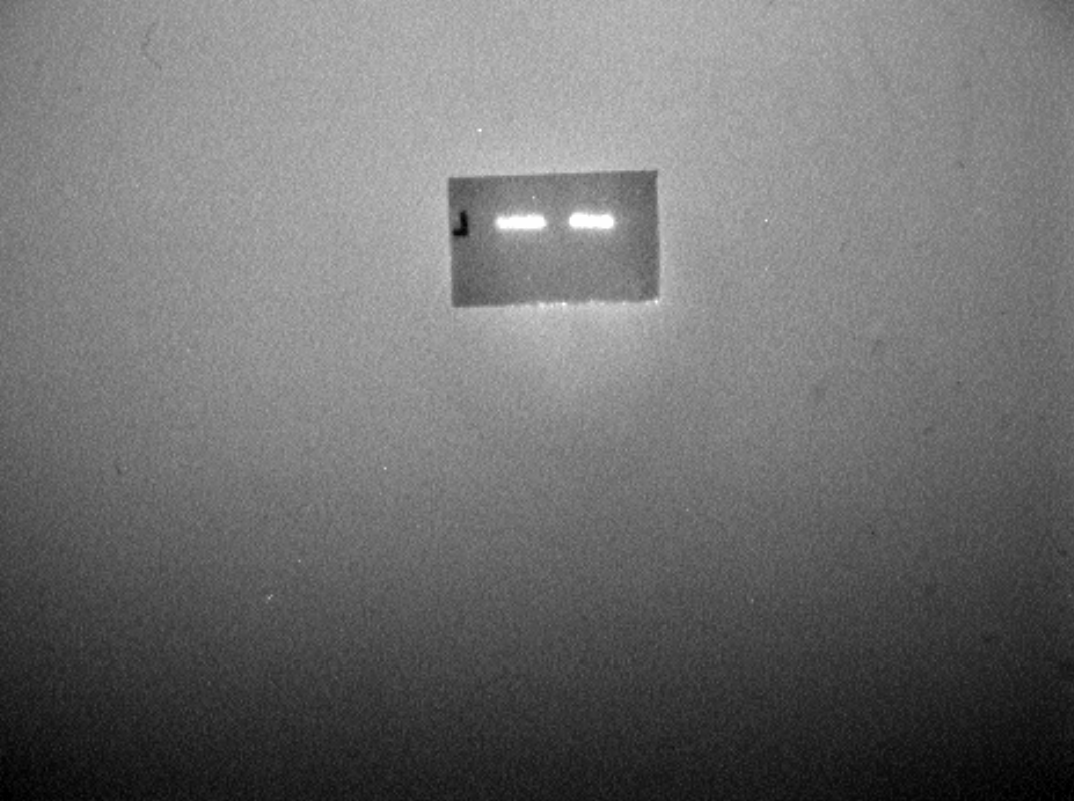

Supplement: Supplementary file 4 [file Data_Sheet_4.ZIP › FIG-1D/HCT15/membrane with marker-HCT15-LIN28A.tif]

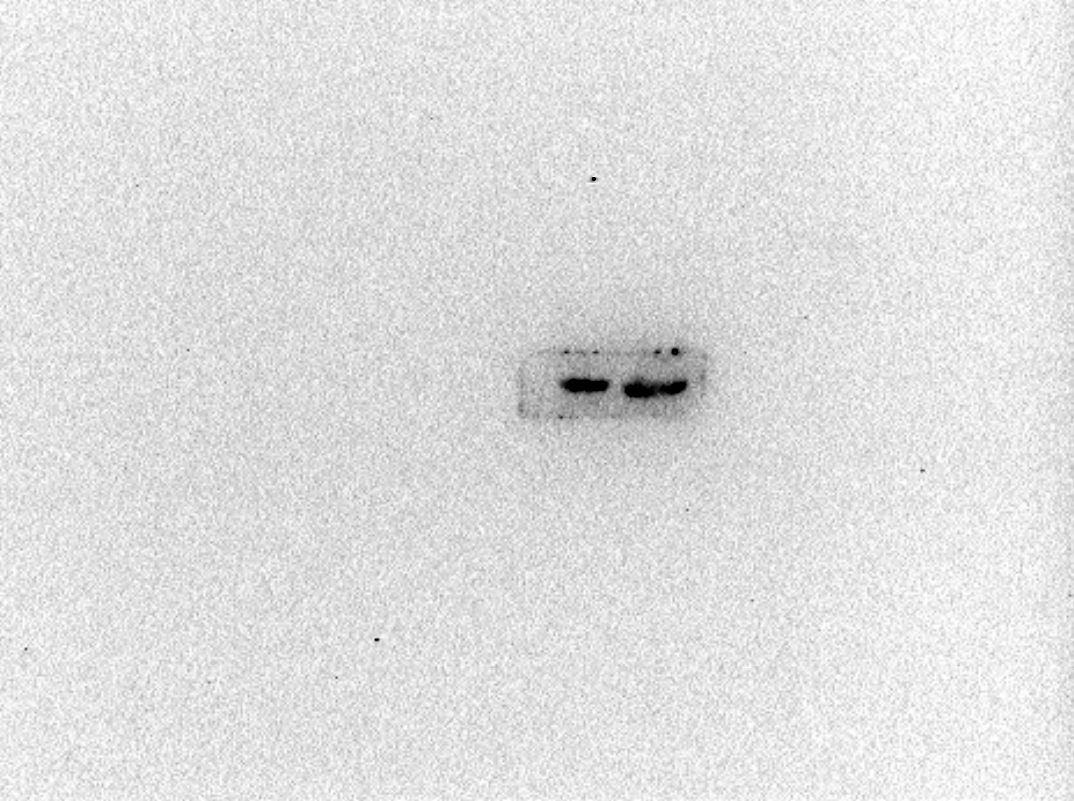

Supplement: Supplementary file 4 [file Data_Sheet_4.ZIP › FIG-1D/HCT15/membrane-HCT15-actin.tif]

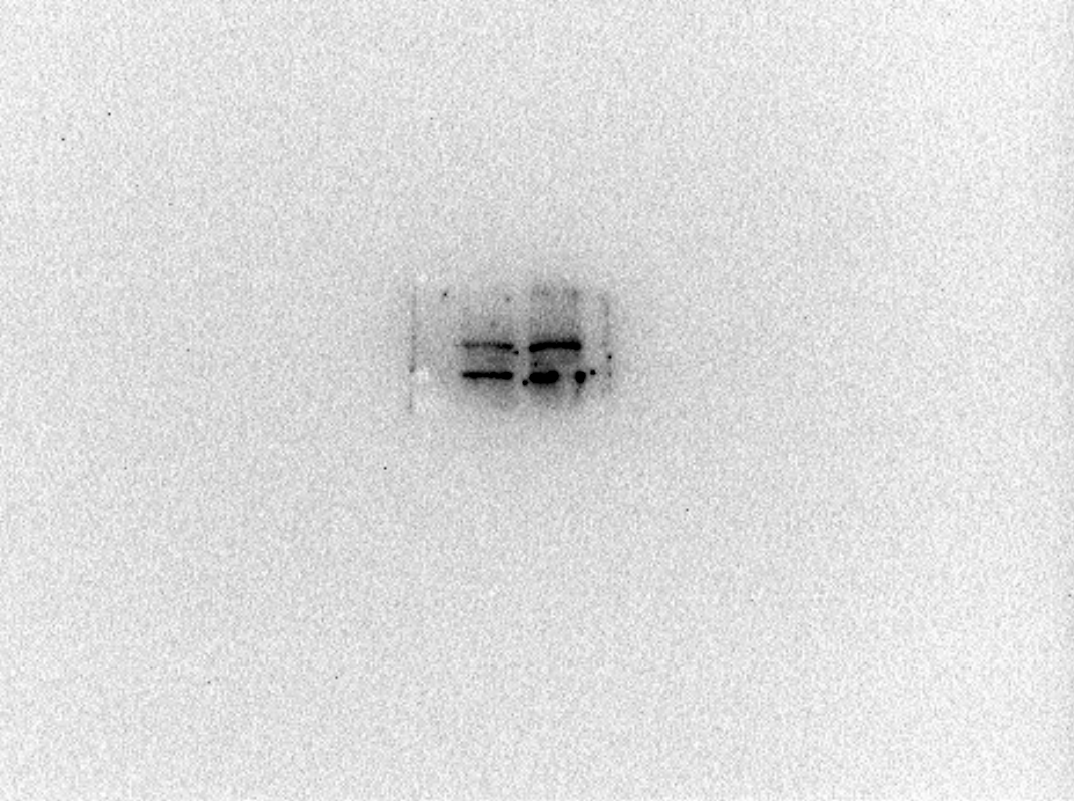

Supplement: Supplementary file 4 [file Data_Sheet_4.ZIP › FIG-1D/HCT15/membrane-HCT15-hifa.tif]

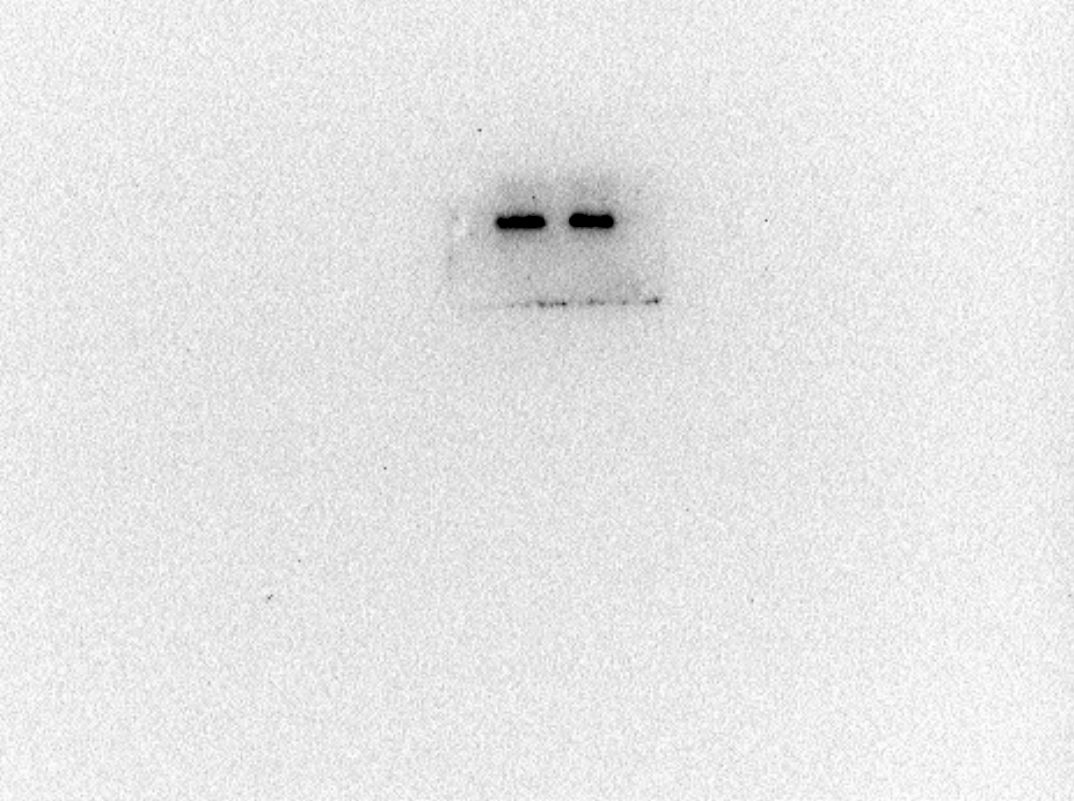

Supplement: Supplementary file 4 [file Data_Sheet_4.ZIP › FIG-1D/HCT15/membrane-HCT15-LIN28A.tif]

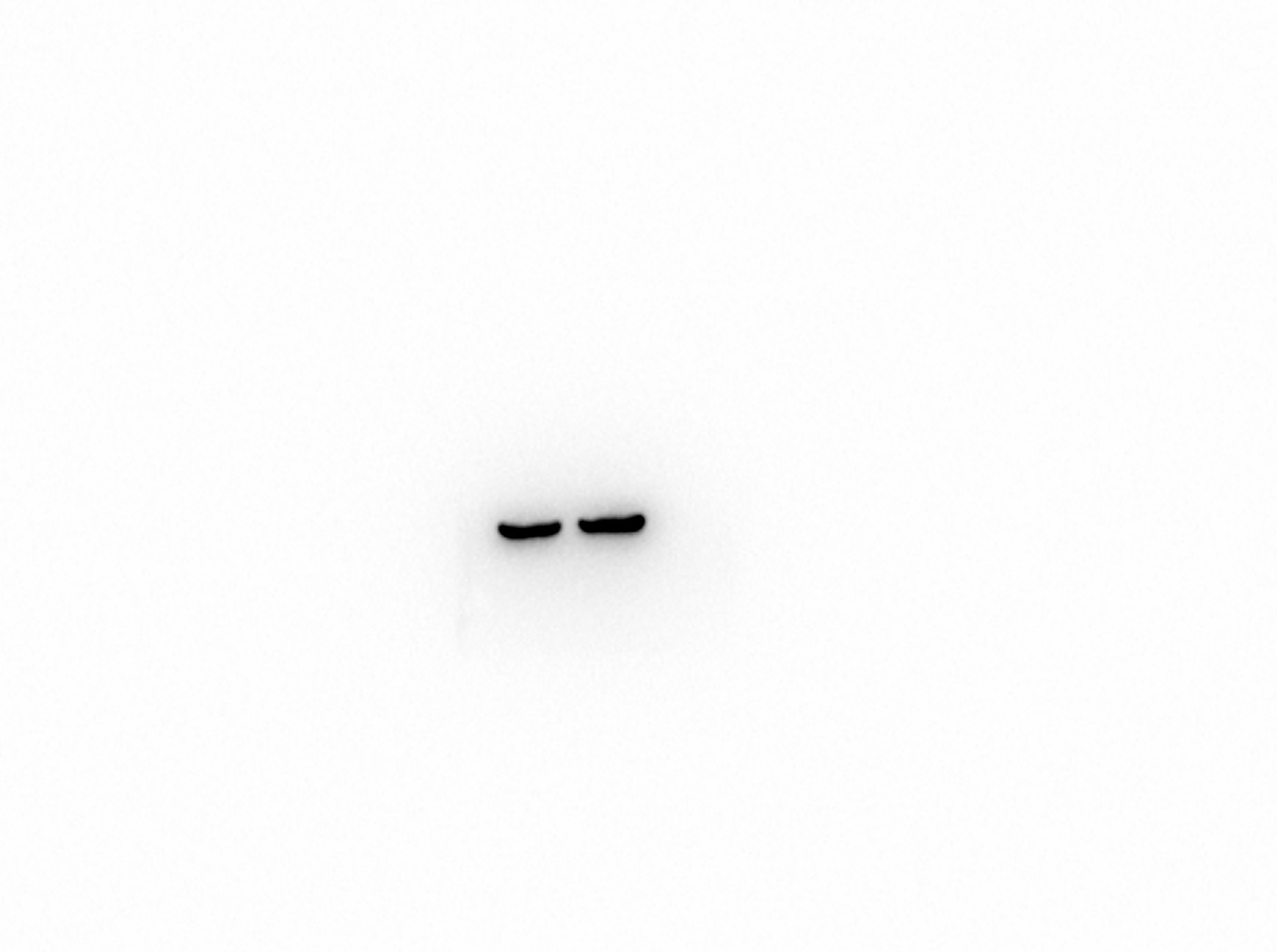

Supplement: Supplementary file 4 [file Data_Sheet_4.ZIP › FIG-1D/SW1116/membrane -ACTIN.tif]

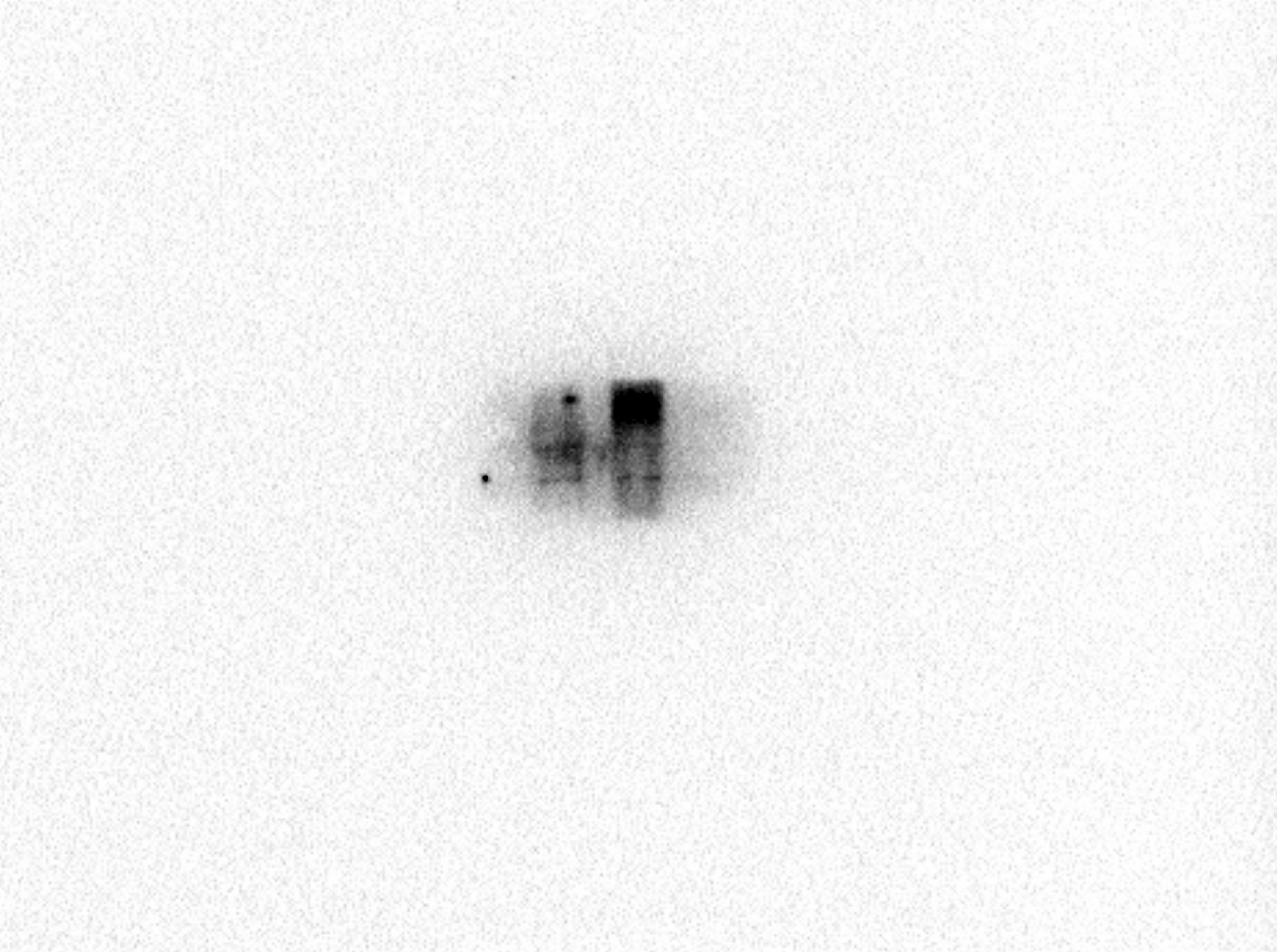

Supplement: Supplementary file 4 [file Data_Sheet_4.ZIP › FIG-1D/SW1116/membrane -HIFA.tif]

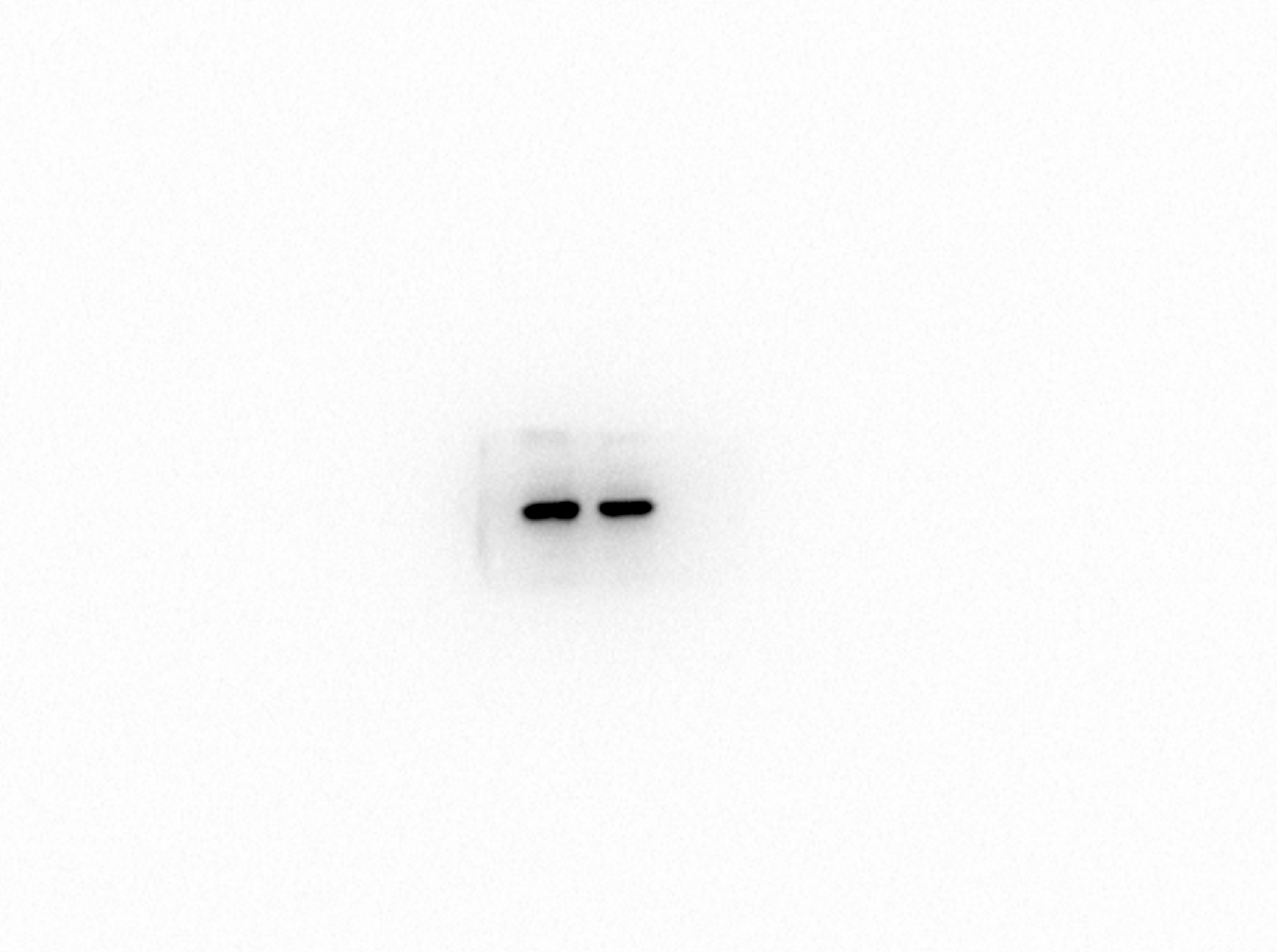

Supplement: Supplementary file 4 [file Data_Sheet_4.ZIP › FIG-1D/SW1116/membrane -LIN28A.tif]

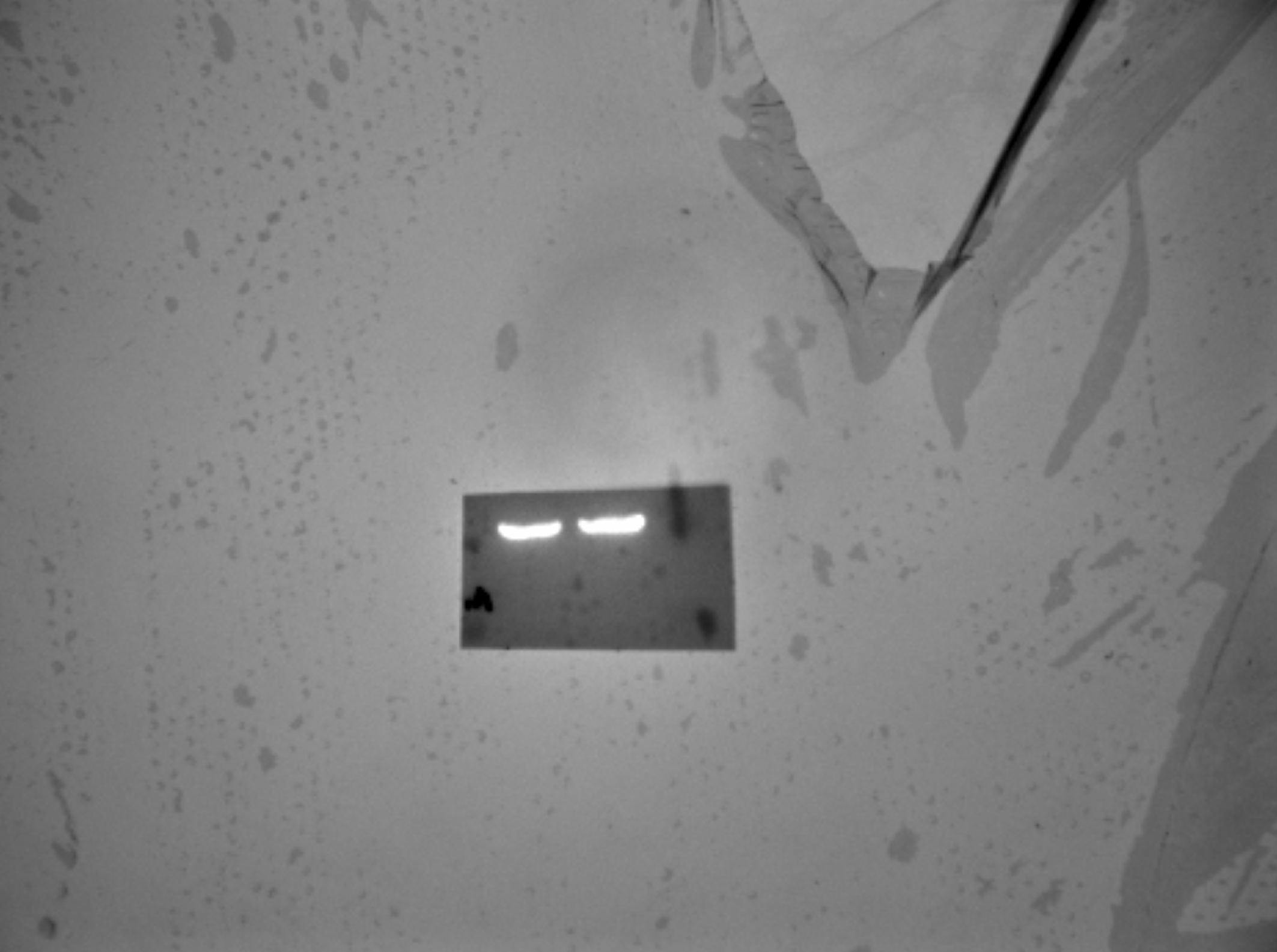

Supplement: Supplementary file 4 [file Data_Sheet_4.ZIP › FIG-1D/SW1116/membrane with marker-ACTIN.tif]

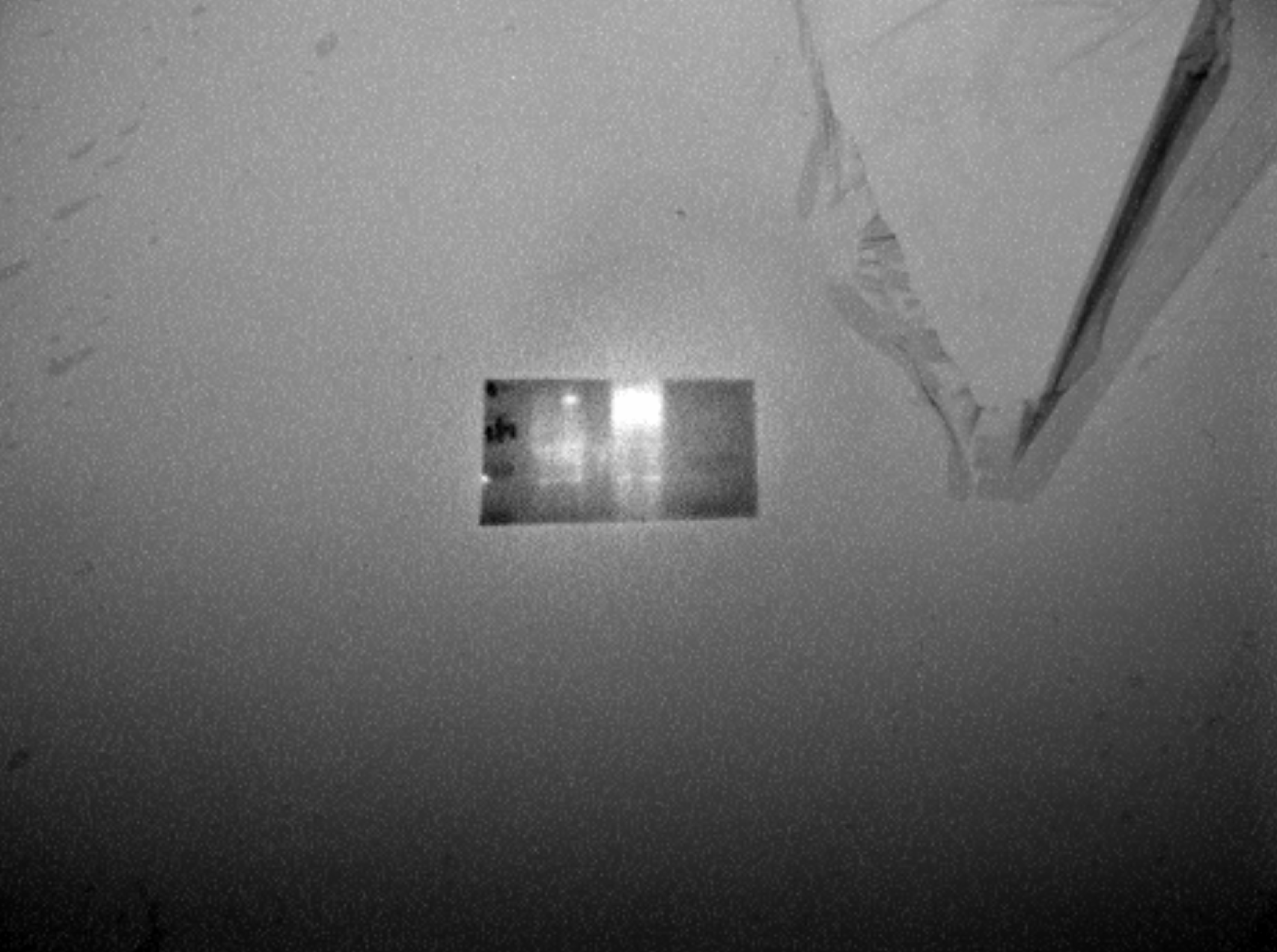

Supplement: Supplementary file 4 [file Data_Sheet_4.ZIP › FIG-1D/SW1116/membrane with marker-HIFA.tif]

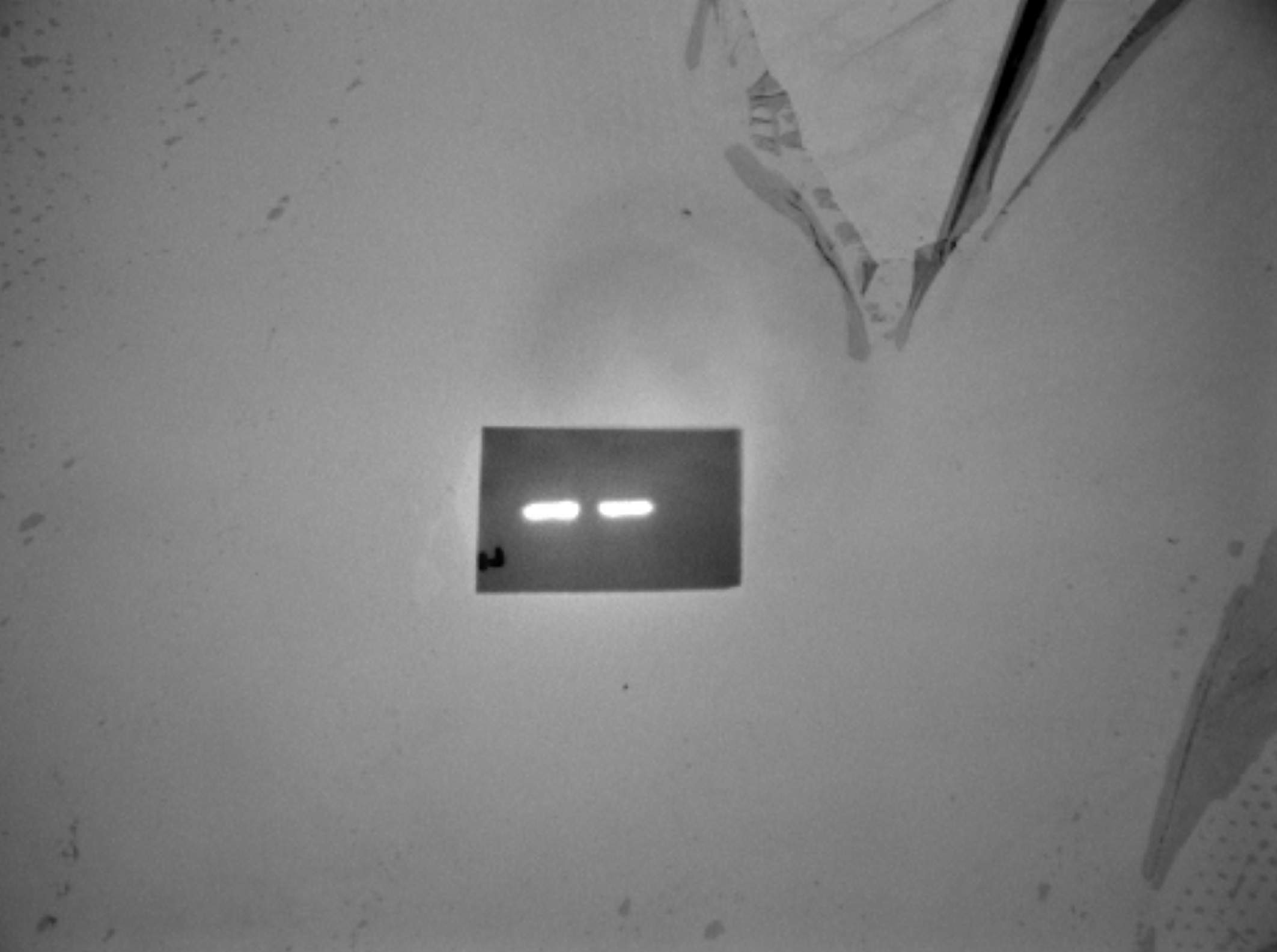

Supplement: Supplementary file 4 [file Data_Sheet_4.ZIP › FIG-1D/SW1116/membrane with marker-LIN28A.tif]

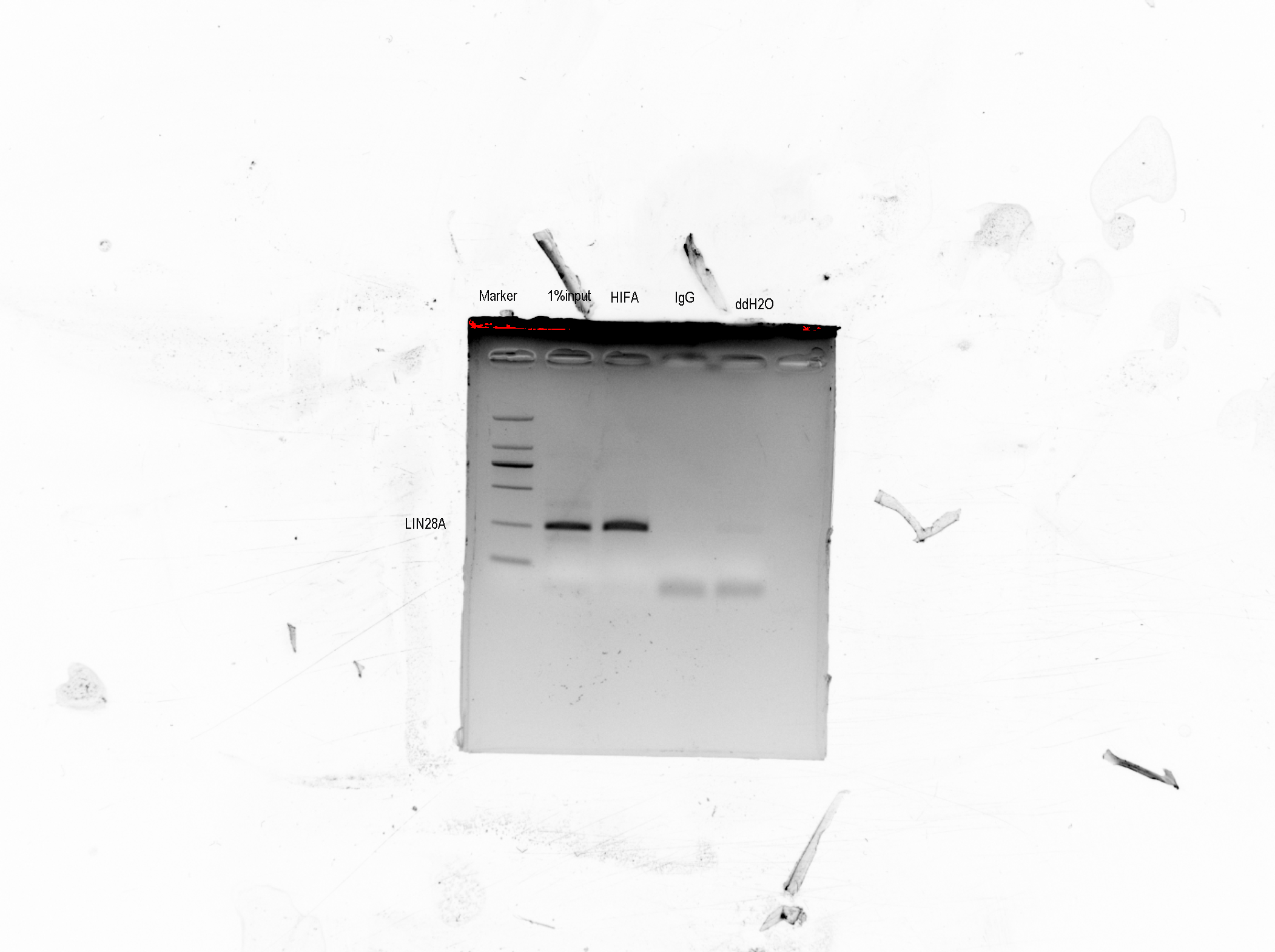

Supplement: Supplementary file 5 [file Data_Sheet_5.ZIP › FIG-1G/LIN28A1.tif]

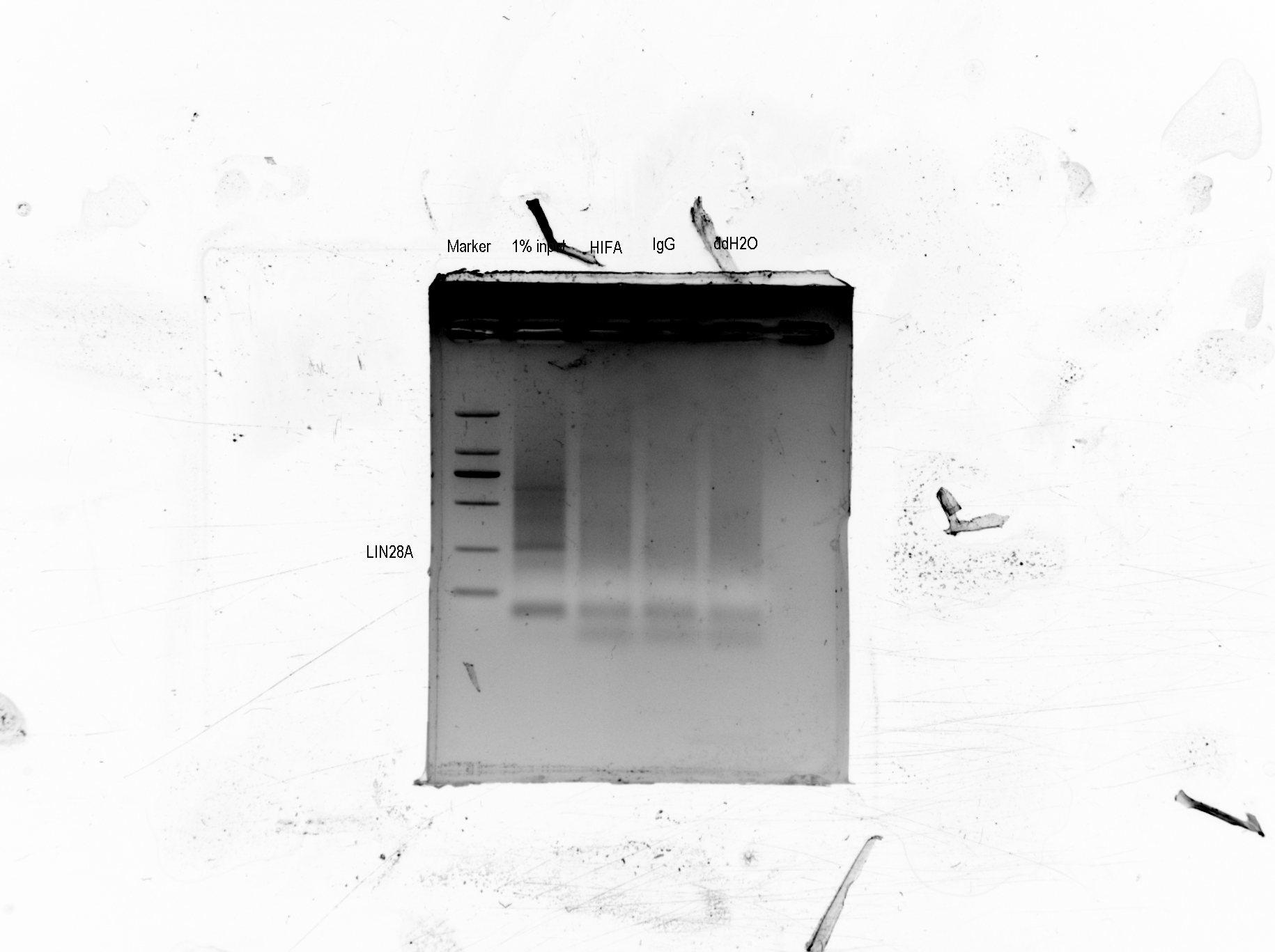

Supplement: Supplementary file 5 [file Data_Sheet_5.ZIP › FIG-1G/LIN28A2.tif]

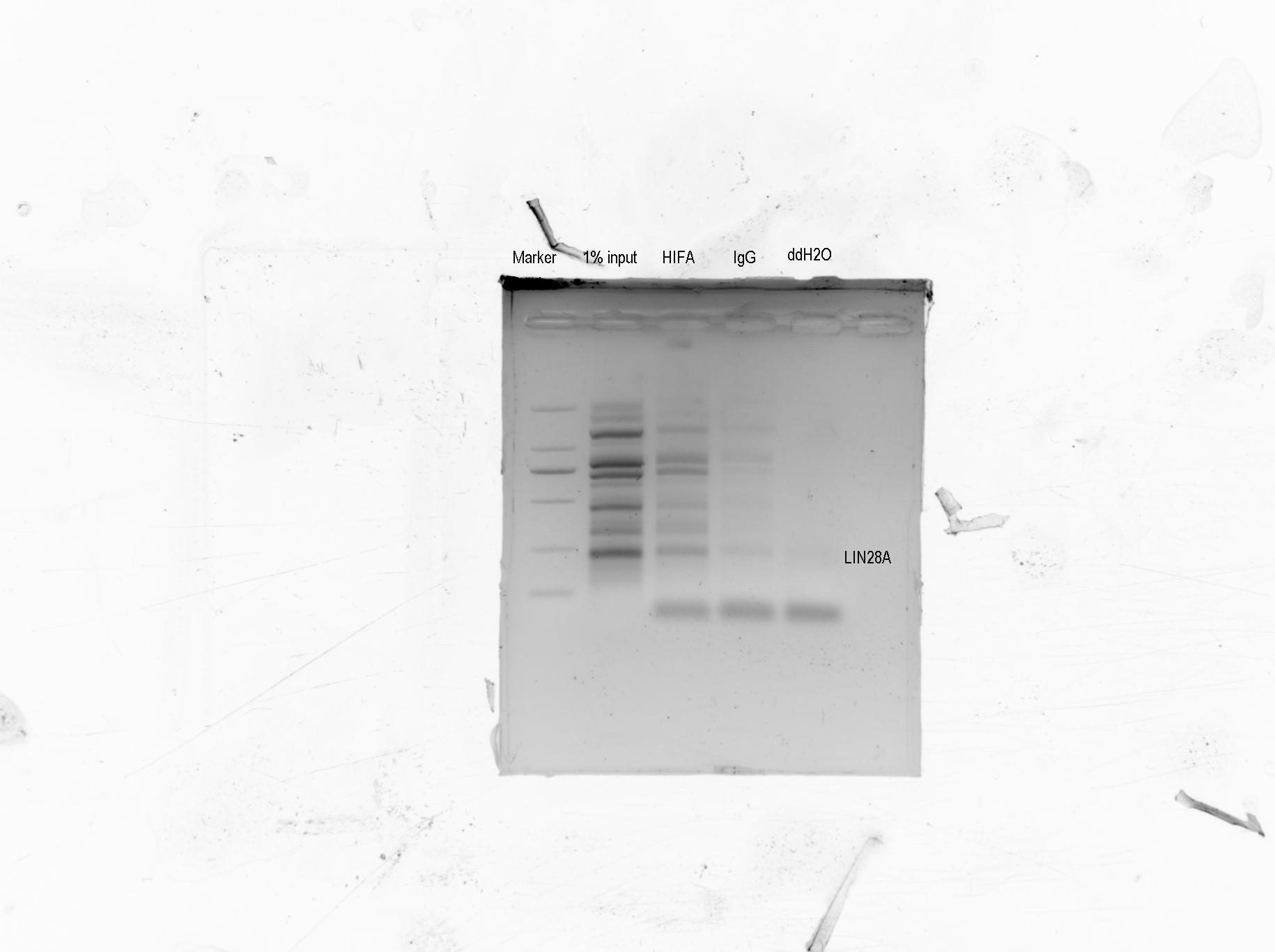

Supplement: Supplementary file 5 [file Data_Sheet_5.ZIP › FIG-1G/LIN28A3.tif]

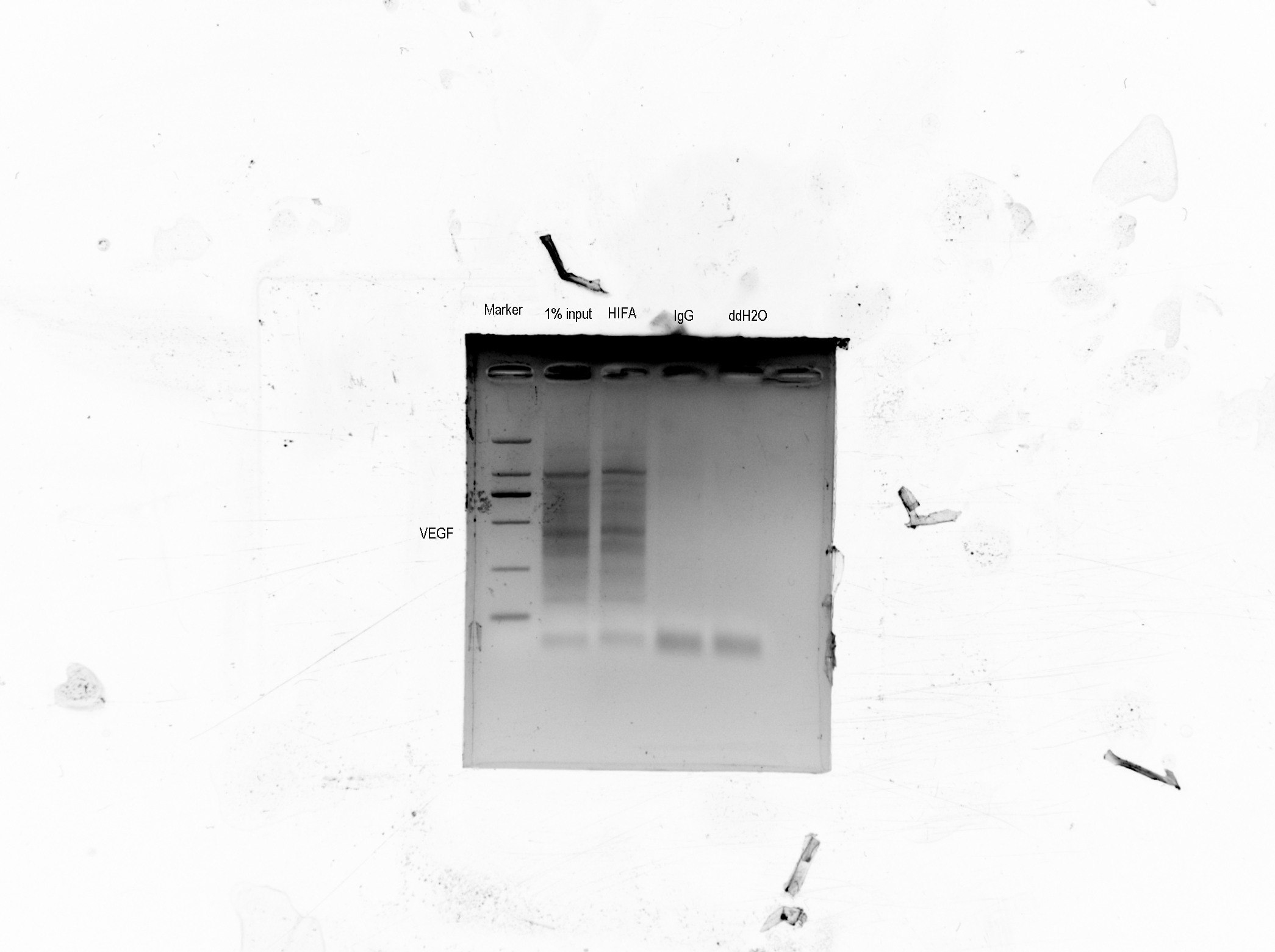

Supplement: Supplementary file 5 [file Data_Sheet_5.ZIP › FIG-1G/VEGF.tif]

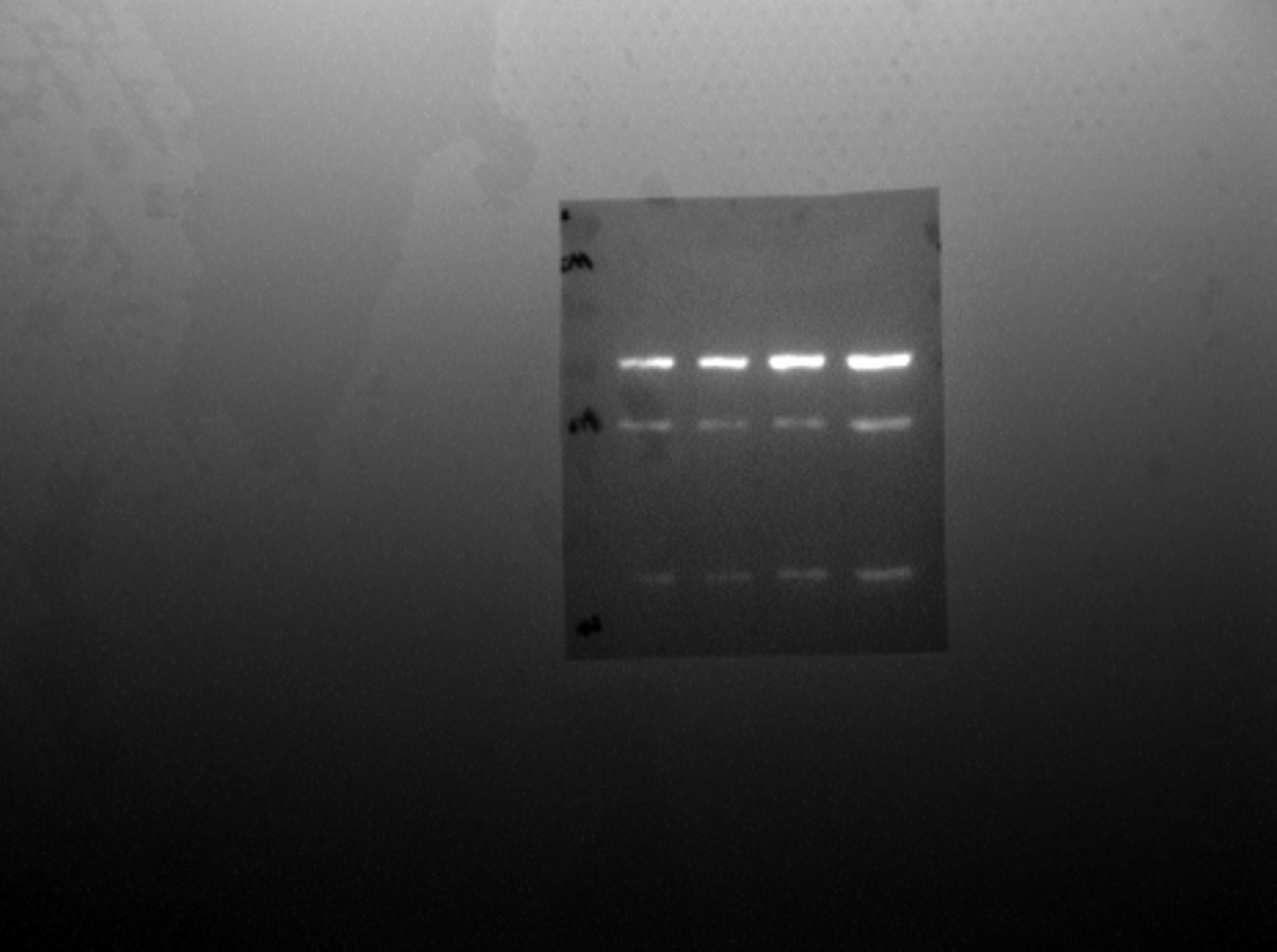

Supplement: Supplementary file 6 [file Data_Sheet_6.ZIP › FIG-2B/membrane with marker- ACTIN.tif]

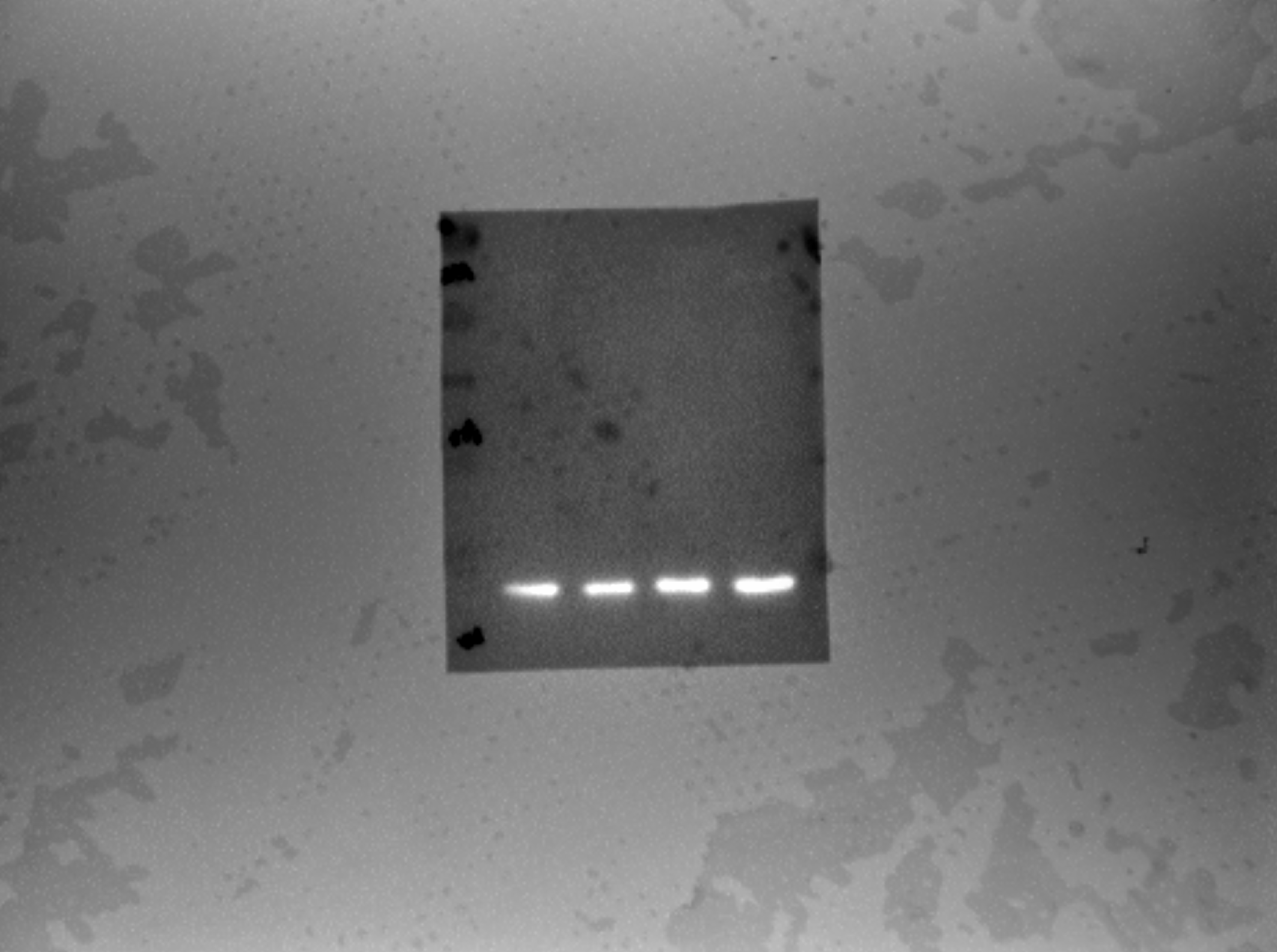

Supplement: Supplementary file 6 [file Data_Sheet_6.ZIP › FIG-2B/membrane with marker-LIN28A.tif]

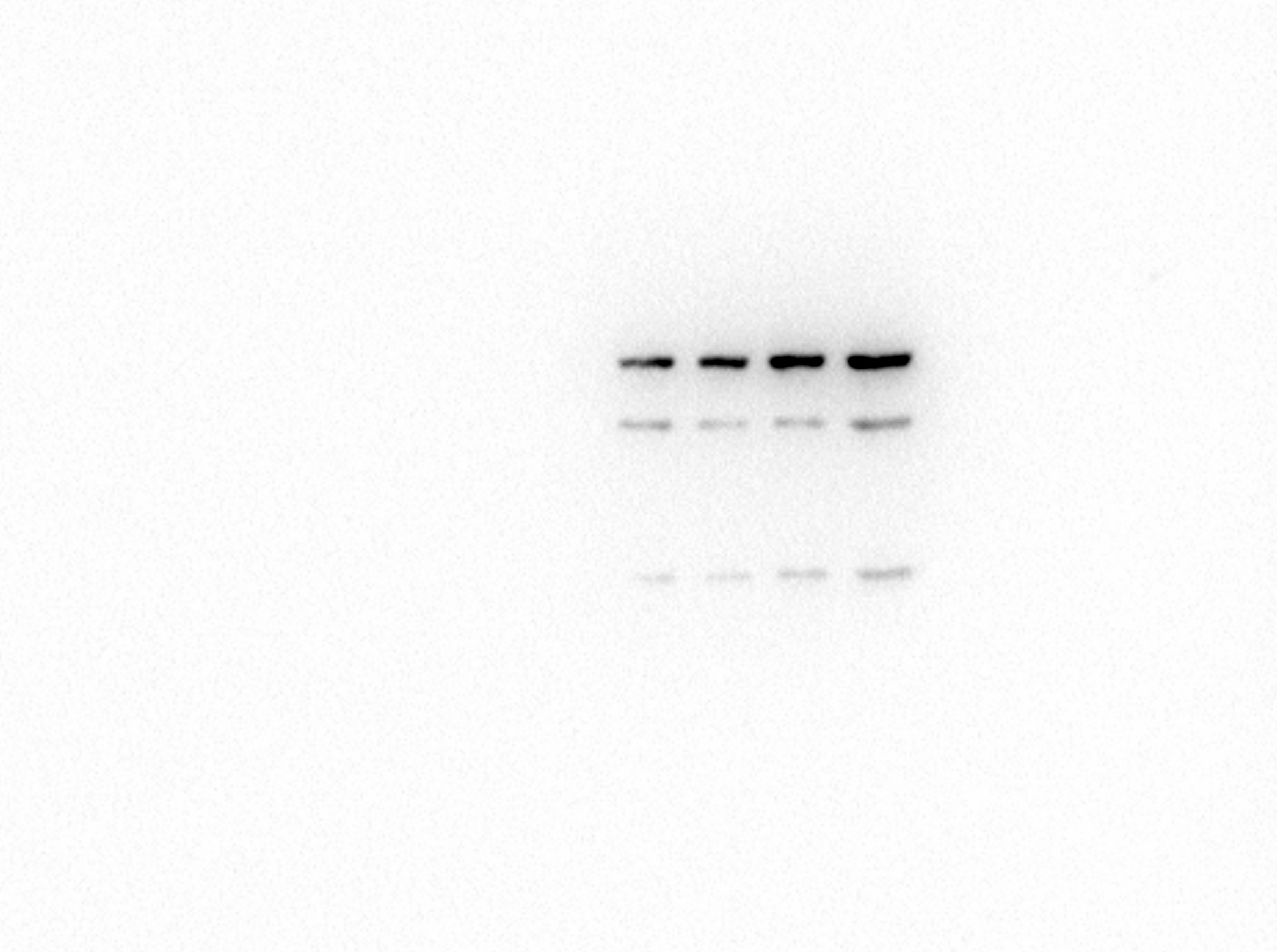

Supplement: Supplementary file 6 [file Data_Sheet_6.ZIP › FIG-2B/membrane-ACTIN.tif]

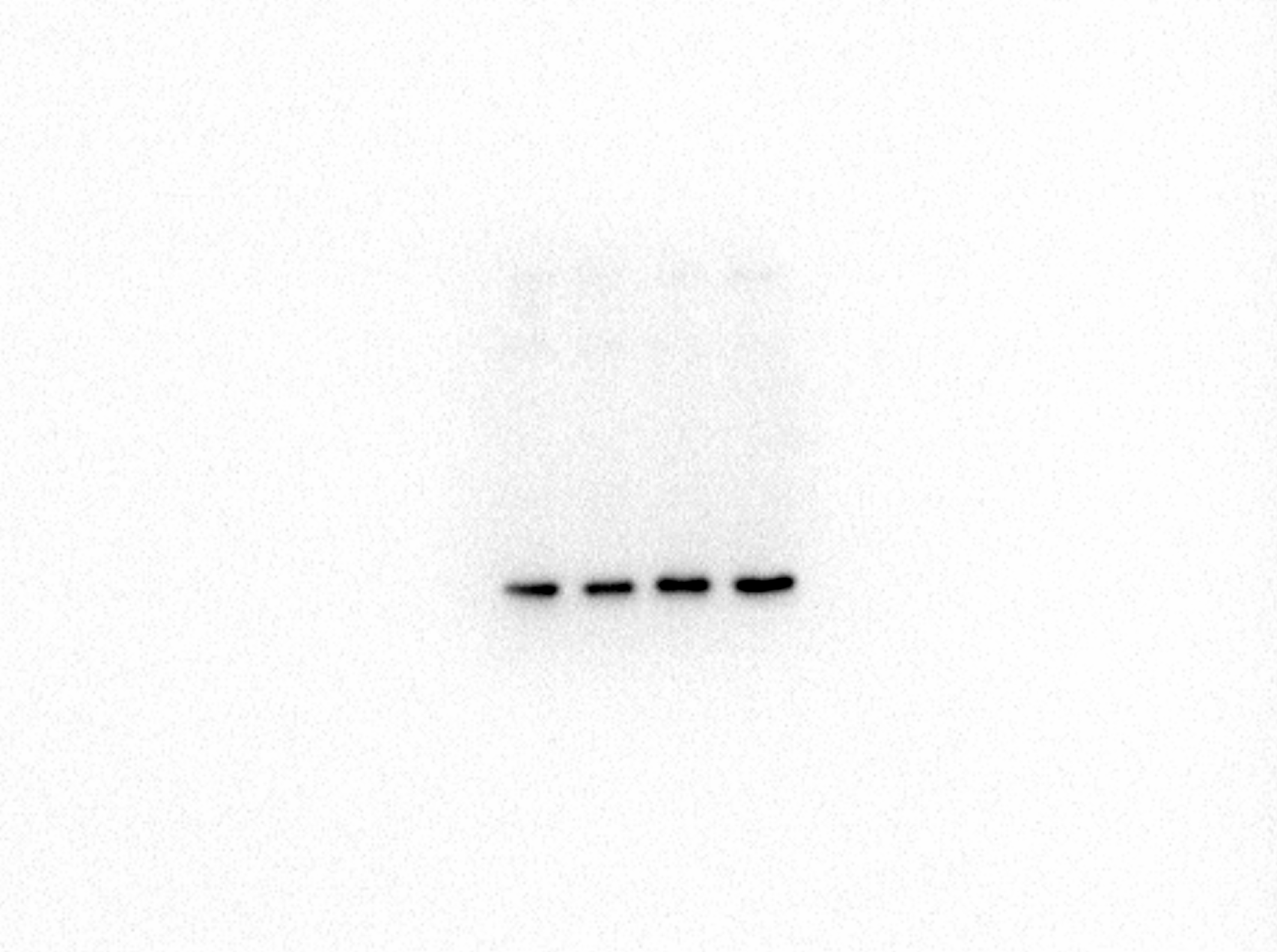

Supplement: Supplementary file 6 [file Data_Sheet_6.ZIP › FIG-2B/membrane-LIN28A.tif]

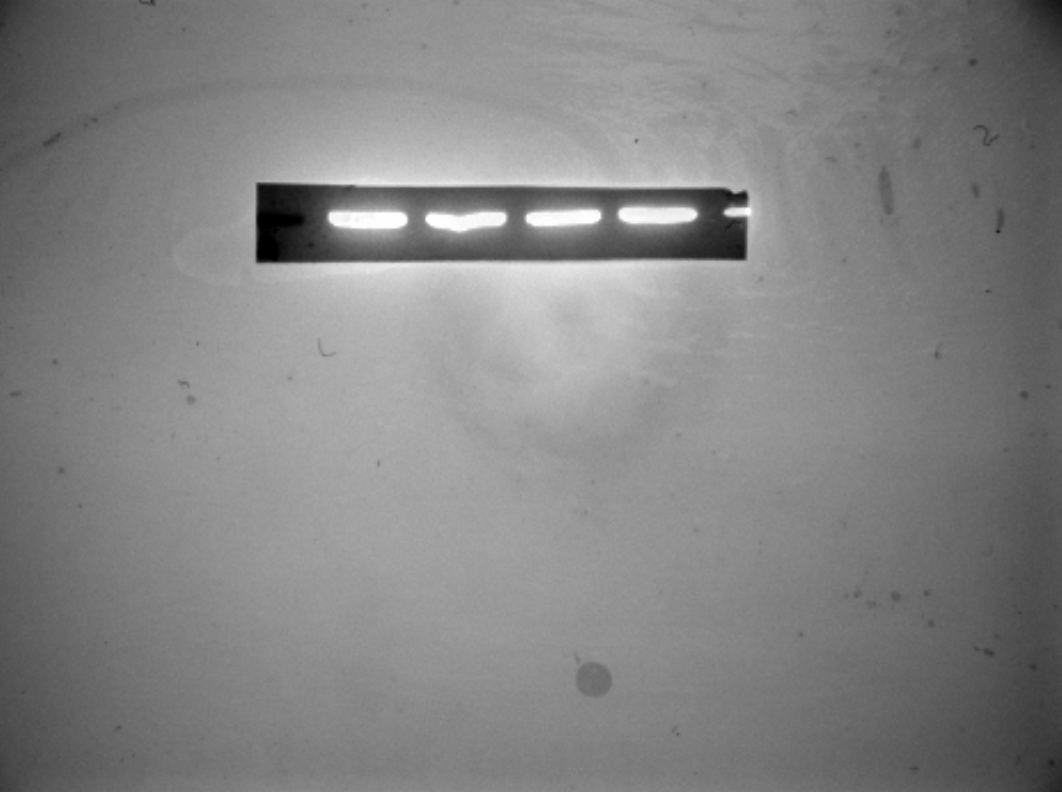

Supplement: Supplementary file 6 [file Data_Sheet_6.ZIP › FIG-2C/membrane with marker-ACTIN.tif]

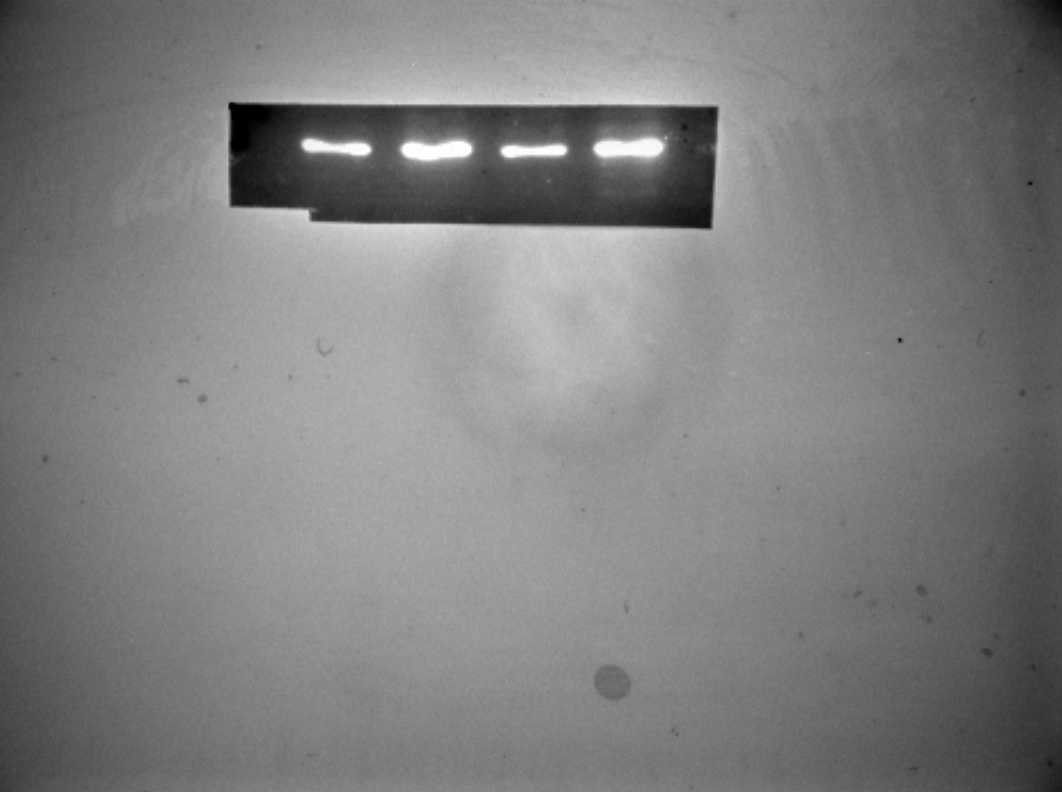

Supplement: Supplementary file 6 [file Data_Sheet_6.ZIP › FIG-2C/membrane with marker-LIN28A-.tif]

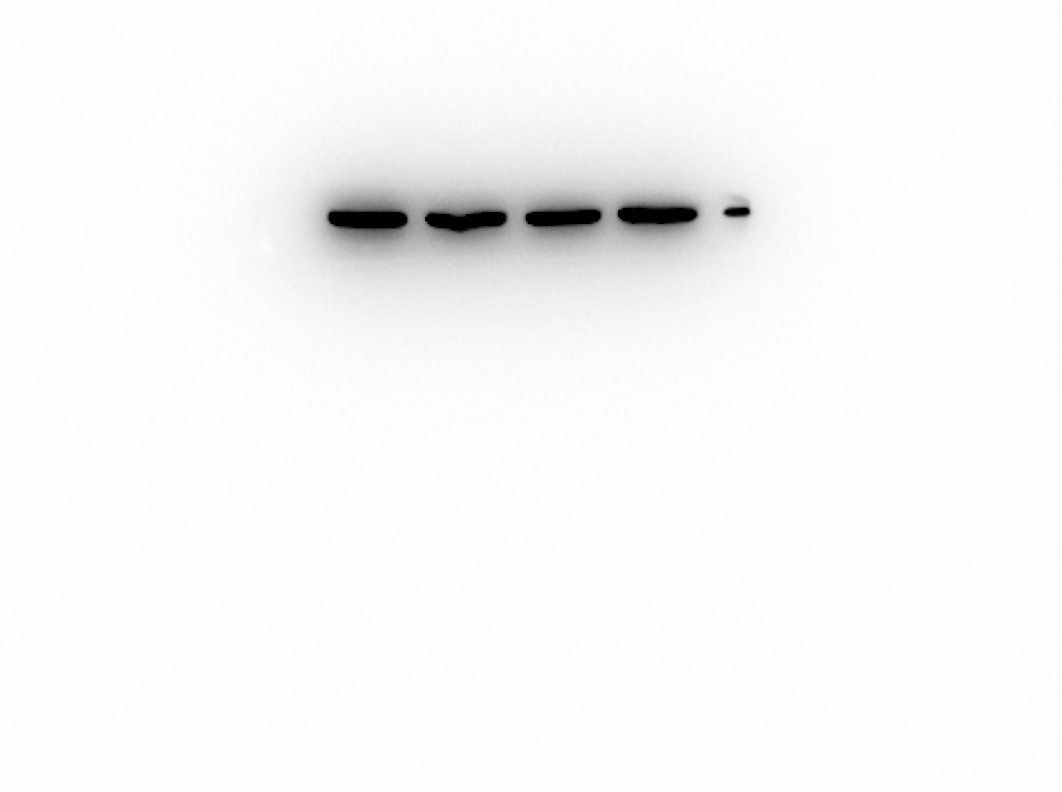

Supplement: Supplementary file 6 [file Data_Sheet_6.ZIP › FIG-2C/membrane-ACTIN.tif]

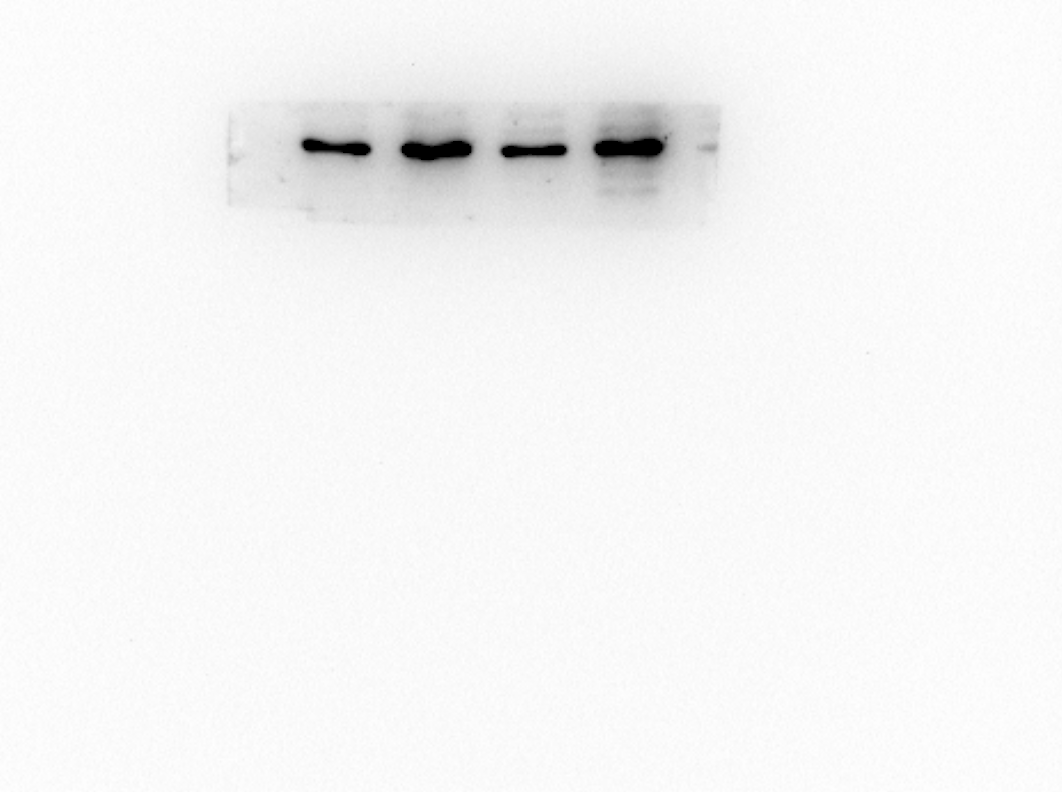

Supplement: Supplementary file 6 [file Data_Sheet_6.ZIP › FIG-2C/membrane-LIN28A.tif]

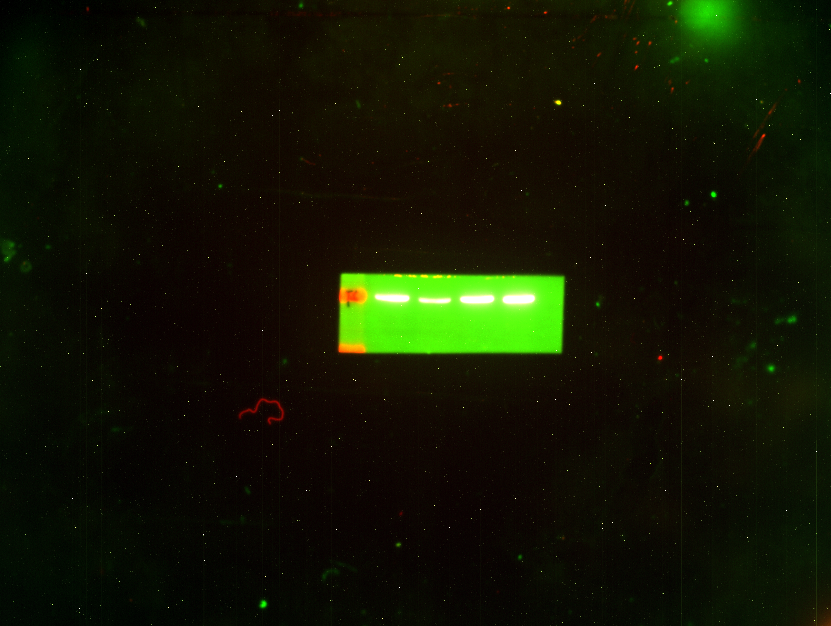

Supplement: Supplementary file 7 [file Data_Sheet_7.ZIP › FIG-5B/HCT116/membrane with marker-HCT116-dicer-tubulin_.png]

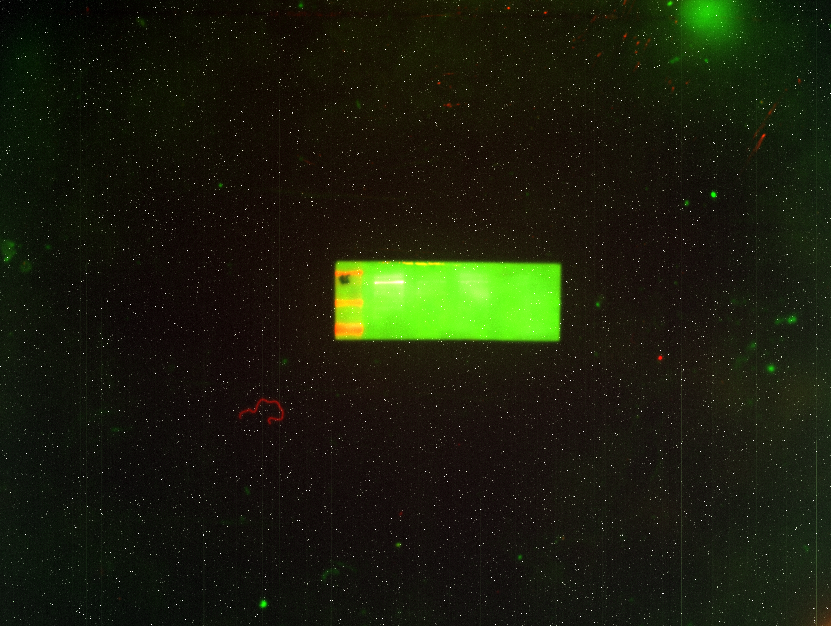

Supplement: Supplementary file 7 [file Data_Sheet_7.ZIP › FIG-5B/HCT116/membrane with marker-HCT116-dicer.png]

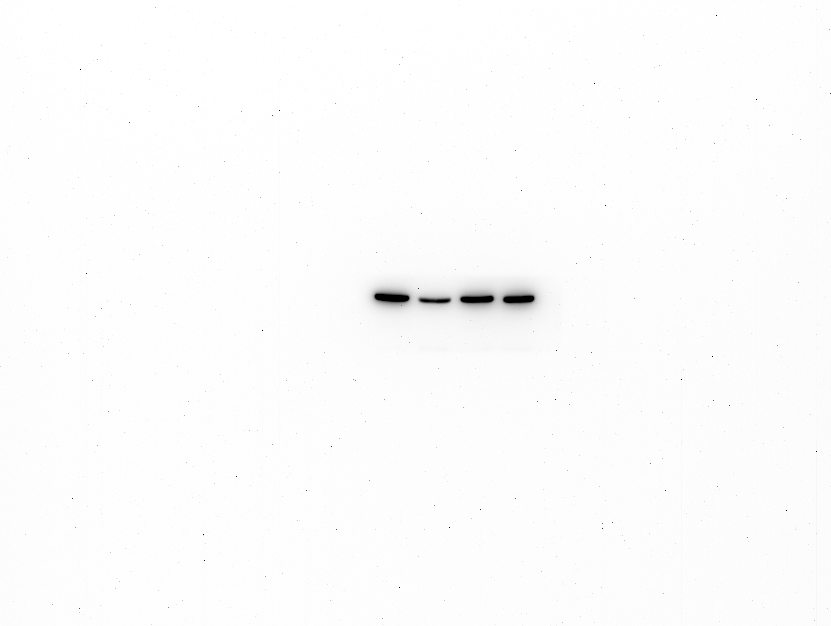

Supplement: Supplementary file 7 [file Data_Sheet_7.ZIP › FIG-5B/HCT116/membrane-HCT116-dicer-tubulin_8bit.png]

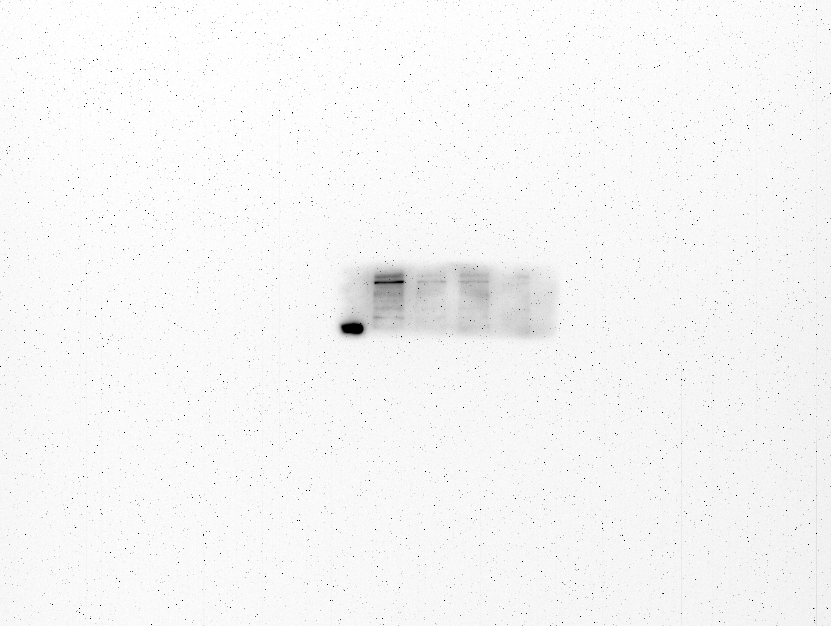

Supplement: Supplementary file 7 [file Data_Sheet_7.ZIP › FIG-5B/HCT116/membrane-HCT116-dicer_8bit.png]

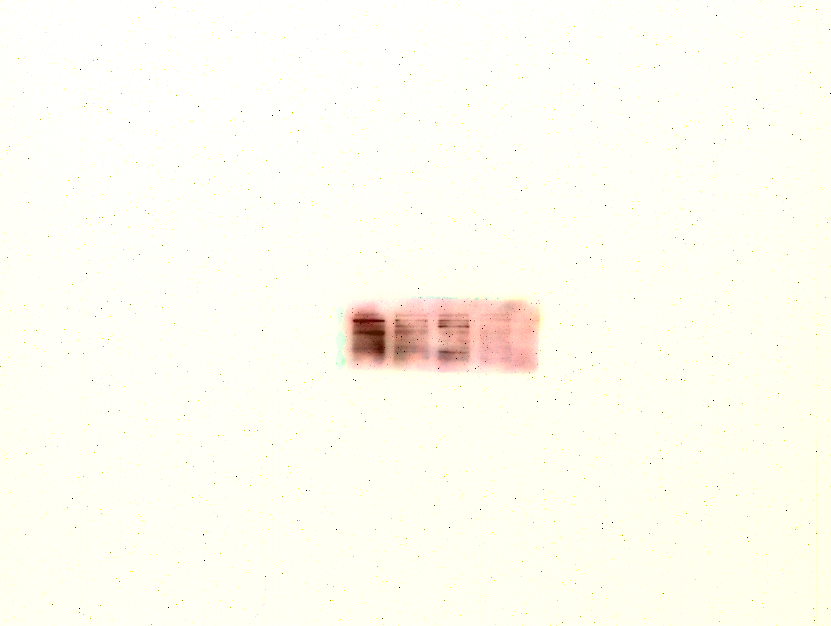

Supplement: Supplementary file 7 [file Data_Sheet_7.ZIP › FIG-5B/SW1116/membrane with marker-SW1116-dicer.png]

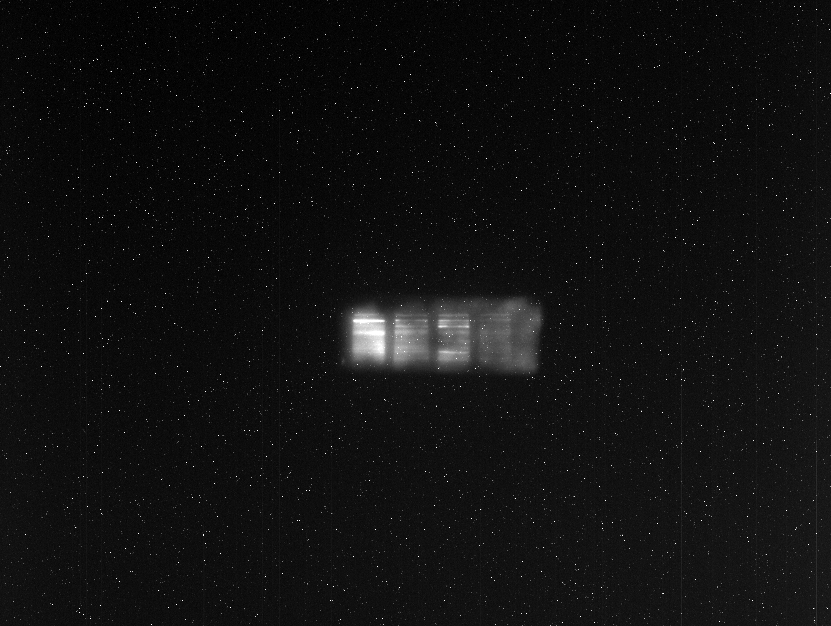

Supplement: Supplementary file 7 [file Data_Sheet_7.ZIP › FIG-5B/SW1116/membrane with marker-SW1116-dicer/2019-01-03_16-12-57_1_16bit.png]

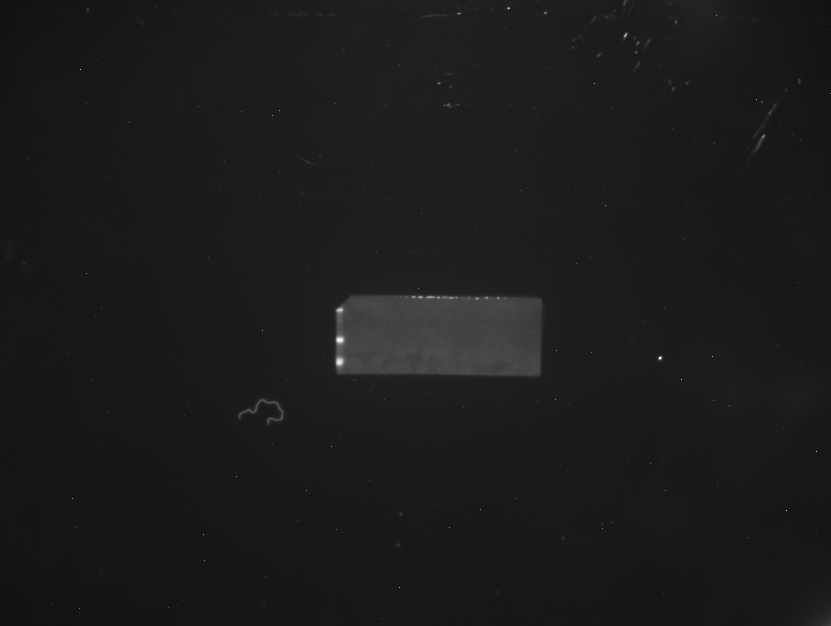

Supplement: Supplementary file 7 [file Data_Sheet_7.ZIP › FIG-5B/SW1116/membrane with marker-SW1116-dicer/2019-01-03_16-12-57_2_16bit.png]

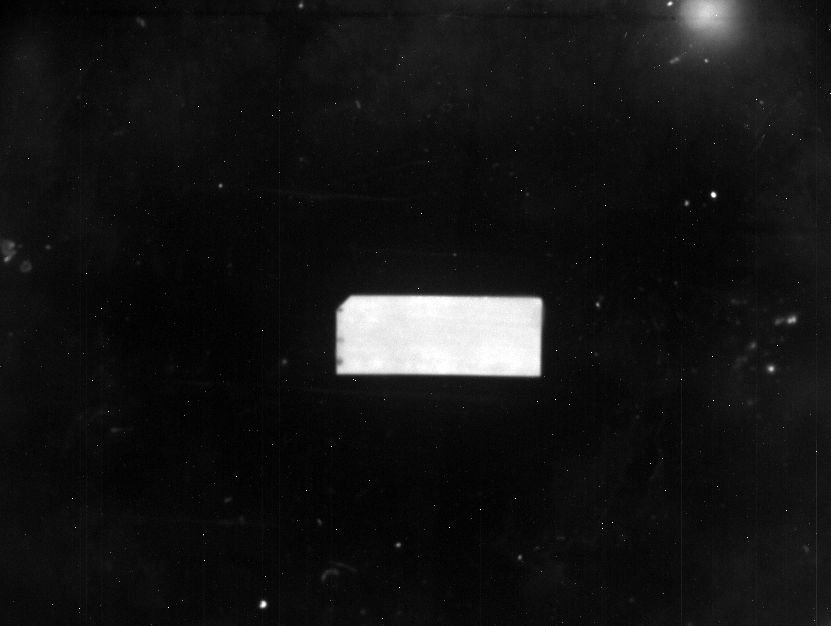

Supplement: Supplementary file 7 [file Data_Sheet_7.ZIP › FIG-5B/SW1116/membrane with marker-SW1116-dicer/2019-01-03_16-12-57_3_16bit.png]

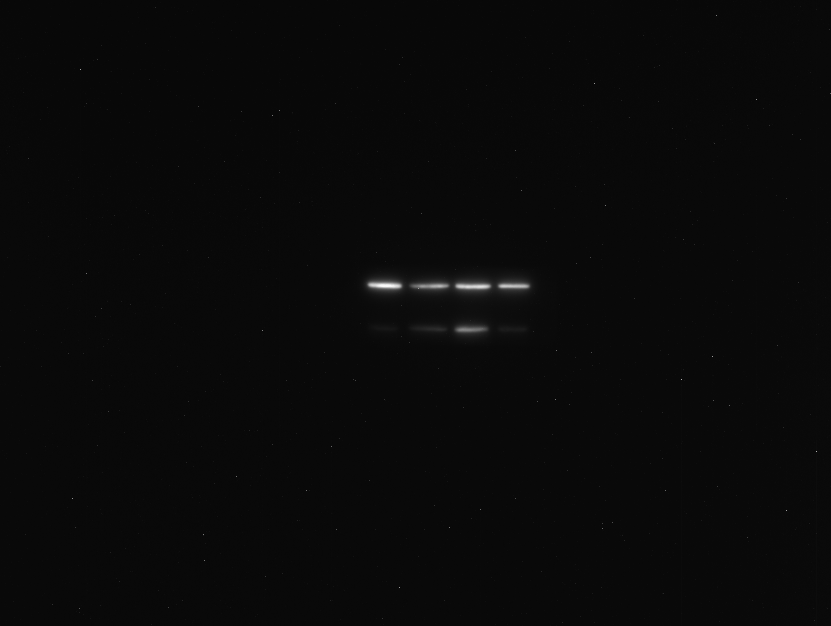

Supplement: Supplementary file 7 [file Data_Sheet_7.ZIP › FIG-5B/SW1116/membrane-SW1116-dicer-tubulin/2019-01-03_15-58-57 Sw-dicer-tubulin_1_16bit.png]

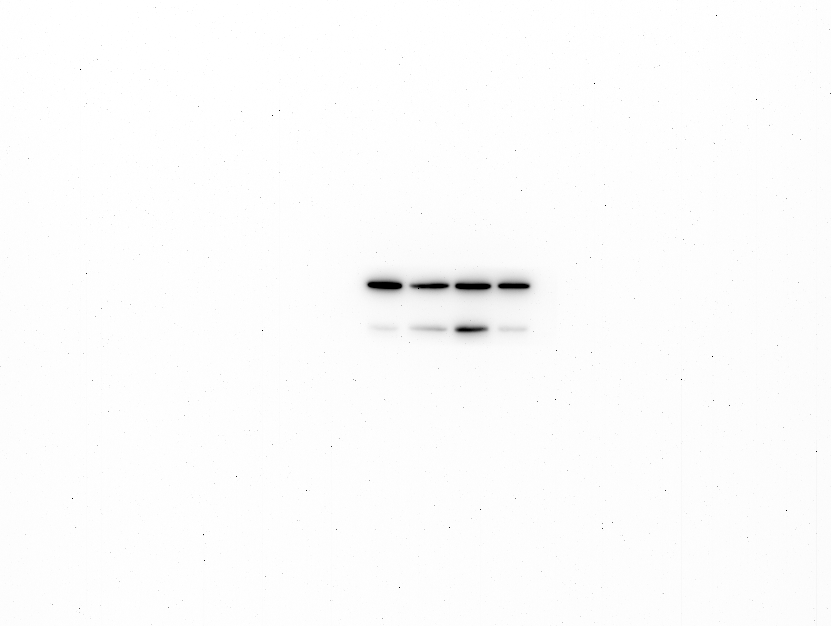

Supplement: Supplementary file 7 [file Data_Sheet_7.ZIP › FIG-5B/SW1116/membrane-SW1116-dicer-tubulin/2019-01-03_15-58-57 Sw-dicer-tubulin_8bit.png]

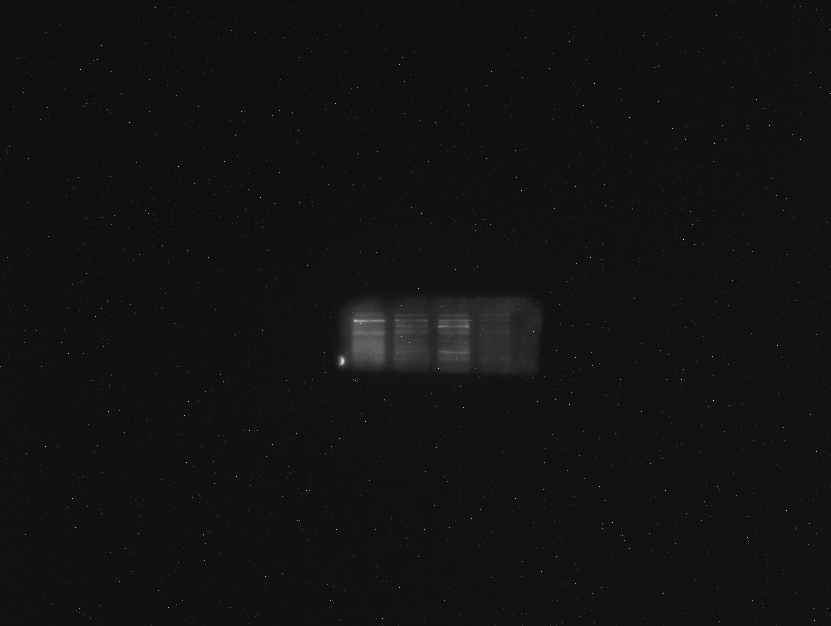

Supplement: Supplementary file 7 [file Data_Sheet_7.ZIP › FIG-5B/SW1116/membrane-SW1116-dicer/2019-01-03_16-11-15 Sw-dicer_1_16bit.png]

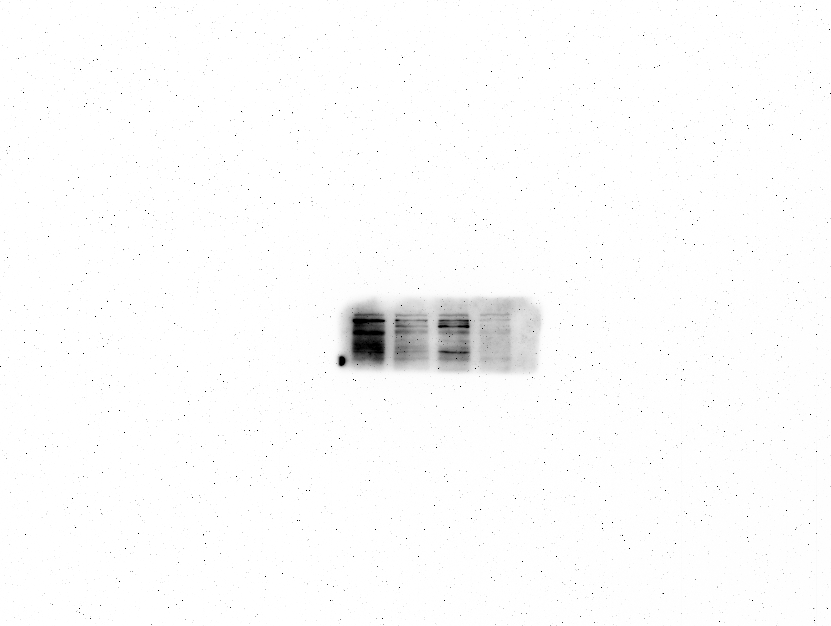

Supplement: Supplementary file 7 [file Data_Sheet_7.ZIP › FIG-5B/SW1116/membrane-SW1116-dicer/2019-01-03_16-11-15 Sw-dicer_8bit.png]

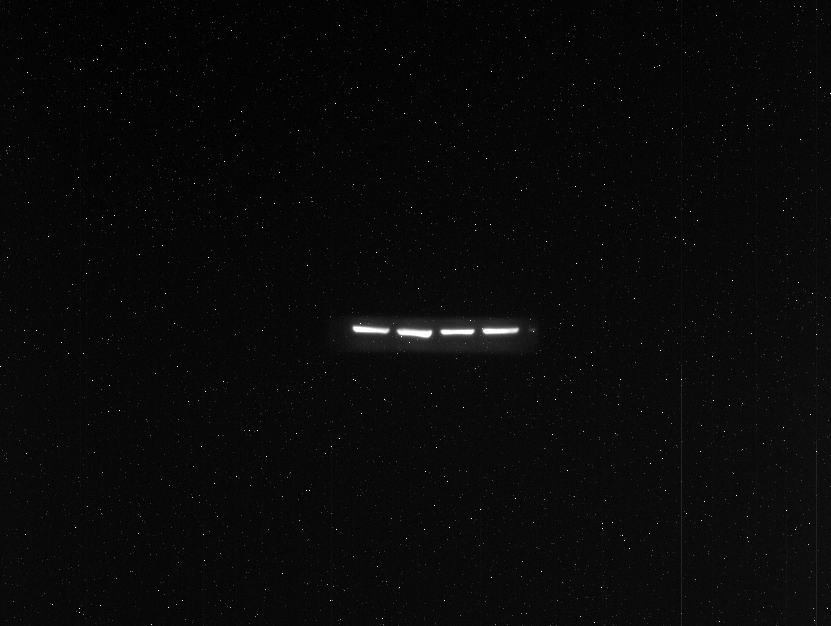

Supplement: Supplementary file 8 [file Data_Sheet_8.ZIP › FIG-5C/Actin/2016-12-06_13-33-19 METAP2-Actin_1_16bit.png]

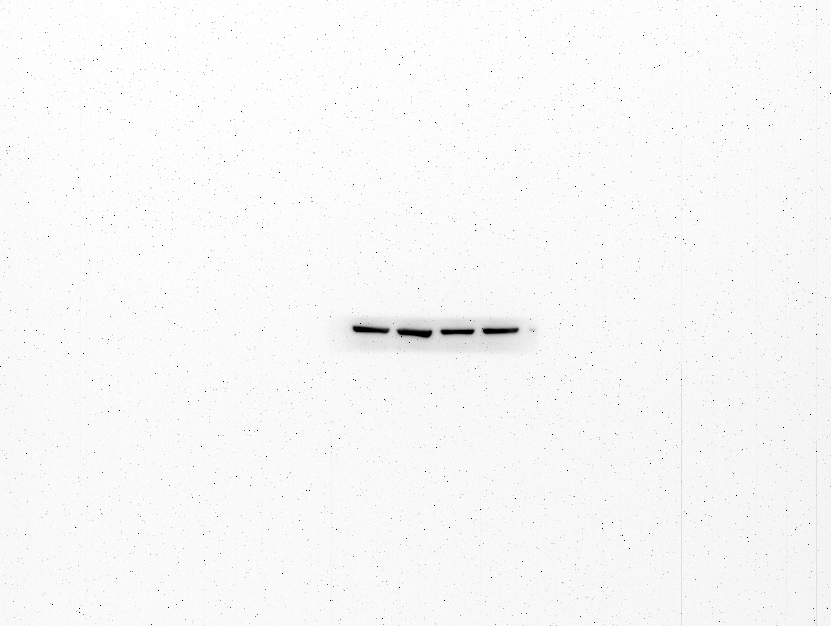

Supplement: Supplementary file 8 [file Data_Sheet_8.ZIP › FIG-5C/membrane Actin_8bit.png]

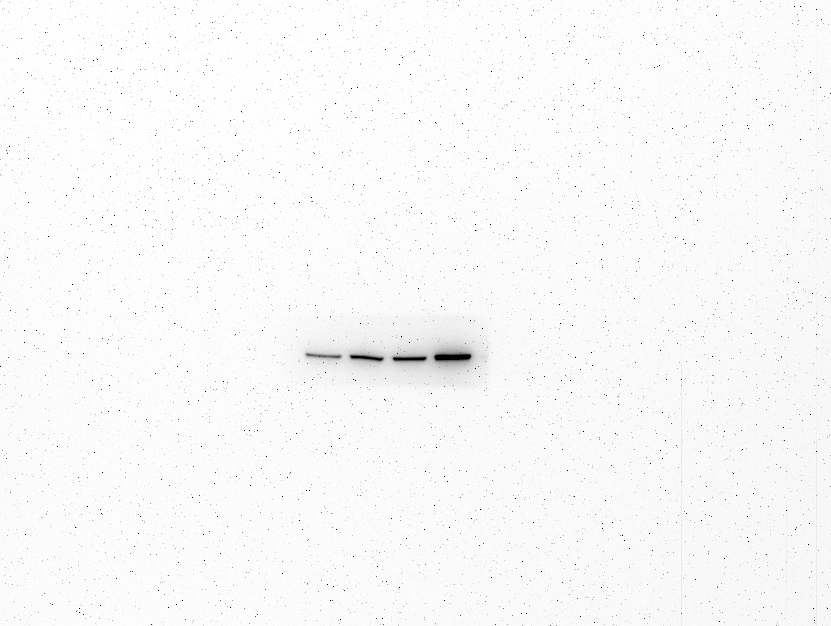

Supplement: Supplementary file 8 [file Data_Sheet_8.ZIP › FIG-5C/membrane- METAP2_8bit.png]

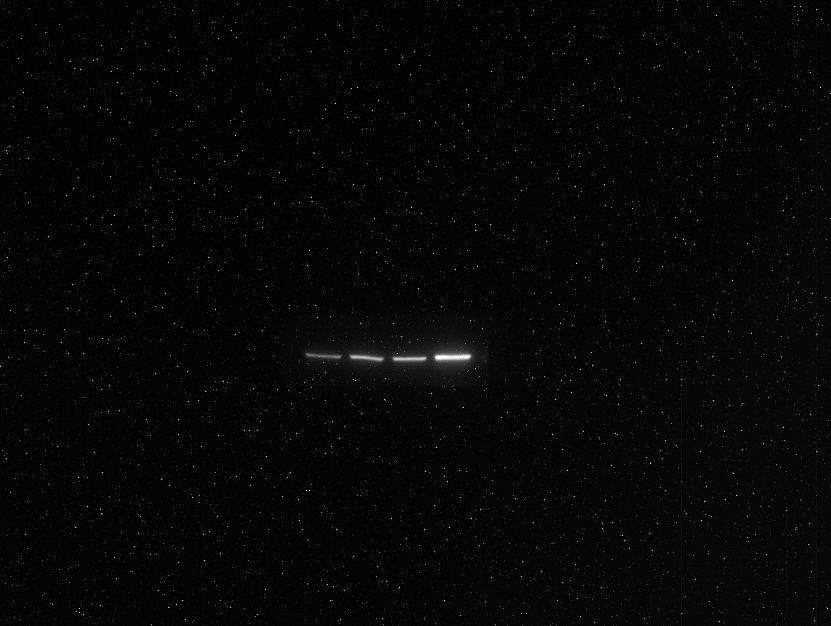

Supplement: Supplementary file 8 [file Data_Sheet_8.ZIP › FIG-5C/METAP2/2016-12-06_13-36-13 METAP2_1_16bit.png]

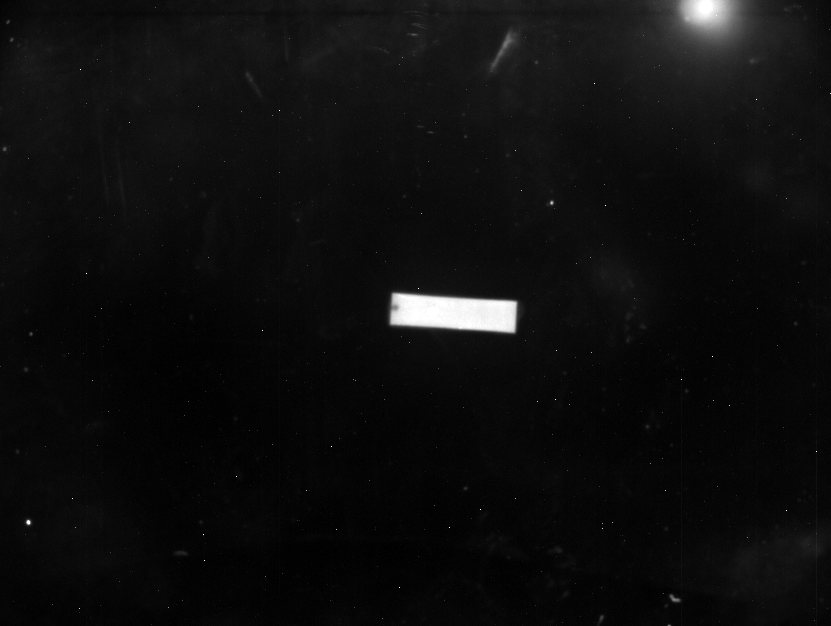

Supplement: Supplementary file 8 [file Data_Sheet_8.ZIP › FIG-5D/HCT116/membrane with marker-HCT116-actin/2019-04-20_14-44-59_3_16bit.png]

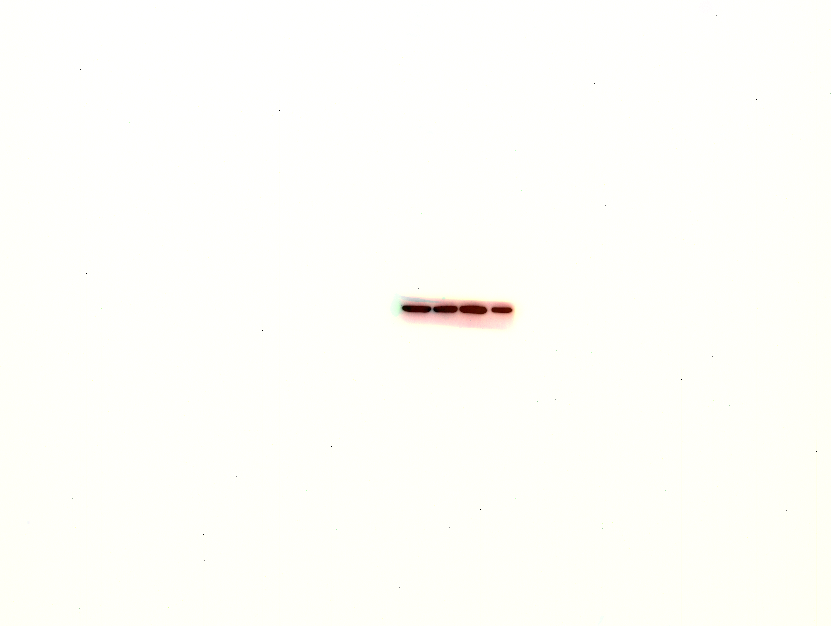

Supplement: Supplementary file 8 [file Data_Sheet_8.ZIP › FIG-5D/HCT116/membrane with marker-HCT116-actin/2019-04-20_14-44-59_8bit.png]

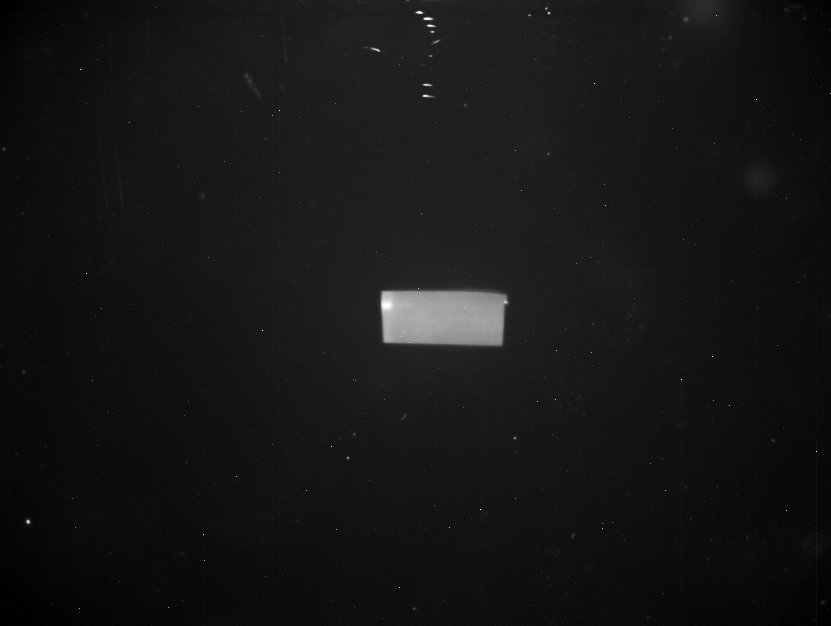

Supplement: Supplementary file 8 [file Data_Sheet_8.ZIP › FIG-5D/HCT116/membrane with marker-HCT116-LIN28A/2019-04-20_14-52-29_2_16bit.png]

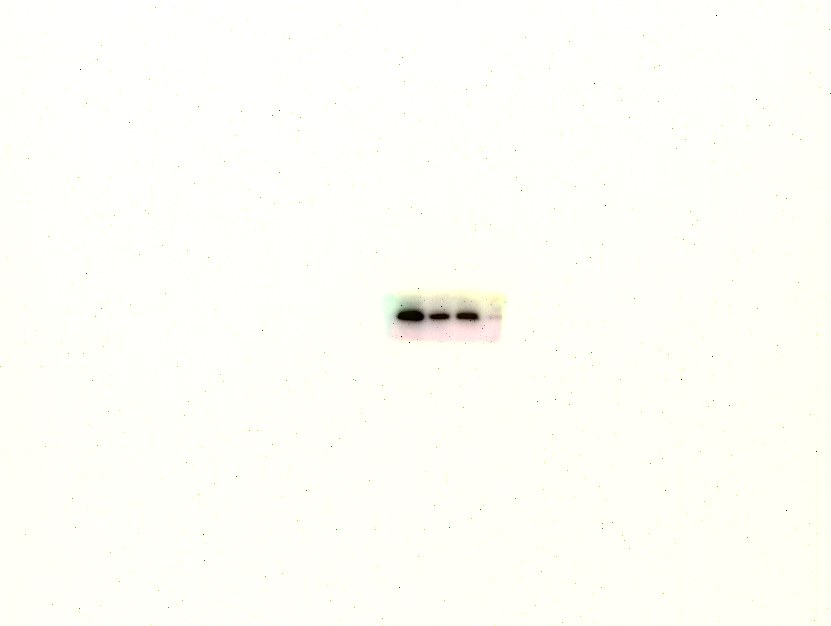

Supplement: Supplementary file 8 [file Data_Sheet_8.ZIP › FIG-5D/HCT116/membrane with marker-HCT116-LIN28A/2019-04-20_14-52-29_8bit.png]

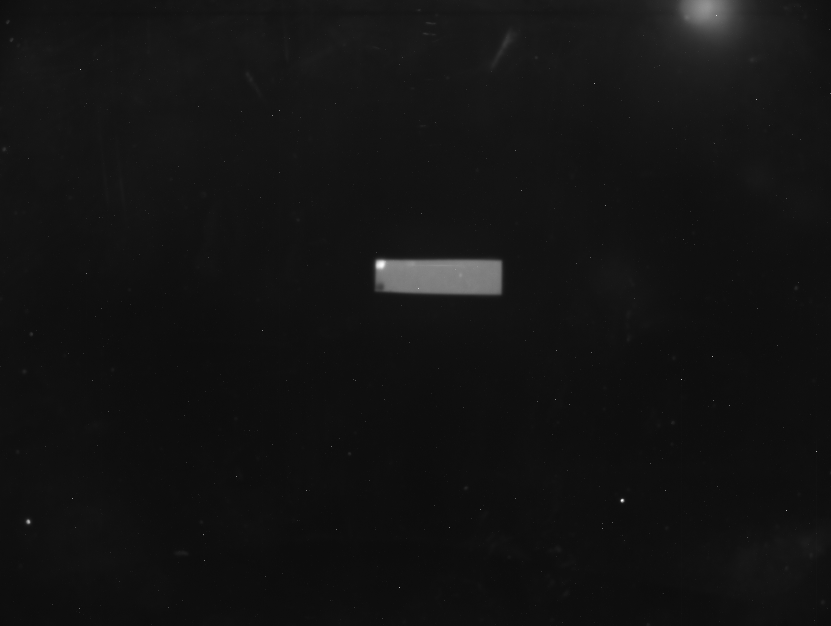

Supplement: Supplementary file 8 [file Data_Sheet_8.ZIP › FIG-5D/HCT116/membrane with marker-HCT116-METAP2/2019-04-20_15-03-13_3_16bit.png]

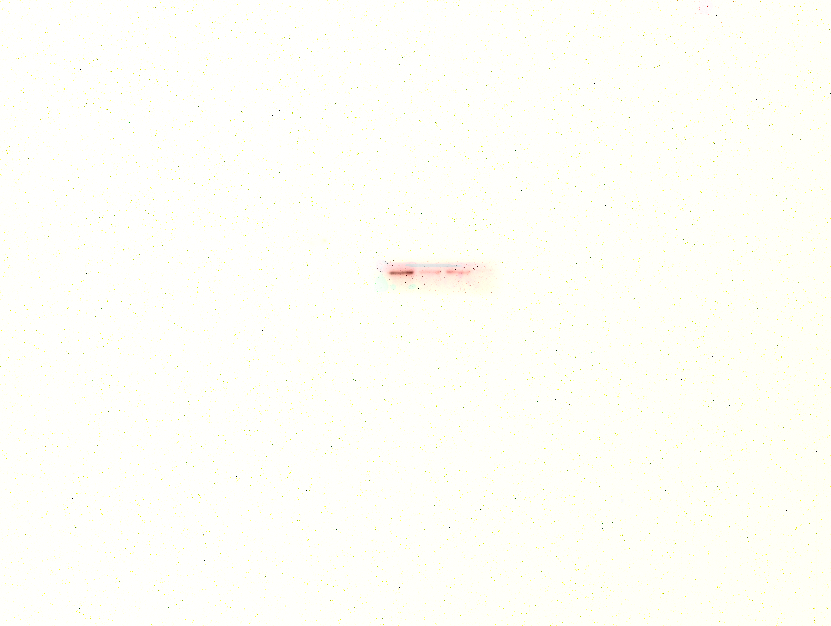

Supplement: Supplementary file 8 [file Data_Sheet_8.ZIP › FIG-5D/HCT116/membrane with marker-HCT116-METAP2/2019-04-20_15-03-13_8bit.png]

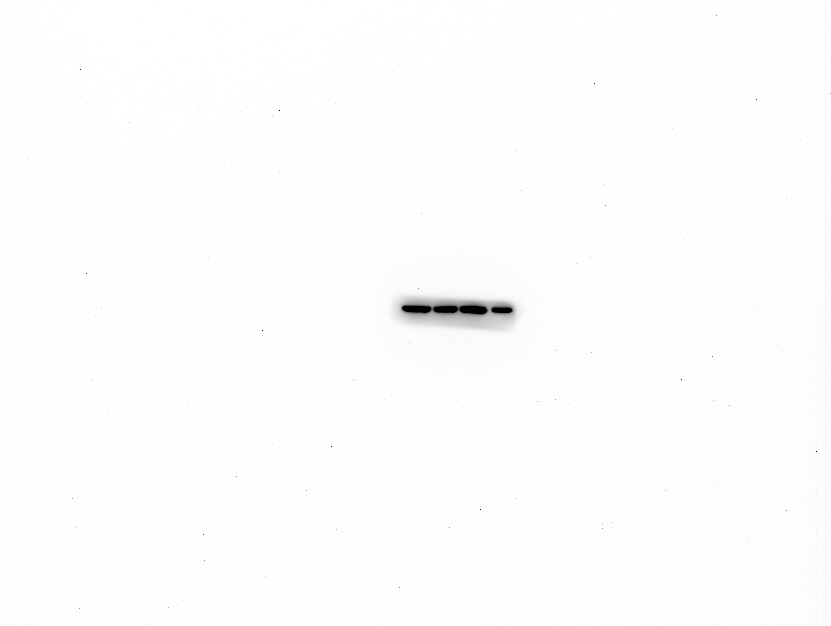

Supplement: Supplementary file 8 [file Data_Sheet_8.ZIP › FIG-5D/HCT116/membrane-HCT116-actin.png]

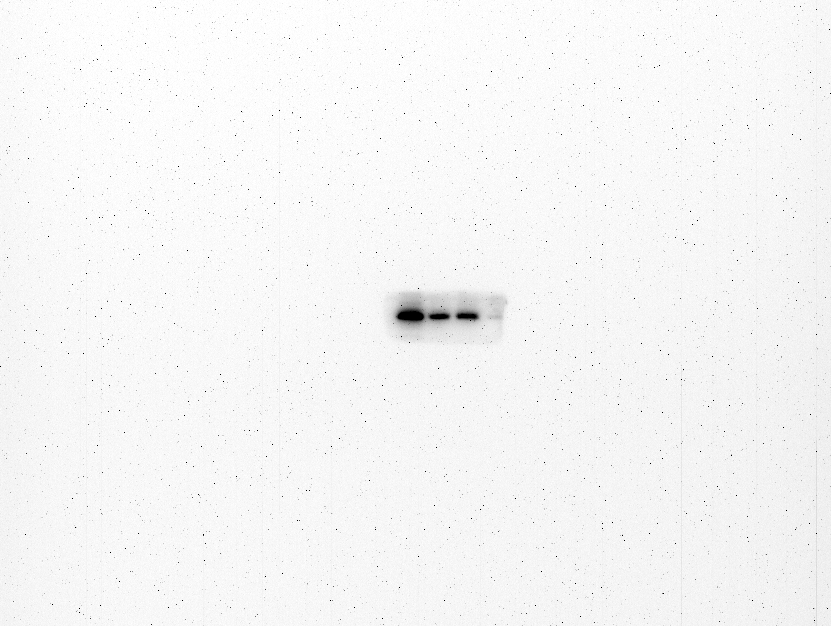

Supplement: Supplementary file 8 [file Data_Sheet_8.ZIP › FIG-5D/HCT116/membrane-HCT116-LIN28A.png]

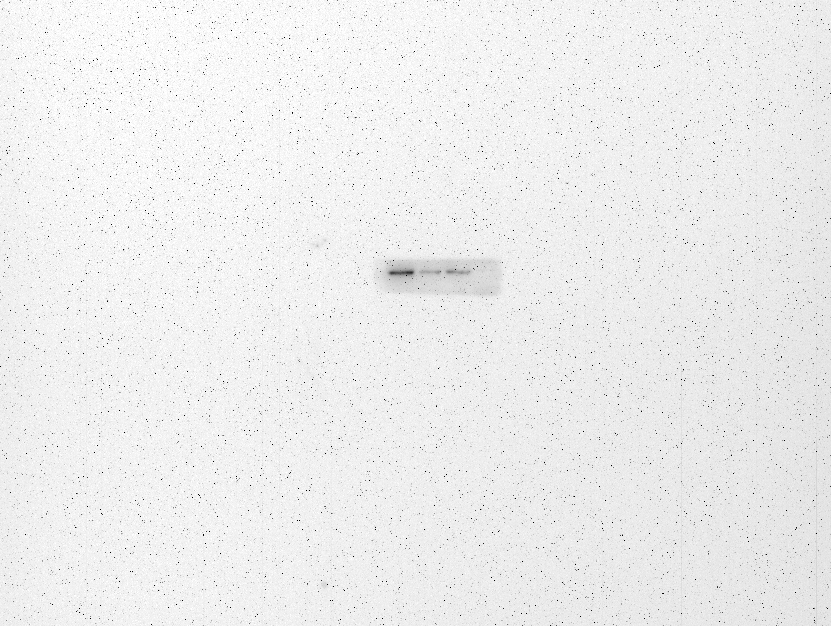

Supplement: Supplementary file 8 [file Data_Sheet_8.ZIP › FIG-5D/HCT116/membrane-HCT116-METAP2.png]

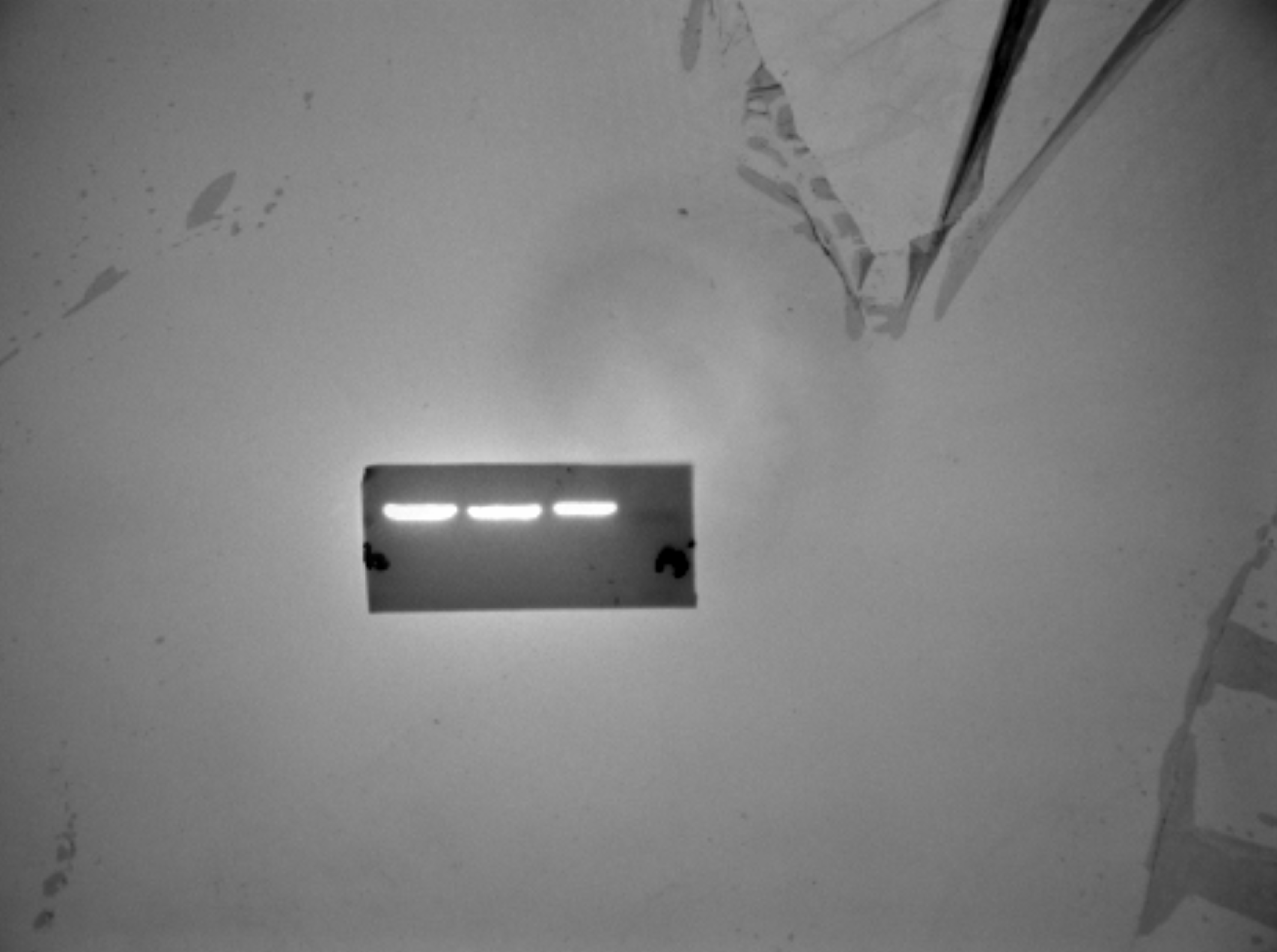

Supplement: Supplementary file 8 [file Data_Sheet_8.ZIP › FIG-5D/SW1116/membrane with marker-ACTIN.tif]

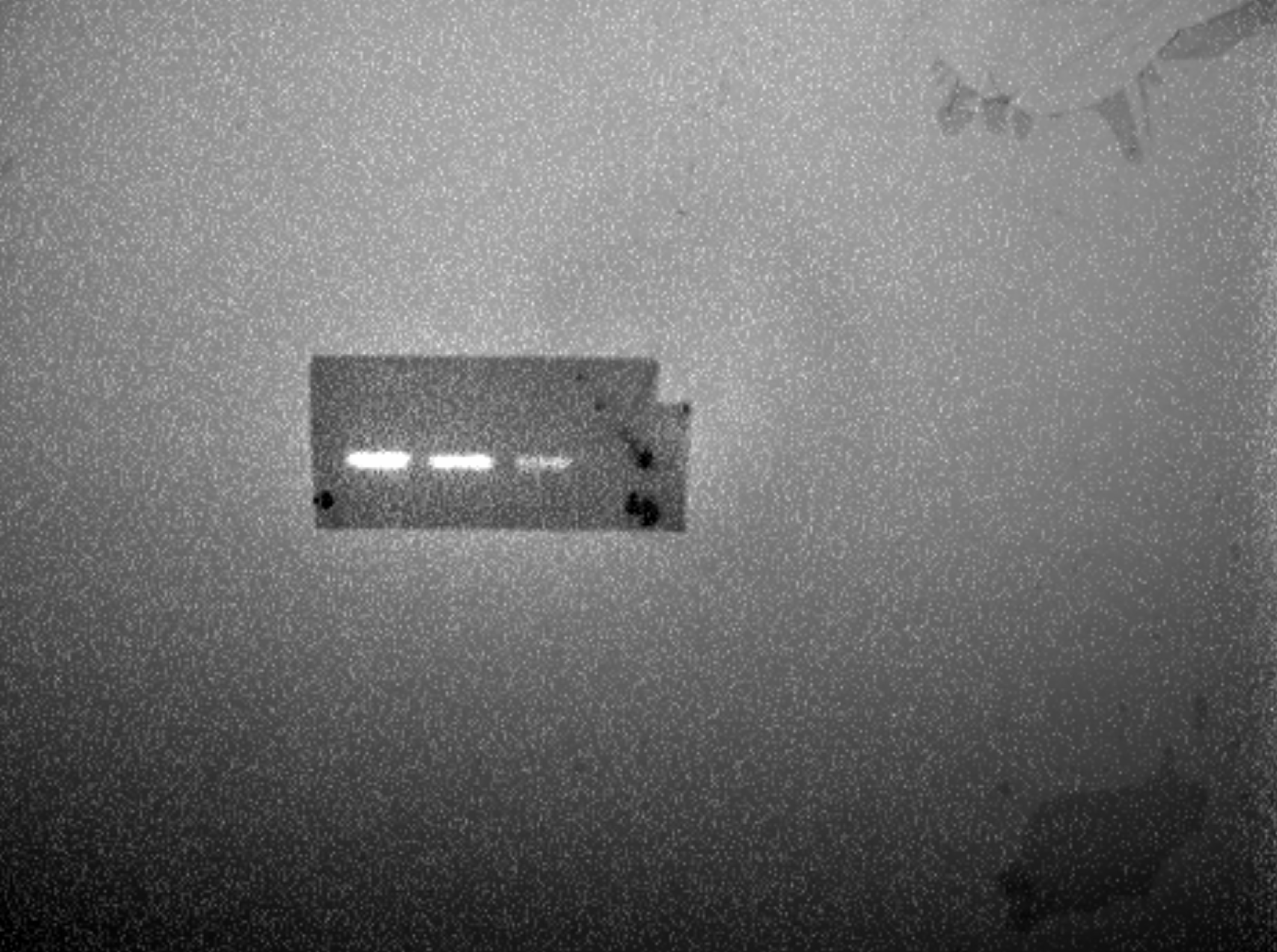

Supplement: Supplementary file 8 [file Data_Sheet_8.ZIP › FIG-5D/SW1116/membrane with marker-LIN28A.tif]

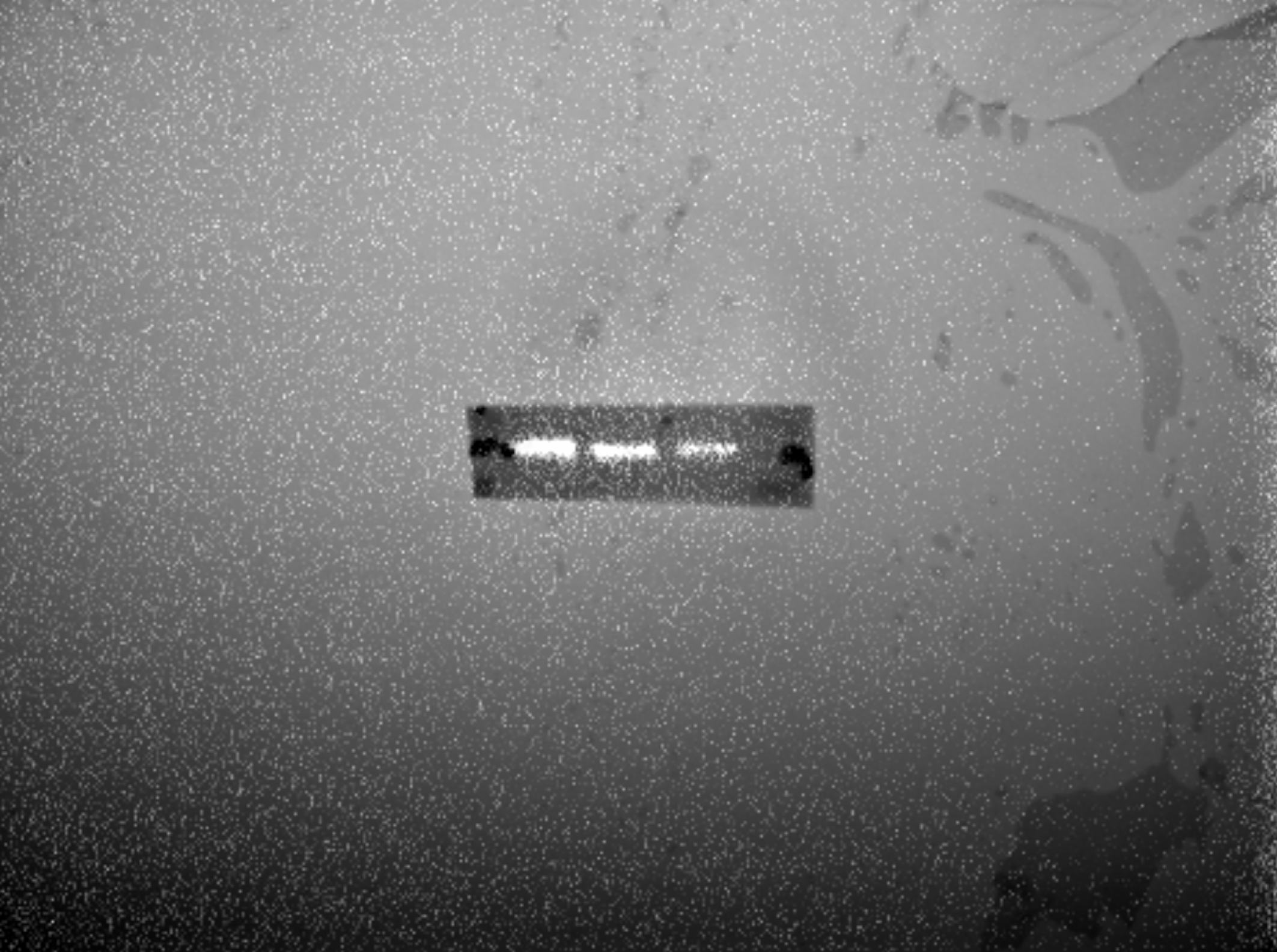

Supplement: Supplementary file 8 [file Data_Sheet_8.ZIP › FIG-5D/SW1116/membrane with marker-METAP2.tif]

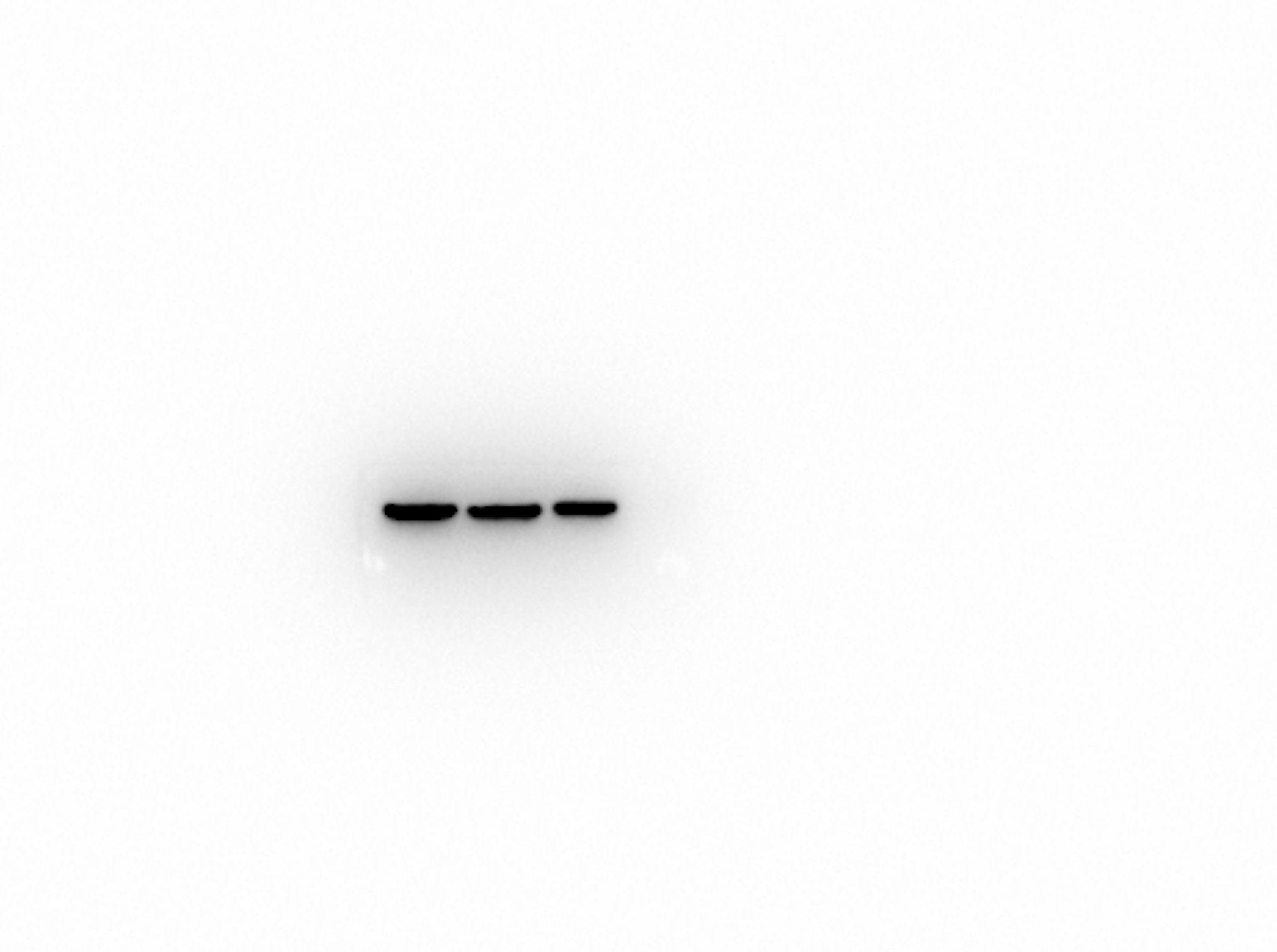

Supplement: Supplementary file 8 [file Data_Sheet_8.ZIP › FIG-5D/SW1116/membrane-ACTIN.tif]

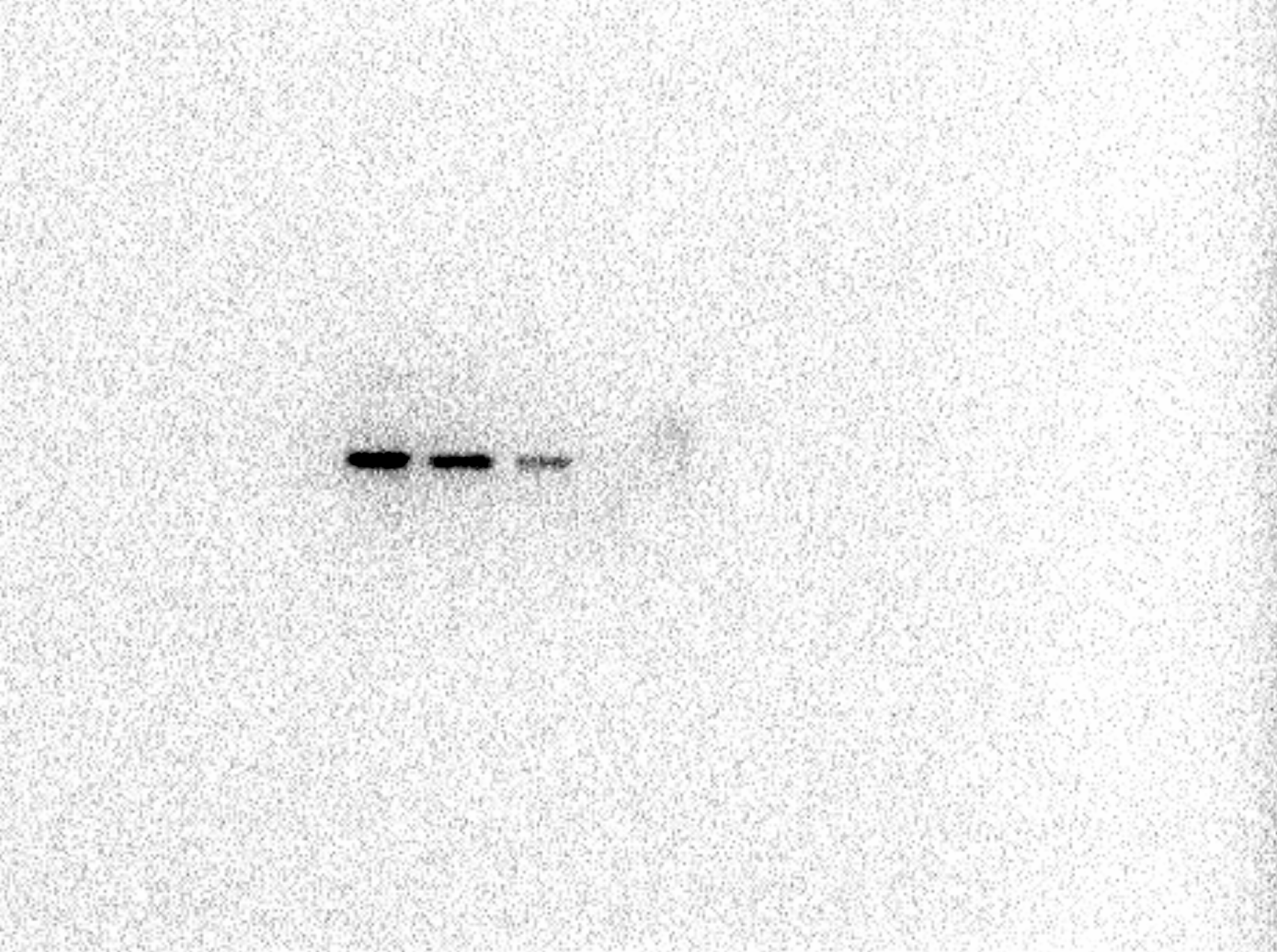

Supplement: Supplementary file 8 [file Data_Sheet_8.ZIP › FIG-5D/SW1116/membrane-LIN28A.tif]

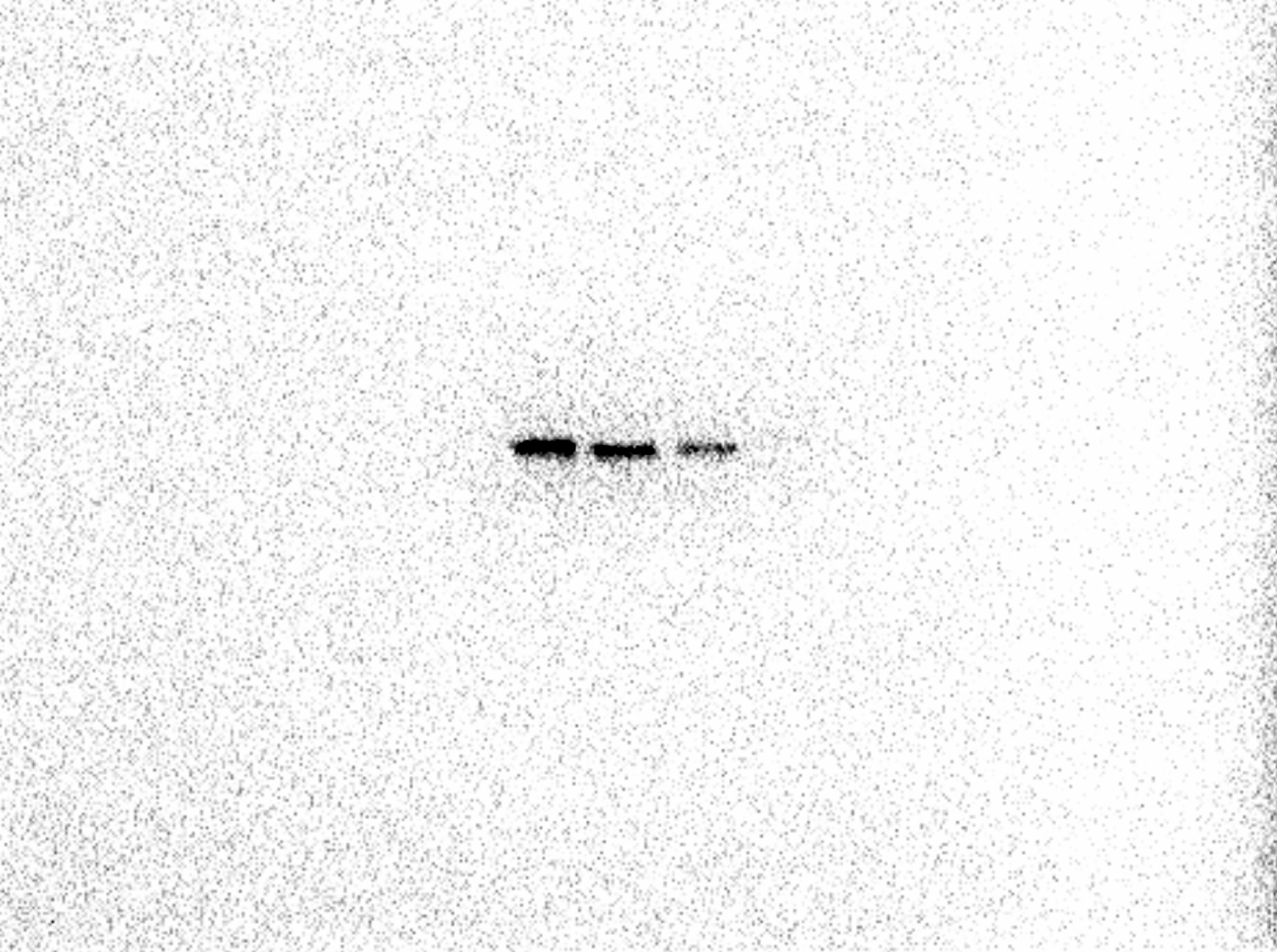

Supplement: Supplementary file 8 [file Data_Sheet_8.ZIP › FIG-5D/SW1116/membrane-METAP2.tif]

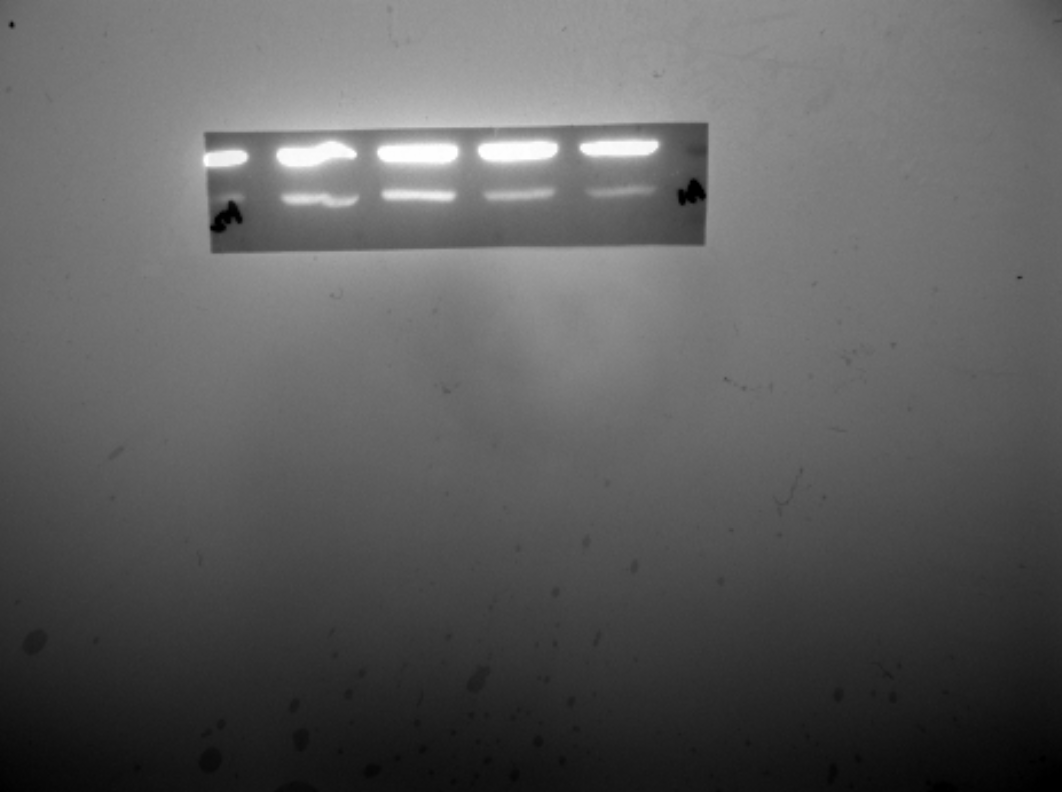

Supplement: Supplementary file 8 [file Data_Sheet_8.ZIP › FIG-5E/membrane with marker-ACTIN.tif]

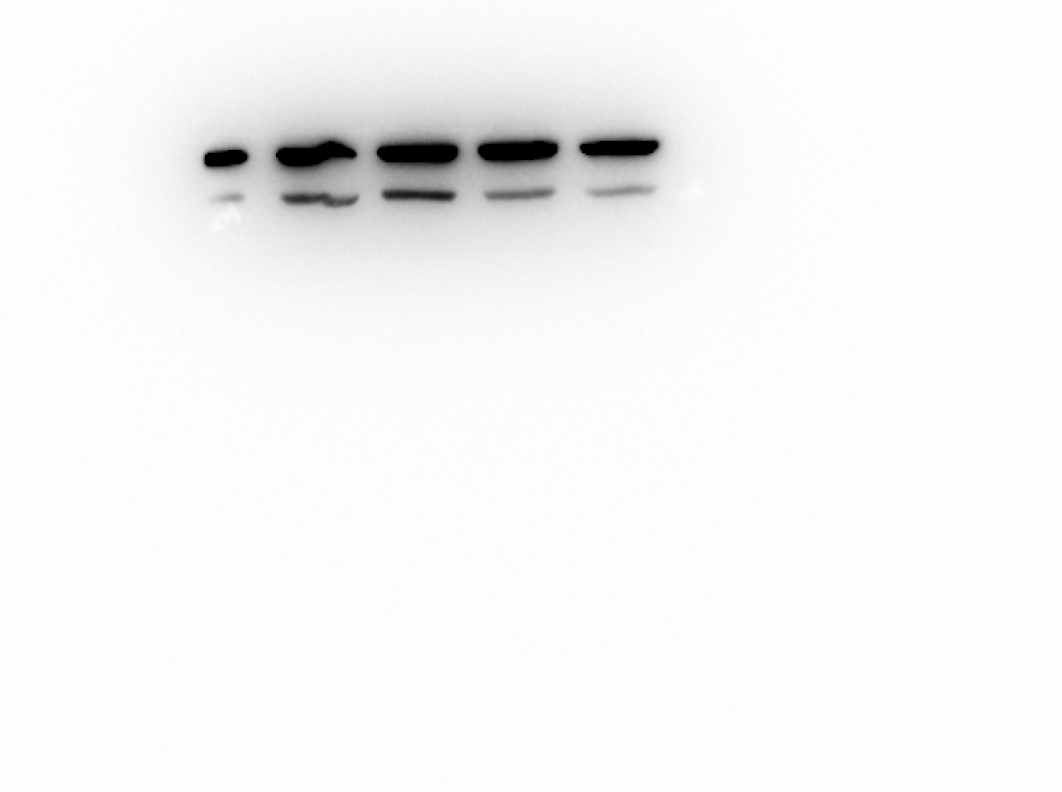

Supplement: Supplementary file 8 [file Data_Sheet_8.ZIP › FIG-5E/membrane-ACTIN.tif]

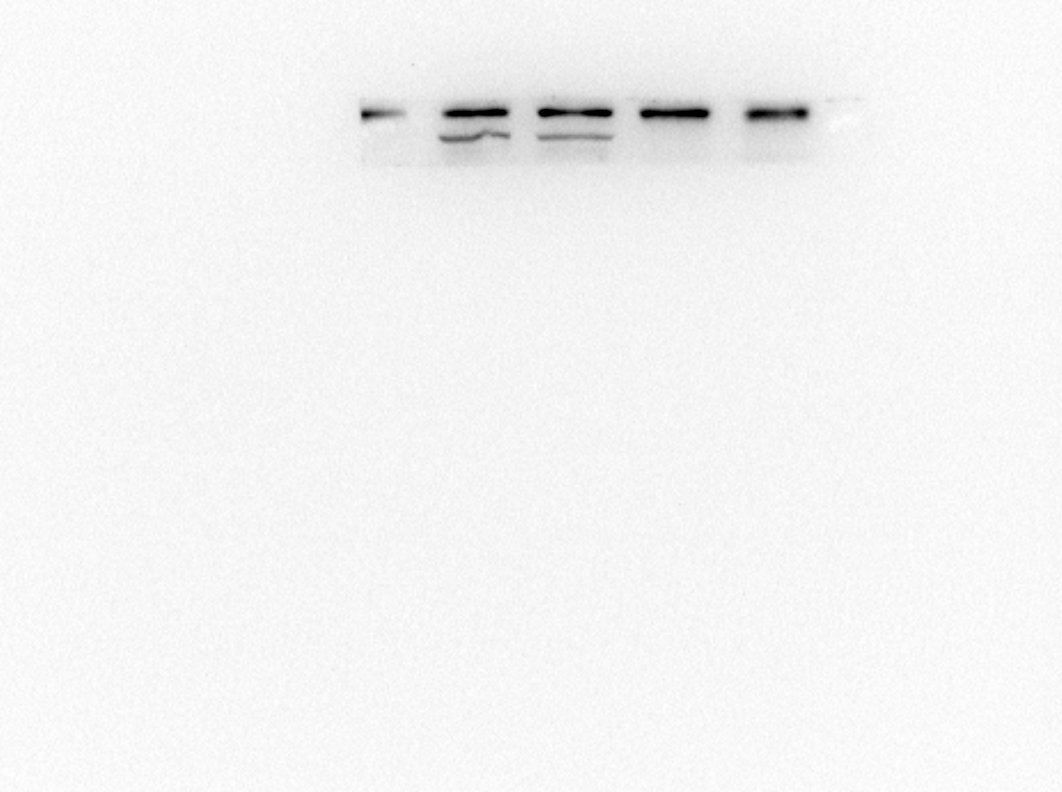

Supplement: Supplementary file 8 [file Data_Sheet_8.ZIP › FIG-5E/membrane-METAP2.tif]

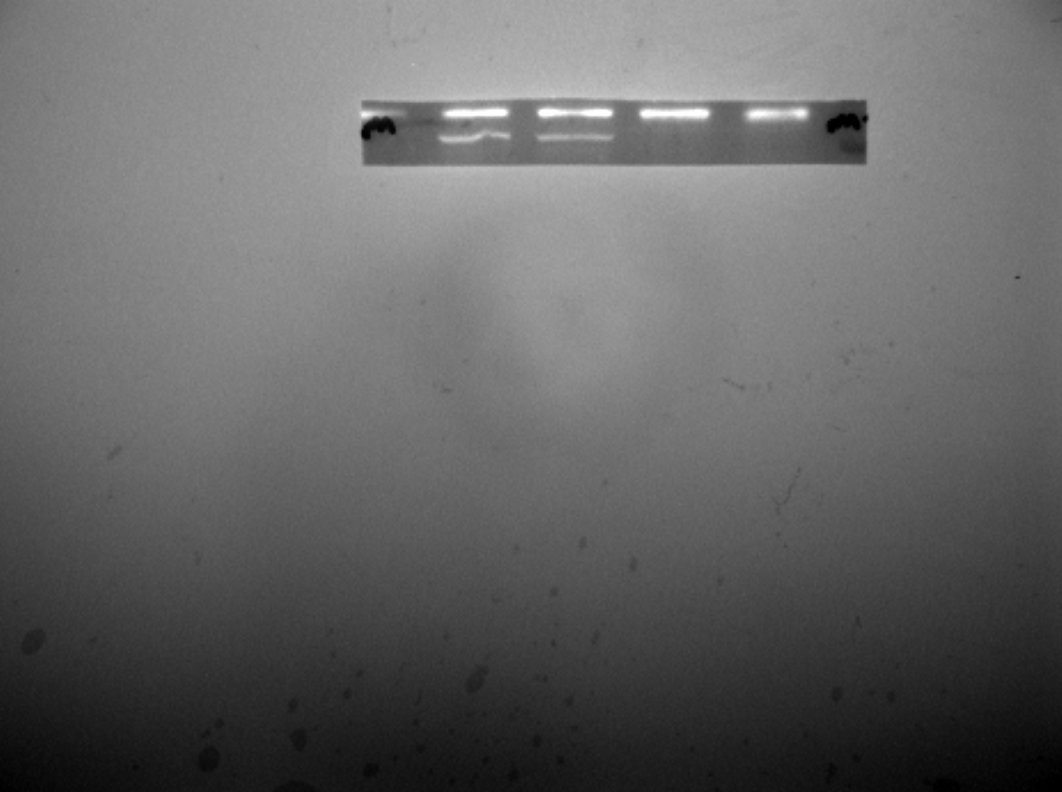

Supplement: Supplementary file 8 [file Data_Sheet_8.ZIP › FIG-5E/membrane-with marker METAP2.tif]

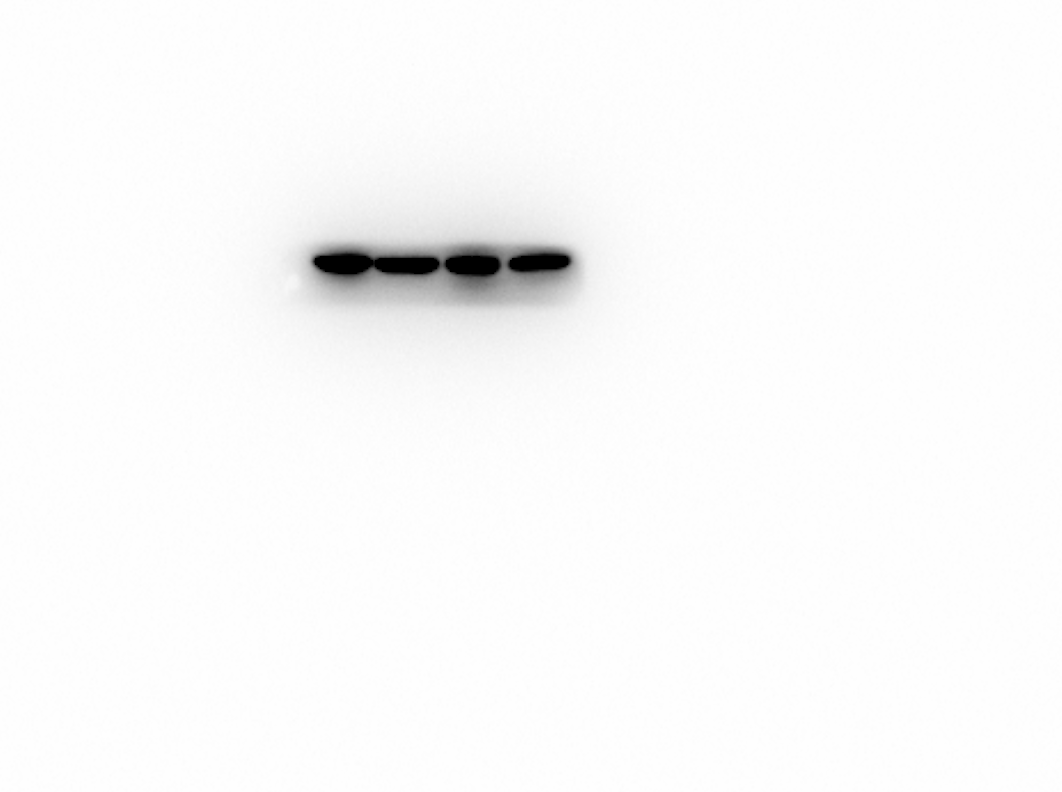

Supplement: Supplementary file 9 [file Data_Sheet_9.ZIP › FIG-5C/HCT116/membrane HCT116-28AF-ACTIN.tif]

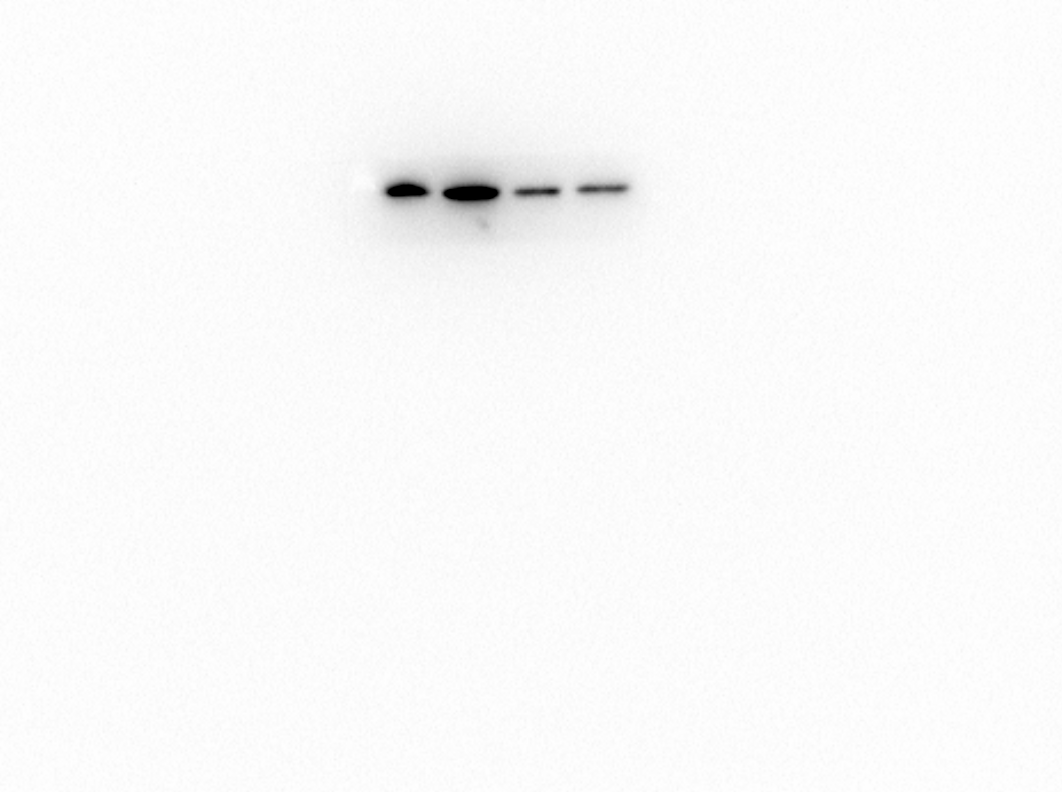

Supplement: Supplementary file 9 [file Data_Sheet_9.ZIP › FIG-5C/HCT116/membrane HCT116-28AF-METAP2.tif]

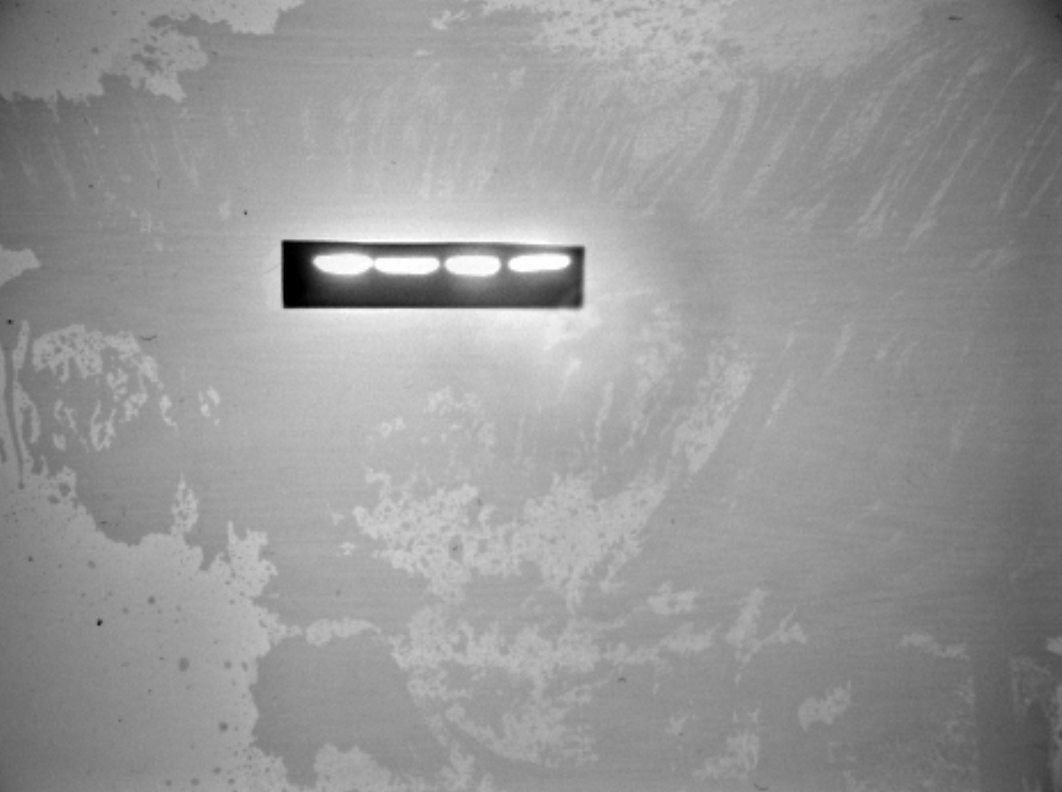

Supplement: Supplementary file 9 [file Data_Sheet_9.ZIP › FIG-5C/HCT116/membrane with marker HCT116-28AF-ACTIN.tif]

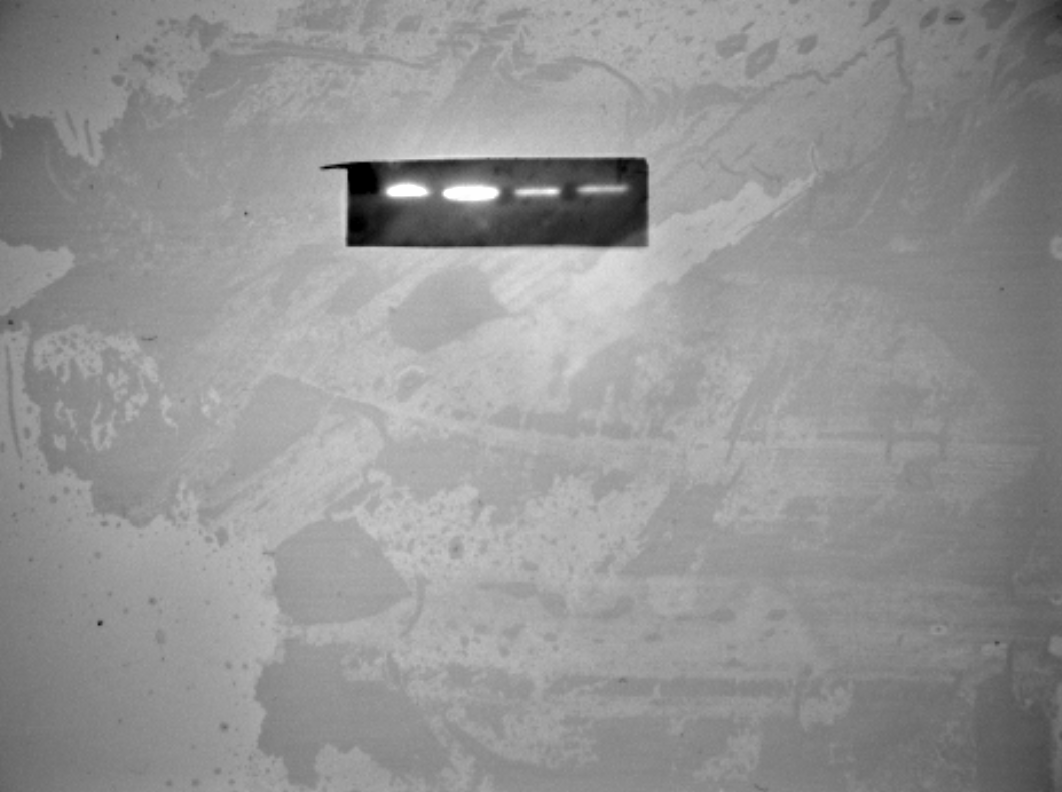

Supplement: Supplementary file 9 [file Data_Sheet_9.ZIP › FIG-5C/HCT116/membrane with marker HCT116-28AF-METAP2.tif]

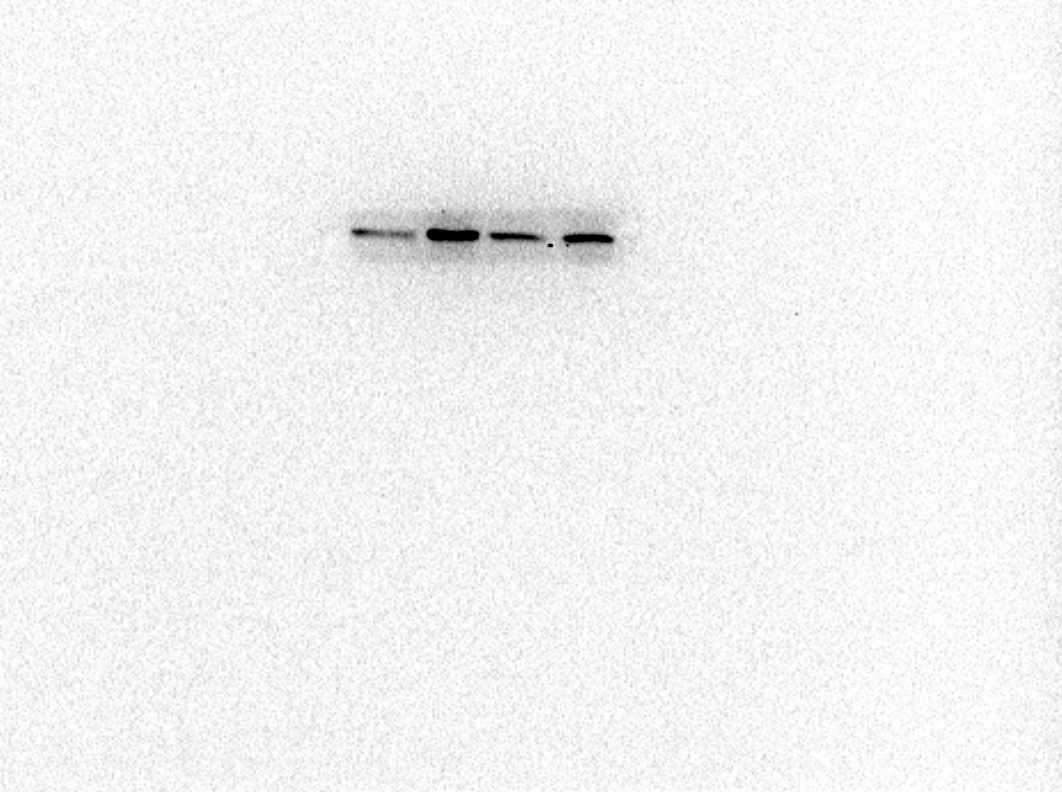

Supplement: Supplementary file 9 [file Data_Sheet_9.ZIP › FIG-5C/SW1116/membrane SW1116-MAP2.tif]

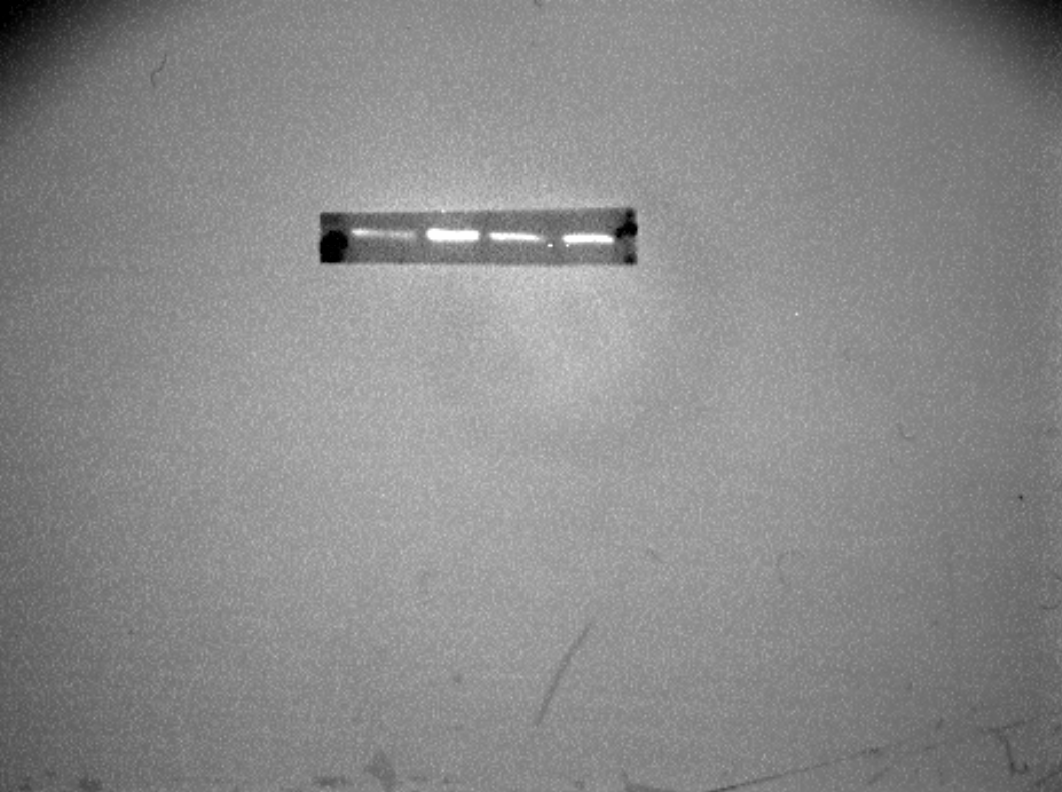

Supplement: Supplementary file 9 [file Data_Sheet_9.ZIP › FIG-5C/SW1116/membrane with marker SW1116-MAP2.tif]

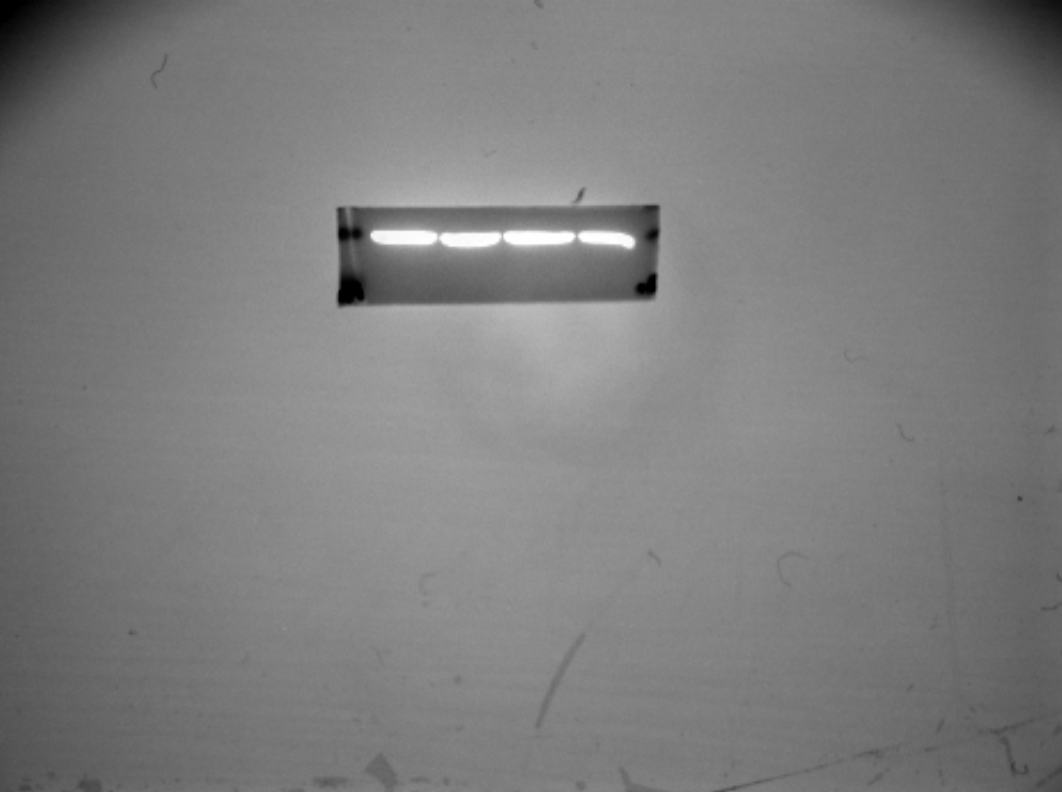

Supplement: Supplementary file 9 [file Data_Sheet_9.ZIP › FIG-5C/SW1116/membrane with marker-SW1116-ACTIN.tif]

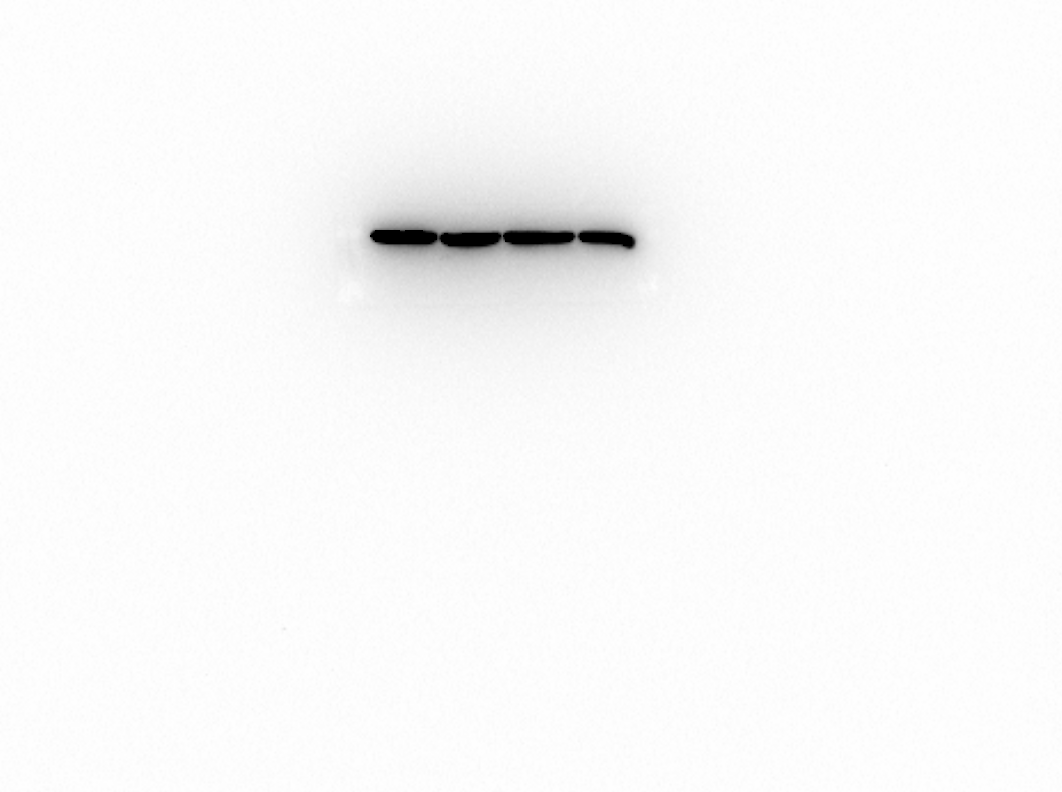

Supplement: Supplementary file 9 [file Data_Sheet_9.ZIP › FIG-5C/SW1116/membrane-SW1116-ACTIN.tif]

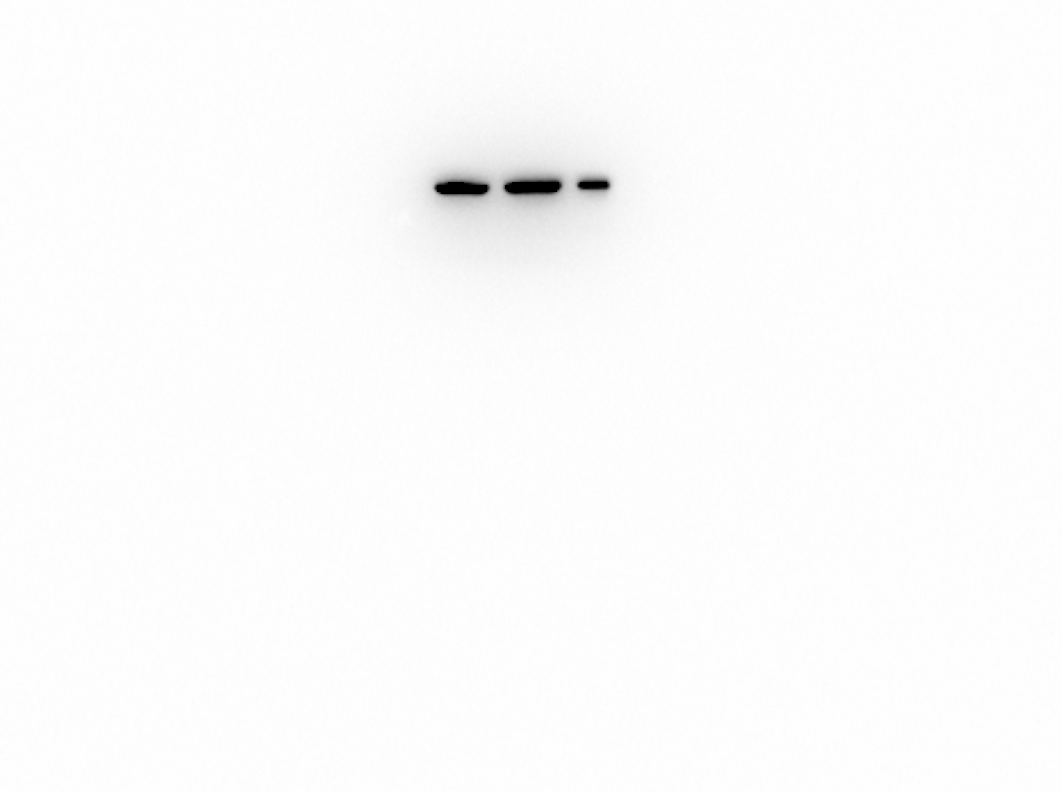

Supplement: Supplementary file 10 [file Data_Sheet_10.ZIP › FIG-5G/HCT116-DFO/membrane -HCT116-DFO-ACTIN.tif]

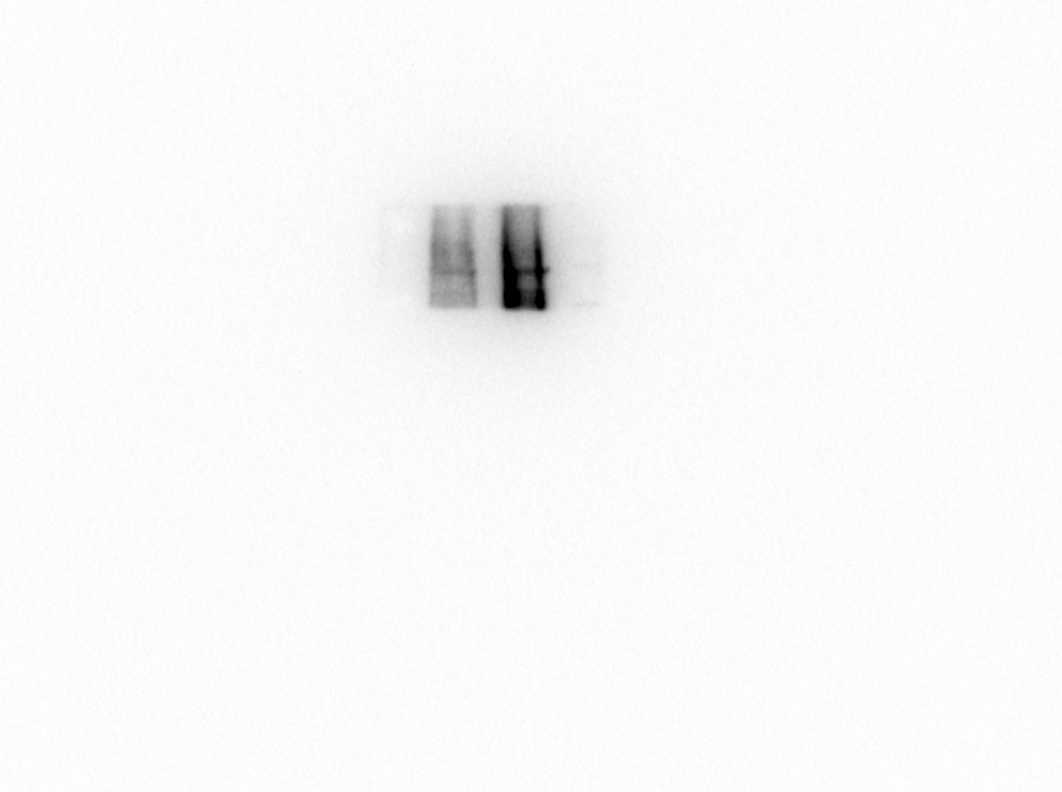

Supplement: Supplementary file 10 [file Data_Sheet_10.ZIP › FIG-5G/HCT116-DFO/membrane HCT116-DFO-HIFa.tif]

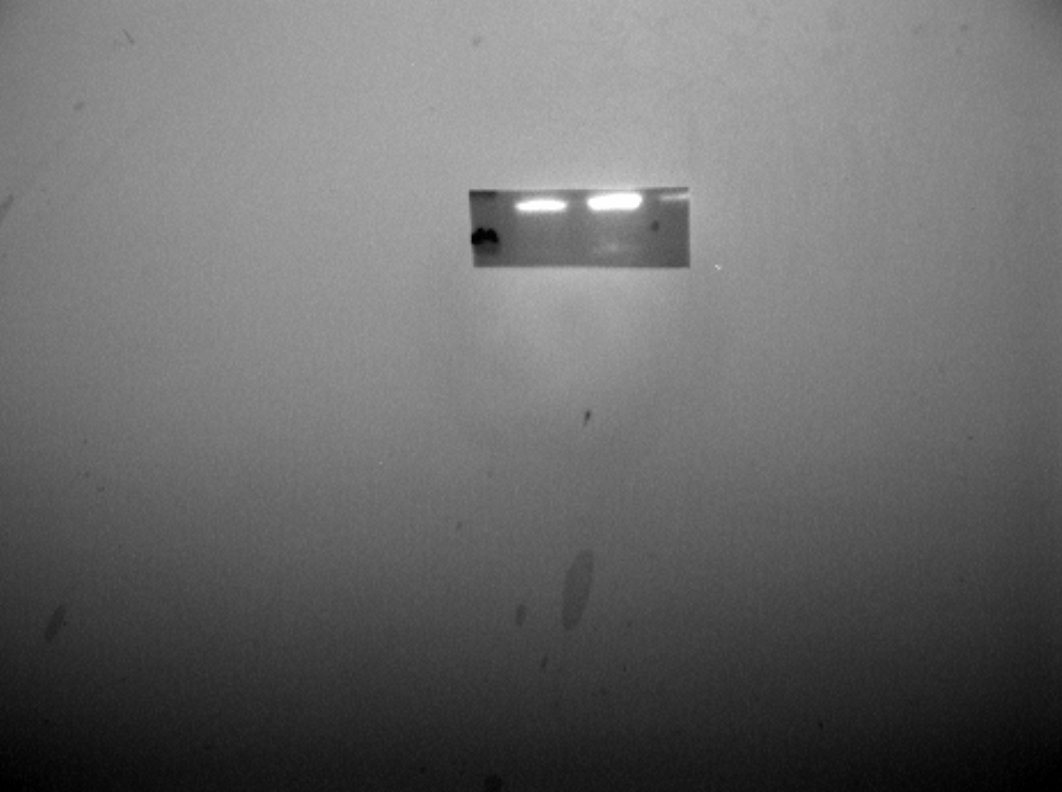

Supplement: Supplementary file 10 [file Data_Sheet_10.ZIP › FIG-5G/HCT116-DFO/membrane with maker HCT116-DFO-METAP2.tif]

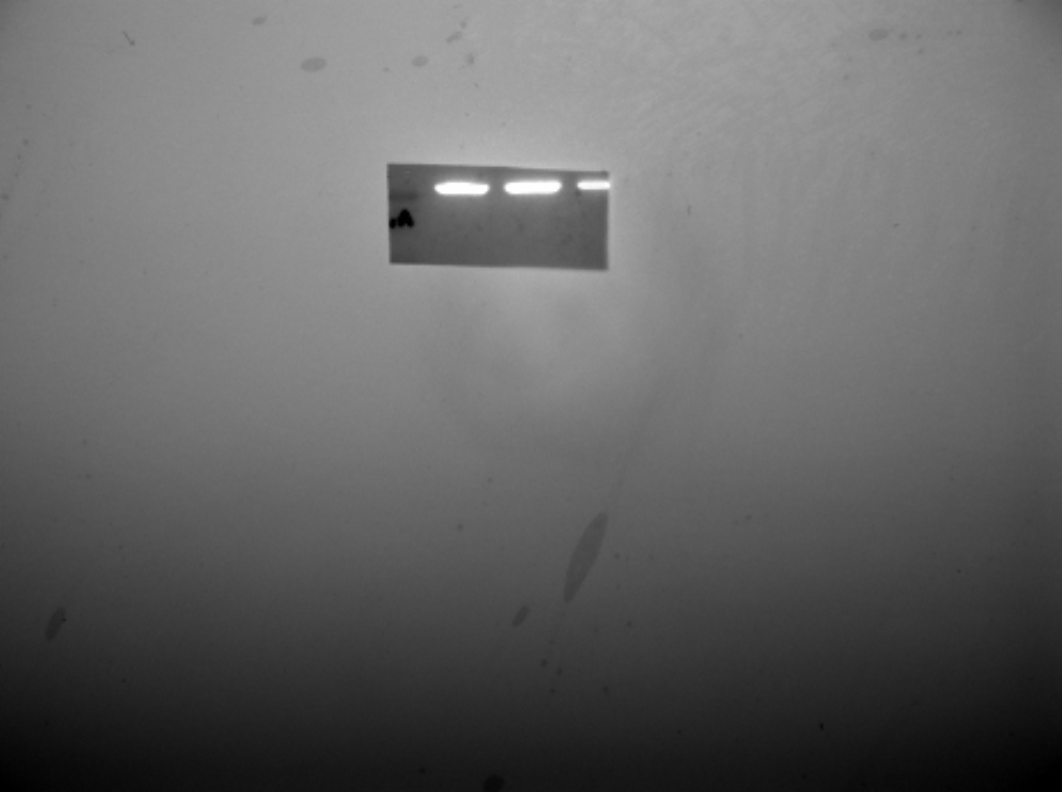

Supplement: Supplementary file 10 [file Data_Sheet_10.ZIP › FIG-5G/HCT116-DFO/membrane with marker-HCT116-DFO-ACTIN.tif]

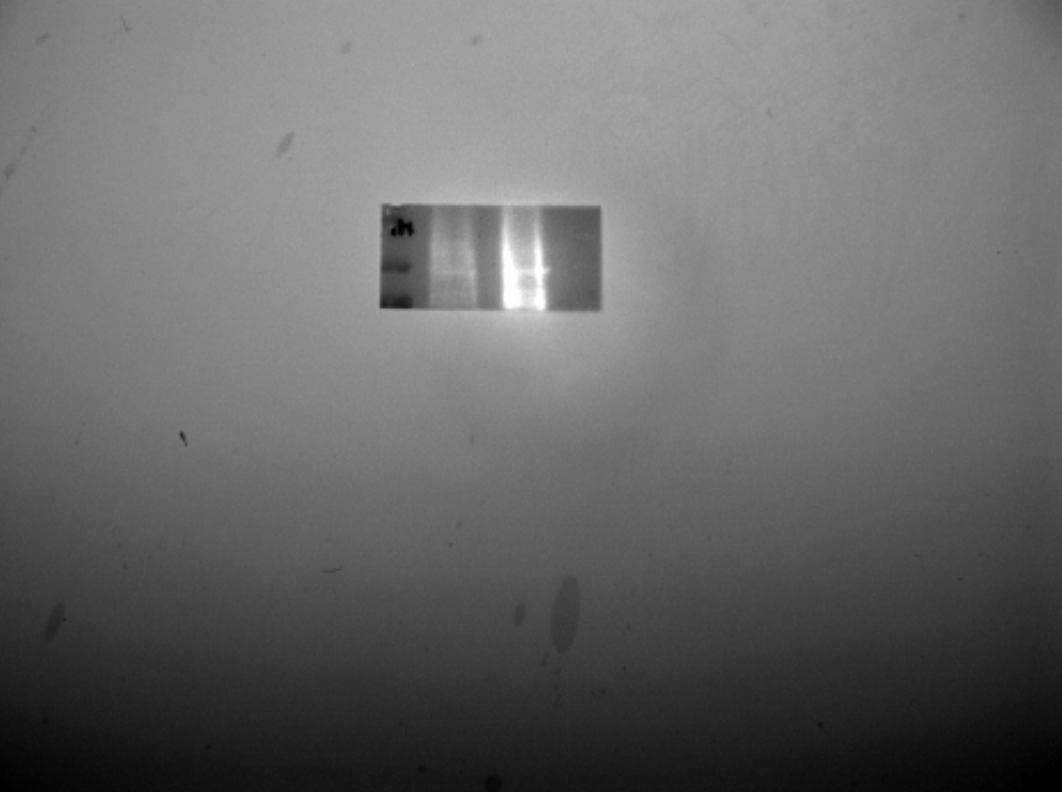

Supplement: Supplementary file 10 [file Data_Sheet_10.ZIP › FIG-5G/HCT116-DFO/membrane with marker-HCT116-DFO-HIFA.tif]

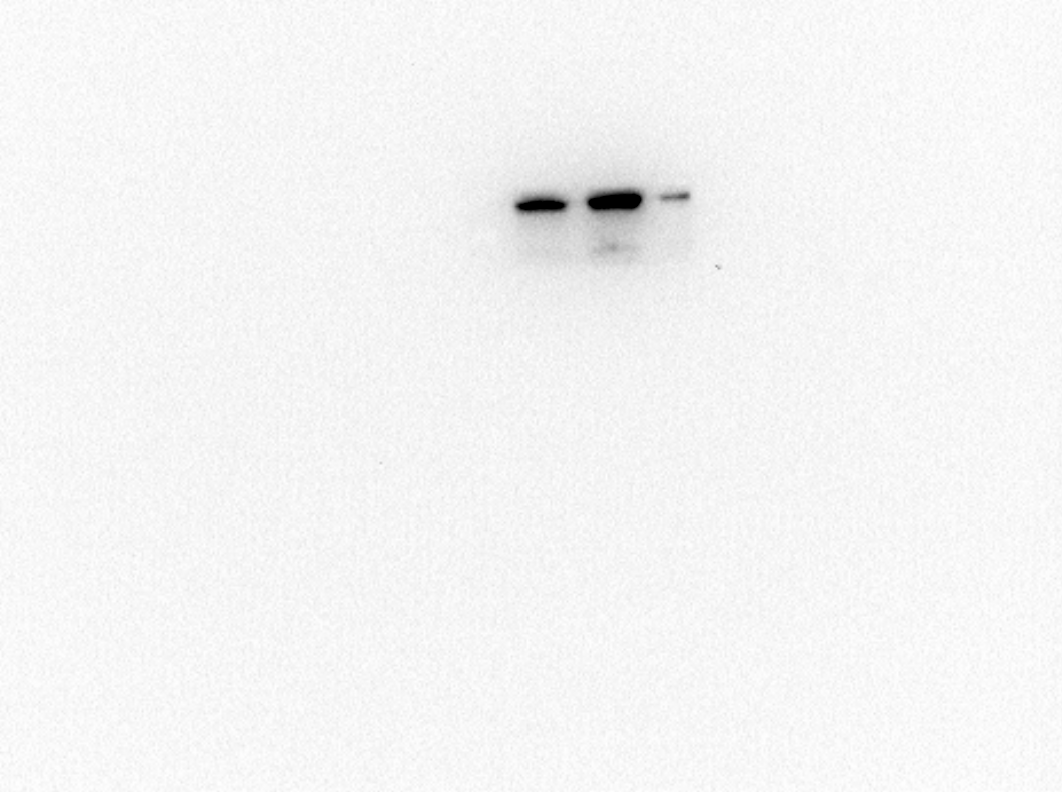

Supplement: Supplementary file 10 [file Data_Sheet_10.ZIP › FIG-5G/HCT116-DFO/membrene-HCT116-DFO-METAP2.tif]

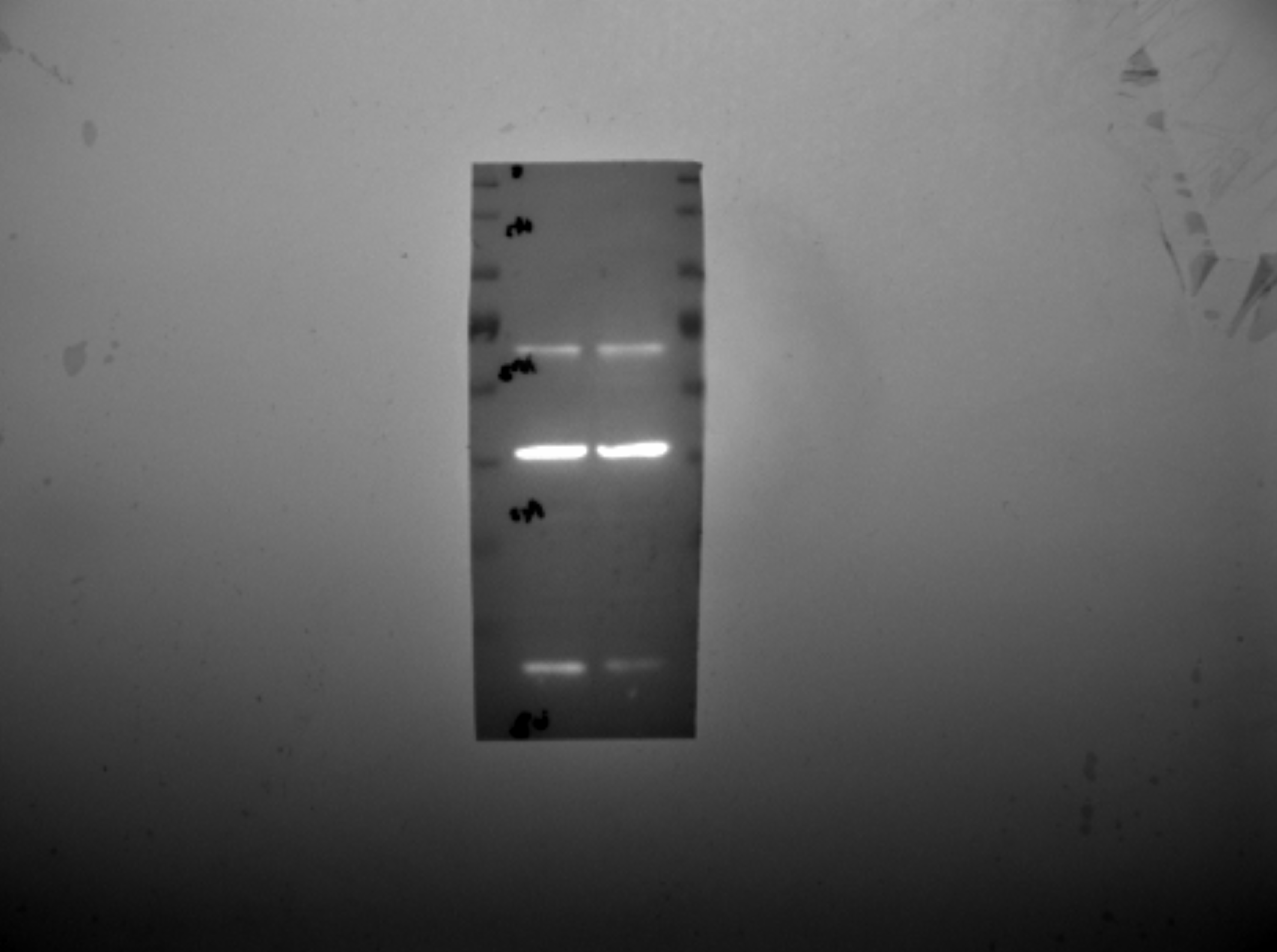

Supplement: Supplementary file 10 [file Data_Sheet_10.ZIP › FIG-5G/SW1116-DFO/membrane with marker-ACTIN.tif]

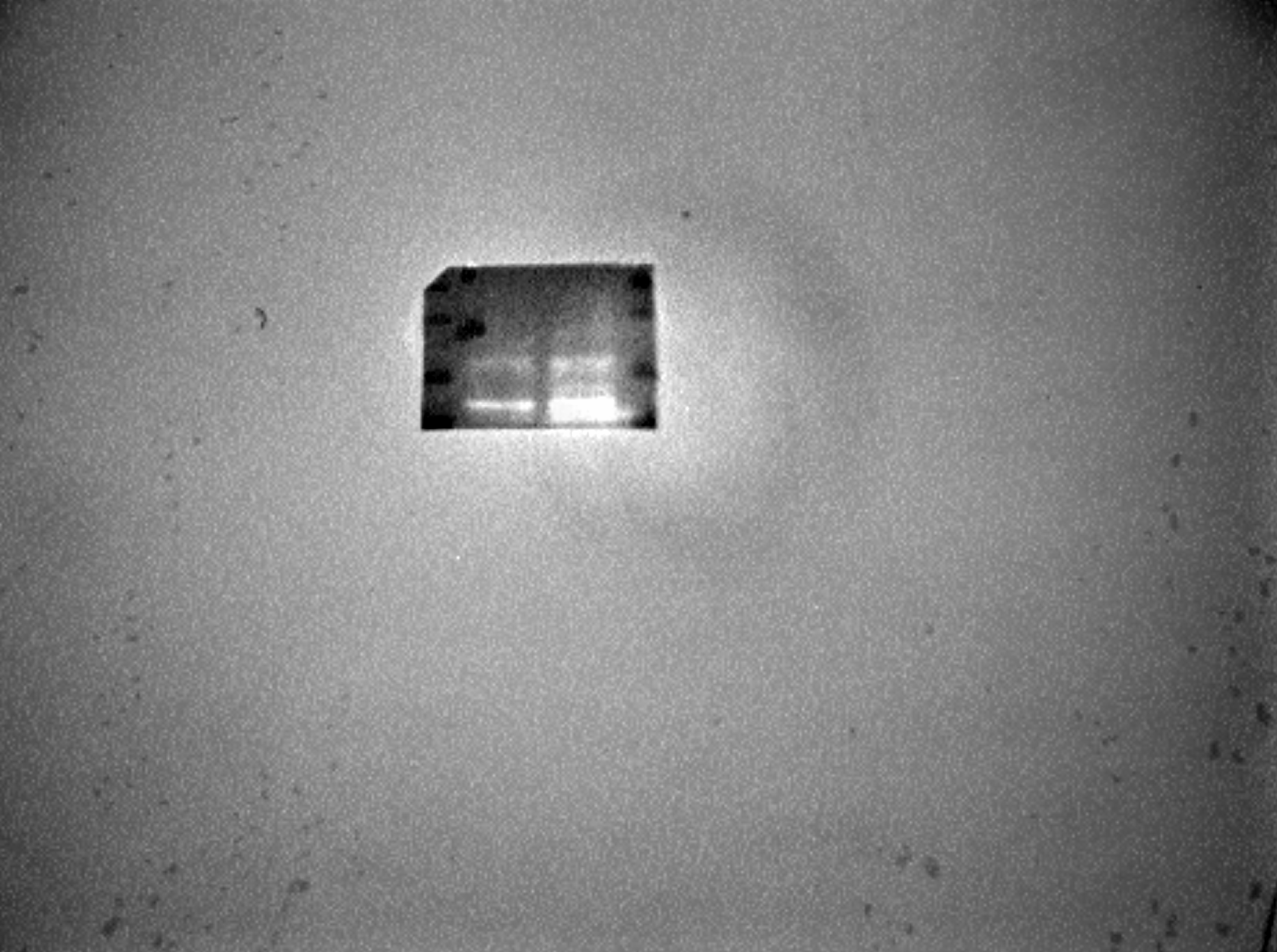

Supplement: Supplementary file 10 [file Data_Sheet_10.ZIP › FIG-5G/SW1116-DFO/membrane with marker-HIFA.tif]

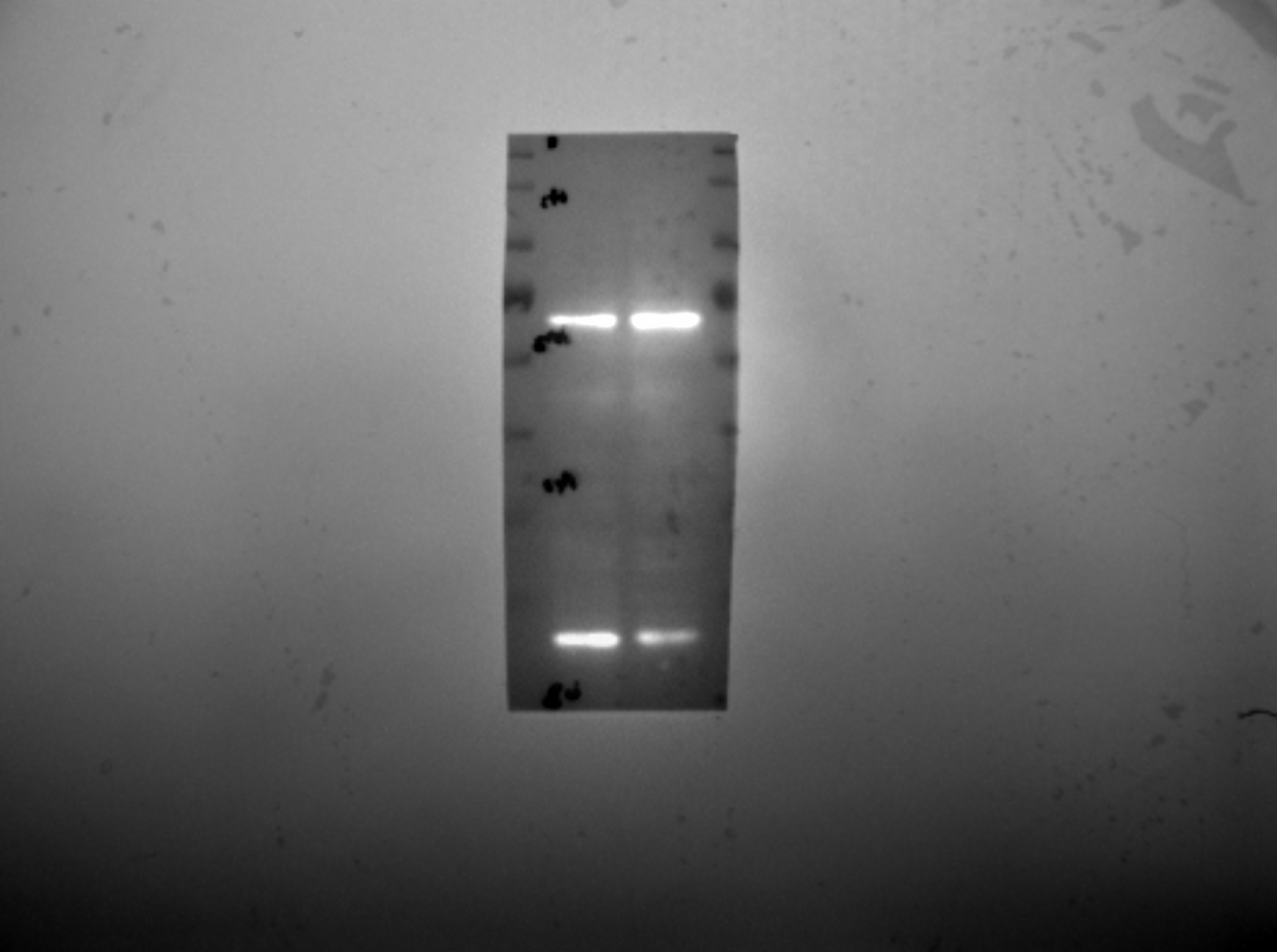

Supplement: Supplementary file 10 [file Data_Sheet_10.ZIP › FIG-5G/SW1116-DFO/membrane with marker-METAP2+LIN28A.tif]
