# Supplementary figures and images for: Hypoxia-Induced LIN28A mRNA Promotes the Metastasis of Colon Cancer in a Protein-Coding-Independent Manner (part 2 of 2)
Source: Front Cell Dev Biol. 2021 Feb 16;9:642930. doi: 10.3389/fcell.2021.642930 (PMC7921329; doi:10.3389/fcell.2021.642930)

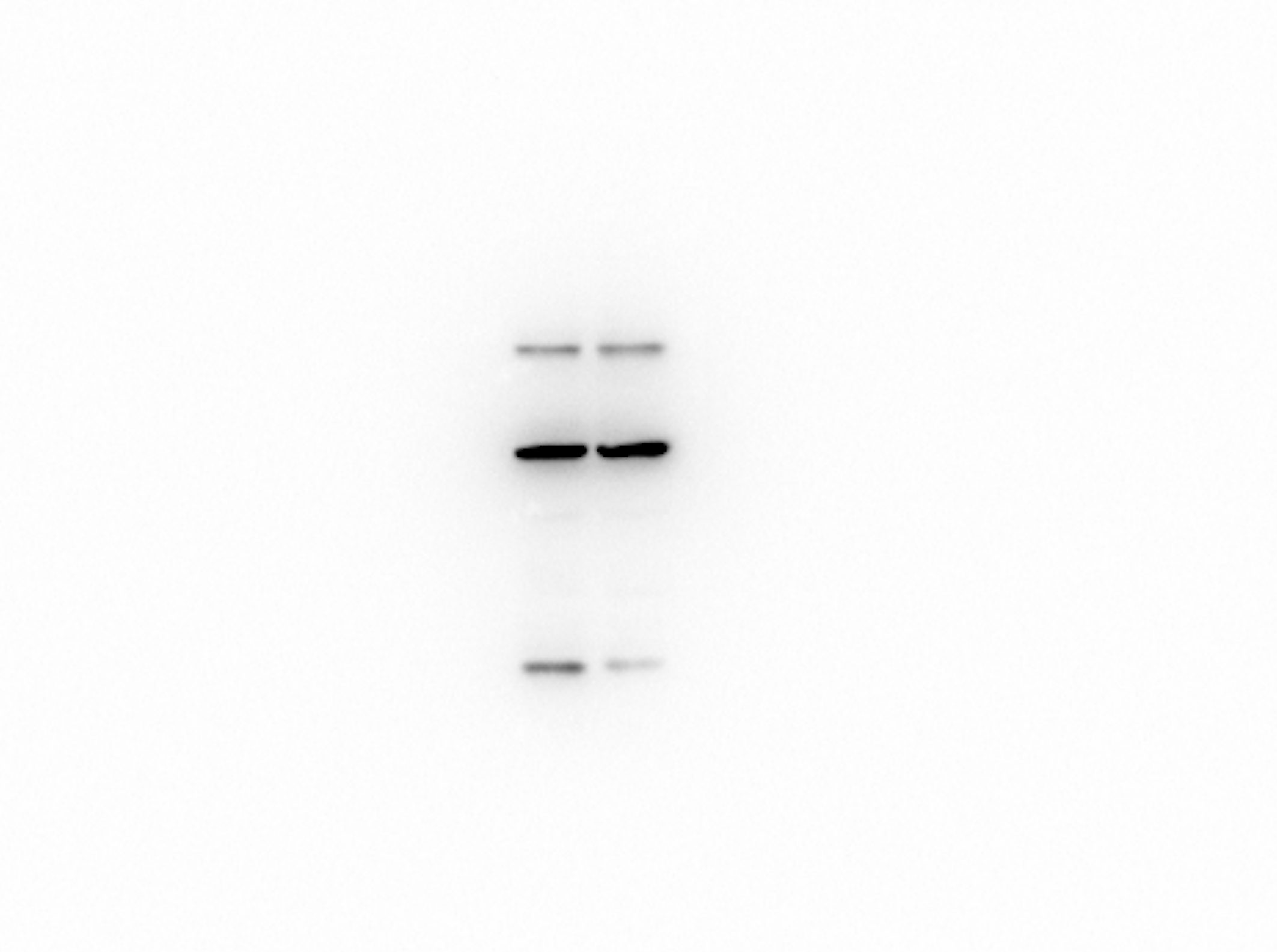

Supplement: Supplementary file 10 [file Data_Sheet_10.ZIP › FIG-5G/SW1116-DFO/membrane-ACTIN.tif]

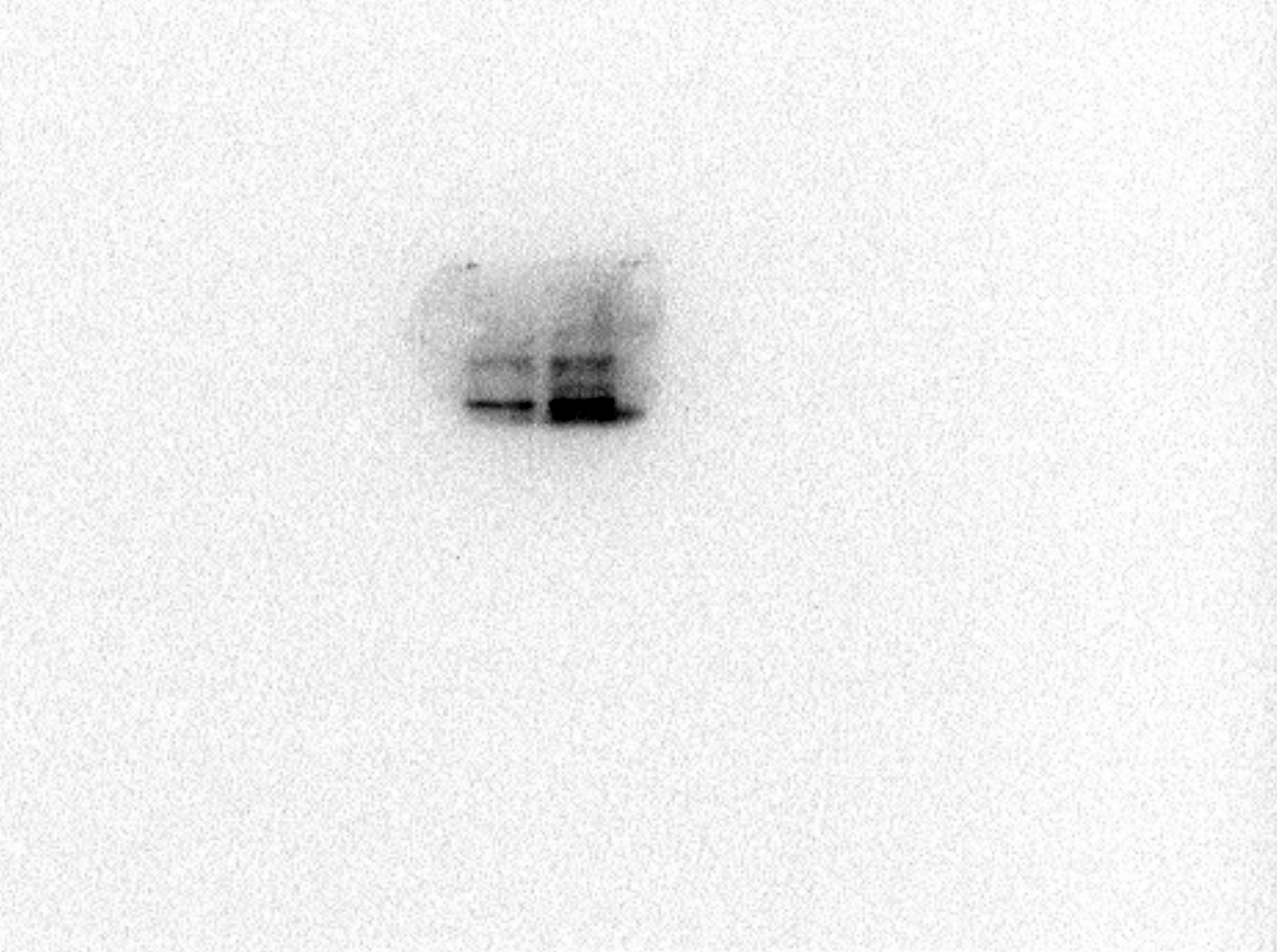

Supplement: Supplementary file 10 [file Data_Sheet_10.ZIP › FIG-5G/SW1116-DFO/membrane-HIFA.tif]

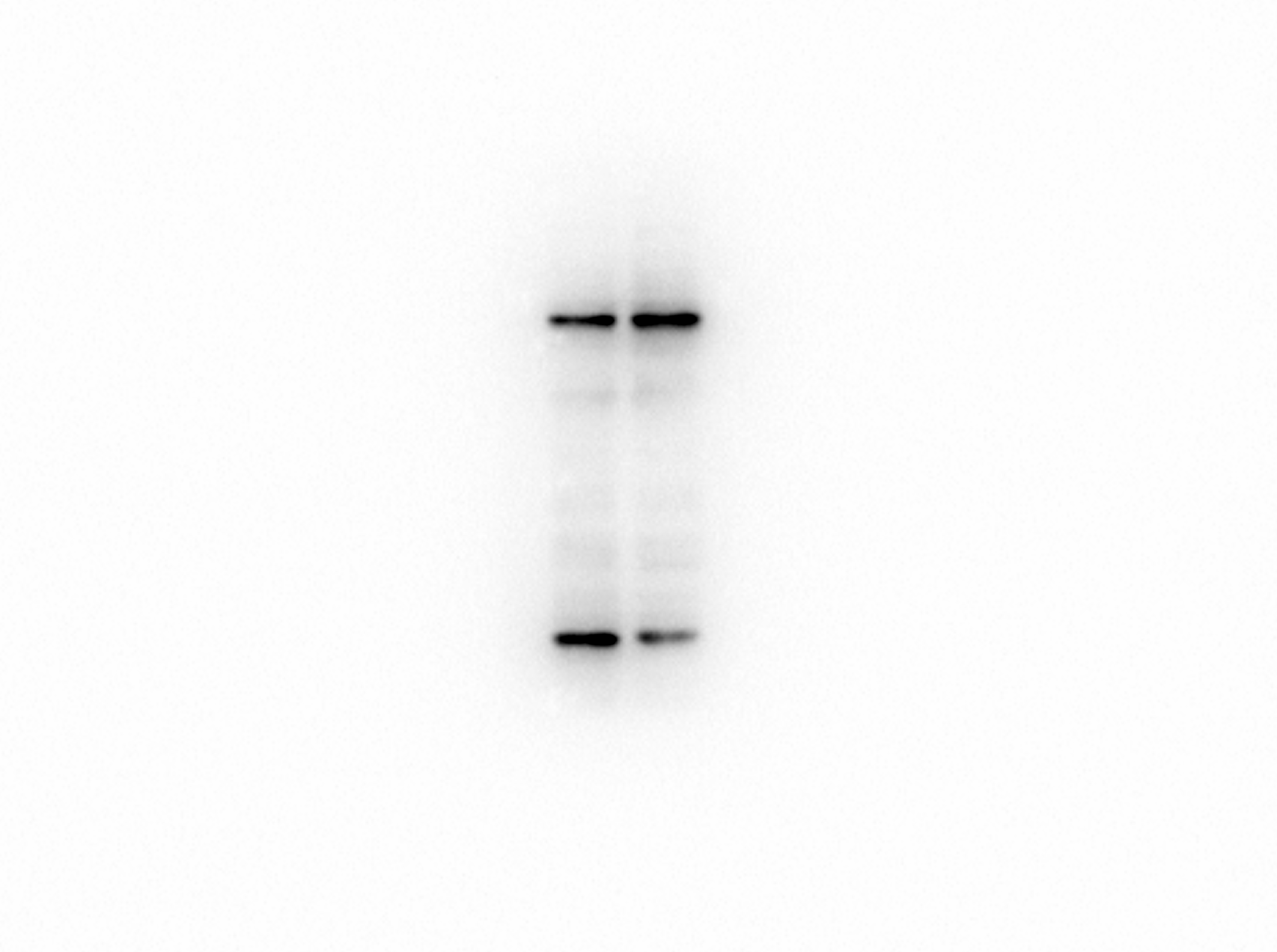

Supplement: Supplementary file 10 [file Data_Sheet_10.ZIP › FIG-5G/SW1116-DFO/membrane-METAP2+LIN28A.tif]

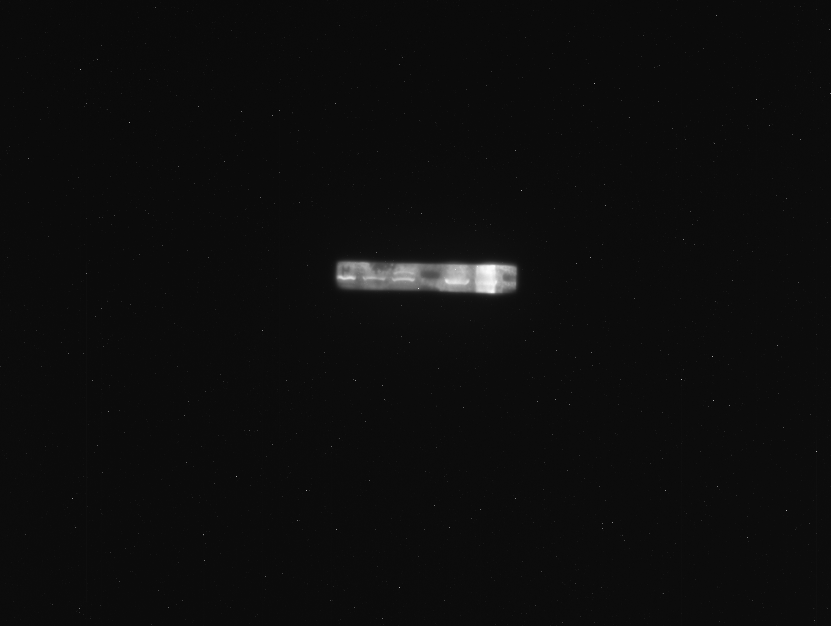

Supplement: Supplementary file 10 [file Data_Sheet_10.ZIP › FIG-5G/SW1116-HCT116-1%O2/membrane SW1116-HCT116-1%O2-hifa/2019-02-25_10-26-45 S-H-hifa_1_16bit.png]

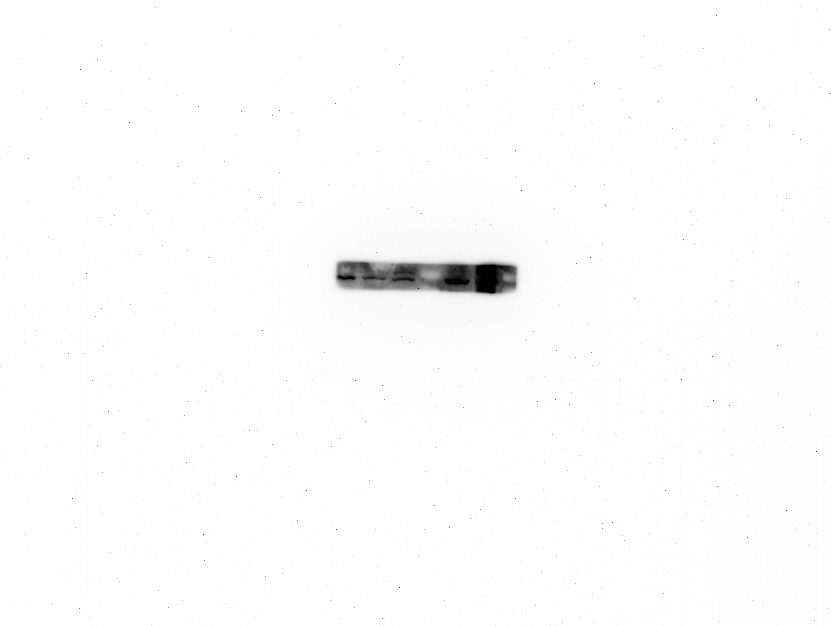

Supplement: Supplementary file 10 [file Data_Sheet_10.ZIP › FIG-5G/SW1116-HCT116-1%O2/membrane SW1116-HCT116-1%O2-hifa/2019-02-25_10-26-45 S-H-hifa_8bit.png]

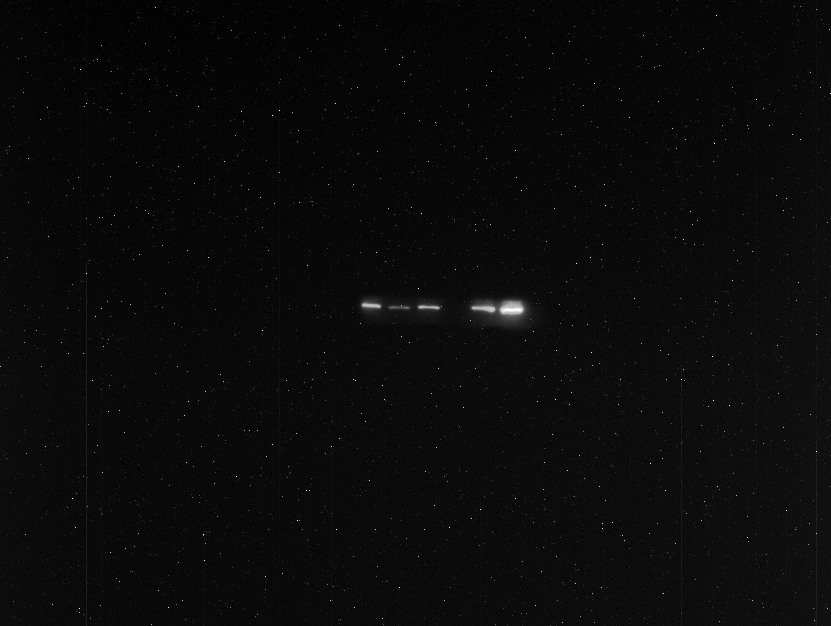

Supplement: Supplementary file 10 [file Data_Sheet_10.ZIP › FIG-5G/SW1116-HCT116-1%O2/membrane SW1116-HCT116-1%O2-METAP2/2019-02-25_10-29-11 S-H-map2_1_16bit.png]

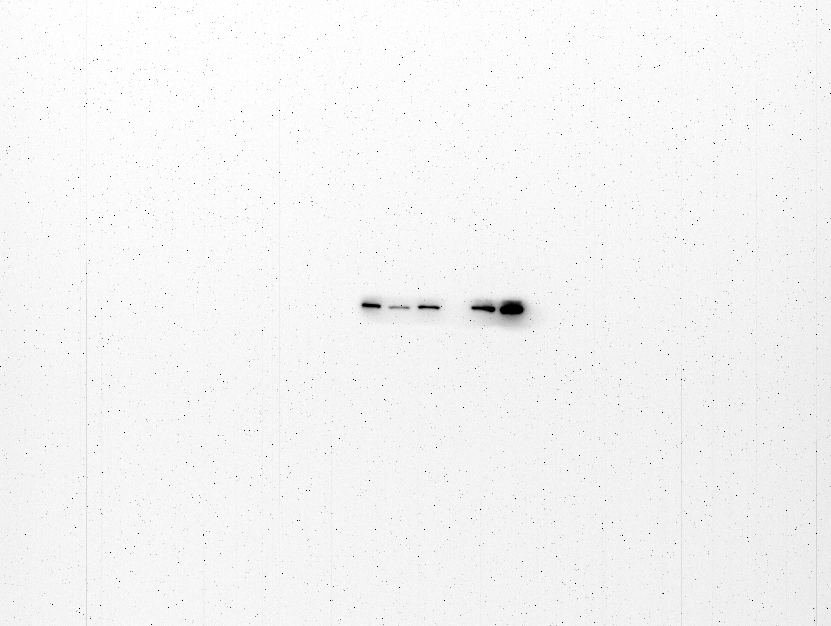

Supplement: Supplementary file 10 [file Data_Sheet_10.ZIP › FIG-5G/SW1116-HCT116-1%O2/membrane SW1116-HCT116-1%O2-METAP2/2019-02-25_10-29-11 S-H-map2_8bit.png]

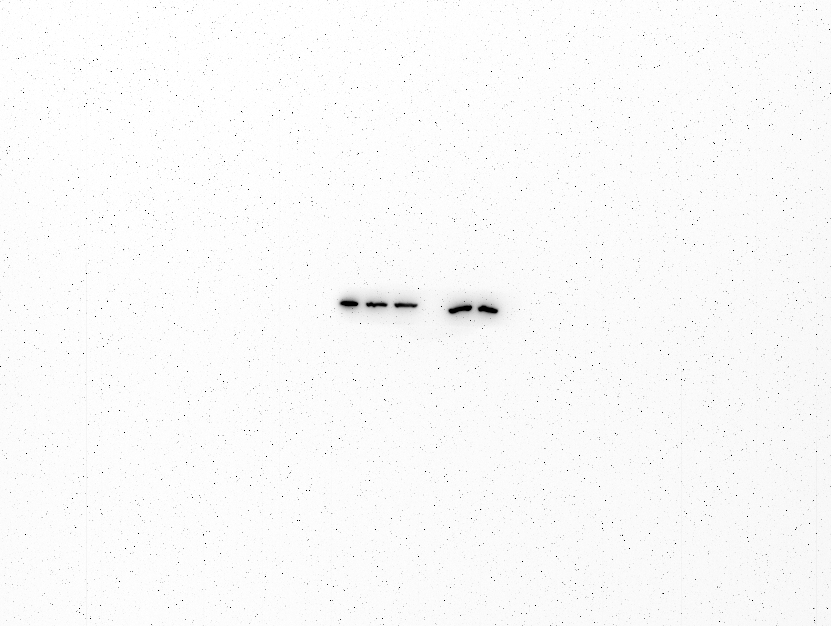

Supplement: Supplementary file 10 [file Data_Sheet_10.ZIP › FIG-5G/SW1116-HCT116-1%O2/membrane-SW1116-HCT116-1%O2-actin.png]

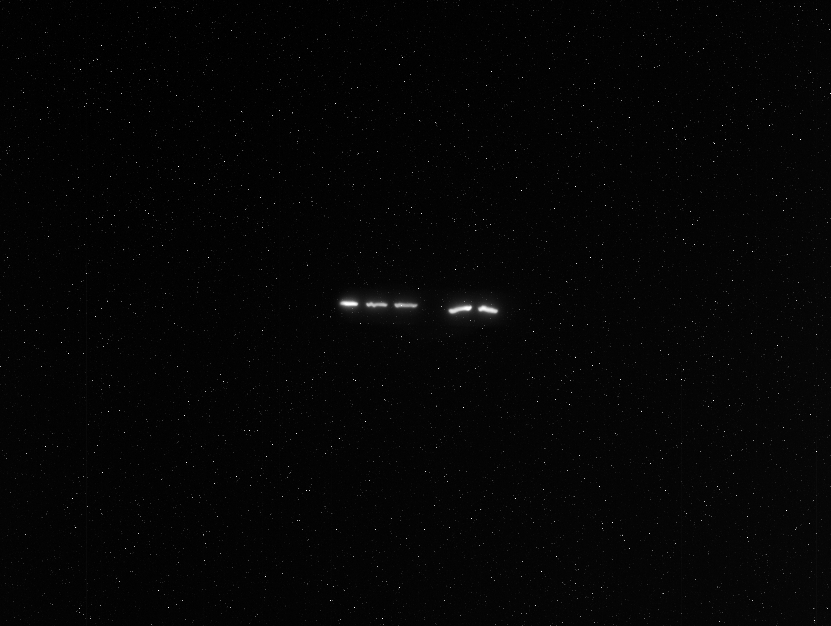

Supplement: Supplementary file 10 [file Data_Sheet_10.ZIP › FIG-5G/SW1116-HCT116-1%O2/membrane-SW1116-HCT116-1%O2-actin/2019-02-25_10-23-14 S-H-actin_1_16bit.png]

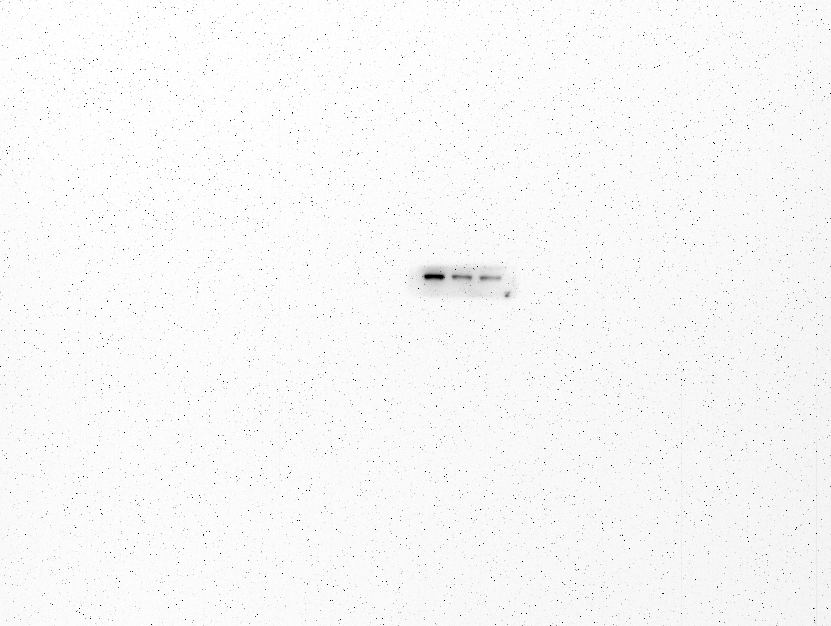

Supplement: Supplementary file 11 [file Data_Sheet_11.ZIP › FIG-5H/HCT116-siMETAP2/membrane HCT116-siMETAP2-METAP2.png]

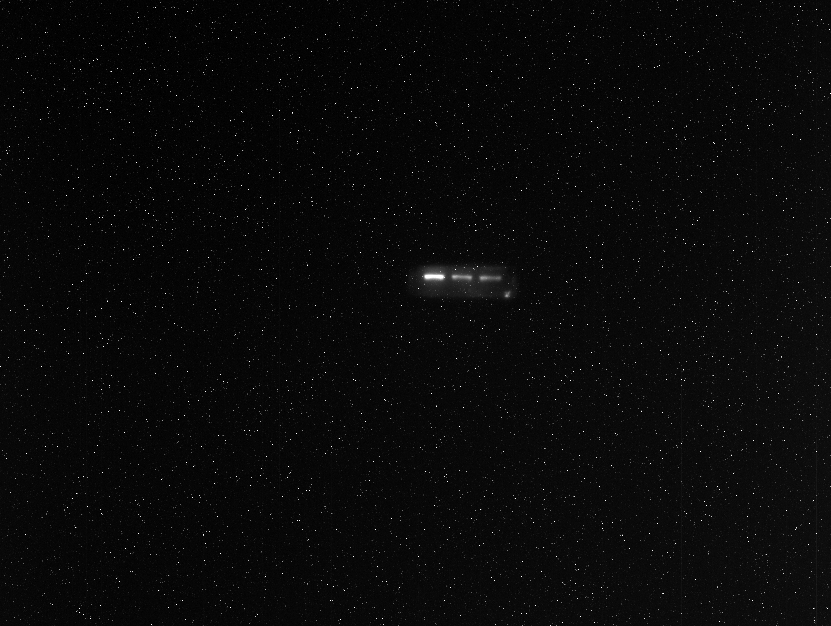

Supplement: Supplementary file 11 [file Data_Sheet_11.ZIP › FIG-5H/HCT116-siMETAP2/membrane HCT116-siMETAP2-METAP2/2019-11-26_13-35-00 H-siMAP2-map2_1_16bit.png]

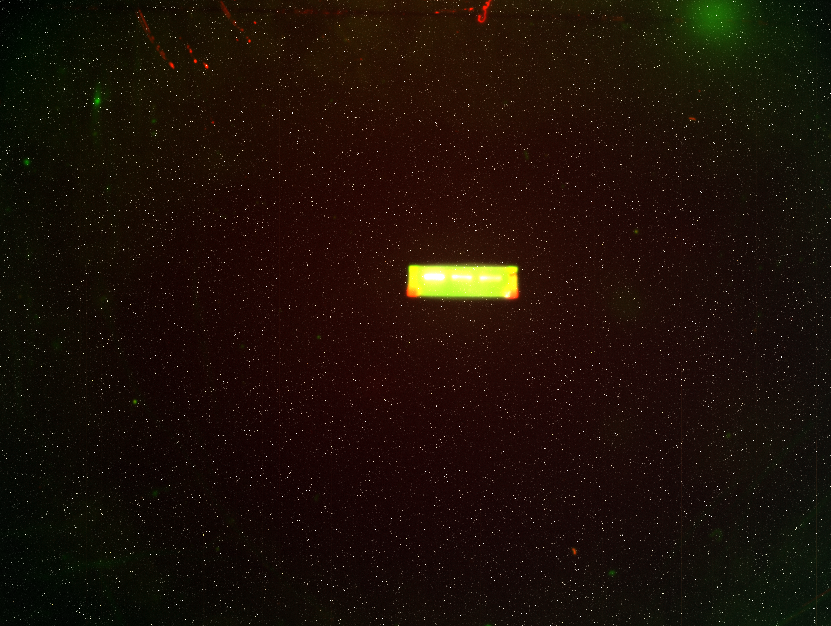

Supplement: Supplementary file 11 [file Data_Sheet_11.ZIP › FIG-5H/HCT116-siMETAP2/membrane with marker HCT116-siMETAP2-METAP2.png]

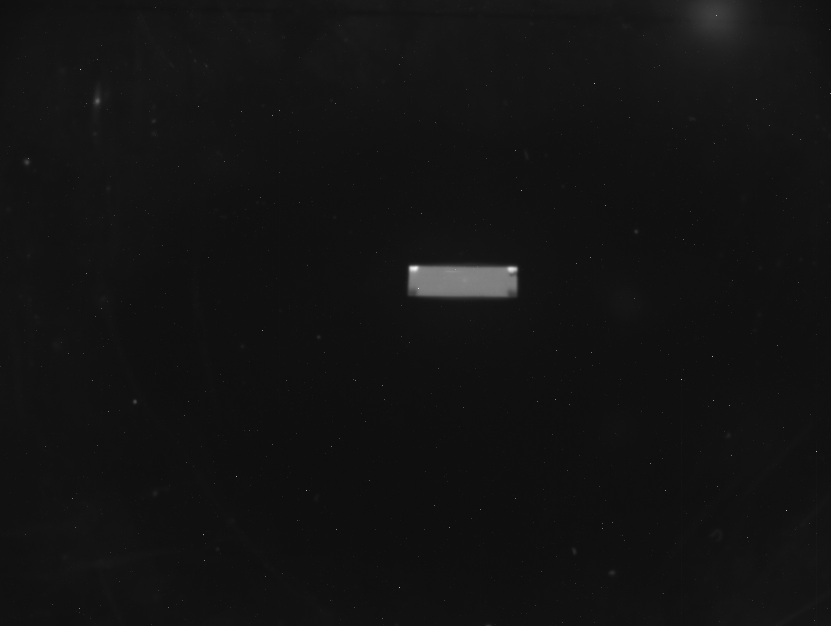

Supplement: Supplementary file 11 [file Data_Sheet_11.ZIP › FIG-5H/HCT116-siMETAP2/membrane with marker HCT116-siMETAP2-METAP2/2019-11-26_13-37-06_3_16bit.png]

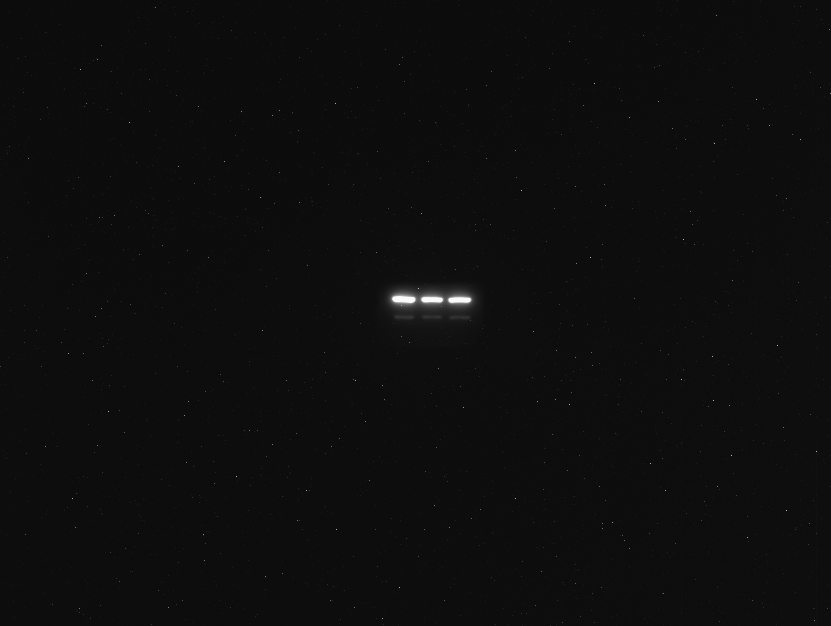

Supplement: Supplementary file 11 [file Data_Sheet_11.ZIP › FIG-5H/HCT116-siMETAP2/membrane-HCT116-siMETAP2-ACTIN/2019-11-26_13-30-33 H-siMAP2-actin_1_16bit.png]

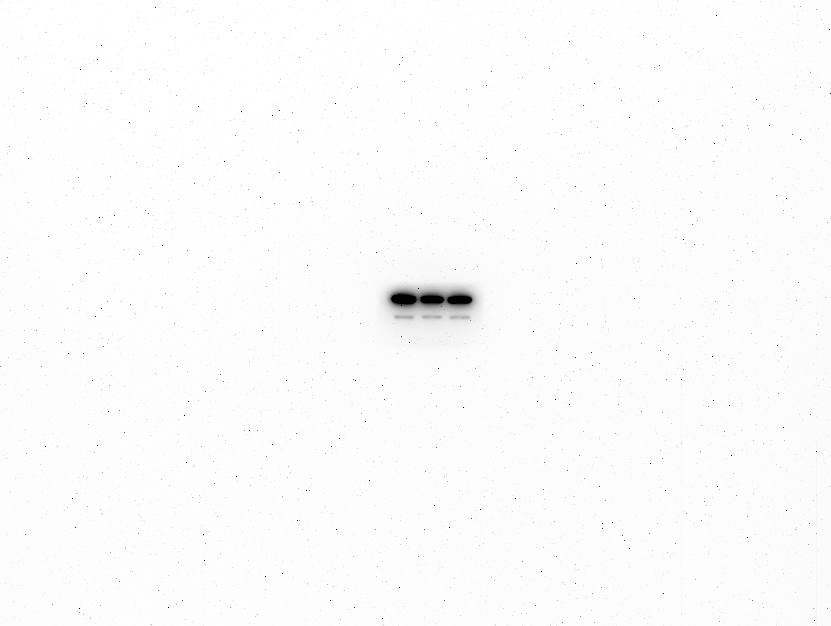

Supplement: Supplementary file 11 [file Data_Sheet_11.ZIP › FIG-5H/HCT116-siMETAP2/membrane-HCT116-siMETAP2-ACTIN/2019-11-26_13-30-33 H-siMAP2-actin_8bit.png]

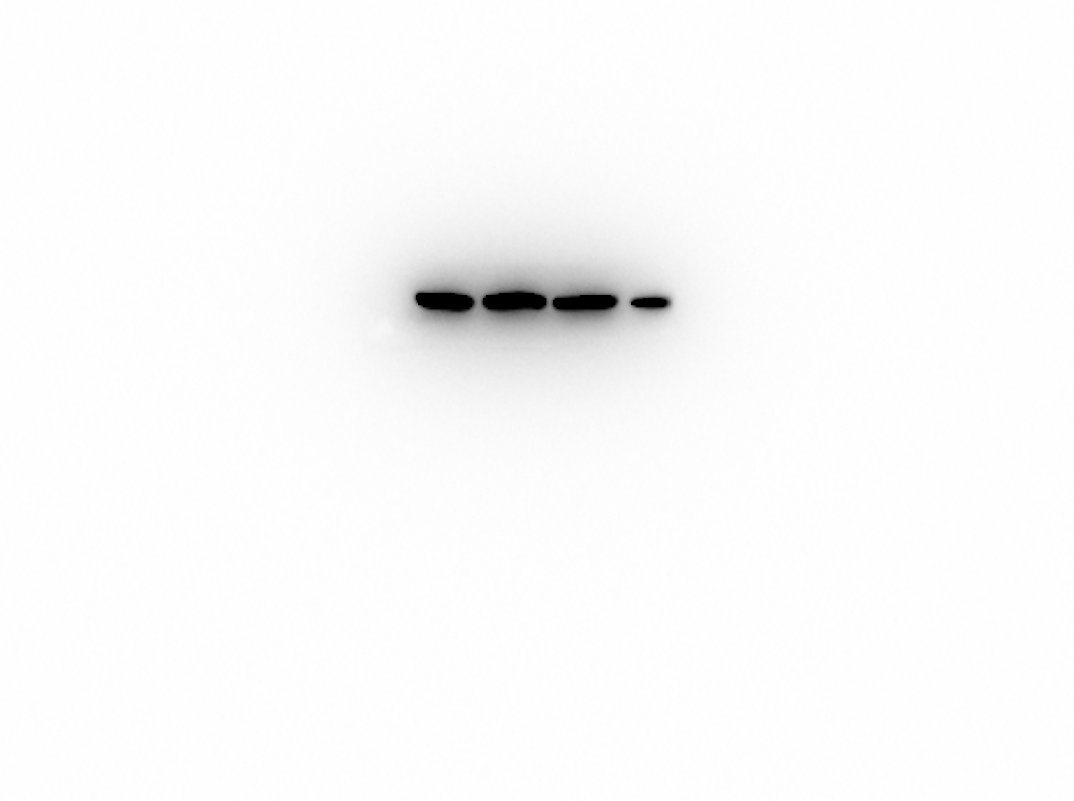

Supplement: Supplementary file 11 [file Data_Sheet_11.ZIP › FIG-5H/SW1116-siMETAP2/membrane SW1116-siMETAP2-ACTIN.tif]

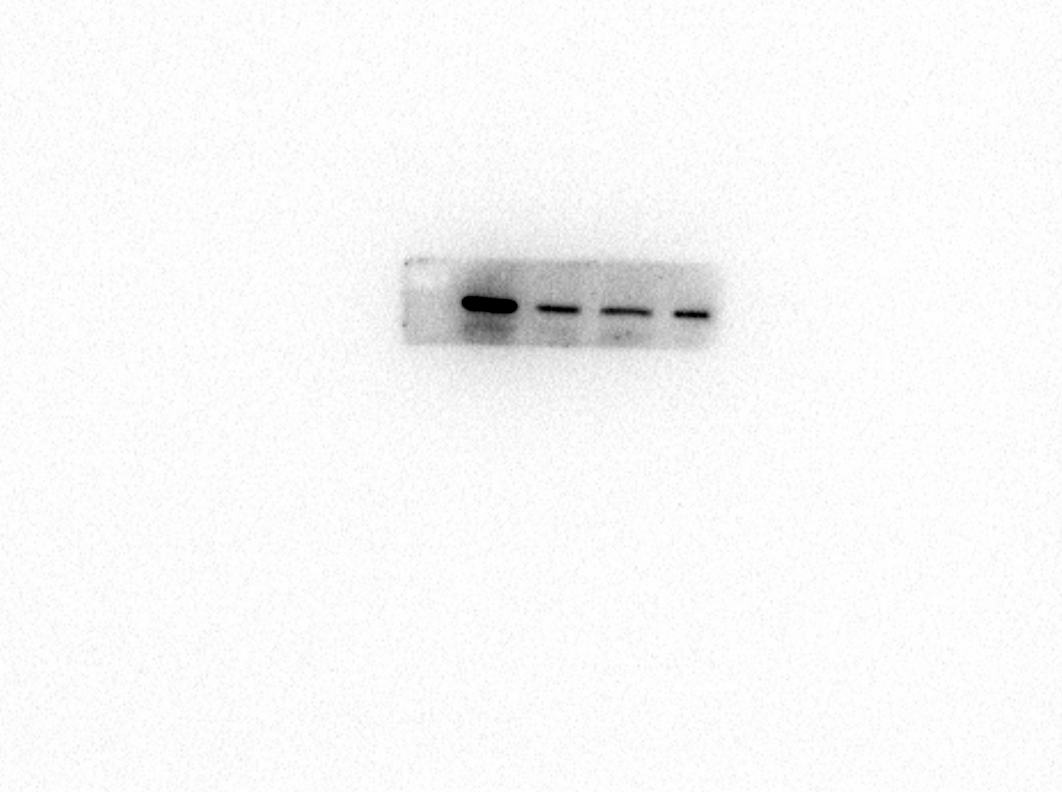

Supplement: Supplementary file 11 [file Data_Sheet_11.ZIP › FIG-5H/SW1116-siMETAP2/membrane SW1116-siMETAP2-METAP2.tif]

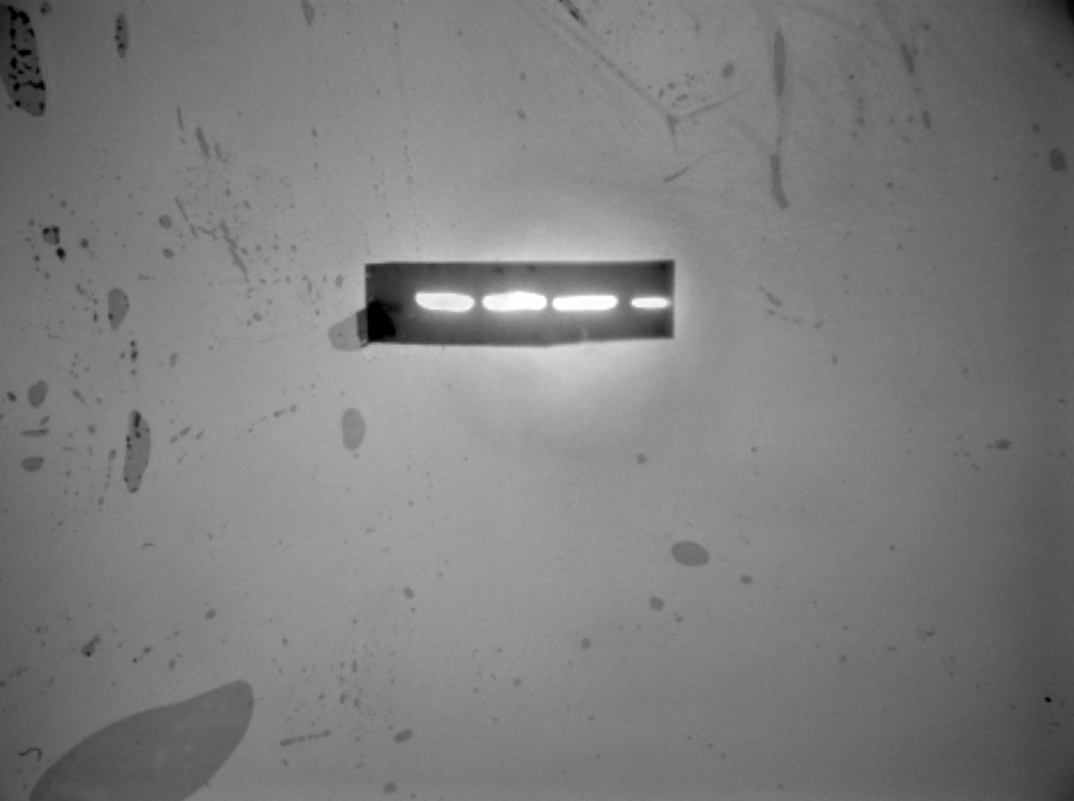

Supplement: Supplementary file 11 [file Data_Sheet_11.ZIP › FIG-5H/SW1116-siMETAP2/membrane with marker SW1116-siMETAP2-ACTIN.tif]

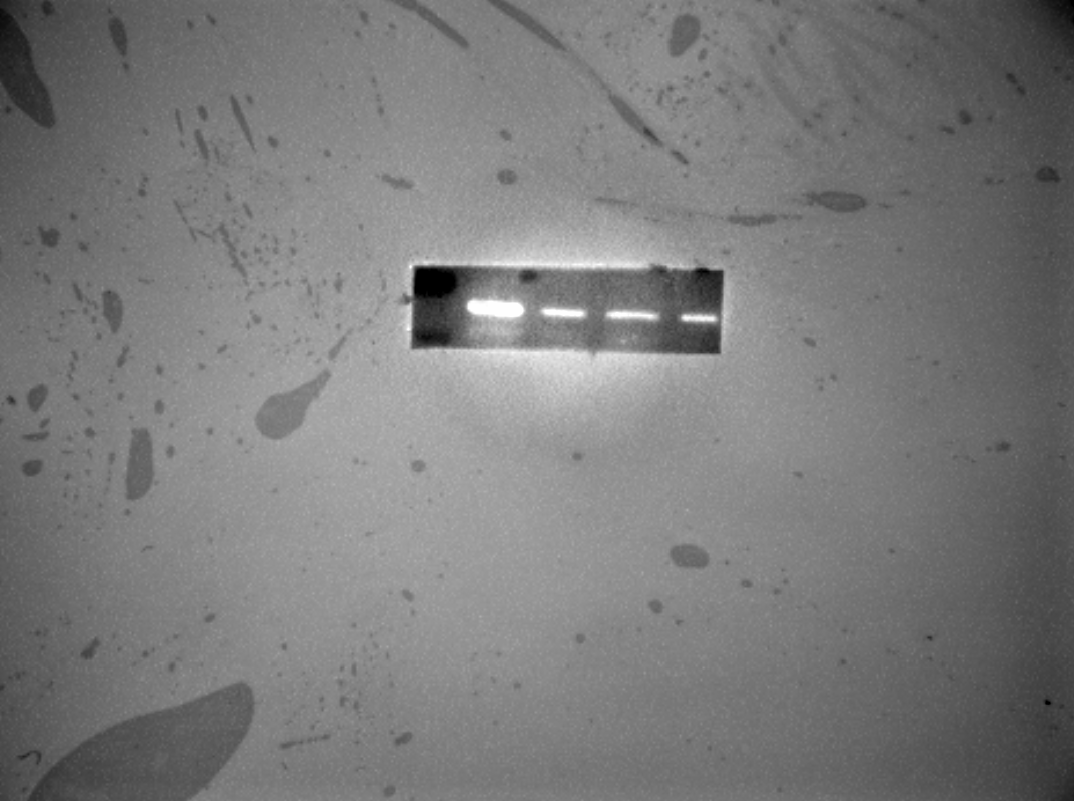

Supplement: Supplementary file 11 [file Data_Sheet_11.ZIP › FIG-5H/SW1116-siMETAP2/membrane with marker-SW1116-si-MAP2-MAP2.tif]

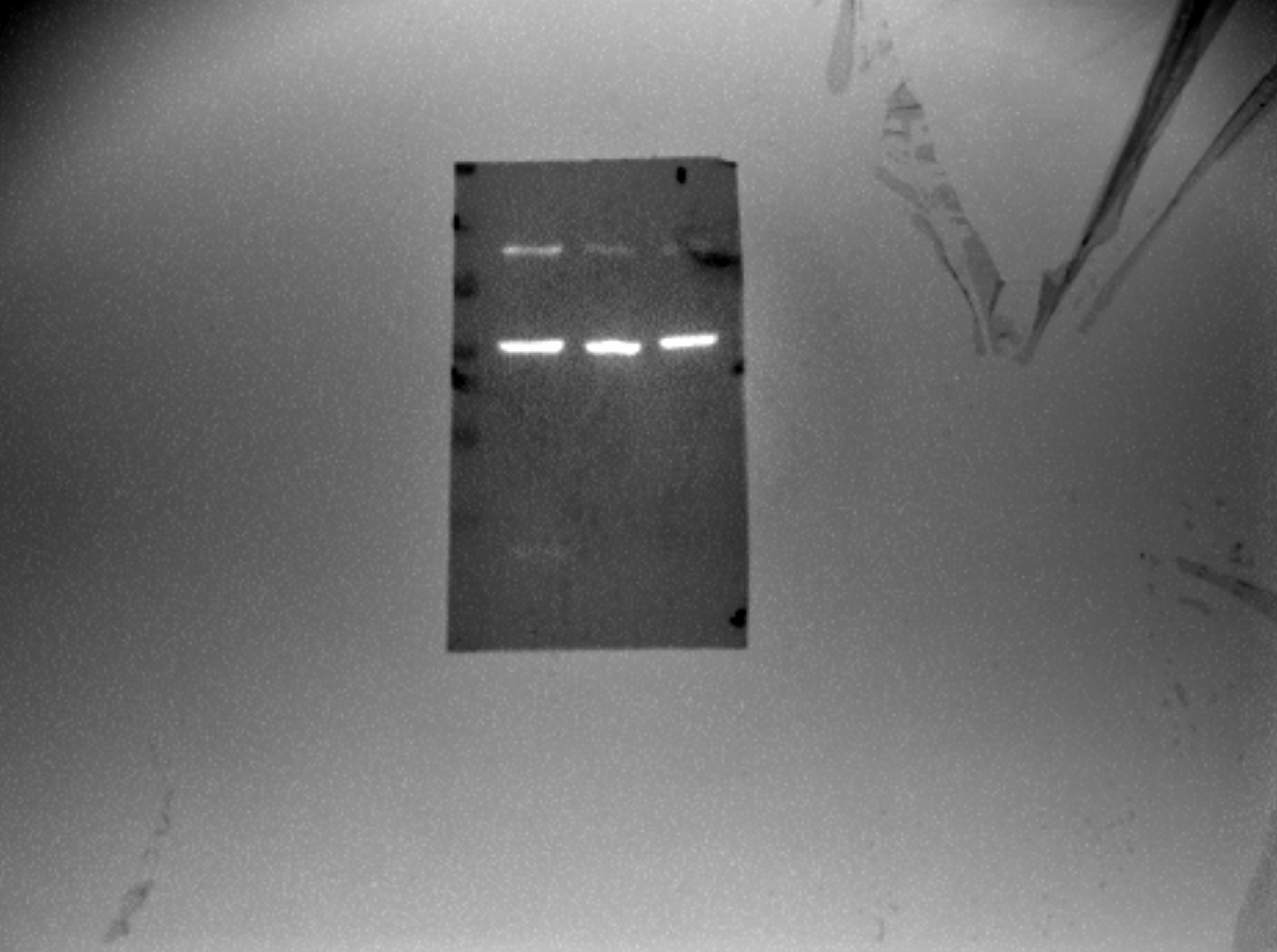

Supplement: Supplementary file 12 [file Data_Sheet_12.ZIP › FIG-6D/HCT116 -OK/membrane with marker-ACTIN.tif]

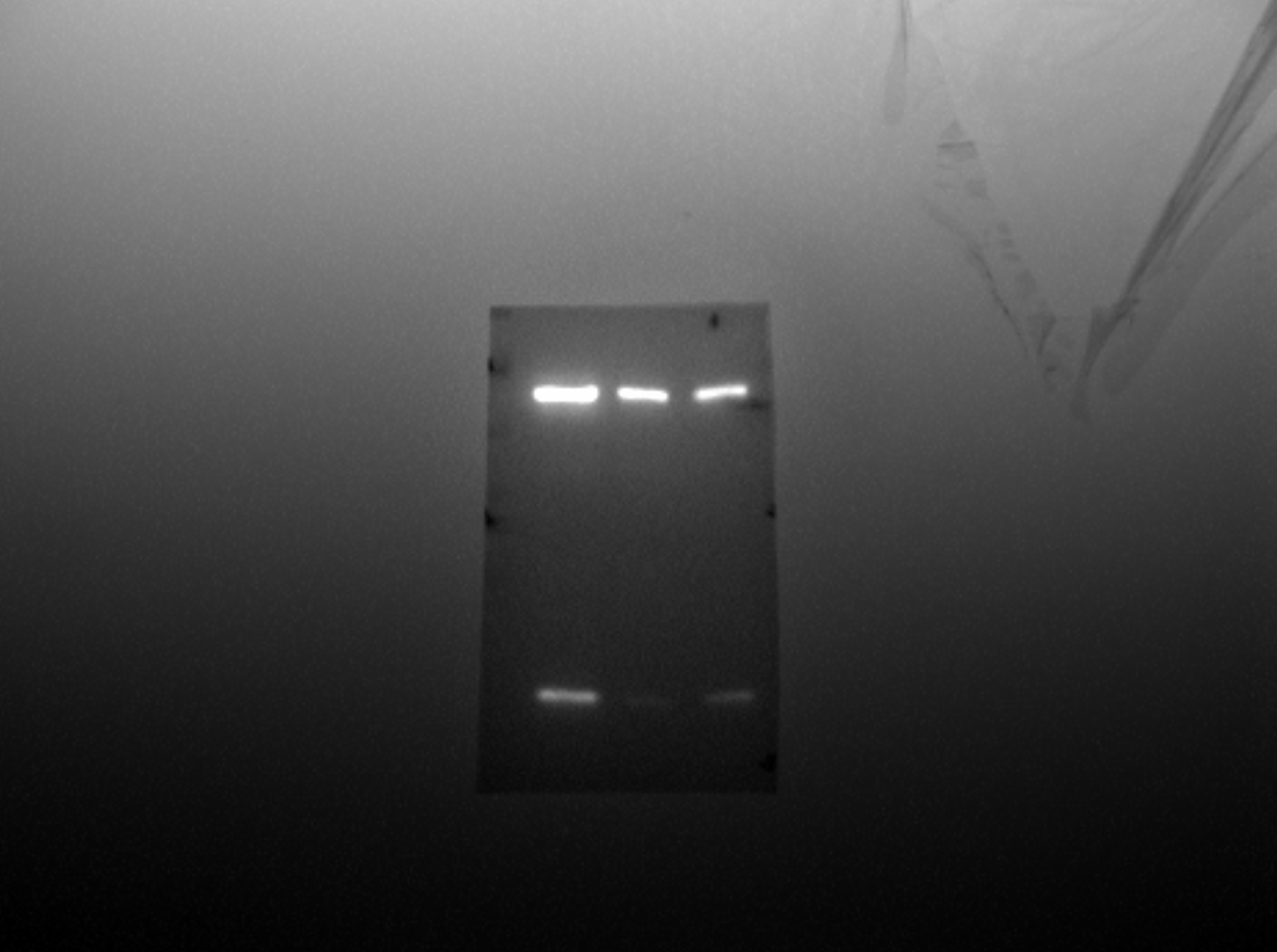

Supplement: Supplementary file 12 [file Data_Sheet_12.ZIP › FIG-6D/HCT116 -OK/membrane with marker-MAP2+LIN28A.tif]

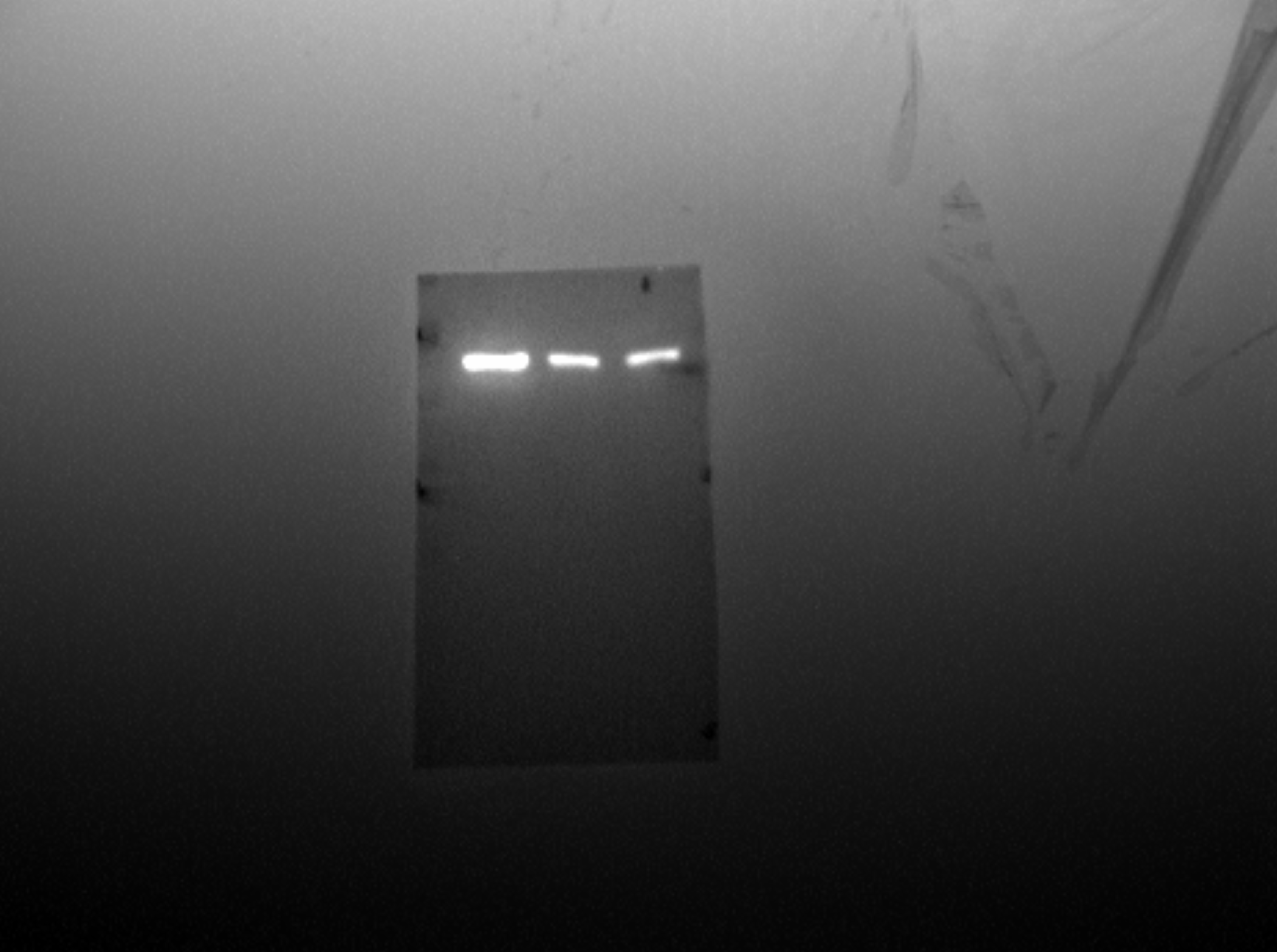

Supplement: Supplementary file 12 [file Data_Sheet_12.ZIP › FIG-6D/HCT116 -OK/membrane with marker-MAP2.tif]

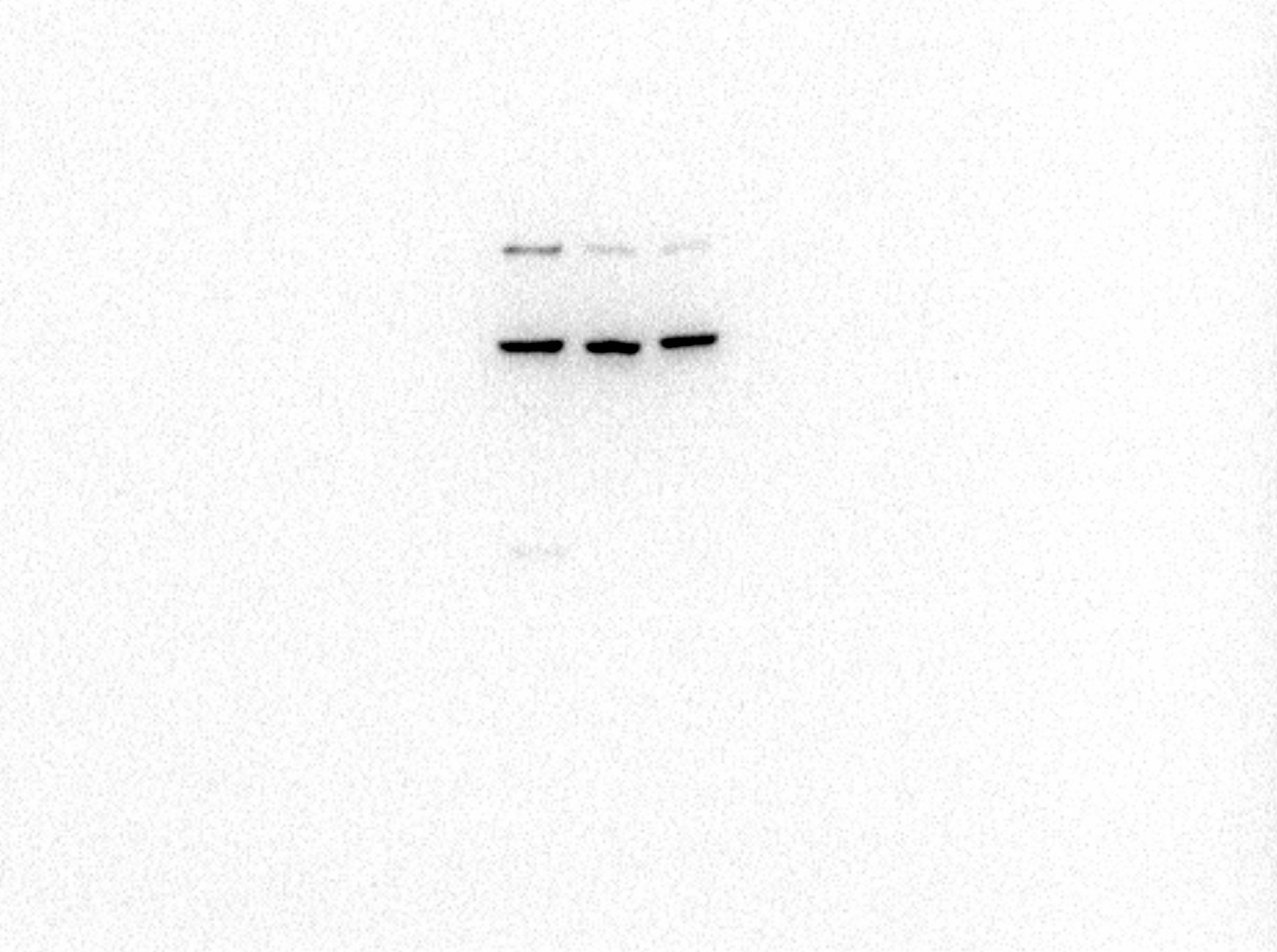

Supplement: Supplementary file 12 [file Data_Sheet_12.ZIP › FIG-6D/HCT116 -OK/membrane-ACTIN.tif]

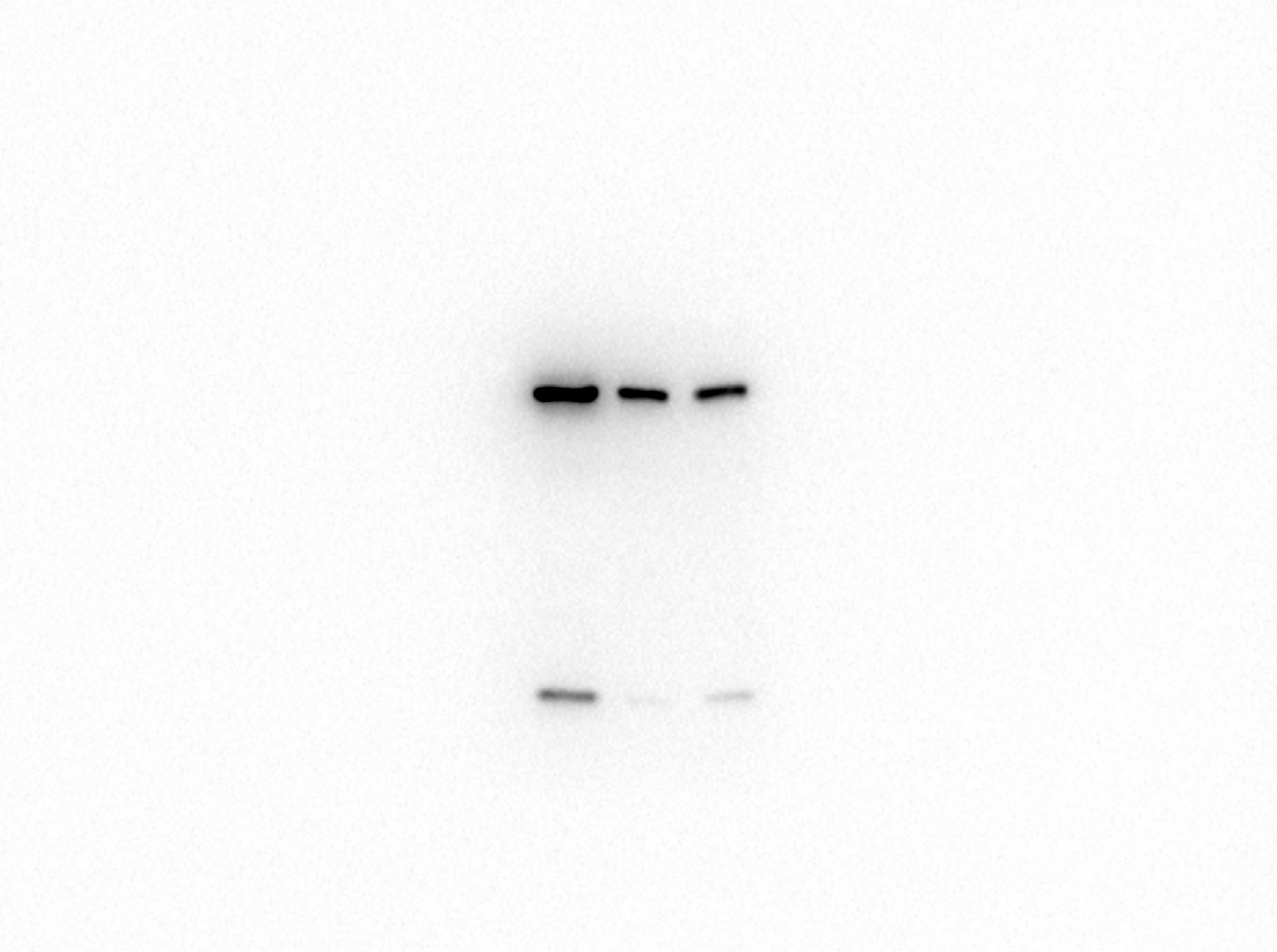

Supplement: Supplementary file 12 [file Data_Sheet_12.ZIP › FIG-6D/HCT116 -OK/membrane-MAP2+LIN28A.tif]

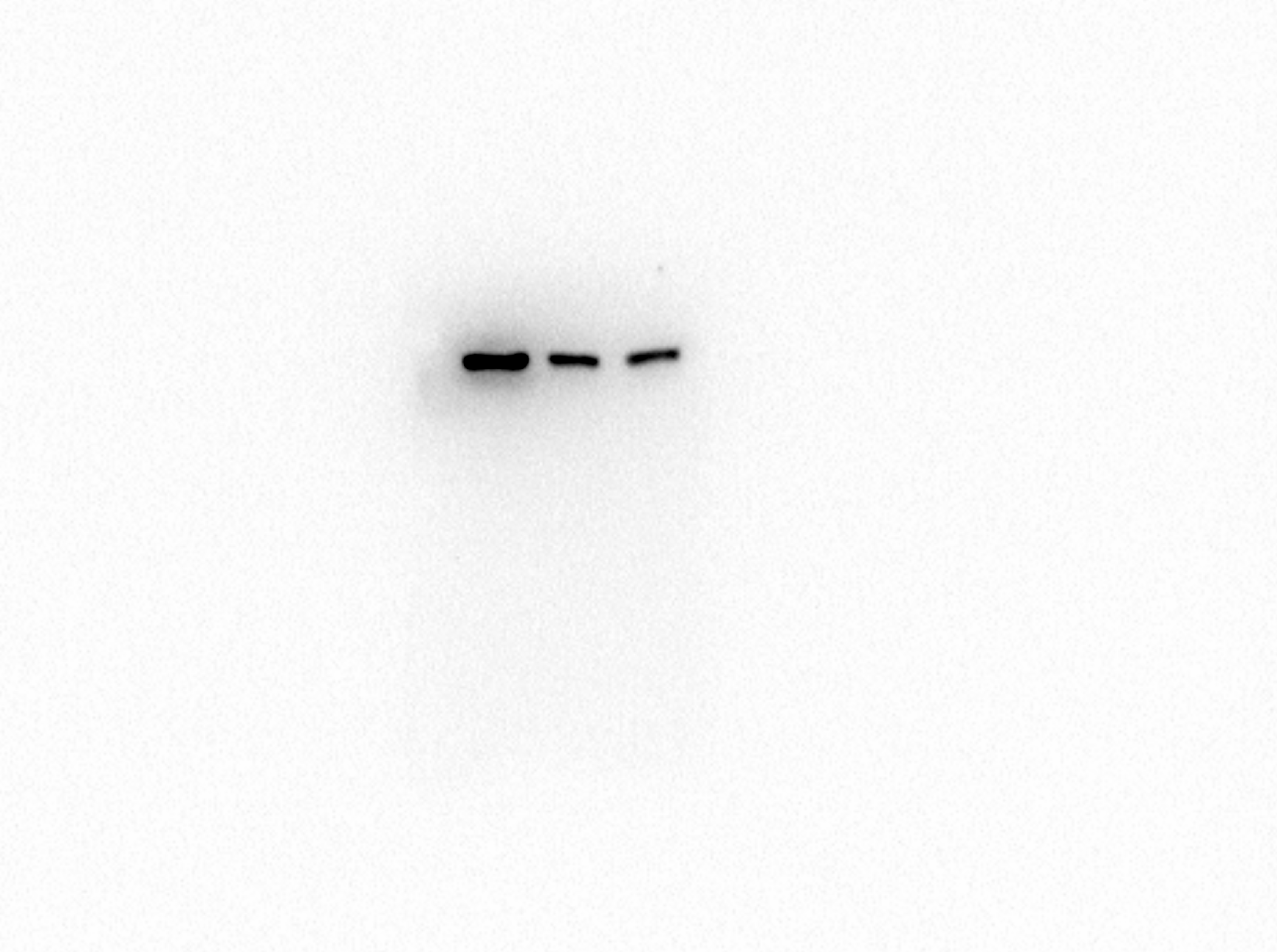

Supplement: Supplementary file 12 [file Data_Sheet_12.ZIP › FIG-6D/HCT116 -OK/membrane-MAP2.tif]

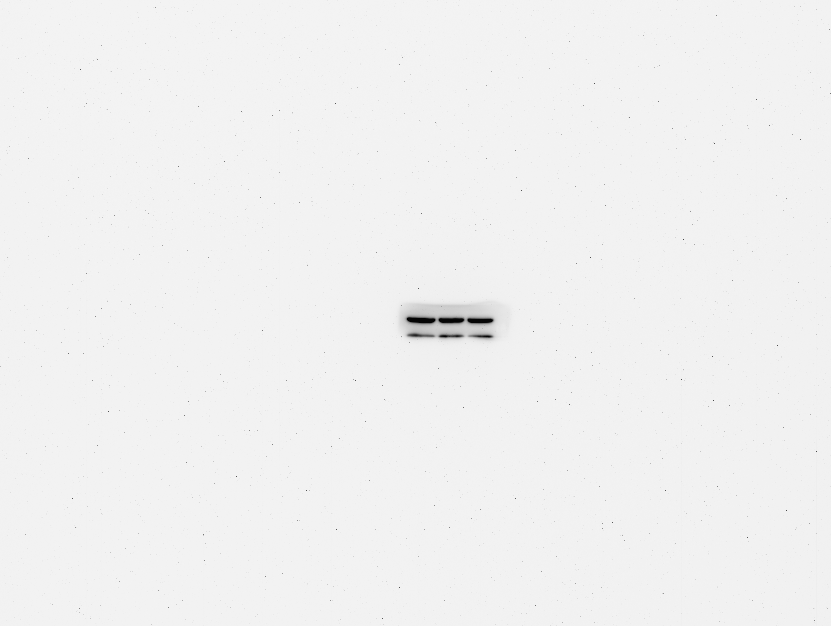

Supplement: Supplementary file 12 [file Data_Sheet_12.ZIP › FIG-6D/SW1116/membrane SW1116-siMETAP2-ACTIN_8bit.png]

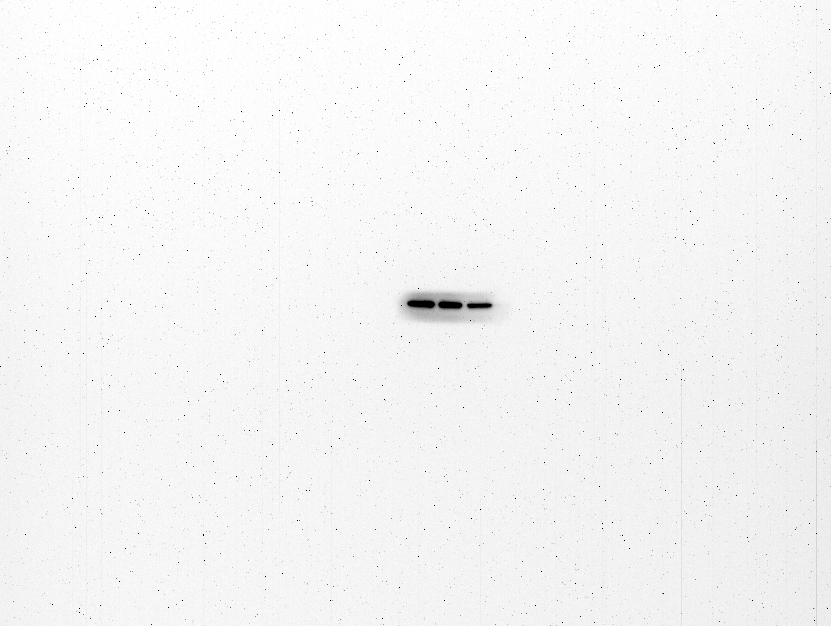

Supplement: Supplementary file 12 [file Data_Sheet_12.ZIP › FIG-6D/SW1116/membrane SW1116-siMETAP2-METAP2_8bit.png]

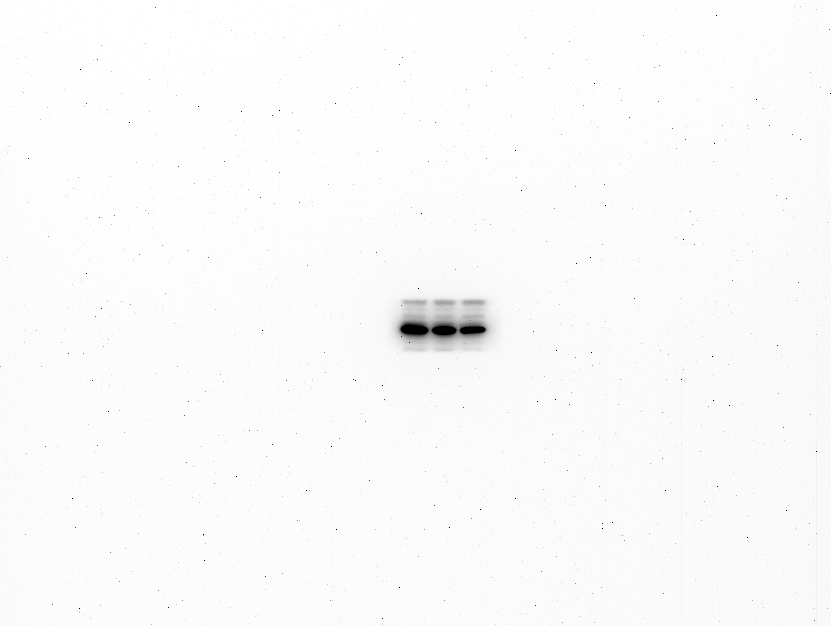

Supplement: Supplementary file 12 [file Data_Sheet_12.ZIP › FIG-6D/SW1116/membrane SW1116-siMWTAP2-LIN28A.png]

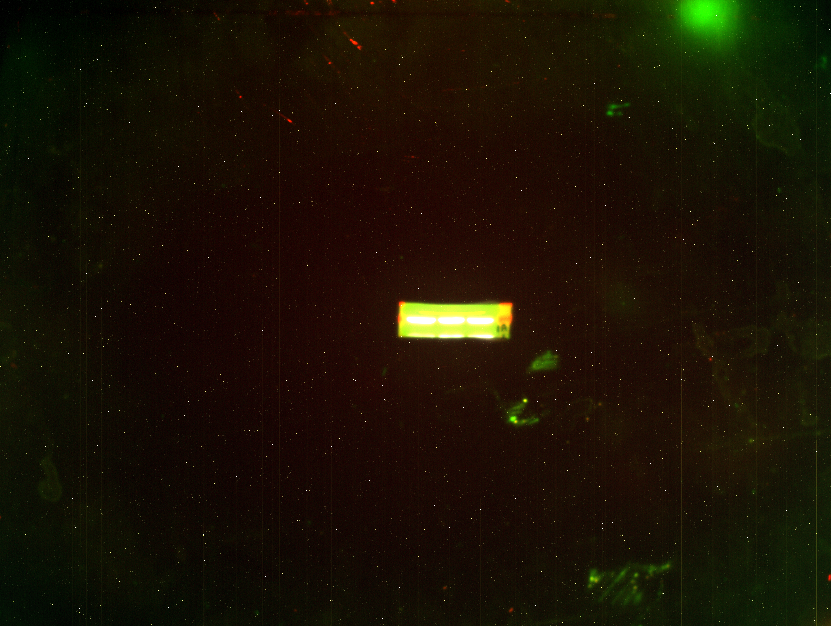

Supplement: Supplementary file 12 [file Data_Sheet_12.ZIP › FIG-6D/SW1116/membrane with marker SW1116-siMETAP2-ACTIN_8bit.png]

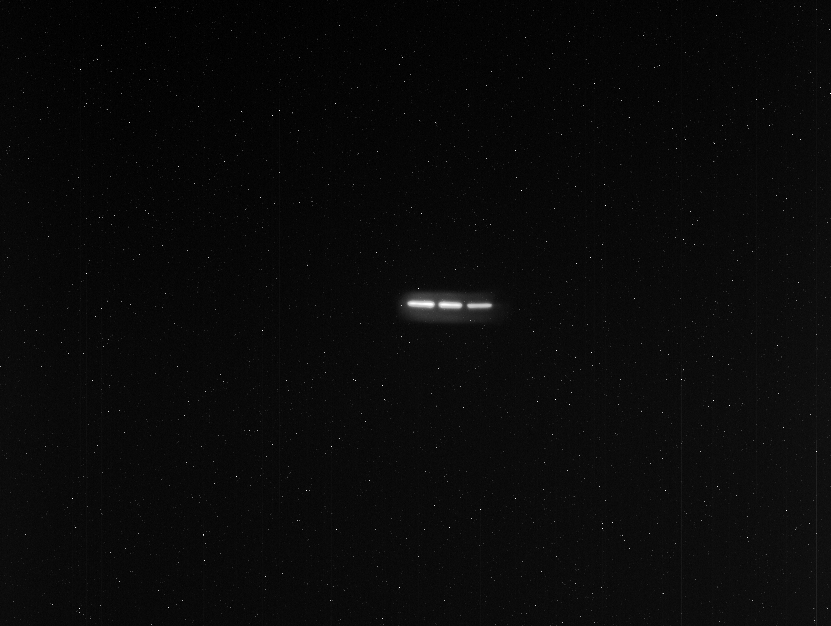

Supplement: Supplementary file 12 [file Data_Sheet_12.ZIP › FIG-6D/SW1116/membrane with marker SW1116-siMETAP2-METAP2.png]

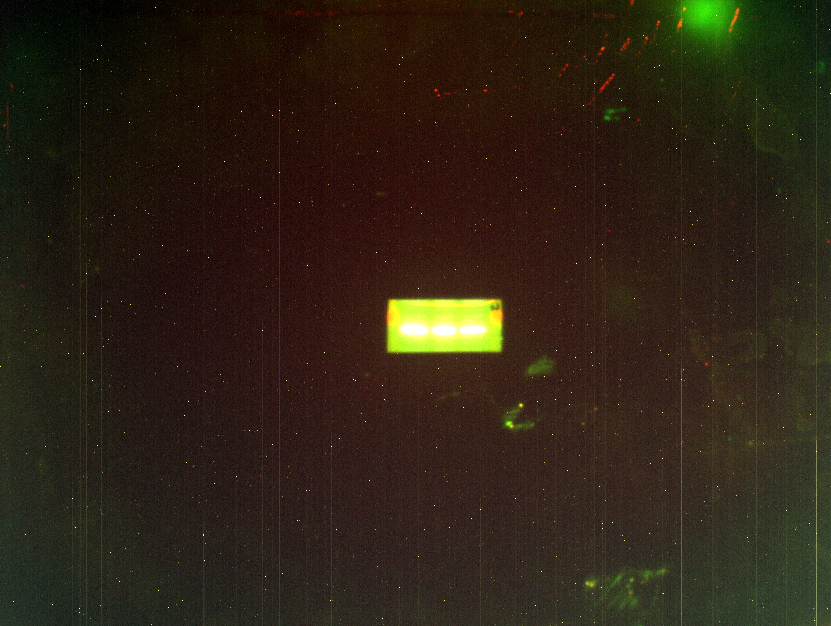

Supplement: Supplementary file 12 [file Data_Sheet_12.ZIP › FIG-6D/SW1116/membrane with marker SW1116-siMWTAP2-LIN28A.png]

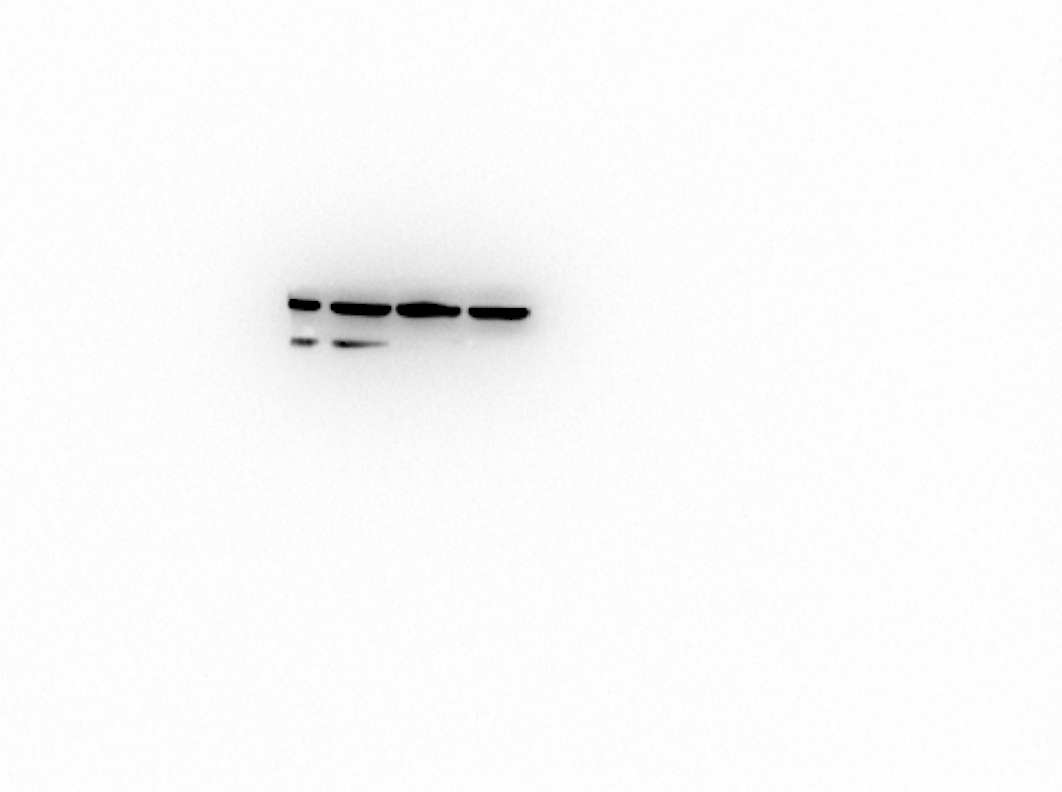

Supplement: Supplementary file 13 [file Data_Sheet_13.ZIP › FIG-6E/HCT116/membrane HCT116-ACTIN.tif]

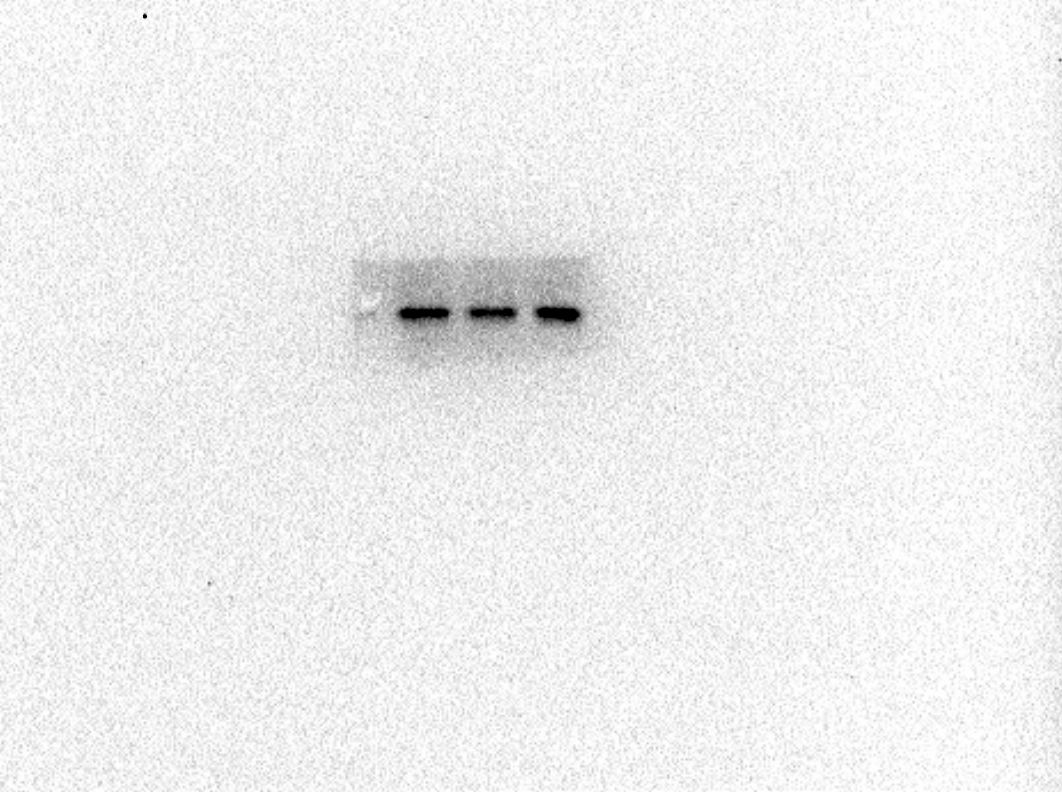

Supplement: Supplementary file 13 [file Data_Sheet_13.ZIP › FIG-6E/HCT116/membrane HCT116-LIN28A.tif]

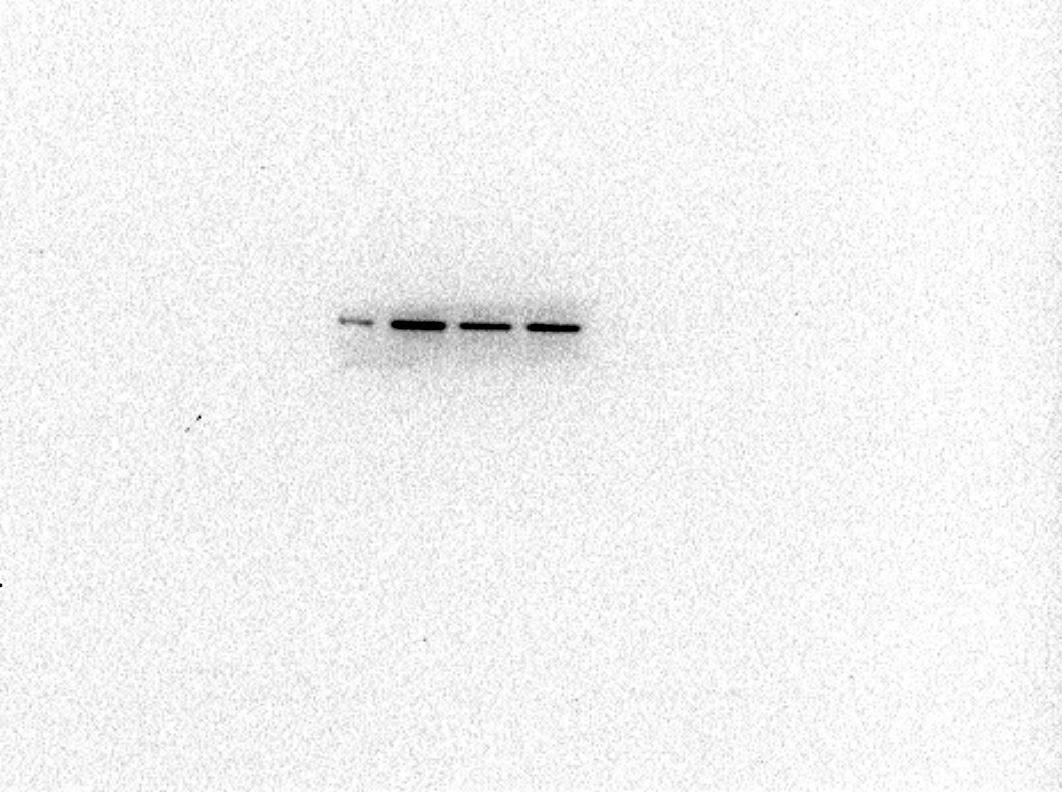

Supplement: Supplementary file 13 [file Data_Sheet_13.ZIP › FIG-6E/HCT116/membrane HCT116-MAP2.tif]

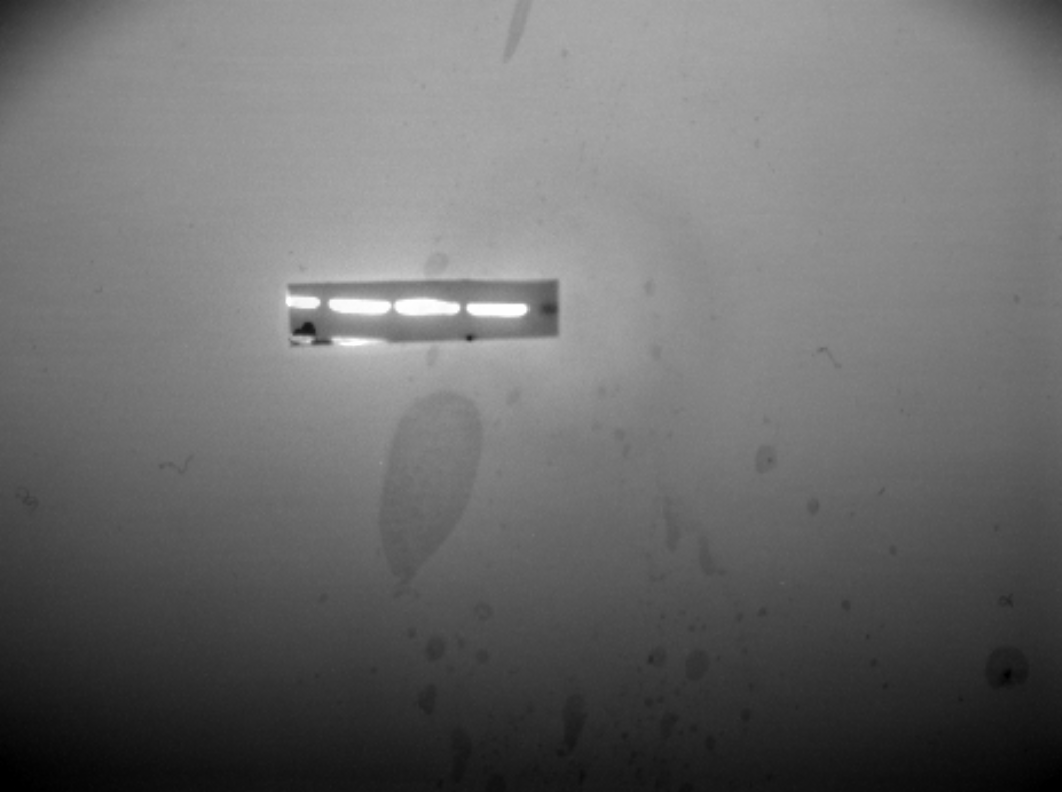

Supplement: Supplementary file 13 [file Data_Sheet_13.ZIP › FIG-6E/HCT116/membrane with marker HCT116-ACTIN.tif]

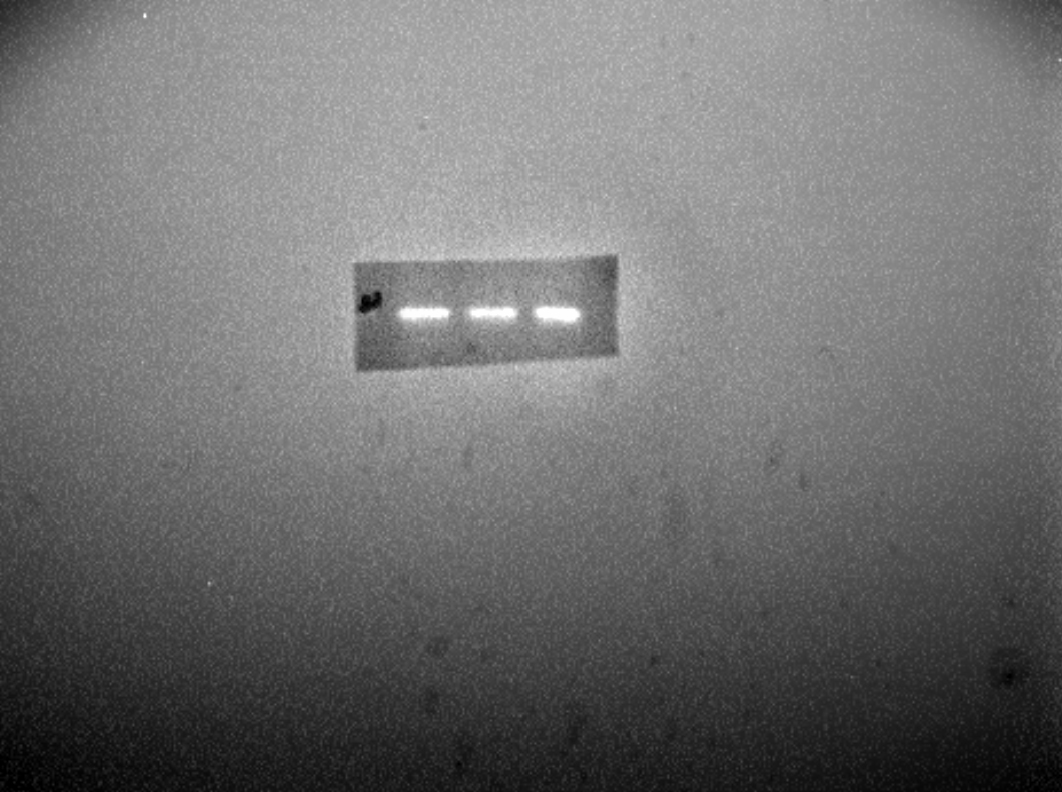

Supplement: Supplementary file 13 [file Data_Sheet_13.ZIP › FIG-6E/HCT116/membrane with marker HCT116-LIN28A.tif]

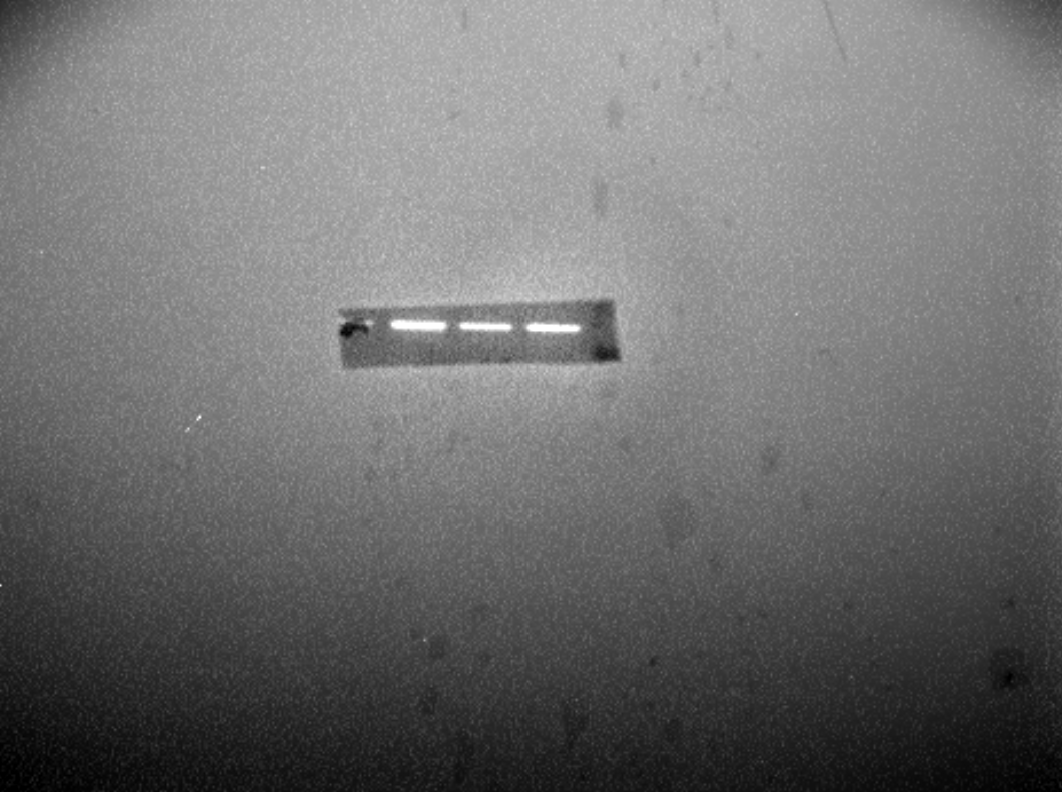

Supplement: Supplementary file 13 [file Data_Sheet_13.ZIP › FIG-6E/HCT116/membrane with marker HCT116-MAP2.tif]

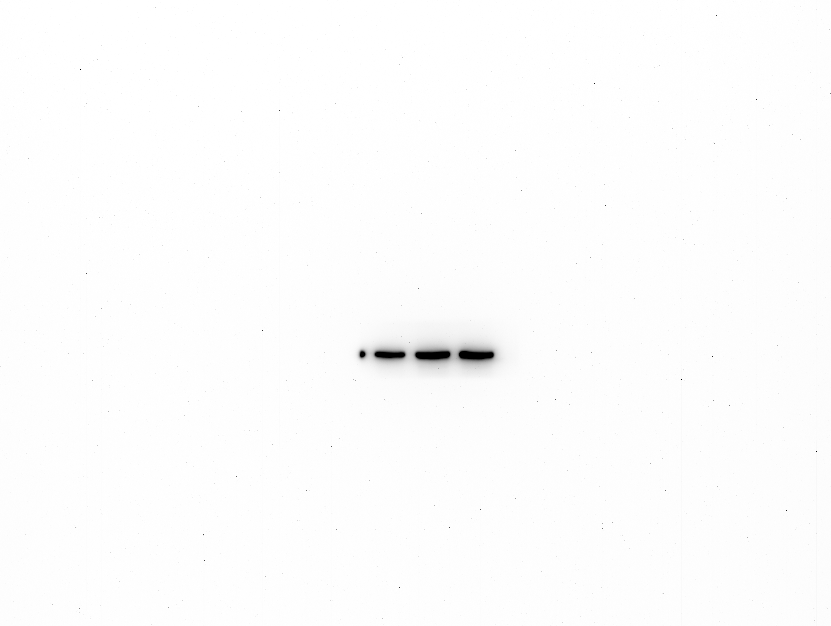

Supplement: Supplementary file 13 [file Data_Sheet_13.ZIP › FIG-6E/SW1116/membrane SW1116-ACTIN.png]

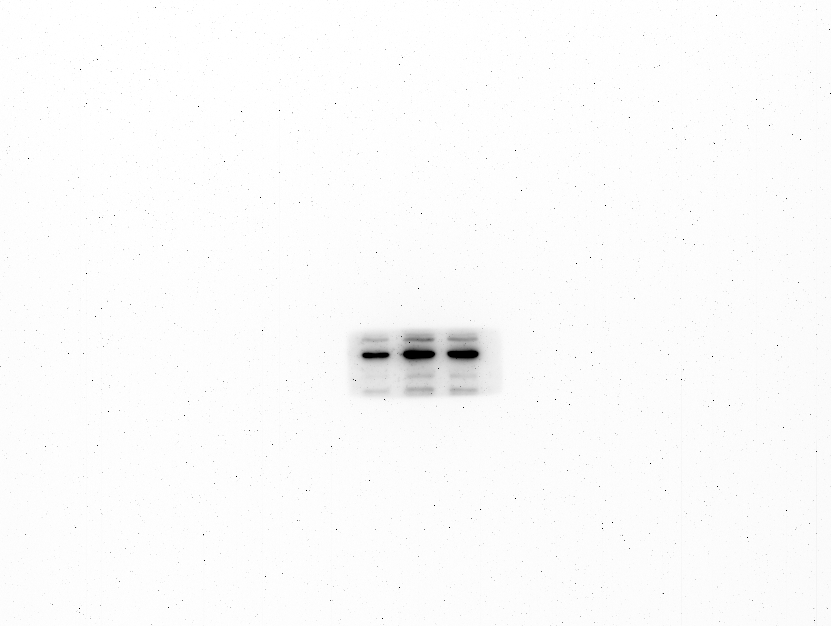

Supplement: Supplementary file 13 [file Data_Sheet_13.ZIP › FIG-6E/SW1116/membrane SW1116-LIN28A.png]

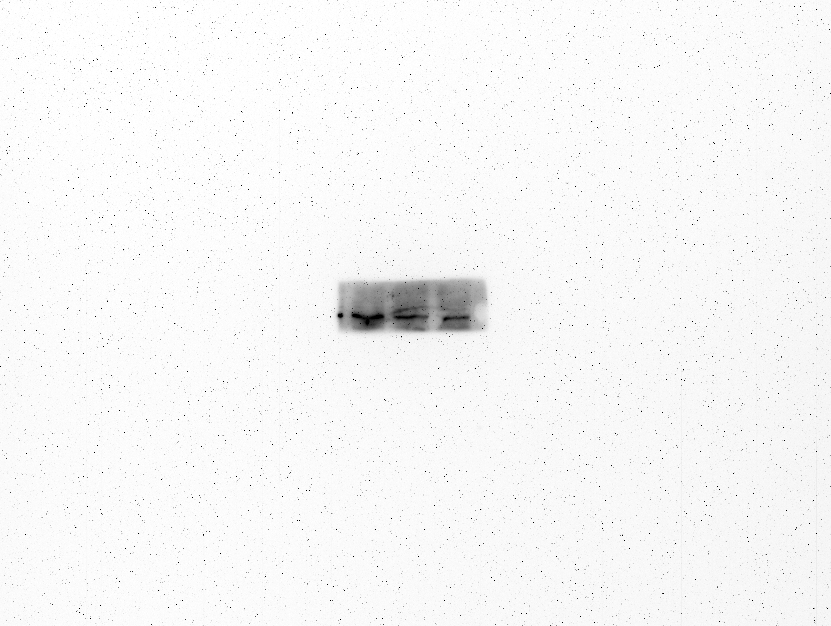

Supplement: Supplementary file 13 [file Data_Sheet_13.ZIP › FIG-6E/SW1116/membrane SW1116-METAP2.png]

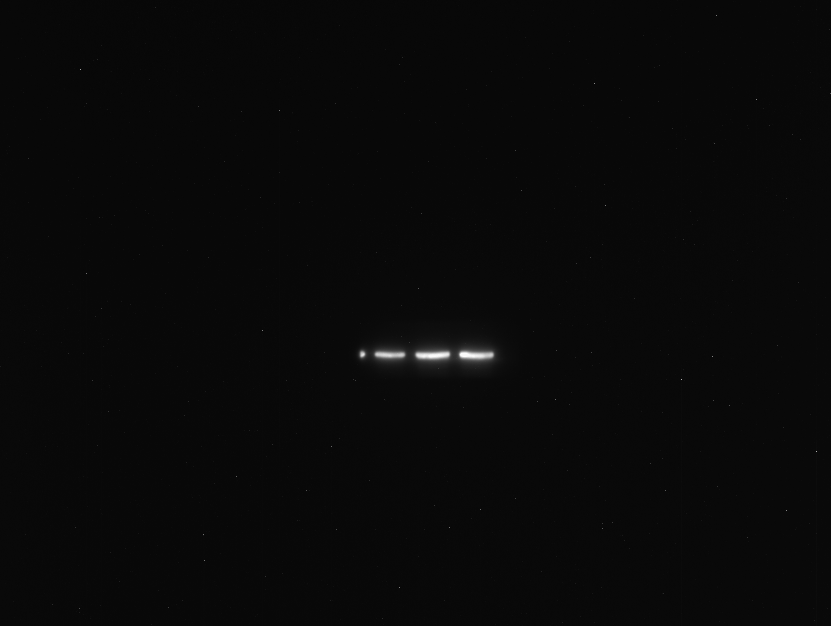

Supplement: Supplementary file 13 [file Data_Sheet_13.ZIP › FIG-6E/SW1116/membrane with marker SW1116-ACTIN.png]

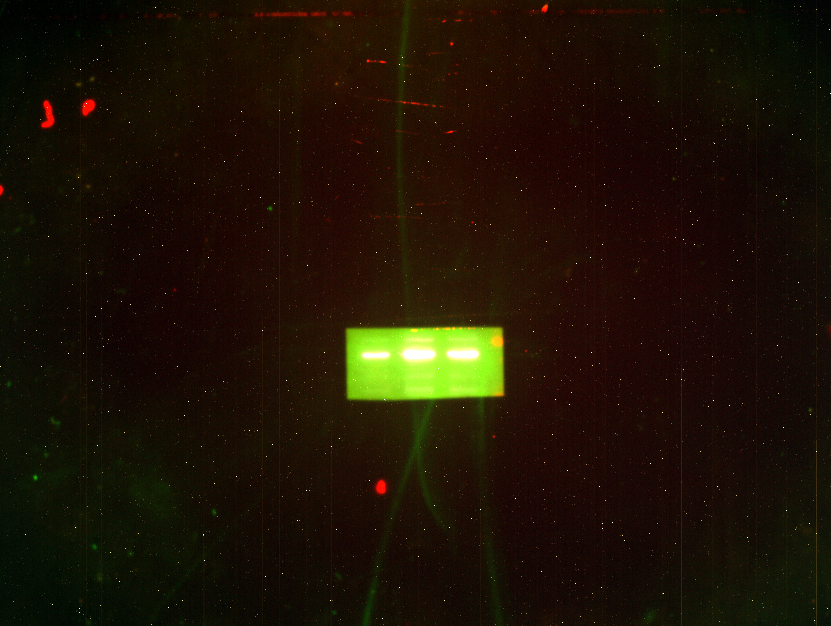

Supplement: Supplementary file 13 [file Data_Sheet_13.ZIP › FIG-6E/SW1116/membrane with marker SW1116-LIN28A.png]

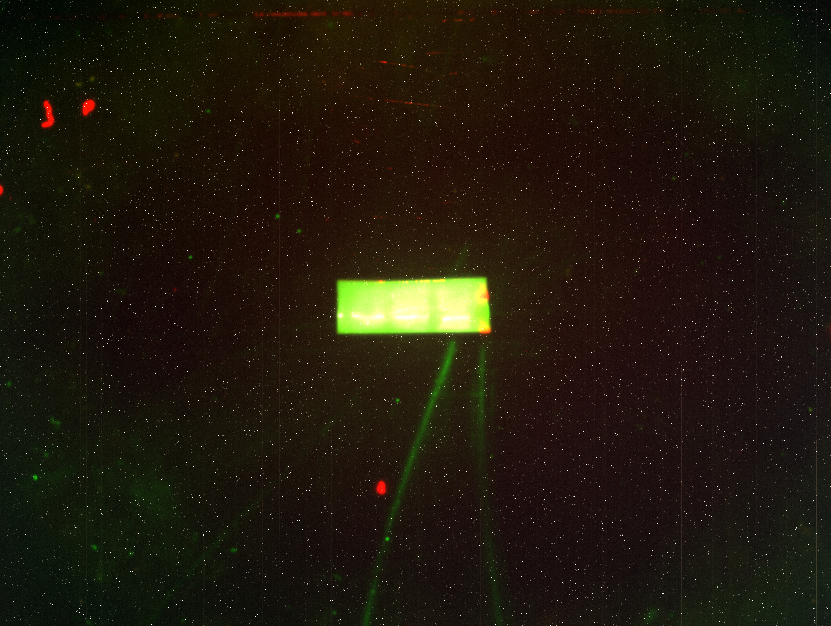

Supplement: Supplementary file 13 [file Data_Sheet_13.ZIP › FIG-6E/SW1116/membrane with marker SW1116-METAP2.png]

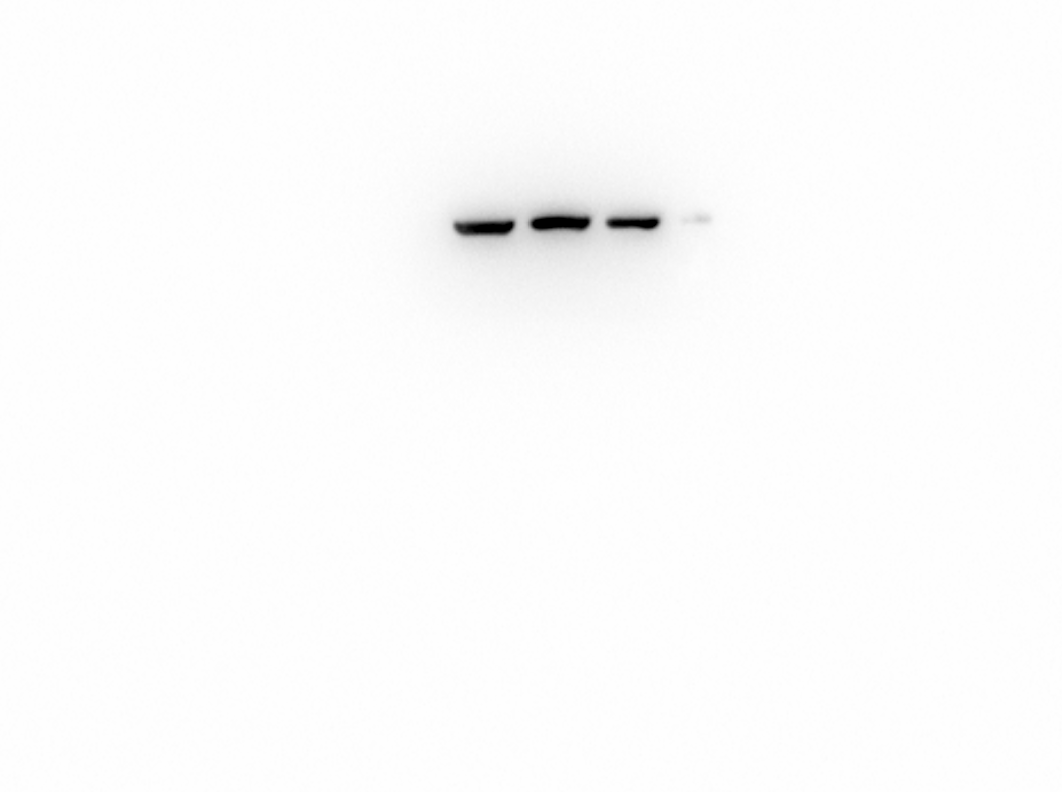

Supplement: Supplementary file 13 [file Data_Sheet_13.ZIP › FIG-6H/HCT116/membrane HCT116-ACTIN.tif]

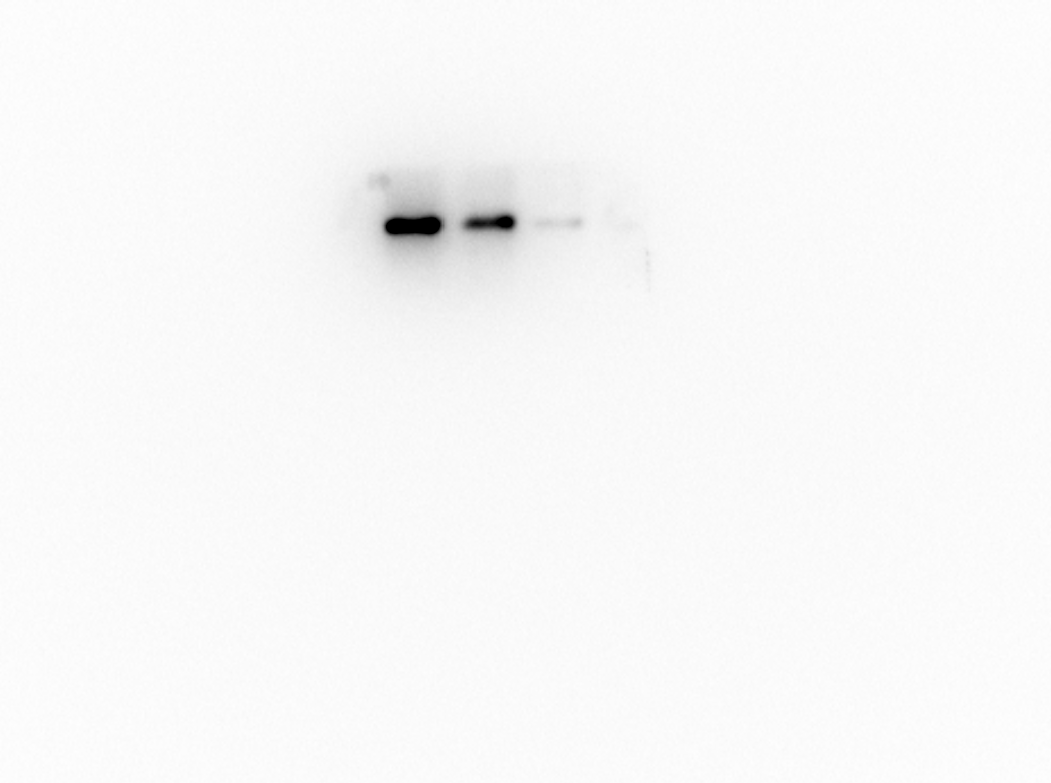

Supplement: Supplementary file 13 [file Data_Sheet_13.ZIP › FIG-6H/HCT116/membrane HCT116-LIN28A.tif]

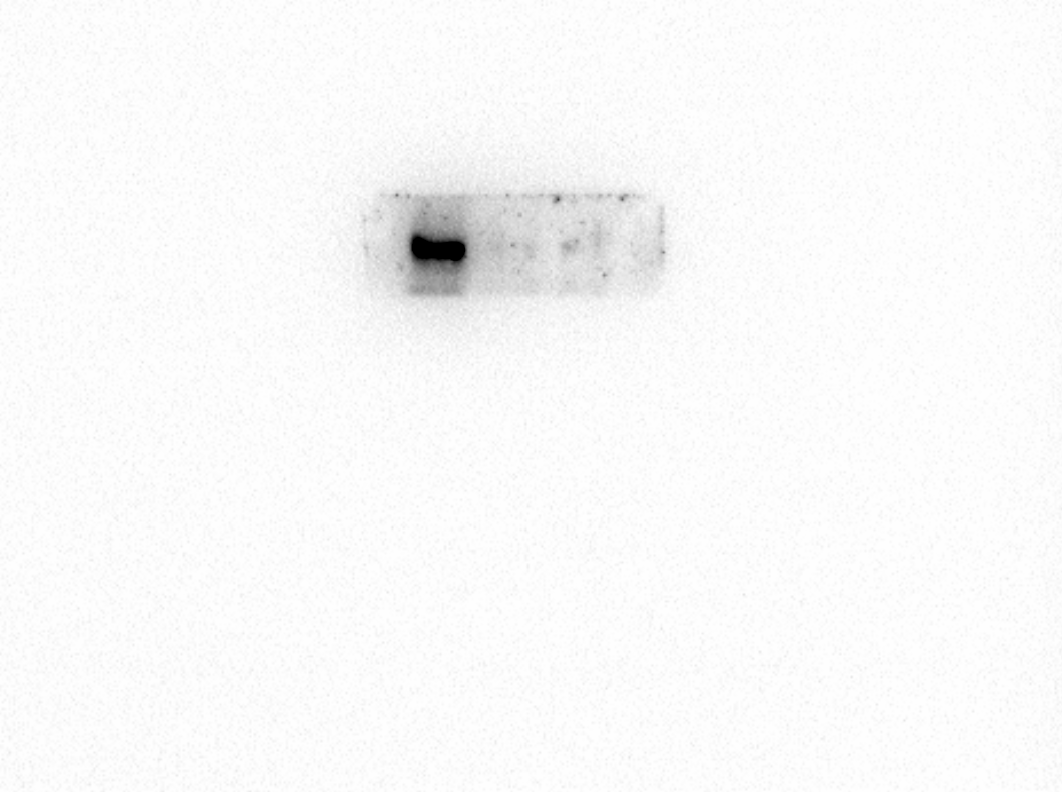

Supplement: Supplementary file 13 [file Data_Sheet_13.ZIP › FIG-6H/HCT116/membrane HCT116-METAP2.tif]

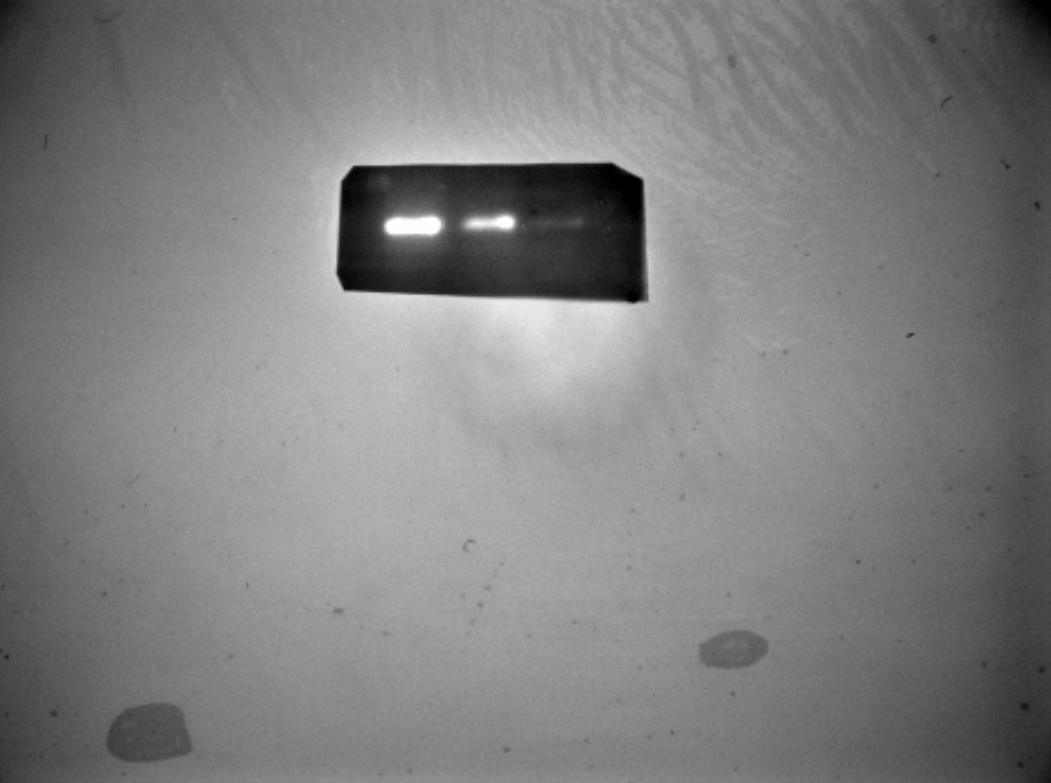

Supplement: Supplementary file 13 [file Data_Sheet_13.ZIP › FIG-6H/HCT116/membrane with marker HCT116-LIN28A.tif]

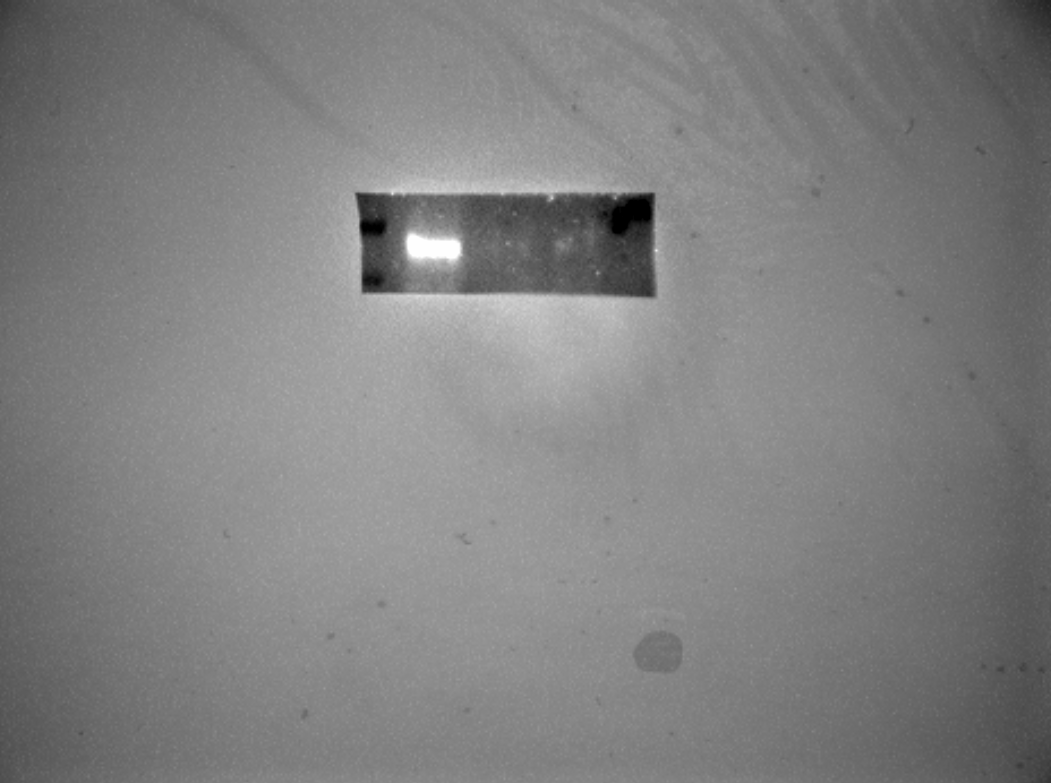

Supplement: Supplementary file 13 [file Data_Sheet_13.ZIP › FIG-6H/HCT116/membrane with marker HCT116-METAP2.tif]

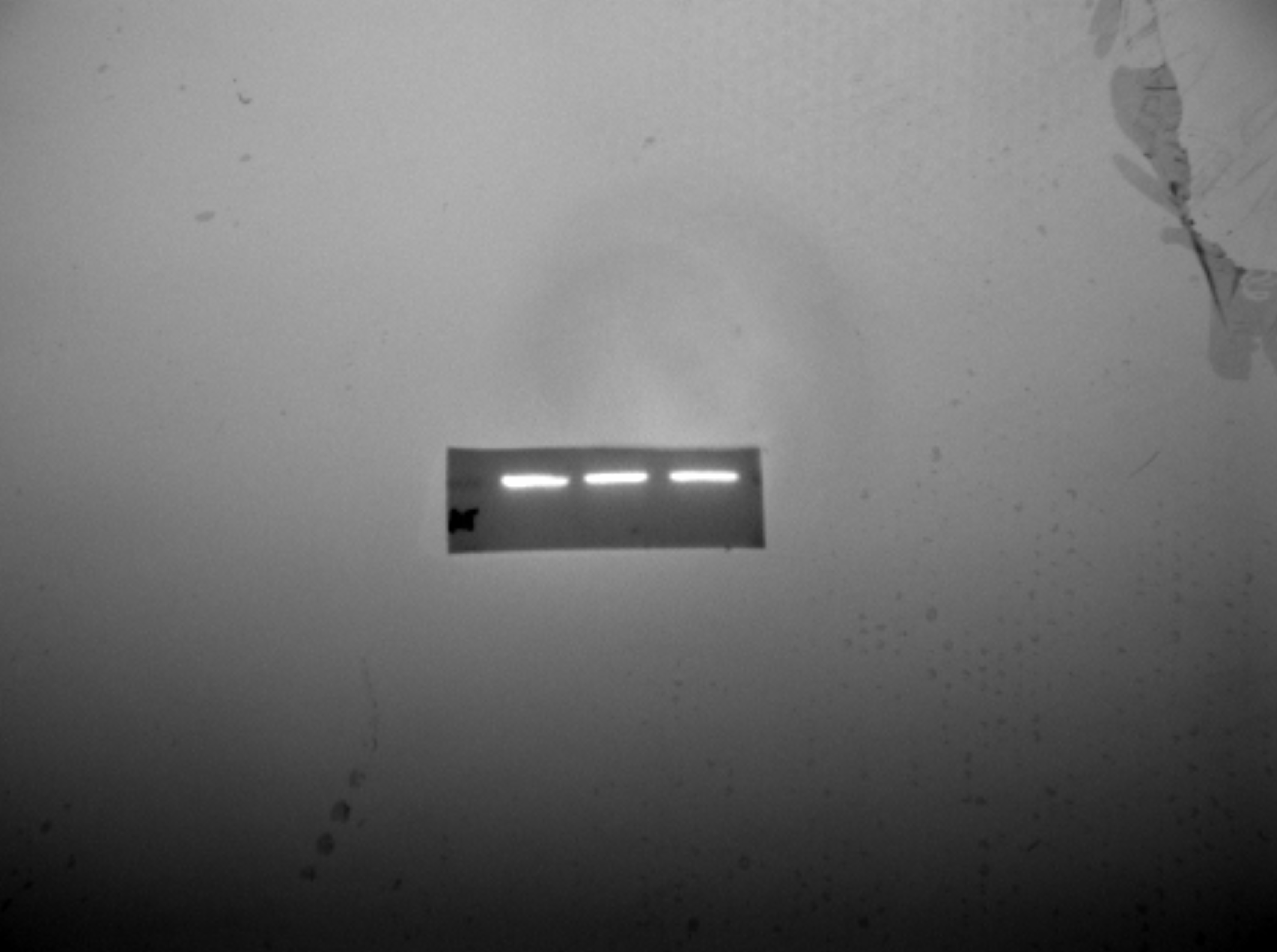

Supplement: Supplementary file 13 [file Data_Sheet_13.ZIP › FIG-6H/SW1116/membrane with marker-ACTIN.tif]

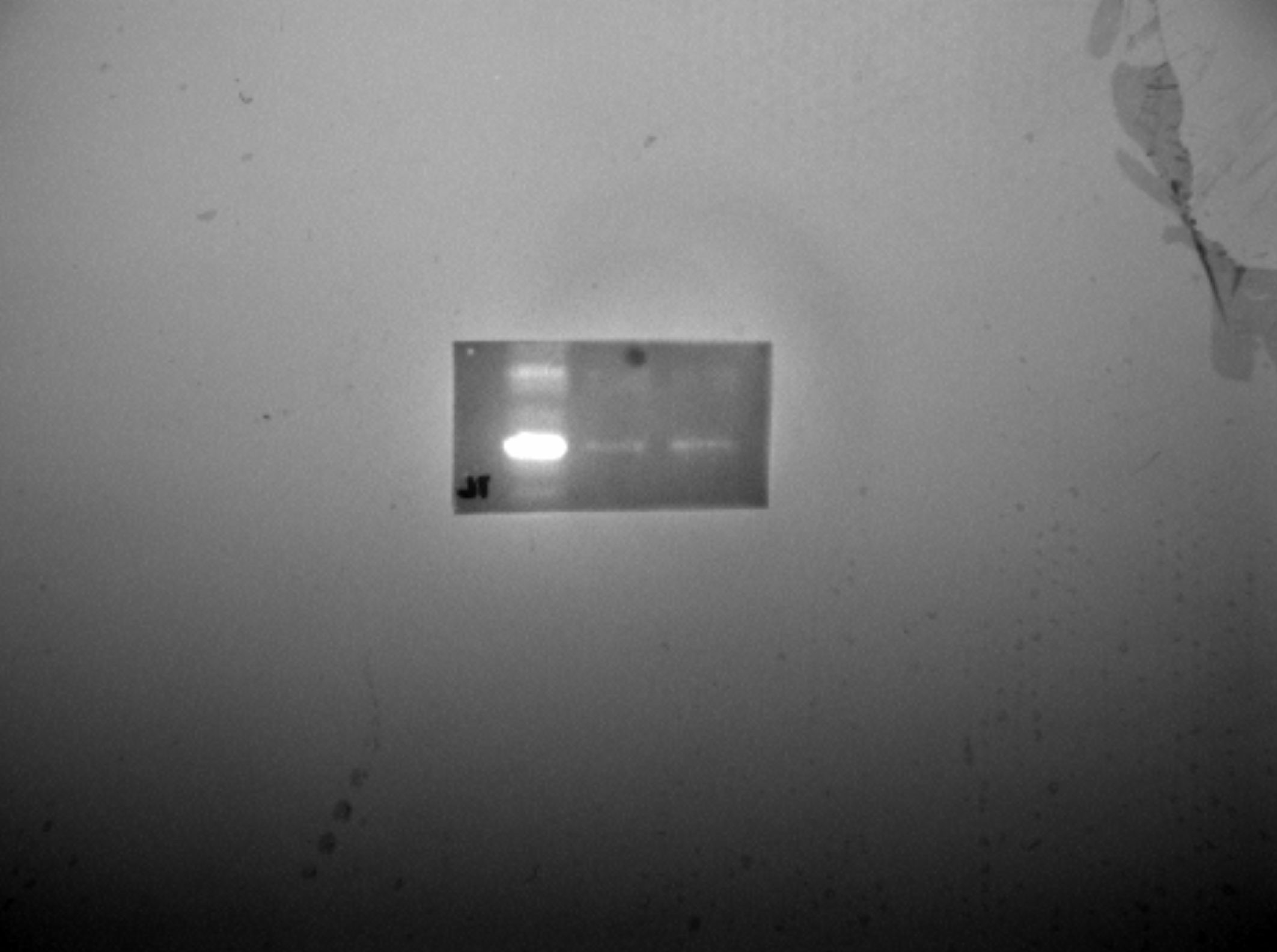

Supplement: Supplementary file 13 [file Data_Sheet_13.ZIP › FIG-6H/SW1116/membrane with marker-LIN28A.tif]

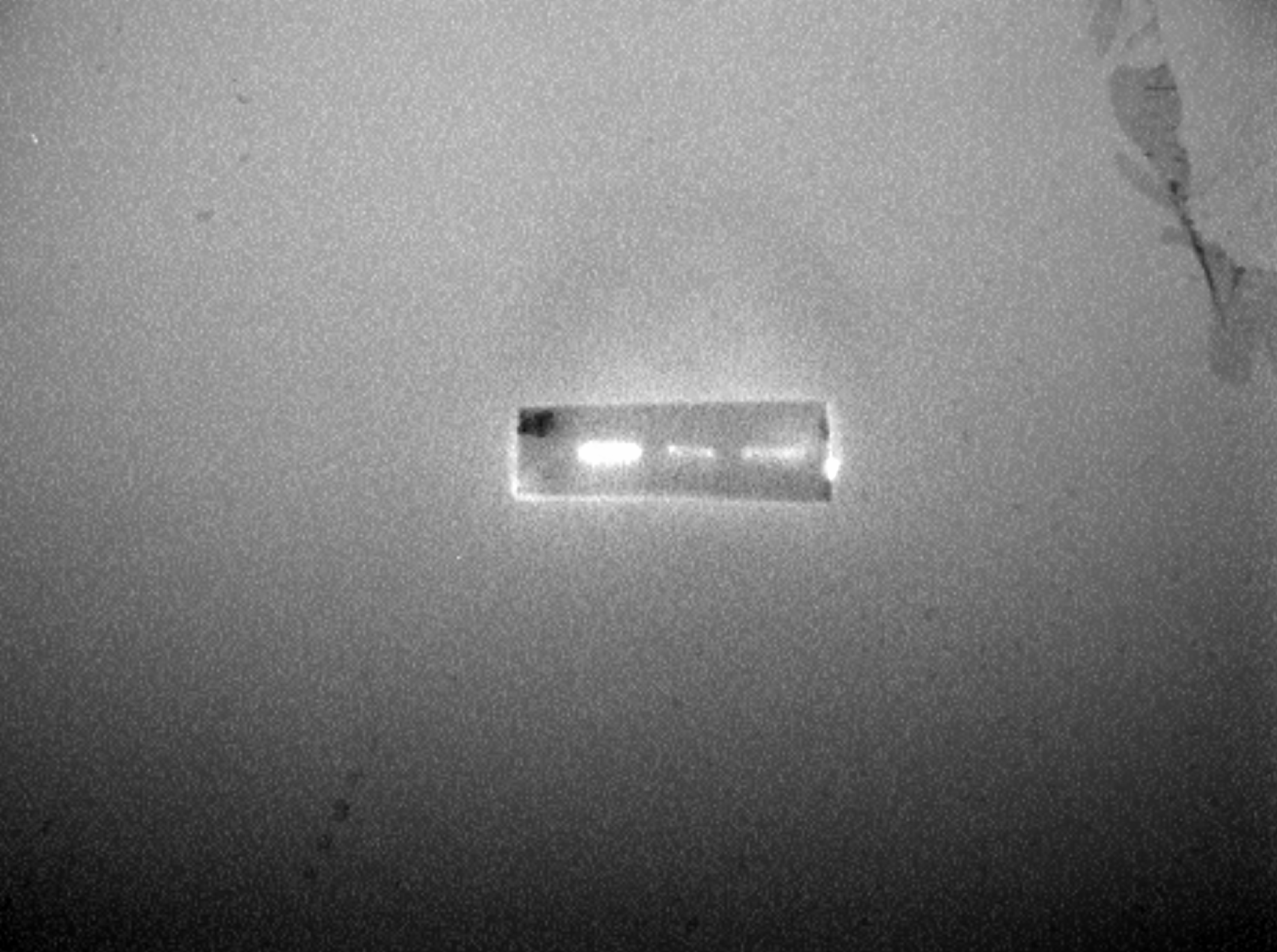

Supplement: Supplementary file 13 [file Data_Sheet_13.ZIP › FIG-6H/SW1116/membrane with marker-METAP2.tif]

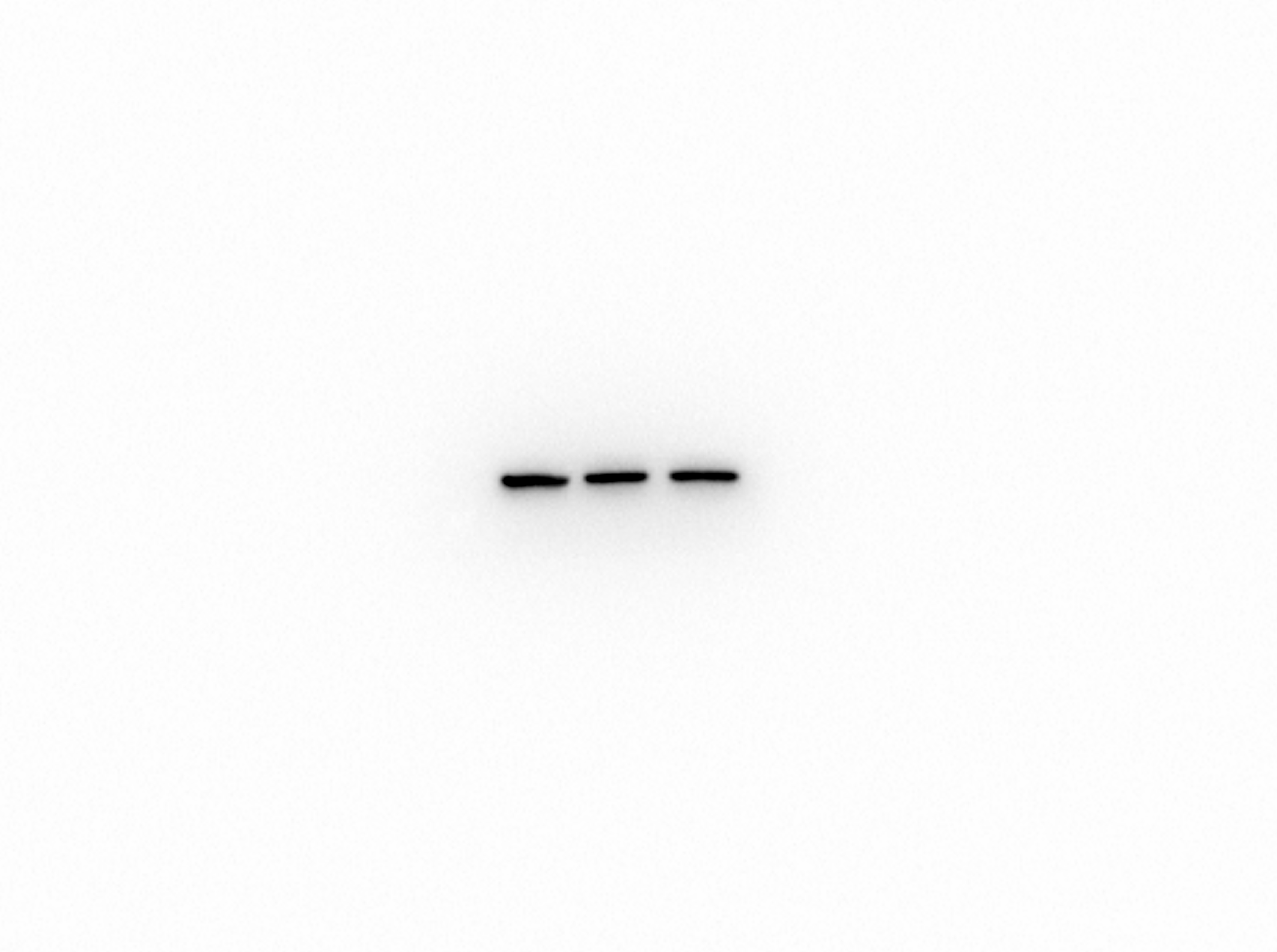

Supplement: Supplementary file 13 [file Data_Sheet_13.ZIP › FIG-6H/SW1116/membrane-ACTIN.tif]

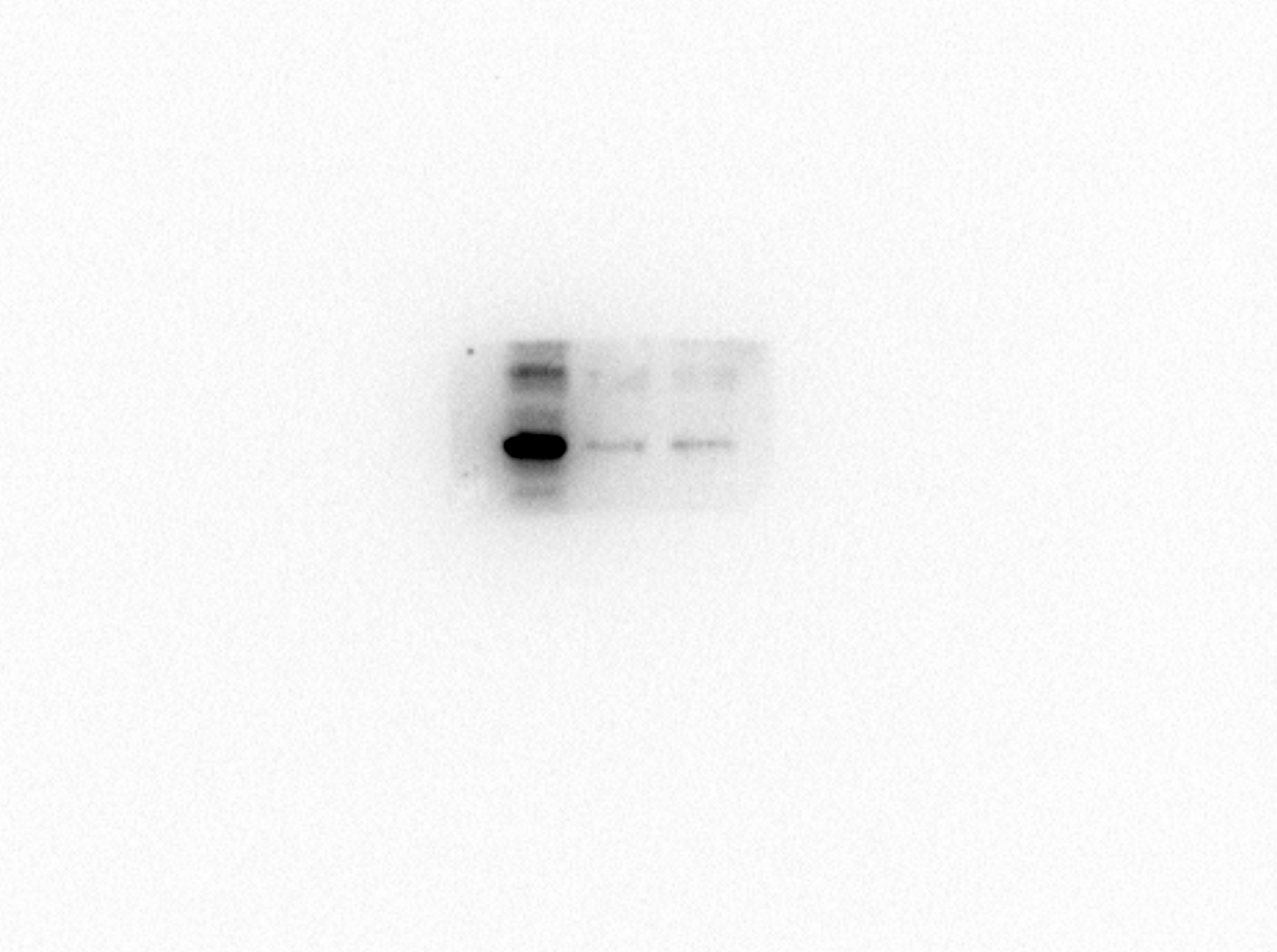

Supplement: Supplementary file 13 [file Data_Sheet_13.ZIP › FIG-6H/SW1116/membrane-LIN28A.tif]

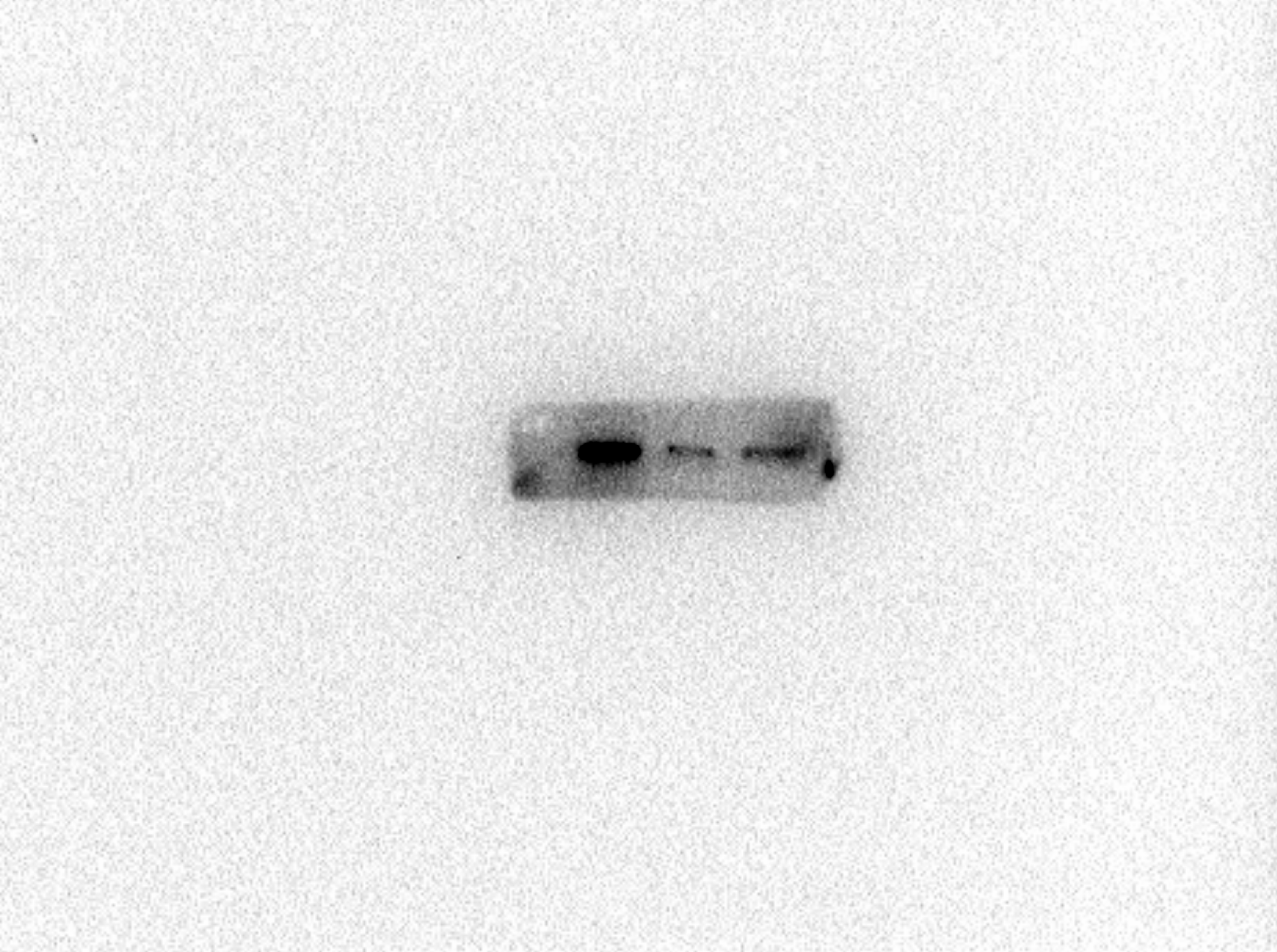

Supplement: Supplementary file 13 [file Data_Sheet_13.ZIP › FIG-6H/SW1116/membrane-METAP2.tif]
